# Genome-wide identification and expression profile analysis of nuclear factor Y family genes in *Sorghum bicolor* L. (Moench)

**DOI:** 10.1371/journal.pone.0222203

**Published:** 2019-09-19

**Authors:** P. Maheshwari, Divya Kummari, Sudhakar Reddy Palakolanu, U. Nagasai Tejaswi, M. Nagaraju, G. Rajasheker, G. Jawahar, N. Jalaja, P. Rathnagiri, P. B. Kavi Kishor

**Affiliations:** 1 Department of Genetics, Osmania University, Hyderabad, India; 2 International Crops Research Institute for the Semi-Arid Tropics (ICRISAT), Patancheru, Hyderabad, India; 3 Department of Biotechnology, Vignan’s Foundation for Science, Technology and Research, Vadlamudi, Guntur, Andhra Pradesh, India; 4 Department of Biochemistry, ICMR-National Institute of Nutrition, Hyderabad, India; 5 Genomix CARL Pvt. Ltd. Rayalapuram Road, Pulivendula, Kadapa, Andhra Pradesh, India; 6 Genomix Molecular Diagnostics Pvt Ltd., Kukatpally, Hyderabad, India; 7 Genomix Biotech Inc., Atlanta, GA, United States of America; ICAR - National Research Center on Plant Biotechnology, INDIA

## Abstract

Members of the plant Heme Activator Protein (HAP) or NUCLEAR FACTOR Y (NF-Y) are trimeric transcription factor complexes composed of the NF-YA, NF-YB and NF-YC subfamilies. They bind to the CCAAT box in the promoter regions of the target genes and regulate gene expressions. Plant NF-Ys were reported to be involved in adaptation to several abiotic stresses as well as in development. *In silico* analysis of *Sorghum bicolor* genome resulted in the identification of a total of 42 *NF-Y* genes, among which 8 code for the *SbNF-YA*, 19 for *SbNF-YB* and 15 for the *SbNF-YC* subunits. Analysis was also performed to characterize gene structures, chromosomal distribution, duplication status, protein subcellular localizations, conserved motifs, ancestral protein sequences, miRNAs and phylogenetic tree construction. Phylogenetic relationships and ortholog predictions displayed that sorghum has additional *NF-YB* genes with unknown functions in comparison with *Arabidopsis*. Analysis of promoters revealed that they harbour many stress-related *cis*-elements like ABRE and HSE, but surprisingly, DRE and MYB elements were not detected in any of the subfamilies. *SbNF-YA1*, *2*, and *6* were found upregulated under 200 mM salt and 200 mM mannitol stresses. While *NF-YA7* appeared associated with high temperature (40°C) stress, *NF-YA8* was triggered by both cold (4°C) and high temperature stresses. Among *NF-YB* genes, *7*, *12*, *15*, and *16* were induced under multiple stress conditions such as salt, mannitol, ABA, cold and high temperatures. Likewise, *NF-YC 6*, *11*, *12*, *14*, and *15* were enhanced significantly in a tissue specific manner under multiple abiotic stress conditions. Majority of the mannitol (drought)-inducible genes were also induced by salt, high temperature stresses and ABA. Few of the high temperature stress-induced genes are also induced by cold stress (NF-YA2, 4, 6, 8, NF-YB2, 7, 10, 11, 12, 14, 16, 17, NF-YC4, 6, 12, and 13) thus suggesting a cross talk among them. This work paves the way for investigating the roles of diverse sorghum NF-Y proteins during abiotic stress responses and provides an insight into the evolution of diverse NF-Y members.

## Introduction

Nuclear Factor Y (NF-Y), also known as heme activator protein (HAP) or CCAAT-binding factor (CBF) is a ubiquitous, complex, heterotrimeric transcription factor. It is evolutionarily conserved in all plants with three distinct subunits called NF-YA or HAP2, NF-YB or HAP3/CBF-A and NF-YC or HAP5/CBF-C [1]. The assembly of NF-Y is complex and occurs both in cytoplasm and nucleus. While NF-YA and NF-YC family members have a nuclear localization signal (NLS), NF-YB members generally lack the same and hence cannot be transported to the nucleus [2]. The NF-YA subunits are localised to the nucleus and bind with varying affinities to the *CCAAT cis*-elements in the promoter regions of the target genes [3, 4]. On the other hand, NF-YB and NF-YC subunits contain the conserved Histone Fold Domain (HFD) or Histone Fold Motif (HFM) and help in protein-DNA and protein-protein interactions [5, 4]. The HFD domain of NF-YB/YC is formed by 3 α-helices separated by 2 loops [6]. The α1-helices of both NF-YB and YC contain putative DNA-binding domains [4]. Via the HFDs, NF-YB and NF-YC form a heterodimer [5], which is critical for the translocation of NF-YB from the cytoplasm to the nucleus [7]. Several members of the NF-Y subfamilies play a vital role not only in a wide array of developmental processes but in tolerance to abiotic stresses as well. For example, they are involved in embryogenesis [8], ABA response and seed germination [9], abiotic stress tolerance [10, 11, 12], flowering time [13], primary root elongation [14], photosynthesis [15], endosperm development [16], and photomorphogenesis [17]. On the other hand, in leguminous plants, they are the key regulators of symbiotic root nodule development [18]. Ni et al. [19] reported that *GmNF-YA3*, a target gene of miR169, is a positive regulator of plant tolerance to drought stress in *A*. *thaliana*. Alam et al. [20] demonstrated that overexpression of *OsHAP2E* gene confers tolerance to drought and salt stresses with enhanced photosynthesis and tiller (stems produced in grass plants) number in comparison with wild-type rice plants. Transgenic rice showed resistance to *Magnaporthe oryzae* and *Xanthomonas oryzae* infections. While overexpression of *NF-YA5* conferred drought stress tolerance in *Arabidopsis* [21], *NF-YA1* in *Arabidopsis* resulted in post germinative growth arrest under salt stress [11]. Further, incorporation of several *NF-YB* and *NF-YC* genes improved drought stress tolerance in diverse plants like *Arabidopsis*, maize, poplar and rice [10, 22, 23, 24]. Thus, evidence is accumulating that *NF-Y* subunits act as key regulators of drought stress tolerance. In plants, *NF-Y* gene families comprise several paralogs. In *Triticum aestivum*, 37 paralogs have been described (10 *NF-YA*, 11 *NF-YB*, 14 *NF-YC* and 2 *Dr1*) [25]; in rice 28 *NF-Ys* (10 *NF-YA*, 11 *NF-YB* and 7 *NF-YC*) [26]; but later [27] reported 34 members in the same species. In *Arabidopsis thaliana*, *NF-Y* members exclude *AtNF-YC11*, *B12*, *B13* (*NC2* subfamily) and *AtNF-YC10*, *C13*, and *B11* (*Dpb3/4* subfamily), but include *AtNF-YC12*. Accordingly, Petroni et al. [3] pointed out that *Arabidopsis* contains 30 members of the *NF-Y* family, 10 from each family (*NF-YA*, *NF-YB* and *NF-YC*) though 36 were originally reported Siefers et al [13]. So, in the updated scheme, 30 members of *NF-Y* have been considered by Zhao et al. [28] in *A*. *thaliana*. In *Brachypodium distachyon* 36 (7 *NF-YA*, 17 *NF-YB*, and 12 *NF-YC*) [29]; in *Brassica napus* 33 (14 *NF-YA*, 14 *NF-YB*, 5 *NF-YC*) [30]; in *Setaria italica* 39 (10 *NF-YA*, 15 *NF-YB* and 14 *NF-YC*) [31]; in *Glycine max* 68 (21 *NF-YA*, 32 *NF-YB*, 15 *NF-YC*) [32]; in *Prunus mume* 29 [33]; in *Ricinus communis* 25 (6 *NF-YA*, 12 *NF-YB* and 7 *NF-YC*) [34]; in *Citrus sinensis* and *C*. *clemantia* 22 (6 *NF-YA*, 11 *NF-YB*, 5 *NF-YC*) [35] were characterised.

*Sorghum bicolor* is a semi-arid and the second most important staple food grain crop. It provides feed, fodder and fuel and shows genetic diversity [36]. Being a C_4_ photosynthetic plant, it is adapted to moderate drought and high temperature. But, salinity and drought coupled with high temperature limit the production and yield stability in sorghum. Enhancing the final yields and productivity of crop plants is especially challenging to the researchers due to the unpredictable nature of drought stress conditions during the growing season and complex drought stress biology [37]. Identification and expression of various transcription factors for abiotic stress tolerance using qRT-PCR and their validation by overexpression or knockouts is therefore critical for developing improved crop varieties with tolerance to water limited conditions. Members of *NF-Y* subfamilies impart tolerance to a very wide spectrum of both biotic and abiotic stresses as mentioned above. The number of *NF-Y* genes that exist in sorghum and their detailed biological roles for multiple stress tolerance and ABA-responsiveness remains unexplored. In this study, we identified 42 *NF-Y* genes using *in silico* approaches and examined their expression patterns under salt, drought, ABA, cold and high temperature stresses. Our gene expression studies reveal that majority of *NF-Y* genes (39) exhibited response to high temperature stress. A large number of them (24) were also expressed under multiple stresses like cold, salt (22) and drought (20). Further, 20 *SbNF-Ys* showed upregulation under ABA stress which indicates their role in ABA-related pathway. Keeping in view of the aforementioned reasons, we aimed to understand how the *SbNF-Y* members regulate abiotic stresses in an ABA-dependent or independent manner which would further delve into investigating their detailed roles during stress.

## Materials and methods

### Plant material and abiotic stress treatments

*Sorghum bicolor* variety BTx623 is an agronomically important inbred line. It is a model variety with known genome sequencing information and moderately tolerant to drought stress. The gene space of the sorghum genome sequence has also been updated by resequencing [38]. Keeping these criteria in mind, seeds of *S*. *bicolor* variety BTx623 were obtained from ICRISAT, Patancheru, Hyderabad, and sown in pots filled with 5 kg of black soil and seedlings were grown in glass house conditions at 28/20°C day/night temperatures. Sixty-day-old seedlings were subjected to 200 mM NaCl solution, 200 mM mannitol solution, and 100 μM ABA for 4 h separately. Cold stress was imposed by keeping the plants at 4°C and high temperature stress by exposing the plants to 40°C for 4 h. Corresponding controls (without any treatment) were maintained under identical conditions. After 4 h of exposure, roots, stems and leaves were collected from treated and control plants and snap frozen in liquid nitrogen and stored at -80°C for subsequent use. Three biological and three technical replicates were used for qRT-PCR analysis.

### Identification and characterization of NF-Y transcription factors

*NFY* gene sequences of *Oryza*, *Zea*, *Setaria* were retrieved from plantTFDB (http://planttfdb.cbi.pku.edu.cn/) (S1 Table) database and searched against *Sorghum bicolor* genome in Gramene database (http://www.gramene.org/) to find out their homologs. Genscan (http://genes.mit.edu/GENSCAN.html) program was used to retrieve the gene and their respective protein sequences. The identified putative *Sorghum* nuclear factors were scanned using HMMER (https://www.ebi.ac.uk/Tools/hmmer/search/hmmsearch) corresponding to the Pfam database and queried against the *Oryza*, *Setaria* and *Zea*. The identified *Sorghum NF-Ys* were confirmed by searching against *Oryza*, *Setaria* and *Zea* genomes in Gramene database (http://www.gramene.org/). Based on homology, the identified putative protein sequences were subjected to Motif Search (http://www.genome.jp/tools/motif/) analysis to check the reliability and to identify their conserved domains [39]. The identified *NF-Y* genes were mapped to their respective chromosomes based on the information provided in the Gramene Genome Database by employing MapInspect software (https://mapinspect.software.informer.com/). Gene Structure Display Server (http://gsds.cbi.pku.edu.cn) software was used for obtaining the *NF-Y* gene structures—exons, introns, and untranslated sequence regions (UTRs) based on the alignments of their coding sequences [40]. MEME software was employed to analyze new sequence patterns and their significance [41]. The software helps to identify the nature of motifs by setting different default parameters, number of motifs from 1–10 with a motif width of 5–50, and the number of motif sites from 5–10.

### NF-Y protein analysis, prediction of potential *cis*-regulatory elements, identification of miRNA target sites and phylogenetic analysis of NF-Ys

Molecular weight (MW), isoelectric point (pI), and GRAVY (grand average of hydropathy) of NF-Ys were identified for all NF-Y proteins by using ProtParam of Expasy tools (http://web.expasy.org/protparam) [42]. Phosphorylation sites of proteins were predicted using NetPhos3.1 software of Expasy tools [43]. Subcellular localization of NF-Y proteins was carried out by WOLFPSORT program (http://wolfpsort.org/) [44]. To predict the putative *cis*-acting elements of *NF-Y* promoter regions, 2000 bp genomic sequences upstream of start codons were analysed using PLANTCARE software [45]. The pSRNATarget software [46] was employed to identify the potential miRNA target sites in identified *SbNF-Ys*. Finally, the neighbor-joining (NJ) phylogenetic tree was constructed with the NF-Y protein sequences of *Sorghum bicolor* with the plants as shown in S1 Table using MEGA 6.2 software [47]. The NJ is a recursive algorithm, a fast method which is suited for large datasets and does not require ultra metric data and permits correction for multiple substitutions. The Poisson correction, pairwise deletion, and bootstrap value (1,000 replicates) parameters were used to draw the NJ phylogenetic tree.

### Phylogenetic divergence and co-expression analysis

Gene duplication events were found [48, 49] using phylogenetic tree based on 70% similarity and 80% coverage of sequences aligned. PAL2NAL program [50] was followed for finding out synonymous and non-synonymous substitutions rates. Protein-protein interaction (PPI) map of NF-Y proteins was generated from the STRING database [51].

### RNA isolation and quantitative real-time PCR analysis

From the stress exposed and control (without any stress) samples, total RNA was isolated using Macherey-Nagel NucleoSpin RNA plant kit by following the instructions given in the manual. To eliminate any genomic DNA contamination in the RNA samples, the purity of RNA was checked using Eppendorf BioPhotometer. Two micrograms of RNA sample was used as template for first strand cDNA synthesis using RevertAid First Strand cDNA Synthesis Kit (#K1622, Thermo Scientific EU, Reinach, Switzerland). To find out the relative gene expression levels of *SbNF-Ys*, 2X Applied Biosystems (ABI) Master Mix with gene specific primers was used (S2 Table). For qRT-PCR analysis, thermal cycling conditions of 95°C for 5 min followed by 40 cycles of 95°C for 30 s, 57°C for 30 s and 72°C f or 30 s were applied to the ABI 7500 real-time PCR system (Applied Biosystems, Foster City, CA, USA). Expression of *SbNF-Y* genes in control and treated samples was normalized with *EIF4a* (Eukaryotic Initiation Factor 4A) and *PP2A* (protein phosphatase2A subunit A3) reference genes [52]. qRT-PCR was carried out with three biological and three technical replicates for each sample. The PCR reaction specificity was confirmed by melting curve analysis of the amplicons. Comparative 2-DDCT method [53] was used to calculate the relative quantities of each transcript.

## Results

### Identification and characterization of SbNF-Y transcription factors, motif analysis and subcellular localization

**Fig 1:**
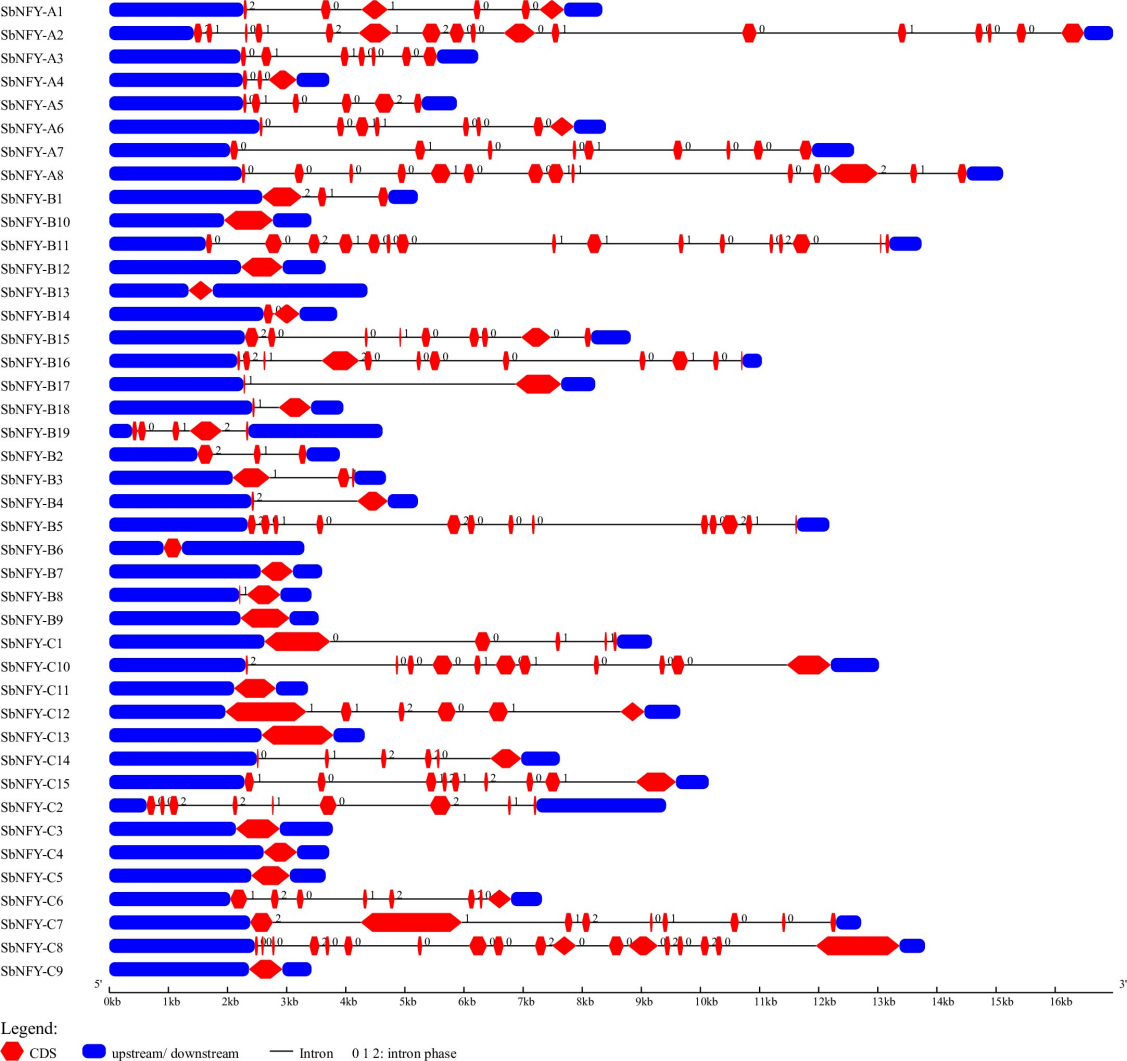
Gene structure analysis of *SbNFYs* (A. *SbNF-YA*; B. *SbNF-YB* and C. *SbNF-YC*).

**Fig 2:**
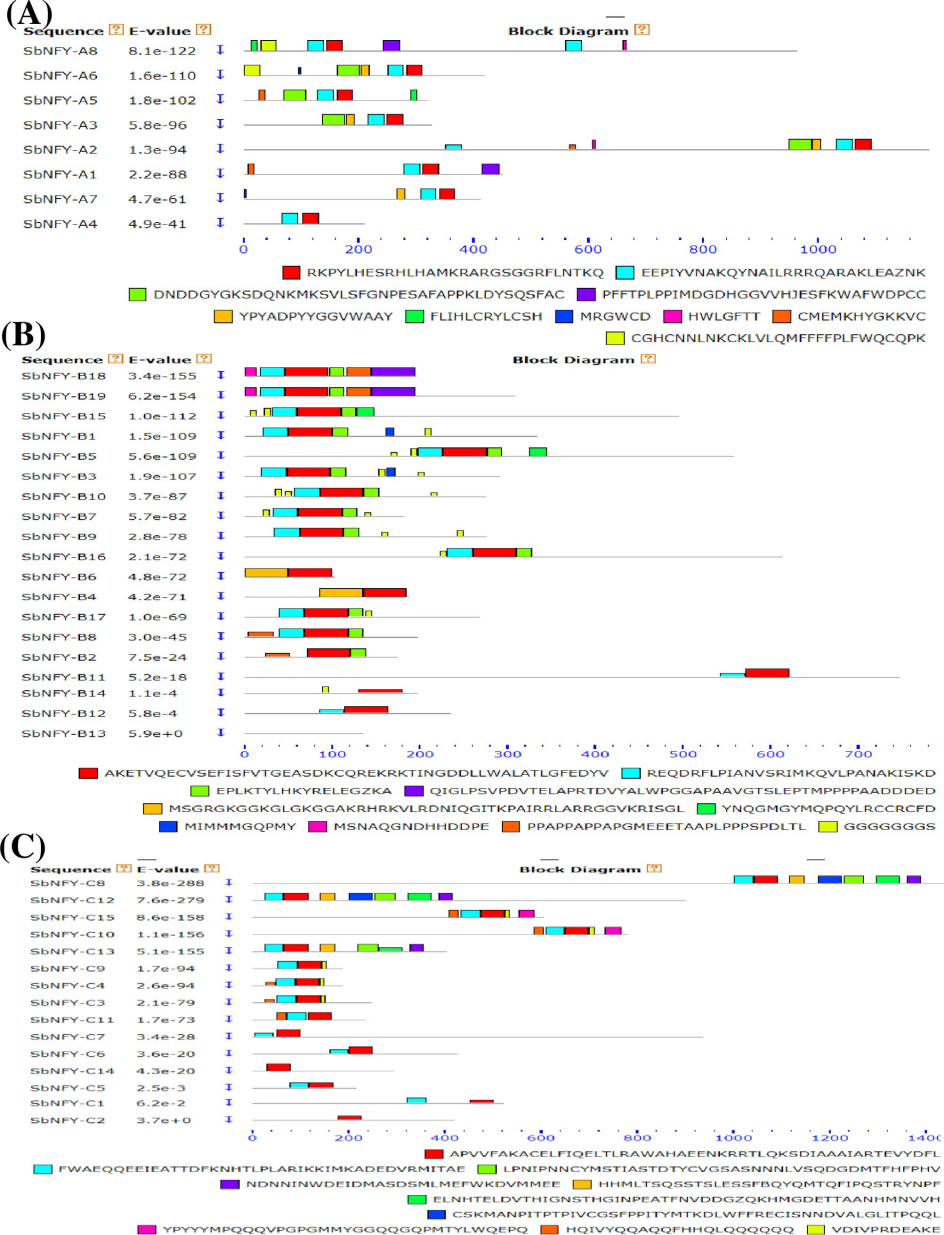
**Distribution of 1–10 conserved motifs in A) *Sb*NF-YA, B) *Sb*NF-YB and C) *Sb*NF-YC groups.** Gene clusters and p values are shown on the left side and motif sizes at the bottom of the figure.

A total of 42 homologous genes comprising 8 *NF-YA*, 19 *NF-YB* and 15 *NF-YC* from the whole genome of sorghum were identified and confirmed. Later, they were crosschecked by using the HMM profile and searching *SbNF-Ys* against *Oryza*, *Setaria* and *Zea* for further confirm to check their reliability (S3 Table). The predicted 8 *NF-YA*, 19 *NF-YB* and 15 *NF-YC* genes were named as *SbNF-YA1* to *SbNF-YA8*, *SbNF-YB1* to *SbNF-YB19* and *SbNF-YC1* to *SbNF-YC15* respectively. Based on the presence of conserved NF-YA, NF-YB and NF-YC domains, the predicted *Sb*NF-Y family of proteins was considered for identification as a member. The exon-intron structures of all the 42 annotated *NF-Y* genes were analysed (Fig 1). While 17 exons and 16 introns (highest) were detected in *SbNF-YA2*, 3 exons (least) and 2 introns were found in *SbNF-YA4* gene. Among the *SbNF-YB* family members, *SbNF-YB11* showed a maximum of 16 exon and 15 intron regions, and 5 of the members displayed 1 intron. A maximum of 18 exons and 17 introns were noticed on *SbNF-YC8*. Also, six of the members were intronless and no member exhibited one intron (Fig 1). The sub-cellular localization of *Sb*NF-Y proteins based on consensus sequence showed a majority of them to be localized to nucleolus and chloroplast although a few of them localized to cytoplasm, mitochondria and plastids (Table 1). All the NF-Ys showed nuclear localization signals (NLS); NF-YA holding LRRR sequence (motif 2 in Fig 2A), KRK motif in NF-YB (motif 1 in Fig 2B) and KRR in NF-YC (motif 1 in Fig 2C). Though they contain nuclear localization signals, their subcellular localizations were different. Majority of the *Sb*NF-YAs showed chloroplast as the important target site. Few of them have been found localized in chloroplasts (NF-YA1, NF-YA7 and NF-YA8), mitochondria (NF-YA4) and plastid (NF-YA6). The number of phosphorylation sites in each NF-Y protein is represented in the S3 Table. All the NFYs exhibited higher number of PKC than CK1, CK2, and PKA types. The PKC number is higher in NF-YA subfamily members than in NF-YB (S4 Table). No transmembrane helices were observed except in NF-YA3 protein.

**Table 1:** List of identified *SbNF-Ys* exhibiting chromosomal location, sub group, length, DNA binding domains (DBD), molecular weight (MW), iso-electric point (pI), GRAVY, number of exons, protein localization, and instability index.

S. No.	Accession	Accession No.	Common name	Chr. No.	Chromosome location	No. of AA	DBD	Sub Cellular Loc.	Exons	pI/Mw	Instability index	Gravy
1	SORBI_001G154500	XM_021451284	SbNF-YA1	1	12361535–12401644	451	277–338	Cp	6	9.96/47716.26	64.18	-0.397
2	SORBI_001G340200	XM_021450885	SbNF-YA2	1	55567388–55607555	1193	1030–1091	N	17	9.17/128227.55	51.38	-0.496
3	SORBI_001G486000	XM_021451588.	SbNF-YA3	1	68468423–68508558	328	215–276	Nr	7	8.63/35632.63	57.35	-0.804
4	SORBI_002G038500	XM_002461436.2	SbNF-YA4	2	3757416–3797476	211	68–126	M	3	9.96/22831.65	61.43	-0.601
5	SORBI_002G370800	XM_021453812.1	SbNF-YA5	2	72920433–72960535	320	128–189	N	6	7.70/35053.98	57.87	-0.749
6	SORBI_004G316500	XM_021458677.1	SbNF-YA6	4	64535410–64575508	420	64–75	P	8	9.73/46774.51	55.73	-0.290
7	SORBI_008G168300	XM_002443504.2	SbNF-YA7	8	52864314–52904375	413	306–367	Cp	9	9.37/45256.77	69.37	-0.651
8	SORBI_008G174600	XM_021445781.1	SbNF-YA8	8	53546204–53586336	964	110–171	Cp	14	6.32/105695.33	50.78	-0.385
9	SORBI_001G338700	XM_002467650.2	SbNF-YB1	1	55430499–55470820	334	26–91	N	3	9.17/35602.50	54.35	-0.806
10	SORBI_002G135100	XM_021454561.	SbNF-YB2	2	20095150–20135252	174	40–112	N	3	5.03/ 19169.40	64.54	-0.902
11	SORBI_002G369800	XM_002463118.2	SbNF-YB3	2	72839156–72879413	291	24–89	N	2	6.97/31189.68	58.08	-0.750
12	SORBI_003G057000	XM_002455085.2	SbNF-YB4	3	5048009–5088028	188	114–176	Cp	2	11.35/20827.56	49.55	-0.399
13	SORBI_003G346500	XM_021456401.1	SbNF-YB5	3	66741363–66781566	559	202–267	N	12	9.26/ 60688.71	48.11	-0.479
14	SORBI_003G347600	XM_002456551	SbNF-YB6	3	66829905–66870081	103	56–91	56–91	N	1	11.48/11409.38	45.35	-0.524
15	SORBI_003G417700	XM_002459011	SbNF-YB7	3	72352365–72392627	182	37–102	M	1	6.15/19094.22	38.54^#^	-0.598
16	SORBI_004G254400	XM_002452581.2	SbNF-YB8	4	59362750–59403321	197	44–109	N	1	8.93/21087.66	48.67	-0.729
17	SORBI_004G254500	XM_002452582.2	SbNF-YB9	4	59389510–59429805	276	39–104	N	1	6.37/29163.20	43.89	-0.601
18	SORBI_007G059500	XM_002445097.2	SbNF-YB10	7	6167326–6207663	275	62–127	Cp/N,	1	6.00/ 27666.53	30.35^#^	-0.365
19	SORBI_007G117100	-	SbNF-YB11	7	49511493–49551626	792	551–612	Cp	15	6.21/87581.38	57.79	-0.255
20	SORBI_007G070200	-	SbNF-YB12	7	7554666–7594741	235	90–155	N	1	4.72/26256.09	60.01	-0.697
21	SORBI_009G152900	-	SbNF-YB13	9	50928726–50968746	136	65–128	N	1	11.29/15267.90	43.01	-0.551
22	SORBI_009G164000	XM_002439878.2	SbNF-YB14	9	52047607–52087876	197	115–170	N	2	10.29/21673.26	45.05	-0.719
23	SORBI_009G166200	XM_021446873.1	SbNF-YB15	9	52266773–52306916	496	36–101	N	9	9.73/55777.04	57.67	-0.313
24	SORBI_009G239600	XM_021446907.1	SbNF-YB16	9	57726747–57766909	613	236–301	Cp	11	9.62/68143.71	55.72	-0.587
25	SORBI_010G119200	XM_002436840.2	SbNF-YB17	10	13365025–13405651	268	44–109	C	2	6.25/28239.54	58.70	-0.353
26	SORBI_010G029500	XM_002437749.1	SbNF-YB18	10	2373338–2413887	196	22–87	M	2	4.34/21156.69	66.56	-0.464
27	SORBI_010G029700	XM_002437749.1	SbNF-YB19	10	2381943–2421964	309	22–83	Cp	5	4.94/33884.66	57.17	-0.223
28	SORBI_001G009200	XM_021451803	SbNF-YC1	1	838611–879724	523	340–370	N	5	10.74/55002.37	68.32	-1.128
29	SORBI_001G094500	XM_021450704	SbNF-YC2	1	7233252–7273270	421	154–218	M	9	6.23/45665.93	49.04	-0.329
30	SORBI_001G435500	XM_021450884.	SbNF-YC3	1	64180902–64221327	247	69–133	N	1	5.31/26178.44	64.40	-0.349
31	SORBI_002G241500	XM_002460379	SbNF-YC4	2	63046266–63086603	188	68–131	N/C	1	5.36/ 20002.74	53.09	-0.078
32	SORBI_003G040500	XM_002457181	SbNF-YC5	3	3740425–3780678	217	97–158	N	1	4.61/ 22949.36	60.31	-0.636
33	SORBI_005G143400	XM_002449602.2	SbNF-YC6	5	51132033–51172245	426	182–242	P	8	9.08/ 46629.63	62.72	-0.627
34	SORBI_006G272400	XM_002448754	SbNF-YC7	6	61309584–61349970	936	24–92	Cp	9	8.85/103880.62	43.92	-0.137
35	SORBI_007G054700	XM_002445073	SbNF-YC8	7	5489299–5530378	1437	1020–1083	N/C	18	5.44/162636.39	44.04	-0.416
36	SORBI_007G219500	XM_002445904.	SbNF-YC9	7	63491753–63532358	188	72–135	N	1	5.51/20247.09	57.58	-0.273
37	SORBI_007G063200	XM_002443915.	SbNF-YC10	7	6585177–6625938	782	628–691	N	11	6.69/88215.13	65.53	-0.282
38	SORBI_007G070100	XM_021465359	SbNF-YC11	7	6585177–6625938	235	91–155	N	1	4.72/26256.09	60.01	-0.697
39	SORBI_008G043000	XM_002445073	SbNF-YC12	8	4187858–4229252	902	44–105	N	6	6.82/100376.83	49.77	-0.240
40	SORBI_008G071900	XM_021445970	SbNF-YC13	8	9604002–9644041	405	45–107	N	1	5.14/45580.38	52.43	-0.478
41	SORBI_009G181900	XM_021447763	SbNF-YC14	9	53568558–53608631	294	8–71	N	6	5.19/32113.74	38.91^#^	-0.719
42	SORBI_010G221400	XM_002437375	SbNF-YC15	10	56171840–56212135	605	452–515	C	9	9.26/67661.50	53.85	-0.348

(# stable; N: Nuclear; M: Mitochondrial; Cp: Chloroplast; P: Plastid; C: Cytoplasm).

The identified *SbNF-Y* genes encoded polypeptides with amino acids ranging from 130 to 1430 and pI values varied from 4.26 to 10.83. Characteristically, they showed DNA binding domains. Molecular weights of the proteins ranged from 10.21 to 83.52 kDa (Table 1). Among the *SbNF-YA* subfamily members, RKPYLHESRHLHAMKRARGSGGRFLNTKQ and EEPIYVNAKQYNAILRRRQARAKLEAZNK large contiguous motifs were found ubiquitous, while rest of the 8 large contiguous motifs showed variability in their distribution (see Figs 2A and S1). Similarly, *SbNF-YB* proteins revealed the uniform presence of one, highly conserved large contiguous motif, i.e. AKETVQECVSEFISFVTGEASDKCQREKRKTINGDDLLWALATLGFEDYY (Figs 2B and S2). On the other hand, analysis of NF-YC proteins revealed a highly conserved large contiguous motif APVVFAKACEFIQELTLRAWHEENKRRTLQKSSDIAAAIARTEVYDFL (see Figs 2C and S3). Motif analysis of complete *Sb*NF-Y family representing conserved motifs (S4 and S5 Figs) reflect typical diagnostic features for different subunits of NF-Y family proteins in general and hence provide confirmatory identification of *Sb*NF-Y proteins from the sorghum genome.

### Phylogeny, divergence, and physical genome mapping of SbNF-Ys

**Fig 3:**
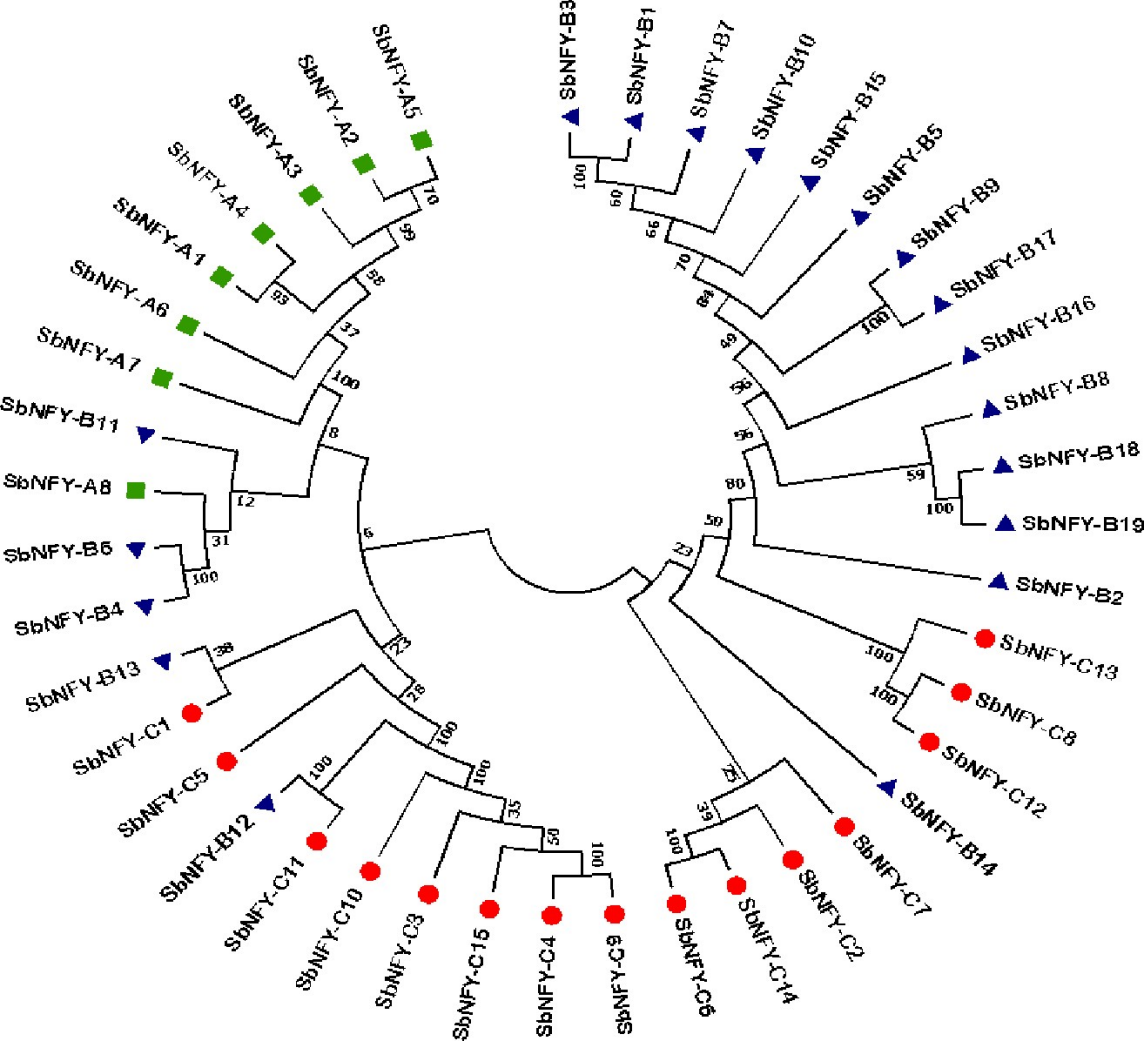
Phylogenetic tree of *SbNF-Y* gene family.

**Fig 4:**
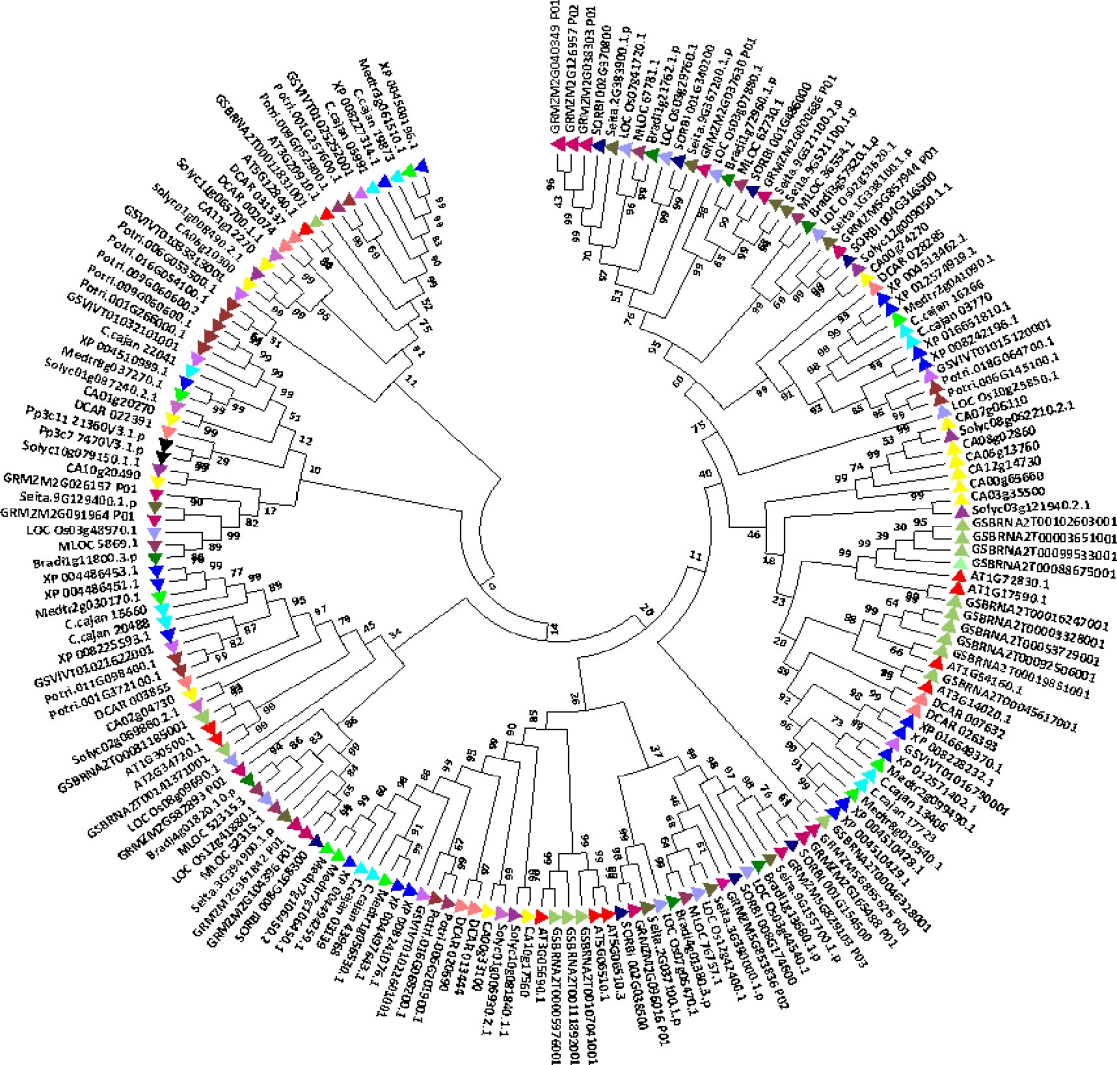
Phylogenetic tree showing the relationship between *Sorghum*, *Oryza*, *Setaria*, *Zea*, *Medicago*, *Glycine max*, *Daucus*, *Solanum*, *Brachypodium*, *Cajanus*, *Populus* and *Arabidopsis NF-YAs*.

**Fig 5:**
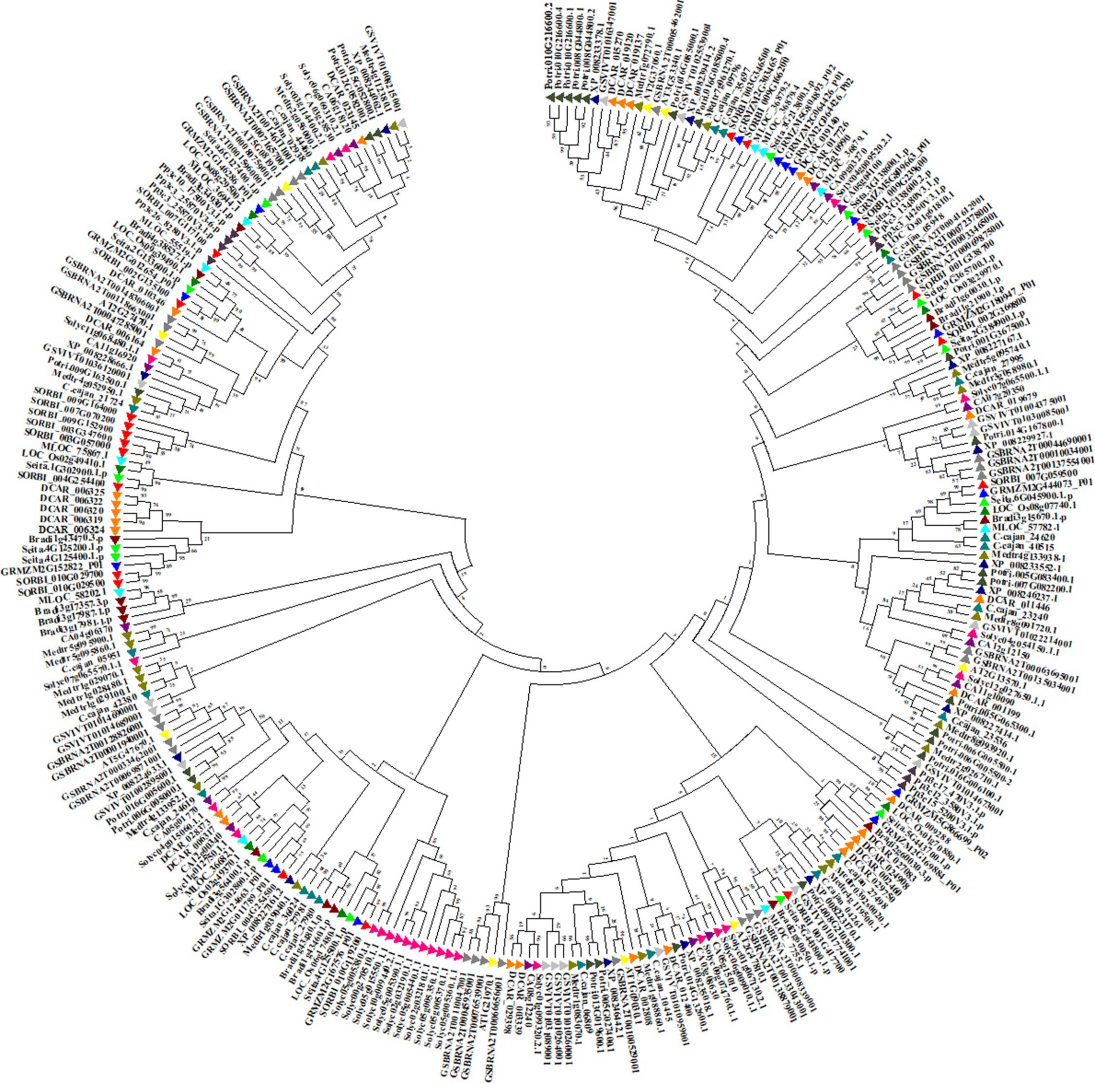
N-j phylogenetic tree representing *NF-YB* family genes of *Sorghum*, *Oryza*, *Setaria*, *Zea*, *Medicago*, *Glycine max*, *Dacus*, *Solanum*, *Brachypodium*, *Cajanus*, *Populus* and *Arabidopsis*.

**Fig 6:**
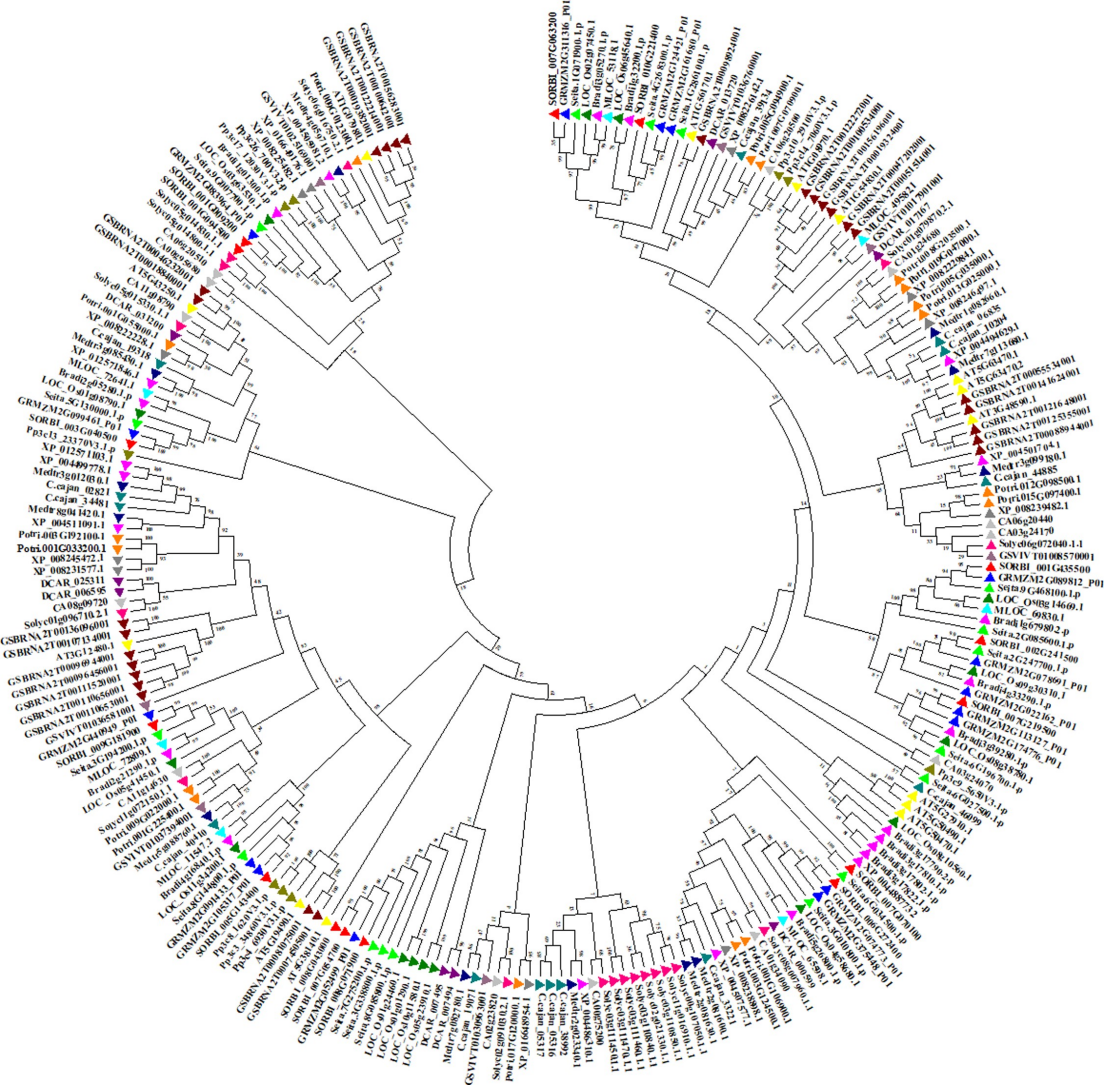
Evolutionary relationship of the NF-YC family genes of *Sorghum*, *Oryza*, *Setaria*, *Zea*, *Medicago*, *Glycine max*, *Dacus*, *Solanum*, *Brachypodium*, *Cajanus*, *Populus* and *Arabidopsis*.

The phylogenetic tree of *Sb*NF-Y proteins was constructed using MEGA 6.2 software. It showed 2 clades, which are subdivided into 4. The phylogenetic analysis displayed a total of 11 paralogous duplication events of which 3 tandem/segmental (*SbNF-YB4/SbNF-YB6*; *SbNF-YB18/SbNF-YB19*; *SbNF-YB12/SbNF-YC11*) and remaining regional duplication events. Interestingly, NF-YBs exhibited paralogous events with *SbNF-YCs* (*SbNF-YB13/SbNF-YC1*; *SbNF-YB12/SbNF-YC11*), indicating their evolutionary relatedness (Figs 3 and S6 and Table 2). To find out the orthologous and the evolutionary relationships of *SbNF-Ys*, *NF-YA*, *NF-YB* and *NF-YCs* have been compared with other plant genomes (see Fig 4A, 4B and 4C respectively). Not surprisingly, a majority of the identified *SbNF-Ys* showed orthologous relationship with *Zea*, few with *Setaria* and one with *Hordeum* (Sorbi009G166200 (SbNF-YB15)/MLOC_36879.2). Of the 8 *SbNF-YAs*, 5 showed orthologous events with *Zea* and 1 with *Setaria* (Fig 4 and S5 Table). The major subfamily *SbNF-YBs* exhibited 11 orthologous events, of which 6 showed with *Zea*, 4 with *Setaria* and 1 with *Hordeum* (Fig 5 and S6 Table). On the other hand, *SbNF-YCs* showed 10 orthologous events, of which 8 with *Zea* and 2 with *Setaria* (Fig 6 and S7 Table).

**Table 2:** Calculation of non-synonymous to synonymous substitution ratios in paralogs of *SbNFYs*.

Sorghum	Chr.	Paralog	Chr.	No. of non-synonymous sites (N)	No. of synonymous sites (S)	Non-synonymous substitution rate (d_N_)	Synonymous substitution rate (d_S_)	d_N_/d_S_
*SbNF-YA1*	1	*SbNF-YA4*	2	494.0	139.0	8.3861	29.9357	0.2853
*SbNF-YA2*	1	*SbNF-YA5*	2	671.3	288.7	16.3841	5.1317	3.1927
*SbNF-YB1*	1	*SbNF-YB3*	2	711.0	162.0	10.1591	1.1361	8.9424
*SbNF-YB4*	3	*SbNF-YB6*	3	257.0	52.0	14.1077	7.5273	1.8742
*SbNF-YB9*	4	*SbNF-YB17*	10	665.9	138.1	3.0355	2.4629	1.2325
*SbNF-YB18*	10	*SbNF-YB19*	10	364.5	223.5	0.0000	0.0045	0.0010
*SbNF-YB13*	9	*SbNF-YC1*	1	343.6	64.4	2.5270	27.0498	0.0934
*SbNF-YB12*	7	*SbNF-YC11*	7	567.0	138.0	0.0000	0.0000	0.4439
*SbNF-YC4*	2	*SbNF-YC9*	7	461.7	102.3	1.5957	3.5070	0.4550
*SbNF-YC6*	5	*SbNF-YC14*	9	610.2	271.8	17.0741	3.8549	4.4292
*SbNF-YC8*	7	*SbNF-YC12*	8	2181.7	524.3	16.0833	0.1711	93.9760

(d_N_/d_S_ >1 = Positive or Darwinian Selection (Driving Change); d_N_/d_S_ <1 = Purifying or Stabilizing Selection (Acting against change); d_N_ /d_S_ = 1 Neutral Selection.

**Fig 7:**
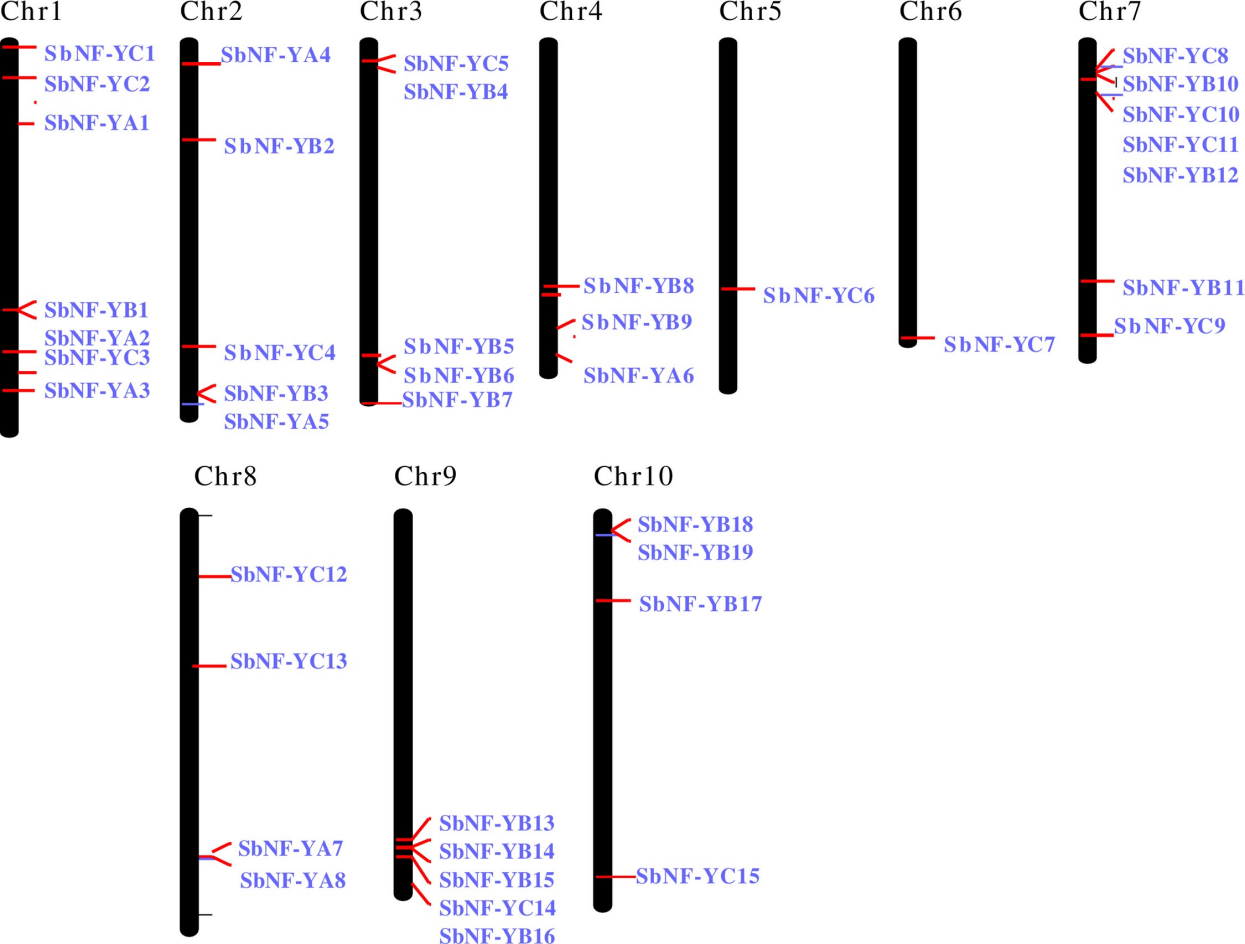
Chromosomal location of *NF-Y genes in Sorghum*.

The identified *SbNF-Ys* were distributed across all the 10 chromosomes. A maximum number of 7 genes each were located on chromosome 1, and 7, 5 genes each on 2, 3, and 9, 4 each on 8 and 10, 3 genes on 4, and 1 each on 5 and 6 chromosomes (Fig 7 and Table 1). Among the *NF-YA* subfamily, *SbNF-YA1*, *SbNF-YA2* and *SbNF-YA3* genes were located on chromosome 1. Chromosomes 2 and 8 have two genes each located on them, i.e. *SbNF-YA4*, *SbNF-YA5* and *SbNF-YA7*, *SbNF-YA8* respectively. Chromosome 4 is having only *SbNF-YA6* localized on it. Among the *SbNF-YB* genes, a maximum of 4 genes each were located on chromosomes 3 and 9. While *SbNF-YB4*, *SbNF-YB5*, *SbNF-YB6* and *SbNF-YB7* were observed on chromosome 3, *SbNF-YB13*, *SbNF-YB14*, *SbNF-YB15* and *SbNF-YB16* were noticed on chromosome 9. Three genes each *SbNF-YB10*, *SbNF-YB11*, *SbNF-YB12* and *SbNF-YB17*, *SbNF-YB18*, *SbNF-YB19* were seen on chromosomes 7 and 10 respectively. Chromosomes 2 and 4 have 2 genes each, i.e., *SbNF-YB2* and *SbNF-YB3* on 2, *SbNF-YB8* and *SbNF-YB9* on 4. Chromosome 1 has only *SbNF-YB1* located on it. Majority of the *SbNF-YC* genes were located on chromosome 7, and it accommodates 4 genes (*SbNF-YC8*, *SbNF-YC9*, *SbNF-YC10*, and *SbNF-YC11*). While chromosome 1 accommodates three genes (*SbNF-YC1*, *SbNF-YC2*, and *SbNF-YC3*), chromosome 8 contains *SbNF-YC12* and *SbNF-YC13*. One gene each *SbNF-YC4*, *SbNF-YC5*, *SbNF-YC6*, *SbNF-YC7*, *SbNF-YC14*, and *SbNF-YC15* was noticed on chromosomes 2, 3, 5, 6, 9, 10 respectively (Fig 7 and Table 1).

### Estimation of non-synonymous and synonymous substitution rates

The non-synonymous (d_N_) and synonymous (d_S_) substitution (d_N_/d_S_) rates were calculated for genes which showed duplication events within *Sorghum* as paralogs and between other genomes as orthologs. The 11 paralogs (S6 Fig) exhibited the d_N_/d_S_ between 0.0010 (*Sb*NF-YB18/*Sb*NF-YB19)-93.9760 (*Sb*NF-YC8/*Sb*NF-YC12). Of the 11 paralogs, only 5 showed the purifying/stabilizing selection (<1), while the remaining exhibited positive/Darwinian selection (>1) (Table 2). The *SbNF-YA* orthologs exhibited d_N_/d_S_ rates ranging from 0.4000 (Sorbi001G340200/Seita.9G367200) and 28.6781 (Sorbi001G486000/Zm2G000686). This indicates that 2 were following purifying selection and the remaining 4 positive selection (S5 Table). The d_N_/d_S_ rates of *SbNF-YB* orthologs varied from 0.0031 (Sorbi001G338700/Seita. 9G365700) and 10.2227 (Sorbi010G119200/ZM2G167576). While majority of them (7) evolved through purifying selection, the remaining evolved by positive selection mechanism (S6 Table). The orthologs of *SbNF-YCs* showed d_N_/d_S_ rates between 0.0797 (Sorbi002G241500/Seita. 2G247700) and 1.9761 (Sorbi007G063200/ZM2G311316) (S7 Table).

### Promoter analysis

Analysis of promoter sequences revealed *cis*-acting elements such as ABA-responsive (ABRE), drought-responsive (DRE, DPBF, MYB and MYC), heat shock-responsive (HSE), and low temperature-responsive (LTR) elements. Aside, methyl jasmonic acid- (MeJA-RE), salicylic acid- (SARE), and defence-responsive elements (TC-rich repeats), associated with biotic stress were detected. Majority of the genes in *SbNF-YA* family have G-BOX (CACGTG) *cis*-acting elements (S8 Table). In the *SbNF-YB* family, G-BOX and Sp1 were observed as major *cis*-acting elements except in *SbNF-YB18* (S9 Table). On the other hand, Skn-1 motif is the most common *cis*-acting element in *SbNF-YC* family. The least expressed *cis*-acting elements in *SbNF-YC* are AUXRR-CORE and GCN4 motif I (S10 Table).

### Protein-protein interaction (PPI) prediction analysis

The PPI mapping of *Sb*NF-Ys showed that a cohort of proteins involved in various cellular, metabolic and molecular pathways are associated with miRNA surveillance pathway, DNA replication, base excision, nucleotide excision repair pathway, purine and pyramidine metabolism. They interacted with core histones, calcineurins, kelch motifs, serine-threonine phosphatases, histone lysine N-methyl transferase, and metal-dependant phosphatase (S7 and S8 Figs).

### *In silico* prediction of miRNA target sites

The *SbNF-YAs* exhibited different miRNA target sites such as sbi-miR169, sbi-miR5389, sbi-miR6225, sbi-miR5568, sbi-miR6220, sbi-miR5567 and sbi-miR6232, *SbNF-YBs* showed sbi-miR5565, sbi-miR5568, sbi-miR6232, sbi-miR6220, sbi-miR821, sbi-miR437, sbi-miR528, sbi-miR395, sbi-miR169, sbi-miR171 and sbi-miR172. Likewise, *SbNF-YCs* showed sbi-miR6232, sbi-miR6235, sbi-miR395, sbi-miR437, sbi-miR395, sbi-miR6220, sbi-miR6225, sbi-miR6227, sbi-miR5569, sbi-miR5568, sbi-miR164, sbi-miR156, sbi-miR160, sbi-miR6218, sbi-miR6230, and sbi-miR821. Interestingly, all of them are known to be associated with translation and cleavage events (S11, S12 and S13 Tables).

### Gene expression analysis of SbNF-Ys in different tissues treated with diverse abiotic stresses

**Fig 8:**
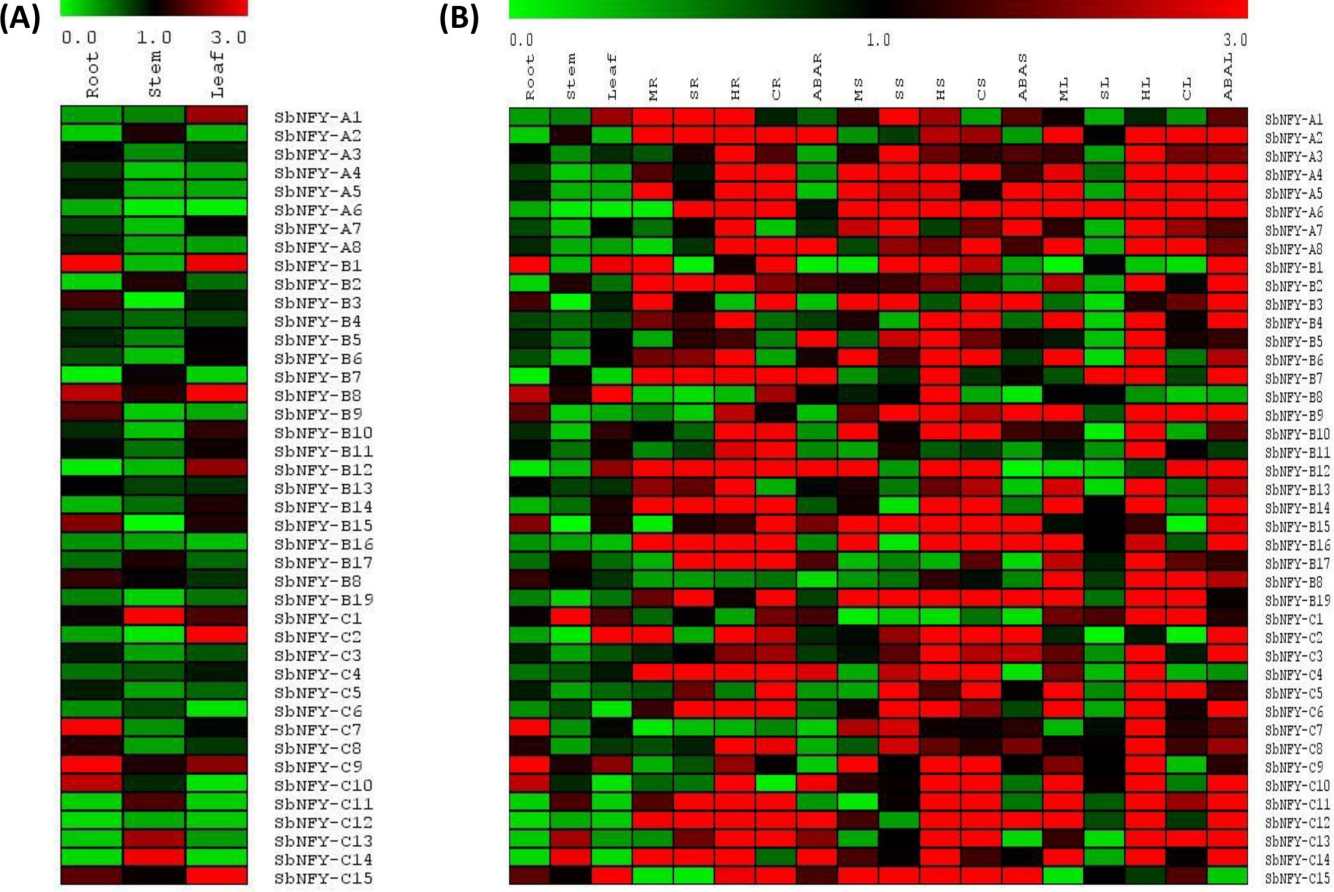
**Expression analysis of *SbNF-Ys* A), in root, stem and leaf tissues B) across diverse tissues under different abiotic stresses.** (R; Root, S; Stem, L; Leaf, MR; Mannitol Root, SR; Salt root, HR; High temperature root, CR; Cold root, ABAR; Abscisic acid root, MS; Mannitol stem, SS; Salt stem, HS; High temperature stem, CS; Cold stem, ABAS; Abscisic acid stem, ML; Mannitol leaf, SL; Salt leaf, HL; High temperature leaf, CL; Cold leaf, ABAL; Abscisic acid leaf).

Expression of all the 42 *NF-Y* family of genes was studied at the transcriptional level in different tissues and abiotic stress conditions besides ABA, and the heat map is presented in Fig 6A and 6B. Over all, the gene expressions were higher in leaf tissues in comparison with stem and root (Fig 8A and S8 Table). Among the 8 *SbNF-YA* genes, *NF-YA6* appeared to be associated with salt, drought (imposed by mannitol), cold and high temperature stresses. It was also strongly triggered by ABA. On the other hand, *NF-YA1* was induced by multiple stresses like salt, mannitol and high temperature stresses, but *NF-YA3* and *NF-YA5* were expressed only under high temperature. While salt and high temperature influenced the expression of *NF-YA7*, *NF-YA8* was upregulated by cold and high temperature stresses. Among the 19 *SbNF-YB* members, *SbNF-YB7*, *B12*, *B15* and *B16* were strongly induced by different stresses like salt, mannitol, ABA, cold and high temperature. In contrast, seven out of fifteen *SbNF-YC* members, *YC1*, *YC3*, *YC4*, *YC7*, *YC8*, *YC9*, and *YC13* were expressed only under high temperature stress. Further, five members (*YC6*, *YC11*, *YC12*, *YC14* and *YC15*) were upregulated by multiple stresses including ABA (Fig 8B and S14 Table).

## Discussion

### Identification and structural analysis of SbNF-Y genes

It is observed from this study that the number of genes are variable in each of the three distinct subfamilies of *NF-Y* (*NF-YA*, *NF-YB* and *NF-YC*) across different taxa. *NF-Ys* are evolutionarily conserved in eukaryotes and each subunit is encoded by a single gene in yeast and animals [4]. But, the same is encoded by a family of genes varying from 8 to 39 in plants. A total of 33 NF-Ys were reported in *Brassica napus* (14 *NF-YA*, 14 *NF-YB*, 5 *NF-YC*) [30]; while 39 in *Setaria italica* (10 *NF-YA*, 15 *NF-YB* and 14 *NF-YC*) [31]; whereas 68 in *Glycine max* (21 *NF-YA*, 32 *NF-YB*, 15 *NF-YC*) [32]. Multiple members of *NF-Y* subunits in plants reflect the redundancy and differentiated functions of these genes which need to be explored by expression profiling. Pereira et al. [35] identified 22 *NF-Y* genes in *Citrus sinensis* and *C*. *clementina* (6 *NF-YA*, 11 *NF-YB* and 5 *NF-YC*). Xu et al. [54] pointed out that such a low number of *NF-Y* genes found in *Citrus* genome could be due to the whole genome duplication events occurred in *A*. *thaliana* when compared to *Citrus*. It appears that subunit *YB* has more number of genes in comparison with *YA* and *YC* members in most of the species including the present report. Single genes, but with multiple splicing isoforms (that encode *NF-Y* subunits) is generally noticed in the yeast and mammals. Contrary to this, in higher plants, multigene families are noticed which can encode each subunit. Such multiple genes are vital for plant systems for tissue specific expressions at various stages of growth and development. Further, such a subunit combination can assist the plant systems in performing diverse roles during stress/development. Malaviya et al. [55] searched the plant transcription factor database (Plant TFDB, (http://plantfdb.cbi.pku.edu.cn/) version 3.0 Jin et al. [56]), for identifying *NF-Y* genes in sorghum. They identified a total of 33 NF-Y transcription factors comprising 8 *NF-YA*, 11 *NF-YB*, and 14 *NF-YC* subunits in sorghum using Plant TFDB. In contrast, in the present study, a total of 42 *NF-Y* genes, among which 8 code for *SbNF-YA*, 19 for *SbNF-YB* and 15 for *SbNF-YC* subunits were identified. This discrepancy is because, in the present study, sorghum genome sequence available in the public domain has been searched.

Koralewski and Krutovsky [57] pointed that finding out exon-intron organization is crucial since it provides an insight into evolutionary relationships among genes and organisms. Malviya et al. [55] reported no introns in 18 of the genes (out of 33), and 5 of them have only one intron. They reported 2 in *NF-YA3*, 5 in *NF-YA5*, 4 in *NF-YA6*, 3 in *NF-YA8*, 4 in *NF-YB1*, 5 in *NF-YC4* and 3 in *YC7*. Interestingly, in the present study, introns were absent in 12 out of 42 *NF-Y TFs*. While *NF-YC18* contained 17 (highest number), *YA2* 16, *YB11* 15, *YA8* 13, *YB16* 11, *YC10* 10, *YA7*, *YB15*, *YC2*, *YC7* and *YC15* 8 introns each. Only *YB4*, *YB8*, *YB14*, *YB17*, and *YB18* (in all 5) contained one intron. Like in *S*. *bicolor*, single intron genes were also noted in *Medicago truncatula* which lead to alternative spliced variants of *NF-YA1* [58]. Similarly, one intron in the 5′-UTRs of the *NF-YA* members was observed in *A*. *thaliana*, *O*. *sativa*, *C*. *sinensis* [28, 27, 35]. This suggests that such a post-transcriptional regulatory mechanism is retained among *NF-YA* genes. Single intron *NF-Ys* were not observed in *SbNF-YC* subtype in the present analysis. Chen et al. [59] reported that most of the *NF-YB* contained only one exon, and the genes from the same clade displayed a similar motif pattern in *Gossypium hirsuyum*. Chu et al. [60] reported 5 exons and 4 introns (6 genes) or 6 exons and 5 introns (2 genes) in *CaNF-YA* gene family members in *Cicer arietinum*. Further, they noticed 1 to 6 exons in *CaNF-YB* family, and 7 intronless out of 11 members in the *CaNF-YC* family. They reported 1 intron in *NF-YC1*, and 3 in *NF-YC9*. This suggests that a post-transcriptional regulatory mechanism is retained among *NF-YA* genes. Thus, the presence of multiple exon/intron gene organizations have been found in all the *NF-Y* family members in other species like *B*. *napus* [30], and *S*. *lycopersicum* [61] also. This infers that the presence of exon/intron is an attribute and typical of *NF-Ys* in higher plants. Loss or gain of spliceosomal introns led to the progress in our understanding of the molecular mechanisms associated with intron evolution and variation in gene function [62]. Fusion of exons and intron loss, might play a key role in the evolution of larger families like *NF-Ys*. Further, several members of the *NF-YB* and *NF-YC* have been found without any introns like in *S*. *bicolor* [55], *Ricinus cummunis* [34] and chickpea [60]. Introns are essential parts of all eukaryotic genes. In eukaryotic systems, introns are known to execute several functions like exon shuffling [63], gene expression alterations [64] and also tune the evolutionary rate of genes [62].

### Motif identification and chromosomal localization of SbNF-Ys

NF-Y proteins display both conserved and non-conserved regions in *Arabidopsis* and others. Such conserved sequences may be vital for DNA interactions at CCAAT sites as pointed out by Siefers et al. [13], Romier et al. [2], and Testa et al. [65]. Hahn et al. [66] demonstrated that the yeast CCAAT box factor is a heteromer that contains HAP2 and HAP3 proteins. Xing et al. [67] showed that HAP2 is a 21 residue region with 3 histidines and arginines. Both *Sb*NF-YB and *Sb*NF-YC proteins have histone domains, but not *Sb*NF-YAs. Besides, they also contain centromere kinetochore components and chromatin reorganizing domains. These residues are conserved in all the 8 *Sb*NF-YA proteins (present study) as well as in *Oryza sativa*, and *Triticum aestivum* [23, 25]. Romier et al. [2] demonstrated that NF-YC/NF-YB sub-complex interacts through histone fold motifs. The role of the alpha C-helix of NF-YC appears to be vital for trimerization as well as a target for regulatory proteins like that of MYC and p53. It looks that heterotrimeric NF-Y proteins recognize the CCAAT regulatory elements represented in promoter and enhancer regions and modulate the genes. Steidl et al. [68], Liu and Howell [69] pointed out that NF-YB and NF-YC form a dimer in the cytoplasm and then translocated to the nucleus to interact with that of NF-YA to form a heterotrimer complex. Further, it has been demonstrated that *bZIP28* and *NF-Y* transcription factors are activated by endoplasmic reticulum stress and assemble into a transcriptional complex to regulate downstream stress response genes in *A*. *thaliana* [69]. Alpha helix transmembrane spans with average hydrophobicity were predicted in 12 of the NF-Y proteins in *S*. *bicolor*. Anchoring of protein to glycosylphosphatidyl inositol (GPI) *via* the C-terminal attachment was predicted in three of the NF-YB proteins namely *Sb*NF-YB1, YB3 and YB6. This appears rational since earlier gene fusion experiments conducted by Caras et al. [70] demonstrated that the C-terminal signal sequence has GPI-anchoring residues. The distribution of *NF-Y* genes appears to be widespread among different chromosomes. While *OsHAP* genes were dispersed on 11 out of the 12 rice chromosomes [26], in *S*. *bicolor*, they are distributed on 10 chromosomes.

### Phylogenetic assessment, divergence and promoter analysis

Among the *NF-YA* family members, *A2* and *A5* appeared on the same clade indicating that they are closer to each other compared to others. While Malviya et al. [55] found that *Sb*NF-YB8 was closer to *Sb*NF-YA and *Sb*NF-YC proteins, we could not observe such a correlation. On the contrary, B12 was observed closer to C11 and B13 to C1 in the present study than YA family members. It appears therefore YB and YC members might have close correlations in comparison with other members. Malviya et al. [55] noticed several ortholog and paralog groups through the phylogenetic analysis of *Sb*NF-Y proteins along with 36 *Arabidopsis* and 28 rice NF-Y proteins. Malviya et al. [55] reported that *Sorghum NF-Y* family gene expansion is due to segmental duplication events. It appears that *SbNF-Y* genes retained their function even after duplication. Generally, gene family expansion occurs through segmental, tandem duplications, and transposition events [71]. In the present investigation, 11 paralogs were observed due to 3 regional duplications, and 8 segmental duplications, inferring that segmental duplications are responsible for *SbNF-Y* gene family expansion. Six duplication events were observed in *SbNF-YB* family, and this is a large number when compared to other subfamilies. *SbNF-YB4*, *B5*, *B13* and *B14* were phylogenetically distinct from other *SbNF-YBs*, and might have formed by recent duplications. *SbNF-Ys* exhibited 20 orthologous events with *Zea*, 7 with *Setaria* and 1 with *Hordeum*, which indicates their monocot ancestors. The synonymous (d_S_) and nonsynonymous (d_N_) substitutions reveal the selective pressure on duplicated genes. In the present study, phylogenetic relationships and ortholog predictions displayed that sorghum has additional *NF-YB* genes with unknown functions in comparison with *Arabidopsis*. The synonymous (d_S_) and nonsynonymous (d_N_) substitutions reveal the selective pressure on duplicated genes. Nekrutenko et al. [72] pointed out that greater than 1 d_N_/d_S_ value represents positive selection, less than 1 functional constraint, and equal to 1 neutral selection. In the present study, it appears that majority of the duplicated genes evolved through purifying selection. The phytohormone-responsive *cis*-acting elements make the plants to tolerate various environmental changes. The ABRE play an important role in ABA signalling and abiotic stress tolerance. In the present investigation, large number of ABA-responsive elements were observed in majority of *NF-Ys* besides Skn elements that participate in endosperm expression [73]. Further, the presence of light-responsive elements like SP1, I-Box, and G-BOX indicate their roles in the regulation of gene responses to light. Interestingly, all the elements are rich with heat shock elements (HSE), which indicates their diverse roles in various stress response mechanisms.

### miRNA analysis and protein-protein interactions

It is known that stress-responsive miRNAs target the transcription factors, which regulate the plant growth and development. The rationale behind finding out miRNA target sites is to know if any miRNAs associated in the regulation of *SbNF-Ys* exist in the genome. Identifying the target sites would subsequently help us in elucidating the regulation of *SbNF-Ys* during salt, drought and high temperature stress conditions. The miRNAs may also involve in gene networks regulated by transcription factors like *NF-Ys*. Identifying the interactions between miRNAs and transcription factors like *NF-Ys* will serve to screen their roles in stress tolerance, signal transduction, different developmental stages and synthesis of secondary metabolites, which will help to develop desired phenotypes with stress tolerance. While Fang et al. [74] reported targeting of the NAC mRNA by miRNA for abiotic stress responses, Stief et al. [75] noticed down regulation of heat stress memory by another miRNA. In the present investigation, miR169 identified was known to participate in post transcriptional regulation [76, 77, 19, 78]. Furthermore, miR169 and *NF-YA5* knockout plants showed hypersensitivity to drought indicating their importance in drought tolerance [21]. Overexpression of miR169c in tomato enhanced the drought tolerance by reducing stomatal opening [79]. Therefore, *in silico* screening for miRNAs and their validation for abiotic stress response is highly important especially in the context of non-coding RNAs playing a gamut of regulatory roles. In addition, the PPI analysis revealed that they interact with calcineurins, the calcium sensors that usually confer spatial specificity in Ca^2+^ signalling, and play important roles in abiotic stress tolerance [80]. NF-Ys also participate in circadian clock and flowering time regulation, serine/threonine phosphatases and metal-dependant phosphatases and control the dephosphorylation of phosphoprotein substrates [81].

### Gene expression analysis in different sorghum tissues under abiotic stress conditions

Analysis of *NF-Y* gene expressions by qRT-PCR indicated tissue-specific and stress-inducible expression profile. *NF-YA5*, *A6*, *B7*, *B12*, *B15*, *B16*, *C6*, *C11*, *C12*, *C14* and *C15* revealed significant differential expression patterns in response to the abiotic stresses in *S*. *bicolor*. Such a tissue-specific expression pattern was earlier noticed in several plants [30, 82]. This may indicate a sub-functionalization of different members in specific tissues under different abiotic stress conditions. Pereira et al. [35] pointed out that *CsNF-YA2*, *CsNF-YB5/11* and *CsNF-YC2/3* could form potential complexes in the citrus fruit. Many *NF*-*Y* genes were reported to be associated with both biotic and abiotic stresses. Xu et al. [83] reported high expression of *BnNF-YA10* and *BnNF-YB3*, *BnNF-YB7*, *BnNF-YB10* and *BnNF-YB14* under NaCl stress. Under polyethylene glycol treatment, expression of *BnNF-YA9*, *10*, *11* and *12* genes increased in *B*. *napus*. Malviya et al. [55] performed *in silico* gene expression analysis under abiotic stress conditions using rice transcriptome data. This revealed several of the sorghum *NF-Y* genes are associated with salt, drought, cold and temperature stresses. Since such an analysis is based on rice transcriptome database, this cannot give accurate results. But, in the present study, detailed gene expression studies were carried out and the results indicate that *SbNF-YA1*, *2*, and *6* are upregulated under 200 mM salt and 200 mM mannitol stresses. *NF-YA7* has been found associated with high temperature (40°C) stress, but *NF-YA8* is triggered by both cold (4°C) and high temperature stresses. Among *NF-YB* genes, *7*, *12*, *15*, and *16* are induced under multiple stress conditions such as salt, mannitol, ABA, cold and high temperatures. Likewise, *NF-YC 6*, *11*, *12*, *14*, and *15* have been found enhanced significantly in a tissue specific manner under multiple abiotic stress conditions. Thus, the present analysis revealed that several of the *NF-Ys* are implicated in abiotic stresses and also modulated by ABA. Such a modulation of the *NF-Ys* by ABA was not shown by Malviya et al. [55]. Zhang et al. [84] found that many *Physcomitrella patens NF-Y* genes were responsive to abiotic stresses through ABA-dependent or independent pathways. In the present study, several genes were upregulated when treated with ABA, indicating that they are ABA-dependent. It has been observed from the present study that majority of the mannitol (drought)-inducible genes were also induced by salt, high temperature stresses and ABA. Few of the high temperature stress-induced genes are also induced by cold stress (*NF-YA2*, *4*, *6*, *8*, *NF-YB2*, *7*, *10*, *11*, *12*, *14*, *16*, *17*, *NF-YC4*, *6*, *12*, and *13*). Seki et al. [85] noticed that drought-inducible genes are also inducible by salt stress and ABA treatments in *A*. *thaliana*. Ha et al. [86] observed that diverse transcription factor families modulate plant responses to abiotic stresses independent of ABA or dependent of ABA [87, 88]. Several members of the TFs also function in both ABA-dependent and independent ways [89–91]. Interestingly, such a crosstalk can be achieved *via* indirect interactions between TFs and *cis*-elements present in the same promoter regions of the target genes [92].

Quach et al. [32] reported involvement of soybean *NF-Y* genes in specific developmental stages and also stress responses. In *Prunus mume*, Yang et al. [33] observed high expression of *PmNF-YA1/2/4/5/6*, *PmNF-YB3/4/8/10/11/13*, and *PmNF-YC1/2/4/5/6/8* under osmotic stress and ABA. In citrus, *CsNF-YA5* and *CsNF-YB1/2/4/5/11* were found upregulated by drought stress [35]. Such a finding was proved later by overexpression of *AtNF-YB1* in *Arabidopsis* and its ortholog *ZmNF-YB2* in maize which showed enhanced drought tolerance [93]. Similarly, overexpression of osmotic and ABA-inducible *NF-YB* genes *PwNF-YB3* from *Picea* and *PdNF-YB7* from poplar in *Arabidopsis* exhibited improved drought tolerance activity [94, 95]. Transgenic rice plants harbouring bermudagrass *NF-YC* gene showed tolerance under drought [95]. *NF-Y* genes participate in stress tolerance mechanism by interacting with other stress inducible genes like antioxidants. The connection between *NF-Ys* and antioxidants was observed in previous reports; *CsNF-YA5* [35], *AtNF-YA5* interacts with glutathione S-transferase, peroxidases and an oxidoreductase [94] and S*iNF-YA1* enhance the activity of superoxide dismutase, peroxidase and catalase [94]. Expression profiles exhibited by paralogous *SbNF-Y* genes in different tissues of sorghum under stress treatments suggest a clear functional redundancy among this gene family members. It is interesting to study how these *NF-Ys* regulate the expression of downstream genes that perform a wide spectrum of functions. Siefers et al. [13] pointed out that some transcription factors control gene expression by binding to *cis*-regulatory elements as individual subunits. But, it also appears that others are deployed in a combinatorial fashion both spatially and temporally.

## Conclusions

Genome-wide screening revealed the existence of a total of 42 *NF-Y* genes (8 *SbNF-YA*, 19 *SbNF-YB* and 15 *SbNF-YC* subunit members) in *Sorghum bicolor*. *In silico* analysis of promoters revealed that they comprise many stress-related *cis*-elements such as ABRE and HSE indicating their role in salt, drought and high temperature stress responsiveness. The tissue specific expression of *NF-Y* transcription factors under salt, drought, ABA, cold and high temperature indicated their role in multiple stress tolerance. In view of this, we firmly believe that our studies have allowed identifying the candidate genes for further validation under an array of abiotic stress conditions in a crop species.

## Compliance with ethical requirement

Authors do not have any other interests that influence the results and discussion of this paper. The authors have read the Journal’s policies and the authors of this paper have the following competing interests. RP is the President & CEO of Genomix Molecular Diagnostics Pvt Ltd., Kukatpally, Hyderabad, India. RP is the CEO of Genomix CARL Pvt. Ltd., Andhra Pradesh, India, but does not receive a salary in this capacity. RP is the President and CEO of Genomix Biotech Inc., 2620 Braithwood Road, Atlanta, GA 30345, USA, but does not receive any salary in this capacity. There are no patents, or products in development or marketed products associated with this research to declare. This does not alter our adherence to PLOS ONE policies on sharing data and materials.

## Supporting information

S1 Fig 1–10 MEME identified motif sequences of *SbNF-YA*. (PPT)

S2 Fig 1–10 MEME identified motif sequences of *SbNF-YB*. (PPT)

S3 Fig 1–10 MEME identified motif sequences of *SbNF-YC*. (PPT)

S4 Fig Distribution of 1–10 MEME identified SbNFY-A, B, and C conserved motifs. Gene clusters and p values are shown on the left side and motif sizes at the bottom of the figure.
(PPT)

S5 Fig 1–10 MEME identified motif sequences of *SbNF-Y A*, *B*, and *C*. (PPT)

S6 Fig Gene duplications of *SbNF-Ys*. (PPTX)

S7 Fig Network analysis of SbNF-Ys. (PPT)

S8 Fig Functional partners of SbNFYs and their role in interaction network. (PPT)

S1 Table List of plants searched against *Sorghum bicolor*. (DOC)

S2 Table *SbNFY* gene specific primers used in the gene expression analysis. (DOC)

S3 Table The reliability of identified *SbNF-Ys*. (XLSX)

S4 Table Number of phosphorylation sites in NFYs. (DOC)

S5 Table Non-synonymous to synonymous substitution ratios of *SbNFY-A* orthologs. (DOC)

S6 Table Non-synonymous to synonymous substitution ratios of *SbNFY-B* orthologs. (DOC)

S7 Table Non-synonymous to synonymous substitution ratios of *SbNFY-C* orthologs. (DOC)

S8 Table Conserved *cis*-acting elements in *SbNFY-A* promoters. (DOC)

S9 Table Conserved *cis*-acting elements in *SbNFY-B* promoters. (DOC)

S10 Table Conserved *cis*-acting elements in *SbNFY-C* promoters. (DOC)

S11 Table *In silico* analysis of miRNAs for *SbNFY-A*. (DOC)

S12 Table *In silico* analysis of miRNAs for *SbNFY-B*. (DOC)

S13 Table *In silico* analysis of miRNAs for *SbNFY-C*. (DOC)

S14 Table Native and relative expression values of *SbNFYs*. (DOC)

## Abbreviations

HAP: Heme activator protein
NFY: nuclear factor Y
miRNA: micro RNA
MW: molecular weights
NJ: neighbour joining
pI: iso electric point
PKA: protein kinase A
PKC: protein kinase C
qRT-PCR: quantitative real-time PCR
UTRs: untranslated sequence regions
