# Supplementary figures and images for: Genome-wide identification and expression profile analysis of nuclear factor Y family genes in Sorghum bicolor L. (Moench)
Source: PLoS One. 2019 Sep 19;14(9):e0222203. doi: 10.1371/journal.pone.0222203 (PMC6752760; doi:10.1371/journal.pone.0222203)

## Slide 1
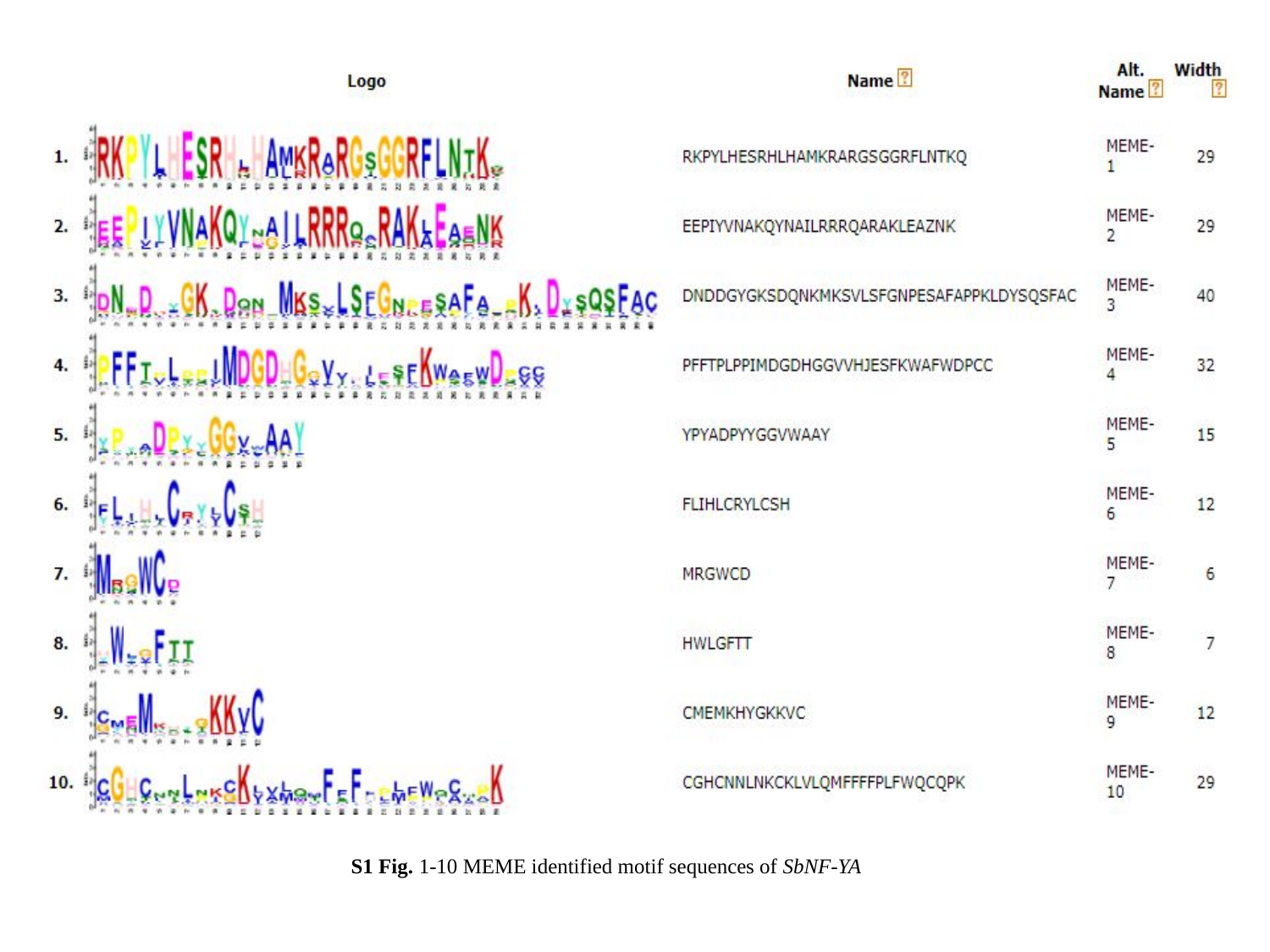

S1 Fig. 1-10 MEME identified motif sequences of SbNF-YA

Supplement: S1 Fig — (PPT) [file pone.0222203.s001.ppt]

## Slide 1
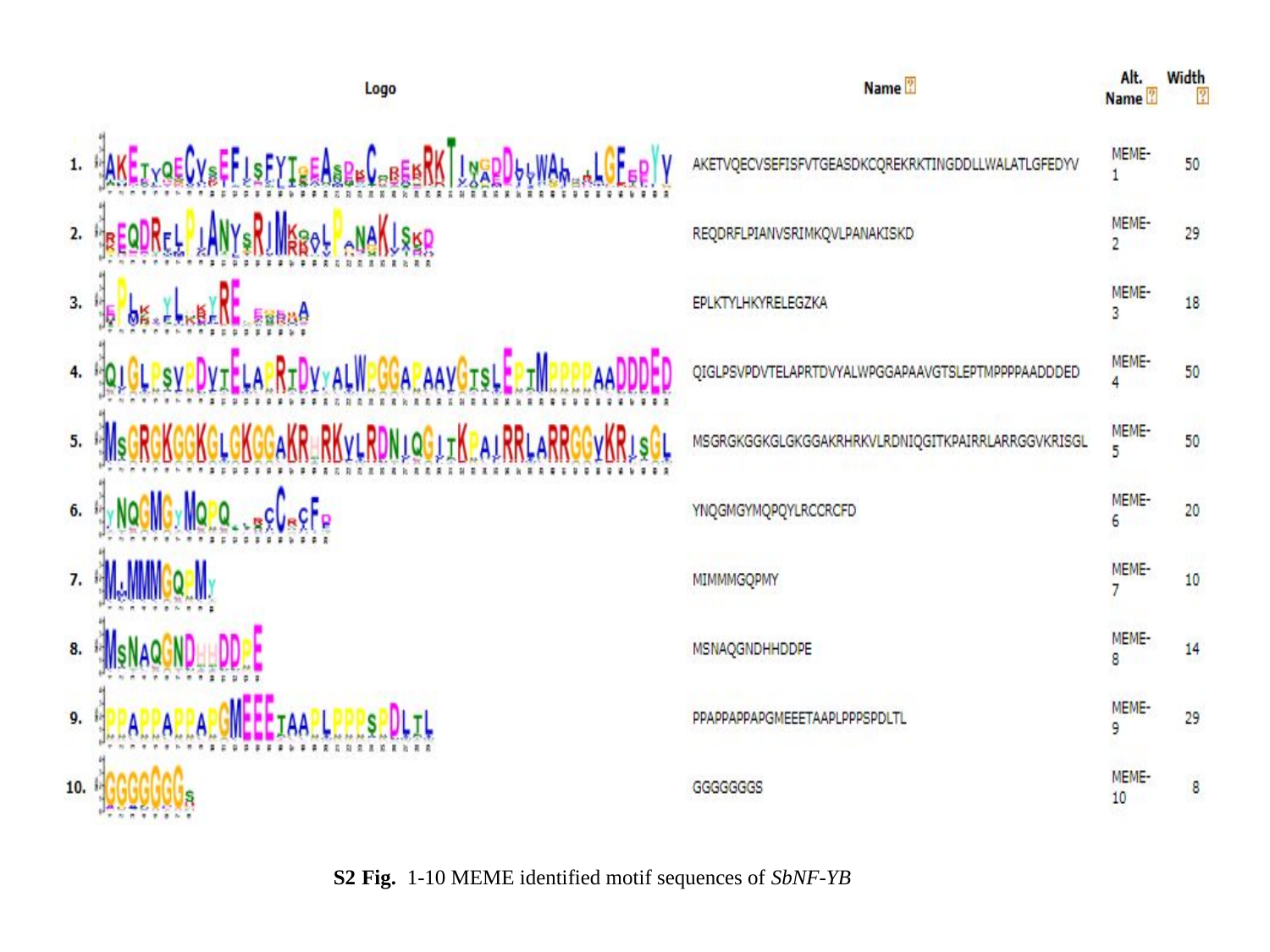

S2 Fig. 1-10 MEME identified motif sequences of SbNF-YB

Supplement: S2 Fig — (PPT) [file pone.0222203.s002.ppt]

## Slide 1
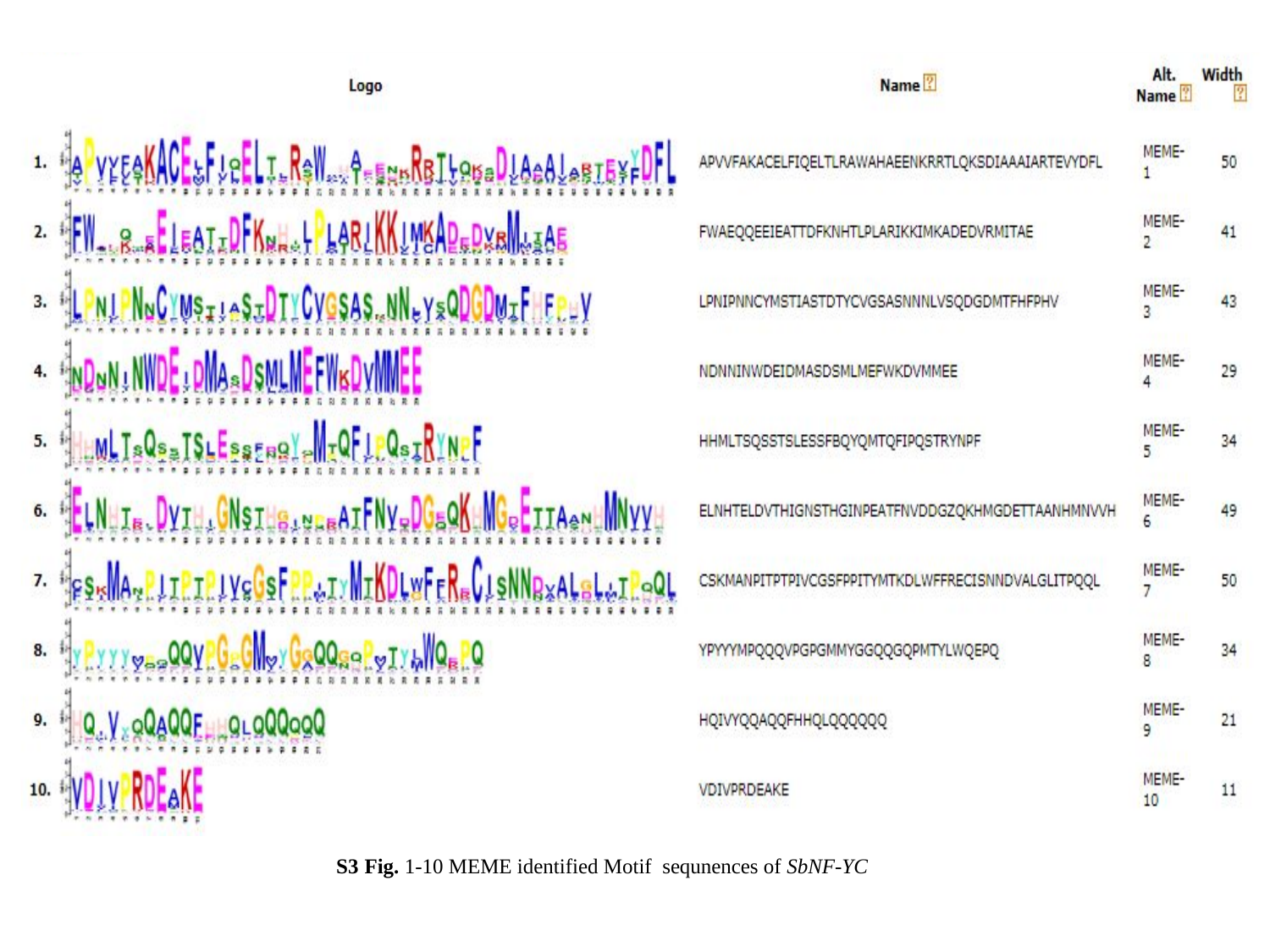

S3 Fig. 1-10 MEME identified Motif sequnences of SbNF-YC

Supplement: S3 Fig — (PPT) [file pone.0222203.s003.ppt]

## Slide 1
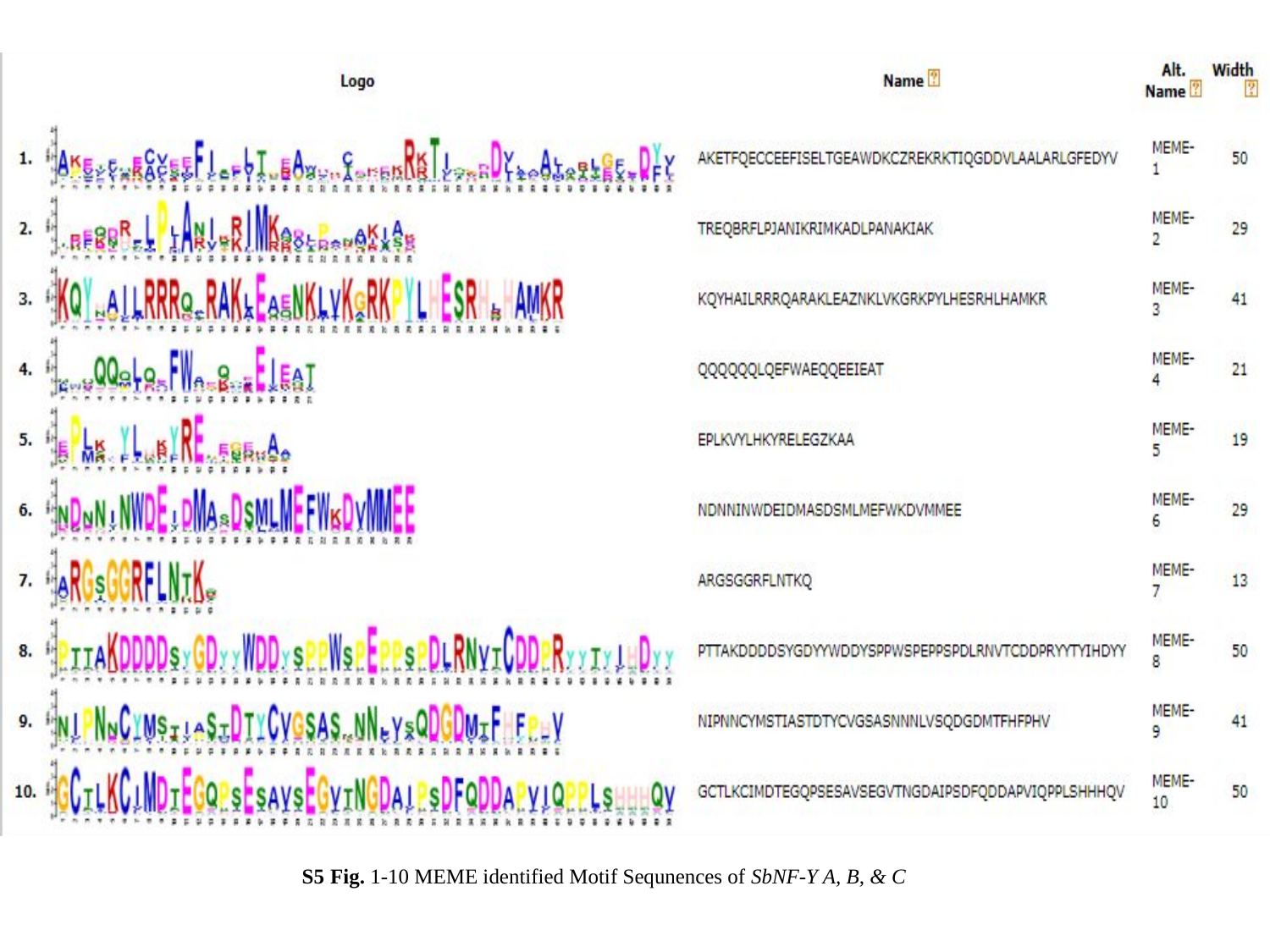

S5 Fig. 1-10 MEME identified Motif Sequnences of SbNF-Y A, B, & C

Supplement: S5 Fig — (PPT) [file pone.0222203.s005.ppt]

## Slide 1
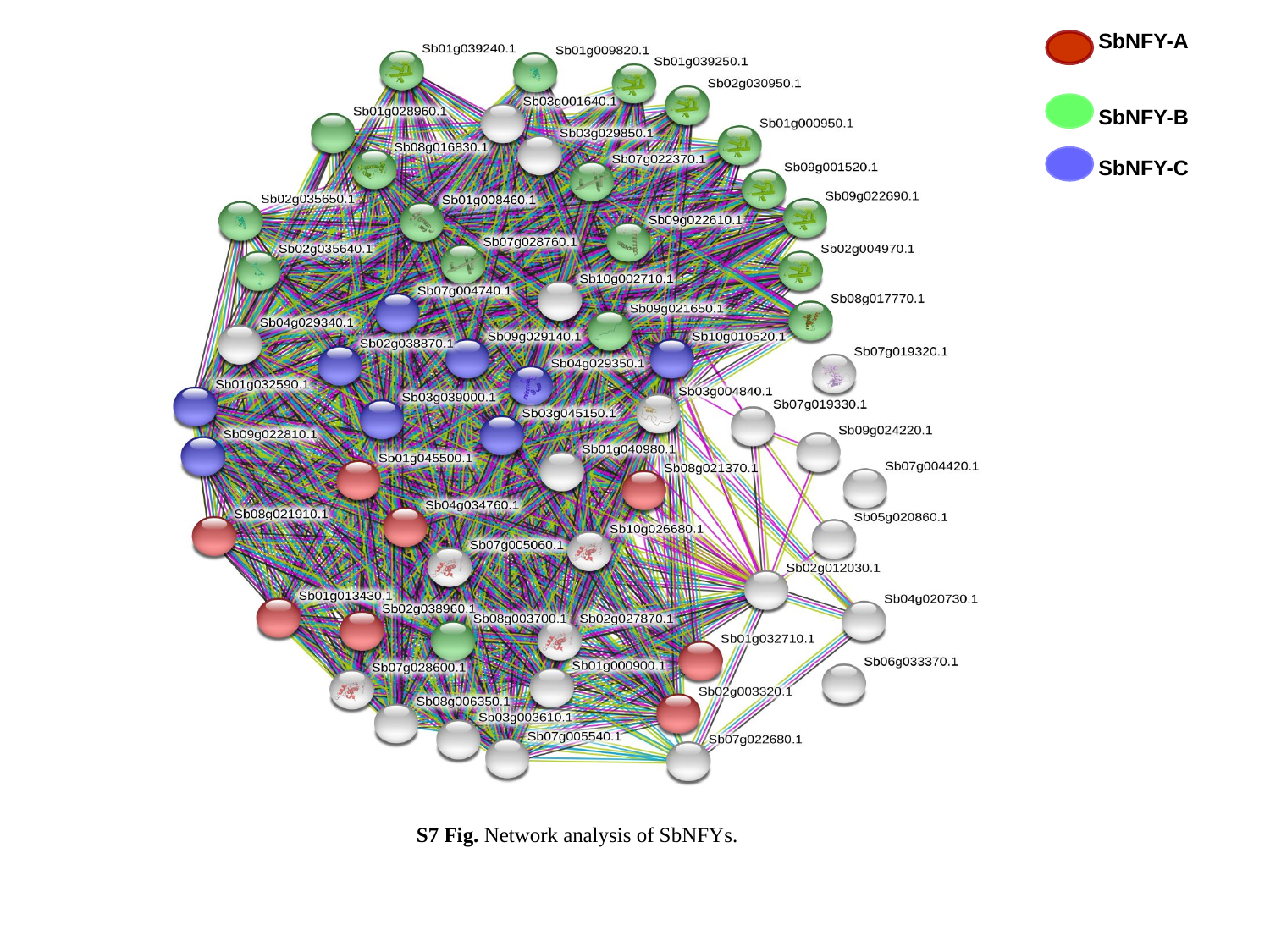

SbNFY-A
SbNFY-B
SbNFY-C
S7 Fig. Network analysis of SbNFYs.

## Slide 2
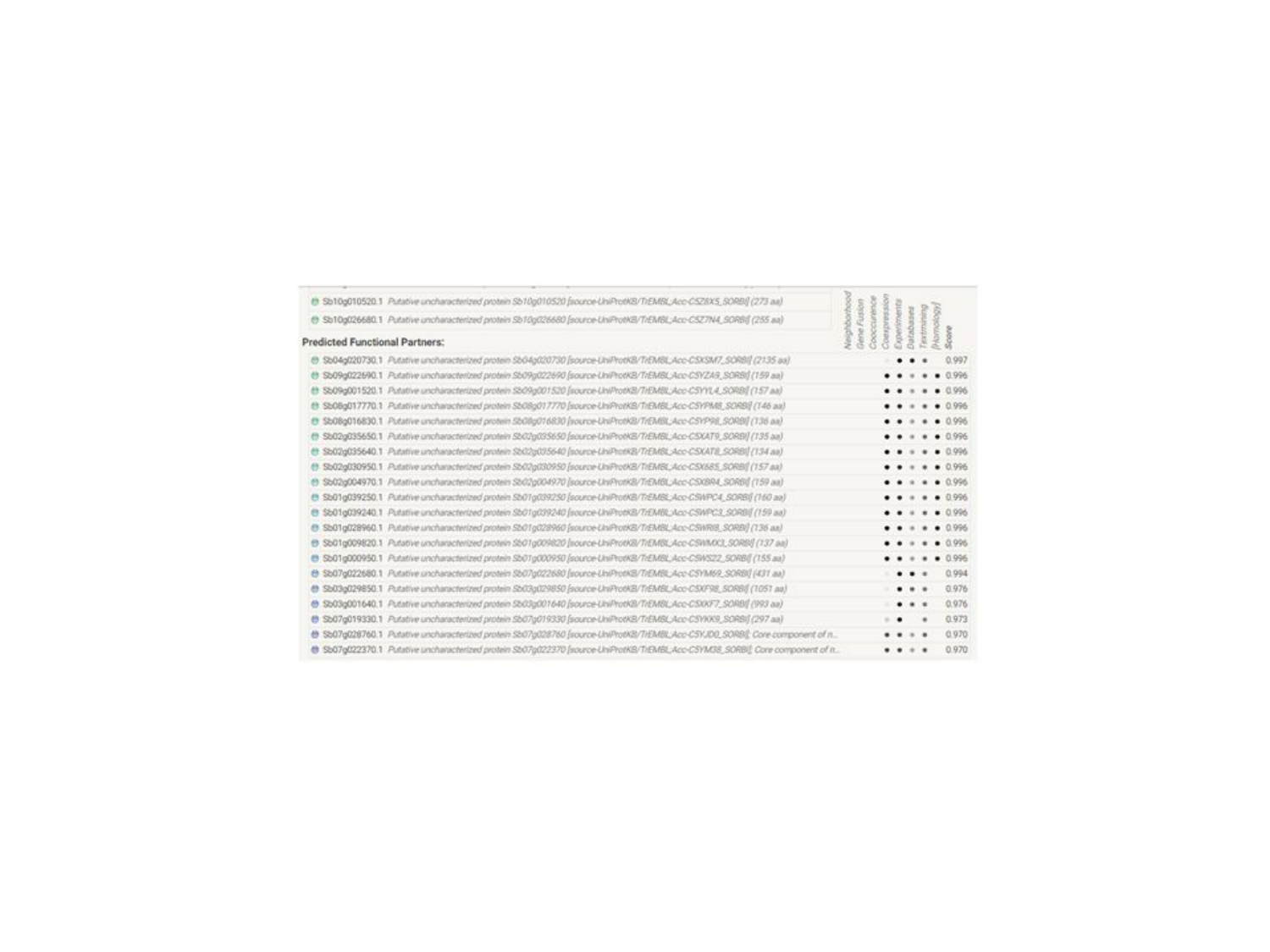

Supplement: S7 Fig — (PPT) [file pone.0222203.s007.ppt]

## Slide 1
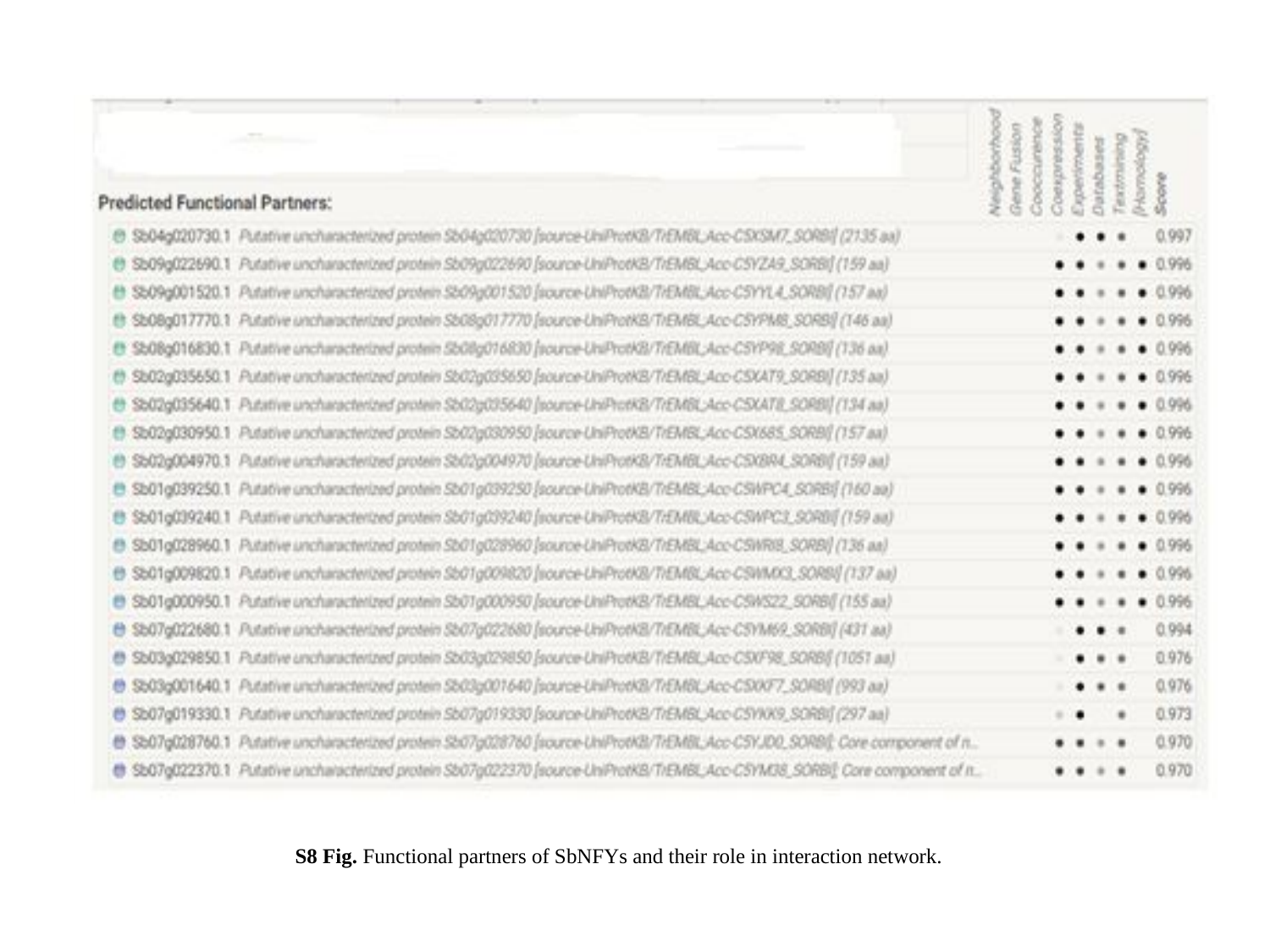

S8 Fig. Functional partners of SbNFYs and their role in interaction network.

Supplement: S8 Fig — (PPT) [file pone.0222203.s008.ppt]
