# Supplementary material for: Genome-wide identification and expression profile analysis of nuclear factor Y family genes in Sorghum bicolor L. (Moench)
Source: PLoS One. 2019 Sep 19;14(9):e0222203. doi: 10.1371/journal.pone.0222203 (PMC6752760; doi:10.1371/journal.pone.0222203)
Supplement: S4 Fig — Gene clusters and p values are shown on the left side and motif sizes at the bottom of the figure. (PPT) [file pone.0222203.s004.ppt]

## Slide 1
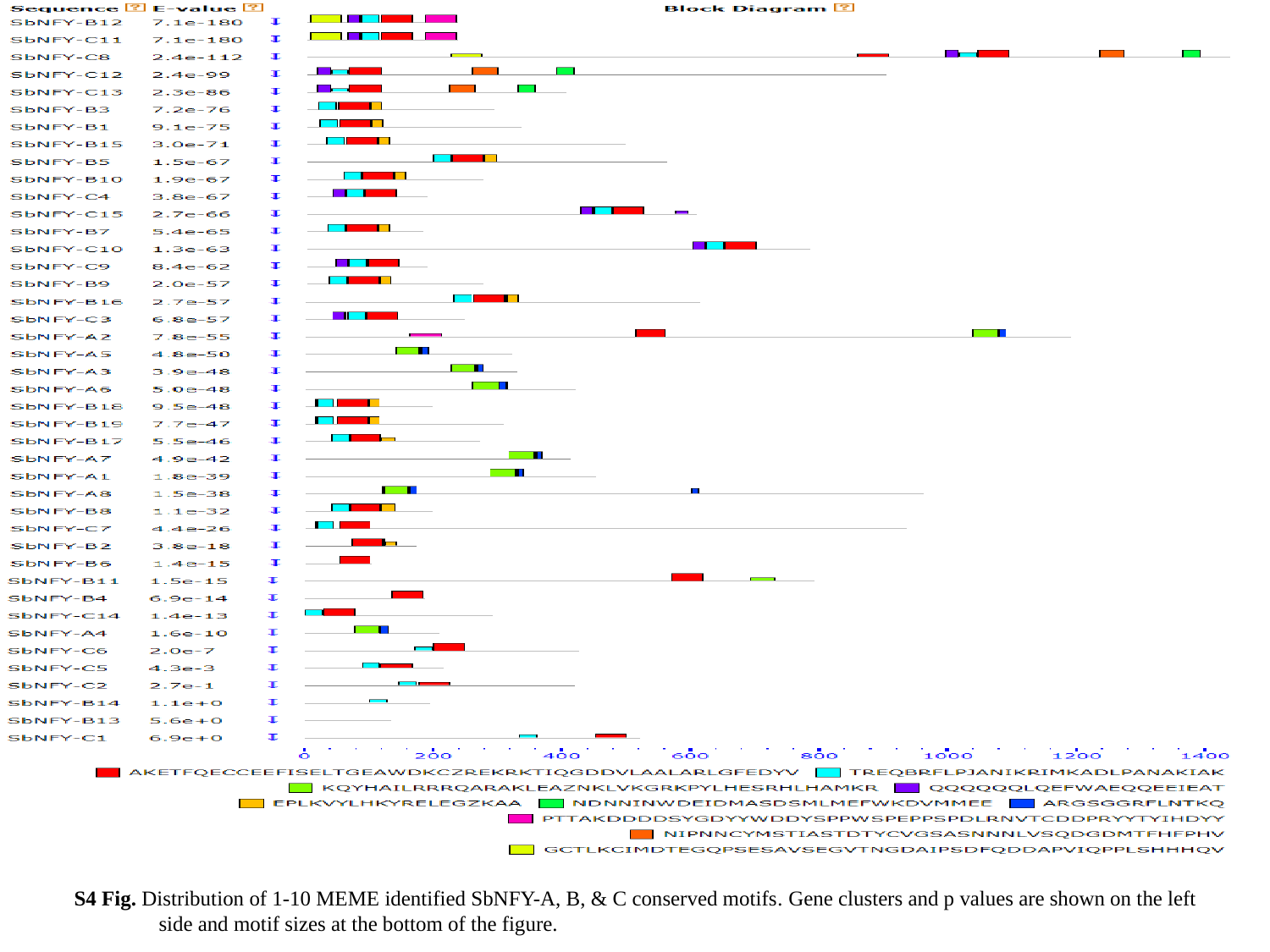

S4 Fig. Distribution of 1-10 MEME identified SbNFY-A, B, & C conserved motifs. Gene clusters and p values are shown on the left
 side and motif sizes at the bottom of the figure.
