# Supplementary material for: Genome-wide identification and expression profile analysis of nuclear factor Y family genes in Sorghum bicolor L. (Moench)
Source: PLoS One. 2019 Sep 19;14(9):e0222203. doi: 10.1371/journal.pone.0222203 (PMC6752760; doi:10.1371/journal.pone.0222203)
Supplement: S6 Fig — (PPTX) [file pone.0222203.s006.pptx]

## Slide 1
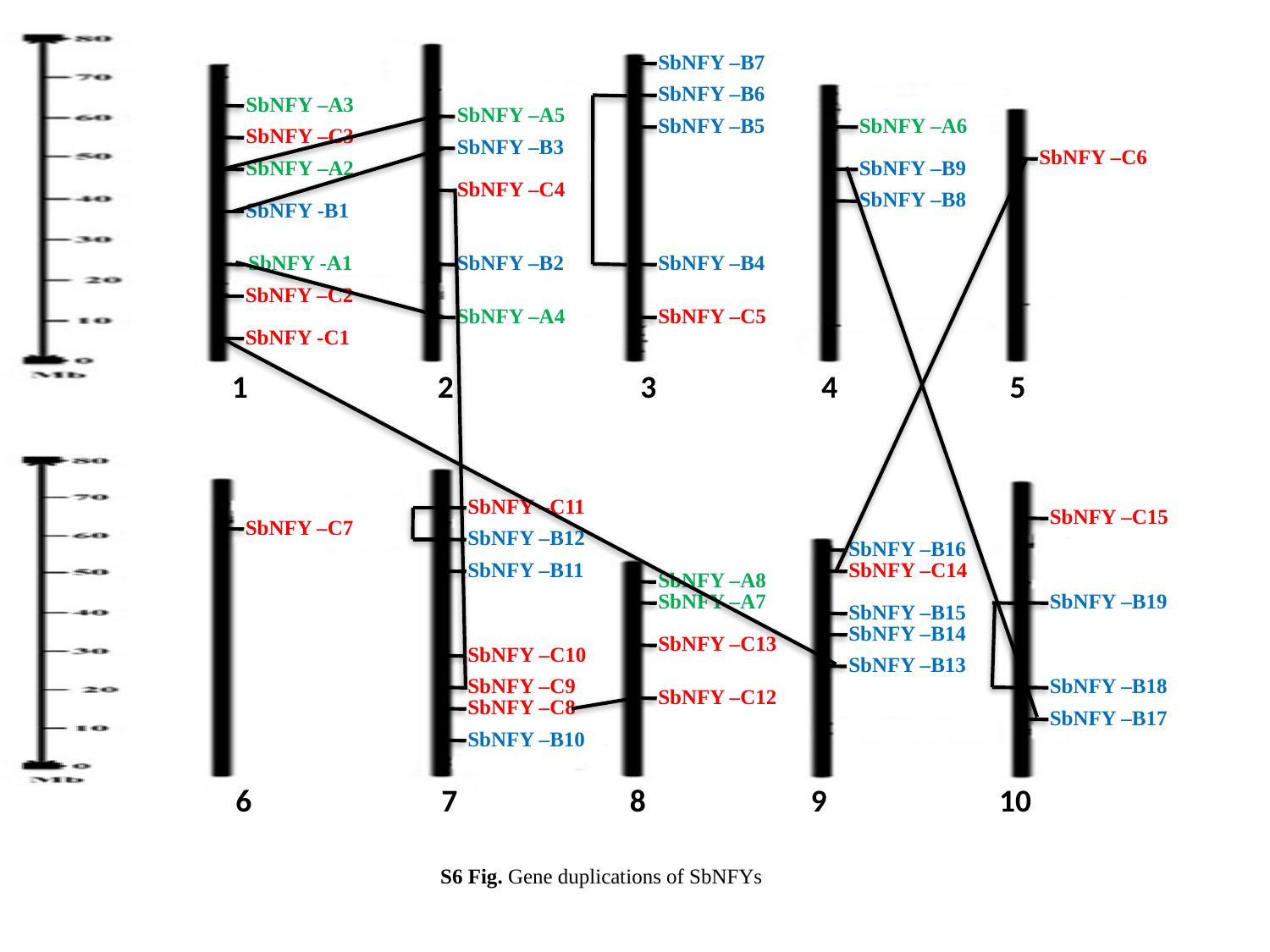

SbNFY –B7
SbNFY –B6
SbNFY –A3
SbNFY –A5
SbNFY –B5
SbNFY –A6
SbNFY –C3
SbNFY –B3
SbNFY –C6
SbNFY –A2
SbNFY –B9
SbNFY –C4
SbNFY –B8
SbNFY -B1
SbNFY -A1
SbNFY –B2
SbNFY –B4
SbNFY –C2
SbNFY –A4
SbNFY –C5
SbNFY -C1
 1	 2 3 4 5
SbNFY –C11
SbNFY –C15
SbNFY –C7
SbNFY –B12
SbNFY –B16
SbNFY –B11
SbNFY –C14
SbNFY –A8
SbNFY –A7
SbNFY –B19
SbNFY –B15
SbNFY –B14
SbNFY –C13
SbNFY –C10
SbNFY –B13
SbNFY –C9
SbNFY –B18
SbNFY –C12
SbNFY –C8
SbNFY –B17
SbNFY –B10
 6	 7 8 9 10
S6 Fig. Gene duplications of SbNFYs
