# Supplementary material for: Genome-wide identification and expression profile analysis of nuclear factor Y family genes in Sorghum bicolor L. (Moench)
Source: PLoS One. 2019 Sep 19;14(9):e0222203. doi: 10.1371/journal.pone.0222203 (PMC6752760; doi:10.1371/journal.pone.0222203)
Supplement: S1 Table — (DOC) [file pone.0222203.s009.doc]

**S1 Table.** List of plants searched against *Sorghum bicolor*

| **S. No.** | **Name of the plant** | **Family** | plantTFDB | | | | **According to literature** | | | | |
| --- | --- | --- | --- | --- | --- | --- | --- | --- | --- | --- | --- |
|  |  |  | **NF-YA** | **NF -YB** | **NF-YC** | **Total** | **NF-**  **YA** | **NF -YB** | **NF-**  **YC** | **Total** | **Ref.** |
|  | *Oryza sativa* | *Poaceae* | 11 | 9 | 15 | 35 | 10 | 11 | 17 | 38 | [27] |
|  | *Zea mays* | *Poaceae* | 16 | 15 | 16 | 47 |  |  |  |  |  |
|  | *Setaria italica* | *Poaceae* | 10 | 15 | 17 | 42 | 10 | 15 | 14 | 39 | [31] |
|  | *Brachypodium distachyon* | *Poaceae* | 7 | 14 | 14 | 35 | 7 | 17 | 12 | 36 | [29] |
|  | *Hordeum vulgare* | *Poaceae* | 7 | 10 | 7 | 24 |  |  |  |  |  |
|  | *Arabidopsis thaliana* | *Brassicaceae* | 11 | 9 | 13 | 33 | 10 | 13 | 13 | 36 | [28] |
|  | *Brassica rapa* | *Brassicaceae* | 38 | 29 | 14 | 81 |  |  |  |  |  |
|  | *Brassica napus* | *Brassicaceae* | 17 | 28 | 27 | 72 | 14 | 14 | 5 | 33 | [30] |
|  | *Gossypium hirsutum* | *Malvaceae* | 29 | 52 | 30 | 111 |  |  |  |  |  |
|  | *Solanum lycopersicum* | *Solanaceae* | 10 | 25 | 18 | 53 | 10 | 29 | 20 | 59 | [61] |
|  | *Capsicum annuum* | *Solanaceae* | 14 | 15 | 13 | 42 |  |  |  |  |  |
|  | *Arachisduranensis* | *Fabaceae* | 8 | 7 | 8 | 23 |  |  |  |  |  |
|  | *Arachis hypogaea* | *Fabaceae* | 7 | 10 | 10 | 27 |  |  |  |  |  |
|  | *Arachis ipaensis* | *Fabaceae* | 9 | 7 | 8 | 24 |  |  |  |  |  |
|  | *Cajanus cajan* | *Fabaceae* | 11 | 21 | 14 | 46 |  |  |  |  |  |
|  | *Cicer arietinum* | *Fabaceae* | 11 | 21 | 10 | 42 | 8 | 21 | 11 | 40 | [60] |
|  | *Phaseolus vulgaris* | *Fabaceae* | 9 | 21 | 17 | 47 |  |  |  |  |  |
|  | *Medicago truncatula* | *Fabaceae* | 8 | 22 | 12 | 42 |  |  |  |  |  |
|  | *Vigna radiate* | *Fabaceae* | 9 | 12 | 13 | 34 |  |  |  |  |  |
|  | *Prunus mume* | *Rosaceae* | 7 | 14 | 11 | 32 | 6 | 13 | 8 | 27 | [33] |
|  | *Dacus carota* | *Apiaceae* | 9 | 27 | 8 | 44 |  |  |  |  |  |
|  | *Beta vulgaris* | *Amaranthaceae* | 7 | 11 | 8 | 26 |  |  |  |  |  |
|  | *Physcomitrella patens* | *Funariaceae* | 2 | 9 | 9 | 20 |  |  |  |  |  |
|  | *Selaginella moellendorffii* | *Selaginellaceae* | 1 | 7 | 5 | 13 |  |  |  |  |  |
|  | *Piceaabies* | *Pinaceae* | 11 | 8 | 10 | 29 |  |  |  |  |  |
|  | *Amborella trichopoda* | *Amborellaceae* | 5 | 9 | 8 | 22 |  |  |  |  |  |
|  | *Solanum tuberosum* | *Solanaceae* | 28 | 31 | 12 | 71 |  |  |  |  |  |
|  | *Fragaria vesca* | *Rosaceae* | 6 | 14 | 9 | 29 |  |  |  |  |  |
|  | *Populus trichocarpa* | *Salicaceae* | 13 | 25 | 17 | 55 |  |  |  |  |  |
|  | *Salix purpurea* | *Salicaceae* | 37 | 33 | 44 | 114 |  |  |  |  |  |
|  | *Vitis vinifera* | *Vitaceae* | 7 | 16 | 7 | 30 |  |  |  |  |  |
