# Supplementary material for: Genome-wide identification and expression profile analysis of nuclear factor Y family genes in Sorghum bicolor L. (Moench)
Source: PLoS One. 2019 Sep 19;14(9):e0222203. doi: 10.1371/journal.pone.0222203 (PMC6752760; doi:10.1371/journal.pone.0222203)
Supplement: S2 Table — (DOC) [file pone.0222203.s010.doc]

**S2 Table. *SbNF-Y* gene specific primers used for gene expression analysis**

| S.  No. | Gene name | Primer | Amplicon length |
| --- | --- | --- | --- |
|  | *NF-YA1* | F: ATTCCATCCGGCCAAGAATC | 107 |
|  |  | R: GAGGAGATTGAAGCTGACGG |  |
|  | *NF-YA2* | F: CGATAATGACCCCCAACCTG | 118 |
|  |  | R: GGTGAGTGGCGGATAAGAAG |  |
|  | *NF-YA3* | F: CCGTGCCTATGTTAAAAGCG | 114 |
|  |  | R: AGCTGTTGGCCCTTATCTCT |  |
|  | *NF-YA4* | F:GCTGCACAAGTACCACCAT | 108 |
|  |  | R:TAGTACGACGCAGTGCCATA |  |
|  | *NF-YA5* | F:AGCAGAACCAGCAGTACCAG | 113 |
|  |  | R:ACCTGCAGTGTCAGAAGAGG |  |
|  | *NF-YA6* | F:CAACTCATCTACGGCTTGGT | 109 |
|  |  | R:GCAAGAGCTGAAGAAACAGC |  |
|  | *NF-YA7* | F: CACCTATAGTGAGCACGTCG | 115 |
|  |  | R: GCCAACTGACCAGTAGTAGC |  |
|  | *NF-YA8* | F: GACTGTGGACTGTGGTGATG | 109 |
|  |  | R: CTGGGACTGTCTGAAACGAG |  |
|  | *NF-YB1* | F: CACGGACATGGTCCTTGAAA | 100 |
|  |  | R: TTGTCGTCGCTCTTTCTTCC |  |
|  | *NF-YB2* | F: GCCACGGCCAATGATATGT | 120 |
|  |  | R: CAGTGAAGTCCTAAGGGGCT |  |
|  | *NF-YB3* | F: GATGGTCATGGGTGGGAAAG | 116 |
|  |  | R: CAAAGTGTTTGCTGCATGGG |  |
|  | *NF-YB4* | F: GACAACATCCAGGGCATCAC | 118 |
|  |  | R: CGAGGAAGATCTTGAGCACG |  |
|  | *NF-YB5* | F: CCATCCTCGCTAAATCGGAC | 99 |
|  |  | R: TCGCTATTTCGATGTGCGTC |  |
|  | *NF-YB6* | F: GACAACATCCAGGGCATCAC | 118 |
|  |  | R: CGAGGAAGATCTTGAGCACG |  |
|  | *NF-YB7* | F: CGCATCATGAAGCAGATCCT | 92 |
|  |  | R: AAGCTGATGAACTCGGAGAC |  |
|  | *NF-YB8* | F: ATGCGTTTCCGATGAGGAG | 115 |
|  |  | R: GTTATCAGGTGAAGTGTGCG |  |
|  | *NF-YB9* | F: CACCACGACATGCAGATG | 137 |
|  |  | R: TAGGGGTAAGGCAGGTAGTG |  |
|  | *NF-YB10* | F:CGAACGCCAAGATCTCCAAG | 122 |
|  |  | R:GTTGATGGTCTTGCGCTTCT |  |
|  | *NF-YB11* | F: TTGGACAACCTTGTCAACCC | 119 |
|  |  | R: GAGTGGAATCGAGGAGGAGT |  |
|  | *NF-YB12* | F: CAATCCCAGCAAGACGATCA | 109 |
|  |  | R: GAAGGCTGTGGGTCTTGAAA |  |
|  | *NF-YB13* | F:CGAGATCCGCAAGTACCAGA | 100 |
|  |  | R:GAGGTCGGTCTTGAAGTCCT |  |
|  | *NFY-B14* | F: TCAAGCACGCTCCTTATTCG | 92 |
|  |  | R: AAGATGGCGGGAAGTTGAAG |  |
|  | *NF-YB15* | F: CCGACTTGCTAAAAGAGGCT | 108 |
|  |  | R: TCCTTTGTGTTCGTCTGGTG |  |
|  | *NF-YB16* | F: TGCTCCAAGGACAAACACG | 119 |
|  |  | R: CGTCTCGATGATCTTCACGG |  |
|  | *NF-YB17* | F: GCGACGATTTTCTCCTGACT | 116 |
|  |  | R: CCGGATTATCACCGTGTTGG |  |
|  | *NF-YB18* | F: GAGTACACGATCCCGAAAGC | 101 |
|  |  | R: CACTGGTCCACAGTCTCTTTG |  |
|  | *NF-YB19* | F: TTACAAGGTATGCAAGCGCC | 104 |
|  |  | R: GCAGTAGACTAAGGGCGTTC |  |
|  | *NF-YC1* | F:CGGTGAAGGATCGTAAGAAG | 106 |
|  |  | R:GCAACTCTCTGCGGTACAAA |  |
|  | *NF-YC2* | F: GGAGCAATTCTCCAGGCTTT | 102 |
|  |  | R: CCTTACCTGAGTTTTCGGCT |  |
|  | *NF-YC3* | F: GAAGATCATGAAGGCCGACG | 99 |
|  |  | R: ATGGTGAGCTCGAGGATGAA |  |
|  | *NF-YC4* | F:CCCGCATCAAGAAGATCA | 106 |
|  |  | R:GTGAGCTCGAGGATGAACA |  |
|  | *NF-YC5* | F: AGGCAATGGAGAAGGATGGA | 116 |
|  |  | R: CTTATTGATGTCCCGGTCCAC |  |
|  | *NF-YC6* | F: CAGCAAAGGACGTCGAGAA | 116 |
|  |  | R: CGTCAACACTCAACAGCACT |  |
|  | *NF-YC7* | F: TCTATAGAGGAAGCCGAGGTG | 118 |
|  |  | R: GTGTCCTTCTCCATCTTGGG |  |
|  | *NF-YC8* | F: GAAAGCTCACCGCTTTGTTG | 119 |
|  |  | R: CCTAGACAATGACAAGGGCG |  |
|  | *NF-YC9* | F:CCACCGACTTCAAGAACCAC | 140 |
|  |  | R:GGTGAGCTCGAGGATGAACA |  |
|  | *NF-YC10* | F: TACATGCGGATTGACGAACC | 114 |
|  |  | R: AACCAGATGGGATGAAAGCG |  |
|  | *NF-YC11* | F: CAATCCCAGCAAGACGATCA | 109 |
|  |  | R: GAAGGCTGTGGGTCTTGAAA |  |
|  | *NF-YC12* | F: GTGACCTTCACTCGACATCC | 110 |
|  |  | R: ATAGAGCCCCAGAGGTGAAC |  |
|  | *NFY-C13* | F: GCAATGGCCCTATTTCCACT | 119 |
|  |  | R: TGTGTCTAACCTTCCATGCG |  |
|  | *NF-YC14* | F: CCATCTGTTCCACGGTCAAA | 107 |
|  |  | R: AAAGCAAACTGGACAGGGTC |  |
|  | *NF-YC15* | F: TGCTTCGGTTCTTACTGTCG | 115 |
|  |  | R:ACTGAGGATCTGTTGGGTCA |  |
| 43. | *PP2A* | F: AACCCGCAAAACCCCAGACTA | 138 |
|  |  | R: TACAGGTCGGGCTCATGGAAC |  |
| 44. | *EIF4α* | F:CAACTTTGTCACCCGCGATGA | 144 |
|  |  | R:TCCAGAAACCTTAGCAGCCCA |  |
