# Supplementary material for: Genome-wide identification and expression profile analysis of nuclear factor Y family genes in Sorghum bicolor L. (Moench)
Source: PLoS One. 2019 Sep 19;14(9):e0222203. doi: 10.1371/journal.pone.0222203 (PMC6752760; doi:10.1371/journal.pone.0222203)
Supplement: S4 Table — (DOC) [file pone.0222203.s012.doc]

| S.No.  **S4 Table.** Number of phosphorylation sites in NFYs | Gene Name | PKA | PKC | CDC-2 | CK-1 | CK-2 | Dnapk | Unsp | cdk5 | p38MAPK | GSK3 | CaM-II | SRC | ATM | PKG |
| --- | --- | --- | --- | --- | --- | --- | --- | --- | --- | --- | --- | --- | --- | --- | --- |
| 1 | SbNF-YA1 | 4 | 15 | 10 | 2 | 3 | 3 | 28 | 5 | 6 | 4 | 0 | 1 | 2 | 1 |
| 2 | SbNF-YA2 | 15 | 35 | 34 | 10 | 11 | 13 | 98 | 14 | 11 | 11 | 0 | 0 | 3 | 2 |
| 3 | SbNF-YA3 | 7 | 7 | 5 | 5 | 6 | 4 | 24 | 1 | 1 | 1 | 0 | 3 | 1 | 3 |
| 4 | SbNF-YA4 | 2 | 6 | 3 | 0 | 0 | 1 | 7 | 0 | 0 | 0 | 0 | 1 | 0 | 0 |
| 5 | SbNF-YA5 | 1 | 10 | 10 | 4 | 8 | 2 | 29 | 2 | 2 | 1 | 0 | 1 | 1 | 1 |
| 6 | SbNF-YA6 | 7 | 19 | 8 | 4 | 0 | 4 | 20 | 1 | 2 | 0 | 0 | 1 | 2 | 1 |
| 7 | SbNF-YA7 | 4 | 13 | 3 | 3 | 5 | 3 | 31 | 3 | 4 | 4 | 0 | 2 | 1 | 2 |
| 8 | SbNF-YA8 | 9 | 19 | 15 | 4 | 14 | 5 | 66 | 9 | 7 | 5 | 0 | 2 | 1 | 3 |
| 9 | SbNF-YB1 | 4 | 10 | 5 | 0 | 5 | 2 | 26 | 2 | 2 | 1 | 0 | 1 | 2 | 1 |
| 10 | SbNF-YB2 | 3 | 2 | 0 | 1 | 3 | 0 | 9 | 0 | 0 | 0 | 0 | 0 | 0 | 3 |
| 11 | SbNF-YB3 | 3 | 6 | 5 | 1 | 5 | 1 | 20 | 4 | 0 | 3 | 0 | 0 | 0 | 0 |
| 12 | SbNF-YB4 | 4 | 6 | 2 | 0 | 0 | 0 | 6 | 0 | 0 | 0 | 0 | 0 | 0 | 0 |
| 13 | SbNF-YB5 | 7 | 15 | 11 | 2 | 3 | 2 | 38 | 2 | 1 | 3 | 0 | 1 | 1 | 3 |
| 14 | SbNF-YB6 | 3 | 3 | 2 | 0 | 1 | 0 | 4 | 0 | 0 | 0 | 0 | 0 | 0 | 0 |
| 15 | SbNF-YB7 | 1 | 4 | 2 | 1 | 3 | 0 | 10 | 0 | 0 | 0 | 0 | 0 | 0 | 0 |
| 16 | SbNF-YB8 | 0 | 5 | 3 | 1 | 3 | 1 | 16 | 2 | 1 | 0 | 0 | 0 | 0 | 0 |
| 17 | SbNF-YB9 | 1 | 3 | 6 | 0 | 2 | 0 | 12 | 1 | 1 | 1 | 0 | 1 | 0 | 0 |
| 18 | SbNF-YB10 | 1 | 6 | 3 | 0 | 4 | 0 | 9 | 3 | 2 | 2 | 0 | 0 | 0 | 1 |
| 19 | SbNF-YB11 | 17 | 32 | 11 | 2 | 9 | 2 | 44 | 6 | 3 | 2 | 0 | 1 | 1 | 7 |
| 20 | SbNF-YB12 | 1 | 7 | 3 | 0 | 3 | 3 | 18 | 3 | 2 | 3 | 0 | 1 | 0 | 0 |
| 21 | SbNF-YB13 | 3 | 6 | 1 | 0 | 0 | 0 | 6 | 0 | 0 | 0 | 0 | 0 | 0 | 2 |
| 22 | SbNF-YB14 | 1 | 9 | 2 | 0 | 0 | 0 | 17 | 2 | 2 | 0 | 0 | 0 | 0 | 1 |
| 23 | SbNF-YB15 | 13 | 18 | 6 | 1 | 2 | 2 | 31 | 2 | 3 | 0 | 0 | 0 | 1 | 3 |
| 24 | SbNF-YB16 | 10 | 28 | 15 | 4 | 7 | 2 | 46 | 2 | 1 | 3 | 0 | 2 | 1 | 2 |
| 25 | SbNF-YB17 | 6 | 5 | 6 | 0 | 4 | 1 | 13 | 1 | 1 | 0 | 0 | 0 | 1 | 1 |
| 26 | SbNF-YB18 | 2 | 5 | 0 | 0 | 2 | 1 | 10 | 0 | 1 | 1 | 0 | 0 | 0 | 1 |
| 27 | SbNF-YB19 | 4 | 8 | 3 | 0 | 2 | 0 | 14 | 1 | 1 | 1 | 0 | 0 | 0 | 1 |
| 28 | SbNF-YC1 | 4 | 19 | 8 | 0 | 2 | 2 | 52 | 16 | 18 | 7 | 0 | 0 | 0 | 6 |
| 29 | SbNF-YC2 | 17 | 9 | 4 | 3 | 9 | 1 | 27 | 2 | 1 | 2 | 0 | 0 | 0 | 6 |
| 30 | SbNF-YC3 | 2 | 1 | 3 | 2 | 3 | 0 | 8 | 1 | 1 | 2 | 0 | 0 | 0 | 0 |
| 31 | SbNF-YC4 | 1 | 2 | 1 | 0 | 1 | 0 | 3 | 0 | 0 | 0 | 0 | 0 | 0 | 0 |
| 32 | SbNF-YC5 | 2 | 1 | 1 | 1 | 7 | 0 | 10 | 0 | 0 | 0 | 0 | 0 | 0 | 0 |
| 33 | SbNF-YC6 | 8 | 11 | 5 | 0 | 8 | 1 | 29 | 4 | 4 | 1 | 0 | 0 | 0 | 2 |
| 34 | SbNF-YC7 | 16 | 16 | 7 | 1 | 5 | 1 | 43 | 1 | 2 | 0 | 0 | 2 | 0 | 7 |
| 35 | SbNF-YC8 | 15 | 33 | 13 | 7 | 17 | 9 | 64 | 7 | 13 | 8 | 0 | 1 | 4 | 7 |
| 36 | SbNF-YC9 | 1 | 2 | 1 | 0 | 1 | 1 | 4 | 0 | 0 | 0 | 0 | 0 | 1 | 0 |
| 37 | SbNF-YC10 | 11 | 15 | 14 | 3 | 10 | 7 | 34 | 2 | 4 | 1 | 0 | 0 | 3 | 4 |
| 38 | SbNF-YC11 | 1 | 7 | 3 | 0 | 3 | 3 | 18 | 3 | 2 | 3 | 0 | 1 | 0 | 1 |
| 39 | SbNF-YC12 | 13 | 25 | 17 | 5 | 14 | 2 | 47 | 5 | 5 | 2 | 0 | 0 | 1 | 5 |
| 40 | SbNF-YC13 | 4 | 10 | 5 | 1 | 6 | 6 | 26 | 3 | 1 | 0 | 0 | 2 | 1 | 2 |
| 41 | SbNF-YC14 | 1 | 12 | 1 | 0 | 7 | 0 | 14 | 0 | 1 | 0 | 0 | 0 | 0 | 0 |
| 42 | SbNF-YC15 | 6 | 21 | 7 | 1 | 4 | 1 | 27 | 4 | 4 | 3 | 0 | 0 | 1 | 2 |

(PKC: Protein Kinase C, CK2: Casein Kinase 2, RSK1: Ribosomal S6 Kinase 1, PKA: Protein Kinase A, CK1: Casein Kinase, DNAPK: DNA dependant Protein Kinase, ATM: Ataxia-telangiectasia mutated **kinase**, EGFR: epidermal growth factor receptor, INSR: insulin receptor tyrosine kinase, PKG: Protein Kinase G, CDK: Cyclin dependent kinases)
