# Supplementary material for: Genome-wide identification and expression profile analysis of nuclear factor Y family genes in Sorghum bicolor L. (Moench)
Source: PLoS One. 2019 Sep 19;14(9):e0222203. doi: 10.1371/journal.pone.0222203 (PMC6752760; doi:10.1371/journal.pone.0222203)
Supplement: S5 Table — (DOC) [file pone.0222203.s013.doc]

| Sorghum | Paralog | No. of Non -synonymous sites  (N) | No. of Synonymous sites (S) | Non -synonymous substitution rate (d_N_) | Synonymous substitution rate (d_S_) | d_N_ / d_S_ |
| --- | --- | --- | --- | --- | --- | --- |
| Sorbi002g038500 | Zm2G096016 | 534.2 | 98.8 | 14.6703 | 3.9669 | 3.6982 |
| Sorbi008G168300 | Zm2G104396 | 498.1 | 146.9 | 6.0025 | 12.4142 | 0.4835 |
| Sorbi001G154500 | Zm2G165488 | 760.1 | 262.9 | 14.8777 | 7.5713 | 1.9650 |
| Sorbi001G340200 | Seita.9G367200 | 660.7 | 278.3 | 6.6242 | 16.5605 | 0.4000 |
| Sorbi001G486000 | Zm2G000686 | 493.5 | 217.5 | 14.6528 | 0.5109 | 28.6781 |
| Sorbi004G316500 | Zm5G857944 | 589.7 | 205.3 | 3.6683 | 1.6678 | 2.1995 |

**S5 Table.** Non Synonymous to synonymous substitution ratios of SbNFY-A orthologs

(**d_N_ / d_S_ >1 = Positive or Darwinian Selection (Driving Change); d_N_ / d_S_ <1 = Purifying or Stabilizing Selection**

**(Acting against change); d_N_ / d_S_ =1 Neutral Selection** )
