# Supplementary material for: Genome-wide identification and expression profile analysis of nuclear factor Y family genes in Sorghum bicolor L. (Moench)
Source: PLoS One. 2019 Sep 19;14(9):e0222203. doi: 10.1371/journal.pone.0222203 (PMC6752760; doi:10.1371/journal.pone.0222203)
Supplement: S6 Table — (DOC) [file pone.0222203.s014.doc]

| Sorghum | Paralog | No. of Non -synonymous sites  (N) | No. of Synonymous sites (S) | Non -synonymous substitution rate (d_N_) | Synonymous substitution rate (d_S_) | d_N_ / d_S_ |
| --- | --- | --- | --- | --- | --- | --- |
| Sorbi009G166200 | MLOC_ 36879.2 | 208.8 | 31.2 | 8.0246 | 9.7418 | 0.8237 |
| Sorbi003G346500 | ZM2G30346500 | 290.0 | 46.0 | 4.0547 | 69.3388 | 0.0585 |
| Sorbi009G239600 | Seita. 3G138600 | 385.8 | 142.2 | 16.8301 | 2.6119 | 6.4436 |
| Sorbi002G135100 | ZM2G012654 | 387.1 | 134.9 | 0.0525 | 0.1623 | 0.3235 |
| Sorbi004G254400 | Seita. 1G302900 | 485.1 | 105.9 | 7.7413 | 2.6865 | 2.8816 |
| Sorbi010G119200 | ZM2G167576 | 631.9 | 154.1 | 15.7927 | 1.5449 | 10.2227 |
| Sorbi004G254500 | ZM2G011789 | 710.3 | 117.7 | 1.7413 | 2.9430 | 0.5917 |
| Sorbi007G059500 | ZM2G444073 | 670.1 | 106.9 | 1.3431 | 2.1848 | 0.6147 |
| Sorbi003G417700 | Seita. 5G443800 | 451.4 | 61.6 | 3.8490 | 2.0487 | 1.8787 |
| Sorbi001G338700 | Seita. 9G365700 | 585.4 | 92.6 | 0.2906 | 93.3503 | 0.0031 |
| Sorbi002G369800 | ZM2G180947 | 554.8 | 60.2 | 3.7410 | 98.2680 | 0.0381 |

**S6 Table**. Non Synonymous to synonymous substitution ratios of SbNFY-B orthologs

(**d_N_ / d_S_ >1 = Positive or Darwinian Selection (Driving Change); d_N_ / d_S_ <1 = Purifying or Stabilizing Selection**

**(Acting against change); d_N_ / d_S_ =1 Neutral Selection** )
