# Supplementary material for: Genome-wide identification and expression profile analysis of nuclear factor Y family genes in Sorghum bicolor L. (Moench)
Source: PLoS One. 2019 Sep 19;14(9):e0222203. doi: 10.1371/journal.pone.0222203 (PMC6752760; doi:10.1371/journal.pone.0222203)
Supplement: S7 Table — (DOC) [file pone.0222203.s015.doc]

| Sorghum | Paralog | No. of Non -synonymous sites  (N) | No. of Synonymous sites (S) | Non -synonymous substitution rate (d_N_) | Synonymous substitution rate (d_S_) | d_N_ / d_S_ |
| --- | --- | --- | --- | --- | --- | --- |
| Sorbi007G063200 | ZM2G311316 | 549.8 | 203.2 | 14.9650 | 7.5730 | 1.9761 |
| Sorbi001G435500 | ZM2G089812 | 602.2 | 132.8 | 1.3575 | 1.9277 | 0.7042 |
| Sorbi002G241500 | Seita. 2G247700 | 447.8 | 116.2 | 3.8465 | 48.2802 | 0.0797 |
| Sorbi007G219500 | ZM2G113127 | 468.7 | 95.3 | 3.2050 | 2.1904 | 1.4623 |
| Sorbi006G272400 | ZM2G074773 | 336.1 | 44.9 | 0.5849 | 1.7184 | 0.3403 |
| Sorbi007G070100 | Seita. 6G034500 | 283.4 | 55.6 | 3.4055 | 2.1686 | 1.5704 |
| Sorbi001G009200 | ZM2G083964 | 877.6 | 223.4 | 0.9952 | 1.437 | 0.5398 |
| Sorbi003G040500 | ZM2G099461 | 479.6 | 129.4 | 1.4503 | 2.3217 | 0.6247 |
| Sorbi005G143400 | ZM2G105317 | 551.4 | 150.6 | 3.1701 | 10.8871 | 0.2912 |
| Sorbi009G181900 | ZM2G440949 | 662.0 | 205.0 | 0.4847 | 0.8830 | 0.5489 |

**S7 Table**. Non Synonymous to synonymous substitution ratios of SbNFY-C orthologs

(**d_N_ / d_S_ >1 = Positive or Darwinian Selection (Driving Change); d_N_ / d_S_ <1 = Purifying or Stabilizing Selection**

**(Acting against change); d_N_ / d_S_ =1 Neutral Selection** )

(**d_N_ / d_S_ >1 = Positive or Darwinian Selection (Driving Change); d_N_ / d_S_ <1 = Purifying or Stabilizing Selection**

**(Acting against change); d_N_ / d_S_ =1 Neutral Selection** )
