# Supplementary material for: Genome-wide identification and expression profile analysis of nuclear factor Y family genes in Sorghum bicolor L. (Moench)
Source: PLoS One. 2019 Sep 19;14(9):e0222203. doi: 10.1371/journal.pone.0222203 (PMC6752760; doi:10.1371/journal.pone.0222203)
Supplement: S10 Table — (DOC) [file pone.0222203.s018.doc]

| **S10 Table.** Conserved *cis*-acting elements in *SbNFY-C* promoters | NF-YC1 | NF-YC2 | NF-YC3 | NF-YC4 | NF-YC5 | NF-YC6 | NF-YC7 | NF-YC8 | NF-YC9 | NF-YC10 | NF-YC11 | NF-YC12 | NF-YC13 | NF-YC14 | NF-YC15 |
| --- | --- | --- | --- | --- | --- | --- | --- | --- | --- | --- | --- | --- | --- | --- | --- |
| **STRESS** |  | | | | | | | | | | | | | | |
| DRE | 0 | 0 | 0 | 0 | 0 | 0 | 0 | 0 | 0 | 0 | 0 | 0 | 0 | 0 | 0 |
| DPBF | 0 | 0 | 0 | 0 | 0 | 0 | 0 | 0 | 0 | 0 | 0 | 0 | 0 | 0 | 0 |
| MYB | 0 | 0 | 0 | 0 | 0 | 0 | 0 | 0 | 0 | 0 | 0 | 0 | 0 | 0 | 0 |
| HSE | 1 | 0 | 0 | 0 | 4 | 3 | 0 | 2 | 3 | 1 | 1 | 0 | 1 | 0 | 1 |
| LTRE | 0 | 0 | 1 | 1 | 0 | 0 | 0 | 0 | 0 | 0 | 1 | 0 | 0 | 0 | 0 |
| GT1GMSCAM4 | 0 | 0 | 0 | 0 | 0 | 0 | 0 | 0 | 0 | 0 | 0 | 0 | 0 | 0 | 0 |
| SP1 | 3 | 1 | 3 | 2 | 2 | 2 | 7 | 3 | 3 | 5 | 2 | 0 | 3 | 9 | 1 |
| CATT-motif | 0 | 0 | 0 | 0 | 0 | 1 | 0 | 0 | 1 | 1 | 0 | 2 | 0 | 0 | 0 |
| G-Box | 5 | 2 | 5 | 6 | 4 | 4 | 0 | 4 | 0 | 3 | 6 | 0 | 5 | 3 | 1 |
| TCT-motif | 1 | 1 | 0 | 1 | 0 | 0 | 1 | 1 | 1 | 0 | 1 | 1 | 2 | 0 | 3 |
| TGG | 0 | 0 | 0 | 0 | 0 | 0 | 0 | 0 | 0 | 0 | 0 | 0 | 0 | 0 | 0 |
| I-BOX | 1 | 5 | 0 | 0 | 1 | 0 | 2 | 0 | 0 | 0 | 0 | 0 | 3 | 0 | 0 |
| **HORMONE** |  | | | | | | | | | | | | | | |
| ABRE | 2 | 0 | 4 | 4 | 1 | 1 | 0 | 0 | 1 | 2 | 1 | 0 | 1 | 0 | 0 |
| TCA-element | 1 | 1 | 1 | 1 | 1 | 2 | 3 | 1 | 1 | 2 | 1 | 0 | 0 | 2 | 2 |
| TGACG-motif | 0 | 1 | 1 | 2 | 5 | 4 | 2 | 2 | 2 | 2 | 3 | 1 | 2 | 2 | 1 |
| CGTCA-motif | 0 | 1 | 1 | 2 | 5 | 4 | 1 | 2 | 2 | 2 | 3 | 2 | 2 | 0 | 1 |
| GARE-motif | 0 | 2 | 1 | 0 | 0 | 0 | 0 | 0 | 2 | 2 | 0 | 0 | 0 | 0 | 0 |
| TGA-box | 0 | 0 | 0 | 0 | 0 | 2 | 2 | 1 | 0 | 0 | 0 | 1 | 0 | 1 | 1 |
| TATC-box | 0 | 0 | 1 | 0 | 1 | 0 | 0 | 1 | 0 | 0 | 0 | 0 | 0 | 0 | 0 |
| AuxRR-core | 0 | 0 | 0 | 0 | 0 | 0 | 0 | 0 | 0 | 0 | 0 | 0 | 0 | 0 | 1 |
| **DEVELOPMENT** |  | | | | | | | | | | | | | | |
| GCN4_motif | 0 | 1 | 0 | 0 | 0 | 3 | 0 | 0 | 0 | 0 | 0 | 0 | 0 | 0 | 0 |
| Skn-1_motif | 2 | 6 | 5 | 1 | 2 | 11 | 4 | 1 | 4 | 3 | 7 | 10 | 3 | 3 | 2 |
| POLLEN | 0 | 0 | 0 | 0 | 0 | 0 | 0 | 0 | 0 | 0 | 0 | 0 | 0 | 0 | 0 |
| CCGTCC-box | 0 | 0 | 0 | 0 | 0 | 0 | 1 | 0 | 0 | 0 | 0 | 0 | 0 | 3 | 0 |
| Circadian | 0 | 2 | 3 | 0 | 1 | 4 | 1 | 2 | 3 | 5 | 1 | 1 | 2 | 0 | 0 |
| **BIOTIC** |  | | | | | | | | | | | | | | |
| TC-rich repeats | 1 | 0 | 1 | 2 | 0 | 2 | 0 | 0 | 3 | 1 | 1 | 0 | 1 | 1 | 2 |
| WBOXATNPR1 | 0 | 0 | 0 | 0 | 0 | 0 | 0 | 0 | 0 | 0 | 0 | 0 | 0 | 0 | 0 |
| WBOXNTERF3 | 0 | 0 | 0 | 0 | 0 | 0 | 0 | 0 | 0 | 0 | 0 | 0 | 0 | 0 | 0 |
| **OTHERS** |  | | | | | | | | | | | | | | |
| TKST1 | 0 | 0 | 0 | 0 | 0 | 0 | 0 | 0 | 0 | 0 | 0 | 0 | 0 | 0 | 0 |
| CGCGBOX | 0 | 0 | 0 | 0 | 0 | 0 | 0 | 0 | 0 | 0 | 0 | 0 | 0 | 0 | 0 |
| O2-site | 1 | 0 | 0 | 0 | 0 | 3 | 1 | 0 | 0 | 1 | 0 | 1 | 0 | 1 | 0 |
| AT-rich element | 0 | 1 | 0 | 1 | 0 | 0 | 0 | 0 | 0 | 1 | 0 | 0 | 1 | 0 | 0 |

(ABRECTAL: Response to ABA, ANAERO: Anaerobic conditions, ARF: ABA and auxin responsive, CGCGBOX: Multiple signal transduction, CURE: Cu and oxygen responsive, DPBF: ABA, DRE: Dehydration responsive elements, GT1GMSAM4: Salt and pathogenesis related, LTRE: Low temperature and cold responsive, MYB: responsive to drought and ABA, MYC: Response to drought, cold and ABA, POLLEN: pollen and anther development, TKST1: Guard cell-specific gene expression, WBOXNTERF3: Wound signal and WBOXATNPR1: Salicylic acid responsive, ERE: Ethylene responsive elements, GARE: Gibberellic acid responsive elements, TCA: Salicylic acid-responsive, TGACG: Me-Jasmonic acid responsive)
