# Supplementary material for: Genome-wide identification and expression profile analysis of nuclear factor Y family genes in Sorghum bicolor L. (Moench)
Source: PLoS One. 2019 Sep 19;14(9):e0222203. doi: 10.1371/journal.pone.0222203 (PMC6752760; doi:10.1371/journal.pone.0222203)
Supplement: S11 Table — (DOC) [file pone.0222203.s019.doc]

| **S11 Table.** In silico analysis of miRNAs for SbNFY-A | | | | | | | |  |  |  |  |  |
| --- | --- | --- | --- | --- | --- | --- | --- | --- | --- | --- | --- | --- |
| miRNA_Acc. | Target_Acc. | Expectation | UPE$ | miRNA_start | miRNA_end | Target_start | Target_end | miRNA_aligned_fragment | Target_aligned_fragment | Inhibition | Target_Desc. | Multiplicity |
| sbi-miR6220-5p | SbNFY-A6 | 0.5 | -1 | 1 | 24 | 12206 | 12229 | CUCCAUCCUAAAUUAUAAGACAUU | AACUUCUUAUAAUUUGGGAUGGAG | Cleavage |  | 1 |
| sbi-miR169o | SbNFY-A1 | 1 | -1 | 1 | 21 | 12857 | 12877 | UAGCCAAGGAUGAUUUGCCUG | UAGGCAAAUCAUUCUUGGCUG | Cleavage |  | 1 |
| sbi-miR169o | SbNFY-A8 | 1 | -1 | 1 | 21 | 7556 | 7576 | UAGCCAAGGAUGAUUUGCCUG | UAGGCAAAUCAUUCUUGGCUG | Cleavage |  | 1 |
| sbi-miR5568f-5p | SbNFY-A6 | 1 | -1 | 1 | 21 | 12208 | 12228 | UCCAUUCCAAAUUGUAAGAUG | CUUCUUAUAAUUUGGGAUGGA | Cleavage |  | 1 |
| sbi-miR6225-5p | SbNFY-A6 | 1 | -1 | 1 | 24 | 15715 | 15738 | AACUAGACUCAAAAGAUUCAUCUC | GAGACAAAUCUUUUGAGUCUAGUU | Cleavage |  | 2 |
| sbi-miR6235-5p | SbNFY-A5 | 1 | -1 | 1 | 24 | 4514 | 4537 | UUGUGAGAGAAAAAUACUGUUGGC | UGAAAUAGUAUUUUUCUCUUACAA | Cleavage |  | 1 |
| sbi-miR169a | SbNFY-A1 | 1.5 | -1 | 1 | 21 | 12857 | 12877 | CAGCCAAGGAUGACUUGCCGA | UAGGCAAAUCAUUCUUGGCUG | Cleavage |  | 2 |
| sbi-miR169a | SbNFY-A8 | 1.5 | -1 | 1 | 21 | 7556 | 7576 | CAGCCAAGGAUGACUUGCCGA | UAGGCAAAUCAUUCUUGGCUG | Cleavage |  | 1 |
| sbi-miR169b | SbNFY-A1 | 1.5 | -1 | 1 | 21 | 12857 | 12877 | CAGCCAAGGAUGACUUGCCGG | UAGGCAAAUCAUUCUUGGCUG | Cleavage |  | 2 |
| sbi-miR169b | SbNFY-A8 | 1.5 | -1 | 1 | 21 | 7556 | 7576 | CAGCCAAGGAUGACUUGCCGG | UAGGCAAAUCAUUCUUGGCUG | Cleavage |  | 1 |
| sbi-miR169i | SbNFY-A1 | 1.5 | -1 | 1 | 21 | 12857 | 12877 | UAGCCAAGAAUGACUUGCCUA | UAGGCAAAUCAUUCUUGGCUG | Cleavage |  | 1 |
| sbi-miR169i | SbNFY-A8 | 1.5 | -1 | 1 | 21 | 7556 | 7576 | UAGCCAAGAAUGACUUGCCUA | UAGGCAAAUCAUUCUUGGCUG | Cleavage |  | 1 |
| sbi-miR169k | SbNFY-A1 | 1.5 | -1 | 1 | 21 | 12857 | 12877 | CAGCCAAGGAUGACUUGCCGG | UAGGCAAAUCAUUCUUGGCUG | Cleavage |  | 2 |
| sbi-miR169k | SbNFY-A8 | 1.5 | -1 | 1 | 21 | 7556 | 7576 | CAGCCAAGGAUGACUUGCCGG | UAGGCAAAUCAUUCUUGGCUG | Cleavage |  | 1 |
| sbi-miR5568g-3p | SbNFY-A2 | 1.5 | -1 | 1 | 21 | 11399 | 11419 | AAAACGUCUUAUAAUUUGGAG | AUUCUAAUUAUAAGACGUUUU | Cleavage |  | 2 |
| sbi-miR6225-5p | SbNFY-A5 | 1.5 | -1 | 1 | 24 | 6350 | 6373 | AACUAGACUCAAAAGAUUCAUCUC | GAGAUGAAUCUUUUGAGCCUAGUU | Cleavage |  | 1 |
| sbi-miR6225-5p | SbNFY-A3 | 1.5 | -1 | 1 | 24 | 13863 | 13886 | AACUAGACUCAAAAGAUUCAUCUC | GAGACGAAUCUUUUAAGUCUAGUU | Translation | | 1 |
| sbi-miR169a | SbNFY-A3 | 2 | -1 | 1 | 21 | 11082 | 11102 | CAGCCAAGGAUGACUUGCCGA | CUGGCAACUCAUCCUUGGCUU | Cleavage |  | 1 |
| sbi-miR169a | SbNFY-A2 | 2 | -1 | 1 | 21 | 16545 | 16565 | CAGCCAAGGAUGACUUGCCGA | GUGGCAACUCAUCCUUGGCUU | Cleavage |  | 2 |
| sbi-miR169a | SbNFY-A5 | 2 | -1 | 1 | 21 | 10864 | 10884 | CAGCCAAGGAUGACUUGCCGA | GUGGCAACUCAUCCUUGGCUU | Cleavage |  | 1 |
| sbi-miR169a | SbNFY-A6 | 2 | -1 | 1 | 21 | 11169 | 11189 | CAGCCAAGGAUGACUUGCCGA | CAGGCAAUUCAUCCUUGGCUU | Cleavage |  | 1 |
| sbi-miR169b | SbNFY-A3 | 2 | -1 | 1 | 21 | 11082 | 11102 | CAGCCAAGGAUGACUUGCCGG | CUGGCAACUCAUCCUUGGCUU | Cleavage |  | 1 |
| sbi-miR169b | SbNFY-A2 | 2 | -1 | 1 | 21 | 16545 | 16565 | CAGCCAAGGAUGACUUGCCGG | GUGGCAACUCAUCCUUGGCUU | Cleavage |  | 2 |
| sbi-miR169b | SbNFY-A5 | 2 | -1 | 1 | 21 | 10864 | 10884 | CAGCCAAGGAUGACUUGCCGG | GUGGCAACUCAUCCUUGGCUU | Cleavage |  | 1 |
| sbi-miR169b | SbNFY-A6 | 2 | -1 | 1 | 21 | 11169 | 11189 | CAGCCAAGGAUGACUUGCCGG | CAGGCAAUUCAUCCUUGGCUU | Cleavage |  | 1 |
| sbi-miR169c | SbNFY-A1 | 2 | -1 | 1 | 21 | 12857 | 12877 | UAGCCAAGGAUGACUUGCCUA | UAGGCAAAUCAUUCUUGGCUG | Cleavage |  | 1 |
| sbi-miR169c | SbNFY-A8 | 2 | -1 | 1 | 21 | 7556 | 7576 | UAGCCAAGGAUGACUUGCCUA | UAGGCAAAUCAUUCUUGGCUG | Cleavage |  | 1 |
| sbi-miR169c | SbNFY-A6 | 2 | -1 | 1 | 21 | 11169 | 11189 | UAGCCAAGGAUGACUUGCCUA | CAGGCAAUUCAUCCUUGGCUU | Cleavage |  | 1 |
| sbi-miR169c | SbNFY-A2 | 2 | -1 | 1 | 21 | 16545 | 16565 | UAGCCAAGGAUGACUUGCCUA | GUGGCAACUCAUCCUUGGCUU | Cleavage |  | 1 |
| sbi-miR169c | SbNFY-A3 | 2 | -1 | 1 | 21 | 11082 | 11102 | UAGCCAAGGAUGACUUGCCUA | CUGGCAACUCAUCCUUGGCUU | Cleavage |  | 1 |
| sbi-miR169c | SbNFY-A5 | 2 | -1 | 1 | 21 | 10864 | 10884 | UAGCCAAGGAUGACUUGCCUA | GUGGCAACUCAUCCUUGGCUU | Cleavage |  | 1 |
| sbi-miR169d-5p | SbNFY-A6 | 2 | -1 | 1 | 20 | 11170 | 11189 | UAGCCAAGGAUGACUUGCCU | AGGCAAUUCAUCCUUGGCUU | Cleavage |  | 1 |
| sbi-miR169d-5p | SbNFY-A1 | 2 | -1 | 1 | 20 | 12858 | 12877 | UAGCCAAGGAUGACUUGCCU | AGGCAAAUCAUUCUUGGCUG | Cleavage |  | 1 |
| sbi-miR169d-5p | SbNFY-A8 | 2 | -1 | 1 | 20 | 7557 | 7576 | UAGCCAAGGAUGACUUGCCU | AGGCAAAUCAUUCUUGGCUG | Cleavage |  | 1 |
| sbi-miR169d-5p | SbNFY-A2 | 2 | -1 | 1 | 20 | 16546 | 16565 | UAGCCAAGGAUGACUUGCCU | UGGCAACUCAUCCUUGGCUU | Cleavage |  | 1 |
| sbi-miR169d-5p | SbNFY-A3 | 2 | -1 | 1 | 20 | 11083 | 11102 | UAGCCAAGGAUGACUUGCCU | UGGCAACUCAUCCUUGGCUU | Cleavage |  | 1 |
| sbi-miR169d-5p | SbNFY-A5 | 2 | -1 | 1 | 20 | 10865 | 10884 | UAGCCAAGGAUGACUUGCCU | UGGCAACUCAUCCUUGGCUU | Cleavage |  | 1 |
| sbi-miR169e | SbNFY-A3 | 2 | -1 | 1 | 21 | 11082 | 11102 | UAGCCAAGGAUGACUUGCCGG | CUGGCAACUCAUCCUUGGCUU | Cleavage |  | 1 |
| sbi-miR169e | SbNFY-A2 | 2 | -1 | 1 | 21 | 16545 | 16565 | UAGCCAAGGAUGACUUGCCGG | GUGGCAACUCAUCCUUGGCUU | Cleavage |  | 1 |
| sbi-miR169e | SbNFY-A5 | 2 | -1 | 1 | 21 | 10864 | 10884 | UAGCCAAGGAUGACUUGCCGG | GUGGCAACUCAUCCUUGGCUU | Cleavage |  | 1 |
| sbi-miR169e | SbNFY-A6 | 2 | -1 | 1 | 21 | 11169 | 11189 | UAGCCAAGGAUGACUUGCCGG | CAGGCAAUUCAUCCUUGGCUU | Cleavage |  | 1 |
| sbi-miR169e | SbNFY-A1 | 2 | -1 | 1 | 21 | 12857 | 12877 | UAGCCAAGGAUGACUUGCCGG | UAGGCAAAUCAUUCUUGGCUG | Cleavage |  | 1 |
| sbi-miR169e | SbNFY-A8 | 2 | -1 | 1 | 21 | 7556 | 7576 | UAGCCAAGGAUGACUUGCCGG | UAGGCAAAUCAUUCUUGGCUG | Cleavage |  | 1 |
| sbi-miR169f | SbNFY-A6 | 2 | -1 | 1 | 21 | 11169 | 11189 | UAGCCAAGGAUGACUUGCCUG | CAGGCAAUUCAUCCUUGGCUU | Cleavage |  | 1 |
| sbi-miR169f | SbNFY-A1 | 2 | -1 | 1 | 21 | 12857 | 12877 | UAGCCAAGGAUGACUUGCCUG | UAGGCAAAUCAUUCUUGGCUG | Cleavage |  | 1 |
| sbi-miR169f | SbNFY-A8 | 2 | -1 | 1 | 21 | 7556 | 7576 | UAGCCAAGGAUGACUUGCCUG | UAGGCAAAUCAUUCUUGGCUG | Cleavage |  | 1 |
| sbi-miR169f | SbNFY-A3 | 2 | -1 | 1 | 21 | 11082 | 11102 | UAGCCAAGGAUGACUUGCCUG | CUGGCAACUCAUCCUUGGCUU | Cleavage |  | 1 |
| sbi-miR169f | SbNFY-A2 | 2 | -1 | 1 | 21 | 16545 | 16565 | UAGCCAAGGAUGACUUGCCUG | GUGGCAACUCAUCCUUGGCUU | Cleavage |  | 1 |
| sbi-miR169f | SbNFY-A5 | 2 | -1 | 1 | 21 | 10864 | 10884 | UAGCCAAGGAUGACUUGCCUG | GUGGCAACUCAUCCUUGGCUU | Cleavage |  | 1 |
| sbi-miR169g | SbNFY-A6 | 2 | -1 | 1 | 21 | 11169 | 11189 | UAGCCAAGGAUGACUUGCCUG | CAGGCAAUUCAUCCUUGGCUU | Cleavage |  | 1 |
| sbi-miR169g | SbNFY-A1 | 2 | -1 | 1 | 21 | 12857 | 12877 | UAGCCAAGGAUGACUUGCCUG | UAGGCAAAUCAUUCUUGGCUG | Cleavage |  | 1 |
| sbi-miR169g | SbNFY-A8 | 2 | -1 | 1 | 21 | 7556 | 7576 | UAGCCAAGGAUGACUUGCCUG | UAGGCAAAUCAUUCUUGGCUG | Cleavage |  | 1 |
| sbi-miR169g | SbNFY-A3 | 2 | -1 | 1 | 21 | 11082 | 11102 | UAGCCAAGGAUGACUUGCCUG | CUGGCAACUCAUCCUUGGCUU | Cleavage |  | 1 |
| sbi-miR169g | SbNFY-A2 | 2 | -1 | 1 | 21 | 16545 | 16565 | UAGCCAAGGAUGACUUGCCUG | GUGGCAACUCAUCCUUGGCUU | Cleavage |  | 1 |
| sbi-miR169g | SbNFY-A5 | 2 | -1 | 1 | 21 | 10864 | 10884 | UAGCCAAGGAUGACUUGCCUG | GUGGCAACUCAUCCUUGGCUU | Cleavage |  | 1 |
| sbi-miR169h | SbNFY-A1 | 2 | -1 | 1 | 21 | 12857 | 12877 | UAGCCAAGGAUGACUUGCCUA | UAGGCAAAUCAUUCUUGGCUG | Cleavage |  | 1 |
| sbi-miR169h | SbNFY-A8 | 2 | -1 | 1 | 21 | 7556 | 7576 | UAGCCAAGGAUGACUUGCCUA | UAGGCAAAUCAUUCUUGGCUG | Cleavage |  | 1 |
| sbi-miR169h | SbNFY-A6 | 2 | -1 | 1 | 21 | 11169 | 11189 | UAGCCAAGGAUGACUUGCCUA | CAGGCAAUUCAUCCUUGGCUU | Cleavage |  | 1 |
| sbi-miR169h | SbNFY-A2 | 2 | -1 | 1 | 21 | 16545 | 16565 | UAGCCAAGGAUGACUUGCCUA | GUGGCAACUCAUCCUUGGCUU | Cleavage |  | 1 |
| sbi-miR169h | SbNFY-A3 | 2 | -1 | 1 | 21 | 11082 | 11102 | UAGCCAAGGAUGACUUGCCUA | CUGGCAACUCAUCCUUGGCUU | Cleavage |  | 1 |
| sbi-miR169h | SbNFY-A5 | 2 | -1 | 1 | 21 | 10864 | 10884 | UAGCCAAGGAUGACUUGCCUA | GUGGCAACUCAUCCUUGGCUU | Cleavage |  | 1 |
| sbi-miR169i | SbNFY-A4 | 2 | -1 | 1 | 21 | 9350 | 9370 | UAGCCAAGAAUGACUUGCCUA | ACGGCAACUCAUUCUUGGCUC | Cleavage |  | 1 |
| sbi-miR169j | SbNFY-A3 | 2 | -1 | 1 | 21 | 11082 | 11102 | UAGCCAAGGAUGACUUGCCGG | CUGGCAACUCAUCCUUGGCUU | Cleavage |  | 1 |
| sbi-miR169j | SbNFY-A2 | 2 | -1 | 1 | 21 | 16545 | 16565 | UAGCCAAGGAUGACUUGCCGG | GUGGCAACUCAUCCUUGGCUU | Cleavage |  | 1 |
| sbi-miR169j | SbNFY-A5 | 2 | -1 | 1 | 21 | 10864 | 10884 | UAGCCAAGGAUGACUUGCCGG | GUGGCAACUCAUCCUUGGCUU | Cleavage |  | 1 |
| sbi-miR169j | SbNFY-A6 | 2 | -1 | 1 | 21 | 11169 | 11189 | UAGCCAAGGAUGACUUGCCGG | CAGGCAAUUCAUCCUUGGCUU | Cleavage |  | 1 |
| sbi-miR169j | SbNFY-A1 | 2 | -1 | 1 | 21 | 12857 | 12877 | UAGCCAAGGAUGACUUGCCGG | UAGGCAAAUCAUUCUUGGCUG | Cleavage |  | 1 |
| sbi-miR169j | SbNFY-A8 | 2 | -1 | 1 | 21 | 7556 | 7576 | UAGCCAAGGAUGACUUGCCGG | UAGGCAAAUCAUUCUUGGCUG | Cleavage |  | 1 |
| sbi-miR169k | SbNFY-A3 | 2 | -1 | 1 | 21 | 11082 | 11102 | CAGCCAAGGAUGACUUGCCGG | CUGGCAACUCAUCCUUGGCUU | Cleavage |  | 1 |
| sbi-miR169k | SbNFY-A2 | 2 | -1 | 1 | 21 | 16545 | 16565 | CAGCCAAGGAUGACUUGCCGG | GUGGCAACUCAUCCUUGGCUU | Cleavage |  | 2 |
| sbi-miR169k | SbNFY-A5 | 2 | -1 | 1 | 21 | 10864 | 10884 | CAGCCAAGGAUGACUUGCCGG | GUGGCAACUCAUCCUUGGCUU | Cleavage |  | 1 |
| sbi-miR169k | SbNFY-A6 | 2 | -1 | 1 | 21 | 11169 | 11189 | CAGCCAAGGAUGACUUGCCGG | CAGGCAAUUCAUCCUUGGCUU | Cleavage |  | 1 |
| sbi-miR169l | SbNFY-A6 | 2 | -1 | 1 | 21 | 11169 | 11189 | UAGCCAAGGAUGACUUGCCUG | CAGGCAAUUCAUCCUUGGCUU | Cleavage |  | 1 |
| sbi-miR169l | SbNFY-A1 | 2 | -1 | 1 | 21 | 12857 | 12877 | UAGCCAAGGAUGACUUGCCUG | UAGGCAAAUCAUUCUUGGCUG | Cleavage |  | 1 |
| sbi-miR169l | SbNFY-A8 | 2 | -1 | 1 | 21 | 7556 | 7576 | UAGCCAAGGAUGACUUGCCUG | UAGGCAAAUCAUUCUUGGCUG | Cleavage |  | 1 |
| sbi-miR169l | SbNFY-A3 | 2 | -1 | 1 | 21 | 11082 | 11102 | UAGCCAAGGAUGACUUGCCUG | CUGGCAACUCAUCCUUGGCUU | Cleavage |  | 1 |
| sbi-miR169l | SbNFY-A2 | 2 | -1 | 1 | 21 | 16545 | 16565 | UAGCCAAGGAUGACUUGCCUG | GUGGCAACUCAUCCUUGGCUU | Cleavage |  | 1 |
| sbi-miR169l | SbNFY-A5 | 2 | -1 | 1 | 21 | 10864 | 10884 | UAGCCAAGGAUGACUUGCCUG | GUGGCAACUCAUCCUUGGCUU | Cleavage |  | 1 |
| sbi-miR169m | SbNFY-A1 | 2 | -1 | 1 | 21 | 12857 | 12877 | UAGCCAAGGAUGACUUGCCUA | UAGGCAAAUCAUUCUUGGCUG | Cleavage |  | 1 |
| sbi-miR169m | SbNFY-A8 | 2 | -1 | 1 | 21 | 7556 | 7576 | UAGCCAAGGAUGACUUGCCUA | UAGGCAAAUCAUUCUUGGCUG | Cleavage |  | 1 |
| sbi-miR169m | SbNFY-A6 | 2 | -1 | 1 | 21 | 11169 | 11189 | UAGCCAAGGAUGACUUGCCUA | CAGGCAAUUCAUCCUUGGCUU | Cleavage |  | 1 |
| sbi-miR169m | SbNFY-A2 | 2 | -1 | 1 | 21 | 16545 | 16565 | UAGCCAAGGAUGACUUGCCUA | GUGGCAACUCAUCCUUGGCUU | Cleavage |  | 1 |
| sbi-miR169m | SbNFY-A3 | 2 | -1 | 1 | 21 | 11082 | 11102 | UAGCCAAGGAUGACUUGCCUA | CUGGCAACUCAUCCUUGGCUU | Cleavage |  | 1 |
| sbi-miR169m | SbNFY-A5 | 2 | -1 | 1 | 21 | 10864 | 10884 | UAGCCAAGGAUGACUUGCCUA | GUGGCAACUCAUCCUUGGCUU | Cleavage |  | 1 |
| sbi-miR169n | SbNFY-A1 | 2 | -1 | 1 | 21 | 12857 | 12877 | UAGCCAAGGAUGACUUGCCUA | UAGGCAAAUCAUUCUUGGCUG | Cleavage |  | 1 |
| sbi-miR169n | SbNFY-A8 | 2 | -1 | 1 | 21 | 7556 | 7576 | UAGCCAAGGAUGACUUGCCUA | UAGGCAAAUCAUUCUUGGCUG | Cleavage |  | 1 |
| sbi-miR169n | SbNFY-A6 | 2 | -1 | 1 | 21 | 11169 | 11189 | UAGCCAAGGAUGACUUGCCUA | CAGGCAAUUCAUCCUUGGCUU | Cleavage |  | 1 |
| sbi-miR169n | SbNFY-A2 | 2 | -1 | 1 | 21 | 16545 | 16565 | UAGCCAAGGAUGACUUGCCUA | GUGGCAACUCAUCCUUGGCUU | Cleavage |  | 1 |
| sbi-miR169n | SbNFY-A3 | 2 | -1 | 1 | 21 | 11082 | 11102 | UAGCCAAGGAUGACUUGCCUA | CUGGCAACUCAUCCUUGGCUU | Cleavage |  | 1 |
| sbi-miR169n | SbNFY-A5 | 2 | -1 | 1 | 21 | 10864 | 10884 | UAGCCAAGGAUGACUUGCCUA | GUGGCAACUCAUCCUUGGCUU | Cleavage |  | 1 |
| sbi-miR169o | SbNFY-A6 | 2 | -1 | 1 | 21 | 11169 | 11189 | UAGCCAAGGAUGAUUUGCCUG | CAGGCAAUUCAUCCUUGGCUU | Cleavage |  | 1 |
| sbi-miR169o | SbNFY-A3 | 2 | -1 | 1 | 21 | 11082 | 11102 | UAGCCAAGGAUGAUUUGCCUG | CUGGCAACUCAUCCUUGGCUU | Cleavage |  | 1 |
| sbi-miR169o | SbNFY-A2 | 2 | -1 | 1 | 21 | 16545 | 16565 | UAGCCAAGGAUGAUUUGCCUG | GUGGCAACUCAUCCUUGGCUU | Cleavage |  | 2 |
| sbi-miR169o | SbNFY-A5 | 2 | -1 | 1 | 21 | 10864 | 10884 | UAGCCAAGGAUGAUUUGCCUG | GUGGCAACUCAUCCUUGGCUU | Cleavage |  | 1 |
| sbi-miR169p | SbNFY-A1 | 2 | -1 | 1 | 21 | 12857 | 12877 | UAGCCAAGAAUGGCUUGCCUA | UAGGCAAAUCAUUCUUGGCUG | Cleavage |  | 1 |
| sbi-miR169p | SbNFY-A8 | 2 | -1 | 1 | 21 | 7556 | 7576 | UAGCCAAGAAUGGCUUGCCUA | UAGGCAAAUCAUUCUUGGCUG | Cleavage |  | 1 |
| sbi-miR169q | SbNFY-A1 | 2 | -1 | 1 | 21 | 12857 | 12877 | UAGCCAAGAAUGGCUUGCCUA | UAGGCAAAUCAUUCUUGGCUG | Cleavage |  | 1 |
| sbi-miR169q | SbNFY-A8 | 2 | -1 | 1 | 21 | 7556 | 7576 | UAGCCAAGAAUGGCUUGCCUA | UAGGCAAAUCAUUCUUGGCUG | Cleavage |  | 1 |
| sbi-miR5389 | SbNFY-A8 | 2 | -1 | 1 | 21 | 601 | 621 | GCUUGAGUUUAUCAGCCGAGU | AUUCGGCUGAUAAGCUCAAAC | Cleavage |  | 1 |
| sbi-miR5567 | SbNFY-A1 | 2 | -1 | 1 | 24 | 3538 | 3561 | UUAAUGAUUCAUGUAUGUGUCCAA | UACGGCACAUGCAUGAAGCAUUAA | Cleavage |  | 2 |
| sbi-miR5567 | SbNFY-A6 | 2 | -1 | 1 | 24 | 15645 | 15669 | UUAAUGAUUCAUGUAUGUGUC-CAA | UUGCGGCACAUGCAUGAAACAUUAA | Cleavage |  | 2 |
| sbi-miR5567 | SbNFY-A4 | 2 | -1 | 1 | 24 | 6784 | 6807 | UUAAUGAUUCAUGUAUGUGUCCAA | UACUACACAUGCAUGAAGCAUUAA | Cleavage |  | 3 |
| sbi-miR6225-3p | SbNFY-A3 | 2 | -1 | 1 | 24 | 15948 | 15971 | GAAACGAAUCUUUUAAGUCUAAUU | AACUAGGCUUAAAAGAUUCGUCUC | Cleavage |  | 5 |
| sbi-miR6235-5p | SbNFY-A1 | 2 | -1 | 1 | 24 | 1653 | 1676 | UUGUGAGAGAAAAAUACUGUUGGC | GUUAACAGUGUUUUUCUUUUAUAA | Cleavage |  | 3 |
| sbi-miR169a | SbNFY-A4 | 2.5 | -1 | 1 | 21 | 9350 | 9370 | CAGCCAAGGAUGACUUGCCGA | ACGGCAACUCAUUCUUGGCUC | Cleavage |  | 1 |
| sbi-miR169b | SbNFY-A4 | 2.5 | -1 | 1 | 21 | 9350 | 9370 | CAGCCAAGGAUGACUUGCCGG | ACGGCAACUCAUUCUUGGCUC | Cleavage |  | 1 |
| sbi-miR169c | SbNFY-A4 | 2.5 | -1 | 1 | 21 | 9350 | 9370 | UAGCCAAGGAUGACUUGCCUA | ACGGCAACUCAUUCUUGGCUC | Cleavage |  | 1 |
| sbi-miR169d-5p | SbNFY-A4 | 2.5 | -1 | 1 | 20 | 9351 | 9370 | UAGCCAAGGAUGACUUGCCU | CGGCAACUCAUUCUUGGCUC | Cleavage |  | 1 |
| sbi-miR169e | SbNFY-A4 | 2.5 | -1 | 1 | 21 | 9350 | 9370 | UAGCCAAGGAUGACUUGCCGG | ACGGCAACUCAUUCUUGGCUC | Cleavage |  | 1 |
| sbi-miR169f | SbNFY-A4 | 2.5 | -1 | 1 | 21 | 9350 | 9370 | UAGCCAAGGAUGACUUGCCUG | ACGGCAACUCAUUCUUGGCUC | Cleavage |  | 1 |
| sbi-miR169g | SbNFY-A4 | 2.5 | -1 | 1 | 21 | 9350 | 9370 | UAGCCAAGGAUGACUUGCCUG | ACGGCAACUCAUUCUUGGCUC | Cleavage |  | 1 |
| sbi-miR169h | SbNFY-A4 | 2.5 | -1 | 1 | 21 | 9350 | 9370 | UAGCCAAGGAUGACUUGCCUA | ACGGCAACUCAUUCUUGGCUC | Cleavage |  | 1 |
| sbi-miR169j | SbNFY-A4 | 2.5 | -1 | 1 | 21 | 9350 | 9370 | UAGCCAAGGAUGACUUGCCGG | ACGGCAACUCAUUCUUGGCUC | Cleavage |  | 1 |
| sbi-miR169k | SbNFY-A4 | 2.5 | -1 | 1 | 21 | 9350 | 9370 | CAGCCAAGGAUGACUUGCCGG | ACGGCAACUCAUUCUUGGCUC | Cleavage |  | 1 |
| sbi-miR169l | SbNFY-A4 | 2.5 | -1 | 1 | 21 | 9350 | 9370 | UAGCCAAGGAUGACUUGCCUG | ACGGCAACUCAUUCUUGGCUC | Cleavage |  | 1 |
| sbi-miR169m | SbNFY-A4 | 2.5 | -1 | 1 | 21 | 9350 | 9370 | UAGCCAAGGAUGACUUGCCUA | ACGGCAACUCAUUCUUGGCUC | Cleavage |  | 1 |
| sbi-miR169n | SbNFY-A4 | 2.5 | -1 | 1 | 21 | 9350 | 9370 | UAGCCAAGGAUGACUUGCCUA | ACGGCAACUCAUUCUUGGCUC | Cleavage |  | 1 |
| sbi-miR169o | SbNFY-A4 | 2.5 | -1 | 1 | 21 | 9350 | 9370 | UAGCCAAGGAUGAUUUGCCUG | ACGGCAACUCAUUCUUGGCUC | Cleavage |  | 3 |
| sbi-miR169p | SbNFY-A4 | 2.5 | -1 | 1 | 21 | 9350 | 9370 | UAGCCAAGAAUGGCUUGCCUA | ACGGCAACUCAUUCUUGGCUC | Cleavage |  | 1 |
| sbi-miR169q | SbNFY-A4 | 2.5 | -1 | 1 | 21 | 9350 | 9370 | UAGCCAAGAAUGGCUUGCCUA | ACGGCAACUCAUUCUUGGCUC | Cleavage |  | 1 |
| sbi-miR5567 | SbNFY-A7 | 2.5 | -1 | 1 | 24 | 7677 | 7700 | UUAAUGAUUCAUGUAUGUGUCCAA | UAUAGCACAUGUAUGAAGCAUUAA | Cleavage |  | 1 |
| sbi-miR5568d-3p | SbNFY-A2 | 2.5 | -1 | 1 | 21 | 11422 | 11442 | AAAGUUGUGUAUCUAGAAAAG | CUUUUAUAGGUACAUAGCUUU | Cleavage |  | 2 |
| sbi-miR6220-3p | SbNFY-A6 | 2.5 | -1 | 1 | 24 | 12002 | 12025 | AUGCCUUAUAAUUUGGGAUGGAGA | AAUCCAUCUCAAAUUAUAAGUCGU | Cleavage |  | 1 |
| sbi-miR6225-3p | SbNFY-A3 | 2.5 | -1 | 1 | 24 | 7081 | 7104 | GAAACGAAUCUUUUAAGUCUAAUU | AACUAGGCUUAAAAGAUUUGUCUC | Cleavage |  | 5 |
| sbi-miR6225-3p | SbNFY-A4 | 2.5 | -1 | 1 | 24 | 2745 | 2768 | GAAACGAAUCUUUUAAGUCUAAUU | AACUAGACUCAAAAGAUUCGUCUC | Cleavage |  | 3 |
| sbi-miR172e | SbNFY-A2 | 3 | -1 | 1 | 21 | 6385 | 6405 | UGAAUCUUGAUGAUGCUGCAC | GUUUAGCAUUAUUAAGAUUUA | Cleavage |  | 1 |
| sbi-miR5565g-3p | SbNFY-A5 | 3 | -1 | 1 | 24 | 15300 | 15323 | ACACAUGUGGAUUGAGAUGAAUAC | GUAUCUAUGUCAAUCCACUUGUGU | Cleavage |  | 1 |
| sbi-miR5567 | SbNFY-A3 | 3 | -1 | 1 | 24 | 13793 | 13816 | UUAAUGAUUCAUGUAUGUGUCCAA | UUGGACACAUGUAUGGAACAUUAA | Cleavage |  | 1 |
| sbi-miR5567 | SbNFY-A1 | 3 | -1 | 1 | 24 | 13707 | 13730 | UUAAUGAUUCAUGUAUGUGUCCAA | UUAGACACAUGCAUGGAAUAUUAA | Cleavage |  | 2 |
| sbi-miR5567 | SbNFY-A4 | 3 | -1 | 1 | 24 | 5565 | 5589 | UUAAUGAUUCAUGUAUGUGUC-CAA | UUGUGGCACAUGCAUGAGGUAUUAA | Cleavage |  | 3 |
| sbi-miR5568c-5p | SbNFY-A6 | 3 | -1 | 1 | 21 | 16495 | 16515 | UCUGUUCCAAAUUGUAAGUCG | UGACUUCUAUUUUGGAACAGA | Cleavage |  | 2 |
| sbi-miR5568c-5p | SbNFY-A6 | 3 | -1 | 1 | 21 | 12208 | 12228 | UCUGUUCCAAAUUGUAAGUCG | CUUCUUAUAAUUUGGGAUGGA | Cleavage |  | 2 |
| sbi-miR5568f-3p | SbNFY-A6 | 3 | -1 | 1 | 21 | 12003 | 12023 | GUCUUAUAAUUUGGAAUGGAG | AUCCAUCUCAAAUUAUAAGUC | Cleavage |  | 1 |
| sbi-miR5568g-3p | SbNFY-A6 | 3 | -1 | 1 | 21 | 12008 | 12028 | AAAACGUCUUAUAAUUUGGAG | UCUCAAAUUAUAAGUCGUUUG | Cleavage |  | 1 |
| sbi-miR6225-3p | SbNFY-A5 | 3 | -1 | 1 | 24 | 6595 | 6618 | GAAACGAAUCUUUUAAGUCUAAUU | AACUAGGCUCAAAAGAUUCGUCUC | Cleavage |  | 1 |
| sbi-miR6225-5p | SbNFY-A1 | 3 | -1 | 1 | 24 | 3609 | 3632 | AACUAGACUCAAAAGAUUCAUCUC | GAGACGAAUCUUUUGAUCCUAGUU | Cleavage |  | 2 |
| sbi-miR6225-5p | SbNFY-A4 | 3 | -1 | 1 | 24 | 6862 | 6885 | AACUAGACUCAAAAGAUUCAUCUC | CCAUUGAAUCUUUUGAGCCUACUU | Cleavage |  | 3 |
| sbi-miR169i | SbNFY-A6 | 3.5 | -1 | 1 | 21 | 11169 | 11189 | UAGCCAAGAAUGACUUGCCUA | CAGGCAAUUCAUCCUUGGCUU | Cleavage |  | 1 |
| sbi-miR169i | SbNFY-A2 | 3.5 | -1 | 1 | 21 | 16545 | 16565 | UAGCCAAGAAUGACUUGCCUA | GUGGCAACUCAUCCUUGGCUU | Cleavage |  | 1 |
| sbi-miR169i | SbNFY-A3 | 3.5 | -1 | 1 | 21 | 11082 | 11102 | UAGCCAAGAAUGACUUGCCUA | CUGGCAACUCAUCCUUGGCUU | Cleavage |  | 1 |
| sbi-miR169i | SbNFY-A5 | 3.5 | -1 | 1 | 21 | 10864 | 10884 | UAGCCAAGAAUGACUUGCCUA | GUGGCAACUCAUCCUUGGCUU | Cleavage |  | 1 |
| sbi-miR5565d | SbNFY-A5 | 3.5 | -1 | 1 | 24 | 15369 | 15392 | ACUUCAAUCCAUGUAUGUUGGUGU | ACUCCAACACAUGUGGAAUGAAGU | Cleavage |  | 1 |
| sbi-miR5565e | SbNFY-A1 | 3.5 | -1 | 1 | 19 | 4190 | 4208 | UUGUUUGGAUGUUGUCGGA | UCCUAUAAUAUUUAGACAA | Cleavage |  | 2 |
| sbi-miR5567 | SbNFY-A6 | 3.5 | -1 | 1 | 24 | 13438 | 13461 | UUAAUGAUUCAUGUAUGUGUCCAA | UACGGCACACGUAUGAAGCAUUAA | Cleavage |  | 2 |
| sbi-miR5568c-5p | SbNFY-A3 | 3.5 | -1 | 1 | 21 | 17326 | 17346 | UCUGUUCCAAAUUGUAAGUCG | CUAAUCAGGAUUUGGAACAGA | Cleavage |  | 2 |
| sbi-miR5568d-5p | SbNFY-A1 | 3.5 | -1 | 1 | 21 | 7627 | 7647 | UGGCUUUUCUAGAUACAUAGC | ACAAUAUAUCUAGGAAAGUUA | Cleavage |  | 1 |
| sbi-miR5568g-5p | SbNFY-A2 | 3.5 | -1 | 1 | 21 | 11488 | 11508 | CAAAUUAUAAGAUGUUUUGGC | GUGAAAACGUUUUAUGAUUUU | Cleavage |  | 2 |
| sbi-miR5568g-5p | SbNFY-A4 | 3.5 | -1 | 1 | 21 | 629 | 649 | CAAAUUAUAAGAUGUUUUGGC | UUUAAAAUGUUUUAUGAUUUU | Cleavage |  | 2 |
| sbi-miR5568g-5p | SbNFY-A6 | 3.5 | -1 | 1 | 21 | 12201 | 12221 | CAAAUUAUAAGAUGUUUUGGC | AGUUAAACUUCUUAUAAUUUG | Cleavage |  | 1 |
| sbi-miR6225-5p | SbNFY-A6 | 3.5 | -1 | 1 | 24 | 13504 | 13527 | AACUAGACUCAAAAGAUUCAUCUC | GAGACGAAUUUUUUAAGCCUAGUU | Translation | | 2 |
| sbi-miR6225-5p | SbNFY-A1 | 3.5 | -1 | 1 | 24 | 13778 | 13801 | AACUAGACUCAAAAGAUUCAUCUC | GAGACGGAUCUUUUAAGCCUAGUU | Translation | | 2 |
| sbi-miR6225-5p | SbNFY-A4 | 3.5 | -1 | 1 | 24 | 2377 | 2399 | AACUAGACUCAAAAGAUUCAUCUC | ACGAU-AAUCUUUUCAGUCUAGUU | Translation | | 3 |
| sbi-miR6232b-3p | SbNFY-A4 | 3.5 | -1 | 1 | 21 | 6762 | 6782 | AAUUCGAUGUACCAAAAAAGU | CAUUUUCUGUUACAUCGAAUC | Cleavage |  | 1 |
| sbi-miR6232b-5p | SbNFY-A1 | 3.5 | -1 | 1 | 21 | 8425 | 8445 | UUUUUGGUACAUUGAAUUUGC | UAAAAUUUAAUGUGACGAGAA | Cleavage |  | 3 |
| sbi-miR6232b-5p | SbNFY-A3 | 3.5 | -1 | 1 | 21 | 7174 | 7194 | UUUUUGGUACAUUGAAUUUGC | AAAAAUUCGAUGUGACGGAAA | Cleavage |  | 3 |
| sbi-miR6232b-5p | SbNFY-A4 | 3.5 | -1 | 1 | 21 | 7266 | 7286 | UUUUUGGUACAUUGAAUUUGC | UAAAAUUUAAUGUGACAGAGA | Cleavage |  | 3 |
| sbi-miR6235-3p | SbNFY-A4 | 3.5 | -1 | 1 | 24 | 14412 | 14435 | AACGAACAGUAUUUUUCUCUUACA | ACUAUGCUAAAAAUACUAUUCGUU | Cleavage |  | 1 |
| sbi-miR156d | SbNFY-A8 | 4 | -1 | 1 | 21 | 13759 | 13779 | UGACAGAAGAGAGAGAGCACA | UCUGCUUUCUUUCUGUUGUCU | Cleavage |  | 1 |
| sbi-miR156e | SbNFY-A1 | 4 | -1 | 1 | 20 | 11832 | 11851 | UGACAGAAGAGAGCGAGCAC | UUGCUCGUACUCUUUUGUUC | Cleavage |  | 1 |
| sbi-miR156e | SbNFY-A5 | 4 | -1 | 1 | 20 | 10659 | 10678 | UGACAGAAGAGAGCGAGCAC | CUGCACGCCCUCUUCUGACA | Cleavage |  | 1 |
| sbi-miR160a | SbNFY-A6 | 4 | -1 | 1 | 21 | 13676 | 13696 | UGCCUGGCUCCCUGUAUGCCA | GGCCAUACAGGCAGCCGGGCC | Translation | | 1 |
| sbi-miR160b | SbNFY-A6 | 4 | -1 | 1 | 21 | 13676 | 13696 | UGCCUGGCUCCCUGUAUGCCA | GGCCAUACAGGCAGCCGGGCC | Translation | | 1 |
| sbi-miR160c | SbNFY-A6 | 4 | -1 | 1 | 21 | 13676 | 13696 | UGCCUGGCUCCCUGUAUGCCA | GGCCAUACAGGCAGCCGGGCC | Translation | | 1 |
| sbi-miR160d | SbNFY-A6 | 4 | -1 | 1 | 21 | 13676 | 13696 | UGCCUGGCUCCCUGUAUGCCA | GGCCAUACAGGCAGCCGGGCC | Translation | | 1 |
| sbi-miR160e | SbNFY-A6 | 4 | -1 | 1 | 21 | 13676 | 13696 | UGCCUGGCUCCCUGUAUGCCA | GGCCAUACAGGCAGCCGGGCC | Translation | | 1 |
| sbi-miR169p | SbNFY-A6 | 4 | -1 | 1 | 21 | 11169 | 11189 | UAGCCAAGAAUGGCUUGCCUA | CAGGCAAUUCAUCCUUGGCUU | Cleavage |  | 1 |
| sbi-miR169p | SbNFY-A2 | 4 | -1 | 1 | 21 | 16545 | 16565 | UAGCCAAGAAUGGCUUGCCUA | GUGGCAACUCAUCCUUGGCUU | Cleavage |  | 1 |
| sbi-miR169p | SbNFY-A3 | 4 | -1 | 1 | 21 | 11082 | 11102 | UAGCCAAGAAUGGCUUGCCUA | CUGGCAACUCAUCCUUGGCUU | Cleavage |  | 1 |
| sbi-miR169p | SbNFY-A5 | 4 | -1 | 1 | 21 | 10864 | 10884 | UAGCCAAGAAUGGCUUGCCUA | GUGGCAACUCAUCCUUGGCUU | Cleavage |  | 1 |
| sbi-miR169q | SbNFY-A6 | 4 | -1 | 1 | 21 | 11169 | 11189 | UAGCCAAGAAUGGCUUGCCUA | CAGGCAAUUCAUCCUUGGCUU | Cleavage |  | 1 |
| sbi-miR169q | SbNFY-A2 | 4 | -1 | 1 | 21 | 16545 | 16565 | UAGCCAAGAAUGGCUUGCCUA | GUGGCAACUCAUCCUUGGCUU | Cleavage |  | 1 |
| sbi-miR169q | SbNFY-A3 | 4 | -1 | 1 | 21 | 11082 | 11102 | UAGCCAAGAAUGGCUUGCCUA | CUGGCAACUCAUCCUUGGCUU | Cleavage |  | 1 |
| sbi-miR169q | SbNFY-A5 | 4 | -1 | 1 | 21 | 10864 | 10884 | UAGCCAAGAAUGGCUUGCCUA | GUGGCAACUCAUCCUUGGCUU | Cleavage |  | 1 |
| sbi-miR172a | SbNFY-A2 | 4 | -1 | 1 | 20 | 6386 | 6405 | AGAAUCUUGAUGAUGCUGCA | UUUAGCAUUAUUAAGAUUUA | Cleavage |  | 1 |
| sbi-miR172b | SbNFY-A2 | 4 | -1 | 1 | 20 | 6386 | 6405 | GGAAUCUUGAUGAUGCUGCA | UUUAGCAUUAUUAAGAUUUA | Cleavage |  | 1 |
| sbi-miR172c | SbNFY-A2 | 4 | -1 | 1 | 20 | 6386 | 6405 | AGAAUCUUGAUGAUGCUGCA | UUUAGCAUUAUUAAGAUUUA | Cleavage |  | 1 |
| sbi-miR172d | SbNFY-A2 | 4 | -1 | 1 | 20 | 6386 | 6405 | AGAAUCUUGAUGAUGCUGCA | UUUAGCAUUAUUAAGAUUUA | Cleavage |  | 1 |
| sbi-miR408 | SbNFY-A1 | 4 | -1 | 1 | 21 | 3115 | 3135 | CUGCACUGCCUCUUCCCUGGC | UUCAGUGCAGGUGCAGUGCAG | Translation | | 1 |
| sbi-miR437a | SbNFY-A3 | 4 | -1 | 1 | 21 | 2533 | 2553 | AAAGUUAGAGAAGUUUGACUU | AUCUUUAGCUUCUCUAACUUG | Cleavage |  | 1 |
| sbi-miR437b | SbNFY-A3 | 4 | -1 | 1 | 21 | 2533 | 2553 | AAAGUUAGAGAAGUUUGACUU | AUCUUUAGCUUCUCUAACUUG | Cleavage |  | 1 |
| sbi-miR437c | SbNFY-A3 | 4 | -1 | 1 | 21 | 2533 | 2553 | AAAGUUAGAGAAGUUUGACUU | AUCUUUAGCUUCUCUAACUUG | Cleavage |  | 1 |
| sbi-miR437d | SbNFY-A3 | 4 | -1 | 1 | 21 | 2533 | 2553 | AAAGUUAGAGAAGUUUGACUU | AUCUUUAGCUUCUCUAACUUG | Cleavage |  | 1 |
| sbi-miR437e | SbNFY-A3 | 4 | -1 | 1 | 21 | 2533 | 2553 | AAAGUUAGAGAAGUUUGACUU | AUCUUUAGCUUCUCUAACUUG | Cleavage |  | 1 |
| sbi-miR437f | SbNFY-A3 | 4 | -1 | 1 | 21 | 2533 | 2553 | AAAGUUAGAGAAGUUUGACUU | AUCUUUAGCUUCUCUAACUUG | Cleavage |  | 1 |
| sbi-miR437g | SbNFY-A3 | 4 | -1 | 1 | 21 | 2533 | 2553 | AAAGUUAGAGAAGUUUGACUU | AUCUUUAGCUUCUCUAACUUG | Cleavage |  | 1 |
| sbi-miR437i | SbNFY-A3 | 4 | -1 | 1 | 21 | 2533 | 2553 | AAAGUUAGAGAAGUUUGACUU | AUCUUUAGCUUCUCUAACUUG | Cleavage |  | 1 |
| sbi-miR437j | SbNFY-A3 | 4 | -1 | 1 | 21 | 2533 | 2553 | AAAGUUAGAGAAGUUUGACUU | AUCUUUAGCUUCUCUAACUUG | Cleavage |  | 1 |
| sbi-miR437k | SbNFY-A3 | 4 | -1 | 1 | 21 | 2533 | 2553 | AAAGUUAGAGAAGUUUGACUU | AUCUUUAGCUUCUCUAACUUG | Cleavage |  | 1 |
| sbi-miR437l | SbNFY-A3 | 4 | -1 | 1 | 21 | 2533 | 2553 | AAAGUUAGAGAAGUUUGACUU | AUCUUUAGCUUCUCUAACUUG | Cleavage |  | 1 |
| sbi-miR437m | SbNFY-A3 | 4 | -1 | 1 | 21 | 2533 | 2553 | AAAGUUAGAGAAGUUUGACUU | AUCUUUAGCUUCUCUAACUUG | Cleavage |  | 1 |
| sbi-miR437n | SbNFY-A3 | 4 | -1 | 1 | 21 | 2533 | 2553 | AAAGUUAGAGAAGUUUGACUU | AUCUUUAGCUUCUCUAACUUG | Cleavage |  | 1 |
| sbi-miR437o | SbNFY-A3 | 4 | -1 | 1 | 21 | 2533 | 2553 | AAAGUUAGAGAAGUUUGACUU | AUCUUUAGCUUCUCUAACUUG | Cleavage |  | 1 |
| sbi-miR437p | SbNFY-A3 | 4 | -1 | 1 | 21 | 2533 | 2553 | AAAGUUAGAGAAGUUUGACUU | AUCUUUAGCUUCUCUAACUUG | Cleavage |  | 1 |
| sbi-miR437q | SbNFY-A3 | 4 | -1 | 1 | 21 | 2533 | 2553 | AAAGUUAGAGAAGUUUGACUU | AUCUUUAGCUUCUCUAACUUG | Cleavage |  | 1 |
| sbi-miR437r | SbNFY-A3 | 4 | -1 | 1 | 21 | 2533 | 2553 | AAAGUUAGAGAAGUUUGACUU | AUCUUUAGCUUCUCUAACUUG | Cleavage |  | 1 |
| sbi-miR437s | SbNFY-A3 | 4 | -1 | 1 | 21 | 2533 | 2553 | AAAGUUAGAGAAGUUUGACUU | AUCUUUAGCUUCUCUAACUUG | Cleavage |  | 1 |
| sbi-miR437t | SbNFY-A3 | 4 | -1 | 1 | 21 | 2533 | 2553 | AAAGUUAGAGAAGUUUGACUU | AUCUUUAGCUUCUCUAACUUG | Cleavage |  | 1 |
| sbi-miR437u | SbNFY-A3 | 4 | -1 | 1 | 21 | 2533 | 2553 | AAAGUUAGAGAAGUUUGACUU | AUCUUUAGCUUCUCUAACUUG | Cleavage |  | 1 |
| sbi-miR437v | SbNFY-A3 | 4 | -1 | 1 | 21 | 2533 | 2553 | AAAGUUAGAGAAGUUUGACUU | AUCUUUAGCUUCUCUAACUUG | Cleavage |  | 1 |
| sbi-miR437w | SbNFY-A3 | 4 | -1 | 1 | 21 | 2533 | 2553 | AAAGUUAGAGAAGUUUGACUU | AUCUUUAGCUUCUCUAACUUG | Cleavage |  | 1 |
| sbi-miR437x-3p | SbNFY-A3 | 4 | -1 | 1 | 24 | 7628 | 7651 | AUUUGACUGACACGGAUUCUAGGA | AAAAAGAGUUUGUGUCAGUUUAGU | Cleavage |  | 1 |
| sbi-miR5565a | SbNFY-A5 | 4 | -1 | 1 | 24 | 15301 | 15324 | AACACAUGUGGAUUGAGGCGAAUC | UAUCUAUGUCAAUCCACUUGUGUU | Cleavage |  | 1 |
| sbi-miR5565b | SbNFY-A5 | 4 | -1 | 1 | 24 | 15301 | 15324 | AACACAUGUGGAUUGAGGCGAAUC | UAUCUAUGUCAAUCCACUUGUGUU | Cleavage |  | 1 |
| sbi-miR5565c | SbNFY-A5 | 4 | -1 | 1 | 21 | 15304 | 15324 | UACACAUGUGGAUUGAGGUGA | CUAUGUCAAUCCACUUGUGUU | Cleavage |  | 1 |
| sbi-miR5565d | SbNFY-A8 | 4 | -1 | 1 | 24 | 940 | 963 | ACUUCAAUCCAUGUAUGUUGGUGU | ACGGGUACAUACGUGGAUUGACGA | Cleavage |  | 1 |
| sbi-miR5565e | SbNFY-A5 | 4 | -1 | 1 | 19 | 15396 | 15414 | UUGUUUGGAUGUUGUCGGA | UACAAGAGUAUCCAAACAA | Cleavage |  | 3 |
| sbi-miR5565g-5p | SbNFY-A5 | 4 | -1 | 1 | 24 | 15372 | 15395 | UUCACAUCAAUCCACAUAUGUUGG | CCAACACAUGUGGAAUGAAGUGAA | Translation | | 1 |
| sbi-miR5567 | SbNFY-A5 | 4 | -1 | 1 | 24 | 6279 | 6302 | UUAAUGAUUCAUGUAUGUGUCCAA | UUAGACGCAUGUAUGGAGUAUUAA | Cleavage |  | 1 |
| sbi-miR5568b-3p | SbNFY-A2 | 4 | -1 | 1 | 21 | 11419 | 11439 | ACUAUGUAUCUAGAAAAGCUA | UGACUUUUAUAGGUACAUAGC | Cleavage |  | 2 |
| sbi-miR5568e-3p | SbNFY-A2 | 4 | -1 | 1 | 21 | 11413 | 11433 | UAUCUAGAAAAGCUAAAACGU | ACGUUUUGACUUUUAUAGGUA | Cleavage |  | 1 |
| sbi-miR5568e-3p | SbNFY-A8 | 4 | -1 | 1 | 21 | 8322 | 8342 | UAUCUAGAAAAGCUAAAACGU | UUUUUUUGGUUUUUUUGGGUG | Cleavage |  | 1 |
| sbi-miR5568e-5p | SbNFY-A5 | 4 | -1 | 1 | 21 | 15116 | 15136 | GAUGUUUUGGGUUUUCUAGAU | CUCUUGUAAACCCAGAACACC | Cleavage |  | 1 |
| sbi-miR6219-3p | SbNFY-A1 | 4 | -1 | 1 | 24 | 15839 | 15862 | AGUCCCGAAACCUUAGUCCCGGCU | CCAUGGAGCUAGGGUUUUGGGGCC | Cleavage |  | 1 |
| sbi-miR6220-5p | SbNFY-A7 | 4 | -1 | 1 | 24 | 6634 | 6657 | CUCCAUCCUAAAUUAUAAGACAUU | ACUUGCAUAUAUUAUAGGAUGGAG | Translation | | 1 |
| sbi-miR6224a-5p | SbNFY-A6 | 4 | -1 | 1 | 21 | 12209 | 12229 | CUCCGUCCUAAUAUAUAAGGC | UUCUUAUAAUUUGGGAUGGAG | Cleavage |  | 1 |
| sbi-miR6224b-5p | SbNFY-A6 | 4 | -1 | 1 | 21 | 12209 | 12229 | CUCCGUCCUAAUAUAUAAGGC | UUCUUAUAAUUUGGGAUGGAG | Cleavage |  | 1 |
| sbi-miR6224c-5p | SbNFY-A6 | 4 | -1 | 1 | 21 | 12209 | 12229 | CUCCGUCCUAAUAUAUAAGGC | UUCUUAUAAUUUGGGAUGGAG | Cleavage |  | 1 |
| sbi-miR6225-3p | SbNFY-A3 | 4 | -1 | 1 | 24 | 12458 | 12481 | GAAACGAAUCUUUUAAGUCUAAUU | AAUUAGAUUUAAAAAAUUUAUUUC | Translation | | 5 |
| sbi-miR6225-3p | SbNFY-A1 | 4 | -1 | 1 | 24 | 16198 | 16221 | GAAACGAAUCUUUUAAGUCUAAUU | AACUAGGAUCAAAAGAUUCGUCUC | Cleavage |  | 4 |
| sbi-miR6225-3p | SbNFY-A8 | 4 | -1 | 1 | 24 | 16429 | 16452 | GAAACGAAUCUUUUAAGUCUAAUU | AACUAGUAUUAAAAGAUUUGUCUC | Cleavage |  | 2 |
| sbi-miR6225-5p | SbNFY-A4 | 4 | -1 | 1 | 24 | 5637 | 5660 | AACUAGACUCAAAAGAUUCAUCUC | GAGAUAAAUCUUUUGACUCUAAUU | Cleavage |  | 3 |
| sbi-miR6228-5p | SbNFY-A7 | 4 | -1 | 1 | 24 | 15239 | 15262 | UUCUAUCUCUAUUAAUUGUGUUGC | GAAAACUAAUCAUUAGAGAUAGAA | Cleavage |  | 1 |
| sbi-miR6232b-5p | SbNFY-A1 | 4 | -1 | 1 | 21 | 2246 | 2266 | UUUUUGGUACAUUGAAUUUGC | UAAAAUUUGAUGUGUCGAACA | Cleavage |  | 3 |
| sbi-miR6235-3p | SbNFY-A3 | 4 | -1 | 1 | 24 | 11256 | 11279 | AACGAACAGUAUUUUUCUCUUACA | GGUGCGGAAAGAAUGCUGUUCUUU | Cleavage |  | 1 |
| sbi-miR821e | SbNFY-A6 | 4 | -1 | 1 | 21 | 7703 | 7723 | AAGUCAUCAAAAUAAAAGUUG | UCGCUGCUAUUUUGAUGCCUU | Cleavage |  | 1 |
| sbi-miR156a | SbNFY-A1 | 4.5 | -1 | 1 | 20 | 11832 | 11851 | UGACAGAAGAGAGUGAGCAC | UUGCUCGUACUCUUUUGUUC | Cleavage |  | 1 |
| sbi-miR156a | SbNFY-A5 | 4.5 | -1 | 1 | 20 | 10659 | 10678 | UGACAGAAGAGAGUGAGCAC | CUGCACGCCCUCUUCUGACA | Cleavage |  | 1 |
| sbi-miR156b | SbNFY-A1 | 4.5 | -1 | 1 | 20 | 11832 | 11851 | UGACAGAAGAGAGUGAGCAC | UUGCUCGUACUCUUUUGUUC | Cleavage |  | 1 |
| sbi-miR156b | SbNFY-A5 | 4.5 | -1 | 1 | 20 | 10659 | 10678 | UGACAGAAGAGAGUGAGCAC | CUGCACGCCCUCUUCUGACA | Cleavage |  | 1 |
| sbi-miR156c | SbNFY-A1 | 4.5 | -1 | 1 | 20 | 11832 | 11851 | UGACAGAAGAGAGUGAGCAC | UUGCUCGUACUCUUUUGUUC | Cleavage |  | 1 |
| sbi-miR156c | SbNFY-A5 | 4.5 | -1 | 1 | 20 | 10659 | 10678 | UGACAGAAGAGAGUGAGCAC | CUGCACGCCCUCUUCUGACA | Cleavage |  | 1 |
| sbi-miR156d | SbNFY-A1 | 4.5 | -1 | 1 | 21 | 620 | 640 | UGACAGAAGAGAGAGAGCACA | AGUGCCCUUUUUUUUUUAUCA | Cleavage |  | 2 |
| sbi-miR156d | SbNFY-A7 | 4.5 | -1 | 1 | 21 | 3892 | 3912 | UGACAGAAGAGAGAGAGCACA | AUCGCUCUCUCGUUUCUGUUU | Translation | | 1 |
| sbi-miR156d | SbNFY-A3 | 4.5 | -1 | 1 | 21 | 10061 | 10081 | UGACAGAAGAGAGAGAGCACA | AUUACUUUCUCCUUUCUGUUG | Translation | | 1 |
| sbi-miR156f | SbNFY-A1 | 4.5 | -1 | 1 | 20 | 11832 | 11851 | UGACAGAAGAGAGUGAGCAC | UUGCUCGUACUCUUUUGUUC | Cleavage |  | 1 |
| sbi-miR156f | SbNFY-A5 | 4.5 | -1 | 1 | 20 | 10659 | 10678 | UGACAGAAGAGAGUGAGCAC | CUGCACGCCCUCUUCUGACA | Cleavage |  | 1 |
| sbi-miR156g | SbNFY-A1 | 4.5 | -1 | 1 | 20 | 11832 | 11851 | UGACAGAAGAGAGUGAGCAC | UUGCUCGUACUCUUUUGUUC | Cleavage |  | 1 |
| sbi-miR156g | SbNFY-A5 | 4.5 | -1 | 1 | 20 | 10659 | 10678 | UGACAGAAGAGAGUGAGCAC | CUGCACGCCCUCUUCUGACA | Cleavage |  | 1 |
| sbi-miR156h | SbNFY-A1 | 4.5 | -1 | 1 | 20 | 11832 | 11851 | UGACAGAAGAGAGUGAGCAC | UUGCUCGUACUCUUUUGUUC | Cleavage |  | 1 |
| sbi-miR156h | SbNFY-A5 | 4.5 | -1 | 1 | 20 | 10659 | 10678 | UGACAGAAGAGAGUGAGCAC | CUGCACGCCCUCUUCUGACA | Cleavage |  | 1 |
| sbi-miR156i | SbNFY-A1 | 4.5 | -1 | 1 | 20 | 11832 | 11851 | UGACAGAAGAGAGUGAGCAC | UUGCUCGUACUCUUUUGUUC | Cleavage |  | 1 |
| sbi-miR156i | SbNFY-A5 | 4.5 | -1 | 1 | 20 | 10659 | 10678 | UGACAGAAGAGAGUGAGCAC | CUGCACGCCCUCUUCUGACA | Cleavage |  | 1 |
| sbi-miR159a | SbNFY-A7 | 4.5 | -1 | 1 | 21 | 8593 | 8613 | UUUGGAUUGAAGGGAGCUCUG | GGGUGCUCCCAUGGAUCCAAA | Translation | | 1 |
| sbi-miR159a | SbNFY-A4 | 4.5 | -1 | 1 | 21 | 4071 | 4091 | UUUGGAUUGAAGGGAGCUCUG | UUGAGAAAUCUUUGAUCCAAA | Cleavage |  | 1 |
| sbi-miR160a | SbNFY-A3 | 4.5 | -1 | 1 | 21 | 15622 | 15642 | UGCCUGGCUCCCUGUAUGCCA | UGGCAGGCAGGCAGCCAGACA | Translation | | 3 |
| sbi-miR160b | SbNFY-A3 | 4.5 | -1 | 1 | 21 | 15622 | 15642 | UGCCUGGCUCCCUGUAUGCCA | UGGCAGGCAGGCAGCCAGACA | Translation | | 3 |
| sbi-miR160c | SbNFY-A3 | 4.5 | -1 | 1 | 21 | 15622 | 15642 | UGCCUGGCUCCCUGUAUGCCA | UGGCAGGCAGGCAGCCAGACA | Translation | | 3 |
| sbi-miR160d | SbNFY-A3 | 4.5 | -1 | 1 | 21 | 15622 | 15642 | UGCCUGGCUCCCUGUAUGCCA | UGGCAGGCAGGCAGCCAGACA | Translation | | 3 |
| sbi-miR160e | SbNFY-A3 | 4.5 | -1 | 1 | 21 | 15622 | 15642 | UGCCUGGCUCCCUGUAUGCCA | UGGCAGGCAGGCAGCCAGACA | Translation | | 3 |
| sbi-miR164c | SbNFY-A7 | 4.5 | -1 | 1 | 21 | 1807 | 1827 | UGGAGAAGCAGGACACGUGAG | UCAACGAGUCUUCCUUCUCCG | Cleavage |  | 1 |
| sbi-miR167a | SbNFY-A2 | 4.5 | -1 | 1 | 21 | 11373 | 11393 | UGAAGCUGCCAGCAUGAUCUA | AUGAUUAUUUUGGAAGUUUCA | Cleavage |  | 1 |
| sbi-miR167b | SbNFY-A2 | 4.5 | -1 | 1 | 21 | 11373 | 11393 | UGAAGCUGCCAGCAUGAUCUA | AUGAUUAUUUUGGAAGUUUCA | Cleavage |  | 1 |
| sbi-miR167c | SbNFY-A2 | 4.5 | -1 | 1 | 21 | 11373 | 11393 | UGAAGCUGCCAGCAUGAUCUG | AUGAUUAUUUUGGAAGUUUCA | Cleavage |  | 1 |
| sbi-miR167d | SbNFY-A2 | 4.5 | -1 | 1 | 21 | 11373 | 11393 | UGAAGCUGCCAGCAUGAUCUG | AUGAUUAUUUUGGAAGUUUCA | Cleavage |  | 1 |
| sbi-miR167e | SbNFY-A2 | 4.5 | -1 | 1 | 21 | 11373 | 11393 | UGAAGCUGCCAGCAUGAUCUG | AUGAUUAUUUUGGAAGUUUCA | Cleavage |  | 1 |
| sbi-miR167f | SbNFY-A2 | 4.5 | -1 | 1 | 21 | 11373 | 11393 | UGAAGCUGCCAGCAUGAUCUG | AUGAUUAUUUUGGAAGUUUCA | Cleavage |  | 1 |
| sbi-miR167g | SbNFY-A2 | 4.5 | -1 | 1 | 21 | 11373 | 11393 | UGAAGCUGCCAGCAUGAUCUG | AUGAUUAUUUUGGAAGUUUCA | Cleavage |  | 1 |
| sbi-miR167h | SbNFY-A2 | 4.5 | -1 | 1 | 21 | 11373 | 11393 | UGAAGCUGCCAGCAUGAUCUG | AUGAUUAUUUUGGAAGUUUCA | Cleavage |  | 1 |
| sbi-miR167i | SbNFY-A2 | 4.5 | -1 | 1 | 21 | 11373 | 11393 | UGAAGCUGCCAGCAUGAUCUA | AUGAUUAUUUUGGAAGUUUCA | Cleavage |  | 1 |
| sbi-miR168 | SbNFY-A4 | 4.5 | -1 | 1 | 21 | 6057 | 6077 | UCGCUUGGUGCAGAUCGGGAC | GGCUCGGCUUCCACCAGGCGA | Translation | | 2 |
| sbi-miR169o | SbNFY-A2 | 4.5 | -1 | 1 | 21 | 4373 | 4393 | UAGCCAAGGAUGAUUUGCCUG | GGGGACAAUCAUCAUUGGUUG | Cleavage |  | 2 |
| sbi-miR393a | SbNFY-A8 | 4.5 | -1 | 1 | 21 | 12483 | 12503 | UCCAAAGGGAUCGCAUUGAUC | UUUUAAUUCGAUCUCUUUGUU | Cleavage |  | 1 |
| sbi-miR393b | SbNFY-A8 | 4.5 | -1 | 1 | 21 | 12483 | 12503 | UCCAAAGGGAUCGCAUUGAUC | UUUUAAUUCGAUCUCUUUGUU | Cleavage |  | 1 |
| sbi-miR395k | SbNFY-A1 | 4.5 | -1 | 1 | 21 | 10155 | 10175 | GUGAAGUGUUUGGAGGAACUC | UUGUUCCUCCAAGAACUGCAG | Cleavage |  | 2 |
| sbi-miR395l | SbNFY-A1 | 4.5 | -1 | 1 | 21 | 10155 | 10175 | GUGAAGUGCUUGGGGGAACUC | UUGUUCCUCCAAGAACUGCAG | Cleavage |  | 1 |
| sbi-miR397-5p | SbNFY-A8 | 4.5 | -1 | 1 | 21 | 6890 | 6910 | UCAUUGAGUGCAGCGUUGAUG | UGUUAGUCCUGCAUUUGAUGG | Cleavage |  | 1 |
| sbi-miR399g | SbNFY-A4 | 4.5 | -1 | 1 | 21 | 12605 | 12625 | UGCCAAAGGAAAUUUGCCCCG | AGCAAUAAAUUUUUUUUGGCA | Cleavage |  | 1 |
| sbi-miR437x-5p | SbNFY-A1 | 4.5 | -1 | 1 | 24 | 2060 | 2083 | UAGAGUUGUCCUAAGUCAAACUUU | UAAACAUCACUUAGGGCAAUCCUA | Cleavage |  | 2 |
| sbi-miR528 | SbNFY-A7 | 4.5 | -1 | 1 | 21 | 6358 | 6378 | UGGAAGGGGCAUGCAGAGGAG | CGAGUCUGCGUGCUUCUUCCU | Cleavage |  | 2 |
| sbi-miR528 | SbNFY-A7 | 4.5 | -1 | 1 | 21 | 6181 | 6201 | UGGAAGGGGCAUGCAGAGGAG | UAUCUCUGCAAGUCUCUUGCA | Translation | | 2 |
| sbi-miR528 | SbNFY-A2 | 4.5 | -1 | 1 | 21 | 3819 | 3839 | UGGAAGGGGCAUGCAGAGGAG | GCCCUUUGUAUUCUCCUUUCC | Translation | | 2 |
| sbi-miR528 | SbNFY-A5 | 4.5 | -1 | 1 | 21 | 10339 | 10359 | UGGAAGGGGCAUGCAGAGGAG | GGCUUCUGCAUCACUCUUUCA | Translation | | 2 |
| sbi-miR528 | SbNFY-A6 | 4.5 | -1 | 1 | 21 | 8538 | 8558 | UGGAAGGGGCAUGCAGAGGAG | UGUCUAUGCAUGUUCCUUUUU | Cleavage |  | 1 |
| sbi-miR529 | SbNFY-A5 | 4.5 | -1 | 1 | 20 | 12539 | 12558 | CUGUACCCUCUCUCUUCUUC | AAAGAACAGAGAGGCAGCAG | Cleavage |  | 1 |
| sbi-miR5385 | SbNFY-A7 | 4.5 | -1 | 1 | 22 | 2985 | 3006 | ACCACCAACCCCACCGCUUCUC | GGCAUGCAUCGGGGUUGGUGGU | Cleavage |  | 2 |
| sbi-miR5385 | SbNFY-A7 | 4.5 | -1 | 1 | 22 | 4227 | 4248 | ACCACCAACCCCACCGCUUCUC | GGCAUGCAUCGGGGUUGGUGGU | Cleavage |  | 2 |
| sbi-miR5565a | SbNFY-A6 | 4.5 | -1 | 1 | 24 | 12706 | 12729 | AACACAUGUGGAUUGAGGCGAAUC | UAUGGGCCGUGAUACAUGUGUGUU | Translation | | 1 |
| sbi-miR5565b | SbNFY-A6 | 4.5 | -1 | 1 | 24 | 12706 | 12729 | AACACAUGUGGAUUGAGGCGAAUC | UAUGGGCCGUGAUACAUGUGUGUU | Translation | | 1 |
| sbi-miR5565c | SbNFY-A6 | 4.5 | -1 | 1 | 21 | 8105 | 8125 | UACACAUGUGGAUUGAGGUGA | UUUUCUCUUUCCAUAUGUGUA | Cleavage |  | 1 |
| sbi-miR5565d | SbNFY-A2 | 4.5 | -1 | 1 | 24 | 17782 | 17804 | ACUUCAAUCCAUGUAUGUUGGUGU | UUAUCAA-ACACAUGGAUGGAAGU | Cleavage |  | 1 |
| sbi-miR5565e | SbNFY-A5 | 4.5 | -1 | 1 | 19 | 17215 | 17233 | UUGUUUGGAUGUUGUCGGA | GGCGACGACGACCAAACAA | Cleavage |  | 3 |
| sbi-miR5565e | SbNFY-A4 | 4.5 | -1 | 1 | 19 | 12272 | 12290 | UUGUUUGGAUGUUGUCGGA | GUCGCCAGCAUUCAAACAU | Cleavage |  | 2 |
| sbi-miR5565e | SbNFY-A4 | 4.5 | -1 | 1 | 19 | 2441 | 2459 | UUGUUUGGAUGUUGUCGGA | UACAACAACAAUUAAACAA | Cleavage |  | 2 |
| sbi-miR5565f | SbNFY-A6 | 4.5 | -1 | 1 | 20 | 12129 | 12148 | UAGUCGGAUUUAUAUCAAUC | GAAUGAAUUAAAUUUGACUA | Cleavage |  | 2 |
| sbi-miR5565f | SbNFY-A8 | 4.5 | -1 | 1 | 20 | 16963 | 16982 | UAGUCGGAUUUAUAUCAAUC | UGUUGAUGUAGUUUUGAUUA | Cleavage |  | 1 |
| sbi-miR5565g-3p | SbNFY-A2 | 4.5 | -1 | 1 | 24 | 9372 | 9394 | ACACAUGUGGAUUGAGAUGAAUAC | GUAU-CAAAUCAAUUUACACGUGU | Cleavage |  | 1 |
| sbi-miR5567 | SbNFY-A4 | 4.5 | -1 | 1 | 24 | 6946 | 6969 | UUAAUGAUUCAUGUAUGUGUCCAA | UGAAUUAUGGACAUGAAUUAUUGU | Cleavage |  | 3 |
| sbi-miR5568a | SbNFY-A8 | 4.5 | -1 | 1 | 21 | 3962 | 3982 | CAGAGCGACUUACAAUUUGGA | GCCAAAUAGAAAGUUGCCCUG | Cleavage |  | 1 |
| sbi-miR5568b-5p | SbNFY-A1 | 4.5 | -1 | 1 | 21 | 7622 | 7642 | UUUCUAGGUACAUAGCUUUUG | UAAAAACAAUAUAUCUAGGAA | Translation | | 1 |
| sbi-miR5568c-3p | SbNFY-A6 | 4.5 | -1 | 1 | 21 | 12002 | 12022 | ACUUACAGUUUGGAACGGAGG | AAUCCAUCUCAAAUUAUAAGU | Cleavage |  | 1 |
| sbi-miR5568d-5p | SbNFY-A2 | 4.5 | -1 | 1 | 21 | 11471 | 11491 | UGGCUUUUCUAGAUACAUAGC | AAAAUGUAUCUAAAAAAGUGA | Cleavage |  | 1 |
| sbi-miR5568e-3p | SbNFY-A3 | 4.5 | -1 | 1 | 21 | 837 | 857 | UAUCUAGAAAAGCUAAAACGU | ACCUUUCGACUUUUCUGGAUA | Cleavage |  | 1 |
| sbi-miR5568e-3p | SbNFY-A5 | 4.5 | -1 | 1 | 21 | 10236 | 10256 | UAUCUAGAAAAGCUAAAACGU | GCGCUUAACUUUUUUUAGAUA | Cleavage |  | 1 |
| sbi-miR5568f-3p | SbNFY-A5 | 4.5 | -1 | 1 | 21 | 16310 | 16330 | GUCUUAUAAUUUGGAAUGGAG | CUCCAUUACAAUUUACAAGGC | Translation | | 1 |
| sbi-miR5568f-5p | SbNFY-A3 | 4.5 | -1 | 1 | 21 | 7692 | 7712 | UCCAUUCCAAAUUGUAAGAUG | UGGAAAGCAAUUUGGAAUGGA | Cleavage |  | 2 |
| sbi-miR5568g-3p | SbNFY-A3 | 4.5 | -1 | 1 | 21 | 17624 | 17644 | AAAACGUCUUAUAAUUUGGAG | GCCCAAAUUAUUGUGUGUUUU | Translation | | 1 |
| sbi-miR5568g-5p | SbNFY-A2 | 4.5 | -1 | 1 | 21 | 13516 | 13536 | CAAAUUAUAAGAUGUUUUGGC | UUAUGAACAUUUUAUGAUUUU | Cleavage |  | 2 |
| sbi-miR5568g-5p | SbNFY-A4 | 4.5 | -1 | 1 | 21 | 2848 | 2868 | CAAAUUAUAAGAUGUUUUGGC | GAUAAAAAAUCUUGUAAAUUU | Cleavage |  | 2 |
| sbi-miR5570 | SbNFY-A8 | 4.5 | -1 | 1 | 21 | 9703 | 9723 | AAAAGACAAAUCAGCAUGUCA | CCUCUUGAUGAUUUGUUUUUA | Cleavage |  | 1 |
| sbi-miR6217a-3p | SbNFY-A5 | 4.5 | -1 | 1 | 24 | 4139 | 4162 | AAAAUUAUCGUAAAUAGAGGUGGC | CAUUUUUCUGGUUAUGAUAGUCUU | Cleavage |  | 1 |
| sbi-miR6217b-3p | SbNFY-A5 | 4.5 | -1 | 1 | 24 | 4139 | 4162 | AAAAUUAUCGUAAAUAGAGGUGGC | CAUUUUUCUGGUUAUGAUAGUCUU | Cleavage |  | 1 |
| sbi-miR6224a-5p | SbNFY-A7 | 4.5 | -1 | 1 | 21 | 6637 | 6657 | CUCCGUCCUAAUAUAUAAGGC | UGCAUAUAUUAUAGGAUGGAG | Translation | | 1 |
| sbi-miR6224b-5p | SbNFY-A7 | 4.5 | -1 | 1 | 21 | 6637 | 6657 | CUCCGUCCUAAUAUAUAAGGC | UGCAUAUAUUAUAGGAUGGAG | Translation | | 1 |
| sbi-miR6224c-5p | SbNFY-A7 | 4.5 | -1 | 1 | 21 | 6637 | 6657 | CUCCGUCCUAAUAUAUAAGGC | UGCAUAUAUUAUAGGAUGGAG | Translation | | 1 |
| sbi-miR6225-3p | SbNFY-A3 | 4.5 | -1 | 1 | 24 | 16900 | 16923 | GAAACGAAUCUUUUAAGUCUAAUU | AACUAGGCUCAAAAGAUUCAUCUC | Cleavage |  | 5 |
| sbi-miR6225-3p | SbNFY-A3 | 4.5 | -1 | 1 | 24 | 3264 | 3287 | GAAACGAAUCUUUUAAGUCUAAUU | ACUGGGCUUUAAAAAACUCGUUUC | Translation | | 5 |
| sbi-miR6225-3p | SbNFY-A4 | 4.5 | -1 | 1 | 24 | 17308 | 17331 | GAAACGAAUCUUUUAAGUCUAAUU | AAGUAGGCUCAAAAGAUUCAUCUC | Cleavage |  | 3 |
| sbi-miR6225-3p | SbNFY-A4 | 4.5 | -1 | 1 | 24 | 3862 | 3885 | GAAACGAAUCUUUUAAGUCUAAUU | UGCAAGAGAUCAAAGAUUCGUUCC | Cleavage |  | 3 |
| sbi-miR6225-3p | SbNFY-A1 | 4.5 | -1 | 1 | 24 | 8333 | 8356 | GAAACGAAUCUUUUAAGUCUAAUU | AAUGAGGCUUAAAAGUUUUGUCUU | Cleavage |  | 4 |
| sbi-miR6226-3p | SbNFY-A6 | 4.5 | -1 | 1 | 24 | 3463 | 3486 | GAUUAGUCACGAUUAGUCGUCCGA | CACGGUGAAUAAUCGUGACUCGUG | Cleavage |  | 1 |
| sbi-miR6227-3p | SbNFY-A3 | 4.5 | -1 | 1 | 22 | 620 | 641 | CUCACAACACUUGCUAUUUGGG | AAAAAGCAGCAGGUGUUGUCAC | Cleavage |  | 1 |
| sbi-miR6229-5p | SbNFY-A7 | 4.5 | -1 | 1 | 24 | 3512 | 3535 | AUUCUCACUUGGGCGACGGAAAGG | CGCGGUUGUCCUUCAAGUGUGAAU | Cleavage |  | 1 |
| sbi-miR6232a-5p | SbNFY-A2 | 4.5 | -1 | 1 | 24 | 2398 | 2421 | GUCGCUUUGACUUUUUUGGUACAU | AUCAUCCAAAAGAGUUGUAGCUAC | Cleavage |  | 1 |
| sbi-miR6232b-5p | SbNFY-A1 | 4.5 | -1 | 1 | 21 | 3339 | 3359 | UUUUUGGUACAUUGAAUUUGC | CAAGAUUCGAUGUGACGGGAA | Cleavage |  | 3 |
| sbi-miR6232b-5p | SbNFY-A3 | 4.5 | -1 | 1 | 21 | 16993 | 17013 | UUUUUGGUACAUUGAAUUUGC | AAAGAUUCGAUGUGACGGAGA | Cleavage |  | 3 |
| sbi-miR6232b-5p | SbNFY-A4 | 4.5 | -1 | 1 | 21 | 606 | 626 | UUUUUGGUACAUUGAAUUUGC | UAAGAUUUGAUGUGAUGAAAA | Cleavage |  | 3 |
| sbi-miR6232b-5p | SbNFY-A4 | 4.5 | -1 | 1 | 21 | 17400 | 17420 | UUUUUGGUACAUUGAAUUUGC | CAAAAUUUGAUGUGACAGGGA | Cleavage |  | 3 |
| sbi-miR6232b-5p | SbNFY-A5 | 4.5 | -1 | 1 | 21 | 10600 | 10620 | UUUUUGGUACAUUGAAUUUGC | AGUAGUUCAAUGUGCUCAAAG | Cleavage |  | 3 |
| sbi-miR6232b-5p | SbNFY-A5 | 4.5 | -1 | 1 | 21 | 6688 | 6708 | UUUUUGGUACAUUGAAUUUGC | AAAGAUUCGAUGUGACGGAGA | Cleavage |  | 3 |
| sbi-miR6232b-5p | SbNFY-A5 | 4.5 | -1 | 1 | 21 | 8849 | 8869 | UUUUUGGUACAUUGAAUUUGC | UAGAAUUGGGUGUAUCGAAAU | Cleavage |  | 3 |
| sbi-miR6232b-5p | SbNFY-A8 | 4.5 | -1 | 1 | 21 | 16522 | 16542 | UUUUUGGUACAUUGAAUUUGC | UAAAAUUUGAUGUGACGGAGA | Cleavage |  | 1 |
| sbi-miR6233-3p | SbNFY-A4 | 4.5 | -1 | 1 | 24 | 12499 | 12522 | CAAGUUUGGUUUUGGUAAUUAAUG | AAAAAAUCACCAAAUUUAAACUUC | Translation | | 1 |
| sbi-miR6233-3p | SbNFY-A7 | 4.5 | -1 | 1 | 24 | 3781 | 3804 | CAAGUUUGGUUUUGGUAAUUAAUG | UAUGUGUUGUGAAAAACAAAUUUG | Cleavage |  | 1 |
| sbi-miR6233-5p | SbNFY-A7 | 4.5 | -1 | 1 | 24 | 5874 | 5897 | UGUUGAGGCUGGAGCGAAACUCGG | CUUUAUUUCAUUCUAGCUUCGCCA | Cleavage |  | 1 |
| sbi-miR6234a-5p | SbNFY-A5 | 4.5 | -1 | 1 | 24 | 1205 | 1228 | AAGUGUGUUCCUCUAUUUGACGCU | CAUAGGGAAUAGAGGAGCCUAUUU | Cleavage |  | 1 |
| sbi-miR6234a-5p | SbNFY-A4 | 4.5 | -1 | 1 | 24 | 16159 | 16182 | AAGUGUGUUCCUCUAUUUGACGCU | GCCGACACAUUGAGGAAUGUGUUU | Cleavage |  | 1 |
| sbi-miR6234b-5p | SbNFY-A5 | 4.5 | -1 | 1 | 24 | 1205 | 1228 | AAGUGUGUUCCUCUAUUUGACGCU | CAUAGGGAAUAGAGGAGCCUAUUU | Cleavage |  | 1 |
| sbi-miR6234b-5p | SbNFY-A4 | 4.5 | -1 | 1 | 24 | 16159 | 16182 | AAGUGUGUUCCUCUAUUUGACGCU | GCCGACACAUUGAGGAAUGUGUUU | Cleavage |  | 1 |
| sbi-miR6235-5p | SbNFY-A3 | 4.5 | -1 | 1 | 24 | 9327 | 9350 | UUGUGAGAGAAAAAUACUGUUGGC | UCAAAUUGUUUUUUUUUUUCGCGA | Cleavage |  | 2 |
| sbi-miR6235-5p | SbNFY-A3 | 4.5 | -1 | 1 | 24 | 12681 | 12704 | UUGUGAGAGAAAAAUACUGUUGGC | CCAAACAGGCCUUUUCUCUCACGU | Cleavage |  | 2 |
| sbi-miR821a | SbNFY-A6 | 4.5 | -1 | 1 | 21 | 6599 | 6619 | AAGUCAUCAACAUAAAAGUUG | AUACUUCUAUGUUAAUGAUAU | Cleavage |  | 1 |
| sbi-miR821c | SbNFY-A6 | 4.5 | -1 | 1 | 21 | 6599 | 6619 | AAGUCAUCAACAUAAAAGUUG | AUACUUCUAUGUUAAUGAUAU | Cleavage |  | 1 |
| sbi-miR821d | SbNFY-A8 | 4.5 | -1 | 1 | 21 | 7841 | 7861 | AAGUCAUCAACAACAAAGUUG | CGGAUUUGUUGGUGAUGAAUU | Translation | | 1 |
| sbi-miR1435b | SbNFY-A4 | 5 | -1 | 1 | 20 | 2834 | 2853 | UUUCUUAAGUCAAACCUUUU | CAAAGAUUUGAUAUGAUAAA | Cleavage |  | 1 |
| sbi-miR156a | SbNFY-A8 | 5 | -1 | 1 | 20 | 13760 | 13779 | UGACAGAAGAGAGUGAGCAC | CUGCUUUCUUUCUGUUGUCU | Cleavage |  | 1 |
| sbi-miR156b | SbNFY-A8 | 5 | -1 | 1 | 20 | 13760 | 13779 | UGACAGAAGAGAGUGAGCAC | CUGCUUUCUUUCUGUUGUCU | Cleavage |  | 1 |
| sbi-miR156c | SbNFY-A8 | 5 | -1 | 1 | 20 | 13760 | 13779 | UGACAGAAGAGAGUGAGCAC | CUGCUUUCUUUCUGUUGUCU | Cleavage |  | 1 |
| sbi-miR156d | SbNFY-A1 | 5 | -1 | 1 | 21 | 11831 | 11851 | UGACAGAAGAGAGAGAGCACA | UUUGCUCGUACUCUUUUGUUC | Cleavage |  | 2 |
| sbi-miR156d | SbNFY-A5 | 5 | -1 | 1 | 21 | 10658 | 10678 | UGACAGAAGAGAGAGAGCACA | CCUGCACGCCCUCUUCUGACA | Cleavage |  | 1 |
| sbi-miR156e | SbNFY-A8 | 5 | -1 | 1 | 20 | 13760 | 13779 | UGACAGAAGAGAGCGAGCAC | CUGCUUUCUUUCUGUUGUCU | Cleavage |  | 1 |
| sbi-miR156f | SbNFY-A8 | 5 | -1 | 1 | 20 | 13760 | 13779 | UGACAGAAGAGAGUGAGCAC | CUGCUUUCUUUCUGUUGUCU | Cleavage |  | 1 |
| sbi-miR156g | SbNFY-A8 | 5 | -1 | 1 | 20 | 13760 | 13779 | UGACAGAAGAGAGUGAGCAC | CUGCUUUCUUUCUGUUGUCU | Cleavage |  | 1 |
| sbi-miR156h | SbNFY-A8 | 5 | -1 | 1 | 20 | 13760 | 13779 | UGACAGAAGAGAGUGAGCAC | CUGCUUUCUUUCUGUUGUCU | Cleavage |  | 1 |
| sbi-miR156i | SbNFY-A8 | 5 | -1 | 1 | 20 | 13760 | 13779 | UGACAGAAGAGAGUGAGCAC | CUGCUUUCUUUCUGUUGUCU | Cleavage |  | 1 |
| sbi-miR159a | SbNFY-A3 | 5 | -1 | 1 | 21 | 3719 | 3739 | UUUGGAUUGAAGGGAGCUCUG | CUGGGCUCAAUUUGGUCCAAA | Cleavage |  | 1 |
| sbi-miR159b | SbNFY-A1 | 5 | -1 | 1 | 21 | 1004 | 1025 | CUUGGAUUGAAGG-GAGCUCCU | AGGGGCUUGCCUUCAAUCUGAG | Cleavage |  | 1 |
| sbi-miR160a | SbNFY-A3 | 5 | -1 | 1 | 21 | 11055 | 11075 | UGCCUGGCUCCCUGUAUGCCA | CGGCGCGUAGGGAGCCAGCCC | Cleavage |  | 3 |
| sbi-miR160a | SbNFY-A3 | 5 | -1 | 1 | 21 | 5917 | 5936 | UGCCUGGCUCCCUGUAUGCCA | UCGCA-AGAGGGGGCCAUGCA | Cleavage |  | 3 |
| sbi-miR160b | SbNFY-A3 | 5 | -1 | 1 | 21 | 11055 | 11075 | UGCCUGGCUCCCUGUAUGCCA | CGGCGCGUAGGGAGCCAGCCC | Cleavage |  | 3 |
| sbi-miR160b | SbNFY-A3 | 5 | -1 | 1 | 21 | 5917 | 5936 | UGCCUGGCUCCCUGUAUGCCA | UCGCA-AGAGGGGGCCAUGCA | Cleavage |  | 3 |
| sbi-miR160c | SbNFY-A3 | 5 | -1 | 1 | 21 | 11055 | 11075 | UGCCUGGCUCCCUGUAUGCCA | CGGCGCGUAGGGAGCCAGCCC | Cleavage |  | 3 |
| sbi-miR160c | SbNFY-A3 | 5 | -1 | 1 | 21 | 5917 | 5936 | UGCCUGGCUCCCUGUAUGCCA | UCGCA-AGAGGGGGCCAUGCA | Cleavage |  | 3 |
| sbi-miR160d | SbNFY-A3 | 5 | -1 | 1 | 21 | 11055 | 11075 | UGCCUGGCUCCCUGUAUGCCA | CGGCGCGUAGGGAGCCAGCCC | Cleavage |  | 3 |
| sbi-miR160d | SbNFY-A3 | 5 | -1 | 1 | 21 | 5917 | 5936 | UGCCUGGCUCCCUGUAUGCCA | UCGCA-AGAGGGGGCCAUGCA | Cleavage |  | 3 |
| sbi-miR160e | SbNFY-A3 | 5 | -1 | 1 | 21 | 11055 | 11075 | UGCCUGGCUCCCUGUAUGCCA | CGGCGCGUAGGGAGCCAGCCC | Cleavage |  | 3 |
| sbi-miR160e | SbNFY-A3 | 5 | -1 | 1 | 21 | 5917 | 5936 | UGCCUGGCUCCCUGUAUGCCA | UCGCA-AGAGGGGGCCAUGCA | Cleavage |  | 3 |
| sbi-miR160f | SbNFY-A3 | 5 | -1 | 1 | 21 | 15622 | 15642 | UGCCUGGCUCCCUGAAUGCCA | UGGCAGGCAGGCAGCCAGACA | Translation | | 1 |
| sbi-miR160f | SbNFY-A6 | 5 | -1 | 1 | 21 | 13676 | 13696 | UGCCUGGCUCCCUGAAUGCCA | GGCCAUACAGGCAGCCGGGCC | Translation | | 1 |
| sbi-miR164a | SbNFY-A7 | 5 | -1 | 1 | 21 | 1807 | 1827 | UGGAGAAGCAGGGCACGUGCA | UCAACGAGUCUUCCUUCUCCG | Cleavage |  | 1 |
| sbi-miR164b | SbNFY-A7 | 5 | -1 | 1 | 21 | 1807 | 1827 | UGGAGAAGCAGGGCACGUGCU | UCAACGAGUCUUCCUUCUCCG | Cleavage |  | 1 |
| sbi-miR164d | SbNFY-A7 | 5 | -1 | 1 | 21 | 1807 | 1827 | UGGAGAAGCAGGGCACGUGCA | UCAACGAGUCUUCCUUCUCCG | Cleavage |  | 1 |
| sbi-miR164e | SbNFY-A7 | 5 | -1 | 1 | 21 | 1807 | 1827 | UGGAGAAGCAGGGCACGUGCA | UCAACGAGUCUUCCUUCUCCG | Cleavage |  | 1 |
| sbi-miR168 | SbNFY-A4 | 5 | -1 | 1 | 21 | 10361 | 10382 | UCGCUUGGUGCAGAU-CGGGAC | CCCCUGCAUCUGCAUCAACUGA | Cleavage |  | 2 |
| sbi-miR169a | SbNFY-A1 | 5 | -1 | 1 | 21 | 15726 | 15745 | CAGCCAAGGAUGACUUGCCGA | CUGGUGA-UCUUCUUUGGCUG | Translation | | 2 |
| sbi-miR169a | SbNFY-A2 | 5 | -1 | 1 | 21 | 4373 | 4393 | CAGCCAAGGAUGACUUGCCGA | GGGGACAAUCAUCAUUGGUUG | Cleavage |  | 2 |
| sbi-miR169b | SbNFY-A1 | 5 | -1 | 1 | 21 | 15726 | 15745 | CAGCCAAGGAUGACUUGCCGG | CUGGUGA-UCUUCUUUGGCUG | Translation | | 2 |
| sbi-miR169b | SbNFY-A2 | 5 | -1 | 1 | 21 | 4373 | 4393 | CAGCCAAGGAUGACUUGCCGG | GGGGACAAUCAUCAUUGGUUG | Cleavage |  | 2 |
| sbi-miR169k | SbNFY-A1 | 5 | -1 | 1 | 21 | 15726 | 15745 | CAGCCAAGGAUGACUUGCCGG | CUGGUGA-UCUUCUUUGGCUG | Translation | | 2 |
| sbi-miR169k | SbNFY-A2 | 5 | -1 | 1 | 21 | 4373 | 4393 | CAGCCAAGGAUGACUUGCCGG | GGGGACAAUCAUCAUUGGUUG | Cleavage |  | 2 |
| sbi-miR169o | SbNFY-A4 | 5 | -1 | 1 | 21 | 9650 | 9670 | UAGCCAAGGAUGAUUUGCCUG | AAAGAAAAUUAUUGUUGGCUG | Cleavage |  | 3 |
| sbi-miR169o | SbNFY-A4 | 5 | -1 | 1 | 21 | 1253 | 1273 | UAGCCAAGGAUGAUUUGCCUG | UUGUUAUAUUAUCUUUUGCUA | Cleavage |  | 3 |
| sbi-miR171h | SbNFY-A6 | 5 | -1 | 1 | 21 | 12089 | 12109 | GGAUUGAGCCGCGUCAAUAUC | UCUUUUGUUGUGGCUUGAUUU | Cleavage |  | 1 |
| sbi-miR172e | SbNFY-A7 | 5 | -1 | 1 | 21 | 207 | 227 | UGAAUCUUGAUGAUGCUGCAC | CCACAGAUCCAUCGAGAUUCA | Cleavage |  | 1 |
| sbi-miR2118-5p | SbNFY-A5 | 5 | -1 | 1 | 22 | 14086 | 14107 | GGCAUGGGAACAUGUAGGAAGG | UCUUCUUCUAUAUUCUUAUGCU | Translation | | 1 |
| sbi-miR2118-5p | SbNFY-A8 | 5 | -1 | 1 | 22 | 10272 | 10293 | GGCAUGGGAACAUGUAGGAAGG | AAAACUAGGGUGUUUCCAUGCC | Cleavage |  | 1 |
| sbi-miR395a | SbNFY-A1 | 5 | -1 | 1 | 21 | 10155 | 10175 | GUGAAGUGUUUGGGGGAACUC | UUGUUCCUCCAAGAACUGCAG | Cleavage |  | 1 |
| sbi-miR395a | SbNFY-A6 | 5 | -1 | 1 | 21 | 476 | 496 | GUGAAGUGUUUGGGGGAACUC | AUUUUACCCCAAACCCAUCAC | Cleavage |  | 1 |
| sbi-miR395b | SbNFY-A1 | 5 | -1 | 1 | 21 | 10155 | 10175 | GUGAAGUGUUUGGGGGAACUC | UUGUUCCUCCAAGAACUGCAG | Cleavage |  | 1 |
| sbi-miR395b | SbNFY-A6 | 5 | -1 | 1 | 21 | 476 | 496 | GUGAAGUGUUUGGGGGAACUC | AUUUUACCCCAAACCCAUCAC | Cleavage |  | 1 |
| sbi-miR395c | SbNFY-A1 | 5 | -1 | 1 | 21 | 10155 | 10175 | GUGAAGUGUUUGGGGGAACUC | UUGUUCCUCCAAGAACUGCAG | Cleavage |  | 1 |
| sbi-miR395c | SbNFY-A6 | 5 | -1 | 1 | 21 | 476 | 496 | GUGAAGUGUUUGGGGGAACUC | AUUUUACCCCAAACCCAUCAC | Cleavage |  | 1 |
| sbi-miR395d | SbNFY-A1 | 5 | -1 | 1 | 21 | 10155 | 10175 | GUGAAGUGUUUGGGGGAACUC | UUGUUCCUCCAAGAACUGCAG | Cleavage |  | 1 |
| sbi-miR395d | SbNFY-A6 | 5 | -1 | 1 | 21 | 476 | 496 | GUGAAGUGUUUGGGGGAACUC | AUUUUACCCCAAACCCAUCAC | Cleavage |  | 1 |
| sbi-miR395e | SbNFY-A1 | 5 | -1 | 1 | 21 | 10155 | 10175 | GUGAAGUGUUUGGGGGAACUC | UUGUUCCUCCAAGAACUGCAG | Cleavage |  | 1 |
| sbi-miR395e | SbNFY-A6 | 5 | -1 | 1 | 21 | 476 | 496 | GUGAAGUGUUUGGGGGAACUC | AUUUUACCCCAAACCCAUCAC | Cleavage |  | 1 |
| sbi-miR395f | SbNFY-A7 | 5 | -1 | 1 | 21 | 16333 | 16353 | AUGAAGUGUUUGGGGGAACUC | CUUUUCCACAAAACAGUUCAU | Cleavage |  | 1 |
| sbi-miR395f | SbNFY-A1 | 5 | -1 | 1 | 21 | 16720 | 16740 | AUGAAGUGUUUGGGGGAACUC | UAGUUUGCUUAGACAUUCCAU | Cleavage |  | 2 |
| sbi-miR395f | SbNFY-A1 | 5 | -1 | 1 | 21 | 10155 | 10175 | AUGAAGUGUUUGGGGGAACUC | UUGUUCCUCCAAGAACUGCAG | Cleavage |  | 2 |
| sbi-miR395g | SbNFY-A1 | 5 | -1 | 1 | 21 | 10155 | 10175 | GUGAAGUGUUUGGGGGAACUC | UUGUUCCUCCAAGAACUGCAG | Cleavage |  | 1 |
| sbi-miR395g | SbNFY-A6 | 5 | -1 | 1 | 21 | 476 | 496 | GUGAAGUGUUUGGGGGAACUC | AUUUUACCCCAAACCCAUCAC | Cleavage |  | 1 |
| sbi-miR395h | SbNFY-A1 | 5 | -1 | 1 | 21 | 10155 | 10175 | GUGAAGUGUUUGGGGGAACUC | UUGUUCCUCCAAGAACUGCAG | Cleavage |  | 1 |
| sbi-miR395h | SbNFY-A6 | 5 | -1 | 1 | 21 | 476 | 496 | GUGAAGUGUUUGGGGGAACUC | AUUUUACCCCAAACCCAUCAC | Cleavage |  | 1 |
| sbi-miR395i | SbNFY-A1 | 5 | -1 | 1 | 21 | 10155 | 10175 | GUGAAGUGUUUGGGGGAACUC | UUGUUCCUCCAAGAACUGCAG | Cleavage |  | 1 |
| sbi-miR395i | SbNFY-A6 | 5 | -1 | 1 | 21 | 476 | 496 | GUGAAGUGUUUGGGGGAACUC | AUUUUACCCCAAACCCAUCAC | Cleavage |  | 1 |
| sbi-miR395j | SbNFY-A1 | 5 | -1 | 1 | 21 | 10155 | 10175 | GUGAAGUGUUUGGGGGAACUC | UUGUUCCUCCAAGAACUGCAG | Cleavage |  | 1 |
| sbi-miR395j | SbNFY-A6 | 5 | -1 | 1 | 21 | 476 | 496 | GUGAAGUGUUUGGGGGAACUC | AUUUUACCCCAAACCCAUCAC | Cleavage |  | 1 |
| sbi-miR395k | SbNFY-A1 | 5 | -1 | 1 | 21 | 7128 | 7148 | GUGAAGUGUUUGGAGGAACUC | AUGUUUCUCUAGAUGAUUCAG | Cleavage |  | 2 |
| sbi-miR395k | SbNFY-A5 | 5 | -1 | 1 | 21 | 5190 | 5210 | GUGAAGUGUUUGGAGGAACUC | UUAUUCCUUCACAUCCUUCAC | Translation | | 1 |
| sbi-miR396e | SbNFY-A2 | 5 | -1 | 1 | 22 | 5170 | 5191 | UUCCACAGGCUUUCUUGAACUG | GUCAUCAAGGAACUAUGUGGAA | Translation | | 1 |
| sbi-miR396e | SbNFY-A6 | 5 | -1 | 1 | 22 | 7552 | 7573 | UUCCACAGGCUUUCUUGAACUG | GUAUUUGUUAAAGGUUGUGGAA | Cleavage |  | 1 |
| sbi-miR397-5p | SbNFY-A1 | 5 | -1 | 1 | 21 | 2651 | 2671 | UCAUUGAGUGCAGCGUUGAUG | CUUCAAAGGUGCACUCAAAGC | Cleavage |  | 1 |
| sbi-miR399a | SbNFY-A7 | 5 | -1 | 1 | 21 | 8690 | 8709 | UGCCAAAGGAGAAUUGCCCUG | CAUGGCU-UUUUCUUUUGGCA | Cleavage |  | 1 |
| sbi-miR399a | SbNFY-A4 | 5 | -1 | 1 | 21 | 1428 | 1448 | UGCCAAAGGAGAAUUGCCCUG | CCGGGCCUUUUUUUUUUGGUU | Cleavage |  | 1 |
| sbi-miR399b | SbNFY-A4 | 5 | -1 | 1 | 21 | 15596 | 15616 | UGCCAAAGGAGAGCUGCCCUG | CCGGCCGGCUCUUCGUCGGCA | Cleavage |  | 1 |
| sbi-miR399c | SbNFY-A7 | 5 | -1 | 1 | 21 | 8690 | 8709 | UGCCAAAGGAGAAUUGCCCUG | CAUGGCU-UUUUCUUUUGGCA | Cleavage |  | 1 |
| sbi-miR399c | SbNFY-A4 | 5 | -1 | 1 | 21 | 1428 | 1448 | UGCCAAAGGAGAAUUGCCCUG | CCGGGCCUUUUUUUUUUGGUU | Cleavage |  | 1 |
| sbi-miR399e | SbNFY-A4 | 5 | -1 | 1 | 21 | 12605 | 12625 | UGCCAAAGGAGAUUUGCCCAG | AGCAAUAAAUUUUUUUUGGCA | Cleavage |  | 1 |
| sbi-miR399f | SbNFY-A4 | 5 | -1 | 1 | 21 | 12605 | 12625 | UGCCAAAGGAGAUUUGCCCAG | AGCAAUAAAUUUUUUUUGGCA | Cleavage |  | 1 |
| sbi-miR399h | SbNFY-A7 | 5 | -1 | 1 | 21 | 8690 | 8709 | UGCCAAAGGAGAAUUGCCCUG | CAUGGCU-UUUUCUUUUGGCA | Cleavage |  | 1 |
| sbi-miR399h | SbNFY-A4 | 5 | -1 | 1 | 21 | 1428 | 1448 | UGCCAAAGGAGAAUUGCCCUG | CCGGGCCUUUUUUUUUUGGUU | Cleavage |  | 1 |
| sbi-miR399j | SbNFY-A7 | 5 | -1 | 1 | 21 | 8690 | 8709 | UGCCAAAGGAGAAUUGCCCUG | CAUGGCU-UUUUCUUUUGGCA | Cleavage |  | 1 |
| sbi-miR399j | SbNFY-A4 | 5 | -1 | 1 | 21 | 1428 | 1448 | UGCCAAAGGAGAAUUGCCCUG | CCGGGCCUUUUUUUUUUGGUU | Cleavage |  | 1 |
| sbi-miR399k | SbNFY-A1 | 5 | -1 | 1 | 21 | 16400 | 16420 | UGCCAAAGGGGAUUUGCCCGG | GCCCCCAAAUCCACUUUGGCG | Cleavage |  | 1 |
| sbi-miR437a | SbNFY-A4 | 5 | -1 | 1 | 21 | 12511 | 12531 | AAAGUUAGAGAAGUUUGACUU | AAUUUAAACUUCUCAGGCUUA | Cleavage |  | 1 |
| sbi-miR437a | SbNFY-A1 | 5 | -1 | 1 | 21 | 2790 | 2810 | AAAGUUAGAGAAGUUUGACUU | UUGUACAAUUUUUUUGGUUUU | Cleavage |  | 1 |
| sbi-miR437b | SbNFY-A4 | 5 | -1 | 1 | 21 | 12511 | 12531 | AAAGUUAGAGAAGUUUGACUU | AAUUUAAACUUCUCAGGCUUA | Cleavage |  | 1 |
| sbi-miR437b | SbNFY-A1 | 5 | -1 | 1 | 21 | 2790 | 2810 | AAAGUUAGAGAAGUUUGACUU | UUGUACAAUUUUUUUGGUUUU | Cleavage |  | 1 |
| sbi-miR437c | SbNFY-A4 | 5 | -1 | 1 | 21 | 12511 | 12531 | AAAGUUAGAGAAGUUUGACUU | AAUUUAAACUUCUCAGGCUUA | Cleavage |  | 1 |
| sbi-miR437c | SbNFY-A1 | 5 | -1 | 1 | 21 | 2790 | 2810 | AAAGUUAGAGAAGUUUGACUU | UUGUACAAUUUUUUUGGUUUU | Cleavage |  | 1 |
| sbi-miR437d | SbNFY-A4 | 5 | -1 | 1 | 21 | 12511 | 12531 | AAAGUUAGAGAAGUUUGACUU | AAUUUAAACUUCUCAGGCUUA | Cleavage |  | 1 |
| sbi-miR437d | SbNFY-A1 | 5 | -1 | 1 | 21 | 2790 | 2810 | AAAGUUAGAGAAGUUUGACUU | UUGUACAAUUUUUUUGGUUUU | Cleavage |  | 1 |
| sbi-miR437e | SbNFY-A4 | 5 | -1 | 1 | 21 | 12511 | 12531 | AAAGUUAGAGAAGUUUGACUU | AAUUUAAACUUCUCAGGCUUA | Cleavage |  | 1 |
| sbi-miR437e | SbNFY-A1 | 5 | -1 | 1 | 21 | 2790 | 2810 | AAAGUUAGAGAAGUUUGACUU | UUGUACAAUUUUUUUGGUUUU | Cleavage |  | 1 |
| sbi-miR437f | SbNFY-A4 | 5 | -1 | 1 | 21 | 12511 | 12531 | AAAGUUAGAGAAGUUUGACUU | AAUUUAAACUUCUCAGGCUUA | Cleavage |  | 1 |
| sbi-miR437f | SbNFY-A1 | 5 | -1 | 1 | 21 | 2790 | 2810 | AAAGUUAGAGAAGUUUGACUU | UUGUACAAUUUUUUUGGUUUU | Cleavage |  | 1 |
| sbi-miR437g | SbNFY-A4 | 5 | -1 | 1 | 21 | 12511 | 12531 | AAAGUUAGAGAAGUUUGACUU | AAUUUAAACUUCUCAGGCUUA | Cleavage |  | 1 |
| sbi-miR437g | SbNFY-A1 | 5 | -1 | 1 | 21 | 2790 | 2810 | AAAGUUAGAGAAGUUUGACUU | UUGUACAAUUUUUUUGGUUUU | Cleavage |  | 1 |
| sbi-miR437i | SbNFY-A4 | 5 | -1 | 1 | 21 | 12511 | 12531 | AAAGUUAGAGAAGUUUGACUU | AAUUUAAACUUCUCAGGCUUA | Cleavage |  | 1 |
| sbi-miR437i | SbNFY-A1 | 5 | -1 | 1 | 21 | 2790 | 2810 | AAAGUUAGAGAAGUUUGACUU | UUGUACAAUUUUUUUGGUUUU | Cleavage |  | 1 |
| sbi-miR437j | SbNFY-A4 | 5 | -1 | 1 | 21 | 12511 | 12531 | AAAGUUAGAGAAGUUUGACUU | AAUUUAAACUUCUCAGGCUUA | Cleavage |  | 1 |
| sbi-miR437j | SbNFY-A1 | 5 | -1 | 1 | 21 | 2790 | 2810 | AAAGUUAGAGAAGUUUGACUU | UUGUACAAUUUUUUUGGUUUU | Cleavage |  | 1 |
| sbi-miR437k | SbNFY-A4 | 5 | -1 | 1 | 21 | 12511 | 12531 | AAAGUUAGAGAAGUUUGACUU | AAUUUAAACUUCUCAGGCUUA | Cleavage |  | 1 |
| sbi-miR437k | SbNFY-A1 | 5 | -1 | 1 | 21 | 2790 | 2810 | AAAGUUAGAGAAGUUUGACUU | UUGUACAAUUUUUUUGGUUUU | Cleavage |  | 1 |
| sbi-miR437l | SbNFY-A4 | 5 | -1 | 1 | 21 | 12511 | 12531 | AAAGUUAGAGAAGUUUGACUU | AAUUUAAACUUCUCAGGCUUA | Cleavage |  | 1 |
| sbi-miR437l | SbNFY-A1 | 5 | -1 | 1 | 21 | 2790 | 2810 | AAAGUUAGAGAAGUUUGACUU | UUGUACAAUUUUUUUGGUUUU | Cleavage |  | 1 |
| sbi-miR437m | SbNFY-A4 | 5 | -1 | 1 | 21 | 12511 | 12531 | AAAGUUAGAGAAGUUUGACUU | AAUUUAAACUUCUCAGGCUUA | Cleavage |  | 1 |
| sbi-miR437m | SbNFY-A1 | 5 | -1 | 1 | 21 | 2790 | 2810 | AAAGUUAGAGAAGUUUGACUU | UUGUACAAUUUUUUUGGUUUU | Cleavage |  | 1 |
| sbi-miR437n | SbNFY-A4 | 5 | -1 | 1 | 21 | 12511 | 12531 | AAAGUUAGAGAAGUUUGACUU | AAUUUAAACUUCUCAGGCUUA | Cleavage |  | 1 |
| sbi-miR437n | SbNFY-A1 | 5 | -1 | 1 | 21 | 2790 | 2810 | AAAGUUAGAGAAGUUUGACUU | UUGUACAAUUUUUUUGGUUUU | Cleavage |  | 1 |
| sbi-miR437o | SbNFY-A4 | 5 | -1 | 1 | 21 | 12511 | 12531 | AAAGUUAGAGAAGUUUGACUU | AAUUUAAACUUCUCAGGCUUA | Cleavage |  | 1 |
| sbi-miR437o | SbNFY-A1 | 5 | -1 | 1 | 21 | 2790 | 2810 | AAAGUUAGAGAAGUUUGACUU | UUGUACAAUUUUUUUGGUUUU | Cleavage |  | 1 |
| sbi-miR437p | SbNFY-A4 | 5 | -1 | 1 | 21 | 12511 | 12531 | AAAGUUAGAGAAGUUUGACUU | AAUUUAAACUUCUCAGGCUUA | Cleavage |  | 1 |
| sbi-miR437p | SbNFY-A1 | 5 | -1 | 1 | 21 | 2790 | 2810 | AAAGUUAGAGAAGUUUGACUU | UUGUACAAUUUUUUUGGUUUU | Cleavage |  | 1 |
| sbi-miR437q | SbNFY-A4 | 5 | -1 | 1 | 21 | 12511 | 12531 | AAAGUUAGAGAAGUUUGACUU | AAUUUAAACUUCUCAGGCUUA | Cleavage |  | 1 |
| sbi-miR437q | SbNFY-A1 | 5 | -1 | 1 | 21 | 2790 | 2810 | AAAGUUAGAGAAGUUUGACUU | UUGUACAAUUUUUUUGGUUUU | Cleavage |  | 1 |
| sbi-miR437r | SbNFY-A4 | 5 | -1 | 1 | 21 | 12511 | 12531 | AAAGUUAGAGAAGUUUGACUU | AAUUUAAACUUCUCAGGCUUA | Cleavage |  | 1 |
| sbi-miR437r | SbNFY-A1 | 5 | -1 | 1 | 21 | 2790 | 2810 | AAAGUUAGAGAAGUUUGACUU | UUGUACAAUUUUUUUGGUUUU | Cleavage |  | 1 |
| sbi-miR437s | SbNFY-A4 | 5 | -1 | 1 | 21 | 12511 | 12531 | AAAGUUAGAGAAGUUUGACUU | AAUUUAAACUUCUCAGGCUUA | Cleavage |  | 1 |
| sbi-miR437s | SbNFY-A1 | 5 | -1 | 1 | 21 | 2790 | 2810 | AAAGUUAGAGAAGUUUGACUU | UUGUACAAUUUUUUUGGUUUU | Cleavage |  | 1 |
| sbi-miR437t | SbNFY-A4 | 5 | -1 | 1 | 21 | 12511 | 12531 | AAAGUUAGAGAAGUUUGACUU | AAUUUAAACUUCUCAGGCUUA | Cleavage |  | 1 |
| sbi-miR437t | SbNFY-A1 | 5 | -1 | 1 | 21 | 2790 | 2810 | AAAGUUAGAGAAGUUUGACUU | UUGUACAAUUUUUUUGGUUUU | Cleavage |  | 1 |
| sbi-miR437u | SbNFY-A4 | 5 | -1 | 1 | 21 | 12511 | 12531 | AAAGUUAGAGAAGUUUGACUU | AAUUUAAACUUCUCAGGCUUA | Cleavage |  | 1 |
| sbi-miR437u | SbNFY-A1 | 5 | -1 | 1 | 21 | 2790 | 2810 | AAAGUUAGAGAAGUUUGACUU | UUGUACAAUUUUUUUGGUUUU | Cleavage |  | 1 |
| sbi-miR437v | SbNFY-A4 | 5 | -1 | 1 | 21 | 12511 | 12531 | AAAGUUAGAGAAGUUUGACUU | AAUUUAAACUUCUCAGGCUUA | Cleavage |  | 1 |
| sbi-miR437v | SbNFY-A1 | 5 | -1 | 1 | 21 | 2790 | 2810 | AAAGUUAGAGAAGUUUGACUU | UUGUACAAUUUUUUUGGUUUU | Cleavage |  | 1 |
| sbi-miR437w | SbNFY-A4 | 5 | -1 | 1 | 21 | 12511 | 12531 | AAAGUUAGAGAAGUUUGACUU | AAUUUAAACUUCUCAGGCUUA | Cleavage |  | 1 |
| sbi-miR437w | SbNFY-A1 | 5 | -1 | 1 | 21 | 2790 | 2810 | AAAGUUAGAGAAGUUUGACUU | UUGUACAAUUUUUUUGGUUUU | Cleavage |  | 1 |
| sbi-miR437x-3p | SbNFY-A6 | 5 | -1 | 1 | 24 | 16295 | 16318 | AUUUGACUGACACGGAUUCUAGGA | UUAUGGAAUGCGGGUGGGUUAAAU | Cleavage |  | 1 |
| sbi-miR437x-5p | SbNFY-A2 | 5 | -1 | 1 | 24 | 3635 | 3658 | UAGAGUUGUCCUAAGUCAAACUUU | ACCUUUUGACAUUGGACACUUUUA | Cleavage |  | 1 |
| sbi-miR437x-5p | SbNFY-A7 | 5 | -1 | 1 | 24 | 8086 | 8109 | UAGAGUUGUCCUAAGUCAAACUUU | AAGAUAUGAGUUGGGAUGAAUCUA | Cleavage |  | 1 |
| sbi-miR437x-5p | SbNFY-A1 | 5 | -1 | 1 | 24 | 16465 | 16488 | UAGAGUUGUCCUAAGUCAAACUUU | UUAUUUUAAGUUAGUAUAACUUUG | Translation | | 2 |
| sbi-miR528 | SbNFY-A2 | 5 | -1 | 1 | 21 | 8870 | 8890 | UGGAAGGGGCAUGCAGAGGAG | UGGCUUUACAUGCCUUUACCA | Cleavage |  | 2 |
| sbi-miR528 | SbNFY-A5 | 5 | -1 | 1 | 21 | 10946 | 10966 | UGGAAGGGGCAUGCAGAGGAG | GACAUAUGCAAGUCCUUUUCA | Translation | | 2 |
| sbi-miR5386 | SbNFY-A4 | 5 | -1 | 1 | 20 | 9117 | 9136 | CGUCGCUGUCGCGCGCGCUG | CGGCGGCGGCGGCGGCGACG | Cleavage |  | 1 |
| sbi-miR5386 | SbNFY-A5 | 5 | -1 | 1 | 20 | 13459 | 13478 | CGUCGCUGUCGCGCGCGCUG | AGGCGAACGCGGCGGCGGCA | Cleavage |  | 1 |
| sbi-miR5386 | SbNFY-A1 | 5 | -1 | 1 | 20 | 15689 | 15708 | CGUCGCUGUCGCGCGCGCUG | CGCCGGCCGCGGCGGCGGCG | Cleavage |  | 1 |
| sbi-miR5387b | SbNFY-A8 | 5 | -1 | 1 | 24 | 2483 | 2506 | CGUGGCUCUGACCGGUGCUAAAGG | GGCUCAGCUCCGGCCGGGGUCACC | Translation | | 1 |
| sbi-miR5564a | SbNFY-A6 | 5 | -1 | 1 | 22 | 6945 | 6966 | UGGGGAAGCAAUUCGUCGAACA | GAAUCUAUAAAUUGUUUUCUCC | Cleavage |  | 1 |
| sbi-miR5564b | SbNFY-A4 | 5 | -1 | 1 | 21 | 3332 | 3352 | GCAAUUCGUCGAACAGCUUGA | GAGAGCUGUUUGGGUAGUUGC | Cleavage |  | 1 |
| sbi-miR5565c | SbNFY-A2 | 5 | -1 | 1 | 21 | 9375 | 9395 | UACACAUGUGGAUUGAGGUGA | UCAAAUCAAUUUACACGUGUG | Cleavage |  | 2 |
| sbi-miR5565c | SbNFY-A2 | 5 | -1 | 1 | 21 | 2739 | 2759 | UACACAUGUGGAUUGAGGUGA | GCACCAUAGUUCGUAUUUGUA | Cleavage |  | 2 |
| sbi-miR5565e | SbNFY-A1 | 5 | -1 | 1 | 19 | 13891 | 13909 | UUGUUUGGAUGUUGUCGGA | UCUUGCAGUUUCCAAACGA | Translation | | 2 |
| sbi-miR5565e | SbNFY-A5 | 5 | -1 | 1 | 19 | 11892 | 11910 | UUGUUUGGAUGUUGUCGGA | UUUAGUAACAACCGAACAA | Cleavage |  | 3 |
| sbi-miR5565e | SbNFY-A8 | 5 | -1 | 1 | 19 | 11488 | 11506 | UUGUUUGGAUGUUGUCGGA | ACCUACAACAUACAAACGU | Cleavage |  | 1 |
| sbi-miR5565e | SbNFY-A7 | 5 | -1 | 1 | 19 | 15614 | 15632 | UUGUUUGGAUGUUGUCGGA | UGCCACAAGAUCUGAAUAA | Translation | | 1 |
| sbi-miR5565e | SbNFY-A2 | 5 | -1 | 1 | 19 | 6930 | 6948 | UUGUUUGGAUGUUGUCGGA | UCCCAAACUAUUCAAGCAA | Cleavage |  | 1 |
| sbi-miR5565f | SbNFY-A6 | 5 | -1 | 1 | 20 | 16312 | 16331 | UAGUCGGAUUUAUAUCAAUC | GUUAAAUAUAAAUUUGACUA | Cleavage |  | 2 |
| sbi-miR5565g-3p | SbNFY-A6 | 5 | -1 | 1 | 24 | 8101 | 8124 | ACACAUGUGGAUUGAGAUGAAUAC | CGUUUUUUCUCUUUCCAUAUGUGU | Cleavage |  | 1 |
| sbi-miR5565g-5p | SbNFY-A4 | 5 | -1 | 1 | 24 | 1133 | 1156 | UUCACAUCAAUCCACAUAUGUUGG | UAUAUAUCUUUUGAUUGAUGAGAA | Cleavage |  | 1 |
| sbi-miR5566 | SbNFY-A4 | 5 | -1 | 1 | 21 | 65 | 85 | UCAGCAUCACCUCCCUGUUGU | AUCUCGGCGAGGUGAUGAUGA | Cleavage |  | 1 |
| sbi-miR5566 | SbNFY-A3 | 5 | -1 | 1 | 21 | 11237 | 11257 | UCAGCAUCACCUCCCUGUUGU | AGAGAGUGGAGGUGAUGCGGG | Cleavage |  | 1 |
| sbi-miR5568a | SbNFY-A5 | 5 | -1 | 1 | 21 | 1993 | 2013 | CAGAGCGACUUACAAUUUGGA | UCAAAAUUGUAACUCCUUUUG | Cleavage |  | 1 |
| sbi-miR5568a | SbNFY-A6 | 5 | -1 | 1 | 21 | 12009 | 12029 | CAGAGCGACUUACAAUUUGGA | CUCAAAUUAUAAGUCGUUUGA | Cleavage |  | 2 |
| sbi-miR5568a | SbNFY-A6 | 5 | -1 | 1 | 21 | 3892 | 3912 | CAGAGCGACUUACAAUUUGGA | AAUAAAAUGUAAGUUGCACGG | Cleavage |  | 2 |
| sbi-miR5568a | SbNFY-A2 | 5 | -1 | 1 | 21 | 11400 | 11420 | CAGAGCGACUUACAAUUUGGA | UUCUAAUUAUAAGACGUUUUG | Cleavage |  | 1 |
| sbi-miR5568b-3p | SbNFY-A2 | 5 | -1 | 1 | 21 | 6342 | 6362 | ACUAUGUAUCUAGAAAAGCUA | UUCCUUUUGUAGGUUUAUAGU | Cleavage |  | 2 |
| sbi-miR5568b-5p | SbNFY-A5 | 5 | -1 | 1 | 21 | 3943 | 3963 | UUUCUAGGUACAUAGCUUUUG | CAAAAUCUAUGUUUUUAUAAA | Cleavage |  | 1 |
| sbi-miR5568b-5p | SbNFY-A2 | 5 | -1 | 1 | 21 | 11466 | 11486 | UUUCUAGGUACAUAGCUUUUG | AUAAAAAAAUGUAUCUAAAAA | Cleavage |  | 1 |
| sbi-miR5568c-5p | SbNFY-A3 | 5 | -1 | 1 | 21 | 15320 | 15340 | UCUGUUCCAAAUUGUAAGUCG | CGAGUGACAAGUUGGGAUGGA | Translation | | 2 |
| sbi-miR5568d-3p | SbNFY-A2 | 5 | -1 | 1 | 21 | 15941 | 15961 | AAAGUUGUGUAUCUAGAAAAG | ACUUUCUAGAUAUACUUUUUA | Cleavage |  | 2 |
| sbi-miR5568d-5p | SbNFY-A7 | 5 | -1 | 1 | 21 | 12462 | 12482 | UGGCUUUUCUAGAUACAUAGC | AUUUUGUGUCUGGCAGGGUCA | Cleavage |  | 1 |
| sbi-miR5568e-5p | SbNFY-A4 | 5 | -1 | 1 | 21 | 17582 | 17602 | GAUGUUUUGGGUUUUCUAGAU | GUAUAGCAGACCAAAAAUAUU | Cleavage |  | 1 |
| sbi-miR5568f-3p | SbNFY-A4 | 5 | -1 | 1 | 21 | 12648 | 12668 | GUCUUAUAAUUUGGAAUGGAG | UUUCAAUGCAAGAUGUAAGAC | Cleavage |  | 1 |
| sbi-miR5568f-3p | SbNFY-A2 | 5 | -1 | 1 | 21 | 11395 | 11414 | GUCUUAUAAUUUGGAAUGGAG | UAGUAUUCUAA-UUAUAAGAC | Translation | | 2 |
| sbi-miR5568f-3p | SbNFY-A2 | 5 | -1 | 1 | 21 | 10441 | 10461 | GUCUUAUAAUUUGGAAUGGAG | UCCCUUUCCGAUUUAUUAGAU | Translation | | 2 |
| sbi-miR5568f-5p | SbNFY-A3 | 5 | -1 | 1 | 21 | 15320 | 15340 | UCCAUUCCAAAUUGUAAGAUG | CGAGUGACAAGUUGGGAUGGA | Translation | | 2 |
| sbi-miR5568g-3p | SbNFY-A2 | 5 | -1 | 1 | 21 | 8996 | 9016 | AAAACGUCUUAUAAUUUGGAG | UUCUAAAAUAUAGGUCAUUUU | Cleavage |  | 2 |
| sbi-miR5568g-3p | SbNFY-A8 | 5 | -1 | 1 | 21 | 9144 | 9164 | AAAACGUCUUAUAAUUUGGAG | UUUCGCAUUGCGAGGCGUUUU | Translation | | 1 |
| sbi-miR5568g-5p | SbNFY-A8 | 5 | -1 | 1 | 21 | 13454 | 13474 | CAAAUUAUAAGAUGUUUUGGC | GACAAAGCAGUUUAUGGCUUG | Cleavage |  | 1 |
| sbi-miR5569 | SbNFY-A3 | 5 | -1 | 1 | 24 | 16877 | 16900 | UAUUGCAUGCUUGAACUAUGGUAA | CAAACAUUGUUCAAUUAUGGAGUA | Translation | | 1 |
| sbi-miR6217a-3p | SbNFY-A4 | 5 | -1 | 1 | 24 | 2363 | 2387 | AAAAUUAUCGUAAA-UAGAGGUGGC | UUUACUUAUAAUUUACGAUAAUCUU | Cleavage |  | 1 |
| sbi-miR6217b-3p | SbNFY-A4 | 5 | -1 | 1 | 24 | 2363 | 2387 | AAAAUUAUCGUAAA-UAGAGGUGGC | UUUACUUAUAAUUUACGAUAAUCUU | Cleavage |  | 1 |
| sbi-miR6218-5p | SbNFY-A1 | 5 | -1 | 1 | 21 | 2792 | 2812 | CGAAAAUCACGAAACUUGUCG | GUACAAUUUUUUUGGUUUUAG | Translation | | 1 |
| sbi-miR6219-5p | SbNFY-A2 | 5 | -1 | 1 | 24 | 5227 | 5250 | GAACCGGGACUAAAGGUGGGACAU | CUUUUCCAUCUUUAUUAUUGGUUU | Translation | | 1 |
| sbi-miR6220-3p | SbNFY-A2 | 5 | -1 | 1 | 24 | 13727 | 13750 | AUGCCUUAUAAUUUGGGAUGGAGA | UAAUGAUCACAAGUUGUGAUGCAA | Cleavage |  | 1 |
| sbi-miR6222-5p | SbNFY-A8 | 5 | -1 | 1 | 21 | 8333 | 8353 | CCUGUUUGGAUCAGCCAAGGC | UUUUUGGGUGAACUAAACAAG | Translation | | 1 |
| sbi-miR6223-3p | SbNFY-A6 | 5 | -1 | 1 | 21 | 3765 | 3785 | CUAGCAUGUUCCUCCUAAGAG | CGCUGGGGAGAAUUAUGCUAG | Translation | | 1 |
| sbi-miR6223-5p | SbNFY-A7 | 5 | -1 | 1 | 21 | 14660 | 14680 | UUCUUGGGAGGAGCAUGCUAG | UUUACAUGCUUUUCUCGAGGG | Cleavage |  | 1 |
| sbi-miR6225-3p | SbNFY-A1 | 5 | -1 | 1 | 24 | 13856 | 13879 | GAAACGAAUCUUUUAAGUCUAAUU | AAUUAUACUUAAUAGAUUUGUCUU | Cleavage |  | 4 |
| sbi-miR6225-3p | SbNFY-A1 | 5 | -1 | 1 | 24 | 3247 | 3269 | GAAACGAAUCUUUUAAGUCUAAUU | AACUAGGAU-AAAAGAUUCGUCUC | Cleavage |  | 4 |
| sbi-miR6225-3p | SbNFY-A8 | 5 | -1 | 1 | 24 | 8204 | 8227 | GAAACGAAUCUUUUAAGUCUAAUU | AACUAGGCUCAAAAGAUUUAUAUC | Cleavage |  | 2 |
| sbi-miR6225-3p | SbNFY-A2 | 5 | -1 | 1 | 24 | 1260 | 1283 | GAAACGAAUCUUUUAAGUCUAAUU | AAACAGAAUUGGGAUAUUUGUUUU | Translation | | 2 |
| sbi-miR6225-3p | SbNFY-A2 | 5 | -1 | 1 | 24 | 8557 | 8580 | GAAACGAAUCUUUUAAGUCUAAUU | GAUCUGACCUAAAAUAUUUAUUUU | Translation | | 2 |
| sbi-miR6227-3p | SbNFY-A8 | 5 | -1 | 1 | 22 | 320 | 341 | CUCACAACACUUGCUAUUUGGG | UACAAAUGGCAACUGUUUUGGU | Translation | | 2 |
| sbi-miR6227-3p | SbNFY-A8 | 5 | -1 | 1 | 22 | 3861 | 3881 | CUCACAACACUUGCUAUUUGGG | ACUGAA-AGCAGGUGUUGUAGG | Cleavage |  | 2 |
| sbi-miR6228-3p | SbNFY-A1 | 5 | -1 | 1 | 24 | 8373 | 8396 | GUGGCAGUAGAAUUAAUGAAGGGA | CUGUGUAAUUAAUUUUAUUUUUAU | Cleavage |  | 2 |
| sbi-miR6228-3p | SbNFY-A1 | 5 | -1 | 1 | 24 | 88 | 111 | GUGGCAGUAGAAUUAAUGAAGGGA | AGAAGUUCUGAAUCUUACUGCUAC | Translation | | 2 |
| sbi-miR6228-5p | SbNFY-A6 | 5 | -1 | 1 | 24 | 15658 | 15681 | UUCUAUCUCUAUUAAUUGUGUUGC | AUGAAACAUUAAAUAUAGAUAAAA | Cleavage |  | 1 |
| sbi-miR6229-3p | SbNFY-A5 | 5 | -1 | 1 | 24 | 12019 | 12042 | GUUUUUCUCGCCGGGUGAGAAGGC | AAGAUUUUACCUUGUGAGAAGAAG | Cleavage |  | 1 |
| sbi-miR6229-5p | SbNFY-A4 | 5 | -1 | 1 | 24 | 9303 | 9326 | AUUCUCACUUGGGCGACGGAAAGG | CCUCGCCGUCGCCCUGGUGGUGGU | Translation | | 1 |
| sbi-miR6230-3p | SbNFY-A8 | 5 | -1 | 1 | 21 | 8125 | 8145 | UAACAAGUUUAGGGAUCUAGA | UUUAGUUUCCAAAACUUUUUG | Translation | | 1 |
| sbi-miR6230-5p | SbNFY-A2 | 5 | -1 | 1 | 21 | 15469 | 15489 | UUUUGGGUCCCUAAACUUGUU | CGCAAAAUUGGAGGCUCAAAA | Translation | | 1 |
| sbi-miR6232a-3p | SbNFY-A6 | 5 | -1 | 1 | 24 | 4568 | 4591 | UGGAUGUACCAAAAAAGUCAAAGC | AUUAGGACGUGUUUGGUGUAGCUA | Cleavage |  | 1 |
| sbi-miR6232b-5p | SbNFY-A3 | 5 | -1 | 1 | 21 | 5341 | 5361 | UUUUUGGUACAUUGAAUUUGC | CAAGAUUCGAUGUGACGGGGA | Cleavage |  | 3 |
| sbi-miR6232b-5p | SbNFY-A2 | 5 | -1 | 1 | 21 | 11629 | 11649 | UUUUUGGUACAUUGAAUUUGC | UGAAAUGCAAAAUACCAAAAU | Translation | | 1 |
| sbi-miR6233-3p | SbNFY-A6 | 5 | -1 | 1 | 24 | 12188 | 12211 | CAAGUUUGGUUUUGGUAAUUAAUG | UUGAGGUUAAAAAAGUUAAACUUC | Cleavage |  | 1 |
| sbi-miR6233-5p | SbNFY-A8 | 5 | -1 | 1 | 24 | 12049 | 12072 | UGUUGAGGCUGGAGCGAAACUCGG | UACAGCUUCAUUCUAGCCAUAACA | Cleavage |  | 1 |
| sbi-miR6235-3p | SbNFY-A2 | 5 | -1 | 1 | 24 | 690 | 713 | AACGAACAGUAUUUUUCUCUUACA | UAUCUUAGAGAAAUAUUUUUCAUU | Cleavage |  | 1 |
| sbi-miR6235-5p | SbNFY-A1 | 5 | -1 | 1 | 24 | 8155 | 8178 | UUGUGAGAGAAAAAUACUGUUGGC | AGAGGCAGUAUCUUUAUCUUGCAC | Cleavage |  | 3 |
| sbi-miR6235-5p | SbNFY-A1 | 5 | -1 | 1 | 24 | 13362 | 13385 | UUGUGAGAGAAAAAUACUGUUGGC | AAGUUUAGGGGUUUUUUUUUGCAA | Cleavage |  | 3 |
| sbi-miR6235-5p | SbNFY-A2 | 5 | -1 | 1 | 24 | 1307 | 1330 | UUGUGAGAGAAAAAUACUGUUGGC | UUUAUUAGUAGUUUUUUUUUAGGA | Cleavage |  | 1 |
| sbi-miR6235-5p | SbNFY-A7 | 5 | -1 | 1 | 24 | 15381 | 15404 | UUGUGAGAGAAAAAUACUGUUGGC | AAAUUCAGAAAAUUUCUAUCACAA | Cleavage |  | 1 |
| sbi-miR821a | SbNFY-A8 | 5 | -1 | 1 | 21 | 9715 | 9735 | AAGUCAUCAACAUAAAAGUUG | UUGUUUUUAUGUUGGAAAUUU | Cleavage |  | 1 |
| sbi-miR821a | SbNFY-A7 | 5 | -1 | 1 | 21 | 14310 | 14330 | AAGUCAUCAACAUAAAAGUUG | CUAUGUGUAUGUUGAUGACAA | Cleavage |  | 1 |
| sbi-miR821b | SbNFY-A6 | 5 | -1 | 1 | 21 | 6599 | 6619 | AAGUUAUGAACAUAAAAGUUG | AUACUUCUAUGUUAAUGAUAU | Cleavage |  | 1 |
| sbi-miR821b | SbNFY-A2 | 5 | -1 | 1 | 21 | 8108 | 8128 | AAGUUAUGAACAUAAAAGUUG | CAACAUAGAUGUUCAUAAUAU | Cleavage |  | 1 |
| sbi-miR821b | SbNFY-A8 | 5 | -1 | 1 | 21 | 9715 | 9735 | AAGUUAUGAACAUAAAAGUUG | UUGUUUUUAUGUUGGAAAUUU | Cleavage |  | 1 |
| sbi-miR821c | SbNFY-A8 | 5 | -1 | 1 | 21 | 9715 | 9735 | AAGUCAUCAACAUAAAAGUUG | UUGUUUUUAUGUUGGAAAUUU | Cleavage |  | 1 |
| sbi-miR821c | SbNFY-A7 | 5 | -1 | 1 | 21 | 14310 | 14330 | AAGUCAUCAACAUAAAAGUUG | CUAUGUGUAUGUUGAUGACAA | Cleavage |  | 1 |
