# Supplementary material for: Genome-wide identification and expression profile analysis of nuclear factor Y family genes in Sorghum bicolor L. (Moench)
Source: PLoS One. 2019 Sep 19;14(9):e0222203. doi: 10.1371/journal.pone.0222203 (PMC6752760; doi:10.1371/journal.pone.0222203)
Supplement: S12 Table — (DOC) [file pone.0222203.s020.doc]

**S12 Table.** In silico analysis of miRNAs for SbNFY-B

| miRNA_Acc. | Target_Acc. | Expectation | UPE$ | miRNA_start | miRNA_end | Target_start | Target_end | miRNA_aligned_fragment | Target_aligned_fragment | Inhibition | Target_Desc. | Multiplicity |
| --- | --- | --- | --- | --- | --- | --- | --- | --- | --- | --- | --- | --- |
| sbi-miR5565e | SbNFY-B14 | 0 | -1 | 1 | 19 | 17492 | 17510 | UUGUUUGGAUGUUGUCGGA | UCCGACAACAUCCAAACAA | Cleavage |  | 2 |
| sbi-miR5568g-3p | SbNFY-B6 | 0 | -1 | 1 | 21 | 3190 | 3210 | AAAACGUCUUAUAAUUUGGAG | UCCCAAAUUAUAAGACGUUUU | Cleavage |  | 2 |
| sbi-miR6232b-3p | SbNFY-B12 | 0 | -1 | 1 | 21 | 14091 | 14111 | AAUUCGAUGUACCAAAAAAGU | ACUUUUUUGGUACAUCGAAUU | Cleavage |  | 1 |
| sbi-miR6220-5p | SbNFY-B4 | 0.5 | -1 | 1 | 24 | 11810 | 11833 | CUCCAUCCUAAAUUAUAAGACAUU | AAUGACUUAUAAUUUGGGAUGGAG | Cleavage |  | 2 |
| sbi-miR6235-5p | SbNFY-B10 | 0.5 | -1 | 1 | 24 | 16616 | 16639 | UUGUGAGAGAAAAAUACUGUUGGC | UACCGCAGUAUUUUUCUCUCAUAA | Cleavage |  | 1 |
| sbi-miR5567 | SbNFY-B7 | 1 | -1 | 1 | 24 | 12768 | 12791 | UUAAUGAUUCAUGUAUGUGUCCAA | UUAGUCAUGUACAUGAAUCAUUAA | Cleavage |  | 4 |
| sbi-miR5568c-3p | SbNFY-B4 | 1 | -1 | 1 | 21 | 7133 | 7153 | ACUUACAGUUUGGAACGGAGG | CCUCCAUUCCAAACUGUAAGU | Cleavage |  | 2 |
| sbi-miR5568c-3p | SbNFY-B6 | 1 | -1 | 1 | 21 | 3457 | 3477 | ACUUACAGUUUGGAACGGAGG | CCUCCAUUCCAAACUGUAAGU | Cleavage |  | 2 |
| sbi-miR5568g-5p | SbNFY-B10 | 1 | -1 | 1 | 21 | 7806 | 7826 | CAAAUUAUAAGAUGUUUUGGC | AAUAAAGCAUCUUAUAAUUUG | Cleavage |  | 1 |
| sbi-miR6225-3p | SbNFY-B19 | 1 | -1 | 1 | 24 | 227 | 250 | GAAACGAAUCUUUUAAGUCUAAUU | AACUAGGCUUAAAAGAUUUGUUUC | Cleavage |  | 4 |
| sbi-miR437x-5p | SbNFY-B10 | 1.5 | -1 | 1 | 24 | 12389 | 12412 | UAGAGUUGUCCUAAGUCAAACUUU | AAAGUUUGACUUAGGACAACUUUU | Cleavage |  | 1 |
| sbi-miR437x-5p | SbNFY-B14 | 1.5 | -1 | 1 | 24 | 539 | 562 | UAGAGUUGUCCUAAGUCAAACUUU | UAGUUUUGACUUAGGAUGAUUCUA | Cleavage |  | 1 |
| sbi-miR5565a | SbNFY-B14 | 1.5 | -1 | 1 | 24 | 11520 | 11543 | AACACAUGUGGAUUGAGGCGAAUC | GAUUCACCUCAAUCCACGUGUGUU | Cleavage |  | 2 |
| sbi-miR5565b | SbNFY-B14 | 1.5 | -1 | 1 | 24 | 11520 | 11543 | AACACAUGUGGAUUGAGGCGAAUC | GAUUCACCUCAAUCCACGUGUGUU | Cleavage |  | 2 |
| sbi-miR5565c | SbNFY-B14 | 1.5 | -1 | 1 | 21 | 11523 | 11543 | UACACAUGUGGAUUGAGGUGA | UCACCUCAAUCCACGUGUGUU | Cleavage |  | 2 |
| sbi-miR5565g-3p | SbNFY-B14 | 1.5 | -1 | 1 | 24 | 11519 | 11542 | ACACAUGUGGAUUGAGAUGAAUAC | GGAUUCACCUCAAUCCACGUGUGU | Cleavage |  | 2 |
| sbi-miR5568b-5p | SbNFY-B8 | 1.5 | -1 | 1 | 21 | 15061 | 15081 | UUUCUAGGUACAUAGCUUUUG | CAAAAGUUGUGUACUUAGAAA | Cleavage |  | 2 |
| sbi-miR5568c-5p | SbNFY-B3 | 1.5 | -1 | 1 | 21 | 16085 | 16105 | UCUGUUCCAAAUUGUAAGUCG | UGACUUACAAUUUGGGAUGGA | Cleavage |  | 4 |
| sbi-miR5568c-5p | SbNFY-B3 | 1.5 | -1 | 1 | 21 | 7473 | 7493 | UCUGUUCCAAAUUGUAAGUCG | UAGCUUAUAAUUUGGAAUAGA | Cleavage |  | 4 |
| sbi-miR5568c-5p | SbNFY-B4 | 1.5 | -1 | 1 | 21 | 7363 | 7383 | UCUGUUCCAAAUUGUAAGUCG | UGACUUACAAUUUGGGAUGGA | Cleavage |  | 3 |
| sbi-miR5568d-3p | SbNFY-B8 | 1.5 | -1 | 1 | 21 | 15011 | 15031 | AAAGUUGUGUAUCUAGAAAAG | CUUUUCUAAAUACACAACUUU | Cleavage |  | 2 |
| sbi-miR5568d-5p | SbNFY-B4 | 1.5 | -1 | 1 | 21 | 5410 | 5430 | UGGCUUUUCUAGAUACAUAGC | GCUACGUAUUUAGAAAAGCCA | Cleavage |  | 1 |
| sbi-miR5568e-3p | SbNFY-B8 | 1.5 | -1 | 1 | 21 | 15002 | 15022 | UAUCUAGAAAAGCUAAAACGU | UCGUUUUAGCUUUUCUAAAUA | Cleavage |  | 2 |
| sbi-miR5568f-5p | SbNFY-B3 | 1.5 | -1 | 1 | 21 | 16085 | 16105 | UCCAUUCCAAAUUGUAAGAUG | UGACUUACAAUUUGGGAUGGA | Cleavage |  | 4 |
| sbi-miR5568f-5p | SbNFY-B4 | 1.5 | -1 | 1 | 21 | 7363 | 7383 | UCCAUUCCAAAUUGUAAGAUG | UGACUUACAAUUUGGGAUGGA | Cleavage |  | 2 |
| sbi-miR5568g-5p | SbNFY-B6 | 1.5 | -1 | 1 | 21 | 3295 | 3315 | CAAAUUAUAAGAUGUUUUGGC | GUCAAAACGUCUUAUAAUUUA | Cleavage |  | 3 |
| sbi-miR5569 | SbNFY-B14 | 1.5 | -1 | 1 | 24 | 5389 | 5412 | UAUUGCAUGCUUGAACUAUGGUAA | UUACCAUAGUUCAAGCAUACAAUA | Cleavage |  | 1 |
| sbi-miR6220-5p | SbNFY-B4 | 1.5 | -1 | 1 | 24 | 7361 | 7384 | CUCCAUCCUAAAUUAUAAGACAUU | AAUGACUUACAAUUUGGGAUGGAG | Cleavage |  | 2 |
| sbi-miR6220-5p | SbNFY-B6 | 1.5 | -1 | 1 | 24 | 3300 | 3323 | CUCCAUCCUAAAUUAUAAGACAUU | AACGUCUUAUAAUUUAGAAUGGAG | Cleavage |  | 2 |
| sbi-miR6220-5p | SbNFY-B3 | 1.5 | -1 | 1 | 24 | 16083 | 16106 | CUCCAUCCUAAAUUAUAAGACAUU | AAUGACUUACAAUUUGGGAUGGAG | Cleavage |  | 3 |
| sbi-miR6225-3p | SbNFY-B3 | 1.5 | -1 | 1 | 24 | 14085 | 14108 | GAAACGAAUCUUUUAAGUCUAAUU | AAUUAGACUUAAAAGAUUCAUUUC | Cleavage |  | 2 |
| sbi-miR6225-3p | SbNFY-B4 | 1.5 | -1 | 1 | 24 | 6943 | 6966 | GAAACGAAUCUUUUAAGUCUAAUU | GACGGGGCUCAAAAGAUUCGUUUC | Cleavage |  | 7 |
| sbi-miR6225-5p | SbNFY-B5 | 1.5 | -1 | 1 | 24 | 4034 | 4057 | AACUAGACUCAAAAGAUUCAUCUC | GAGAUGAAUCUUUUGAGUCUAAUU | Cleavage |  | 3 |
| sbi-miR6225-5p | SbNFY-B10 | 1.5 | -1 | 1 | 24 | 111 | 134 | AACUAGACUCAAAAGAUUCAUCUC | GAGAUGAAUCUUUUGAGCCUAGUU | Cleavage |  | 5 |
| sbi-miR6225-5p | SbNFY-B10 | 1.5 | -1 | 1 | 24 | 8318 | 8341 | AACUAGACUCAAAAGAUUCAUCUC | GAGACGAAUCUUUUGAGCCUAGUU | Cleavage |  | 5 |
| sbi-miR6225-5p | SbNFY-B10 | 1.5 | -1 | 1 | 24 | 17072 | 17095 | AACUAGACUCAAAAGAUUCAUCUC | GAGACGAAUCUUUUGAGCCUAGUU | Cleavage |  | 5 |
| sbi-miR6225-5p | SbNFY-B6 | 1.5 | -1 | 1 | 24 | 6118 | 6141 | AACUAGACUCAAAAGAUUCAUCUC | GAGAUGAAUCUUUUGAGCCUAGUU | Cleavage |  | 4 |
| sbi-miR6225-5p | SbNFY-B6 | 1.5 | -1 | 1 | 24 | 9663 | 9686 | AACUAGACUCAAAAGAUUCAUCUC | GAGACGAAUCUUUUGAGCCUAGUU | Cleavage |  | 4 |
| sbi-miR6225-5p | SbNFY-B19 | 1.5 | -1 | 1 | 24 | 3840 | 3863 | AACUAGACUCAAAAGAUUCAUCUC | GAAAUGAAUCUUUUGAGCCUAGUU | Cleavage |  | 1 |
| sbi-miR6225-5p | SbNFY-B4 | 1.5 | -1 | 1 | 24 | 3137 | 3160 | AACUAGACUCAAAAGAUUCAUCUC | GAGACGAAUCUUUUGAGCCUAGUU | Cleavage |  | 3 |
| sbi-miR6225-5p | SbNFY-B4 | 1.5 | -1 | 1 | 24 | 6775 | 6798 | AACUAGACUCAAAAGAUUCAUCUC | GAGACGAAUCUUUUGAGCCUAGUU | Cleavage |  | 3 |
| sbi-miR6225-5p | SbNFY-B9 | 1.5 | -1 | 1 | 24 | 4138 | 4161 | AACUAGACUCAAAAGAUUCAUCUC | GAAACGAAUCUUUUGAGCCUAGUU | Cleavage |  | 3 |
| sbi-miR6225-5p | SbNFY-B18 | 1.5 | -1 | 1 | 24 | 16822 | 16845 | AACUAGACUCAAAAGAUUCAUCUC | GAAGCGAAUCUUUUGAGCCUAGUU | Cleavage |  | 3 |
| sbi-miR6232a-3p | SbNFY-B12 | 1.5 | -1 | 1 | 24 | 14085 | 14108 | UGGAUGUACCAAAAAAGUCAAAGC | GCUUUGACUUUUUUGGUACAUCGA | Cleavage |  | 1 |
| sbi-miR1435a | SbNFY-B10 | 2 | -1 | 1 | 20 | 12387 | 12406 | UUUCUUAAGUCAAACUUUUC | CAAAAGUUUGACUUAGGACA | Cleavage |  | 1 |
| sbi-miR437a | SbNFY-B2 | 2 | -1 | 1 | 21 | 10665 | 10685 | AAAGUUAGAGAAGUUUGACUU | CGGUCAAACUUUUCCAACUUU | Cleavage |  | 1 |
| sbi-miR437b | SbNFY-B2 | 2 | -1 | 1 | 21 | 10665 | 10685 | AAAGUUAGAGAAGUUUGACUU | CGGUCAAACUUUUCCAACUUU | Cleavage |  | 1 |
| sbi-miR437c | SbNFY-B2 | 2 | -1 | 1 | 21 | 10665 | 10685 | AAAGUUAGAGAAGUUUGACUU | CGGUCAAACUUUUCCAACUUU | Cleavage |  | 1 |
| sbi-miR437d | SbNFY-B2 | 2 | -1 | 1 | 21 | 10665 | 10685 | AAAGUUAGAGAAGUUUGACUU | CGGUCAAACUUUUCCAACUUU | Cleavage |  | 1 |
| sbi-miR437e | SbNFY-B2 | 2 | -1 | 1 | 21 | 10665 | 10685 | AAAGUUAGAGAAGUUUGACUU | CGGUCAAACUUUUCCAACUUU | Cleavage |  | 1 |
| sbi-miR437f | SbNFY-B2 | 2 | -1 | 1 | 21 | 10665 | 10685 | AAAGUUAGAGAAGUUUGACUU | CGGUCAAACUUUUCCAACUUU | Cleavage |  | 1 |
| sbi-miR437g | SbNFY-B2 | 2 | -1 | 1 | 21 | 10665 | 10685 | AAAGUUAGAGAAGUUUGACUU | CGGUCAAACUUUUCCAACUUU | Cleavage |  | 1 |
| sbi-miR437i | SbNFY-B2 | 2 | -1 | 1 | 21 | 10665 | 10685 | AAAGUUAGAGAAGUUUGACUU | CGGUCAAACUUUUCCAACUUU | Cleavage |  | 1 |
| sbi-miR437j | SbNFY-B2 | 2 | -1 | 1 | 21 | 10665 | 10685 | AAAGUUAGAGAAGUUUGACUU | CGGUCAAACUUUUCCAACUUU | Cleavage |  | 1 |
| sbi-miR437k | SbNFY-B2 | 2 | -1 | 1 | 21 | 10665 | 10685 | AAAGUUAGAGAAGUUUGACUU | CGGUCAAACUUUUCCAACUUU | Cleavage |  | 1 |
| sbi-miR437l | SbNFY-B2 | 2 | -1 | 1 | 21 | 10665 | 10685 | AAAGUUAGAGAAGUUUGACUU | CGGUCAAACUUUUCCAACUUU | Cleavage |  | 1 |
| sbi-miR437m | SbNFY-B2 | 2 | -1 | 1 | 21 | 10665 | 10685 | AAAGUUAGAGAAGUUUGACUU | CGGUCAAACUUUUCCAACUUU | Cleavage |  | 1 |
| sbi-miR437n | SbNFY-B2 | 2 | -1 | 1 | 21 | 10665 | 10685 | AAAGUUAGAGAAGUUUGACUU | CGGUCAAACUUUUCCAACUUU | Cleavage |  | 1 |
| sbi-miR437o | SbNFY-B2 | 2 | -1 | 1 | 21 | 10665 | 10685 | AAAGUUAGAGAAGUUUGACUU | CGGUCAAACUUUUCCAACUUU | Cleavage |  | 1 |
| sbi-miR437p | SbNFY-B2 | 2 | -1 | 1 | 21 | 10665 | 10685 | AAAGUUAGAGAAGUUUGACUU | CGGUCAAACUUUUCCAACUUU | Cleavage |  | 1 |
| sbi-miR437q | SbNFY-B2 | 2 | -1 | 1 | 21 | 10665 | 10685 | AAAGUUAGAGAAGUUUGACUU | CGGUCAAACUUUUCCAACUUU | Cleavage |  | 1 |
| sbi-miR437r | SbNFY-B2 | 2 | -1 | 1 | 21 | 10665 | 10685 | AAAGUUAGAGAAGUUUGACUU | CGGUCAAACUUUUCCAACUUU | Cleavage |  | 1 |
| sbi-miR437s | SbNFY-B2 | 2 | -1 | 1 | 21 | 10665 | 10685 | AAAGUUAGAGAAGUUUGACUU | CGGUCAAACUUUUCCAACUUU | Cleavage |  | 1 |
| sbi-miR437t | SbNFY-B2 | 2 | -1 | 1 | 21 | 10665 | 10685 | AAAGUUAGAGAAGUUUGACUU | CGGUCAAACUUUUCCAACUUU | Cleavage |  | 1 |
| sbi-miR437u | SbNFY-B2 | 2 | -1 | 1 | 21 | 10665 | 10685 | AAAGUUAGAGAAGUUUGACUU | CGGUCAAACUUUUCCAACUUU | Cleavage |  | 1 |
| sbi-miR437v | SbNFY-B2 | 2 | -1 | 1 | 21 | 10665 | 10685 | AAAGUUAGAGAAGUUUGACUU | CGGUCAAACUUUUCCAACUUU | Cleavage |  | 1 |
| sbi-miR437w | SbNFY-B2 | 2 | -1 | 1 | 21 | 10665 | 10685 | AAAGUUAGAGAAGUUUGACUU | CGGUCAAACUUUUCCAACUUU | Cleavage |  | 1 |
| sbi-miR5565a | SbNFY-B14 | 2 | -1 | 1 | 24 | 17397 | 17420 | AACACAUGUGGAUUGAGGCGAAUC | GAUUCACAUCAAUCCACAUGUGUU | Cleavage |  | 2 |
| sbi-miR5565b | SbNFY-B14 | 2 | -1 | 1 | 24 | 17397 | 17420 | AACACAUGUGGAUUGAGGCGAAUC | GAUUCACAUCAAUCCACAUGUGUU | Cleavage |  | 2 |
| sbi-miR5565c | SbNFY-B14 | 2 | -1 | 1 | 21 | 17400 | 17420 | UACACAUGUGGAUUGAGGUGA | UCACAUCAAUCCACAUGUGUU | Cleavage |  | 2 |
| sbi-miR5565g-3p | SbNFY-B14 | 2 | -1 | 1 | 24 | 17396 | 17419 | ACACAUGUGGAUUGAGAUGAAUAC | GGAUUCACAUCAAUCCACAUGUGU | Cleavage |  | 2 |
| sbi-miR5567 | SbNFY-B4 | 2 | -1 | 1 | 24 | 4500 | 4524 | UUAAUGAUUCAUGUAUGUGUC-CAA | UUGUGGCACAUGCAUGAAACAUUAA | Cleavage |  | 4 |
| sbi-miR5568a | SbNFY-B12 | 2 | -1 | 1 | 21 | 14070 | 14090 | CAGAGCGACUUACAAUUUGGA | UUCAAAUUAUAAGUCGCUUUG | Cleavage |  | 2 |
| sbi-miR5568c-5p | SbNFY-B4 | 2 | -1 | 1 | 21 | 11812 | 11832 | UCUGUUCCAAAUUGUAAGUCG | UGACUUAUAAUUUGGGAUGGA | Cleavage |  | 3 |
| sbi-miR5568c-5p | SbNFY-B10 | 2 | -1 | 1 | 21 | 7813 | 7833 | UCUGUUCCAAAUUGUAAGUCG | CAUCUUAUAAUUUGGAACGGA | Cleavage |  | 1 |
| sbi-miR5568c-5p | SbNFY-B8 | 2 | -1 | 1 | 21 | 12383 | 12403 | UCUGUUCCAAAUUGUAAGUCG | UAAUUUAUAGUUUGGAACGGA | Cleavage |  | 2 |
| sbi-miR5568d-3p | SbNFY-B8 | 2 | -1 | 1 | 21 | 8683 | 8703 | AAAGUUGUGUAUCUAGAAAAG | CUUUUCUGAAUACACAACUUU | Cleavage |  | 2 |
| sbi-miR5568d-5p | SbNFY-B6 | 2 | -1 | 1 | 21 | 3278 | 3298 | UGGCUUUUCUAGAUACAUAGC | ACUAUGAAUUUAGAAAAGUCA | Cleavage |  | 1 |
| sbi-miR5568e-3p | SbNFY-B8 | 2 | -1 | 1 | 21 | 8674 | 8694 | UAUCUAGAAAAGCUAAAACGU | CUGUUUUAGCUUUUCUGAAUA | Cleavage |  | 2 |
| sbi-miR5568f-3p | SbNFY-B6 | 2 | -1 | 1 | 21 | 3185 | 3205 | GUCUUAUAAUUUGGAAUGGAG | CUCUGUCCCAAAUUAUAAGAC | Cleavage |  | 2 |
| sbi-miR5568f-3p | SbNFY-B19 | 2 | -1 | 1 | 21 | 17613 | 17633 | GUCUUAUAAUUUGGAAUGGAG | UUCUCUUCUAAAUUAUAAGAC | Cleavage |  | 1 |
| sbi-miR5568f-5p | SbNFY-B10 | 2 | -1 | 1 | 21 | 7813 | 7833 | UCCAUUCCAAAUUGUAAGAUG | CAUCUUAUAAUUUGGAACGGA | Cleavage |  | 2 |
| sbi-miR5568f-5p | SbNFY-B6 | 2 | -1 | 1 | 21 | 3302 | 3322 | UCCAUUCCAAAUUGUAAGAUG | CGUCUUAUAAUUUAGAAUGGA | Cleavage |  | 3 |
| sbi-miR5568f-5p | SbNFY-B4 | 2 | -1 | 1 | 21 | 11812 | 11832 | UCCAUUCCAAAUUGUAAGAUG | UGACUUAUAAUUUGGGAUGGA | Cleavage |  | 2 |
| sbi-miR5568f-5p | SbNFY-B5 | 2 | -1 | 1 | 21 | 14390 | 14410 | UCCAUUCCAAAUUGUAAGAUG | AUUUUUACAAUUUAGAAUGGA | Cleavage |  | 2 |
| sbi-miR5568g-3p | SbNFY-B12 | 2 | -1 | 1 | 21 | 4931 | 4951 | AAAACGUCUUAUAAUUUGGAG | UUCCAAAUUAUAAGUUGUUUU | Cleavage |  | 2 |
| sbi-miR5568g-3p | SbNFY-B19 | 2 | -1 | 1 | 21 | 17618 | 17638 | AAAACGUCUUAUAAUUUGGAG | UUCUAAAUUAUAAGACUUUUU | Cleavage |  | 1 |
| sbi-miR5568g-5p | SbNFY-B12 | 2 | -1 | 1 | 21 | 5034 | 5054 | CAAAUUAUAAGAUGUUUUGGC | GUCAAAGCAACUUAUAAUUUG | Cleavage |  | 2 |
| sbi-miR5568g-5p | SbNFY-B3 | 2 | -1 | 1 | 21 | 7466 | 7486 | CAAAUUAUAAGAUGUUUUGGC | ACCAAAAUAGCUUAUAAUUUG | Cleavage |  | 1 |
| sbi-miR6220-3p | SbNFY-B4 | 2 | -1 | 1 | 24 | 11584 | 11607 | AUGCCUUAUAAUUUGGGAUGGAGA | CCUCCAUCCCAAAUUAUAAGUUAU | Cleavage |  | 1 |
| sbi-miR6225-5p | SbNFY-B10 | 2 | -1 | 1 | 24 | 13684 | 13707 | AACUAGACUCAAAAGAUUCAUCUC | AAGACGGAUCUUUUGAGCCUAGUU | Cleavage |  | 5 |
| sbi-miR6225-5p | SbNFY-B6 | 2 | -1 | 1 | 24 | 10343 | 10366 | AACUAGACUCAAAAGAUUCAUCUC | GAGACGAAUUUUUUAAGUCUAGUU | Translation | | 4 |
| sbi-miR6225-5p | SbNFY-B8 | 2 | -1 | 1 | 24 | 2241 | 2264 | AACUAGACUCAAAAGAUUCAUCUC | GAGACGGAUCUUUUGAGUCUAGCU | Cleavage |  | 1 |
| sbi-miR6230-3p | SbNFY-B3 | 2 | -1 | 1 | 21 | 17697 | 17717 | UAACAAGUUUAGGGAUCUAGA | CAUAGGCCUCUAAACUUGUUA | Cleavage |  | 1 |
| sbi-miR6235-5p | SbNFY-B19 | 2 | -1 | 1 | 24 | 488 | 511 | UUGUGAGAGAAAAAUACUGUUGGC | UUCAAUAAUGUUUUUCUCUCACAA | Cleavage |  | 1 |
| sbi-miR437a | SbNFY-B9 | 2.5 | -1 | 1 | 21 | 7903 | 7923 | AAAGUUAGAGAAGUUUGACUU | AAGUCAAACUUUUUAAACUUU | Cleavage |  | 2 |
| sbi-miR437b | SbNFY-B9 | 2.5 | -1 | 1 | 21 | 7903 | 7923 | AAAGUUAGAGAAGUUUGACUU | AAGUCAAACUUUUUAAACUUU | Cleavage |  | 2 |
| sbi-miR437c | SbNFY-B9 | 2.5 | -1 | 1 | 21 | 7903 | 7923 | AAAGUUAGAGAAGUUUGACUU | AAGUCAAACUUUUUAAACUUU | Cleavage |  | 2 |
| sbi-miR437d | SbNFY-B9 | 2.5 | -1 | 1 | 21 | 7903 | 7923 | AAAGUUAGAGAAGUUUGACUU | AAGUCAAACUUUUUAAACUUU | Cleavage |  | 2 |
| sbi-miR437e | SbNFY-B9 | 2.5 | -1 | 1 | 21 | 7903 | 7923 | AAAGUUAGAGAAGUUUGACUU | AAGUCAAACUUUUUAAACUUU | Cleavage |  | 2 |
| sbi-miR437f | SbNFY-B9 | 2.5 | -1 | 1 | 21 | 7903 | 7923 | AAAGUUAGAGAAGUUUGACUU | AAGUCAAACUUUUUAAACUUU | Cleavage |  | 2 |
| sbi-miR437g | SbNFY-B9 | 2.5 | -1 | 1 | 21 | 7903 | 7923 | AAAGUUAGAGAAGUUUGACUU | AAGUCAAACUUUUUAAACUUU | Cleavage |  | 2 |
| sbi-miR437i | SbNFY-B9 | 2.5 | -1 | 1 | 21 | 7903 | 7923 | AAAGUUAGAGAAGUUUGACUU | AAGUCAAACUUUUUAAACUUU | Cleavage |  | 2 |
| sbi-miR437j | SbNFY-B9 | 2.5 | -1 | 1 | 21 | 7903 | 7923 | AAAGUUAGAGAAGUUUGACUU | AAGUCAAACUUUUUAAACUUU | Cleavage |  | 2 |
| sbi-miR437k | SbNFY-B9 | 2.5 | -1 | 1 | 21 | 7903 | 7923 | AAAGUUAGAGAAGUUUGACUU | AAGUCAAACUUUUUAAACUUU | Cleavage |  | 2 |
| sbi-miR437l | SbNFY-B9 | 2.5 | -1 | 1 | 21 | 7903 | 7923 | AAAGUUAGAGAAGUUUGACUU | AAGUCAAACUUUUUAAACUUU | Cleavage |  | 2 |
| sbi-miR437m | SbNFY-B9 | 2.5 | -1 | 1 | 21 | 7903 | 7923 | AAAGUUAGAGAAGUUUGACUU | AAGUCAAACUUUUUAAACUUU | Cleavage |  | 2 |
| sbi-miR437n | SbNFY-B9 | 2.5 | -1 | 1 | 21 | 7903 | 7923 | AAAGUUAGAGAAGUUUGACUU | AAGUCAAACUUUUUAAACUUU | Cleavage |  | 2 |
| sbi-miR437o | SbNFY-B9 | 2.5 | -1 | 1 | 21 | 7903 | 7923 | AAAGUUAGAGAAGUUUGACUU | AAGUCAAACUUUUUAAACUUU | Cleavage |  | 2 |
| sbi-miR437p | SbNFY-B9 | 2.5 | -1 | 1 | 21 | 7903 | 7923 | AAAGUUAGAGAAGUUUGACUU | AAGUCAAACUUUUUAAACUUU | Cleavage |  | 2 |
| sbi-miR437q | SbNFY-B9 | 2.5 | -1 | 1 | 21 | 7903 | 7923 | AAAGUUAGAGAAGUUUGACUU | AAGUCAAACUUUUUAAACUUU | Cleavage |  | 2 |
| sbi-miR437r | SbNFY-B9 | 2.5 | -1 | 1 | 21 | 7903 | 7923 | AAAGUUAGAGAAGUUUGACUU | AAGUCAAACUUUUUAAACUUU | Cleavage |  | 2 |
| sbi-miR437s | SbNFY-B9 | 2.5 | -1 | 1 | 21 | 7903 | 7923 | AAAGUUAGAGAAGUUUGACUU | AAGUCAAACUUUUUAAACUUU | Cleavage |  | 2 |
| sbi-miR437t | SbNFY-B9 | 2.5 | -1 | 1 | 21 | 7903 | 7923 | AAAGUUAGAGAAGUUUGACUU | AAGUCAAACUUUUUAAACUUU | Cleavage |  | 2 |
| sbi-miR437u | SbNFY-B9 | 2.5 | -1 | 1 | 21 | 7903 | 7923 | AAAGUUAGAGAAGUUUGACUU | AAGUCAAACUUUUUAAACUUU | Cleavage |  | 2 |
| sbi-miR437v | SbNFY-B9 | 2.5 | -1 | 1 | 21 | 7903 | 7923 | AAAGUUAGAGAAGUUUGACUU | AAGUCAAACUUUUUAAACUUU | Cleavage |  | 2 |
| sbi-miR437w | SbNFY-B9 | 2.5 | -1 | 1 | 21 | 7903 | 7923 | AAAGUUAGAGAAGUUUGACUU | AAGUCAAACUUUUUAAACUUU | Cleavage |  | 2 |
| sbi-miR5386 | SbNFY-B16 | 2.5 | -1 | 1 | 20 | 8155 | 8174 | CGUCGCUGUCGCGCGCGCUG | GAGCGAGCGCGGCGGUGACG | Cleavage |  | 3 |
| sbi-miR5389 | SbNFY-B18 | 2.5 | -1 | 1 | 21 | 15335 | 15355 | GCUUGAGUUUAUCAGCCGAGU | AUUUGACAGAUAAACUCAAGC | Cleavage |  | 2 |
| sbi-miR5565d | SbNFY-B14 | 2.5 | -1 | 1 | 24 | 17465 | 17488 | ACUUCAAUCCAUGUAUGUUGGUGU | ACUUCAACACAUGUGGAUUGAGGU | Cleavage |  | 2 |
| sbi-miR5565e | SbNFY-B14 | 2.5 | -1 | 1 | 19 | 11592 | 11610 | UUGUUUGGAUGUUGUCGGA | UUCGACUACAUUCAAACAA | Cleavage |  | 2 |
| sbi-miR5565f | SbNFY-B14 | 2.5 | -1 | 1 | 20 | 11580 | 11599 | UAGUCGGAUUUAUAUCAAUC | GAUUAAUGUGAAUUCGACUA | Cleavage |  | 2 |
| sbi-miR5565f | SbNFY-B10 | 2.5 | -1 | 1 | 20 | 539 | 558 | UAGUCGGAUUUAUAUCAAUC | AGUUCAUAUGAAUUCGACUA | Cleavage |  | 1 |
| sbi-miR5565g-5p | SbNFY-B14 | 2.5 | -1 | 1 | 24 | 17468 | 17491 | UUCACAUCAAUCCACAUAUGUUGG | UCAACACAUGUGGAUUGAGGUGAA | Cleavage |  | 2 |
| sbi-miR5565g-5p | SbNFY-B14 | 2.5 | -1 | 1 | 24 | 11568 | 11591 | UUCACAUCAAUCCACAUAUGUUGG | CCGAUACAUGUGGAUUAAUGUGAA | Cleavage |  | 2 |
| sbi-miR5567 | SbNFY-B5 | 2.5 | -1 | 1 | 24 | 12381 | 12404 | UUAAUGAUUCAUGUAUGUGUCCAA | UUAGACACAUGCAUGGAACAUUAA | Cleavage |  | 5 |
| sbi-miR5567 | SbNFY-B10 | 2.5 | -1 | 1 | 24 | 17001 | 17024 | UUAAUGAUUCAUGUAUGUGUCCAA | UUAGACACAUACAUGGAGUAUUAA | Cleavage |  | 6 |
| sbi-miR5567 | SbNFY-B8 | 2.5 | -1 | 1 | 24 | 2170 | 2193 | UUAAUGAUUCAUGUAUGUGUCCAA | UUAGACGCAUGCAUGAAGCAUUAA | Cleavage |  | 2 |
| sbi-miR5567 | SbNFY-B4 | 2.5 | -1 | 1 | 24 | 4821 | 4844 | UUAAUGAUUCAUGUAUGUGUCCAA | CGCGAUAUAUACAUGAAACAUUAA | Cleavage |  | 4 |
| sbi-miR5567 | SbNFY-B2 | 2.5 | -1 | 1 | 24 | 4277 | 4301 | UUAAUGAUUCAUGUAUGUGUC-CAA | UUGCGGCGCAUGCAUGAAGCAUUAA | Cleavage |  | 1 |
| sbi-miR5567 | SbNFY-B19 | 2.5 | -1 | 1 | 24 | 3767 | 3791 | UUAAUGAUUCAUGUAUGUGUC-CAA | UUGCGGCACAUGCAUGGAGCAUUAA | Cleavage |  | 1 |
| sbi-miR5568b-5p | SbNFY-B4 | 2.5 | -1 | 1 | 21 | 5405 | 5425 | UUUCUAGGUACAUAGCUUUUG | UAAAAGCUACGUAUUUAGAAA | Cleavage |  | 1 |
| sbi-miR5568c-5p | SbNFY-B12 | 2.5 | -1 | 1 | 21 | 14153 | 14173 | UCUGUUCCAAAUUGUAAGUCG | CAACUUAUAAUUUGGAAAGGA | Cleavage |  | 2 |
| sbi-miR5568d-5p | SbNFY-B8 | 2.5 | -1 | 1 | 21 | 15066 | 15086 | UGGCUUUUCUAGAUACAUAGC | GUUGUGUACUUAGAAAAGCCA | Cleavage |  | 2 |
| sbi-miR5568g-5p | SbNFY-B12 | 2.5 | -1 | 1 | 21 | 14146 | 14166 | CAAAUUAUAAGAUGUUUUGGC | GCCAAACCAACUUAUAAUUUG | Cleavage |  | 2 |
| sbi-miR5568g-5p | SbNFY-B18 | 2.5 | -1 | 1 | 21 | 16705 | 16725 | CAAAUUAUAAGAUGUUUUGGC | UCUAAAAUAUUUUAUAAUUUU | Cleavage |  | 2 |
| sbi-miR6220-3p | SbNFY-B6 | 2.5 | -1 | 1 | 24 | 3184 | 3207 | AUGCCUUAUAAUUUGGGAUGGAGA | CCUCUGUCCCAAAUUAUAAGACGU | Cleavage |  | 2 |
| sbi-miR6225-3p | SbNFY-B19 | 2.5 | -1 | 1 | 24 | 17444 | 17467 | GAAACGAAUCUUUUAAGUCUAAUU | AACUAGACUCAAAAGAUUCGUCUC | Cleavage |  | 4 |
| sbi-miR6225-3p | SbNFY-B4 | 2.5 | -1 | 1 | 24 | 5129 | 5152 | GAAACGAAUCUUUUAAGUCUAAUU | AACUAGACUUAAAAGAUUUAUUUU | Cleavage |  | 7 |
| sbi-miR6225-3p | SbNFY-B1 | 2.5 | -1 | 1 | 24 | 14932 | 14955 | GAAACGAAUCUUUUAAGUCUAAUU | AACUAGGCUUAAAAGAUUUGUCUC | Cleavage |  | 1 |
| sbi-miR6225-3p | SbNFY-B14 | 2.5 | -1 | 1 | 24 | 11308 | 11331 | GAAACGAAUCUUUUAAGUCUAAUU | AACUAGACUCAAAAGAUUCGUCUC | Cleavage |  | 1 |
| sbi-miR6225-3p | SbNFY-B15 | 2.5 | -1 | 1 | 24 | 8109 | 8132 | GAAACGAAUCUUUUAAGUCUAAUU | AACUAGACUCAAAAGAUUCGUCUC | Cleavage |  | 1 |
| sbi-miR6225-5p | SbNFY-B9 | 2.5 | -1 | 1 | 24 | 11001 | 11024 | AACUAGACUCAAAAGAUUCAUCUC | GAGACAAAUCUUUUGAGUAUAGUU | Cleavage |  | 3 |
| sbi-miR6232a-5p | SbNFY-B5 | 2.5 | -1 | 1 | 24 | 8070 | 8093 | GUCGCUUUGACUUUUUUGGUACAU | UGAAACAAAAAAAGUCAAAGCAAC | Cleavage |  | 2 |
| sbi-miR821e | SbNFY-B17 | 2.5 | -1 | 1 | 21 | 17031 | 17051 | AAGUCAUCAAAAUAAAAGUUG | UGACUUUUAUUUUGGUCAUUU | Cleavage |  | 1 |
| sbi-miR1435b | SbNFY-B10 | 3 | -1 | 1 | 20 | 12387 | 12406 | UUUCUUAAGUCAAACCUUUU | CAAAAGUUUGACUUAGGACA | Cleavage |  | 1 |
| sbi-miR319a | SbNFY-B17 | 3 | -1 | 1 | 20 | 12186 | 12205 | UUGGACUGAAGGGUGCUCCC | UGGGCUACUCUUCAGUUCAA | Cleavage |  | 1 |
| sbi-miR319b | SbNFY-B17 | 3 | -1 | 1 | 20 | 12186 | 12205 | UUGGACUGAAGGGUGCUCCC | UGGGCUACUCUUCAGUUCAA | Cleavage |  | 1 |
| sbi-miR528 | SbNFY-B5 | 3 | -1 | 1 | 21 | 5988 | 6008 | UGGAAGGGGCAUGCAGAGGAG | UGUCUCUGCUUGCCCUUUUCA | Cleavage |  | 1 |
| sbi-miR5381 | SbNFY-B16 | 3 | -1 | 1 | 19 | 17142 | 17160 | AAGAUCUGUGGCGCCGAGC | UUUCGGCGUCGCAGAUUUU | Cleavage |  | 1 |
| sbi-miR5389 | SbNFY-B18 | 3 | -1 | 1 | 21 | 16246 | 16266 | GCUUGAGUUUAUCAGCCGAGU | AUUCGAUUGAUAAGCUCAAGG | Cleavage |  | 2 |
| sbi-miR5567 | SbNFY-B6 | 3 | -1 | 1 | 24 | 7043 | 7067 | UUAAUGAUUCAUGUAUGUGUC-CAA | UUGCGGCACAUGCAUGAAGCAUUAU | Cleavage |  | 5 |
| sbi-miR5567 | SbNFY-B5 | 3 | -1 | 1 | 24 | 3963 | 3986 | UUAAUGAUUCAUGUAUGUGUCCAA | UUGGUCGCAUGCAUGGAGCAUUAA | Cleavage |  | 5 |
| sbi-miR5567 | SbNFY-B9 | 3 | -1 | 1 | 24 | 10931 | 10954 | UUAAUGAUUCAUGUAUGUGUCCAA | UGCAACACAUGCAUGGAGUAUUAA | Cleavage |  | 5 |
| sbi-miR5567 | SbNFY-B18 | 3 | -1 | 1 | 24 | 15492 | 15515 | UUAAUGAUUCAUGUAUGUGUCCAA | GUAGGCACAUGCAUGGAGCAUUAG | Cleavage |  | 7 |
| sbi-miR5567 | SbNFY-B3 | 3 | -1 | 1 | 24 | 11568 | 11592 | UUAAUGAUUCAUGUAUGUGUC-CAA | UUGCGGUAUAUACAUGGAACAUUAA | Cleavage |  | 1 |
| sbi-miR5567 | SbNFY-B13 | 3 | -1 | 1 | 24 | 12298 | 12321 | UUAAUGAUUCAUGUAUGUGUCCAA | UGCAGUACAUGCAUGGAGCAUUAA | Cleavage |  | 1 |
| sbi-miR5568a | SbNFY-B12 | 3 | -1 | 1 | 21 | 4932 | 4952 | CAGAGCGACUUACAAUUUGGA | UCCAAAUUAUAAGUUGUUUUG | Cleavage |  | 2 |
| sbi-miR5568a | SbNFY-B6 | 3 | -1 | 1 | 21 | 3464 | 3484 | CAGAGCGACUUACAAUUUGGA | UCCAAACUGUAAGUCGUUUUA | Cleavage |  | 2 |
| sbi-miR5568c-3p | SbNFY-B12 | 3 | -1 | 1 | 21 | 14063 | 14083 | ACUUACAGUUUGGAACGGAGG | ACUCUGUUUCAAAUUAUAAGU | Cleavage |  | 2 |
| sbi-miR5568d-5p | SbNFY-B8 | 3 | -1 | 1 | 21 | 12359 | 12379 | UGGCUUUUCUAGAUACAUAGC | GCUAUUUAUCUAGAAAAGAUA | Cleavage |  | 2 |
| sbi-miR5568e-5p | SbNFY-B6 | 3 | -1 | 1 | 21 | 3285 | 3305 | GAUGUUUUGGGUUUUCUAGAU | AUUUAGAAAAGUCAAAACGUC | Translation | | 1 |
| sbi-miR5568f-3p | SbNFY-B12 | 3 | -1 | 1 | 21 | 14064 | 14084 | GUCUUAUAAUUUGGAAUGGAG | CUCUGUUUCAAAUUAUAAGUC | Cleavage |  | 2 |
| sbi-miR5568f-5p | SbNFY-B3 | 3 | -1 | 1 | 21 | 7473 | 7493 | UCCAUUCCAAAUUGUAAGAUG | UAGCUUAUAAUUUGGAAUAGA | Cleavage |  | 4 |
| sbi-miR5568f-5p | SbNFY-B12 | 3 | -1 | 1 | 21 | 14153 | 14173 | UCCAUUCCAAAUUGUAAGAUG | CAACUUAUAAUUUGGAAAGGA | Cleavage |  | 1 |
| sbi-miR5568g-3p | SbNFY-B6 | 3 | -1 | 1 | 21 | 3463 | 3483 | AAAACGUCUUAUAAUUUGGAG | UUCCAAACUGUAAGUCGUUUU | Cleavage |  | 2 |
| sbi-miR5568g-3p | SbNFY-B10 | 3 | -1 | 1 | 21 | 7700 | 7720 | AAAACGUCUUAUAAUUUGGAG | UCCCAAAUUACAAGAUGUUUC | Translation | | 2 |
| sbi-miR6220-5p | SbNFY-B6 | 3 | -1 | 1 | 24 | 10941 | 10964 | CUCCAUCCUAAAUUAUAAGACAUU | AAUGAGUUAGAGUUUGGGAUGGAG | Cleavage |  | 2 |
| sbi-miR6220-5p | SbNFY-B5 | 3 | -1 | 1 | 24 | 14388 | 14411 | CUCCAUCCUAAAUUAUAAGACAUU | AGAUUUUUACAAUUUAGAAUGGAG | Cleavage |  | 2 |
| sbi-miR6220-5p | SbNFY-B7 | 3 | -1 | 1 | 24 | 16146 | 16168 | CUCCAUCCUAAAUUAUAAGACAUU | CCUAUUUUA-GAUUUAGGAUGGAG | Cleavage |  | 1 |
| sbi-miR6225-3p | SbNFY-B8 | 3 | -1 | 1 | 24 | 1027 | 1050 | GAAACGAAUCUUUUAAGUCUAAUU | AAUUAGGCUAAAAAGAUUCGUCUC | Cleavage |  | 1 |
| sbi-miR6225-3p | SbNFY-B16 | 3 | -1 | 1 | 24 | 308 | 331 | GAAACGAAUCUUUUAAGUCUAAUU | AAUUAGGCUCAAAAGAUUCGUCUC | Cleavage |  | 2 |
| sbi-miR6225-3p | SbNFY-B10 | 3 | -1 | 1 | 24 | 12990 | 13013 | GAAACGAAUCUUUUAAGUCUAAUU | AACUACGCUUAAAAGAUUCGUCUC | Cleavage |  | 4 |
| sbi-miR6225-3p | SbNFY-B11 | 3 | -1 | 1 | 24 | 15342 | 15365 | GAAACGAAUCUUUUAAGUCUAAUU | AACUAGGCUCAAAAGAUUCGUCUC | Cleavage |  | 1 |
| sbi-miR6225-3p | SbNFY-B18 | 3 | -1 | 1 | 24 | 11949 | 11972 | GAAACGAAUCUUUUAAGUCUAAUU | AACUAGGCUCAAAAGAUUCAUUUC | Cleavage |  | 1 |
| sbi-miR6225-5p | SbNFY-B5 | 3 | -1 | 1 | 24 | 2869 | 2892 | AACUAGACUCAAAAGAUUCAUCUC | GAGACGAAUCUUUUAAGCCUAGUU | Translation | | 3 |
| sbi-miR6225-5p | SbNFY-B5 | 3 | -1 | 1 | 24 | 12452 | 12475 | AACUAGACUCAAAAGAUUCAUCUC | GAGACGAAUCUUUUAAGCCUAGUU | Translation | | 3 |
| sbi-miR6225-5p | SbNFY-B6 | 3 | -1 | 1 | 24 | 7115 | 7138 | AACUAGACUCAAAAGAUUCAUCUC | GAGACGAAUCUUUUGAUCCUAGUU | Cleavage |  | 4 |
| sbi-miR6225-5p | SbNFY-B4 | 3 | -1 | 1 | 24 | 4890 | 4913 | AACUAGACUCAAAAGAUUCAUCUC | GAGACGAAUCUUUUGAUGCUAGUU | Cleavage |  | 3 |
| sbi-miR6225-5p | SbNFY-B7 | 3 | -1 | 1 | 24 | 12274 | 12297 | AACUAGACUCAAAAGAUUCAUCUC | GAGACGAAUCUUUUUAAUCUAGUU | Translation | | 2 |
| sbi-miR6225-5p | SbNFY-B2 | 3 | -1 | 1 | 24 | 4349 | 4372 | AACUAGACUCAAAAGAUUCAUCUC | GAGACGAAUCUUUUAAGCCUAGUU | Translation | | 2 |
| sbi-miR6225-5p | SbNFY-B13 | 3 | -1 | 1 | 24 | 12365 | 12388 | AACUAGACUCAAAAGAUUCAUCUC | ACGACGAAUCUUUUGAGCCUAAUU | Cleavage |  | 1 |
| sbi-miR6230-5p | SbNFY-B3 | 3 | -1 | 1 | 21 | 17642 | 17662 | UUUUGGGUCCCUAAACUUGUU | AACAAGUUUAGAGGCCUAGAA | Translation | | 2 |
| sbi-miR6232a-5p | SbNFY-B14 | 3 | -1 | 1 | 24 | 9943 | 9968 | GUCGCUUUGACUUUU--UUGGUACAU | AUGAGCUAAGAAAAAGUCAAAGCGAC | Cleavage |  | 1 |
| sbi-miR6232b-5p | SbNFY-B12 | 3 | -1 | 1 | 21 | 14124 | 14144 | UUUUUGGUACAUUGAAUUUGC | AGACAUUGGAUGUACUAAAAA | Cleavage |  | 4 |
| sbi-miR6233-5p | SbNFY-B3 | 3 | -1 | 1 | 24 | 2619 | 2642 | UGUUGAGGCUGGAGCGAAACUCGG | UGGCGCUUUGUUCCAGCCUUAGCA | Cleavage |  | 1 |
| sbi-miR821e | SbNFY-B11 | 3 | -1 | 1 | 21 | 9174 | 9194 | AAGUCAUCAAAAUAAAAGUUG | UAGUUUUGAUUUUGAUGGUUU | Cleavage |  | 2 |
| sbi-miR156a | SbNFY-B5 | 3.5 | -1 | 1 | 20 | 4536 | 4555 | UGACAGAAGAGAGUGAGCAC | UAUUUCACUCUCUUCUGUUG | Cleavage |  | 2 |
| sbi-miR156b | SbNFY-B5 | 3.5 | -1 | 1 | 20 | 4536 | 4555 | UGACAGAAGAGAGUGAGCAC | UAUUUCACUCUCUUCUGUUG | Cleavage |  | 2 |
| sbi-miR156c | SbNFY-B5 | 3.5 | -1 | 1 | 20 | 4536 | 4555 | UGACAGAAGAGAGUGAGCAC | UAUUUCACUCUCUUCUGUUG | Cleavage |  | 2 |
| sbi-miR156f | SbNFY-B5 | 3.5 | -1 | 1 | 20 | 4536 | 4555 | UGACAGAAGAGAGUGAGCAC | UAUUUCACUCUCUUCUGUUG | Cleavage |  | 2 |
| sbi-miR156g | SbNFY-B5 | 3.5 | -1 | 1 | 20 | 4536 | 4555 | UGACAGAAGAGAGUGAGCAC | UAUUUCACUCUCUUCUGUUG | Cleavage |  | 2 |
| sbi-miR156h | SbNFY-B5 | 3.5 | -1 | 1 | 20 | 4536 | 4555 | UGACAGAAGAGAGUGAGCAC | UAUUUCACUCUCUUCUGUUG | Cleavage |  | 2 |
| sbi-miR156i | SbNFY-B5 | 3.5 | -1 | 1 | 20 | 4536 | 4555 | UGACAGAAGAGAGUGAGCAC | UAUUUCACUCUCUUCUGUUG | Cleavage |  | 2 |
| sbi-miR172b | SbNFY-B17 | 3.5 | -1 | 1 | 20 | 3024 | 3043 | GGAAUCUUGAUGAUGCUGCA | UGUAGCUUUGUUGAGAUUCC | Cleavage |  | 1 |
| sbi-miR2118-5p | SbNFY-B1 | 3.5 | -1 | 1 | 22 | 8449 | 8470 | GGCAUGGGAACAUGUAGGAAGG | CCGUCCCACGUGUUCCCGGGCC | Cleavage |  | 1 |
| sbi-miR395l | SbNFY-B5 | 3.5 | -1 | 1 | 21 | 5040 | 5060 | GUGAAGUGCUUGGGGGAACUC | UUGUUUUCUCAAGCAUUUUAG | Cleavage |  | 1 |
| sbi-miR437a | SbNFY-B6 | 3.5 | -1 | 1 | 21 | 3495 | 3515 | AAAGUUAGAGAAGUUUGACUU | AAGUUAAACAUCUAUAACUUU | Cleavage |  | 1 |
| sbi-miR437b | SbNFY-B6 | 3.5 | -1 | 1 | 21 | 3495 | 3515 | AAAGUUAGAGAAGUUUGACUU | AAGUUAAACAUCUAUAACUUU | Cleavage |  | 1 |
| sbi-miR437c | SbNFY-B6 | 3.5 | -1 | 1 | 21 | 3495 | 3515 | AAAGUUAGAGAAGUUUGACUU | AAGUUAAACAUCUAUAACUUU | Cleavage |  | 1 |
| sbi-miR437d | SbNFY-B6 | 3.5 | -1 | 1 | 21 | 3495 | 3515 | AAAGUUAGAGAAGUUUGACUU | AAGUUAAACAUCUAUAACUUU | Cleavage |  | 1 |
| sbi-miR437e | SbNFY-B6 | 3.5 | -1 | 1 | 21 | 3495 | 3515 | AAAGUUAGAGAAGUUUGACUU | AAGUUAAACAUCUAUAACUUU | Cleavage |  | 1 |
| sbi-miR437f | SbNFY-B6 | 3.5 | -1 | 1 | 21 | 3495 | 3515 | AAAGUUAGAGAAGUUUGACUU | AAGUUAAACAUCUAUAACUUU | Cleavage |  | 1 |
| sbi-miR437g | SbNFY-B6 | 3.5 | -1 | 1 | 21 | 3495 | 3515 | AAAGUUAGAGAAGUUUGACUU | AAGUUAAACAUCUAUAACUUU | Cleavage |  | 1 |
| sbi-miR437i | SbNFY-B6 | 3.5 | -1 | 1 | 21 | 3495 | 3515 | AAAGUUAGAGAAGUUUGACUU | AAGUUAAACAUCUAUAACUUU | Cleavage |  | 1 |
| sbi-miR437j | SbNFY-B6 | 3.5 | -1 | 1 | 21 | 3495 | 3515 | AAAGUUAGAGAAGUUUGACUU | AAGUUAAACAUCUAUAACUUU | Cleavage |  | 1 |
| sbi-miR437k | SbNFY-B6 | 3.5 | -1 | 1 | 21 | 3495 | 3515 | AAAGUUAGAGAAGUUUGACUU | AAGUUAAACAUCUAUAACUUU | Cleavage |  | 1 |
| sbi-miR437l | SbNFY-B6 | 3.5 | -1 | 1 | 21 | 3495 | 3515 | AAAGUUAGAGAAGUUUGACUU | AAGUUAAACAUCUAUAACUUU | Cleavage |  | 1 |
| sbi-miR437m | SbNFY-B6 | 3.5 | -1 | 1 | 21 | 3495 | 3515 | AAAGUUAGAGAAGUUUGACUU | AAGUUAAACAUCUAUAACUUU | Cleavage |  | 1 |
| sbi-miR437n | SbNFY-B6 | 3.5 | -1 | 1 | 21 | 3495 | 3515 | AAAGUUAGAGAAGUUUGACUU | AAGUUAAACAUCUAUAACUUU | Cleavage |  | 1 |
| sbi-miR437o | SbNFY-B6 | 3.5 | -1 | 1 | 21 | 3495 | 3515 | AAAGUUAGAGAAGUUUGACUU | AAGUUAAACAUCUAUAACUUU | Cleavage |  | 1 |
| sbi-miR437p | SbNFY-B6 | 3.5 | -1 | 1 | 21 | 3495 | 3515 | AAAGUUAGAGAAGUUUGACUU | AAGUUAAACAUCUAUAACUUU | Cleavage |  | 1 |
| sbi-miR437q | SbNFY-B6 | 3.5 | -1 | 1 | 21 | 3495 | 3515 | AAAGUUAGAGAAGUUUGACUU | AAGUUAAACAUCUAUAACUUU | Cleavage |  | 1 |
| sbi-miR437r | SbNFY-B6 | 3.5 | -1 | 1 | 21 | 3495 | 3515 | AAAGUUAGAGAAGUUUGACUU | AAGUUAAACAUCUAUAACUUU | Cleavage |  | 1 |
| sbi-miR437s | SbNFY-B6 | 3.5 | -1 | 1 | 21 | 3495 | 3515 | AAAGUUAGAGAAGUUUGACUU | AAGUUAAACAUCUAUAACUUU | Cleavage |  | 1 |
| sbi-miR437t | SbNFY-B6 | 3.5 | -1 | 1 | 21 | 3495 | 3515 | AAAGUUAGAGAAGUUUGACUU | AAGUUAAACAUCUAUAACUUU | Cleavage |  | 1 |
| sbi-miR437u | SbNFY-B6 | 3.5 | -1 | 1 | 21 | 3495 | 3515 | AAAGUUAGAGAAGUUUGACUU | AAGUUAAACAUCUAUAACUUU | Cleavage |  | 1 |
| sbi-miR437v | SbNFY-B6 | 3.5 | -1 | 1 | 21 | 3495 | 3515 | AAAGUUAGAGAAGUUUGACUU | AAGUUAAACAUCUAUAACUUU | Cleavage |  | 1 |
| sbi-miR437w | SbNFY-B6 | 3.5 | -1 | 1 | 21 | 3495 | 3515 | AAAGUUAGAGAAGUUUGACUU | AAGUUAAACAUCUAUAACUUU | Cleavage |  | 1 |
| sbi-miR437x-3p | SbNFY-B13 | 3.5 | -1 | 1 | 24 | 15686 | 15709 | AUUUGACUGACACGGAUUCUAGGA | AAUGUAGAUCUGUGUCAGACAAAU | Cleavage |  | 1 |
| sbi-miR437x-5p | SbNFY-B7 | 3.5 | -1 | 1 | 24 | 14394 | 14417 | UAGAGUUGUCCUAAGUCAAACUUU | AAUAUUUGAUUUAAGACAACUCCA | Translation | | 1 |
| sbi-miR5386 | SbNFY-B10 | 3.5 | -1 | 1 | 20 | 9195 | 9214 | CGUCGCUGUCGCGCGCGCUG | CGGCGCCCGCCACGGCGACG | Translation | | 3 |
| sbi-miR5565f | SbNFY-B14 | 3.5 | -1 | 1 | 20 | 17480 | 17499 | UAGUCGGAUUUAUAUCAAUC | GAUUGAGGUGAAUCCGACAA | Cleavage |  | 2 |
| sbi-miR5567 | SbNFY-B6 | 3.5 | -1 | 1 | 24 | 6047 | 6070 | UUAAUGAUUCAUGUAUGUGUCCAA | UUGGAUGUAUACAUGGAGCAUUAA | Cleavage |  | 5 |
| sbi-miR5567 | SbNFY-B6 | 3.5 | -1 | 1 | 24 | 9592 | 9615 | UUAAUGAUUCAUGUAUGUGUCCAA | UUAGACGCAUGCAUGGAGUAUUAA | Cleavage |  | 5 |
| sbi-miR5567 | SbNFY-B6 | 3.5 | -1 | 1 | 24 | 10272 | 10295 | UUAAUGAUUCAUGUAUGUGUCCAA | UUGUAUAUAUGCAUGGAGCAUUAA | Cleavage |  | 5 |
| sbi-miR5567 | SbNFY-B7 | 3.5 | -1 | 1 | 24 | 3840 | 3863 | UUAAUGAUUCAUGUAUGUGUCCAA | UUAGACGCAUGCAUGGAGUAUUAA | Cleavage |  | 4 |
| sbi-miR5567 | SbNFY-B5 | 3.5 | -1 | 1 | 24 | 2798 | 2821 | UUAAUGAUUCAUGUAUGUGUCCAA | UUAGACGUAUGCAUGAAGUAUUAA | Cleavage |  | 5 |
| sbi-miR5567 | SbNFY-B5 | 3.5 | -1 | 1 | 24 | 10795 | 10818 | UUAAUGAUUCAUGUAUGUGUCCAA | ACAUUCACAGAUAUGUAUUAUUAA | Cleavage |  | 5 |
| sbi-miR5567 | SbNFY-B8 | 3.5 | -1 | 1 | 24 | 15789 | 15812 | UUAAUGAUUCAUGUAUGUGUCCAA | UAUAGCACAUGCAUAAAGCAUUAA | Translation | | 2 |
| sbi-miR5567 | SbNFY-B4 | 3.5 | -1 | 1 | 24 | 6704 | 6727 | UUAAUGAUUCAUGUAUGUGUCCAA | UUAGACGCAUGUAUGAAAUAUUAA | Cleavage |  | 4 |
| sbi-miR5567 | SbNFY-B4 | 3.5 | -1 | 1 | 24 | 3066 | 3089 | UUAAUGAUUCAUGUAUGUGUCCAA | UUUAACGUAUGCAUGAAGUAUUAA | Cleavage |  | 4 |
| sbi-miR5567 | SbNFY-B9 | 3.5 | -1 | 1 | 24 | 4067 | 4090 | UUAAUGAUUCAUGUAUGUGUCCAA | UUAGACGCAUGCAUGGAGUAUUAA | Cleavage |  | 5 |
| sbi-miR5567 | SbNFY-B18 | 3.5 | -1 | 1 | 24 | 15986 | 16009 | UUAAUGAUUCAUGUAUGUGUCCAA | UUAGACACAUACAUGAAGCAAUGA | Cleavage |  | 7 |
| sbi-miR5567 | SbNFY-B18 | 3.5 | -1 | 1 | 24 | 16752 | 16775 | UUAAUGAUUCAUGUAUGUGUCCAA | UACGGCACAUGCAUGAAGCACUAA | Cleavage |  | 7 |
| sbi-miR5567 | SbNFY-B11 | 3.5 | -1 | 1 | 24 | 7023 | 7046 | UUAAUGAUUCAUGUAUGUGUCCAA | UACGGUACAUGCAUGGAGUAUUAA | Cleavage |  | 1 |
| sbi-miR5568b-5p | SbNFY-B10 | 3.5 | -1 | 1 | 21 | 620 | 640 | UUUCUAGGUACAUAGCUUUUG | CCAAGUCUAUAUAUCUAGAAA | Translation | | 1 |
| sbi-miR5568e-3p | SbNFY-B10 | 3.5 | -1 | 1 | 21 | 7714 | 7734 | UAUCUAGAAAAGCUAAAACGU | AUGUUUCGGUCUUUCUAGAUA | Translation | | 1 |
| sbi-miR5568f-3p | SbNFY-B6 | 3.5 | -1 | 1 | 21 | 3458 | 3478 | GUCUUAUAAUUUGGAAUGGAG | CUCCAUUCCAAACUGUAAGUC | Cleavage |  | 2 |
| sbi-miR5568f-3p | SbNFY-B4 | 3.5 | -1 | 1 | 21 | 11585 | 11605 | GUCUUAUAAUUUGGAAUGGAG | CUCCAUCCCAAAUUAUAAGUU | Cleavage |  | 2 |
| sbi-miR5568g-3p | SbNFY-B12 | 3.5 | -1 | 1 | 21 | 14069 | 14089 | AAAACGUCUUAUAAUUUGGAG | UUUCAAAUUAUAAGUCGCUUU | Cleavage |  | 2 |
| sbi-miR5568g-3p | SbNFY-B3 | 3.5 | -1 | 1 | 21 | 9976 | 9996 | AAAACGUCUUAUAAUUUGGAG | UUUCAAAUUAUAAGCAGUUUU | Cleavage |  | 1 |
| sbi-miR5568g-3p | SbNFY-B9 | 3.5 | -1 | 1 | 21 | 15662 | 15682 | AAAACGUCUUAUAAUUUGGAG | UACCAAAUUAUGAGAUCUUUC | Cleavage |  | 1 |
| sbi-miR5568g-5p | SbNFY-B5 | 3.5 | -1 | 1 | 21 | 14352 | 14372 | CAAAUUAUAAGAUGUUUUGGC | GCUAGAACAACUUAUAAUUUU | Cleavage |  | 2 |
| sbi-miR5568g-5p | SbNFY-B19 | 3.5 | -1 | 1 | 21 | 17554 | 17574 | CAAAUUAUAAGAUGUUUUGGC | UCUGAAAAAUUUUAUAAUUUU | Cleavage |  | 3 |
| sbi-miR5568g-5p | SbNFY-B19 | 3.5 | -1 | 1 | 21 | 16828 | 16848 | CAAAUUAUAAGAUGUUUUGGC | AUCUAAAAAUUUUAUAAUUUU | Cleavage |  | 3 |
| sbi-miR5568g-5p | SbNFY-B4 | 3.5 | -1 | 1 | 21 | 11805 | 11825 | CAAAUUAUAAGAUGUUUUGGC | UUUAGAAUGACUUAUAAUUUG | Cleavage |  | 2 |
| sbi-miR5568g-5p | SbNFY-B8 | 3.5 | -1 | 1 | 21 | 12376 | 12396 | CAAAUUAUAAGAUGUUUUGGC | GAUAAAAUAAUUUAUAGUUUG | Cleavage |  | 3 |
| sbi-miR5569 | SbNFY-B11 | 3.5 | -1 | 1 | 24 | 16433 | 16456 | UAUUGCAUGCUUGAACUAUGGUAA | UUACUAUAGUUCAAGCGUAGAAUA | Cleavage |  | 1 |
| sbi-miR6220-5p | SbNFY-B3 | 3.5 | -1 | 1 | 24 | 7471 | 7494 | CUCCAUCCUAAAUUAUAAGACAUU | AAUAGCUUAUAAUUUGGAAUAGAG | Cleavage |  | 3 |
| sbi-miR6220-5p | SbNFY-B10 | 3.5 | -1 | 1 | 24 | 7811 | 7834 | CUCCAUCCUAAAUUAUAAGACAUU | AGCAUCUUAUAAUUUGGAACGGAG | Cleavage |  | 1 |
| sbi-miR6220-5p | SbNFY-B12 | 3.5 | -1 | 1 | 24 | 14151 | 14174 | CUCCAUCCUAAAUUAUAAGACAUU | ACCAACUUAUAAUUUGGAAAGGAG | Cleavage |  | 1 |
| sbi-miR6224a-5p | SbNFY-B15 | 3.5 | -1 | 1 | 21 | 10941 | 10961 | CUCCGUCCUAAUAUAUAAGGC | UGUUUAUUUAUUGGGAUGGAA | Cleavage |  | 1 |
| sbi-miR6224b-5p | SbNFY-B15 | 3.5 | -1 | 1 | 21 | 10941 | 10961 | CUCCGUCCUAAUAUAUAAGGC | UGUUUAUUUAUUGGGAUGGAA | Cleavage |  | 1 |
| sbi-miR6224c-5p | SbNFY-B15 | 3.5 | -1 | 1 | 21 | 10941 | 10961 | CUCCGUCCUAAUAUAUAAGGC | UGUUUAUUUAUUGGGAUGGAA | Cleavage |  | 1 |
| sbi-miR6225-3p | SbNFY-B19 | 3.5 | -1 | 1 | 24 | 17209 | 17232 | GAAACGAAUCUUUUAAGUCUAAUU | AACUAGGUUCAAAAGAUUCGUCUC | Cleavage |  | 4 |
| sbi-miR6225-3p | SbNFY-B12 | 3.5 | -1 | 1 | 24 | 9017 | 9040 | GAAACGAAUCUUUUAAGUCUAAUU | AACUAGUCUCAAAAGAUUCGUCUC | Cleavage |  | 1 |
| sbi-miR6227-5p | SbNFY-B15 | 3.5 | -1 | 1 | 24 | 10870 | 10893 | GGGCCCAAAUAGCAAGUGUUGUGA | UCUUGACAGUUGCUAUUUUGGUCU | Cleavage |  | 1 |
| sbi-miR6230-3p | SbNFY-B15 | 3.5 | -1 | 1 | 21 | 6975 | 6995 | UAACAAGUUUAGGGAUCUAGA | AAGAGACCCCUAAACUUGUCA | Cleavage |  | 1 |
| sbi-miR6230-5p | SbNFY-B11 | 3.5 | -1 | 1 | 21 | 6164 | 6184 | UUUUGGGUCCCUAAACUUGUU | AUGAAGCUUGGGGGCCCAAGA | Cleavage |  | 1 |
| sbi-miR6232b-5p | SbNFY-B10 | 3.5 | -1 | 1 | 21 | 13368 | 13388 | UUUUUGGUACAUUGAAUUUGC | AAAGAUUCGAUGUAACGGAAA | Cleavage |  | 4 |
| sbi-miR6233-5p | SbNFY-B5 | 3.5 | -1 | 1 | 24 | 10914 | 10937 | UGUUGAGGCUGGAGCGAAACUCGG | UUGGCUUUCCCUCCAGCCUGAAUG | Cleavage |  | 2 |
| sbi-miR6235-3p | SbNFY-B7 | 3.5 | -1 | 1 | 24 | 4143 | 4166 | AACGAACAGUAUUUUUCUCUUACA | AAAACAACGAAAAUACUGUUCGUA | Cleavage |  | 1 |
| sbi-miR821b | SbNFY-B17 | 3.5 | -1 | 1 | 21 | 16253 | 16273 | AAGUUAUGAACAUAAAAGUUG | AAACUUUGAUGUUUACAAUUU | Cleavage |  | 1 |
| sbi-miR1435a | SbNFY-B7 | 4 | -1 | 1 | 20 | 12352 | 12371 | UUUCUUAAGUCAAACUUUUC | GAAAUUUUUUACUUAAGGAA | Translation | | 2 |
| sbi-miR1435a | SbNFY-B7 | 4 | -1 | 1 | 20 | 14392 | 14411 | UUUCUUAAGUCAAACUUUUC | AAAAUAUUUGAUUUAAGACA | Cleavage |  | 2 |
| sbi-miR1435b | SbNFY-B1 | 4 | -1 | 1 | 20 | 14501 | 14520 | UUUCUUAAGUCAAACCUUUU | AAAAGUGUUGGCUUCAGAAA | Cleavage |  | 1 |
| sbi-miR1435b | SbNFY-B7 | 4 | -1 | 1 | 20 | 14392 | 14411 | UUUCUUAAGUCAAACCUUUU | AAAAUAUUUGAUUUAAGACA | Cleavage |  | 2 |
| sbi-miR1435b | SbNFY-B7 | 4 | -1 | 1 | 20 | 12352 | 12371 | UUUCUUAAGUCAAACCUUUU | GAAAUUUUUUACUUAAGGAA | Translation | | 2 |
| sbi-miR156a | SbNFY-B16 | 4 | -1 | 1 | 20 | 17641 | 17660 | UGACAGAAGAGAGUGAGCAC | UUCUUCAUUCUCUUUUGUUU | Cleavage |  | 1 |
| sbi-miR156b | SbNFY-B16 | 4 | -1 | 1 | 20 | 17641 | 17660 | UGACAGAAGAGAGUGAGCAC | UUCUUCAUUCUCUUUUGUUU | Cleavage |  | 1 |
| sbi-miR156c | SbNFY-B16 | 4 | -1 | 1 | 20 | 17641 | 17660 | UGACAGAAGAGAGUGAGCAC | UUCUUCAUUCUCUUUUGUUU | Cleavage |  | 1 |
| sbi-miR156d | SbNFY-B5 | 4 | -1 | 1 | 21 | 5394 | 5414 | UGACAGAAGAGAGAGAGCACA | GAUCCUUUUUUUUUUCUGUCU | Cleavage |  | 2 |
| sbi-miR156f | SbNFY-B16 | 4 | -1 | 1 | 20 | 17641 | 17660 | UGACAGAAGAGAGUGAGCAC | UUCUUCAUUCUCUUUUGUUU | Cleavage |  | 1 |
| sbi-miR156g | SbNFY-B16 | 4 | -1 | 1 | 20 | 17641 | 17660 | UGACAGAAGAGAGUGAGCAC | UUCUUCAUUCUCUUUUGUUU | Cleavage |  | 1 |
| sbi-miR156h | SbNFY-B16 | 4 | -1 | 1 | 20 | 17641 | 17660 | UGACAGAAGAGAGUGAGCAC | UUCUUCAUUCUCUUUUGUUU | Cleavage |  | 1 |
| sbi-miR156i | SbNFY-B16 | 4 | -1 | 1 | 20 | 17641 | 17660 | UGACAGAAGAGAGUGAGCAC | UUCUUCAUUCUCUUUUGUUU | Cleavage |  | 1 |
| sbi-miR159a | SbNFY-B2 | 4 | -1 | 1 | 21 | 10321 | 10341 | UUUGGAUUGAAGGGAGCUCUG | AAAAGCUGCCUACAAUUCAAA | Translation | | 2 |
| sbi-miR159b | SbNFY-B6 | 4 | -1 | 1 | 21 | 13400 | 13420 | CUUGGAUUGAAGGGAGCUCCU | UUGAGCUCCUUACAGUACAAG | Translation | | 1 |
| sbi-miR164a | SbNFY-B10 | 4 | -1 | 1 | 21 | 3270 | 3290 | UGGAGAAGCAGGGCACGUGCA | UCCUCGUGCACUGCUUUUCCU | Cleavage |  | 1 |
| sbi-miR164b | SbNFY-B10 | 4 | -1 | 1 | 21 | 3270 | 3290 | UGGAGAAGCAGGGCACGUGCU | UCCUCGUGCACUGCUUUUCCU | Cleavage |  | 1 |
| sbi-miR164d | SbNFY-B10 | 4 | -1 | 1 | 21 | 3270 | 3290 | UGGAGAAGCAGGGCACGUGCA | UCCUCGUGCACUGCUUUUCCU | Cleavage |  | 1 |
| sbi-miR164e | SbNFY-B10 | 4 | -1 | 1 | 21 | 3270 | 3290 | UGGAGAAGCAGGGCACGUGCA | UCCUCGUGCACUGCUUUUCCU | Cleavage |  | 1 |
| sbi-miR169p | SbNFY-B4 | 4 | -1 | 1 | 21 | 8189 | 8209 | UAGCCAAGAAUGGCUUGCCUA | UAGGCAGGCCAUGCUUCGCUG | Cleavage |  | 1 |
| sbi-miR169q | SbNFY-B4 | 4 | -1 | 1 | 21 | 8189 | 8209 | UAGCCAAGAAUGGCUUGCCUA | UAGGCAGGCCAUGCUUCGCUG | Cleavage |  | 1 |
| sbi-miR172a | SbNFY-B13 | 4 | -1 | 1 | 20 | 1904 | 1923 | AGAAUCUUGAUGAUGCUGCA | CGCAGCUUUGUCAAGGUACU | Cleavage |  | 2 |
| sbi-miR172c | SbNFY-B13 | 4 | -1 | 1 | 20 | 1904 | 1923 | AGAAUCUUGAUGAUGCUGCA | CGCAGCUUUGUCAAGGUACU | Cleavage |  | 2 |
| sbi-miR172d | SbNFY-B13 | 4 | -1 | 1 | 20 | 1904 | 1923 | AGAAUCUUGAUGAUGCUGCA | CGCAGCUUUGUCAAGGUACU | Cleavage |  | 2 |
| sbi-miR2118-5p | SbNFY-B2 | 4 | -1 | 1 | 22 | 12132 | 12153 | GGCAUGGGAACAUGUAGGAAGG | GCUUUUUACAUGUCCUCAUGCA | Cleavage |  | 1 |
| sbi-miR319a | SbNFY-B14 | 4 | -1 | 1 | 20 | 15750 | 15769 | UUGGACUGAAGGGUGCUCCC | UGGAGCUUCUUUCAGGUCAA | Cleavage |  | 1 |
| sbi-miR319b | SbNFY-B14 | 4 | -1 | 1 | 20 | 15750 | 15769 | UUGGACUGAAGGGUGCUCCC | UGGAGCUUCUUUCAGGUCAA | Cleavage |  | 1 |
| sbi-miR395a | SbNFY-B5 | 4 | -1 | 1 | 21 | 5040 | 5060 | GUGAAGUGUUUGGGGGAACUC | UUGUUUUCUCAAGCAUUUUAG | Cleavage |  | 3 |
| sbi-miR395b | SbNFY-B5 | 4 | -1 | 1 | 21 | 5040 | 5060 | GUGAAGUGUUUGGGGGAACUC | UUGUUUUCUCAAGCAUUUUAG | Cleavage |  | 3 |
| sbi-miR395c | SbNFY-B5 | 4 | -1 | 1 | 21 | 5040 | 5060 | GUGAAGUGUUUGGGGGAACUC | UUGUUUUCUCAAGCAUUUUAG | Cleavage |  | 3 |
| sbi-miR395d | SbNFY-B5 | 4 | -1 | 1 | 21 | 5040 | 5060 | GUGAAGUGUUUGGGGGAACUC | UUGUUUUCUCAAGCAUUUUAG | Cleavage |  | 3 |
| sbi-miR395e | SbNFY-B5 | 4 | -1 | 1 | 21 | 5040 | 5060 | GUGAAGUGUUUGGGGGAACUC | UUGUUUUCUCAAGCAUUUUAG | Cleavage |  | 3 |
| sbi-miR395f | SbNFY-B5 | 4 | -1 | 1 | 21 | 5040 | 5060 | AUGAAGUGUUUGGGGGAACUC | UUGUUUUCUCAAGCAUUUUAG | Cleavage |  | 2 |
| sbi-miR395f | SbNFY-B16 | 4 | -1 | 1 | 21 | 5314 | 5334 | AUGAAGUGUUUGGGGGAACUC | UUGUUUUACCAAACACUUUUU | Cleavage |  | 1 |
| sbi-miR395g | SbNFY-B5 | 4 | -1 | 1 | 21 | 5040 | 5060 | GUGAAGUGUUUGGGGGAACUC | UUGUUUUCUCAAGCAUUUUAG | Cleavage |  | 3 |
| sbi-miR395h | SbNFY-B5 | 4 | -1 | 1 | 21 | 5040 | 5060 | GUGAAGUGUUUGGGGGAACUC | UUGUUUUCUCAAGCAUUUUAG | Cleavage |  | 3 |
| sbi-miR395i | SbNFY-B5 | 4 | -1 | 1 | 21 | 5040 | 5060 | GUGAAGUGUUUGGGGGAACUC | UUGUUUUCUCAAGCAUUUUAG | Cleavage |  | 3 |
| sbi-miR395j | SbNFY-B5 | 4 | -1 | 1 | 21 | 5040 | 5060 | GUGAAGUGUUUGGGGGAACUC | UUGUUUUCUCAAGCAUUUUAG | Cleavage |  | 3 |
| sbi-miR408 | SbNFY-B18 | 4 | -1 | 1 | 21 | 17492 | 17512 | CUGCACUGCCUCUUCCCUGGC | GUCGGGGAAGUGGUAGUGCGU | Translation | | 1 |
| sbi-miR437a | SbNFY-B9 | 4 | -1 | 1 | 21 | 5864 | 5884 | AAAGUUAGAGAAGUUUGACUU | UUGUAAAACUUCUCUUGCUUA | Cleavage |  | 2 |
| sbi-miR437a | SbNFY-B14 | 4 | -1 | 1 | 21 | 381 | 401 | AAAGUUAGAGAAGUUUGACUU | AUGUCAAACUAUUUUAAGUUU | Translation | | 1 |
| sbi-miR437b | SbNFY-B9 | 4 | -1 | 1 | 21 | 5864 | 5884 | AAAGUUAGAGAAGUUUGACUU | UUGUAAAACUUCUCUUGCUUA | Cleavage |  | 2 |
| sbi-miR437b | SbNFY-B14 | 4 | -1 | 1 | 21 | 381 | 401 | AAAGUUAGAGAAGUUUGACUU | AUGUCAAACUAUUUUAAGUUU | Translation | | 1 |
| sbi-miR437c | SbNFY-B9 | 4 | -1 | 1 | 21 | 5864 | 5884 | AAAGUUAGAGAAGUUUGACUU | UUGUAAAACUUCUCUUGCUUA | Cleavage |  | 2 |
| sbi-miR437c | SbNFY-B14 | 4 | -1 | 1 | 21 | 381 | 401 | AAAGUUAGAGAAGUUUGACUU | AUGUCAAACUAUUUUAAGUUU | Translation | | 1 |
| sbi-miR437d | SbNFY-B9 | 4 | -1 | 1 | 21 | 5864 | 5884 | AAAGUUAGAGAAGUUUGACUU | UUGUAAAACUUCUCUUGCUUA | Cleavage |  | 2 |
| sbi-miR437d | SbNFY-B14 | 4 | -1 | 1 | 21 | 381 | 401 | AAAGUUAGAGAAGUUUGACUU | AUGUCAAACUAUUUUAAGUUU | Translation | | 1 |
| sbi-miR437e | SbNFY-B9 | 4 | -1 | 1 | 21 | 5864 | 5884 | AAAGUUAGAGAAGUUUGACUU | UUGUAAAACUUCUCUUGCUUA | Cleavage |  | 2 |
| sbi-miR437e | SbNFY-B14 | 4 | -1 | 1 | 21 | 381 | 401 | AAAGUUAGAGAAGUUUGACUU | AUGUCAAACUAUUUUAAGUUU | Translation | | 1 |
| sbi-miR437f | SbNFY-B9 | 4 | -1 | 1 | 21 | 5864 | 5884 | AAAGUUAGAGAAGUUUGACUU | UUGUAAAACUUCUCUUGCUUA | Cleavage |  | 2 |
| sbi-miR437f | SbNFY-B14 | 4 | -1 | 1 | 21 | 381 | 401 | AAAGUUAGAGAAGUUUGACUU | AUGUCAAACUAUUUUAAGUUU | Translation | | 1 |
| sbi-miR437g | SbNFY-B9 | 4 | -1 | 1 | 21 | 5864 | 5884 | AAAGUUAGAGAAGUUUGACUU | UUGUAAAACUUCUCUUGCUUA | Cleavage |  | 2 |
| sbi-miR437g | SbNFY-B14 | 4 | -1 | 1 | 21 | 381 | 401 | AAAGUUAGAGAAGUUUGACUU | AUGUCAAACUAUUUUAAGUUU | Translation | | 1 |
| sbi-miR437i | SbNFY-B9 | 4 | -1 | 1 | 21 | 5864 | 5884 | AAAGUUAGAGAAGUUUGACUU | UUGUAAAACUUCUCUUGCUUA | Cleavage |  | 2 |
| sbi-miR437i | SbNFY-B14 | 4 | -1 | 1 | 21 | 381 | 401 | AAAGUUAGAGAAGUUUGACUU | AUGUCAAACUAUUUUAAGUUU | Translation | | 1 |
| sbi-miR437j | SbNFY-B9 | 4 | -1 | 1 | 21 | 5864 | 5884 | AAAGUUAGAGAAGUUUGACUU | UUGUAAAACUUCUCUUGCUUA | Cleavage |  | 2 |
| sbi-miR437j | SbNFY-B14 | 4 | -1 | 1 | 21 | 381 | 401 | AAAGUUAGAGAAGUUUGACUU | AUGUCAAACUAUUUUAAGUUU | Translation | | 1 |
| sbi-miR437k | SbNFY-B9 | 4 | -1 | 1 | 21 | 5864 | 5884 | AAAGUUAGAGAAGUUUGACUU | UUGUAAAACUUCUCUUGCUUA | Cleavage |  | 2 |
| sbi-miR437k | SbNFY-B14 | 4 | -1 | 1 | 21 | 381 | 401 | AAAGUUAGAGAAGUUUGACUU | AUGUCAAACUAUUUUAAGUUU | Translation | | 1 |
| sbi-miR437l | SbNFY-B9 | 4 | -1 | 1 | 21 | 5864 | 5884 | AAAGUUAGAGAAGUUUGACUU | UUGUAAAACUUCUCUUGCUUA | Cleavage |  | 2 |
| sbi-miR437l | SbNFY-B14 | 4 | -1 | 1 | 21 | 381 | 401 | AAAGUUAGAGAAGUUUGACUU | AUGUCAAACUAUUUUAAGUUU | Translation | | 1 |
| sbi-miR437m | SbNFY-B9 | 4 | -1 | 1 | 21 | 5864 | 5884 | AAAGUUAGAGAAGUUUGACUU | UUGUAAAACUUCUCUUGCUUA | Cleavage |  | 2 |
| sbi-miR437m | SbNFY-B14 | 4 | -1 | 1 | 21 | 381 | 401 | AAAGUUAGAGAAGUUUGACUU | AUGUCAAACUAUUUUAAGUUU | Translation | | 1 |
| sbi-miR437n | SbNFY-B9 | 4 | -1 | 1 | 21 | 5864 | 5884 | AAAGUUAGAGAAGUUUGACUU | UUGUAAAACUUCUCUUGCUUA | Cleavage |  | 2 |
| sbi-miR437n | SbNFY-B14 | 4 | -1 | 1 | 21 | 381 | 401 | AAAGUUAGAGAAGUUUGACUU | AUGUCAAACUAUUUUAAGUUU | Translation | | 1 |
| sbi-miR437o | SbNFY-B9 | 4 | -1 | 1 | 21 | 5864 | 5884 | AAAGUUAGAGAAGUUUGACUU | UUGUAAAACUUCUCUUGCUUA | Cleavage |  | 2 |
| sbi-miR437o | SbNFY-B14 | 4 | -1 | 1 | 21 | 381 | 401 | AAAGUUAGAGAAGUUUGACUU | AUGUCAAACUAUUUUAAGUUU | Translation | | 1 |
| sbi-miR437p | SbNFY-B9 | 4 | -1 | 1 | 21 | 5864 | 5884 | AAAGUUAGAGAAGUUUGACUU | UUGUAAAACUUCUCUUGCUUA | Cleavage |  | 2 |
| sbi-miR437p | SbNFY-B14 | 4 | -1 | 1 | 21 | 381 | 401 | AAAGUUAGAGAAGUUUGACUU | AUGUCAAACUAUUUUAAGUUU | Translation | | 1 |
| sbi-miR437q | SbNFY-B9 | 4 | -1 | 1 | 21 | 5864 | 5884 | AAAGUUAGAGAAGUUUGACUU | UUGUAAAACUUCUCUUGCUUA | Cleavage |  | 2 |
| sbi-miR437q | SbNFY-B14 | 4 | -1 | 1 | 21 | 381 | 401 | AAAGUUAGAGAAGUUUGACUU | AUGUCAAACUAUUUUAAGUUU | Translation | | 1 |
| sbi-miR437r | SbNFY-B9 | 4 | -1 | 1 | 21 | 5864 | 5884 | AAAGUUAGAGAAGUUUGACUU | UUGUAAAACUUCUCUUGCUUA | Cleavage |  | 2 |
| sbi-miR437r | SbNFY-B14 | 4 | -1 | 1 | 21 | 381 | 401 | AAAGUUAGAGAAGUUUGACUU | AUGUCAAACUAUUUUAAGUUU | Translation | | 1 |
| sbi-miR437s | SbNFY-B9 | 4 | -1 | 1 | 21 | 5864 | 5884 | AAAGUUAGAGAAGUUUGACUU | UUGUAAAACUUCUCUUGCUUA | Cleavage |  | 2 |
| sbi-miR437s | SbNFY-B14 | 4 | -1 | 1 | 21 | 381 | 401 | AAAGUUAGAGAAGUUUGACUU | AUGUCAAACUAUUUUAAGUUU | Translation | | 1 |
| sbi-miR437t | SbNFY-B9 | 4 | -1 | 1 | 21 | 5864 | 5884 | AAAGUUAGAGAAGUUUGACUU | UUGUAAAACUUCUCUUGCUUA | Cleavage |  | 2 |
| sbi-miR437t | SbNFY-B14 | 4 | -1 | 1 | 21 | 381 | 401 | AAAGUUAGAGAAGUUUGACUU | AUGUCAAACUAUUUUAAGUUU | Translation | | 1 |
| sbi-miR437u | SbNFY-B9 | 4 | -1 | 1 | 21 | 5864 | 5884 | AAAGUUAGAGAAGUUUGACUU | UUGUAAAACUUCUCUUGCUUA | Cleavage |  | 2 |
| sbi-miR437u | SbNFY-B14 | 4 | -1 | 1 | 21 | 381 | 401 | AAAGUUAGAGAAGUUUGACUU | AUGUCAAACUAUUUUAAGUUU | Translation | | 1 |
| sbi-miR437v | SbNFY-B9 | 4 | -1 | 1 | 21 | 5864 | 5884 | AAAGUUAGAGAAGUUUGACUU | UUGUAAAACUUCUCUUGCUUA | Cleavage |  | 2 |
| sbi-miR437v | SbNFY-B14 | 4 | -1 | 1 | 21 | 381 | 401 | AAAGUUAGAGAAGUUUGACUU | AUGUCAAACUAUUUUAAGUUU | Translation | | 1 |
| sbi-miR437w | SbNFY-B9 | 4 | -1 | 1 | 21 | 5864 | 5884 | AAAGUUAGAGAAGUUUGACUU | UUGUAAAACUUCUCUUGCUUA | Cleavage |  | 2 |
| sbi-miR437w | SbNFY-B14 | 4 | -1 | 1 | 21 | 381 | 401 | AAAGUUAGAGAAGUUUGACUU | AUGUCAAACUAUUUUAAGUUU | Translation | | 1 |
| sbi-miR528 | SbNFY-B18 | 4 | -1 | 1 | 21 | 2136 | 2155 | UGGAAGGGGCAUGCAGAGGAG | UUUCGCU-CGUGCCCCUUCCA | Cleavage |  | 1 |
| sbi-miR5387a | SbNFY-B9 | 4 | -1 | 1 | 25 | 7738 | 7762 | UAACACGAACCGGUGCUAAAGGAUC | GGUGCUUUGACUCUGGUUUGUGUUG | Cleavage |  | 1 |
| sbi-miR5564c-3p | SbNFY-B8 | 4 | -1 | 1 | 21 | 325 | 345 | ACGCGAGCUGUUUGGCGAAUU | UUUUCGUCAAACAGUUCGUUG | Cleavage |  | 1 |
| sbi-miR5565e | SbNFY-B13 | 4 | -1 | 1 | 19 | 5963 | 5981 | UUGUUUGGAUGUUGUCGGA | GGCGAUAACAUCCAGACAC | Cleavage |  | 2 |
| sbi-miR5565e | SbNFY-B19 | 4 | -1 | 1 | 19 | 15646 | 15664 | UUGUUUGGAUGUUGUCGGA | UCCGACAACAUAUAAGAAA | Cleavage |  | 2 |
| sbi-miR5565e | SbNFY-B10 | 4 | -1 | 1 | 19 | 551 | 569 | UUGUUUGGAUGUUGUCGGA | UUCGACUAUAUCUAAAUAG | Cleavage |  | 1 |
| sbi-miR5565e | SbNFY-B8 | 4 | -1 | 1 | 19 | 12415 | 12433 | UUGUUUGGAUGUUGUCGGA | UCAUACAACAUCUAAAAAA | Cleavage |  | 1 |
| sbi-miR5567 | SbNFY-B10 | 4 | -1 | 1 | 24 | 1649 | 1672 | UUAAUGAUUCAUGUAUGUGUCCAA | UUGGACACAUGCAUGGAGCAUUUA | Cleavage |  | 6 |
| sbi-miR5567 | SbNFY-B10 | 4 | -1 | 1 | 24 | 279 | 302 | UUAAUGAUUCAUGUAUGUGUCCAA | UUUGACACAUGCAUAAAGUAUUAA | Translation | | 6 |
| sbi-miR5567 | SbNFY-B10 | 4 | -1 | 1 | 24 | 40 | 63 | UUAAUGAUUCAUGUAUGUGUCCAA | UUGAUCAUAUACAUUGAACAUUAA | Translation | | 6 |
| sbi-miR5567 | SbNFY-B15 | 4 | -1 | 1 | 24 | 4265 | 4288 | UUAAUGAUUCAUGUAUGUGUCCAA | GGCAAAAUAUAUGUGAAUUAUUGG | Cleavage |  | 2 |
| sbi-miR5568a | SbNFY-B6 | 4 | -1 | 1 | 21 | 3191 | 3211 | CAGAGCGACUUACAAUUUGGA | CCCAAAUUAUAAGACGUUUUG | Cleavage |  | 2 |
| sbi-miR5568b-3p | SbNFY-B10 | 4 | -1 | 1 | 21 | 7720 | 7740 | ACUAUGUAUCUAGAAAAGCUA | CGGUCUUUCUAGAUACAUACA | Cleavage |  | 2 |
| sbi-miR5568b-3p | SbNFY-B12 | 4 | -1 | 1 | 21 | 4994 | 5014 | ACUAUGUAUCUAGAAAAGCUA | UUAUUUAUCUAGAUGCAUAGC | Cleavage |  | 1 |
| sbi-miR5568b-5p | SbNFY-B3 | 4 | -1 | 1 | 21 | 10054 | 10074 | UUUCUAGGUACAUAGCUUUUG | UAAAAGCUCUGUACCUACAAU | Cleavage |  | 2 |
| sbi-miR5568c-3p | SbNFY-B4 | 4 | -1 | 1 | 21 | 11584 | 11604 | ACUUACAGUUUGGAACGGAGG | CCUCCAUCCCAAAUUAUAAGU | Cleavage |  | 2 |
| sbi-miR5568c-5p | SbNFY-B6 | 4 | -1 | 1 | 21 | 3302 | 3322 | UCUGUUCCAAAUUGUAAGUCG | CGUCUUAUAAUUUAGAAUGGA | Cleavage |  | 2 |
| sbi-miR5568c-5p | SbNFY-B6 | 4 | -1 | 1 | 21 | 10943 | 10963 | UCUGUUCCAAAUUGUAAGUCG | UGAGUUAGAGUUUGGGAUGGA | Cleavage |  | 2 |
| sbi-miR5568c-5p | SbNFY-B5 | 4 | -1 | 1 | 21 | 14390 | 14410 | UCUGUUCCAAAUUGUAAGUCG | AUUUUUACAAUUUAGAAUGGA | Cleavage |  | 1 |
| sbi-miR5568d-3p | SbNFY-B18 | 4 | -1 | 1 | 21 | 996 | 1016 | AAAGUUGUGUAUCUAGAAAAG | CUUUUUGAGAUCUACAAUUUU | Translation | | 2 |
| sbi-miR5568d-3p | SbNFY-B18 | 4 | -1 | 1 | 21 | 380 | 400 | AAAGUUGUGUAUCUAGAAAAG | CUUGAUGAGAUAUACAACUUU | Cleavage |  | 2 |
| sbi-miR5568d-5p | SbNFY-B10 | 4 | -1 | 1 | 21 | 625 | 645 | UGGCUUUUCUAGAUACAUAGC | UCUAUAUAUCUAGAAAAAUCU | Cleavage |  | 1 |
| sbi-miR5568e-5p | SbNFY-B2 | 4 | -1 | 1 | 21 | 12836 | 12856 | GAUGUUUUGGGUUUUCUAGAU | UGCCAGAAGACCUGAGACAUG | Cleavage |  | 2 |
| sbi-miR5568f-3p | SbNFY-B12 | 4 | -1 | 1 | 21 | 4926 | 4946 | GUCUUAUAAUUUGGAAUGGAG | ACUCUUUCCAAAUUAUAAGUU | Cleavage |  | 2 |
| sbi-miR5568f-5p | SbNFY-B6 | 4 | -1 | 1 | 21 | 10943 | 10963 | UCCAUUCCAAAUUGUAAGAUG | UGAGUUAGAGUUUGGGAUGGA | Cleavage |  | 3 |
| sbi-miR5568f-5p | SbNFY-B8 | 4 | -1 | 1 | 21 | 12383 | 12403 | UCCAUUCCAAAUUGUAAGAUG | UAAUUUAUAGUUUGGAACGGA | Cleavage |  | 1 |
| sbi-miR5568g-3p | SbNFY-B14 | 4 | -1 | 1 | 21 | 6519 | 6539 | AAAACGUCUUAUAAUUUGGAG | UUCUAAAUUAUAAGUUAUUUU | Cleavage |  | 2 |
| sbi-miR5568g-3p | SbNFY-B8 | 4 | -1 | 1 | 21 | 8660 | 8680 | AAAACGUCUUAUAAUUUGGAG | UUCAAAGUUAUAGGCUGUUUU | Cleavage |  | 1 |
| sbi-miR5568g-5p | SbNFY-B6 | 4 | -1 | 1 | 21 | 10755 | 10774 | CAAAUUAUAAGAUGUUUUGGC | GUCAAACCAUCUUA-AAUUUG | Cleavage |  | 3 |
| sbi-miR5568g-5p | SbNFY-B18 | 4 | -1 | 1 | 21 | 3447 | 3467 | CAAAUUAUAAGAUGUUUUGGC | UAUAAGGCAUCUAAUAAUUUU | Cleavage |  | 2 |
| sbi-miR5568g-5p | SbNFY-B14 | 4 | -1 | 1 | 21 | 9958 | 9978 | CAAAUUAUAAGAUGUUUUGGC | GUCAAAGCGACUUAUAAUUCG | Cleavage |  | 2 |
| sbi-miR5568g-5p | SbNFY-B17 | 4 | -1 | 1 | 21 | 14813 | 14833 | CAAAUUAUAAGAUGUUUUGGC | GUCAAAAAAUCUUUUAGUUUA | Cleavage |  | 1 |
| sbi-miR5568g-5p | SbNFY-B15 | 4 | -1 | 1 | 21 | 14907 | 14927 | CAAAUUAUAAGAUGUUUUGGC | UUCAGGACAUUUUAUAAUCUU | Cleavage |  | 1 |
| sbi-miR6217a-3p | SbNFY-B12 | 4 | -1 | 1 | 24 | 7539 | 7562 | AAAAUUAUCGUAAAUAGAGGUGGC | CGGAUAGUUCUUUAUGAUAAUUUU | Cleavage |  | 1 |
| sbi-miR6217b-3p | SbNFY-B12 | 4 | -1 | 1 | 24 | 7539 | 7562 | AAAAUUAUCGUAAAUAGAGGUGGC | CGGAUAGUUCUUUAUGAUAAUUUU | Cleavage |  | 1 |
| sbi-miR6220-3p | SbNFY-B12 | 4 | -1 | 1 | 24 | 4925 | 4948 | AUGCCUUAUAAUUUGGGAUGGAGA | UACUCUUUCCAAAUUAUAAGUUGU | Cleavage |  | 2 |
| sbi-miR6220-3p | SbNFY-B10 | 4 | -1 | 1 | 24 | 7694 | 7717 | AUGCCUUAUAAUUUGGGAUGGAGA | CCUUGAUCCCAAAUUACAAGAUGU | Cleavage |  | 3 |
| sbi-miR6220-5p | SbNFY-B5 | 4 | -1 | 1 | 24 | 14367 | 14389 | CUCCAUCCUAAAUUAUAAGACAUU | AAU-UUUUAUAAUUUAGAGUGAAG | Cleavage |  | 2 |
| sbi-miR6223-5p | SbNFY-B17 | 4 | -1 | 1 | 21 | 4608 | 4628 | UUCUUGGGAGGAGCAUGCUAG | UUAAUAUUCUCCUCCCAACAA | Cleavage |  | 1 |
| sbi-miR6224a-5p | SbNFY-B4 | 4 | -1 | 1 | 21 | 11813 | 11833 | CUCCGUCCUAAUAUAUAAGGC | GACUUAUAAUUUGGGAUGGAG | Cleavage |  | 2 |
| sbi-miR6224b-5p | SbNFY-B4 | 4 | -1 | 1 | 21 | 11813 | 11833 | CUCCGUCCUAAUAUAUAAGGC | GACUUAUAAUUUGGGAUGGAG | Cleavage |  | 2 |
| sbi-miR6224c-5p | SbNFY-B4 | 4 | -1 | 1 | 21 | 11813 | 11833 | CUCCGUCCUAAUAUAUAAGGC | GACUUAUAAUUUGGGAUGGAG | Cleavage |  | 2 |
| sbi-miR6225-3p | SbNFY-B3 | 4 | -1 | 1 | 24 | 11742 | 11765 | GAAACGAAUCUUUUAAGUCUAAUU | AACUAGGAUCAAAAGAUUCGUCUC | Cleavage |  | 2 |
| sbi-miR6225-3p | SbNFY-B4 | 4 | -1 | 1 | 24 | 14964 | 14987 | GAAACGAAUCUUUUAAGUCUAAUU | AACUAGGAUCAAAAGAUUCGUCUC | Cleavage |  | 7 |
| sbi-miR6225-3p | SbNFY-B4 | 4 | -1 | 1 | 24 | 152 | 175 | GAAACGAAUCUUUUAAGUCUAAUU | AACUAGGAUCAAAAGAUUCGUCUC | Cleavage |  | 7 |
| sbi-miR6225-3p | SbNFY-B6 | 4 | -1 | 1 | 24 | 16144 | 16167 | GAAACGAAUCUUUUAAGUCUAAUU | AAUUAGGGUCAAAAGAUUCGUCUC | Cleavage |  | 3 |
| sbi-miR6225-3p | SbNFY-B10 | 4 | -1 | 1 | 24 | 15668 | 15691 | GAAACGAAUCUUUUAAGUCUAAUU | AACUAGGUUUAAAAGAUUCAUAUC | Cleavage |  | 4 |
| sbi-miR6225-3p | SbNFY-B10 | 4 | -1 | 1 | 24 | 15786 | 15809 | GAAACGAAUCUUUUAAGUCUAAUU | AACUAGGUUUAAAAGAUUCAUCUC | Cleavage |  | 4 |
| sbi-miR6225-3p | SbNFY-B10 | 4 | -1 | 1 | 24 | 13275 | 13298 | GAAACGAAUCUUUUAAGUCUAAUU | AUCUAAGCUCAAAAGAUUCGUCUC | Cleavage |  | 4 |
| sbi-miR6225-5p | SbNFY-B7 | 4 | -1 | 1 | 24 | 3911 | 3934 | AACUAGACUCAAAAGAUUCAUCUC | GAGACAAAUCUUUUAAGCCUAGUU | Translation | | 2 |
| sbi-miR6225-5p | SbNFY-B18 | 4 | -1 | 1 | 24 | 16050 | 16073 | AACUAGACUCAAAAGAUUCAUCUC | GAGACGAAGCUUUUGAGCCUAAUU | Cleavage |  | 3 |
| sbi-miR6225-5p | SbNFY-B18 | 4 | -1 | 1 | 24 | 15562 | 15585 | AACUAGACUCAAAAGAUUCAUCUC | GAAACAAAUCUUUUAAGCCUAGUU | Translation | | 3 |
| sbi-miR6227-5p | SbNFY-B9 | 4 | -1 | 1 | 24 | 17287 | 17310 | GGGCCCAAAUAGCAAGUGUUGUGA | CGAGGAAAUUUGUUAUUUGGGUUA | Cleavage |  | 1 |
| sbi-miR6228-5p | SbNFY-B15 | 4 | -1 | 1 | 24 | 4758 | 4781 | UUCUAUCUCUAUUAAUUGUGUUGC | AUGGAAUAUUAAAUAUAGAUAGAA | Cleavage |  | 1 |
| sbi-miR6230-5p | SbNFY-B9 | 4 | -1 | 1 | 21 | 2641 | 2661 | UUUUGGGUCCCUAAACUUGUU | ACGAAGUUUGGGUGCCCAAAG | Cleavage |  | 1 |
| sbi-miR6230-5p | SbNFY-B8 | 4 | -1 | 1 | 21 | 17578 | 17598 | UUUUGGGUCCCUAAACUUGUU | ACGAAGUUUGGGUGCCCAAAG | Cleavage |  | 1 |
| sbi-miR6232a-5p | SbNFY-B8 | 4 | -1 | 1 | 24 | 2528 | 2550 | GUCGCUUUGACUUUUUUGGUACAU | AUG-GCCCAGACAGUCAAGGCGGC | Cleavage |  | 3 |
| sbi-miR6232a-5p | SbNFY-B19 | 4 | -1 | 1 | 24 | 16152 | 16175 | GUCGCUUUGACUUUUUUGGUACAU | GAUUCCUAAAAAAAUCAGAGGGAC | Translation | | 2 |
| sbi-miR6232b-5p | SbNFY-B6 | 4 | -1 | 1 | 21 | 13432 | 13452 | UUUUUGGUACAUUGAAUUUGC | UCCAAUUCAGUGUGUUGAGAA | Cleavage |  | 5 |
| sbi-miR6232b-5p | SbNFY-B6 | 4 | -1 | 1 | 21 | 16236 | 16256 | UUUUUGGUACAUUGAAUUUGC | UAAGAUUCGAUGUGACAGAGA | Cleavage |  | 5 |
| sbi-miR6232b-5p | SbNFY-B4 | 4 | -1 | 1 | 21 | 246 | 266 | UUUUUGGUACAUUGAAUUUGC | AAAGAUUCGAUGUGACAAGGA | Cleavage |  | 4 |
| sbi-miR6233-3p | SbNFY-B17 | 4 | -1 | 1 | 24 | 16210 | 16233 | CAAGUUUGGUUUUGGUAAUUAAUG | GAGAAAUGAUCAAAAUAAAAUUUG | Cleavage |  | 3 |
| sbi-miR6233-3p | SbNFY-B6 | 4 | -1 | 1 | 24 | 10150 | 10172 | CAAGUUUGGUUUUGGUAAUUAAUG | CUACAGUUAA-AAGACCAAACUUG | Cleavage |  | 1 |
| sbi-miR6235-3p | SbNFY-B8 | 4 | -1 | 1 | 24 | 1573 | 1596 | AACGAACAGUAUUUUUCUCUUACA | UUAUAAUUGAAAAUAUUGUUCGUU | Cleavage |  | 1 |
| sbi-miR6235-5p | SbNFY-B17 | 4 | -1 | 1 | 24 | 15051 | 15074 | UUGUGAGAGAAAAAUACUGUUGGC | ACGAAUAAUAUUUUUAUUUCAUAA | Cleavage |  | 2 |
| sbi-miR6235-5p | SbNFY-B17 | 4 | -1 | 1 | 24 | 16791 | 16814 | UUGUGAGAGAAAAAUACUGUUGGC | GUAACUGGUAUUUCUCUCUCAUAC | Translation | | 2 |
| sbi-miR821a | SbNFY-B17 | 4 | -1 | 1 | 21 | 17031 | 17051 | AAGUCAUCAACAUAAAAGUUG | UGACUUUUAUUUUGGUCAUUU | Translation | | 1 |
| sbi-miR821b | SbNFY-B7 | 4 | -1 | 1 | 21 | 17096 | 17116 | AAGUUAUGAACAUAAAAGUUG | UAGUUUUUGUGUUCAUCACUC | Cleavage |  | 2 |
| sbi-miR821b | SbNFY-B11 | 4 | -1 | 1 | 21 | 7665 | 7685 | AAGUUAUGAACAUAAAAGUUG | UCAUAUUUGUUUUCAUAAUUU | Translation | | 1 |
| sbi-miR821c | SbNFY-B17 | 4 | -1 | 1 | 21 | 17031 | 17051 | AAGUCAUCAACAUAAAAGUUG | UGACUUUUAUUUUGGUCAUUU | Translation | | 1 |
| sbi-miR821e | SbNFY-B10 | 4 | -1 | 1 | 21 | 16296 | 16316 | AAGUCAUCAAAAUAAAAGUUG | AAGCUCUCAUUAUGAUGACUU | Translation | | 2 |
| sbi-miR1432 | SbNFY-B3 | 4.5 | -1 | 1 | 21 | 2865 | 2885 | CUCAGGAGAGAUGACACCGAC | CUUGGUGGUGACUUUCCUGAG | Translation | | 1 |
| sbi-miR1435a | SbNFY-B9 | 4.5 | -1 | 1 | 20 | 2512 | 2531 | UUUCUUAAGUCAAACUUUUC | UAGUAGCUUGACUUAGGCAA | Cleavage |  | 2 |
| sbi-miR1435a | SbNFY-B16 | 4.5 | -1 | 1 | 20 | 13165 | 13184 | UUUCUUAAGUCAAACUUUUC | AAAAAUUUUGAUUACAGAAA | Cleavage |  | 1 |
| sbi-miR1435a | SbNFY-B11 | 4.5 | -1 | 1 | 20 | 10749 | 10768 | UUUCUUAAGUCAAACUUUUC | CAAAAUUUUGAGUUAACAGA | Cleavage |  | 1 |
| sbi-miR1435a | SbNFY-B6 | 4.5 | -1 | 1 | 20 | 748 | 767 | UUUCUUAAGUCAAACUUUUC | AACAUGGUUGACUGAAGAAA | Cleavage |  | 1 |
| sbi-miR1435a | SbNFY-B8 | 4.5 | -1 | 1 | 20 | 17449 | 17468 | UUUCUUAAGUCAAACUUUUC | UAGUAGCUUGACUUAGGCAA | Cleavage |  | 1 |
| sbi-miR1435b | SbNFY-B9 | 4.5 | -1 | 1 | 20 | 12321 | 12340 | UUUCUUAAGUCAAACCUUUU | GAAAGAUUUGAUUUGAUGAG | Cleavage |  | 1 |
| sbi-miR1435b | SbNFY-B6 | 4.5 | -1 | 1 | 20 | 748 | 767 | UUUCUUAAGUCAAACCUUUU | AACAUGGUUGACUGAAGAAA | Cleavage |  | 1 |
| sbi-miR156d | SbNFY-B7 | 4.5 | -1 | 1 | 21 | 13849 | 13869 | UGACAGAAGAGAGAGAGCACA | UGUGUCUUUUUUCUUUUGUCC | Cleavage |  | 1 |
| sbi-miR156d | SbNFY-B5 | 4.5 | -1 | 1 | 21 | 4535 | 4555 | UGACAGAAGAGAGAGAGCACA | GUAUUUCACUCUCUUCUGUUG | Cleavage |  | 2 |
| sbi-miR156d | SbNFY-B10 | 4.5 | -1 | 1 | 21 | 2035 | 2055 | UGACAGAAGAGAGAGAGCACA | GUGGCUUUUUCUUUGCUGUCG | Cleavage |  | 2 |
| sbi-miR156e | SbNFY-B5 | 4.5 | -1 | 1 | 20 | 4536 | 4555 | UGACAGAAGAGAGCGAGCAC | UAUUUCACUCUCUUCUGUUG | Cleavage |  | 2 |
| sbi-miR159a | SbNFY-B14 | 4.5 | -1 | 1 | 21 | 15750 | 15770 | UUUGGAUUGAAGGGAGCUCUG | UGGAGCUUCUUUCAGGUCAAU | Cleavage |  | 2 |
| sbi-miR159a | SbNFY-B13 | 4.5 | -1 | 1 | 21 | 12631 | 12651 | UUUGGAUUGAAGGGAGCUCUG | UAGGUCUUCUUUCAAUUGAAA | Cleavage |  | 1 |
| sbi-miR159a | SbNFY-B2 | 4.5 | -1 | 1 | 21 | 2208 | 2228 | UUUGGAUUGAAGGGAGCUCUG | AGAAGCUUCCAUCAAACCAAA | Translation | | 2 |
| sbi-miR159a | SbNFY-B6 | 4.5 | -1 | 1 | 21 | 13400 | 13420 | UUUGGAUUGAAGGGAGCUCUG | UUGAGCUCCUUACAGUACAAG | Translation | | 1 |
| sbi-miR159b | SbNFY-B14 | 4.5 | -1 | 1 | 21 | 15750 | 15770 | CUUGGAUUGAAGGGAGCUCCU | UGGAGCUUCUUUCAGGUCAAU | Cleavage |  | 1 |
| sbi-miR159b | SbNFY-B18 | 4.5 | -1 | 1 | 21 | 46 | 66 | CUUGGAUUGAAGGGAGCUCCU | UUGAGUUUUUUUCCAUUUAAG | Cleavage |  | 1 |
| sbi-miR166e | SbNFY-B3 | 4.5 | -1 | 1 | 21 | 11282 | 11302 | UCGGACCAGGCUUCAAUCCCU | UUGCAUGGGGGCUUGGUUUGA | Cleavage |  | 1 |
| sbi-miR166g | SbNFY-B3 | 4.5 | -1 | 1 | 21 | 11282 | 11302 | UCGGACCAGGCUUCAAUCCCU | UUGCAUGGGGGCUUGGUUUGA | Cleavage |  | 1 |
| sbi-miR169d-3p | SbNFY-B13 | 4.5 | -1 | 1 | 20 | 3027 | 3046 | GGGCGGUCACCUUGGCUAGC | ACUAGUCAAAGUGACUUCCU | Translation | | 1 |
| sbi-miR172a | SbNFY-B17 | 4.5 | -1 | 1 | 20 | 3024 | 3043 | AGAAUCUUGAUGAUGCUGCA | UGUAGCUUUGUUGAGAUUCC | Cleavage |  | 1 |
| sbi-miR172b | SbNFY-B13 | 4.5 | -1 | 1 | 20 | 1904 | 1923 | GGAAUCUUGAUGAUGCUGCA | CGCAGCUUUGUCAAGGUACU | Cleavage |  | 1 |
| sbi-miR172c | SbNFY-B17 | 4.5 | -1 | 1 | 20 | 3024 | 3043 | AGAAUCUUGAUGAUGCUGCA | UGUAGCUUUGUUGAGAUUCC | Cleavage |  | 1 |
| sbi-miR172d | SbNFY-B17 | 4.5 | -1 | 1 | 20 | 3024 | 3043 | AGAAUCUUGAUGAUGCUGCA | UGUAGCUUUGUUGAGAUUCC | Cleavage |  | 1 |
| sbi-miR172e | SbNFY-B17 | 4.5 | -1 | 1 | 21 | 3023 | 3043 | UGAAUCUUGAUGAUGCUGCAC | GUGUAGCUUUGUUGAGAUUCC | Cleavage |  | 1 |
| sbi-miR172e | SbNFY-B7 | 4.5 | -1 | 1 | 21 | 8904 | 8924 | UGAAUCUUGAUGAUGCUGCAC | UAGCACGAUGAUCAGGAUUUA | Cleavage |  | 1 |
| sbi-miR172f | SbNFY-B13 | 4.5 | -1 | 1 | 21 | 664 | 684 | AGAAUCCUGAUGAUGCUGCAC | AGACAGCAUCAGCUGGAUUUU | Translation | | 1 |
| sbi-miR390 | SbNFY-B3 | 4.5 | -1 | 1 | 21 | 1613 | 1633 | AAGCUCAGGAGGGAUAGCGCC | GGCUCUCUUCCUGUUGAGCUU | Cleavage |  | 1 |
| sbi-miR390 | SbNFY-B12 | 4.5 | -1 | 1 | 21 | 7612 | 7632 | AAGCUCAGGAGGGAUAGCGCC | CUUGCUAGCGCUACUGAGCUU | Cleavage |  | 1 |
| sbi-miR395f | SbNFY-B18 | 4.5 | -1 | 1 | 21 | 7084 | 7104 | AUGAAGUGUUUGGGGGAACUC | AAGUUCCUCCAAAAAAUUUGU | Cleavage |  | 2 |
| sbi-miR395k | SbNFY-B18 | 4.5 | -1 | 1 | 21 | 7084 | 7104 | GUGAAGUGUUUGGAGGAACUC | AAGUUCCUCCAAAAAAUUUGU | Cleavage |  | 2 |
| sbi-miR395k | SbNFY-B9 | 4.5 | -1 | 1 | 21 | 877 | 897 | GUGAAGUGUUUGGAGGAACUC | GCGGUUCUUCGAGUACUUCAG | Cleavage |  | 1 |
| sbi-miR395l | SbNFY-B9 | 4.5 | -1 | 1 | 21 | 877 | 897 | GUGAAGUGCUUGGGGGAACUC | GCGGUUCUUCGAGUACUUCAG | Cleavage |  | 1 |
| sbi-miR396a | SbNFY-B11 | 4.5 | -1 | 1 | 21 | 8834 | 8854 | UUCCACAGCUUUCUUGAACUG | GCGUGUGAGAGAGUUUUGGAA | Cleavage |  | 1 |
| sbi-miR396b | SbNFY-B11 | 4.5 | -1 | 1 | 21 | 8834 | 8854 | UUCCACAGCUUUCUUGAACUG | GCGUGUGAGAGAGUUUUGGAA | Cleavage |  | 1 |
| sbi-miR396c | SbNFY-B11 | 4.5 | -1 | 1 | 21 | 8834 | 8854 | UUCCACAGCUUUCUUGAACUU | GCGUGUGAGAGAGUUUUGGAA | Cleavage |  | 1 |
| sbi-miR397-3p | SbNFY-B3 | 4.5 | -1 | 1 | 21 | 10396 | 10416 | UCACCGGCGCUGCACUCAAUU | AUUUGAUUGAGGCGCCGUUGA | Cleavage |  | 1 |
| sbi-miR397-5p | SbNFY-B2 | 4.5 | -1 | 1 | 21 | 8401 | 8421 | UCAUUGAGUGCAGCGUUGAUG | AAUCAAGGCUGCAAGUAAUGA | Cleavage |  | 1 |
| sbi-miR397-5p | SbNFY-B14 | 4.5 | -1 | 1 | 21 | 462 | 482 | UCAUUGAGUGCAGCGUUGAUG | UCUUCAUGGUGUAUUCAAUGA | Cleavage |  | 1 |
| sbi-miR399e | SbNFY-B3 | 4.5 | -1 | 1 | 21 | 7560 | 7580 | UGCCAAAGGAGAUUUGCCCAG | ACUAUUAGAUCUCUUUUGGCA | Cleavage |  | 1 |
| sbi-miR399f | SbNFY-B3 | 4.5 | -1 | 1 | 21 | 7560 | 7580 | UGCCAAAGGAGAUUUGCCCAG | ACUAUUAGAUCUCUUUUGGCA | Cleavage |  | 1 |
| sbi-miR437a | SbNFY-B10 | 4.5 | -1 | 1 | 21 | 12917 | 12937 | AAAGUUAGAGAAGUUUGACUU | AGAUCUAAUUUUUUUGAUUUU | Cleavage |  | 1 |
| sbi-miR437b | SbNFY-B10 | 4.5 | -1 | 1 | 21 | 12917 | 12937 | AAAGUUAGAGAAGUUUGACUU | AGAUCUAAUUUUUUUGAUUUU | Cleavage |  | 1 |
| sbi-miR437c | SbNFY-B10 | 4.5 | -1 | 1 | 21 | 12917 | 12937 | AAAGUUAGAGAAGUUUGACUU | AGAUCUAAUUUUUUUGAUUUU | Cleavage |  | 1 |
| sbi-miR437d | SbNFY-B10 | 4.5 | -1 | 1 | 21 | 12917 | 12937 | AAAGUUAGAGAAGUUUGACUU | AGAUCUAAUUUUUUUGAUUUU | Cleavage |  | 1 |
| sbi-miR437e | SbNFY-B10 | 4.5 | -1 | 1 | 21 | 12917 | 12937 | AAAGUUAGAGAAGUUUGACUU | AGAUCUAAUUUUUUUGAUUUU | Cleavage |  | 1 |
| sbi-miR437f | SbNFY-B10 | 4.5 | -1 | 1 | 21 | 12917 | 12937 | AAAGUUAGAGAAGUUUGACUU | AGAUCUAAUUUUUUUGAUUUU | Cleavage |  | 1 |
| sbi-miR437g | SbNFY-B10 | 4.5 | -1 | 1 | 21 | 12917 | 12937 | AAAGUUAGAGAAGUUUGACUU | AGAUCUAAUUUUUUUGAUUUU | Cleavage |  | 1 |
| sbi-miR437i | SbNFY-B10 | 4.5 | -1 | 1 | 21 | 12917 | 12937 | AAAGUUAGAGAAGUUUGACUU | AGAUCUAAUUUUUUUGAUUUU | Cleavage |  | 1 |
| sbi-miR437j | SbNFY-B10 | 4.5 | -1 | 1 | 21 | 12917 | 12937 | AAAGUUAGAGAAGUUUGACUU | AGAUCUAAUUUUUUUGAUUUU | Cleavage |  | 1 |
| sbi-miR437k | SbNFY-B10 | 4.5 | -1 | 1 | 21 | 12917 | 12937 | AAAGUUAGAGAAGUUUGACUU | AGAUCUAAUUUUUUUGAUUUU | Cleavage |  | 1 |
| sbi-miR437l | SbNFY-B10 | 4.5 | -1 | 1 | 21 | 12917 | 12937 | AAAGUUAGAGAAGUUUGACUU | AGAUCUAAUUUUUUUGAUUUU | Cleavage |  | 1 |
| sbi-miR437m | SbNFY-B10 | 4.5 | -1 | 1 | 21 | 12917 | 12937 | AAAGUUAGAGAAGUUUGACUU | AGAUCUAAUUUUUUUGAUUUU | Cleavage |  | 1 |
| sbi-miR437n | SbNFY-B10 | 4.5 | -1 | 1 | 21 | 12917 | 12937 | AAAGUUAGAGAAGUUUGACUU | AGAUCUAAUUUUUUUGAUUUU | Cleavage |  | 1 |
| sbi-miR437o | SbNFY-B10 | 4.5 | -1 | 1 | 21 | 12917 | 12937 | AAAGUUAGAGAAGUUUGACUU | AGAUCUAAUUUUUUUGAUUUU | Cleavage |  | 1 |
| sbi-miR437p | SbNFY-B10 | 4.5 | -1 | 1 | 21 | 12917 | 12937 | AAAGUUAGAGAAGUUUGACUU | AGAUCUAAUUUUUUUGAUUUU | Cleavage |  | 1 |
| sbi-miR437q | SbNFY-B10 | 4.5 | -1 | 1 | 21 | 12917 | 12937 | AAAGUUAGAGAAGUUUGACUU | AGAUCUAAUUUUUUUGAUUUU | Cleavage |  | 1 |
| sbi-miR437r | SbNFY-B10 | 4.5 | -1 | 1 | 21 | 12917 | 12937 | AAAGUUAGAGAAGUUUGACUU | AGAUCUAAUUUUUUUGAUUUU | Cleavage |  | 1 |
| sbi-miR437s | SbNFY-B10 | 4.5 | -1 | 1 | 21 | 12917 | 12937 | AAAGUUAGAGAAGUUUGACUU | AGAUCUAAUUUUUUUGAUUUU | Cleavage |  | 1 |
| sbi-miR437t | SbNFY-B10 | 4.5 | -1 | 1 | 21 | 12917 | 12937 | AAAGUUAGAGAAGUUUGACUU | AGAUCUAAUUUUUUUGAUUUU | Cleavage |  | 1 |
| sbi-miR437u | SbNFY-B10 | 4.5 | -1 | 1 | 21 | 12917 | 12937 | AAAGUUAGAGAAGUUUGACUU | AGAUCUAAUUUUUUUGAUUUU | Cleavage |  | 1 |
| sbi-miR437v | SbNFY-B10 | 4.5 | -1 | 1 | 21 | 12917 | 12937 | AAAGUUAGAGAAGUUUGACUU | AGAUCUAAUUUUUUUGAUUUU | Cleavage |  | 1 |
| sbi-miR437w | SbNFY-B10 | 4.5 | -1 | 1 | 21 | 12917 | 12937 | AAAGUUAGAGAAGUUUGACUU | AGAUCUAAUUUUUUUGAUUUU | Cleavage |  | 1 |
| sbi-miR437x-5p | SbNFY-B1 | 4.5 | -1 | 1 | 24 | 15156 | 15179 | UAGAGUUGUCCUAAGUCAAACUUU | GCUACUAGCCUUAGGACAAUCUUA | Cleavage |  | 1 |
| sbi-miR528 | SbNFY-B1 | 4.5 | -1 | 1 | 21 | 6821 | 6841 | UGGAAGGGGCAUGCAGAGGAG | CUUUUCUGUUUGCCUUUUUCA | Cleavage |  | 2 |
| sbi-miR528 | SbNFY-B16 | 4.5 | -1 | 1 | 21 | 8346 | 8366 | UGGAAGGGGCAUGCAGAGGAG | CUCAUUUGCAUUCCCCUUUCU | Translation | | 1 |
| sbi-miR528 | SbNFY-B2 | 4.5 | -1 | 1 | 21 | 11670 | 11690 | UGGAAGGGGCAUGCAGAGGAG | CCCCUUUGCCUCCUCCUUUCA | Translation | | 2 |
| sbi-miR529 | SbNFY-B13 | 4.5 | -1 | 1 | 20 | 1033 | 1052 | CUGUACCCUCUCUCUUCUUC | GAAGCAGACAGGAGGUACAG | Cleavage |  | 1 |
| sbi-miR529 | SbNFY-B12 | 4.5 | -1 | 1 | 20 | 13182 | 13201 | CUGUACCCUCUCUCUUCUUC | AAAAAAGGGAAAAGGUACAG | Translation | | 2 |
| sbi-miR5386 | SbNFY-B10 | 4.5 | -1 | 1 | 20 | 8799 | 8817 | CGUCGCUGUCGCGCGCGCUG | CGGUGC-CGUGGCAGCGGCG | Cleavage |  | 3 |
| sbi-miR5389 | SbNFY-B19 | 4.5 | -1 | 1 | 21 | 9979 | 9999 | GCUUGAGUUUAUCAGCCGAGU | AUCAGCCUGAUAAACUCUAGC | Cleavage |  | 1 |
| sbi-miR5564a | SbNFY-B5 | 4.5 | -1 | 1 | 22 | 11696 | 11717 | UGGGGAAGCAAUUCGUCGAACA | CGAUUGAUGGAUUGCUUCACUC | Cleavage |  | 1 |
| sbi-miR5565c | SbNFY-B16 | 4.5 | -1 | 1 | 21 | 1975 | 1995 | UACACAUGUGGAUUGAGGUGA | AGACUCCGGUCCGCCUGUGUA | Cleavage |  | 2 |
| sbi-miR5565e | SbNFY-B19 | 4.5 | -1 | 1 | 19 | 14699 | 14717 | UUGUUUGGAUGUUGUCGGA | UUUGAAAACAUCUACGCAA | Cleavage |  | 2 |
| sbi-miR5565e | SbNFY-B17 | 4.5 | -1 | 1 | 19 | 11602 | 11620 | UUGUUUGGAUGUUGUCGGA | UUUGGUGAUAUCUAAAUGA | Cleavage |  | 1 |
| sbi-miR5565e | SbNFY-B3 | 4.5 | -1 | 1 | 19 | 337 | 355 | UUGUUUGGAUGUUGUCGGA | UUUGGUAACAUUCAAAGAG | Cleavage |  | 2 |
| sbi-miR5565g-3p | SbNFY-B7 | 4.5 | -1 | 1 | 24 | 548 | 571 | ACACAUGUGGAUUGAGAUGAAUAC | GUACGUGUCUCGAUCCGUGUCUGU | Cleavage |  | 1 |
| sbi-miR5567 | SbNFY-B7 | 4.5 | -1 | 1 | 24 | 8976 | 8999 | UUAAUGAUUCAUGUAUGUGUCCAA | GUUGUUACUUAAAUGAAUCAGUAA | Cleavage |  | 4 |
| sbi-miR5567 | SbNFY-B10 | 4.5 | -1 | 1 | 24 | 13615 | 13639 | UUAAUGAUUCAUGUAUGUGUC-CAA | UUGCGGCACAUGUAUAAAGUAUUAA | Translation | | 6 |
| sbi-miR5567 | SbNFY-B9 | 4.5 | -1 | 1 | 24 | 7060 | 7083 | UUAAUGAUUCAUGUAUGUGUCCAA | UUGGACACAUCCAUAAAGUAUUAA | Translation | | 5 |
| sbi-miR5567 | SbNFY-B9 | 4.5 | -1 | 1 | 24 | 12621 | 12644 | UUAAUGAUUCAUGUAUGUGUCCAA | UAGGGCAAAUGCAUACAUCAUUAA | Translation | | 5 |
| sbi-miR5567 | SbNFY-B18 | 4.5 | -1 | 1 | 24 | 7931 | 7954 | UUAAUGAUUCAUGUAUGUGUCCAA | UGUUUCAUCUACCUGAGUUAUUGA | Cleavage |  | 7 |
| sbi-miR5568b-3p | SbNFY-B10 | 4.5 | -1 | 1 | 21 | 7767 | 7787 | ACUAUGUAUCUAGAAAAGCUA | CAUCAUAUCUAGAUUCAUAGU | Cleavage |  | 2 |
| sbi-miR5568b-5p | SbNFY-B6 | 4.5 | -1 | 1 | 21 | 3273 | 3293 | UUUCUAGGUACAUAGCUUUUG | CAAACACUAUGAAUUUAGAAA | Translation | | 1 |
| sbi-miR5568b-5p | SbNFY-B1 | 4.5 | -1 | 1 | 21 | 10262 | 10282 | UUUCUAGGUACAUAGCUUUUG | UAAAAUAUAUGUAUUUAGAUA | Cleavage |  | 1 |
| sbi-miR5568b-5p | SbNFY-B5 | 4.5 | -1 | 1 | 21 | 15256 | 15276 | UUUCUAGGUACAUAGCUUUUG | AAAAUGCUGUGUAUCAGGGAA | Cleavage |  | 1 |
| sbi-miR5568c-3p | SbNFY-B6 | 4.5 | -1 | 1 | 21 | 3184 | 3204 | ACUUACAGUUUGGAACGGAGG | CCUCUGUCCCAAAUUAUAAGA | Cleavage |  | 2 |
| sbi-miR5568c-3p | SbNFY-B12 | 4.5 | -1 | 1 | 21 | 4925 | 4945 | ACUUACAGUUUGGAACGGAGG | UACUCUUUCCAAAUUAUAAGU | Cleavage |  | 2 |
| sbi-miR5568c-3p | SbNFY-B3 | 4.5 | -1 | 1 | 21 | 9970 | 9990 | ACUUACAGUUUGGAACGGAGG | CCUCAGUUUCAAAUUAUAAGC | Cleavage |  | 1 |
| sbi-miR5568c-3p | SbNFY-B14 | 4.5 | -1 | 1 | 21 | 6513 | 6533 | ACUUACAGUUUGGAACGGAGG | CUUCAUUUCUAAAUUAUAAGU | Cleavage |  | 1 |
| sbi-miR5568c-5p | SbNFY-B3 | 4.5 | -1 | 1 | 21 | 10064 | 10084 | UCUGUUCCAAAUUGUAAGUCG | GUACCUACAAUUUGGAAUGAU | Cleavage |  | 4 |
| sbi-miR5568c-5p | SbNFY-B8 | 4.5 | -1 | 1 | 21 | 15090 | 15110 | UCUGUUCCAAAUUGUAAGUCG | CUAUCUAUAAUUUAGGAUAGA | Cleavage |  | 2 |
| sbi-miR5568c-5p | SbNFY-B12 | 4.5 | -1 | 1 | 21 | 5041 | 5061 | UCUGUUCCAAAUUGUAAGUCG | CAACUUAUAAUUUGGAACUAA | Cleavage |  | 2 |
| sbi-miR5568c-5p | SbNFY-B14 | 4.5 | -1 | 1 | 21 | 6354 | 6374 | UCUGUUCCAAAUUGUAAGUCG | AAAUUUACGAGUUGGAGAAGA | Translation | | 2 |
| sbi-miR5568d-3p | SbNFY-B19 | 4.5 | -1 | 1 | 21 | 17641 | 17661 | AAAGUUGUGUAUCUAGAAAAG | CUUUCUUAAAUAUAUAAUUUU | Cleavage |  | 1 |
| sbi-miR5568d-3p | SbNFY-B5 | 4.5 | -1 | 1 | 21 | 11236 | 11256 | AAAGUUGUGUAUCUAGAAAAG | GUUUUUUAGGUAUAUGAUGUU | Cleavage |  | 2 |
| sbi-miR5568d-5p | SbNFY-B5 | 4.5 | -1 | 1 | 21 | 15261 | 15281 | UGGCUUUUCUAGAUACAUAGC | GCUGUGUAUCAGGGAAAGUCC | Translation | | 1 |
| sbi-miR5568e-3p | SbNFY-B6 | 4.5 | -1 | 1 | 21 | 3204 | 3224 | UAUCUAGAAAAGCUAAAACGU | ACGUUUUGGCAUUUCUAAAUC | Translation | | 1 |
| sbi-miR5568e-3p | SbNFY-B19 | 4.5 | -1 | 1 | 21 | 17632 | 17652 | UAUCUAGAAAAGCUAAAACGU | ACUUUUUAGCUUUCUUAAAUA | Cleavage |  | 1 |
| sbi-miR5568e-3p | SbNFY-B3 | 4.5 | -1 | 1 | 21 | 9990 | 10009 | UAUCUAGAAAAGCUAAAACGU | CAGUUUUAGCUUUUAUA-AUA | Cleavage |  | 1 |
| sbi-miR5568e-3p | SbNFY-B18 | 4.5 | -1 | 1 | 21 | 402 | 421 | UAUCUAGAAAAGCUAAAACGU | UAGUUUCAGUUUUUC-AGAUA | Cleavage |  | 1 |
| sbi-miR5568e-5p | SbNFY-B3 | 4.5 | -1 | 1 | 21 | 15886 | 15906 | GAUGUUUUGGGUUUUCUAGAU | AUCUUGGAGAGUCAAAGCAUC | Translation | | 3 |
| sbi-miR5568e-5p | SbNFY-B3 | 4.5 | -1 | 1 | 21 | 12985 | 13005 | GAUGUUUUGGGUUUUCUAGAU | AAUUAAAAGGUCUAAAAUAUU | Cleavage |  | 3 |
| sbi-miR5568e-5p | SbNFY-B3 | 4.5 | -1 | 1 | 21 | 17309 | 17329 | GAUGUUUUGGGUUUUCUAGAU | UACCAGGAAAUCCAAGAUGUA | Cleavage |  | 3 |
| sbi-miR5568e-5p | SbNFY-B12 | 4.5 | -1 | 1 | 21 | 16044 | 16065 | GAUGUUUUGGGUUUU-CUAGAU | CAAUAGCAGAACCUGAAACAUC | Cleavage |  | 1 |
| sbi-miR5568e-5p | SbNFY-B15 | 4.5 | -1 | 1 | 21 | 3684 | 3704 | GAUGUUUUGGGUUUUCUAGAU | UACCAAAAAAGCUAAAACAUU | Translation | | 1 |
| sbi-miR5568f-3p | SbNFY-B4 | 4.5 | -1 | 1 | 21 | 7134 | 7154 | GUCUUAUAAUUUGGAAUGGAG | CUCCAUUCCAAACUGUAAGUG | Cleavage |  | 2 |
| sbi-miR5568f-3p | SbNFY-B3 | 4.5 | -1 | 1 | 21 | 9971 | 9991 | GUCUUAUAAUUUGGAAUGGAG | CUCAGUUUCAAAUUAUAAGCA | Cleavage |  | 3 |
| sbi-miR5568f-3p | SbNFY-B10 | 4.5 | -1 | 1 | 21 | 7695 | 7715 | GUCUUAUAAUUUGGAAUGGAG | CUUGAUCCCAAAUUACAAGAU | Cleavage |  | 2 |
| sbi-miR5568f-3p | SbNFY-B16 | 4.5 | -1 | 1 | 21 | 7699 | 7719 | GUCUUAUAAUUUGGAAUGGAG | CCCUAUCUCAAAUUUUAAGAG | Cleavage |  | 1 |
| sbi-miR5568f-5p | SbNFY-B3 | 4.5 | -1 | 1 | 21 | 10064 | 10084 | UCCAUUCCAAAUUGUAAGAUG | GUACCUACAAUUUGGAAUGAU | Cleavage |  | 4 |
| sbi-miR5568f-5p | SbNFY-B5 | 4.5 | -1 | 1 | 21 | 14368 | 14388 | UCCAUUCCAAAUUGUAAGAUG | AUUUUUAUAAUUUAGAGUGAA | Cleavage |  | 2 |
| sbi-miR5568g-3p | SbNFY-B7 | 4.5 | -1 | 1 | 21 | 9182 | 9202 | AAAACGUCUUAUAAUUUGGAG | AACGAAAUUUUAAGAUGAUUU | Cleavage |  | 1 |
| sbi-miR5568g-5p | SbNFY-B14 | 4.5 | -1 | 1 | 21 | 4498 | 4518 | CAAAUUAUAAGAUGUUUUGGC | AGCAAAACAUCAUAUAGGUUC | Translation | | 2 |
| sbi-miR5568g-5p | SbNFY-B8 | 4.5 | -1 | 1 | 21 | 12770 | 12790 | CAAAUUAUAAGAUGUUUUGGC | UCCAUAUUAUCUUAUGAAUUG | Cleavage |  | 3 |
| sbi-miR5568g-5p | SbNFY-B7 | 4.5 | -1 | 1 | 21 | 3144 | 3164 | CAAAUUAUAAGAUGUUUUGGC | GACGAAGACUCUUAUAAUUUA | Cleavage |  | 1 |
| sbi-miR5570 | SbNFY-B15 | 4.5 | -1 | 1 | 21 | 11612 | 11632 | AAAAGACAAAUCAGCAUGUCA | UAACUUUAUGAUUGGUCUUUU | Cleavage |  | 1 |
| sbi-miR6217a-3p | SbNFY-B3 | 4.5 | -1 | 1 | 24 | 7248 | 7270 | AAAAUUAUCGUAAAUAGAGGUGGC | GAUAAUUCUAUUUAU-AUAGUUUU | Cleavage |  | 1 |
| sbi-miR6217a-3p | SbNFY-B19 | 4.5 | -1 | 1 | 24 | 15118 | 15141 | AAAAUUAUCGUAAAUAGAGGUGGC | UUAUGAUUUAUUGACAAUAAUUUU | Cleavage |  | 1 |
| sbi-miR6217a-3p | SbNFY-B1 | 4.5 | -1 | 1 | 24 | 14212 | 14235 | AAAAUUAUCGUAAAUAGAGGUGGC | UCAUAUUCUUUUCAUGAUAAUUUG | Cleavage |  | 1 |
| sbi-miR6217b-3p | SbNFY-B3 | 4.5 | -1 | 1 | 24 | 7248 | 7270 | AAAAUUAUCGUAAAUAGAGGUGGC | GAUAAUUCUAUUUAU-AUAGUUUU | Cleavage |  | 1 |
| sbi-miR6217b-3p | SbNFY-B19 | 4.5 | -1 | 1 | 24 | 15118 | 15141 | AAAAUUAUCGUAAAUAGAGGUGGC | UUAUGAUUUAUUGACAAUAAUUUU | Cleavage |  | 1 |
| sbi-miR6217b-3p | SbNFY-B1 | 4.5 | -1 | 1 | 24 | 14212 | 14235 | AAAAUUAUCGUAAAUAGAGGUGGC | UCAUAUUCUUUUCAUGAUAAUUUG | Cleavage |  | 1 |
| sbi-miR6218-3p | SbNFY-B15 | 4.5 | -1 | 1 | 21 | 14593 | 14613 | ACAAGUUUCGUGAUUUUUGGA | AGCAAAUAUUGUGGAACUCGU | Cleavage |  | 2 |
| sbi-miR6218-5p | SbNFY-B2 | 4.5 | -1 | 1 | 21 | 7209 | 7229 | CGAAAAUCACGAAACUUGUCG | AAACAUGUUUUGAGAUUUUAG | Cleavage |  | 1 |
| sbi-miR6220-3p | SbNFY-B6 | 4.5 | -1 | 1 | 24 | 3457 | 3480 | AUGCCUUAUAAUUUGGGAUGGAGA | CCUCCAUUCCAAACUGUAAGUCGU | Translation | | 2 |
| sbi-miR6220-3p | SbNFY-B12 | 4.5 | -1 | 1 | 24 | 14063 | 14086 | AUGCCUUAUAAUUUGGGAUGGAGA | ACUCUGUUUCAAAUUAUAAGUCGC | Cleavage |  | 2 |
| sbi-miR6220-3p | SbNFY-B5 | 4.5 | -1 | 1 | 24 | 618 | 641 | AUGCCUUAUAAUUUGGGAUGGAGA | CCUCAGUUCUAAAUUAGAGGGUGU | Cleavage |  | 1 |
| sbi-miR6220-5p | SbNFY-B8 | 4.5 | -1 | 1 | 24 | 15088 | 15111 | CUCCAUCCUAAAUUAUAAGACAUU | UGCUAUCUAUAAUUUAGGAUAGAA | Cleavage |  | 1 |
| sbi-miR6220-5p | SbNFY-B16 | 4.5 | -1 | 1 | 24 | 4317 | 4340 | CUCCAUCCUAAAUUAUAAGACAUU | ACGCACUCAAGAUUUAGGAUUGGG | Cleavage |  | 1 |
| sbi-miR6221-5p | SbNFY-B2 | 4.5 | -1 | 1 | 21 | 10378 | 10398 | UUCUGACUUCUGGCCCCUGCU | UGGAUGGCCCAGAACUCAGAA | Cleavage |  | 1 |
| sbi-miR6222-3p | SbNFY-B10 | 4.5 | -1 | 1 | 21 | 16245 | 16265 | CUAGCUGAUCCAAACAGGCCC | AGGCUUUUUUGGAUGAGCCAG | Cleavage |  | 1 |
| sbi-miR6222-5p | SbNFY-B9 | 4.5 | -1 | 1 | 21 | 2090 | 2110 | CCUGUUUGGAUCAGCCAAGGC | GACUUGGUUGAUAUAAGCAUG | Cleavage |  | 1 |
| sbi-miR6223-3p | SbNFY-B3 | 4.5 | -1 | 1 | 21 | 3505 | 3525 | CUAGCAUGUUCCUCCUAAGAG | AGGUUAGAGGGAGCAUGCUGU | Cleavage |  | 1 |
| sbi-miR6223-5p | SbNFY-B12 | 4.5 | -1 | 1 | 21 | 17284 | 17304 | UUCUUGGGAGGAGCAUGCUAG | CUAAAAUGGUUCUUCCAAGAA | Cleavage |  | 1 |
| sbi-miR6223-5p | SbNFY-B9 | 4.5 | -1 | 1 | 21 | 6872 | 6892 | UUCUUGGGAGGAGCAUGCUAG | CAAGCAGGCCCCUCCUGAGAU | Cleavage |  | 2 |
| sbi-miR6223-5p | SbNFY-B18 | 4.5 | -1 | 1 | 21 | 4339 | 4359 | UUCUUGGGAGGAGCAUGCUAG | AAGGAAUCCUCCUCUUGAGAG | Cleavage |  | 1 |
| sbi-miR6223-5p | SbNFY-B3 | 4.5 | -1 | 1 | 21 | 2110 | 2130 | UUCUUGGGAGGAGCAUGCUAG | GGAGAAUCUUCUUCCUAGGAG | Cleavage |  | 1 |
| sbi-miR6225-3p | SbNFY-B19 | 4.5 | -1 | 1 | 24 | 16598 | 16621 | GAAACGAAUCUUUUAAGUCUAAUU | AACUAGGCUCAAAAGAUCCGUCUC | Cleavage |  | 4 |
| sbi-miR6225-3p | SbNFY-B16 | 4.5 | -1 | 1 | 24 | 5026 | 5049 | GAAACGAAUCUUUUAAGUCUAAUU | AACUAGGCUCAAAAGAUUCAUCUC | Cleavage |  | 2 |
| sbi-miR6225-3p | SbNFY-B4 | 4.5 | -1 | 1 | 24 | 6259 | 6282 | GAAACGAAUCUUUUAAGUCUAAUU | AACUAGGCUUAAAAAAUUUGUCUU | Translation | | 7 |
| sbi-miR6225-3p | SbNFY-B4 | 4.5 | -1 | 1 | 24 | 7434 | 7457 | GAAACGAAUCUUUUAAGUCUAAUU | AACUAGGCUCAAAAUAUUCGUCUC | Translation | | 7 |
| sbi-miR6225-3p | SbNFY-B6 | 4.5 | -1 | 1 | 24 | 6306 | 6329 | GAAACGAAUCUUUUAAGUCUAAUU | AACUAGGAUCAAAAGAUUUGUCUC | Cleavage |  | 3 |
| sbi-miR6225-3p | SbNFY-B6 | 4.5 | -1 | 1 | 24 | 15653 | 15676 | GAAACGAAUCUUUUAAGUCUAAUU | UGUUUGUGGUAAAAGAUUCGUCUC | Cleavage |  | 3 |
| sbi-miR6225-5p | SbNFY-B9 | 4.5 | -1 | 1 | 24 | 12684 | 12707 | AACUAGACUCAAAAGAUUCAUCUC | GAGAUAAAUCUUUUGAACUUAGUU | Cleavage |  | 3 |
| sbi-miR6226-3p | SbNFY-B12 | 4.5 | -1 | 1 | 24 | 14762 | 14785 | GAUUAGUCACGAUUAGUCGUCCGA | CAGGACGACUAAUCGCGAUUAACG | Cleavage |  | 2 |
| sbi-miR6226-3p | SbNFY-B12 | 4.5 | -1 | 1 | 24 | 14255 | 14278 | GAUUAGUCACGAUUAGUCGUCCGA | CCAGAUGAUUAAUCUUGAUUUAUC | Translation | | 2 |
| sbi-miR6226-3p | SbNFY-B15 | 4.5 | -1 | 1 | 24 | 12239 | 12263 | GAUUAGUCACGAUUAGUC-GUCCGA | GCUGACAGAUUGAUUGUGGCUGAUC | Cleavage |  | 1 |
| sbi-miR6226-5p | SbNFY-B2 | 4.5 | -1 | 1 | 24 | 16336 | 16359 | AGAUCGGACGACUAAUCGCGAUUA | AAAAUGCGUUUAGUAGAUCGAUCU | Translation | | 1 |
| sbi-miR6228-3p | SbNFY-B4 | 4.5 | -1 | 1 | 24 | 436 | 459 | GUGGCAGUAGAAUUAAUGAAGGGA | UUUCUUCAAUAAAUAUACUGUCAC | Translation | | 2 |
| sbi-miR6228-3p | SbNFY-B4 | 4.5 | -1 | 1 | 24 | 15897 | 15920 | GUGGCAGUAGAAUUAAUGAAGGGA | UCCUGUCCUUAAUUUUACUUCCCC | Cleavage |  | 2 |
| sbi-miR6228-3p | SbNFY-B14 | 4.5 | -1 | 1 | 24 | 1602 | 1625 | GUGGCAGUAGAAUUAAUGAAGGGA | AUUCUUCAGUAAUUCUCCUGUUGU | Cleavage |  | 1 |
| sbi-miR6228-3p | SbNFY-B10 | 4.5 | -1 | 1 | 24 | 10071 | 10094 | GUGGCAGUAGAAUUAAUGAAGGGA | GGAGUUGUUUAAUUCUACUGGCGU | Cleavage |  | 1 |
| sbi-miR6228-5p | SbNFY-B7 | 4.5 | -1 | 1 | 24 | 12215 | 12238 | UUCUAUCUCUAUUAAUUGUGUUGC | AUGGAGUAUUAAAUAUAGAUAGAA | Cleavage |  | 1 |
| sbi-miR6230-3p | SbNFY-B8 | 4.5 | -1 | 1 | 21 | 4112 | 4132 | UAACAAGUUUAGGGAUCUAGA | GCUAGAUCUCUGAACAUUUUG | Cleavage |  | 1 |
| sbi-miR6230-3p | SbNFY-B10 | 4.5 | -1 | 1 | 21 | 565 | 585 | UAACAAGUUUAGGGAUCUAGA | AAUAGAACCUUAGAAUUGUUU | Cleavage |  | 1 |
| sbi-miR6231-3p | SbNFY-B19 | 4.5 | -1 | 1 | 21 | 11709 | 11729 | UAUUUGUGGACUCAUGGACAU | UGGUCCAUCAGUCCAUGGGUU | Cleavage |  | 1 |
| sbi-miR6232a-3p | SbNFY-B10 | 4.5 | -1 | 1 | 24 | 13114 | 13137 | UGGAUGUACCAAAAAAGUCAAAGC | AUUUUGGAUUUUUGGGUGCAUUUA | Translation | | 2 |
| sbi-miR6232a-3p | SbNFY-B10 | 4.5 | -1 | 1 | 24 | 7527 | 7550 | UGGAUGUACCAAAAAAGUCAAAGC | ACAUGGAAUAUUUUGGUGUUUCCA | Cleavage |  | 2 |
| sbi-miR6232a-5p | SbNFY-B8 | 4.5 | -1 | 1 | 24 | 8755 | 8777 | GUCGCUUUGACUUUUUUGGUACAU | AUAUAC-AGAAAAGUCAAAACGAU | Cleavage |  | 3 |
| sbi-miR6232b-3p | SbNFY-B7 | 4.5 | -1 | 1 | 21 | 12181 | 12201 | AAUUCGAUGUACCAAAAAAGU | GAUUUUUUGUCAUAUCGAAUC | Translation | | 1 |
| sbi-miR6232b-5p | SbNFY-B10 | 4.5 | -1 | 1 | 21 | 13083 | 13103 | UUUUUGGUACAUUGAAUUUGC | CAAGAUUCGAUGUGAUGAGAA | Cleavage |  | 4 |
| sbi-miR6232b-5p | SbNFY-B10 | 4.5 | -1 | 1 | 21 | 15999 | 16019 | UUUUUGGUACAUUGAAUUUGC | CAACAUUCGAUGUGAUAGAAA | Cleavage |  | 4 |
| sbi-miR6232b-5p | SbNFY-B6 | 4.5 | -1 | 1 | 21 | 15746 | 15766 | UUUUUGGUACAUUGAAUUUGC | AAAGAUUCGAUGUGACGGGAA | Cleavage |  | 5 |
| sbi-miR6232b-5p | SbNFY-B6 | 4.5 | -1 | 1 | 21 | 6399 | 6419 | UUUUUGGUACAUUGAAUUUGC | CAAGAUUCGAUGUGACGAGGA | Cleavage |  | 5 |
| sbi-miR6232b-5p | SbNFY-B6 | 4.5 | -1 | 1 | 21 | 1374 | 1393 | UUUUUGGUACAUUGAAUUUGC | CAAAAUUUAA-GUACCAGAAG | Translation | | 5 |
| sbi-miR6232b-5p | SbNFY-B4 | 4.5 | -1 | 1 | 21 | 11898 | 11918 | UUUUUGGUACAUUGAAUUUGC | AUCAAUUCAGUGUAUCCAAAU | Cleavage |  | 4 |
| sbi-miR6232b-5p | SbNFY-B4 | 4.5 | -1 | 1 | 21 | 7036 | 7056 | UUUUUGGUACAUUGAAUUUGC | AAAGAUUCGAUGUGACGGAGA | Cleavage |  | 4 |
| sbi-miR6232b-5p | SbNFY-B4 | 4.5 | -1 | 1 | 21 | 15057 | 15077 | UUUUUGGUACAUUGAAUUUGC | CAAGAUUCGAUGUGACGGAGA | Cleavage |  | 4 |
| sbi-miR6232b-5p | SbNFY-B12 | 4.5 | -1 | 1 | 21 | 9110 | 9130 | UUUUUGGUACAUUGAAUUUGC | AAAGAUUCGAUGUGACGGAGA | Cleavage |  | 4 |
| sbi-miR6232b-5p | SbNFY-B12 | 4.5 | -1 | 1 | 21 | 15448 | 15468 | UUUUUGGUACAUUGAAUUUGC | AUGAGAUGAAUGUGCUGAAAA | Cleavage |  | 4 |
| sbi-miR6232b-5p | SbNFY-B16 | 4.5 | -1 | 1 | 21 | 16147 | 16167 | UUUUUGGUACAUUGAAUUUGC | GCACAAGUAGUGUAUCAAAAA | Cleavage |  | 1 |
| sbi-miR6232b-5p | SbNFY-B14 | 4.5 | -1 | 1 | 21 | 6290 | 6310 | UUUUUGGUACAUUGAAUUUGC | UUUAAUUCAAUCUCCUAAAAA | Translation | | 1 |
| sbi-miR6232b-5p | SbNFY-B11 | 4.5 | -1 | 1 | 21 | 15435 | 15455 | UUUUUGGUACAUUGAAUUUGC | AAAGAUUUGAUGUGACAAGGA | Cleavage |  | 1 |
| sbi-miR6232b-5p | SbNFY-B3 | 4.5 | -1 | 1 | 21 | 11835 | 11855 | UUUUUGGUACAUUGAAUUUGC | CAAGAUUCGAUGUGACGAGGA | Cleavage |  | 1 |
| sbi-miR6232b-5p | SbNFY-B8 | 4.5 | -1 | 1 | 21 | 1120 | 1140 | UUUUUGGUACAUUGAAUUUGC | UAAGAUUUAAUGUGAUGAGAA | Cleavage |  | 2 |
| sbi-miR6232b-5p | SbNFY-B19 | 4.5 | -1 | 1 | 21 | 17302 | 17322 | UUUUUGGUACAUUGAAUUUGC | CAAGAUUCGAUGUGACGGGAA | Cleavage |  | 4 |
| sbi-miR6232b-5p | SbNFY-B13 | 4.5 | -1 | 1 | 21 | 3239 | 3259 | UUUUUGGUACAUUGAAUUUGC | CAAGUUUCAAUGUACAAAAGU | Cleavage |  | 1 |
| sbi-miR6232b-5p | SbNFY-B5 | 4.5 | -1 | 1 | 21 | 220 | 240 | UUUUUGGUACAUUGAAUUUGC | CAAGCUUCACUGUGCCAGAGA | Cleavage |  | 2 |
| sbi-miR6232b-5p | SbNFY-B2 | 4.5 | -1 | 1 | 21 | 417 | 437 | UUUUUGGUACAUUGAAUUUGC | CUACACUUAAUGUACUCAAAA | Cleavage |  | 1 |
| sbi-miR6233-3p | SbNFY-B10 | 4.5 | -1 | 1 | 24 | 6667 | 6690 | CAAGUUUGGUUUUGGUAAUUAAUG | CUCCGGUUGCUAAAACUAAACUAA | Cleavage |  | 1 |
| sbi-miR6233-3p | SbNFY-B18 | 4.5 | -1 | 1 | 24 | 14142 | 14165 | CAAGUUUGGUUUUGGUAAUUAAUG | GCAGACAUGCAAAAAUCAAAUUUG | Cleavage |  | 1 |
| sbi-miR6233-3p | SbNFY-B12 | 4.5 | -1 | 1 | 24 | 4662 | 4685 | CAAGUUUGGUUUUGGUAAUUAAUG | AUGCAAGUGCUAAAACCAGGAUUG | Cleavage |  | 1 |
| sbi-miR6233-5p | SbNFY-B11 | 4.5 | -1 | 1 | 24 | 789 | 812 | UGUUGAGGCUGGAGCGAAACUCGG | UGAUGCUUUGCUCUGGCAUCAAUA | Cleavage |  | 2 |
| sbi-miR6233-5p | SbNFY-B11 | 4.5 | -1 | 1 | 24 | 11555 | 11578 | UGUUGAGGCUGGAGCGAAACUCGG | ACAGCAUUCCCUCCAACCUCAACU | Cleavage |  | 2 |
| sbi-miR6233-5p | SbNFY-B18 | 4.5 | -1 | 1 | 24 | 5764 | 5787 | UGUUGAGGCUGGAGCGAAACUCGG | CAAUAUUUUUCUCCAGCCUUAAAG | Cleavage |  | 1 |
| sbi-miR6234a-5p | SbNFY-B7 | 4.5 | -1 | 1 | 24 | 7824 | 7847 | AAGUGUGUUCCUCUAUUUGACGCU | ACUAUCAAGUUGAGGGGUACCCUU | Cleavage |  | 2 |
| sbi-miR6234b-5p | SbNFY-B7 | 4.5 | -1 | 1 | 24 | 7824 | 7847 | AAGUGUGUUCCUCUAUUUGACGCU | ACUAUCAAGUUGAGGGGUACCCUU | Cleavage |  | 2 |
| sbi-miR6235-3p | SbNFY-B15 | 4.5 | -1 | 1 | 24 | 4205 | 4228 | AACGAACAGUAUUUUUCUCUUACA | UCUAUGAAAACAACGUUGUUCGUU | Translation | | 1 |
| sbi-miR6235-5p | SbNFY-B18 | 4.5 | -1 | 1 | 24 | 11132 | 11155 | UUGUGAGAGAAAAAUACUGUUGGC | GCUGCUAGCAUUAUUUUCUCACAU | Cleavage |  | 1 |
| sbi-miR6235-5p | SbNFY-B5 | 4.5 | -1 | 1 | 24 | 2689 | 2712 | UUGUGAGAGAAAAAUACUGUUGGC | CCUCCUAGUAUUUUUUUUUAAAAA | Cleavage |  | 1 |
| sbi-miR821a | SbNFY-B11 | 4.5 | -1 | 1 | 21 | 9174 | 9194 | AAGUCAUCAACAUAAAAGUUG | UAGUUUUGAUUUUGAUGGUUU | Translation | | 1 |
| sbi-miR821b | SbNFY-B7 | 4.5 | -1 | 1 | 21 | 15988 | 16008 | AAGUUAUGAACAUAAAAGUUG | CGACUUUAGUGUACAUAUCUU | Cleavage |  | 2 |
| sbi-miR821b | SbNFY-B5 | 4.5 | -1 | 1 | 21 | 8271 | 8291 | AAGUUAUGAACAUAAAAGUUG | CCAUUUAUAUUUUUAUAGUUU | Translation | | 1 |
| sbi-miR821c | SbNFY-B11 | 4.5 | -1 | 1 | 21 | 9174 | 9194 | AAGUCAUCAACAUAAAAGUUG | UAGUUUUGAUUUUGAUGGUUU | Translation | | 1 |
| sbi-miR821d | SbNFY-B3 | 4.5 | -1 | 1 | 21 | 2858 | 2878 | AAGUCAUCAACAACAAAGUUG | GAGCUCUCUUGGUGGUGACUU | Translation | | 1 |
| sbi-miR821e | SbNFY-B10 | 4.5 | -1 | 1 | 21 | 9834 | 9854 | AAGUCAUCAAAAUAAAAGUUG | AAAUUUUGGUUUUGGUUAUUU | Cleavage |  | 2 |
| sbi-miR1432 | SbNFY-B16 | 5 | -1 | 1 | 21 | 14766 | 14786 | CUCAGGAGAGAUGACACCGAC | AUCAGUGUUAUUUCGUCUGAA | Cleavage |  | 1 |
| sbi-miR1435a | SbNFY-B1 | 5 | -1 | 1 | 20 | 14500 | 14520 | UUUCUUAAGUCAA-ACUUUUC | GAAAAGUGUUGGCUUCAGAAA | Cleavage |  | 1 |
| sbi-miR1435a | SbNFY-B9 | 5 | -1 | 1 | 20 | 12321 | 12340 | UUUCUUAAGUCAAACUUUUC | GAAAGAUUUGAUUUGAUGAG | Cleavage |  | 2 |
| sbi-miR1435b | SbNFY-B18 | 5 | -1 | 1 | 20 | 121 | 140 | UUUCUUAAGUCAAACCUUUU | AAAGGGCUUGUCUUAGAAAA | Translation | | 1 |
| sbi-miR1435b | SbNFY-B14 | 5 | -1 | 1 | 20 | 537 | 556 | UUUCUUAAGUCAAACCUUUU | GAUAGUUUUGACUUAGGAUG | Cleavage |  | 1 |
| sbi-miR156a | SbNFY-B5 | 5 | -1 | 1 | 20 | 5395 | 5414 | UGACAGAAGAGAGUGAGCAC | AUCCUUUUUUUUUUCUGUCU | Cleavage |  | 2 |
| sbi-miR156b | SbNFY-B5 | 5 | -1 | 1 | 20 | 5395 | 5414 | UGACAGAAGAGAGUGAGCAC | AUCCUUUUUUUUUUCUGUCU | Cleavage |  | 2 |
| sbi-miR156c | SbNFY-B5 | 5 | -1 | 1 | 20 | 5395 | 5414 | UGACAGAAGAGAGUGAGCAC | AUCCUUUUUUUUUUCUGUCU | Cleavage |  | 2 |
| sbi-miR156d | SbNFY-B10 | 5 | -1 | 1 | 21 | 2066 | 2087 | UGACAGAAGAGAGA-GAGCACA | CAGGCUCAUCUCCCUUUUGUCA | Translation | | 2 |
| sbi-miR156d | SbNFY-B14 | 5 | -1 | 1 | 21 | 9017 | 9037 | UGACAGAAGAGAGAGAGCACA | AUUAUUCUCUCUUUUUUGUAU | Cleavage |  | 1 |
| sbi-miR156d | SbNFY-B18 | 5 | -1 | 1 | 21 | 17443 | 17463 | UGACAGAAGAGAGAGAGCACA | UCGUCGUUCUUUCUUUUGUUA | Cleavage |  | 1 |
| sbi-miR156d | SbNFY-B3 | 5 | -1 | 1 | 21 | 17739 | 17759 | UGACAGAAGAGAGAGAGCACA | ACUGGUCUUUCUUUUUAGUUG | Cleavage |  | 2 |
| sbi-miR156d | SbNFY-B3 | 5 | -1 | 1 | 21 | 5800 | 5820 | UGACAGAAGAGAGAGAGCACA | CAUACUUUUUUUUUUCUUUUA | Cleavage |  | 2 |
| sbi-miR156d | SbNFY-B16 | 5 | -1 | 1 | 21 | 17640 | 17660 | UGACAGAAGAGAGAGAGCACA | AUUCUUCAUUCUCUUUUGUUU | Cleavage |  | 1 |
| sbi-miR156d | SbNFY-B19 | 5 | -1 | 1 | 21 | 1455 | 1475 | UGACAGAAGAGAGAGAGCACA | UUUGAACUCGCUCUUUUGUUG | Cleavage |  | 1 |
| sbi-miR156e | SbNFY-B5 | 5 | -1 | 1 | 20 | 5395 | 5414 | UGACAGAAGAGAGCGAGCAC | AUCCUUUUUUUUUUCUGUCU | Cleavage |  | 2 |
| sbi-miR156e | SbNFY-B16 | 5 | -1 | 1 | 20 | 17641 | 17660 | UGACAGAAGAGAGCGAGCAC | UUCUUCAUUCUCUUUUGUUU | Cleavage |  | 1 |
| sbi-miR156f | SbNFY-B5 | 5 | -1 | 1 | 20 | 5395 | 5414 | UGACAGAAGAGAGUGAGCAC | AUCCUUUUUUUUUUCUGUCU | Cleavage |  | 2 |
| sbi-miR156g | SbNFY-B5 | 5 | -1 | 1 | 20 | 5395 | 5414 | UGACAGAAGAGAGUGAGCAC | AUCCUUUUUUUUUUCUGUCU | Cleavage |  | 2 |
| sbi-miR156h | SbNFY-B5 | 5 | -1 | 1 | 20 | 5395 | 5414 | UGACAGAAGAGAGUGAGCAC | AUCCUUUUUUUUUUCUGUCU | Cleavage |  | 2 |
| sbi-miR156i | SbNFY-B5 | 5 | -1 | 1 | 20 | 5395 | 5414 | UGACAGAAGAGAGUGAGCAC | AUCCUUUUUUUUUUCUGUCU | Cleavage |  | 2 |
| sbi-miR159a | SbNFY-B14 | 5 | -1 | 1 | 21 | 8087 | 8107 | UUUGGAUUGAAGGGAGCUCUG | UCCAGUUUGUUUUAAUUCAAA | Cleavage |  | 2 |
| sbi-miR159a | SbNFY-B4 | 5 | -1 | 1 | 21 | 7125 | 7145 | UUUGGAUUGAAGGGAGCUCUG | UAGUACUCCCUCCAUUCCAAA | Translation | | 1 |
| sbi-miR159a | SbNFY-B10 | 5 | -1 | 1 | 21 | 9710 | 9730 | UUUGGAUUGAAGGGAGCUCUG | AUGAGUUCAUUUGAAUUUAAA | Cleavage |  | 1 |
| sbi-miR159a | SbNFY-B18 | 5 | -1 | 1 | 21 | 46 | 66 | UUUGGAUUGAAGGGAGCUCUG | UUGAGUUUUUUUCCAUUUAAG | Cleavage |  | 1 |
| sbi-miR159a | SbNFY-B7 | 5 | -1 | 1 | 21 | 15172 | 15192 | UUUGGAUUGAAGGGAGCUCUG | AAGGCUUACCUUCACUUCAAA | Cleavage |  | 1 |
| sbi-miR159a | SbNFY-B17 | 5 | -1 | 1 | 21 | 12185 | 12206 | UUUGGAUUGAAGGG-AGCUCUG | AUGGGCUACUCUUCAGUUCAAC | Cleavage |  | 1 |
| sbi-miR159a | SbNFY-B9 | 5 | -1 | 1 | 21 | 3968 | 3988 | UUUGGAUUGAAGGGAGCUCUG | CCGAACUGUCUGCAGUUCAAA | Translation | | 1 |
| sbi-miR159b | SbNFY-B2 | 5 | -1 | 1 | 21 | 10321 | 10341 | CUUGGAUUGAAGGGAGCUCCU | AAAAGCUGCCUACAAUUCAAA | Translation | | 1 |
| sbi-miR159b | SbNFY-B17 | 5 | -1 | 1 | 21 | 12185 | 12206 | CUUGGAUUGAAGGG-AGCUCCU | AUGGGCUACUCUUCAGUUCAAC | Cleavage |  | 1 |
| sbi-miR160a | SbNFY-B11 | 5 | -1 | 1 | 21 | 14435 | 14455 | UGCCUGGCUCCCUGUAUGCCA | GUGCGUGCGGGGGGCGGGGCC | Cleavage |  | 1 |
| sbi-miR160a | SbNFY-B4 | 5 | -1 | 1 | 21 | 13049 | 13069 | UGCCUGGCUCCCUGUAUGCCA | CGGCCUCCAGUGAGCUGGGUA | Translation | | 1 |
| sbi-miR160b | SbNFY-B11 | 5 | -1 | 1 | 21 | 14435 | 14455 | UGCCUGGCUCCCUGUAUGCCA | GUGCGUGCGGGGGGCGGGGCC | Cleavage |  | 1 |
| sbi-miR160b | SbNFY-B4 | 5 | -1 | 1 | 21 | 13049 | 13069 | UGCCUGGCUCCCUGUAUGCCA | CGGCCUCCAGUGAGCUGGGUA | Translation | | 1 |
| sbi-miR160c | SbNFY-B11 | 5 | -1 | 1 | 21 | 14435 | 14455 | UGCCUGGCUCCCUGUAUGCCA | GUGCGUGCGGGGGGCGGGGCC | Cleavage |  | 1 |
| sbi-miR160c | SbNFY-B4 | 5 | -1 | 1 | 21 | 13049 | 13069 | UGCCUGGCUCCCUGUAUGCCA | CGGCCUCCAGUGAGCUGGGUA | Translation | | 1 |
| sbi-miR160d | SbNFY-B11 | 5 | -1 | 1 | 21 | 14435 | 14455 | UGCCUGGCUCCCUGUAUGCCA | GUGCGUGCGGGGGGCGGGGCC | Cleavage |  | 1 |
| sbi-miR160d | SbNFY-B4 | 5 | -1 | 1 | 21 | 13049 | 13069 | UGCCUGGCUCCCUGUAUGCCA | CGGCCUCCAGUGAGCUGGGUA | Translation | | 1 |
| sbi-miR160e | SbNFY-B11 | 5 | -1 | 1 | 21 | 14435 | 14455 | UGCCUGGCUCCCUGUAUGCCA | GUGCGUGCGGGGGGCGGGGCC | Cleavage |  | 1 |
| sbi-miR160e | SbNFY-B4 | 5 | -1 | 1 | 21 | 13049 | 13069 | UGCCUGGCUCCCUGUAUGCCA | CGGCCUCCAGUGAGCUGGGUA | Translation | | 1 |
| sbi-miR160f | SbNFY-B4 | 5 | -1 | 1 | 21 | 13049 | 13069 | UGCCUGGCUCCCUGAAUGCCA | CGGCCUCCAGUGAGCUGGGUA | Translation | | 1 |
| sbi-miR164a | SbNFY-B16 | 5 | -1 | 1 | 21 | 16798 | 16818 | UGGAGAAGCAGGGCACGUGCA | CCACCGCGCCCUCCUUCUCCG | Cleavage |  | 1 |
| sbi-miR164b | SbNFY-B16 | 5 | -1 | 1 | 21 | 16798 | 16818 | UGGAGAAGCAGGGCACGUGCU | CCACCGCGCCCUCCUUCUCCG | Cleavage |  | 1 |
| sbi-miR164c | SbNFY-B3 | 5 | -1 | 1 | 21 | 10449 | 10469 | UGGAGAAGCAGGACACGUGAG | AGUGUGUGUUUUGUUUUUCUU | Cleavage |  | 1 |
| sbi-miR164c | SbNFY-B16 | 5 | -1 | 1 | 21 | 11502 | 11522 | UGGAGAAGCAGGACACGUGAG | UACAGAUAUCCUGUUUCUACA | Cleavage |  | 1 |
| sbi-miR164d | SbNFY-B16 | 5 | -1 | 1 | 21 | 16798 | 16818 | UGGAGAAGCAGGGCACGUGCA | CCACCGCGCCCUCCUUCUCCG | Cleavage |  | 1 |
| sbi-miR164e | SbNFY-B16 | 5 | -1 | 1 | 21 | 16798 | 16818 | UGGAGAAGCAGGGCACGUGCA | CCACCGCGCCCUCCUUCUCCG | Cleavage |  | 1 |
| sbi-miR166f | SbNFY-B13 | 5 | -1 | 1 | 21 | 15523 | 15543 | UCGGACCAGGCUUCAUUCCUC | UAGUCAUCAAGCCUGGUCAGG | Cleavage |  | 1 |
| sbi-miR166k | SbNFY-B13 | 5 | -1 | 1 | 20 | 15524 | 15543 | UCGGACCAGGCUUCAUUCCU | AGUCAUCAAGCCUGGUCAGG | Cleavage |  | 1 |
| sbi-miR168 | SbNFY-B12 | 5 | -1 | 1 | 21 | 3987 | 4007 | UCGCUUGGUGCAGAUCGGGAC | GUCCUGAUUUGAGCAAAGUGA | Translation | | 1 |
| sbi-miR168 | SbNFY-B15 | 5 | -1 | 1 | 21 | 14226 | 14246 | UCGCUUGGUGCAGAUCGGGAC | UUCCAGACCUGCUCCGGGUGA | Cleavage |  | 2 |
| sbi-miR168 | SbNFY-B15 | 5 | -1 | 1 | 21 | 3205 | 3225 | UCGCUUGGUGCAGAUCGGGAC | ACCUCGGCUUCCACCAGGCGG | Translation | | 2 |
| sbi-miR168 | SbNFY-B10 | 5 | -1 | 1 | 21 | 4616 | 4635 | UCGCUUGGUGCAGAUCGGGAC | GCCACGGUCUGCAC-AGGCGA | Cleavage |  | 1 |
| sbi-miR169c | SbNFY-B4 | 5 | -1 | 1 | 21 | 17248 | 17268 | UAGCCAAGGAUGACUUGCCUA | UUGCUAAGUUGUCUUUUGUUA | Cleavage |  | 1 |
| sbi-miR169d-3p | SbNFY-B6 | 5 | -1 | 1 | 20 | 12691 | 12710 | GGGCGGUCACCUUGGCUAGC | UGUGGAUAAGGUGAUCGCAC | Cleavage |  | 1 |
| sbi-miR169d-5p | SbNFY-B4 | 5 | -1 | 1 | 20 | 17249 | 17268 | UAGCCAAGGAUGACUUGCCU | UGCUAAGUUGUCUUUUGUUA | Cleavage |  | 1 |
| sbi-miR169e | SbNFY-B4 | 5 | -1 | 1 | 21 | 17248 | 17268 | UAGCCAAGGAUGACUUGCCGG | UUGCUAAGUUGUCUUUUGUUA | Cleavage |  | 1 |
| sbi-miR169f | SbNFY-B4 | 5 | -1 | 1 | 21 | 17248 | 17268 | UAGCCAAGGAUGACUUGCCUG | UUGCUAAGUUGUCUUUUGUUA | Cleavage |  | 1 |
| sbi-miR169g | SbNFY-B4 | 5 | -1 | 1 | 21 | 17248 | 17268 | UAGCCAAGGAUGACUUGCCUG | UUGCUAAGUUGUCUUUUGUUA | Cleavage |  | 1 |
| sbi-miR169h | SbNFY-B4 | 5 | -1 | 1 | 21 | 17248 | 17268 | UAGCCAAGGAUGACUUGCCUA | UUGCUAAGUUGUCUUUUGUUA | Cleavage |  | 1 |
| sbi-miR169i | SbNFY-B11 | 5 | -1 | 1 | 21 | 60 | 79 | UAGCCAAGAAUGACUUGCCUA | GAGGCGAG-CAUUUUUGGCUC | Cleavage |  | 1 |
| sbi-miR169j | SbNFY-B4 | 5 | -1 | 1 | 21 | 17248 | 17268 | UAGCCAAGGAUGACUUGCCGG | UUGCUAAGUUGUCUUUUGUUA | Cleavage |  | 1 |
| sbi-miR169l | SbNFY-B4 | 5 | -1 | 1 | 21 | 17248 | 17268 | UAGCCAAGGAUGACUUGCCUG | UUGCUAAGUUGUCUUUUGUUA | Cleavage |  | 1 |
| sbi-miR169m | SbNFY-B4 | 5 | -1 | 1 | 21 | 17248 | 17268 | UAGCCAAGGAUGACUUGCCUA | UUGCUAAGUUGUCUUUUGUUA | Cleavage |  | 1 |
| sbi-miR169n | SbNFY-B4 | 5 | -1 | 1 | 21 | 17248 | 17268 | UAGCCAAGGAUGACUUGCCUA | UUGCUAAGUUGUCUUUUGUUA | Cleavage |  | 1 |
| sbi-miR169o | SbNFY-B9 | 5 | -1 | 1 | 21 | 15634 | 15654 | UAGCCAAGGAUGAUUUGCCUG | CAGUUCAAUCAGUCUUGGUUA | Translation | | 1 |
| sbi-miR169p | SbNFY-B11 | 5 | -1 | 1 | 21 | 60 | 79 | UAGCCAAGAAUGGCUUGCCUA | GAGGCGAGC-AUUUUUGGCUC | Cleavage |  | 1 |
| sbi-miR169p | SbNFY-B6 | 5 | -1 | 1 | 21 | 1579 | 1599 | UAGCCAAGAAUGGCUUGCCUA | CAAGCAAACUAUUCUUGACUU | Cleavage |  | 1 |
| sbi-miR169q | SbNFY-B11 | 5 | -1 | 1 | 21 | 60 | 79 | UAGCCAAGAAUGGCUUGCCUA | GAGGCGAGC-AUUUUUGGCUC | Cleavage |  | 1 |
| sbi-miR169q | SbNFY-B6 | 5 | -1 | 1 | 21 | 1579 | 1599 | UAGCCAAGAAUGGCUUGCCUA | CAAGCAAACUAUUCUUGACUU | Cleavage |  | 1 |
| sbi-miR171a | SbNFY-B12 | 5 | -1 | 1 | 21 | 17322 | 17342 | UGAUUGAGCCGUGCCAAUAUC | UUUACAGGUAUGGCUCAGUGA | Cleavage |  | 1 |
| sbi-miR171b | SbNFY-B12 | 5 | -1 | 1 | 21 | 17322 | 17342 | UGAUUGAGCCGUGCCAAUAUC | UUUACAGGUAUGGCUCAGUGA | Cleavage |  | 1 |
| sbi-miR171d | SbNFY-B12 | 5 | -1 | 1 | 21 | 17322 | 17342 | UGAUUGAGCCGUGCCAAUAUC | UUUACAGGUAUGGCUCAGUGA | Cleavage |  | 1 |
| sbi-miR171i | SbNFY-B12 | 5 | -1 | 1 | 21 | 17322 | 17342 | UGAUUGAGCCGUGCCAAUAUC | UUUACAGGUAUGGCUCAGUGA | Cleavage |  | 1 |
| sbi-miR171k | SbNFY-B12 | 5 | -1 | 1 | 21 | 17322 | 17342 | UGAUUGAGCCGUGCCAAUAUC | UUUACAGGUAUGGCUCAGUGA | Cleavage |  | 1 |
| sbi-miR172a | SbNFY-B13 | 5 | -1 | 1 | 20 | 665 | 684 | AGAAUCUUGAUGAUGCUGCA | GACAGCAUCAGCUGGAUUUU | Translation | | 2 |
| sbi-miR172a | SbNFY-B14 | 5 | -1 | 1 | 20 | 9876 | 9895 | AGAAUCUUGAUGAUGCUGCA | UUGAGUAUUAUUUAGAUUCU | Cleavage |  | 1 |
| sbi-miR172a | SbNFY-B2 | 5 | -1 | 1 | 20 | 11897 | 11916 | AGAAUCUUGAUGAUGCUGCA | AGCAGGGUUAUCCAAAUUCU | Cleavage |  | 1 |
| sbi-miR172a | SbNFY-B16 | 5 | -1 | 1 | 20 | 14523 | 14541 | AGAAUCUUGAUGAUGCUGCA | AGCCGC-UCAACAAGGUUCU | Translation | | 1 |
| sbi-miR172c | SbNFY-B13 | 5 | -1 | 1 | 20 | 665 | 684 | AGAAUCUUGAUGAUGCUGCA | GACAGCAUCAGCUGGAUUUU | Translation | | 2 |
| sbi-miR172c | SbNFY-B14 | 5 | -1 | 1 | 20 | 9876 | 9895 | AGAAUCUUGAUGAUGCUGCA | UUGAGUAUUAUUUAGAUUCU | Cleavage |  | 1 |
| sbi-miR172c | SbNFY-B2 | 5 | -1 | 1 | 20 | 11897 | 11916 | AGAAUCUUGAUGAUGCUGCA | AGCAGGGUUAUCCAAAUUCU | Cleavage |  | 1 |
| sbi-miR172c | SbNFY-B16 | 5 | -1 | 1 | 20 | 14523 | 14541 | AGAAUCUUGAUGAUGCUGCA | AGCCGC-UCAACAAGGUUCU | Translation | | 1 |
| sbi-miR172d | SbNFY-B13 | 5 | -1 | 1 | 20 | 665 | 684 | AGAAUCUUGAUGAUGCUGCA | GACAGCAUCAGCUGGAUUUU | Translation | | 2 |
| sbi-miR172d | SbNFY-B14 | 5 | -1 | 1 | 20 | 9876 | 9895 | AGAAUCUUGAUGAUGCUGCA | UUGAGUAUUAUUUAGAUUCU | Cleavage |  | 1 |
| sbi-miR172d | SbNFY-B2 | 5 | -1 | 1 | 20 | 11897 | 11916 | AGAAUCUUGAUGAUGCUGCA | AGCAGGGUUAUCCAAAUUCU | Cleavage |  | 1 |
| sbi-miR172d | SbNFY-B16 | 5 | -1 | 1 | 20 | 14523 | 14541 | AGAAUCUUGAUGAUGCUGCA | AGCCGC-UCAACAAGGUUCU | Translation | | 1 |
| sbi-miR172e | SbNFY-B18 | 5 | -1 | 1 | 21 | 10365 | 10385 | UGAAUCUUGAUGAUGCUGCAC | CUACAACAUUUUUAAGAUUUA | Translation | | 1 |
| sbi-miR172e | SbNFY-B14 | 5 | -1 | 1 | 21 | 8225 | 8245 | UGAAUCUUGAUGAUGCUGCAC | GCCCGCCAUCAGUAAGAUUUA | Translation | | 1 |
| sbi-miR172e | SbNFY-B13 | 5 | -1 | 1 | 21 | 1903 | 1923 | UGAAUCUUGAUGAUGCUGCAC | UCGCAGCUUUGUCAAGGUACU | Cleavage |  | 1 |
| sbi-miR172e | SbNFY-B3 | 5 | -1 | 1 | 21 | 3637 | 3657 | UGAAUCUUGAUGAUGCUGCAC | UGGCAGCUUCGCCAAAGUUCA | Translation | | 1 |
| sbi-miR172f | SbNFY-B17 | 5 | -1 | 1 | 21 | 4006 | 4026 | AGAAUCCUGAUGAUGCUGCAC | UUUCAUUGUCGUCAGGCUUCU | Cleavage |  | 1 |
| sbi-miR172f | SbNFY-B3 | 5 | -1 | 1 | 21 | 3082 | 3102 | AGAAUCCUGAUGAUGCUGCAC | UUACAGUGUCAUCGGGGAUUU | Cleavage |  | 1 |
| sbi-miR172f | SbNFY-B7 | 5 | -1 | 1 | 21 | 8904 | 8924 | AGAAUCCUGAUGAUGCUGCAC | UAGCACGAUGAUCAGGAUUUA | Cleavage |  | 1 |
| sbi-miR172f | SbNFY-B11 | 5 | -1 | 1 | 21 | 11310 | 11330 | AGAAUCCUGAUGAUGCUGCAC | AUGUCUCUUGAUCAGGAUUCU | Cleavage |  | 1 |
| sbi-miR2118-3p | SbNFY-B13 | 5 | -1 | 1 | 22 | 9716 | 9737 | UUCCUGAUGCCUCCCAUGCCUA | GCUUCGUGCGGGCCAUCGGGAA | Translation | | 1 |
| sbi-miR2118-5p | SbNFY-B18 | 5 | -1 | 1 | 22 | 11105 | 11126 | GGCAUGGGAACAUGUAGGAAGG | GUUACCUACAUGUAACCAUGCA | Cleavage |  | 1 |
| sbi-miR2118-5p | SbNFY-B12 | 5 | -1 | 1 | 22 | 7116 | 7137 | GGCAUGGGAACAUGUAGGAAGG | AGUUCUUGCCUUUUCUCAUGUC | Translation | | 1 |
| sbi-miR390 | SbNFY-B16 | 5 | -1 | 1 | 21 | 16544 | 16564 | AAGCUCAGGAGGGAUAGCGCC | GGCCUUGACCCCCUUGAGCUU | Translation | | 1 |
| sbi-miR393a | SbNFY-B11 | 5 | -1 | 1 | 21 | 12386 | 12406 | UCCAAAGGGAUCGCAUUGAUC | CAUCCAUUCGAGCCAUUUGGA | Translation | | 1 |
| sbi-miR393b | SbNFY-B11 | 5 | -1 | 1 | 21 | 12386 | 12406 | UCCAAAGGGAUCGCAUUGAUC | CAUCCAUUCGAGCCAUUUGGA | Translation | | 1 |
| sbi-miR394a | SbNFY-B19 | 5 | -1 | 1 | 20 | 16236 | 16255 | UUGGCAUUCUGUCCACCUCC | CCGCGUAGGCAGGAUGCCAA | Cleavage |  | 1 |
| sbi-miR394b | SbNFY-B19 | 5 | -1 | 1 | 20 | 16236 | 16255 | UUGGCAUUCUGUCCACCUCC | CCGCGUAGGCAGGAUGCCAA | Cleavage |  | 1 |
| sbi-miR395a | SbNFY-B5 | 5 | -1 | 1 | 21 | 5626 | 5646 | GUGAAGUGUUUGGGGGAACUC | UUUUUUUCUUGGACACUUUGC | Cleavage |  | 3 |
| sbi-miR395a | SbNFY-B5 | 5 | -1 | 1 | 21 | 13011 | 13031 | GUGAAGUGUUUGGGGGAACUC | UCGUUCGCACAGACGCUUCUC | Cleavage |  | 3 |
| sbi-miR395a | SbNFY-B18 | 5 | -1 | 1 | 21 | 7084 | 7104 | GUGAAGUGUUUGGGGGAACUC | AAGUUCCUCCAAAAAAUUUGU | Cleavage |  | 1 |
| sbi-miR395a | SbNFY-B16 | 5 | -1 | 1 | 21 | 5314 | 5334 | GUGAAGUGUUUGGGGGAACUC | UUGUUUUACCAAACACUUUUU | Cleavage |  | 1 |
| sbi-miR395a | SbNFY-B9 | 5 | -1 | 1 | 21 | 877 | 897 | GUGAAGUGUUUGGGGGAACUC | GCGGUUCUUCGAGUACUUCAG | Cleavage |  | 1 |
| sbi-miR395b | SbNFY-B5 | 5 | -1 | 1 | 21 | 5626 | 5646 | GUGAAGUGUUUGGGGGAACUC | UUUUUUUCUUGGACACUUUGC | Cleavage |  | 3 |
| sbi-miR395b | SbNFY-B5 | 5 | -1 | 1 | 21 | 13011 | 13031 | GUGAAGUGUUUGGGGGAACUC | UCGUUCGCACAGACGCUUCUC | Cleavage |  | 3 |
| sbi-miR395b | SbNFY-B18 | 5 | -1 | 1 | 21 | 7084 | 7104 | GUGAAGUGUUUGGGGGAACUC | AAGUUCCUCCAAAAAAUUUGU | Cleavage |  | 1 |
| sbi-miR395b | SbNFY-B16 | 5 | -1 | 1 | 21 | 5314 | 5334 | GUGAAGUGUUUGGGGGAACUC | UUGUUUUACCAAACACUUUUU | Cleavage |  | 1 |
| sbi-miR395b | SbNFY-B9 | 5 | -1 | 1 | 21 | 877 | 897 | GUGAAGUGUUUGGGGGAACUC | GCGGUUCUUCGAGUACUUCAG | Cleavage |  | 1 |
| sbi-miR395c | SbNFY-B5 | 5 | -1 | 1 | 21 | 5626 | 5646 | GUGAAGUGUUUGGGGGAACUC | UUUUUUUCUUGGACACUUUGC | Cleavage |  | 3 |
| sbi-miR395c | SbNFY-B5 | 5 | -1 | 1 | 21 | 13011 | 13031 | GUGAAGUGUUUGGGGGAACUC | UCGUUCGCACAGACGCUUCUC | Cleavage |  | 3 |
| sbi-miR395c | SbNFY-B18 | 5 | -1 | 1 | 21 | 7084 | 7104 | GUGAAGUGUUUGGGGGAACUC | AAGUUCCUCCAAAAAAUUUGU | Cleavage |  | 1 |
| sbi-miR395c | SbNFY-B16 | 5 | -1 | 1 | 21 | 5314 | 5334 | GUGAAGUGUUUGGGGGAACUC | UUGUUUUACCAAACACUUUUU | Cleavage |  | 1 |
| sbi-miR395c | SbNFY-B9 | 5 | -1 | 1 | 21 | 877 | 897 | GUGAAGUGUUUGGGGGAACUC | GCGGUUCUUCGAGUACUUCAG | Cleavage |  | 1 |
| sbi-miR395d | SbNFY-B5 | 5 | -1 | 1 | 21 | 5626 | 5646 | GUGAAGUGUUUGGGGGAACUC | UUUUUUUCUUGGACACUUUGC | Cleavage |  | 3 |
| sbi-miR395d | SbNFY-B5 | 5 | -1 | 1 | 21 | 13011 | 13031 | GUGAAGUGUUUGGGGGAACUC | UCGUUCGCACAGACGCUUCUC | Cleavage |  | 3 |
| sbi-miR395d | SbNFY-B18 | 5 | -1 | 1 | 21 | 7084 | 7104 | GUGAAGUGUUUGGGGGAACUC | AAGUUCCUCCAAAAAAUUUGU | Cleavage |  | 1 |
| sbi-miR395d | SbNFY-B16 | 5 | -1 | 1 | 21 | 5314 | 5334 | GUGAAGUGUUUGGGGGAACUC | UUGUUUUACCAAACACUUUUU | Cleavage |  | 1 |
| sbi-miR395d | SbNFY-B9 | 5 | -1 | 1 | 21 | 877 | 897 | GUGAAGUGUUUGGGGGAACUC | GCGGUUCUUCGAGUACUUCAG | Cleavage |  | 1 |
| sbi-miR395e | SbNFY-B5 | 5 | -1 | 1 | 21 | 5626 | 5646 | GUGAAGUGUUUGGGGGAACUC | UUUUUUUCUUGGACACUUUGC | Cleavage |  | 3 |
| sbi-miR395e | SbNFY-B5 | 5 | -1 | 1 | 21 | 13011 | 13031 | GUGAAGUGUUUGGGGGAACUC | UCGUUCGCACAGACGCUUCUC | Cleavage |  | 3 |
| sbi-miR395e | SbNFY-B18 | 5 | -1 | 1 | 21 | 7084 | 7104 | GUGAAGUGUUUGGGGGAACUC | AAGUUCCUCCAAAAAAUUUGU | Cleavage |  | 1 |
| sbi-miR395e | SbNFY-B16 | 5 | -1 | 1 | 21 | 5314 | 5334 | GUGAAGUGUUUGGGGGAACUC | UUGUUUUACCAAACACUUUUU | Cleavage |  | 1 |
| sbi-miR395e | SbNFY-B9 | 5 | -1 | 1 | 21 | 877 | 897 | GUGAAGUGUUUGGGGGAACUC | GCGGUUCUUCGAGUACUUCAG | Cleavage |  | 1 |
| sbi-miR395f | SbNFY-B18 | 5 | -1 | 1 | 21 | 12940 | 12960 | AUGAAGUGUUUGGGGGAACUC | GAGUUGCUCCAAACUUUUGAU | Cleavage |  | 2 |
| sbi-miR395f | SbNFY-B5 | 5 | -1 | 1 | 21 | 5283 | 5303 | AUGAAGUGUUUGGGGGAACUC | GCUUUCUUUCUAACACUUUGU | Translation | | 2 |
| sbi-miR395f | SbNFY-B9 | 5 | -1 | 1 | 21 | 877 | 897 | AUGAAGUGUUUGGGGGAACUC | GCGGUUCUUCGAGUACUUCAG | Cleavage |  | 2 |
| sbi-miR395f | SbNFY-B9 | 5 | -1 | 1 | 21 | 14399 | 14419 | AUGAAGUGUUUGGGGGAACUC | ACAUUAUCUCAAACACUUGGU | Cleavage |  | 2 |
| sbi-miR395f | SbNFY-B13 | 5 | -1 | 1 | 21 | 185 | 205 | AUGAAGUGUUUGGGGGAACUC | AUGUGCCCUCAAAUAAUUGAU | Cleavage |  | 1 |
| sbi-miR395f | SbNFY-B12 | 5 | -1 | 1 | 21 | 16455 | 16475 | AUGAAGUGUUUGGGGGAACUC | UUGUGACUUCAGACACUUCCU | Cleavage |  | 1 |
| sbi-miR395g | SbNFY-B5 | 5 | -1 | 1 | 21 | 5626 | 5646 | GUGAAGUGUUUGGGGGAACUC | UUUUUUUCUUGGACACUUUGC | Cleavage |  | 3 |
| sbi-miR395g | SbNFY-B5 | 5 | -1 | 1 | 21 | 13011 | 13031 | GUGAAGUGUUUGGGGGAACUC | UCGUUCGCACAGACGCUUCUC | Cleavage |  | 3 |
| sbi-miR395g | SbNFY-B18 | 5 | -1 | 1 | 21 | 7084 | 7104 | GUGAAGUGUUUGGGGGAACUC | AAGUUCCUCCAAAAAAUUUGU | Cleavage |  | 1 |
| sbi-miR395g | SbNFY-B16 | 5 | -1 | 1 | 21 | 5314 | 5334 | GUGAAGUGUUUGGGGGAACUC | UUGUUUUACCAAACACUUUUU | Cleavage |  | 1 |
| sbi-miR395g | SbNFY-B9 | 5 | -1 | 1 | 21 | 877 | 897 | GUGAAGUGUUUGGGGGAACUC | GCGGUUCUUCGAGUACUUCAG | Cleavage |  | 1 |
| sbi-miR395h | SbNFY-B5 | 5 | -1 | 1 | 21 | 5626 | 5646 | GUGAAGUGUUUGGGGGAACUC | UUUUUUUCUUGGACACUUUGC | Cleavage |  | 3 |
| sbi-miR395h | SbNFY-B5 | 5 | -1 | 1 | 21 | 13011 | 13031 | GUGAAGUGUUUGGGGGAACUC | UCGUUCGCACAGACGCUUCUC | Cleavage |  | 3 |
| sbi-miR395h | SbNFY-B18 | 5 | -1 | 1 | 21 | 7084 | 7104 | GUGAAGUGUUUGGGGGAACUC | AAGUUCCUCCAAAAAAUUUGU | Cleavage |  | 1 |
| sbi-miR395h | SbNFY-B16 | 5 | -1 | 1 | 21 | 5314 | 5334 | GUGAAGUGUUUGGGGGAACUC | UUGUUUUACCAAACACUUUUU | Cleavage |  | 1 |
| sbi-miR395h | SbNFY-B9 | 5 | -1 | 1 | 21 | 877 | 897 | GUGAAGUGUUUGGGGGAACUC | GCGGUUCUUCGAGUACUUCAG | Cleavage |  | 1 |
| sbi-miR395i | SbNFY-B5 | 5 | -1 | 1 | 21 | 5626 | 5646 | GUGAAGUGUUUGGGGGAACUC | UUUUUUUCUUGGACACUUUGC | Cleavage |  | 3 |
| sbi-miR395i | SbNFY-B5 | 5 | -1 | 1 | 21 | 13011 | 13031 | GUGAAGUGUUUGGGGGAACUC | UCGUUCGCACAGACGCUUCUC | Cleavage |  | 3 |
| sbi-miR395i | SbNFY-B18 | 5 | -1 | 1 | 21 | 7084 | 7104 | GUGAAGUGUUUGGGGGAACUC | AAGUUCCUCCAAAAAAUUUGU | Cleavage |  | 1 |
| sbi-miR395i | SbNFY-B16 | 5 | -1 | 1 | 21 | 5314 | 5334 | GUGAAGUGUUUGGGGGAACUC | UUGUUUUACCAAACACUUUUU | Cleavage |  | 1 |
| sbi-miR395i | SbNFY-B9 | 5 | -1 | 1 | 21 | 877 | 897 | GUGAAGUGUUUGGGGGAACUC | GCGGUUCUUCGAGUACUUCAG | Cleavage |  | 1 |
| sbi-miR395j | SbNFY-B5 | 5 | -1 | 1 | 21 | 5626 | 5646 | GUGAAGUGUUUGGGGGAACUC | UUUUUUUCUUGGACACUUUGC | Cleavage |  | 3 |
| sbi-miR395j | SbNFY-B5 | 5 | -1 | 1 | 21 | 13011 | 13031 | GUGAAGUGUUUGGGGGAACUC | UCGUUCGCACAGACGCUUCUC | Cleavage |  | 3 |
| sbi-miR395j | SbNFY-B18 | 5 | -1 | 1 | 21 | 7084 | 7104 | GUGAAGUGUUUGGGGGAACUC | AAGUUCCUCCAAAAAAUUUGU | Cleavage |  | 1 |
| sbi-miR395j | SbNFY-B16 | 5 | -1 | 1 | 21 | 5314 | 5334 | GUGAAGUGUUUGGGGGAACUC | UUGUUUUACCAAACACUUUUU | Cleavage |  | 1 |
| sbi-miR395j | SbNFY-B9 | 5 | -1 | 1 | 21 | 877 | 897 | GUGAAGUGUUUGGGGGAACUC | GCGGUUCUUCGAGUACUUCAG | Cleavage |  | 1 |
| sbi-miR395k | SbNFY-B18 | 5 | -1 | 1 | 21 | 12940 | 12960 | GUGAAGUGUUUGGAGGAACUC | GAGUUGCUCCAAACUUUUGAU | Cleavage |  | 2 |
| sbi-miR395k | SbNFY-B16 | 5 | -1 | 1 | 21 | 5314 | 5334 | GUGAAGUGUUUGGAGGAACUC | UUGUUUUACCAAACACUUUUU | Cleavage |  | 1 |
| sbi-miR395k | SbNFY-B15 | 5 | -1 | 1 | 21 | 14030 | 14050 | GUGAAGUGUUUGGAGGAACUC | UCAAUUCUUCAAGCCCUUCAC | Cleavage |  | 1 |
| sbi-miR395k | SbNFY-B5 | 5 | -1 | 1 | 21 | 5283 | 5303 | GUGAAGUGUUUGGAGGAACUC | GCUUUCUUUCUAACACUUUGU | Translation | | 2 |
| sbi-miR395k | SbNFY-B5 | 5 | -1 | 1 | 21 | 5040 | 5060 | GUGAAGUGUUUGGAGGAACUC | UUGUUUUCUCAAGCAUUUUAG | Cleavage |  | 2 |
| sbi-miR395l | SbNFY-B15 | 5 | -1 | 1 | 21 | 14030 | 14050 | GUGAAGUGCUUGGGGGAACUC | UCAAUUCUUCAAGCCCUUCAC | Cleavage |  | 1 |
| sbi-miR396a | SbNFY-B15 | 5 | -1 | 1 | 21 | 2972 | 2992 | UUCCACAGCUUUCUUGAACUG | CAGUUUGCGAAACUUGUGGAU | Cleavage |  | 1 |
| sbi-miR396a | SbNFY-B3 | 5 | -1 | 1 | 21 | 13560 | 13580 | UUCCACAGCUUUCUUGAACUG | CAGUCGCAGAAAGCUGGGGGA | Cleavage |  | 1 |
| sbi-miR396b | SbNFY-B15 | 5 | -1 | 1 | 21 | 2972 | 2992 | UUCCACAGCUUUCUUGAACUG | CAGUUUGCGAAACUUGUGGAU | Cleavage |  | 1 |
| sbi-miR396b | SbNFY-B3 | 5 | -1 | 1 | 21 | 13560 | 13580 | UUCCACAGCUUUCUUGAACUG | CAGUCGCAGAAAGCUGGGGGA | Cleavage |  | 1 |
| sbi-miR396c | SbNFY-B15 | 5 | -1 | 1 | 21 | 2972 | 2992 | UUCCACAGCUUUCUUGAACUU | CAGUUUGCGAAACUUGUGGAU | Cleavage |  | 1 |
| sbi-miR396c | SbNFY-B3 | 5 | -1 | 1 | 21 | 13560 | 13580 | UUCCACAGCUUUCUUGAACUU | CAGUCGCAGAAAGCUGGGGGA | Cleavage |  | 1 |
| sbi-miR397-3p | SbNFY-B19 | 5 | -1 | 1 | 21 | 11988 | 12008 | UCACCGGCGCUGCACUCAAUU | AGGUGCUUGUGGUGCCGGUGG | Cleavage |  | 1 |
| sbi-miR397-5p | SbNFY-B19 | 5 | -1 | 1 | 21 | 15220 | 15240 | UCAUUGAGUGCAGCGUUGAUG | CUGUAGUGCUGCAUACAAUGG | Cleavage |  | 1 |
| sbi-miR399a | SbNFY-B6 | 5 | -1 | 1 | 21 | 6912 | 6932 | UGCCAAAGGAGAAUUGCCCUG | UAGGACAAUUUUUUUUUAGCU | Cleavage |  | 1 |
| sbi-miR399a | SbNFY-B19 | 5 | -1 | 1 | 21 | 8956 | 8976 | UGCCAAAGGAGAAUUGCCCUG | GAGAGUGAUUUUCCUUUAGCU | Cleavage |  | 1 |
| sbi-miR399c | SbNFY-B6 | 5 | -1 | 1 | 21 | 6912 | 6932 | UGCCAAAGGAGAAUUGCCCUG | UAGGACAAUUUUUUUUUAGCU | Cleavage |  | 1 |
| sbi-miR399c | SbNFY-B19 | 5 | -1 | 1 | 21 | 8956 | 8976 | UGCCAAAGGAGAAUUGCCCUG | GAGAGUGAUUUUCCUUUAGCU | Cleavage |  | 1 |
| sbi-miR399d | SbNFY-B19 | 5 | -1 | 1 | 21 | 13381 | 13401 | UGCCAAAGGAGAGUUGCCCUG | GUGUGCCACUUGUCUUUGGUA | Translation | | 1 |
| sbi-miR399g | SbNFY-B19 | 5 | -1 | 1 | 21 | 5641 | 5661 | UGCCAAAGGAAAUUUGCCCCG | ACCAGCAAGUUUUGUUUGGCG | Cleavage |  | 1 |
| sbi-miR399g | SbNFY-B16 | 5 | -1 | 1 | 21 | 4947 | 4967 | UGCCAAAGGAAAUUUGCCCCG | ACUGGCAAAAUUUUUUUUGCA | Cleavage |  | 1 |
| sbi-miR399h | SbNFY-B6 | 5 | -1 | 1 | 21 | 6912 | 6932 | UGCCAAAGGAGAAUUGCCCUG | UAGGACAAUUUUUUUUUAGCU | Cleavage |  | 1 |
| sbi-miR399h | SbNFY-B19 | 5 | -1 | 1 | 21 | 8956 | 8976 | UGCCAAAGGAGAAUUGCCCUG | GAGAGUGAUUUUCCUUUAGCU | Cleavage |  | 1 |
| sbi-miR399i | SbNFY-B19 | 5 | -1 | 1 | 21 | 13381 | 13401 | UGCCAAAGGAGAGUUGCCCUG | GUGUGCCACUUGUCUUUGGUA | Translation | | 1 |
| sbi-miR399j | SbNFY-B6 | 5 | -1 | 1 | 21 | 6912 | 6932 | UGCCAAAGGAGAAUUGCCCUG | UAGGACAAUUUUUUUUUAGCU | Cleavage |  | 1 |
| sbi-miR399j | SbNFY-B19 | 5 | -1 | 1 | 21 | 8956 | 8976 | UGCCAAAGGAGAAUUGCCCUG | GAGAGUGAUUUUCCUUUAGCU | Cleavage |  | 1 |
| sbi-miR399k | SbNFY-B3 | 5 | -1 | 1 | 21 | 7560 | 7580 | UGCCAAAGGGGAUUUGCCCGG | ACUAUUAGAUCUCUUUUGGCA | Cleavage |  | 1 |
| sbi-miR399k | SbNFY-B8 | 5 | -1 | 1 | 21 | 468 | 488 | UGCCAAAGGGGAUUUGCCCGG | AUGAGUGUGUCCCAUUUGGCA | Cleavage |  | 1 |
| sbi-miR408 | SbNFY-B4 | 5 | -1 | 1 | 21 | 14449 | 14469 | CUGCACUGCCUCUUCCCUGGC | GACAGGGAGGAGCCGGUGCCU | Cleavage |  | 1 |
| sbi-miR408 | SbNFY-B5 | 5 | -1 | 1 | 21 | 15455 | 15475 | CUGCACUGCCUCUUCCCUGGC | CCCAGUGCAGACGUAGUGCAU | Translation | | 1 |
| sbi-miR437a | SbNFY-B11 | 5 | -1 | 1 | 21 | 12561 | 12581 | AAAGUUAGAGAAGUUUGACUU | AAGUAGAACGUCUCUACUUUU | Cleavage |  | 1 |
| sbi-miR437a | SbNFY-B17 | 5 | -1 | 1 | 21 | 5202 | 5222 | AAAGUUAGAGAAGUUUGACUU | AACGCGAACUUCUCUAGUUCU | Cleavage |  | 1 |
| sbi-miR437a | SbNFY-B15 | 5 | -1 | 1 | 21 | 14402 | 14422 | AAAGUUAGAGAAGUUUGACUU | GAGUGGAGUUUUUUUUACUUU | Cleavage |  | 1 |
| sbi-miR437a | SbNFY-B1 | 5 | -1 | 1 | 21 | 3109 | 3129 | AAAGUUAGAGAAGUUUGACUU | AGCAUAAAUUUCUUUUACUUU | Cleavage |  | 1 |
| sbi-miR437a | SbNFY-B18 | 5 | -1 | 1 | 21 | 979 | 999 | AAAGUUAGAGAAGUUUGACUU | GCUACAAAGUUCUAUAACUUU | Cleavage |  | 1 |
| sbi-miR437a | SbNFY-B19 | 5 | -1 | 1 | 21 | 10713 | 10733 | AAAGUUAGAGAAGUUUGACUU | UCCUUACAUUUUUCUAAGUUU | Cleavage |  | 1 |
| sbi-miR437b | SbNFY-B11 | 5 | -1 | 1 | 21 | 12561 | 12581 | AAAGUUAGAGAAGUUUGACUU | AAGUAGAACGUCUCUACUUUU | Cleavage |  | 1 |
| sbi-miR437b | SbNFY-B17 | 5 | -1 | 1 | 21 | 5202 | 5222 | AAAGUUAGAGAAGUUUGACUU | AACGCGAACUUCUCUAGUUCU | Cleavage |  | 1 |
| sbi-miR437b | SbNFY-B15 | 5 | -1 | 1 | 21 | 14402 | 14422 | AAAGUUAGAGAAGUUUGACUU | GAGUGGAGUUUUUUUUACUUU | Cleavage |  | 1 |
| sbi-miR437b | SbNFY-B1 | 5 | -1 | 1 | 21 | 3109 | 3129 | AAAGUUAGAGAAGUUUGACUU | AGCAUAAAUUUCUUUUACUUU | Cleavage |  | 1 |
| sbi-miR437b | SbNFY-B18 | 5 | -1 | 1 | 21 | 979 | 999 | AAAGUUAGAGAAGUUUGACUU | GCUACAAAGUUCUAUAACUUU | Cleavage |  | 1 |
| sbi-miR437b | SbNFY-B19 | 5 | -1 | 1 | 21 | 10713 | 10733 | AAAGUUAGAGAAGUUUGACUU | UCCUUACAUUUUUCUAAGUUU | Cleavage |  | 1 |
| sbi-miR437c | SbNFY-B11 | 5 | -1 | 1 | 21 | 12561 | 12581 | AAAGUUAGAGAAGUUUGACUU | AAGUAGAACGUCUCUACUUUU | Cleavage |  | 1 |
| sbi-miR437c | SbNFY-B17 | 5 | -1 | 1 | 21 | 5202 | 5222 | AAAGUUAGAGAAGUUUGACUU | AACGCGAACUUCUCUAGUUCU | Cleavage |  | 1 |
| sbi-miR437c | SbNFY-B15 | 5 | -1 | 1 | 21 | 14402 | 14422 | AAAGUUAGAGAAGUUUGACUU | GAGUGGAGUUUUUUUUACUUU | Cleavage |  | 1 |
| sbi-miR437c | SbNFY-B1 | 5 | -1 | 1 | 21 | 3109 | 3129 | AAAGUUAGAGAAGUUUGACUU | AGCAUAAAUUUCUUUUACUUU | Cleavage |  | 1 |
| sbi-miR437c | SbNFY-B18 | 5 | -1 | 1 | 21 | 979 | 999 | AAAGUUAGAGAAGUUUGACUU | GCUACAAAGUUCUAUAACUUU | Cleavage |  | 1 |
| sbi-miR437c | SbNFY-B19 | 5 | -1 | 1 | 21 | 10713 | 10733 | AAAGUUAGAGAAGUUUGACUU | UCCUUACAUUUUUCUAAGUUU | Cleavage |  | 1 |
| sbi-miR437d | SbNFY-B11 | 5 | -1 | 1 | 21 | 12561 | 12581 | AAAGUUAGAGAAGUUUGACUU | AAGUAGAACGUCUCUACUUUU | Cleavage |  | 1 |
| sbi-miR437d | SbNFY-B17 | 5 | -1 | 1 | 21 | 5202 | 5222 | AAAGUUAGAGAAGUUUGACUU | AACGCGAACUUCUCUAGUUCU | Cleavage |  | 1 |
| sbi-miR437d | SbNFY-B15 | 5 | -1 | 1 | 21 | 14402 | 14422 | AAAGUUAGAGAAGUUUGACUU | GAGUGGAGUUUUUUUUACUUU | Cleavage |  | 1 |
| sbi-miR437d | SbNFY-B1 | 5 | -1 | 1 | 21 | 3109 | 3129 | AAAGUUAGAGAAGUUUGACUU | AGCAUAAAUUUCUUUUACUUU | Cleavage |  | 1 |
| sbi-miR437d | SbNFY-B18 | 5 | -1 | 1 | 21 | 979 | 999 | AAAGUUAGAGAAGUUUGACUU | GCUACAAAGUUCUAUAACUUU | Cleavage |  | 1 |
| sbi-miR437d | SbNFY-B19 | 5 | -1 | 1 | 21 | 10713 | 10733 | AAAGUUAGAGAAGUUUGACUU | UCCUUACAUUUUUCUAAGUUU | Cleavage |  | 1 |
| sbi-miR437e | SbNFY-B11 | 5 | -1 | 1 | 21 | 12561 | 12581 | AAAGUUAGAGAAGUUUGACUU | AAGUAGAACGUCUCUACUUUU | Cleavage |  | 1 |
| sbi-miR437e | SbNFY-B17 | 5 | -1 | 1 | 21 | 5202 | 5222 | AAAGUUAGAGAAGUUUGACUU | AACGCGAACUUCUCUAGUUCU | Cleavage |  | 1 |
| sbi-miR437e | SbNFY-B15 | 5 | -1 | 1 | 21 | 14402 | 14422 | AAAGUUAGAGAAGUUUGACUU | GAGUGGAGUUUUUUUUACUUU | Cleavage |  | 1 |
| sbi-miR437e | SbNFY-B1 | 5 | -1 | 1 | 21 | 3109 | 3129 | AAAGUUAGAGAAGUUUGACUU | AGCAUAAAUUUCUUUUACUUU | Cleavage |  | 1 |
| sbi-miR437e | SbNFY-B18 | 5 | -1 | 1 | 21 | 979 | 999 | AAAGUUAGAGAAGUUUGACUU | GCUACAAAGUUCUAUAACUUU | Cleavage |  | 1 |
| sbi-miR437e | SbNFY-B19 | 5 | -1 | 1 | 21 | 10713 | 10733 | AAAGUUAGAGAAGUUUGACUU | UCCUUACAUUUUUCUAAGUUU | Cleavage |  | 1 |
| sbi-miR437f | SbNFY-B11 | 5 | -1 | 1 | 21 | 12561 | 12581 | AAAGUUAGAGAAGUUUGACUU | AAGUAGAACGUCUCUACUUUU | Cleavage |  | 1 |
| sbi-miR437f | SbNFY-B17 | 5 | -1 | 1 | 21 | 5202 | 5222 | AAAGUUAGAGAAGUUUGACUU | AACGCGAACUUCUCUAGUUCU | Cleavage |  | 1 |
| sbi-miR437f | SbNFY-B15 | 5 | -1 | 1 | 21 | 14402 | 14422 | AAAGUUAGAGAAGUUUGACUU | GAGUGGAGUUUUUUUUACUUU | Cleavage |  | 1 |
| sbi-miR437f | SbNFY-B1 | 5 | -1 | 1 | 21 | 3109 | 3129 | AAAGUUAGAGAAGUUUGACUU | AGCAUAAAUUUCUUUUACUUU | Cleavage |  | 1 |
| sbi-miR437f | SbNFY-B18 | 5 | -1 | 1 | 21 | 979 | 999 | AAAGUUAGAGAAGUUUGACUU | GCUACAAAGUUCUAUAACUUU | Cleavage |  | 1 |
| sbi-miR437f | SbNFY-B19 | 5 | -1 | 1 | 21 | 10713 | 10733 | AAAGUUAGAGAAGUUUGACUU | UCCUUACAUUUUUCUAAGUUU | Cleavage |  | 1 |
| sbi-miR437g | SbNFY-B11 | 5 | -1 | 1 | 21 | 12561 | 12581 | AAAGUUAGAGAAGUUUGACUU | AAGUAGAACGUCUCUACUUUU | Cleavage |  | 1 |
| sbi-miR437g | SbNFY-B17 | 5 | -1 | 1 | 21 | 5202 | 5222 | AAAGUUAGAGAAGUUUGACUU | AACGCGAACUUCUCUAGUUCU | Cleavage |  | 1 |
| sbi-miR437g | SbNFY-B15 | 5 | -1 | 1 | 21 | 14402 | 14422 | AAAGUUAGAGAAGUUUGACUU | GAGUGGAGUUUUUUUUACUUU | Cleavage |  | 1 |
| sbi-miR437g | SbNFY-B1 | 5 | -1 | 1 | 21 | 3109 | 3129 | AAAGUUAGAGAAGUUUGACUU | AGCAUAAAUUUCUUUUACUUU | Cleavage |  | 1 |
| sbi-miR437g | SbNFY-B18 | 5 | -1 | 1 | 21 | 979 | 999 | AAAGUUAGAGAAGUUUGACUU | GCUACAAAGUUCUAUAACUUU | Cleavage |  | 1 |
| sbi-miR437g | SbNFY-B19 | 5 | -1 | 1 | 21 | 10713 | 10733 | AAAGUUAGAGAAGUUUGACUU | UCCUUACAUUUUUCUAAGUUU | Cleavage |  | 1 |
| sbi-miR437i | SbNFY-B11 | 5 | -1 | 1 | 21 | 12561 | 12581 | AAAGUUAGAGAAGUUUGACUU | AAGUAGAACGUCUCUACUUUU | Cleavage |  | 1 |
| sbi-miR437i | SbNFY-B17 | 5 | -1 | 1 | 21 | 5202 | 5222 | AAAGUUAGAGAAGUUUGACUU | AACGCGAACUUCUCUAGUUCU | Cleavage |  | 1 |
| sbi-miR437i | SbNFY-B15 | 5 | -1 | 1 | 21 | 14402 | 14422 | AAAGUUAGAGAAGUUUGACUU | GAGUGGAGUUUUUUUUACUUU | Cleavage |  | 1 |
| sbi-miR437i | SbNFY-B1 | 5 | -1 | 1 | 21 | 3109 | 3129 | AAAGUUAGAGAAGUUUGACUU | AGCAUAAAUUUCUUUUACUUU | Cleavage |  | 1 |
| sbi-miR437i | SbNFY-B18 | 5 | -1 | 1 | 21 | 979 | 999 | AAAGUUAGAGAAGUUUGACUU | GCUACAAAGUUCUAUAACUUU | Cleavage |  | 1 |
| sbi-miR437i | SbNFY-B19 | 5 | -1 | 1 | 21 | 10713 | 10733 | AAAGUUAGAGAAGUUUGACUU | UCCUUACAUUUUUCUAAGUUU | Cleavage |  | 1 |
| sbi-miR437j | SbNFY-B11 | 5 | -1 | 1 | 21 | 12561 | 12581 | AAAGUUAGAGAAGUUUGACUU | AAGUAGAACGUCUCUACUUUU | Cleavage |  | 1 |
| sbi-miR437j | SbNFY-B17 | 5 | -1 | 1 | 21 | 5202 | 5222 | AAAGUUAGAGAAGUUUGACUU | AACGCGAACUUCUCUAGUUCU | Cleavage |  | 1 |
| sbi-miR437j | SbNFY-B15 | 5 | -1 | 1 | 21 | 14402 | 14422 | AAAGUUAGAGAAGUUUGACUU | GAGUGGAGUUUUUUUUACUUU | Cleavage |  | 1 |
| sbi-miR437j | SbNFY-B1 | 5 | -1 | 1 | 21 | 3109 | 3129 | AAAGUUAGAGAAGUUUGACUU | AGCAUAAAUUUCUUUUACUUU | Cleavage |  | 1 |
| sbi-miR437j | SbNFY-B18 | 5 | -1 | 1 | 21 | 979 | 999 | AAAGUUAGAGAAGUUUGACUU | GCUACAAAGUUCUAUAACUUU | Cleavage |  | 1 |
| sbi-miR437j | SbNFY-B19 | 5 | -1 | 1 | 21 | 10713 | 10733 | AAAGUUAGAGAAGUUUGACUU | UCCUUACAUUUUUCUAAGUUU | Cleavage |  | 1 |
| sbi-miR437k | SbNFY-B11 | 5 | -1 | 1 | 21 | 12561 | 12581 | AAAGUUAGAGAAGUUUGACUU | AAGUAGAACGUCUCUACUUUU | Cleavage |  | 1 |
| sbi-miR437k | SbNFY-B17 | 5 | -1 | 1 | 21 | 5202 | 5222 | AAAGUUAGAGAAGUUUGACUU | AACGCGAACUUCUCUAGUUCU | Cleavage |  | 1 |
| sbi-miR437k | SbNFY-B15 | 5 | -1 | 1 | 21 | 14402 | 14422 | AAAGUUAGAGAAGUUUGACUU | GAGUGGAGUUUUUUUUACUUU | Cleavage |  | 1 |
| sbi-miR437k | SbNFY-B1 | 5 | -1 | 1 | 21 | 3109 | 3129 | AAAGUUAGAGAAGUUUGACUU | AGCAUAAAUUUCUUUUACUUU | Cleavage |  | 1 |
| sbi-miR437k | SbNFY-B18 | 5 | -1 | 1 | 21 | 979 | 999 | AAAGUUAGAGAAGUUUGACUU | GCUACAAAGUUCUAUAACUUU | Cleavage |  | 1 |
| sbi-miR437k | SbNFY-B19 | 5 | -1 | 1 | 21 | 10713 | 10733 | AAAGUUAGAGAAGUUUGACUU | UCCUUACAUUUUUCUAAGUUU | Cleavage |  | 1 |
| sbi-miR437l | SbNFY-B11 | 5 | -1 | 1 | 21 | 12561 | 12581 | AAAGUUAGAGAAGUUUGACUU | AAGUAGAACGUCUCUACUUUU | Cleavage |  | 1 |
| sbi-miR437l | SbNFY-B17 | 5 | -1 | 1 | 21 | 5202 | 5222 | AAAGUUAGAGAAGUUUGACUU | AACGCGAACUUCUCUAGUUCU | Cleavage |  | 1 |
| sbi-miR437l | SbNFY-B15 | 5 | -1 | 1 | 21 | 14402 | 14422 | AAAGUUAGAGAAGUUUGACUU | GAGUGGAGUUUUUUUUACUUU | Cleavage |  | 1 |
| sbi-miR437l | SbNFY-B1 | 5 | -1 | 1 | 21 | 3109 | 3129 | AAAGUUAGAGAAGUUUGACUU | AGCAUAAAUUUCUUUUACUUU | Cleavage |  | 1 |
| sbi-miR437l | SbNFY-B18 | 5 | -1 | 1 | 21 | 979 | 999 | AAAGUUAGAGAAGUUUGACUU | GCUACAAAGUUCUAUAACUUU | Cleavage |  | 1 |
| sbi-miR437l | SbNFY-B19 | 5 | -1 | 1 | 21 | 10713 | 10733 | AAAGUUAGAGAAGUUUGACUU | UCCUUACAUUUUUCUAAGUUU | Cleavage |  | 1 |
| sbi-miR437m | SbNFY-B11 | 5 | -1 | 1 | 21 | 12561 | 12581 | AAAGUUAGAGAAGUUUGACUU | AAGUAGAACGUCUCUACUUUU | Cleavage |  | 1 |
| sbi-miR437m | SbNFY-B17 | 5 | -1 | 1 | 21 | 5202 | 5222 | AAAGUUAGAGAAGUUUGACUU | AACGCGAACUUCUCUAGUUCU | Cleavage |  | 1 |
| sbi-miR437m | SbNFY-B15 | 5 | -1 | 1 | 21 | 14402 | 14422 | AAAGUUAGAGAAGUUUGACUU | GAGUGGAGUUUUUUUUACUUU | Cleavage |  | 1 |
| sbi-miR437m | SbNFY-B1 | 5 | -1 | 1 | 21 | 3109 | 3129 | AAAGUUAGAGAAGUUUGACUU | AGCAUAAAUUUCUUUUACUUU | Cleavage |  | 1 |
| sbi-miR437m | SbNFY-B18 | 5 | -1 | 1 | 21 | 979 | 999 | AAAGUUAGAGAAGUUUGACUU | GCUACAAAGUUCUAUAACUUU | Cleavage |  | 1 |
| sbi-miR437m | SbNFY-B19 | 5 | -1 | 1 | 21 | 10713 | 10733 | AAAGUUAGAGAAGUUUGACUU | UCCUUACAUUUUUCUAAGUUU | Cleavage |  | 1 |
| sbi-miR437n | SbNFY-B11 | 5 | -1 | 1 | 21 | 12561 | 12581 | AAAGUUAGAGAAGUUUGACUU | AAGUAGAACGUCUCUACUUUU | Cleavage |  | 1 |
| sbi-miR437n | SbNFY-B17 | 5 | -1 | 1 | 21 | 5202 | 5222 | AAAGUUAGAGAAGUUUGACUU | AACGCGAACUUCUCUAGUUCU | Cleavage |  | 1 |
| sbi-miR437n | SbNFY-B15 | 5 | -1 | 1 | 21 | 14402 | 14422 | AAAGUUAGAGAAGUUUGACUU | GAGUGGAGUUUUUUUUACUUU | Cleavage |  | 1 |
| sbi-miR437n | SbNFY-B1 | 5 | -1 | 1 | 21 | 3109 | 3129 | AAAGUUAGAGAAGUUUGACUU | AGCAUAAAUUUCUUUUACUUU | Cleavage |  | 1 |
| sbi-miR437n | SbNFY-B18 | 5 | -1 | 1 | 21 | 979 | 999 | AAAGUUAGAGAAGUUUGACUU | GCUACAAAGUUCUAUAACUUU | Cleavage |  | 1 |
| sbi-miR437n | SbNFY-B19 | 5 | -1 | 1 | 21 | 10713 | 10733 | AAAGUUAGAGAAGUUUGACUU | UCCUUACAUUUUUCUAAGUUU | Cleavage |  | 1 |
| sbi-miR437o | SbNFY-B11 | 5 | -1 | 1 | 21 | 12561 | 12581 | AAAGUUAGAGAAGUUUGACUU | AAGUAGAACGUCUCUACUUUU | Cleavage |  | 1 |
| sbi-miR437o | SbNFY-B17 | 5 | -1 | 1 | 21 | 5202 | 5222 | AAAGUUAGAGAAGUUUGACUU | AACGCGAACUUCUCUAGUUCU | Cleavage |  | 1 |
| sbi-miR437o | SbNFY-B15 | 5 | -1 | 1 | 21 | 14402 | 14422 | AAAGUUAGAGAAGUUUGACUU | GAGUGGAGUUUUUUUUACUUU | Cleavage |  | 1 |
| sbi-miR437o | SbNFY-B1 | 5 | -1 | 1 | 21 | 3109 | 3129 | AAAGUUAGAGAAGUUUGACUU | AGCAUAAAUUUCUUUUACUUU | Cleavage |  | 1 |
| sbi-miR437o | SbNFY-B18 | 5 | -1 | 1 | 21 | 979 | 999 | AAAGUUAGAGAAGUUUGACUU | GCUACAAAGUUCUAUAACUUU | Cleavage |  | 1 |
| sbi-miR437o | SbNFY-B19 | 5 | -1 | 1 | 21 | 10713 | 10733 | AAAGUUAGAGAAGUUUGACUU | UCCUUACAUUUUUCUAAGUUU | Cleavage |  | 1 |
| sbi-miR437p | SbNFY-B11 | 5 | -1 | 1 | 21 | 12561 | 12581 | AAAGUUAGAGAAGUUUGACUU | AAGUAGAACGUCUCUACUUUU | Cleavage |  | 1 |
| sbi-miR437p | SbNFY-B17 | 5 | -1 | 1 | 21 | 5202 | 5222 | AAAGUUAGAGAAGUUUGACUU | AACGCGAACUUCUCUAGUUCU | Cleavage |  | 1 |
| sbi-miR437p | SbNFY-B15 | 5 | -1 | 1 | 21 | 14402 | 14422 | AAAGUUAGAGAAGUUUGACUU | GAGUGGAGUUUUUUUUACUUU | Cleavage |  | 1 |
| sbi-miR437p | SbNFY-B1 | 5 | -1 | 1 | 21 | 3109 | 3129 | AAAGUUAGAGAAGUUUGACUU | AGCAUAAAUUUCUUUUACUUU | Cleavage |  | 1 |
| sbi-miR437p | SbNFY-B18 | 5 | -1 | 1 | 21 | 979 | 999 | AAAGUUAGAGAAGUUUGACUU | GCUACAAAGUUCUAUAACUUU | Cleavage |  | 1 |
| sbi-miR437p | SbNFY-B19 | 5 | -1 | 1 | 21 | 10713 | 10733 | AAAGUUAGAGAAGUUUGACUU | UCCUUACAUUUUUCUAAGUUU | Cleavage |  | 1 |
| sbi-miR437q | SbNFY-B11 | 5 | -1 | 1 | 21 | 12561 | 12581 | AAAGUUAGAGAAGUUUGACUU | AAGUAGAACGUCUCUACUUUU | Cleavage |  | 1 |
| sbi-miR437q | SbNFY-B17 | 5 | -1 | 1 | 21 | 5202 | 5222 | AAAGUUAGAGAAGUUUGACUU | AACGCGAACUUCUCUAGUUCU | Cleavage |  | 1 |
| sbi-miR437q | SbNFY-B15 | 5 | -1 | 1 | 21 | 14402 | 14422 | AAAGUUAGAGAAGUUUGACUU | GAGUGGAGUUUUUUUUACUUU | Cleavage |  | 1 |
| sbi-miR437q | SbNFY-B1 | 5 | -1 | 1 | 21 | 3109 | 3129 | AAAGUUAGAGAAGUUUGACUU | AGCAUAAAUUUCUUUUACUUU | Cleavage |  | 1 |
| sbi-miR437q | SbNFY-B18 | 5 | -1 | 1 | 21 | 979 | 999 | AAAGUUAGAGAAGUUUGACUU | GCUACAAAGUUCUAUAACUUU | Cleavage |  | 1 |
| sbi-miR437q | SbNFY-B19 | 5 | -1 | 1 | 21 | 10713 | 10733 | AAAGUUAGAGAAGUUUGACUU | UCCUUACAUUUUUCUAAGUUU | Cleavage |  | 1 |
| sbi-miR437r | SbNFY-B11 | 5 | -1 | 1 | 21 | 12561 | 12581 | AAAGUUAGAGAAGUUUGACUU | AAGUAGAACGUCUCUACUUUU | Cleavage |  | 1 |
| sbi-miR437r | SbNFY-B17 | 5 | -1 | 1 | 21 | 5202 | 5222 | AAAGUUAGAGAAGUUUGACUU | AACGCGAACUUCUCUAGUUCU | Cleavage |  | 1 |
| sbi-miR437r | SbNFY-B15 | 5 | -1 | 1 | 21 | 14402 | 14422 | AAAGUUAGAGAAGUUUGACUU | GAGUGGAGUUUUUUUUACUUU | Cleavage |  | 1 |
| sbi-miR437r | SbNFY-B1 | 5 | -1 | 1 | 21 | 3109 | 3129 | AAAGUUAGAGAAGUUUGACUU | AGCAUAAAUUUCUUUUACUUU | Cleavage |  | 1 |
| sbi-miR437r | SbNFY-B18 | 5 | -1 | 1 | 21 | 979 | 999 | AAAGUUAGAGAAGUUUGACUU | GCUACAAAGUUCUAUAACUUU | Cleavage |  | 1 |
| sbi-miR437r | SbNFY-B19 | 5 | -1 | 1 | 21 | 10713 | 10733 | AAAGUUAGAGAAGUUUGACUU | UCCUUACAUUUUUCUAAGUUU | Cleavage |  | 1 |
| sbi-miR437s | SbNFY-B11 | 5 | -1 | 1 | 21 | 12561 | 12581 | AAAGUUAGAGAAGUUUGACUU | AAGUAGAACGUCUCUACUUUU | Cleavage |  | 1 |
| sbi-miR437s | SbNFY-B17 | 5 | -1 | 1 | 21 | 5202 | 5222 | AAAGUUAGAGAAGUUUGACUU | AACGCGAACUUCUCUAGUUCU | Cleavage |  | 1 |
| sbi-miR437s | SbNFY-B15 | 5 | -1 | 1 | 21 | 14402 | 14422 | AAAGUUAGAGAAGUUUGACUU | GAGUGGAGUUUUUUUUACUUU | Cleavage |  | 1 |
| sbi-miR437s | SbNFY-B1 | 5 | -1 | 1 | 21 | 3109 | 3129 | AAAGUUAGAGAAGUUUGACUU | AGCAUAAAUUUCUUUUACUUU | Cleavage |  | 1 |
| sbi-miR437s | SbNFY-B18 | 5 | -1 | 1 | 21 | 979 | 999 | AAAGUUAGAGAAGUUUGACUU | GCUACAAAGUUCUAUAACUUU | Cleavage |  | 1 |
| sbi-miR437s | SbNFY-B19 | 5 | -1 | 1 | 21 | 10713 | 10733 | AAAGUUAGAGAAGUUUGACUU | UCCUUACAUUUUUCUAAGUUU | Cleavage |  | 1 |
| sbi-miR437t | SbNFY-B11 | 5 | -1 | 1 | 21 | 12561 | 12581 | AAAGUUAGAGAAGUUUGACUU | AAGUAGAACGUCUCUACUUUU | Cleavage |  | 1 |
| sbi-miR437t | SbNFY-B17 | 5 | -1 | 1 | 21 | 5202 | 5222 | AAAGUUAGAGAAGUUUGACUU | AACGCGAACUUCUCUAGUUCU | Cleavage |  | 1 |
| sbi-miR437t | SbNFY-B15 | 5 | -1 | 1 | 21 | 14402 | 14422 | AAAGUUAGAGAAGUUUGACUU | GAGUGGAGUUUUUUUUACUUU | Cleavage |  | 1 |
| sbi-miR437t | SbNFY-B1 | 5 | -1 | 1 | 21 | 3109 | 3129 | AAAGUUAGAGAAGUUUGACUU | AGCAUAAAUUUCUUUUACUUU | Cleavage |  | 1 |
| sbi-miR437t | SbNFY-B18 | 5 | -1 | 1 | 21 | 979 | 999 | AAAGUUAGAGAAGUUUGACUU | GCUACAAAGUUCUAUAACUUU | Cleavage |  | 1 |
| sbi-miR437t | SbNFY-B19 | 5 | -1 | 1 | 21 | 10713 | 10733 | AAAGUUAGAGAAGUUUGACUU | UCCUUACAUUUUUCUAAGUUU | Cleavage |  | 1 |
| sbi-miR437u | SbNFY-B11 | 5 | -1 | 1 | 21 | 12561 | 12581 | AAAGUUAGAGAAGUUUGACUU | AAGUAGAACGUCUCUACUUUU | Cleavage |  | 1 |
| sbi-miR437u | SbNFY-B17 | 5 | -1 | 1 | 21 | 5202 | 5222 | AAAGUUAGAGAAGUUUGACUU | AACGCGAACUUCUCUAGUUCU | Cleavage |  | 1 |
| sbi-miR437u | SbNFY-B15 | 5 | -1 | 1 | 21 | 14402 | 14422 | AAAGUUAGAGAAGUUUGACUU | GAGUGGAGUUUUUUUUACUUU | Cleavage |  | 1 |
| sbi-miR437u | SbNFY-B1 | 5 | -1 | 1 | 21 | 3109 | 3129 | AAAGUUAGAGAAGUUUGACUU | AGCAUAAAUUUCUUUUACUUU | Cleavage |  | 1 |
| sbi-miR437u | SbNFY-B18 | 5 | -1 | 1 | 21 | 979 | 999 | AAAGUUAGAGAAGUUUGACUU | GCUACAAAGUUCUAUAACUUU | Cleavage |  | 1 |
| sbi-miR437u | SbNFY-B19 | 5 | -1 | 1 | 21 | 10713 | 10733 | AAAGUUAGAGAAGUUUGACUU | UCCUUACAUUUUUCUAAGUUU | Cleavage |  | 1 |
| sbi-miR437v | SbNFY-B11 | 5 | -1 | 1 | 21 | 12561 | 12581 | AAAGUUAGAGAAGUUUGACUU | AAGUAGAACGUCUCUACUUUU | Cleavage |  | 1 |
| sbi-miR437v | SbNFY-B17 | 5 | -1 | 1 | 21 | 5202 | 5222 | AAAGUUAGAGAAGUUUGACUU | AACGCGAACUUCUCUAGUUCU | Cleavage |  | 1 |
| sbi-miR437v | SbNFY-B15 | 5 | -1 | 1 | 21 | 14402 | 14422 | AAAGUUAGAGAAGUUUGACUU | GAGUGGAGUUUUUUUUACUUU | Cleavage |  | 1 |
| sbi-miR437v | SbNFY-B1 | 5 | -1 | 1 | 21 | 3109 | 3129 | AAAGUUAGAGAAGUUUGACUU | AGCAUAAAUUUCUUUUACUUU | Cleavage |  | 1 |
| sbi-miR437v | SbNFY-B18 | 5 | -1 | 1 | 21 | 979 | 999 | AAAGUUAGAGAAGUUUGACUU | GCUACAAAGUUCUAUAACUUU | Cleavage |  | 1 |
| sbi-miR437v | SbNFY-B19 | 5 | -1 | 1 | 21 | 10713 | 10733 | AAAGUUAGAGAAGUUUGACUU | UCCUUACAUUUUUCUAAGUUU | Cleavage |  | 1 |
| sbi-miR437w | SbNFY-B11 | 5 | -1 | 1 | 21 | 12561 | 12581 | AAAGUUAGAGAAGUUUGACUU | AAGUAGAACGUCUCUACUUUU | Cleavage |  | 1 |
| sbi-miR437w | SbNFY-B17 | 5 | -1 | 1 | 21 | 5202 | 5222 | AAAGUUAGAGAAGUUUGACUU | AACGCGAACUUCUCUAGUUCU | Cleavage |  | 1 |
| sbi-miR437w | SbNFY-B15 | 5 | -1 | 1 | 21 | 14402 | 14422 | AAAGUUAGAGAAGUUUGACUU | GAGUGGAGUUUUUUUUACUUU | Cleavage |  | 1 |
| sbi-miR437w | SbNFY-B1 | 5 | -1 | 1 | 21 | 3109 | 3129 | AAAGUUAGAGAAGUUUGACUU | AGCAUAAAUUUCUUUUACUUU | Cleavage |  | 1 |
| sbi-miR437w | SbNFY-B18 | 5 | -1 | 1 | 21 | 979 | 999 | AAAGUUAGAGAAGUUUGACUU | GCUACAAAGUUCUAUAACUUU | Cleavage |  | 1 |
| sbi-miR437w | SbNFY-B19 | 5 | -1 | 1 | 21 | 10713 | 10733 | AAAGUUAGAGAAGUUUGACUU | UCCUUACAUUUUUCUAAGUUU | Cleavage |  | 1 |
| sbi-miR437x-3p | SbNFY-B7 | 5 | -1 | 1 | 24 | 14250 | 14273 | AUUUGACUGACACGGAUUCUAGGA | UCCUAGAAUUCAUAUCGGUCAAAG | Translation | | 1 |
| sbi-miR437x-3p | SbNFY-B16 | 5 | -1 | 1 | 24 | 9628 | 9651 | AUUUGACUGACACGGAUUCUAGGA | CAUCUGGAUGCGUGUUGGUUGAGA | Cleavage |  | 1 |
| sbi-miR437x-3p | SbNFY-B8 | 5 | -1 | 1 | 24 | 5758 | 5781 | AUUUGACUGACACGGAUUCUAGGA | UCGUCGACUCUGUGUUAGUCUCAU | Cleavage |  | 1 |
| sbi-miR437x-3p | SbNFY-B9 | 5 | -1 | 1 | 24 | 7345 | 7368 | AUUUGACUGACACGGAUUCUAGGA | CUACAGGGACUGUGUCAGCCAAAG | Cleavage |  | 1 |
| sbi-miR437x-3p | SbNFY-B10 | 5 | -1 | 1 | 24 | 12208 | 12231 | AUUUGACUGACACGGAUUCUAGGA | UCCCUGAAAGCGUAUCAGUCAAUU | Translation | | 1 |
| sbi-miR528 | SbNFY-B1 | 5 | -1 | 1 | 21 | 5233 | 5253 | UGGAAGGGGCAUGCAGAGGAG | CACCGUUGCUUGCUCCUUCGA | Cleavage |  | 2 |
| sbi-miR528 | SbNFY-B2 | 5 | -1 | 1 | 21 | 4462 | 4482 | UGGAAGGGGCAUGCAGAGGAG | UUUCUUUUCAUGUUCUUUCUC | Cleavage |  | 2 |
| sbi-miR528 | SbNFY-B4 | 5 | -1 | 1 | 21 | 13187 | 13207 | UGGAAGGGGCAUGCAGAGGAG | UUCUACUGAAUGUUCCUUCUG | Cleavage |  | 1 |
| sbi-miR528 | SbNFY-B11 | 5 | -1 | 1 | 21 | 525 | 545 | UGGAAGGGGCAUGCAGAGGAG | UGAUUCCGCAAGCCCCUUCCC | Translation | | 1 |
| sbi-miR528 | SbNFY-B8 | 5 | -1 | 1 | 21 | 12494 | 12514 | UGGAAGGGGCAUGCAGAGGAG | GCAUUCUCUAUGCUUCUUCCC | Cleavage |  | 1 |
| sbi-miR529 | SbNFY-B11 | 5 | -1 | 1 | 20 | 14855 | 14874 | CUGUACCCUCUCUCUUCUUC | GAGGGACAGAGGGAGUGCGG | Cleavage |  | 1 |
| sbi-miR529 | SbNFY-B12 | 5 | -1 | 1 | 20 | 14164 | 14183 | CUGUACCCUCUCUCUUCUUC | UUGGAAAGGAGGGAGUACAG | Cleavage |  | 2 |
| sbi-miR529 | SbNFY-B14 | 5 | -1 | 1 | 20 | 16120 | 16139 | CUGUACCCUCUCUCUUCUUC | UCAGACGAGAGGAGGUAUGG | Cleavage |  | 1 |
| sbi-miR5383 | SbNFY-B16 | 5 | -1 | 1 | 24 | 4288 | 4311 | AUGACAGAGCUCCGGCAGAGAUAU | CGUGAUCUGCUGGCGUUUCGUUAU | Translation | | 1 |
| sbi-miR5386 | SbNFY-B16 | 5 | -1 | 1 | 20 | 8099 | 8119 | CGUCGCUGUCGCG-CGCGCUG | CGGCGCGGCGCGGCGGUGACG | Cleavage |  | 3 |
| sbi-miR5386 | SbNFY-B16 | 5 | -1 | 1 | 20 | 8856 | 8875 | CGUCGCUGUCGCGCGCGCUG | GGCCGCCUGCGGCGGCGGCG | Cleavage |  | 3 |
| sbi-miR5386 | SbNFY-B10 | 5 | -1 | 1 | 20 | 9372 | 9390 | CGUCGCUGUCGCGCGCGCUG | CGGCG-GUGCGGCAGCGUCG | Cleavage |  | 3 |
| sbi-miR5386 | SbNFY-B13 | 5 | -1 | 1 | 20 | 2518 | 2537 | CGUCGCUGUCGCGCGCGCUG | CAGCGAGAGAGGCAGCGGCG | Translation | | 1 |
| sbi-miR5386 | SbNFY-B11 | 5 | -1 | 1 | 20 | 13646 | 13664 | CGUCGCUGUCGCGCGCGCUG | CUGCGCG-GCGGCAGCGAUG | Cleavage |  | 1 |
| sbi-miR5387a | SbNFY-B15 | 5 | -1 | 1 | 25 | 2907 | 2931 | UAACACGAACCGGUGCUAAAGGAUC | CUUCUGUUGGCAUCUGUUCAUGUUU | Translation | | 1 |
| sbi-miR5388 | SbNFY-B19 | 5 | -1 | 1 | 22 | 870 | 891 | AUCUUUGCCGGGUGUCUCUGAC | CUCCGUGACGUCCGGCAUGGAU | Cleavage |  | 2 |
| sbi-miR5388 | SbNFY-B19 | 5 | -1 | 1 | 22 | 6519 | 6540 | AUCUUUGCCGGGUGUCUCUGAC | CUCCGUGACGUCCGGCACGGAU | Cleavage |  | 2 |
| sbi-miR5388 | SbNFY-B8 | 5 | -1 | 1 | 22 | 838 | 859 | AUCUUUGCCGGGUGUCUCUGAC | AGUAGAGAGAAUCGGCAAGGGG | Cleavage |  | 1 |
| sbi-miR5389 | SbNFY-B8 | 5 | -1 | 1 | 21 | 6815 | 6835 | GCUUGAGUUUAUCAGCCGAGU | GCUUGGAUGAUGGACGCAAGG | Cleavage |  | 1 |
| sbi-miR5389 | SbNFY-B11 | 5 | -1 | 1 | 21 | 3465 | 3485 | GCUUGAGUUUAUCAGCCGAGU | AGUUGGCUGUUGGACUUGAGG | Cleavage |  | 1 |
| sbi-miR5389 | SbNFY-B15 | 5 | -1 | 1 | 21 | 1539 | 1559 | GCUUGAGUUUAUCAGCCGAGU | AAUUGACAGAUAAAUUGGAGC | Cleavage |  | 1 |
| sbi-miR5564b | SbNFY-B8 | 5 | -1 | 1 | 21 | 11739 | 11759 | GCAAUUCGUCGAACAGCUUGA | UUAGAUUUUUCUAUGAAUUGC | Translation | | 1 |
| sbi-miR5564b | SbNFY-B2 | 5 | -1 | 1 | 21 | 7362 | 7384 | GCAAUUCGUCGAAC--AGCUUGA | UGAGGUUAGGUUUGGUGAAUUGC | Cleavage |  | 1 |
| sbi-miR5564c-3p | SbNFY-B12 | 5 | -1 | 1 | 21 | 5691 | 5711 | ACGCGAGCUGUUUGGCGAAUU | AAUUUGCCAAACUGUUCGAGA | Cleavage |  | 1 |
| sbi-miR5564c-5p | SbNFY-B4 | 5 | -1 | 1 | 21 | 10243 | 10263 | AAUUCGUCGAACAGCUGCAGC | UCGCCAUGUGUUUGAUGAAUU | Cleavage |  | 1 |
| sbi-miR5565a | SbNFY-B7 | 5 | -1 | 1 | 24 | 549 | 572 | AACACAUGUGGAUUGAGGCGAAUC | UACGUGUCUCGAUCCGUGUCUGUC | Cleavage |  | 1 |
| sbi-miR5565a | SbNFY-B3 | 5 | -1 | 1 | 24 | 7842 | 7865 | AACACAUGUGGAUUGAGGCGAAUC | UGAAUGCCUCGCUUCACAAGUGUG | Cleavage |  | 1 |
| sbi-miR5565a | SbNFY-B8 | 5 | -1 | 1 | 24 | 11687 | 11710 | AACACAUGUGGAUUGAGGCGAAUC | UUACUGCAUGAAUCUACAUAUGUG | Cleavage |  | 2 |
| sbi-miR5565a | SbNFY-B8 | 5 | -1 | 1 | 24 | 11915 | 11938 | AACACAUGUGGAUUGAGGCGAAUC | UUACUGCAUGAAUCUACAUAUGUG | Cleavage |  | 2 |
| sbi-miR5565b | SbNFY-B7 | 5 | -1 | 1 | 24 | 549 | 572 | AACACAUGUGGAUUGAGGCGAAUC | UACGUGUCUCGAUCCGUGUCUGUC | Cleavage |  | 1 |
| sbi-miR5565b | SbNFY-B3 | 5 | -1 | 1 | 24 | 7842 | 7865 | AACACAUGUGGAUUGAGGCGAAUC | UGAAUGCCUCGCUUCACAAGUGUG | Cleavage |  | 1 |
| sbi-miR5565b | SbNFY-B8 | 5 | -1 | 1 | 24 | 11687 | 11710 | AACACAUGUGGAUUGAGGCGAAUC | UUACUGCAUGAAUCUACAUAUGUG | Cleavage |  | 2 |
| sbi-miR5565b | SbNFY-B8 | 5 | -1 | 1 | 24 | 11915 | 11938 | AACACAUGUGGAUUGAGGCGAAUC | UUACUGCAUGAAUCUACAUAUGUG | Cleavage |  | 2 |
| sbi-miR5565c | SbNFY-B3 | 5 | -1 | 1 | 21 | 7845 | 7865 | UACACAUGUGGAUUGAGGUGA | AUGCCUCGCUUCACAAGUGUG | Cleavage |  | 1 |
| sbi-miR5565c | SbNFY-B16 | 5 | -1 | 1 | 21 | 10895 | 10915 | UACACAUGUGGAUUGAGGUGA | AAACACUAAGCUAUAUGUGUA | Cleavage |  | 2 |
| sbi-miR5565c | SbNFY-B8 | 5 | -1 | 1 | 21 | 11690 | 11710 | UACACAUGUGGAUUGAGGUGA | CUGCAUGAAUCUACAUAUGUG | Cleavage |  | 2 |
| sbi-miR5565c | SbNFY-B8 | 5 | -1 | 1 | 21 | 11918 | 11938 | UACACAUGUGGAUUGAGGUGA | CUGCAUGAAUCUACAUAUGUG | Cleavage |  | 2 |
| sbi-miR5565d | SbNFY-B14 | 5 | -1 | 1 | 24 | 11525 | 11548 | ACUUCAAUCCAUGUAUGUUGGUGU | ACCUCAAUCCACGUGUGUUGAAGU | Cleavage |  | 2 |
| sbi-miR5565e | SbNFY-B13 | 5 | -1 | 1 | 19 | 14685 | 14703 | UUGUUUGGAUGUUGUCGGA | CCCCGCAAGAUCCAAACGG | Translation | | 2 |
| sbi-miR5565e | SbNFY-B3 | 5 | -1 | 1 | 19 | 13797 | 13815 | UUGUUUGGAUGUUGUCGGA | GUCGACGACGUCGAGACAG | Cleavage |  | 2 |
| sbi-miR5565e | SbNFY-B11 | 5 | -1 | 1 | 19 | 7831 | 7849 | UUGUUUGGAUGUUGUCGGA | UCUGAAACCCUCUAAACAA | Translation | | 1 |
| sbi-miR5565e | SbNFY-B15 | 5 | -1 | 1 | 19 | 15672 | 15690 | UUGUUUGGAUGUUGUCGGA | UCCAACAGCACUUAAAUAG | Cleavage |  | 1 |
| sbi-miR5565e | SbNFY-B18 | 5 | -1 | 1 | 19 | 13228 | 13246 | UUGUUUGGAUGUUGUCGGA | UCUUGCAAGGUUCGAACAA | Translation | | 1 |
| sbi-miR5565e | SbNFY-B6 | 5 | -1 | 1 | 19 | 4270 | 4288 | UUGUUUGGAUGUUGUCGGA | UCUACCGCCAUCCAAACGA | Cleavage |  | 1 |
| sbi-miR5565e | SbNFY-B9 | 5 | -1 | 1 | 19 | 15416 | 15434 | UUGUUUGGAUGUUGUCGGA | UCUAAAAACAUUCAAGGAA | Cleavage |  | 1 |
| sbi-miR5565f | SbNFY-B19 | 5 | -1 | 1 | 20 | 6062 | 6083 | UAGUCGGAUUUAUA--UCAAUC | CUUUGAACUGUAAAUCUGGCUA | Cleavage |  | 1 |
| sbi-miR5565f | SbNFY-B6 | 5 | -1 | 1 | 20 | 15289 | 15308 | UAGUCGGAUUUAUAUCAAUC | AAUUGAGAUGAGUCUGGUAA | Cleavage |  | 1 |
| sbi-miR5565g-3p | SbNFY-B2 | 5 | -1 | 1 | 24 | 9498 | 9521 | ACACAUGUGGAUUGAGAUGAAUAC | AUGAGAAUGCCAAUCCAUAUGAGU | Cleavage |  | 2 |
| sbi-miR5565g-3p | SbNFY-B2 | 5 | -1 | 1 | 24 | 10137 | 10159 | ACACAUGUGGAUUGAGAUGAAUAC | AUACACUUCGCAAUC-ACAUGUGU | Cleavage |  | 2 |
| sbi-miR5565g-5p | SbNFY-B7 | 5 | -1 | 1 | 24 | 10029 | 10054 | UUCACAUCAAUCCACAU--AUGUUGG | CCCAUGUGGAUGAGGGUUGGUGUGAA | Cleavage |  | 2 |
| sbi-miR5565g-5p | SbNFY-B7 | 5 | -1 | 1 | 24 | 17223 | 17246 | UUCACAUCAAUCCACAUAUGUUGG | UUGCGAAGUGAGGAUCGGUGUGGA | Cleavage |  | 2 |
| sbi-miR5565g-5p | SbNFY-B5 | 5 | -1 | 1 | 24 | 4603 | 4626 | UUCACAUCAAUCCACAUAUGUUGG | AUUAUGAGUGUGGUUUGGUGUGAC | Translation | | 1 |
| sbi-miR5566 | SbNFY-B17 | 5 | -1 | 1 | 21 | 4845 | 4865 | UCAGCAUCACCUCCCUGUUGU | GCAGUAGUGAUCUGAUGCUGA | Translation | | 1 |
| sbi-miR5567 | SbNFY-B6 | 5 | -1 | 1 | 24 | 8564 | 8588 | UUAAUGAUU-CAUGUAUGUGUCCAA | GAAGGCAUAUAUAUGCAAUUAUUGA | Cleavage |  | 5 |
| sbi-miR5567 | SbNFY-B7 | 5 | -1 | 1 | 24 | 12203 | 12226 | UUAAUGAUUCAUGUAUGUGUCCAA | UUAGAGGCAUGUAUGGAGUAUUAA | Cleavage |  | 4 |
| sbi-miR5567 | SbNFY-B5 | 5 | -1 | 1 | 24 | 16152 | 16175 | UUAAUGAUUCAUGUAUGUGUCCAA | UUUCUCAUAAACAUUGAUCAUUUA | Translation | | 5 |
| sbi-miR5567 | SbNFY-B10 | 5 | -1 | 1 | 24 | 8247 | 8270 | UUAAUGAUUCAUGUAUGUGUCCAA | UUAGACGCAUGCAUUGAGUAUUAA | Translation | | 6 |
| sbi-miR5567 | SbNFY-B14 | 5 | -1 | 1 | 24 | 16387 | 16410 | UUAAUGAUUCAUGUAUGUGUCCAA | UUGGAUAUAUGCAUAAAGUAUUAA | Translation | | 1 |
| sbi-miR5567 | SbNFY-B9 | 5 | -1 | 1 | 24 | 8502 | 8525 | UUAAUGAUUCAUGUAUGUGUCCAA | UUCAGUAUAUAGAUAGAUUAUUAA | Translation | | 5 |
| sbi-miR5567 | SbNFY-B18 | 5 | -1 | 1 | 24 | 15759 | 15782 | UUAAUGAUUCAUGUAUGUGUCCAA | UUGGACAUAUGUAUGAAGUACUAA | Cleavage |  | 7 |
| sbi-miR5567 | SbNFY-B18 | 5 | -1 | 1 | 24 | 555 | 578 | UUAAUGAUUCAUGUAUGUGUCCAA | GGGCACACCUGCAUGGACCAUUGU | Cleavage |  | 7 |
| sbi-miR5567 | SbNFY-B18 | 5 | -1 | 1 | 24 | 8387 | 8410 | UUAAUGAUUCAUGUAUGUGUCCAA | AAUAACAAAUACAAGAAUAAUUAC | Translation | | 7 |
| sbi-miR5567 | SbNFY-B12 | 5 | -1 | 1 | 24 | 15927 | 15950 | UUAAUGAUUCAUGUAUGUGUCCAA | UCAGAUGUGUACGUGAAUCAUUUC | Cleavage |  | 1 |
| sbi-miR5567 | SbNFY-B15 | 5 | -1 | 1 | 24 | 1557 | 1580 | UUAAUGAUUCAUGUAUGUGUCCAA | AGCAACAAAUGCAUGAUCCAUUAG | Cleavage |  | 2 |
| sbi-miR5567 | SbNFY-B1 | 5 | -1 | 1 | 24 | 4248 | 4271 | UUAAUGAUUCAUGUAUGUGUCCAA | AUGUUUAUCGAUGUGAAUUAUUAG | Cleavage |  | 1 |
| sbi-miR5568a | SbNFY-B9 | 5 | -1 | 1 | 21 | 16540 | 16560 | CAGAGCGACUUACAAUUUGGA | UCCGAGUUCUGGGUCGCUUUU | Cleavage |  | 1 |
| sbi-miR5568a | SbNFY-B14 | 5 | -1 | 1 | 21 | 6520 | 6540 | CAGAGCGACUUACAAUUUGGA | UCUAAAUUAUAAGUUAUUUUG | Cleavage |  | 1 |
| sbi-miR5568a | SbNFY-B18 | 5 | -1 | 1 | 21 | 8711 | 8731 | CAGAGCGACUUACAAUUUGGA | AUUAGGUUGUUAGUCGUUUUC | Translation | | 1 |
| sbi-miR5568a | SbNFY-B11 | 5 | -1 | 1 | 21 | 12677 | 12697 | CAGAGCGACUUACAAUUUGGA | UGUAAAUUGUAGCUCGUGUUG | Cleavage |  | 1 |
| sbi-miR5568b-3p | SbNFY-B3 | 5 | -1 | 1 | 21 | 9996 | 10015 | ACUAUGUAUCUAGAAAAGCUA | UAGCUUUUAUA-AUAUAUAGU | Translation | | 1 |
| sbi-miR5568b-3p | SbNFY-B19 | 5 | -1 | 1 | 21 | 17638 | 17658 | ACUAUGUAUCUAGAAAAGCUA | UAGCUUUCUUAAAUAUAUAAU | Translation | | 1 |
| sbi-miR5568b-3p | SbNFY-B6 | 5 | -1 | 1 | 21 | 3210 | 3230 | ACUAUGUAUCUAGAAAAGCUA | UGGCAUUUCUAAAUCCAUAGC | Translation | | 1 |
| sbi-miR5568b-3p | SbNFY-B1 | 5 | -1 | 1 | 21 | 14186 | 14206 | ACUAUGUAUCUAGAAAAGCUA | UCCAAAUUCUGGAUAUAUAGU | Cleavage |  | 1 |
| sbi-miR5568b-5p | SbNFY-B8 | 5 | -1 | 1 | 21 | 12354 | 12374 | UUUCUAGGUACAUAGCUUUUG | UUUUUGCUAUUUAUCUAGAAA | Translation | | 2 |
| sbi-miR5568b-5p | SbNFY-B3 | 5 | -1 | 1 | 21 | 6052 | 6071 | UUUCUAGGUACAUAGCUUUUG | GAAUAGC-AUAUAUCUAGAAA | Translation | | 2 |
| sbi-miR5568b-5p | SbNFY-B12 | 5 | -1 | 1 | 21 | 14108 | 14128 | UUUCUAGGUACAUAGCUUUUG | AAUUUGCUAUGUAUCUAGACA | Cleavage |  | 1 |
| sbi-miR5568b-5p | SbNFY-B14 | 5 | -1 | 1 | 21 | 13336 | 13356 | UUUCUAGGUACAUAGCUUUUG | CGAUAUCGAUGUCCCUAGAAG | Cleavage |  | 1 |
| sbi-miR5568c-3p | SbNFY-B19 | 5 | -1 | 1 | 21 | 17612 | 17632 | ACUUACAGUUUGGAACGGAGG | CUUCUCUUCUAAAUUAUAAGA | Cleavage |  | 1 |
| sbi-miR5568c-5p | SbNFY-B3 | 5 | -1 | 1 | 21 | 11053 | 11073 | UCUGUUCCAAAUUGUAAGUCG | CCAUUUGCGAUUUGGCAAGGA | Cleavage |  | 4 |
| sbi-miR5568c-5p | SbNFY-B4 | 5 | -1 | 1 | 21 | 5430 | 5450 | UCUGUUCCAAAUUGUAAGUCG | AGACCUGCAAUUUAGAAUAAA | Cleavage |  | 3 |
| sbi-miR5568c-5p | SbNFY-B14 | 5 | -1 | 1 | 21 | 9965 | 9985 | UCUGUUCCAAAUUGUAAGUCG | CGACUUAUAAUUCGGAACGAU | Cleavage |  | 2 |
| sbi-miR5568c-5p | SbNFY-B1 | 5 | -1 | 1 | 21 | 10435 | 10455 | UCUGUUCCAAAUUGUAAGUCG | UUACUUGCUAUUUGGGACAAU | Cleavage |  | 1 |
| sbi-miR5568c-5p | SbNFY-B13 | 5 | -1 | 1 | 21 | 5062 | 5082 | UCUGUUCCAAAUUGUAAGUCG | ACAAUCACAGUUAGGAAUAGG | Cleavage |  | 1 |
| sbi-miR5568d-3p | SbNFY-B11 | 5 | -1 | 1 | 21 | 6880 | 6900 | AAAGUUGUGUAUCUAGAAAAG | CUUUUCUGUUUACACGACUUG | Cleavage |  | 1 |
| sbi-miR5568d-3p | SbNFY-B5 | 5 | -1 | 1 | 21 | 4642 | 4662 | AAAGUUGUGUAUCUAGAAAAG | GGUUGCGUGAUGCGCAGUUUU | Cleavage |  | 2 |
| sbi-miR5568d-3p | SbNFY-B14 | 5 | -1 | 1 | 21 | 9918 | 9938 | AAAGUUGUGUAUCUAGAAAAG | UAUGUCUAGAUAUAUAAUUGA | Cleavage |  | 1 |
| sbi-miR5568d-3p | SbNFY-B15 | 5 | -1 | 1 | 21 | 1137 | 1157 | AAAGUUGUGUAUCUAGAAAAG | UAAUUCUGGACAUGCAGUUUU | Translation | | 1 |
| sbi-miR5568d-3p | SbNFY-B3 | 5 | -1 | 1 | 21 | 12777 | 12797 | AAAGUUGUGUAUCUAGAAAAG | AAUUCUUAGUUACAAAAUUUU | Cleavage |  | 1 |
| sbi-miR5568d-5p | SbNFY-B17 | 5 | -1 | 1 | 21 | 17281 | 17301 | UGGCUUUUCUAGAUACAUAGC | GUUGUACAUCUUGAAAAGUUA | Translation | | 2 |
| sbi-miR5568d-5p | SbNFY-B17 | 5 | -1 | 1 | 21 | 16230 | 16250 | UGGCUUUUCUAGAUACAUAGC | UUUGUACAUCUCGAAAAGUUA | Translation | | 2 |
| sbi-miR5568d-5p | SbNFY-B3 | 5 | -1 | 1 | 21 | 6056 | 6076 | UGGCUUUUCUAGAUACAUAGC | AGCAUAUAUCUAGAAAACCAA | Cleavage |  | 1 |
| sbi-miR5568d-5p | SbNFY-B9 | 5 | -1 | 1 | 21 | 3859 | 3879 | UGGCUUUUCUAGAUACAUAGC | CUUAAAUUUUUAGAGAAGCCC | Cleavage |  | 1 |
| sbi-miR5568e-3p | SbNFY-B16 | 5 | -1 | 1 | 21 | 13335 | 13355 | UAUCUAGAAAAGCUAAAACGU | CAGUUUGGGCUUAUUUGGAUU | Cleavage |  | 1 |
| sbi-miR5568e-5p | SbNFY-B2 | 5 | -1 | 1 | 21 | 601 | 621 | GAUGUUUUGGGUUUUCUAGAU | AGAUAGAAAGUUUGAACCAUC | Cleavage |  | 2 |
| sbi-miR5568e-5p | SbNFY-B16 | 5 | -1 | 1 | 21 | 5131 | 5151 | GAUGUUUUGGGUUUUCUAGAU | UGACGGAGGAUCUAAAACAUU | Cleavage |  | 1 |
| sbi-miR5568e-5p | SbNFY-B11 | 5 | -1 | 1 | 21 | 6887 | 6907 | GAUGUUUUGGGUUUUCUAGAU | GUUUACACGACUUGAAACAUU | Cleavage |  | 1 |
| sbi-miR5568f-3p | SbNFY-B3 | 5 | -1 | 1 | 21 | 9886 | 9906 | GUCUUAUAAUUUGGAAUGGAG | GAUAAUUUUAAAUUUUAAGAU | Cleavage |  | 3 |
| sbi-miR5568f-3p | SbNFY-B3 | 5 | -1 | 1 | 21 | 16966 | 16986 | GUCUUAUAAUUUGGAAUGGAG | AUAAAUUUCAAAUUUUGAGAU | Cleavage |  | 3 |
| sbi-miR5568f-3p | SbNFY-B10 | 5 | -1 | 1 | 21 | 2309 | 2329 | GUCUUAUAAUUUGGAAUGGAG | AUCUAUUCUAAAUUAUUAGCA | Cleavage |  | 2 |
| sbi-miR5568f-3p | SbNFY-B1 | 5 | -1 | 1 | 21 | 10229 | 10249 | GUCUUAUAAUUUGGAAUGGAG | ACCCAUUUCAAAUUAUAGUCC | Cleavage |  | 1 |
| sbi-miR5568f-3p | SbNFY-B11 | 5 | -1 | 1 | 21 | 8815 | 8835 | GUCUUAUAAUUUGGAAUGGAG | CUUCAGUCUGGUUUAUAAGGC | Translation | | 1 |
| sbi-miR5568f-3p | SbNFY-B5 | 5 | -1 | 1 | 21 | 619 | 639 | GUCUUAUAAUUUGGAAUGGAG | CUCAGUUCUAAAUUAGAGGGU | Cleavage |  | 1 |
| sbi-miR5568f-3p | SbNFY-B9 | 5 | -1 | 1 | 21 | 15657 | 15677 | GUCUUAUAAUUUGGAAUGGAG | UGGUUUACCAAAUUAUGAGAU | Cleavage |  | 1 |
| sbi-miR5568f-5p | SbNFY-B10 | 5 | -1 | 1 | 21 | 13398 | 13418 | UCCAUUCCAAAUUGUAAGAUG | AGUUUUUGGAUUUUGAGUGGA | Cleavage |  | 2 |
| sbi-miR5568f-5p | SbNFY-B6 | 5 | -1 | 1 | 21 | 15776 | 15796 | UCCAUUCCAAAUUGUAAGAUG | AGUUUUUGGAUUUUGAGUGGA | Cleavage |  | 3 |
| sbi-miR5568f-5p | SbNFY-B3 | 5 | -1 | 1 | 21 | 11366 | 11386 | UCCAUUCCAAAUUGUAAGAUG | UUUUUUAAGACUUGGGGUGGG | Translation | | 4 |
| sbi-miR5568f-5p | SbNFY-B1 | 5 | -1 | 1 | 21 | 15055 | 15075 | UCCAUUCCAAAUUGUAAGAUG | AAUUUUGCAUUUUAGGGUGGA | Cleavage |  | 1 |
| sbi-miR5568f-5p | SbNFY-B7 | 5 | -1 | 1 | 21 | 16148 | 16167 | UCCAUUCCAAAUUGUAAGAUG | UAUUUUA-GAUUUAGGAUGGA | Cleavage |  | 1 |
| sbi-miR5568g-3p | SbNFY-B10 | 5 | -1 | 1 | 21 | 487 | 507 | AAAACGUCUUAUAAUUUGGAG | CUUUGAAUUAUUGGAUGUUUA | Translation | | 2 |
| sbi-miR5568g-3p | SbNFY-B14 | 5 | -1 | 1 | 21 | 6782 | 6802 | AAAACGUCUUAUAAUUUGGAG | AACUAAAAUACAUGAUGUUUU | Translation | | 2 |
| sbi-miR5568g-5p | SbNFY-B6 | 5 | -1 | 1 | 21 | 6892 | 6911 | CAAAUUAUAAGAUGUUUUGGC | UACAAAUUAU-UUGUAAUUUG | Translation | | 3 |
| sbi-miR5568g-5p | SbNFY-B5 | 5 | -1 | 1 | 21 | 17360 | 17380 | CAAAUUAUAAGAUGUUUUGGC | GUAUAGACUUUUUGUAAUUUG | Cleavage |  | 2 |
| sbi-miR5568g-5p | SbNFY-B19 | 5 | -1 | 1 | 21 | 16704 | 16724 | CAAAUUAUAAGAUGUUUUGGC | GACAGAAUAUCUAAAAAUUUU | Cleavage |  | 3 |
| sbi-miR5568g-5p | SbNFY-B4 | 5 | -1 | 1 | 21 | 11974 | 11994 | CAAAUUAUAAGAUGUUUUGGC | AUAUAUACAUUUUAAAAUUUG | Cleavage |  | 2 |
| sbi-miR5568g-5p | SbNFY-B8 | 5 | -1 | 1 | 21 | 8767 | 8787 | CAAAUUAUAAGAUGUUUUGGC | GUCAAAACGAUCUAUAAUUUA | Translation | | 3 |
| sbi-miR5568g-5p | SbNFY-B16 | 5 | -1 | 1 | 21 | 12166 | 12186 | CAAAUUAUAAGAUGUUUUGGC | GAUGAACCAUCUUAAGAUUUA | Cleavage |  | 1 |
| sbi-miR5568g-5p | SbNFY-B11 | 5 | -1 | 1 | 21 | 16809 | 16829 | CAAAUUAUAAGAUGUUUUGGC | AUUAAACUGUUUUAUAGUUCG | Cleavage |  | 1 |
| sbi-miR5568g-5p | SbNFY-B2 | 5 | -1 | 1 | 21 | 4066 | 4086 | CAAAUUAUAAGAUGUUUUGGC | UUCAUAAAACUUUAUGGUUUG | Cleavage |  | 1 |
| sbi-miR5569 | SbNFY-B13 | 5 | -1 | 1 | 24 | 8592 | 8615 | UAUUGCAUGCUUGAACUAUGGUAA | CAAGGAUUGUUCAAGCCAGCAGUG | Cleavage |  | 1 |
| sbi-miR5569 | SbNFY-B3 | 5 | -1 | 1 | 24 | 14111 | 14133 | UAUUGCAUGCUUGAACUAUGGUAA | AAAAUAUA-UACAACUAUGCAAUA | Translation | | 1 |
| sbi-miR5570 | SbNFY-B18 | 5 | -1 | 1 | 21 | 17125 | 17145 | AAAAGACAAAUCAGCAUGUCA | AUACAUGUAGAUUUGUUUGUA | Cleavage |  | 1 |
| sbi-miR5570 | SbNFY-B3 | 5 | -1 | 1 | 21 | 12456 | 12476 | AAAAGACAAAUCAGCAUGUCA | UUAGAUGUUGGUGUGUGUUUU | Cleavage |  | 1 |
| sbi-miR5570 | SbNFY-B11 | 5 | -1 | 1 | 21 | 9605 | 9625 | AAAAGACAAAUCAGCAUGUCA | GAACUUUCUGCUUUGUGUUUU | Translation | | 1 |
| sbi-miR6217a-3p | SbNFY-B10 | 5 | -1 | 1 | 24 | 13235 | 13258 | AAAAUUAUCGUAAAUAGAGGUGGC | CUUUCGUUUGUUUGUGGUAAUUAU | Cleavage |  | 1 |
| sbi-miR6217a-3p | SbNFY-B11 | 5 | -1 | 1 | 24 | 16663 | 16686 | AAAAUUAUCGUAAAUAGAGGUGGC | UUUUCUGUGAUUUACUAUGAUUUU | Cleavage |  | 1 |
| sbi-miR6217a-5p | SbNFY-B5 | 5 | -1 | 1 | 24 | 6984 | 7007 | UAGCCACUUUGAGUUACGAUAAUU | UCGUGUCAUGACUCAACUUGGUUA | Cleavage |  | 1 |
| sbi-miR6217b-3p | SbNFY-B10 | 5 | -1 | 1 | 24 | 13235 | 13258 | AAAAUUAUCGUAAAUAGAGGUGGC | CUUUCGUUUGUUUGUGGUAAUUAU | Cleavage |  | 1 |
| sbi-miR6217b-3p | SbNFY-B11 | 5 | -1 | 1 | 24 | 16663 | 16686 | AAAAUUAUCGUAAAUAGAGGUGGC | UUUUCUGUGAUUUACUAUGAUUUU | Cleavage |  | 1 |
| sbi-miR6217b-5p | SbNFY-B5 | 5 | -1 | 1 | 24 | 6984 | 7007 | UAGCCACUUUGAGUUACGAUAAUU | UCGUGUCAUGACUCAACUUGGUUA | Cleavage |  | 1 |
| sbi-miR6218-3p | SbNFY-B17 | 5 | -1 | 1 | 21 | 16240 | 16260 | ACAAGUUUCGUGAUUUUUGGA | UCGAAAAGUUAUGAAACUUUG | Cleavage |  | 1 |
| sbi-miR6218-3p | SbNFY-B12 | 5 | -1 | 1 | 21 | 2382 | 2402 | ACAAGUUUCGUGAUUUUUGGA | CUCAAUCUUCAUGAAGCUUGG | Cleavage |  | 1 |
| sbi-miR6218-3p | SbNFY-B15 | 5 | -1 | 1 | 21 | 11669 | 11689 | ACAAGUUUCGUGAUUUUUGGA | CAUAAAAUUUGUGUAAUUUGU | Cleavage |  | 2 |
| sbi-miR6218-3p | SbNFY-B19 | 5 | -1 | 1 | 21 | 15023 | 15043 | ACAAGUUUCGUGAUUUUUGGA | AUGAUAAAUCGUGUGACUUGU | Cleavage |  | 1 |
| sbi-miR6218-3p | SbNFY-B14 | 5 | -1 | 1 | 21 | 17514 | 17534 | ACAAGUUUCGUGAUUUUUGGA | CUCAAUGAUUAUUAAGUUUGU | Cleavage |  | 1 |
| sbi-miR6219-3p | SbNFY-B11 | 5 | -1 | 1 | 24 | 1110 | 1133 | AGUCCCGAAACCUUAGUCCCGGCU | UCUUCGGACUUGGGGUUCGUGGCU | Translation | | 1 |
| sbi-miR6220-3p | SbNFY-B3 | 5 | -1 | 1 | 24 | 15854 | 15878 | AUGCCUUAUAA-UUUGGGAUGGAGA | CCUCUAUCCCAAAAUUGUAAGUCAU | Translation | | 1 |
| sbi-miR6220-3p | SbNFY-B14 | 5 | -1 | 1 | 24 | 6512 | 6536 | AUGCCUUAUAAUUU-GGGAUGGAGA | CCUUCAUUUCUAAAUUAUAAGUUAU | Cleavage |  | 1 |
| sbi-miR6220-3p | SbNFY-B11 | 5 | -1 | 1 | 24 | 8814 | 8837 | AUGCCUUAUAAUUUGGGAUGGAGA | GCUUCAGUCUGGUUUAUAAGGCGU | Cleavage |  | 1 |
| sbi-miR6220-3p | SbNFY-B10 | 5 | -1 | 1 | 24 | 5722 | 5745 | AUGCCUUAUAAUUUGGGAUGGAGA | ACCCGAACCCGAAUUAUCGGGUAC | Cleavage |  | 3 |
| sbi-miR6220-3p | SbNFY-B10 | 5 | -1 | 1 | 24 | 10776 | 10799 | AUGCCUUAUAAUUUGGGAUGGAGA | ACCCGAACCCGAAUUAUCGGGUAC | Cleavage |  | 3 |
| sbi-miR6220-3p | SbNFY-B19 | 5 | -1 | 1 | 24 | 17612 | 17635 | AUGCCUUAUAAUUUGGGAUGGAGA | CUUCUCUUCUAAAUUAUAAGACUU | Cleavage |  | 1 |
| sbi-miR6220-3p | SbNFY-B1 | 5 | -1 | 1 | 24 | 10228 | 10251 | AUGCCUUAUAAUUUGGGAUGGAGA | CACCCAUUUCAAAUUAUAGUCCGU | Cleavage |  | 1 |
| sbi-miR6220-3p | SbNFY-B18 | 5 | -1 | 1 | 24 | 1266 | 1289 | AUGCCUUAUAAUUUGGGAUGGAGA | AUGAUGUCCAAAAUUUUACGGUAU | Cleavage |  | 1 |
| sbi-miR6220-5p | SbNFY-B3 | 5 | -1 | 1 | 24 | 11364 | 11387 | CUCCAUCCUAAAUUAUAAGACAUU | UUUUUUUUAAGACUUGGGGUGGGG | Cleavage |  | 3 |
| sbi-miR6220-5p | SbNFY-B1 | 5 | -1 | 1 | 24 | 15053 | 15076 | CUCCAUCCUAAAUUAUAAGACAUU | AAAAUUUUGCAUUUUAGGGUGGAA | Cleavage |  | 1 |
| sbi-miR6221-3p | SbNFY-B3 | 5 | -1 | 1 | 21 | 1900 | 1920 | CCGGGGCCAGAUCUCAGAAGC | UCGGCUGGGAUUUGGCCUCUG | Cleavage |  | 1 |
| sbi-miR6221-3p | SbNFY-B12 | 5 | -1 | 1 | 21 | 1691 | 1711 | CCGGGGCCAGAUCUCAGAAGC | GUUUCUAUGAUCUGGUUUUGU | Cleavage |  | 1 |
| sbi-miR6221-5p | SbNFY-B4 | 5 | -1 | 1 | 21 | 13799 | 13818 | UUCUGACUUCUGGCCCCUGCU | CGCCGGGGCC-GAAGUCGGAG | Translation | | 1 |
| sbi-miR6222-3p | SbNFY-B16 | 5 | -1 | 1 | 21 | 2435 | 2455 | CUAGCUGAUCCAAACAGGCCC | AUGCAUGUGUGGCUCGGUUAG | Cleavage |  | 1 |
| sbi-miR6222-5p | SbNFY-B8 | 5 | -1 | 1 | 21 | 4973 | 4993 | CCUGUUUGGAUCAGCCAAGGC | GCGACGGCCGAUCCAAGCAGG | Cleavage |  | 1 |
| sbi-miR6222-5p | SbNFY-B17 | 5 | -1 | 1 | 21 | 6866 | 6886 | CCUGUUUGGAUCAGCCAAGGC | AGCUUGGUUGAUUAAAGCGCG | Cleavage |  | 1 |
| sbi-miR6223-3p | SbNFY-B6 | 5 | -1 | 1 | 21 | 2839 | 2859 | CUAGCAUGUUCCUCCUAAGAG | UUCAUGGUGGGAGCAUGGUAG | Cleavage |  | 1 |
| sbi-miR6223-3p | SbNFY-B1 | 5 | -1 | 1 | 21 | 11449 | 11469 | CUAGCAUGUUCCUCCUAAGAG | CAGUGAGGAGGGGCAUUUUAG | Cleavage |  | 1 |
| sbi-miR6223-5p | SbNFY-B9 | 5 | -1 | 1 | 21 | 8551 | 8571 | UUCUUGGGAGGAGCAUGCUAG | CGAGCACGAUCCUCAUGAGAA | Cleavage |  | 2 |
| sbi-miR6224a-3p | SbNFY-B11 | 5 | -1 | 1 | 21 | 8813 | 8833 | CUUAUAUACUAGGACGGAGGG | CGCUUCAGUCUGGUUUAUAAG | Cleavage |  | 1 |
| sbi-miR6224a-5p | SbNFY-B4 | 5 | -1 | 1 | 21 | 7364 | 7384 | CUCCGUCCUAAUAUAUAAGGC | GACUUACAAUUUGGGAUGGAG | Cleavage |  | 2 |
| sbi-miR6224a-5p | SbNFY-B3 | 5 | -1 | 1 | 21 | 16086 | 16106 | CUCCGUCCUAAUAUAUAAGGC | GACUUACAAUUUGGGAUGGAG | Cleavage |  | 1 |
| sbi-miR6224a-5p | SbNFY-B7 | 5 | -1 | 1 | 21 | 16149 | 16168 | CUCCGUCCUAAUAUAUAAGGC | AUUUUAGAU-UUAGGAUGGAG | Cleavage |  | 1 |
| sbi-miR6224b-3p | SbNFY-B11 | 5 | -1 | 1 | 21 | 8813 | 8833 | CUUAUAUACUAGGACGGAGGG | CGCUUCAGUCUGGUUUAUAAG | Cleavage |  | 1 |
| sbi-miR6224b-5p | SbNFY-B4 | 5 | -1 | 1 | 21 | 7364 | 7384 | CUCCGUCCUAAUAUAUAAGGC | GACUUACAAUUUGGGAUGGAG | Cleavage |  | 2 |
| sbi-miR6224b-5p | SbNFY-B3 | 5 | -1 | 1 | 21 | 16086 | 16106 | CUCCGUCCUAAUAUAUAAGGC | GACUUACAAUUUGGGAUGGAG | Cleavage |  | 1 |
| sbi-miR6224b-5p | SbNFY-B7 | 5 | -1 | 1 | 21 | 16149 | 16168 | CUCCGUCCUAAUAUAUAAGGC | AUUUUAGAU-UUAGGAUGGAG | Cleavage |  | 1 |
| sbi-miR6224c-3p | SbNFY-B11 | 5 | -1 | 1 | 21 | 8813 | 8833 | CUUAUAUACUAGGACGGAGGG | CGCUUCAGUCUGGUUUAUAAG | Cleavage |  | 1 |
| sbi-miR6224c-5p | SbNFY-B4 | 5 | -1 | 1 | 21 | 7364 | 7384 | CUCCGUCCUAAUAUAUAAGGC | GACUUACAAUUUGGGAUGGAG | Cleavage |  | 2 |
| sbi-miR6224c-5p | SbNFY-B3 | 5 | -1 | 1 | 21 | 16086 | 16106 | CUCCGUCCUAAUAUAUAAGGC | GACUUACAAUUUGGGAUGGAG | Cleavage |  | 1 |
| sbi-miR6224c-5p | SbNFY-B7 | 5 | -1 | 1 | 21 | 16149 | 16168 | CUCCGUCCUAAUAUAUAAGGC | AUUUUAGAU-UUAGGAUGGAG | Cleavage |  | 1 |
| sbi-miR6225-3p | SbNFY-B4 | 5 | -1 | 1 | 24 | 7323 | 7346 | GAAACGAAUCUUUUAAGUCUAAUU | GGUCAAACUUAAAAAGUUUGAUUC | Translation | | 7 |
| sbi-miR6225-3p | SbNFY-B9 | 5 | -1 | 1 | 24 | 13305 | 13328 | GAAACGAAUCUUUUAAGUCUAAUU | AACUAGGCUCAAAAGAUUUAUCUC | Cleavage |  | 1 |
| sbi-miR6225-5p | SbNFY-B10 | 5 | -1 | 1 | 24 | 350 | 372 | AACUAGACUCAAAAGAUUCAUCUC | GAGAU-AAUCUUUUAAAUCUAGUU | Translation | | 5 |
| sbi-miR6225-5p | SbNFY-B2 | 5 | -1 | 1 | 24 | 9125 | 9148 | AACUAGACUCAAAAGAUUCAUCUC | AACAGGCAGCUUUUGACUCCAGUU | Cleavage |  | 2 |
| sbi-miR6226-3p | SbNFY-B16 | 5 | -1 | 1 | 24 | 10678 | 10702 | GAUUAGUCAC-GAUUAGUCGUCCGA | UAGAUAGACUAAUUUGUGACUAAUU | Translation | | 1 |
| sbi-miR6227-5p | SbNFY-B10 | 5 | -1 | 1 | 24 | 6591 | 6614 | GGGCCCAAAUAGCAAGUGUUGUGA | CUUAAAAACGUGCUAUUUCGGCUG | Cleavage |  | 1 |
| sbi-miR6228-3p | SbNFY-B6 | 5 | -1 | 1 | 24 | 10830 | 10853 | GUGGCAGUAGAAUUAAUGAAGGGA | GUUCUUCAUUAAUUAUAUUUUUAU | Translation | | 1 |
| sbi-miR6228-3p | SbNFY-B18 | 5 | -1 | 1 | 24 | 15143 | 15166 | GUGGCAGUAGAAUUAAUGAAGGGA | UGCUUUAGUUAAUUCGACUGUUAA | Cleavage |  | 1 |
| sbi-miR6228-3p | SbNFY-B19 | 5 | -1 | 1 | 24 | 3274 | 3297 | GUGGCAGUAGAAUUAAUGAAGGGA | UAUAUUUAUUUGUUUUGCUGACAU | Cleavage |  | 1 |
| sbi-miR6228-5p | SbNFY-B5 | 5 | -1 | 1 | 24 | 12393 | 12416 | UUCUAUCUCUAUUAAUUGUGUUGC | AUGGAACAUUAAAUAUAGAUAAAA | Cleavage |  | 1 |
| sbi-miR6228-5p | SbNFY-B4 | 5 | -1 | 1 | 24 | 4833 | 4856 | UUCUAUCUCUAUUAAUUGUGUUGC | AUGAAACAUUAAAUAUAGAUAAAA | Cleavage |  | 1 |
| sbi-miR6229-3p | SbNFY-B2 | 5 | -1 | 1 | 24 | 7796 | 7819 | GUUUUUCUCGCCGGGUGAGAAGGC | CCCGGCUCGGUGGGCGGGAAGGAC | Cleavage |  | 1 |
| sbi-miR6229-3p | SbNFY-B8 | 5 | -1 | 1 | 24 | 5267 | 5290 | GUUUUUCUCGCCGGGUGAGAAGGC | CUGGCCUCGCUCGACGGGGGGAAU | Translation | | 1 |
| sbi-miR6229-5p | SbNFY-B17 | 5 | -1 | 1 | 24 | 14768 | 14791 | AUUCUCACUUGGGCGACGGAAAGG | UACUCCCUUCGUCUAAGAAAGAAU | Cleavage |  | 1 |
| sbi-miR6230-3p | SbNFY-B14 | 5 | -1 | 1 | 21 | 9899 | 9919 | UAACAAGUUUAGGGAUCUAGA | GCUAUAUAUUUAGACAUGUUA | Cleavage |  | 1 |
| sbi-miR6230-5p | SbNFY-B3 | 5 | -1 | 1 | 21 | 17588 | 17608 | UUUUGGGUCCCUAAACUUGUU | AAUGCGUUUAGAGGCCUAGAA | Translation | | 2 |
| sbi-miR6230-5p | SbNFY-B14 | 5 | -1 | 1 | 21 | 7066 | 7086 | UUUUGGGUCCCUAAACUUGUU | CUCAAGGGUACGGAUCCGAAG | Translation | | 1 |
| sbi-miR6231-3p | SbNFY-B14 | 5 | -1 | 1 | 21 | 876 | 896 | UAUUUGUGGACUCAUGGACAU | AUGUCCGUAAUUUCACGGAUA | Translation | | 1 |
| sbi-miR6231-3p | SbNFY-B11 | 5 | -1 | 1 | 21 | 3684 | 3704 | UAUUUGUGGACUCAUGGACAU | AAGUUGUUGUGUCUAUAAAUA | Cleavage |  | 1 |
| sbi-miR6231-5p | SbNFY-B9 | 5 | -1 | 1 | 21 | 4782 | 4802 | GUCCGUGAGUCCACAAAUAGG | CAAAUUCUUGGAUUUGUGGAC | Cleavage |  | 1 |
| sbi-miR6232a-3p | SbNFY-B16 | 5 | -1 | 1 | 24 | 430 | 453 | UGGAUGUACCAAAAAAGUCAAAGC | CUUUUGAAUUUUUGUGUGCAUCUA | Translation | | 1 |
| sbi-miR6232a-5p | SbNFY-B5 | 5 | -1 | 1 | 24 | 7000 | 7023 | GUCGCUUUGACUUUUUUGGUACAU | CUUGGUUACACAAGUUAAAGAGAC | Cleavage |  | 2 |
| sbi-miR6232a-5p | SbNFY-B8 | 5 | -1 | 1 | 24 | 10880 | 10903 | GUCGCUUUGACUUUUUUGGUACAU | ACUCUUCAAAAAAGUUAGAAUGGA | Cleavage |  | 3 |
| sbi-miR6232a-5p | SbNFY-B19 | 5 | -1 | 1 | 24 | 13616 | 13639 | GUCGCUUUGACUUUUUUGGUACAU | GCCACACGGCAAAGUCAGGGUGAU | Cleavage |  | 2 |
| sbi-miR6232a-5p | SbNFY-B10 | 5 | -1 | 1 | 24 | 8967 | 8990 | GUCGCUUUGACUUUUUUGGUACAU | GAUCUCCAAGGAGGCCAAGGAGAC | Translation | | 1 |
| sbi-miR6232a-5p | SbNFY-B7 | 5 | -1 | 1 | 24 | 5970 | 5993 | GUCGCUUUGACUUUUUUGGUACAU | GAUCUCCAAGGAGGCCAAGGAGAC | Translation | | 1 |
| sbi-miR6232b-3p | SbNFY-B10 | 5 | -1 | 1 | 21 | 12306 | 12326 | AAUUCGAUGUACCAAAAAAGU | UAUUUUAUGGUAAAUCUAAUG | Cleavage |  | 1 |
| sbi-miR6232b-3p | SbNFY-B6 | 5 | -1 | 1 | 21 | 6025 | 6045 | AAUUCGAUGUACCAAAAAAGU | GAUUUCUCGUCACAUCGAAUU | Translation | | 1 |
| sbi-miR6232b-5p | SbNFY-B10 | 5 | -1 | 1 | 21 | 15877 | 15897 | UUUUUGGUACAUUGAAUUUGC | UGAGAUUCGACGUGACAAGAA | Translation | | 4 |
| sbi-miR6232b-5p | SbNFY-B12 | 5 | -1 | 1 | 21 | 5013 | 5033 | UUUUUGGUACAUUGAAUUUGC | GCAAAAUAGAUAUAUUAAAAA | Translation | | 4 |
| sbi-miR6232b-5p | SbNFY-B8 | 5 | -1 | 1 | 21 | 11878 | 11898 | UUUUUGGUACAUUGAAUUUGC | ACAAAAAAAGUGUACUAAAAU | Cleavage |  | 2 |
| sbi-miR6232b-5p | SbNFY-B19 | 5 | -1 | 1 | 21 | 16691 | 16711 | UUUUUGGUACAUUGAAUUUGC | AAAGAUUUGAUGUGACAGAAU | Cleavage |  | 4 |
| sbi-miR6232b-5p | SbNFY-B19 | 5 | -1 | 1 | 21 | 16807 | 16827 | UUUUUGGUACAUUGAAUUUGC | AAAGAUUUGAUGUGACAGAAU | Cleavage |  | 4 |
| sbi-miR6232b-5p | SbNFY-B19 | 5 | -1 | 1 | 21 | 17532 | 17552 | UUUUUGGUACAUUGAAUUUGC | AAAGAUUCGAUGUGACGGGGA | Cleavage |  | 4 |
| sbi-miR6232b-5p | SbNFY-B1 | 5 | -1 | 1 | 21 | 15025 | 15045 | UUUUUGGUACAUUGAAUUUGC | AAAGAUUCGAUGUGACGGGGA | Cleavage |  | 1 |
| sbi-miR6232b-5p | SbNFY-B5 | 5 | -1 | 1 | 21 | 15255 | 15275 | UUUUUGGUACAUUGAAUUUGC | CAAAAUGCUGUGUAUCAGGGA | Cleavage |  | 2 |
| sbi-miR6232b-5p | SbNFY-B18 | 5 | -1 | 1 | 21 | 12043 | 12063 | UUUUUGGUACAUUGAAUUUGC | CAAGAUUCGAUGUGAUGGAGA | Cleavage |  | 1 |
| sbi-miR6232b-5p | SbNFY-B7 | 5 | -1 | 1 | 21 | 11558 | 11579 | UUUUUGGUACAUUGA-AUUUGC | CAAAAUAUUGAUGUGUUGAAAA | Cleavage |  | 1 |
| sbi-miR6233-3p | SbNFY-B4 | 5 | -1 | 1 | 24 | 5720 | 5743 | CAAGUUUGGUUUUGGUAAUUAAUG | GCCCUAUUGUCCACACCAAACUUU | Translation | | 1 |
| sbi-miR6233-3p | SbNFY-B17 | 5 | -1 | 1 | 24 | 17116 | 17139 | CAAGUUUGGUUUUGGUAAUUAAUG | AAUAAAUGAUCAAAAUAAAAGUUG | Cleavage |  | 3 |
| sbi-miR6233-3p | SbNFY-B17 | 5 | -1 | 1 | 24 | 17261 | 17284 | CAAGUUUGGUUUUGGUAAUUAAUG | GAGAAACUAUCAAAAUAAAAGUUG | Cleavage |  | 3 |
| sbi-miR6233-3p | SbNFY-B9 | 5 | -1 | 1 | 24 | 2400 | 2423 | CAAGUUUGGUUUUGGUAAUUAAUG | UGGACAUCAUCAAGGCUAGACUAG | Cleavage |  | 1 |
| sbi-miR6233-3p | SbNFY-B19 | 5 | -1 | 1 | 24 | 1631 | 1654 | CAAGUUUGGUUUUGGUAAUUAAUG | AGAUAGUUACAAGUGUCAAAUUUG | Translation | | 1 |
| sbi-miR6233-5p | SbNFY-B5 | 5 | -1 | 1 | 24 | 14970 | 14993 | UGUUGAGGCUGGAGCGAAACUCGG | GUAUGCCUUUUUCCAGCCUCGGCA | Cleavage |  | 2 |
| sbi-miR6233-5p | SbNFY-B12 | 5 | -1 | 1 | 24 | 16207 | 16230 | UGUUGAGGCUGGAGCGAAACUCGG | GUUCGUUUCCCUUCAGCUCCAGCU | Cleavage |  | 1 |
| sbi-miR6233-5p | SbNFY-B1 | 5 | -1 | 1 | 24 | 15582 | 15605 | UGUUGAGGCUGGAGCGAAACUCGG | UCUCUUUUUGAAUCAGCUUCGAUA | Cleavage |  | 1 |
| sbi-miR6233-5p | SbNFY-B2 | 5 | -1 | 1 | 24 | 9130 | 9154 | UGUUGAGGCUGGAG-CGAAACUCGG | GCAGCUUUUGACUCCAGUUUCCACA | Cleavage |  | 1 |
| sbi-miR6234a-5p | SbNFY-B7 | 5 | -1 | 1 | 24 | 4291 | 4314 | AAGUGUGUUCCUCUAUUUGACGCU | UGAAACACAGAGAGAAACAAACUU | Translation | | 2 |
| sbi-miR6234a-5p | SbNFY-B17 | 5 | -1 | 1 | 24 | 1581 | 1604 | AAGUGUGUUCCUCUAUUUGACGCU | AUUAUGCAAUCGAGGAACAUGCUC | Cleavage |  | 1 |
| sbi-miR6234b-5p | SbNFY-B7 | 5 | -1 | 1 | 24 | 4291 | 4314 | AAGUGUGUUCCUCUAUUUGACGCU | UGAAACACAGAGAGAAACAAACUU | Translation | | 2 |
| sbi-miR6234b-5p | SbNFY-B17 | 5 | -1 | 1 | 24 | 1581 | 1604 | AAGUGUGUUCCUCUAUUUGACGCU | AUUAUGCAAUCGAGGAACAUGCUC | Cleavage |  | 1 |
| sbi-miR6235-3p | SbNFY-B4 | 5 | -1 | 1 | 24 | 7379 | 7401 | AACGAACAGUAUUUUUCUCUUACA | AUGGAGAGAGUAGUACU-UUCGUU | Cleavage |  | 1 |
| sbi-miR6235-5p | SbNFY-B15 | 5 | -1 | 1 | 24 | 3825 | 3848 | UUGUGAGAGAAAAAUACUGUUGGC | AGUUUCCGUGUUUUUUUCCUGCAG | Cleavage |  | 1 |
| sbi-miR6235-5p | SbNFY-B14 | 5 | -1 | 1 | 24 | 6089 | 6112 | UUGUGAGAGAAAAAUACUGUUGGC | UUAAAUUUUAUUUUUCUUUAACAG | Cleavage |  | 3 |
| sbi-miR6235-5p | SbNFY-B14 | 5 | -1 | 1 | 24 | 1182 | 1205 | UUGUGAGAGAAAAAUACUGUUGGC | AAUUUUUUUAUUUUUCUUUCCCAA | Cleavage |  | 3 |
| sbi-miR6235-5p | SbNFY-B14 | 5 | -1 | 1 | 24 | 6426 | 6449 | UUGUGAGAGAAAAAUACUGUUGGC | GAGGACAUUUUUUCUUUUUUACAA | Translation | | 3 |
| sbi-miR821b | SbNFY-B1 | 5 | -1 | 1 | 21 | 2813 | 2833 | AAGUUAUGAACAUAAAAGUUG | GUCAUUUUAACUUCAUAACUU | Translation | | 1 |
| sbi-miR821b | SbNFY-B18 | 5 | -1 | 1 | 21 | 1689 | 1709 | AAGUUAUGAACAUAAAAGUUG | CUGCUCUUGUCUUCGUGAUUU | Translation | | 1 |
| sbi-miR821d | SbNFY-B11 | 5 | -1 | 1 | 21 | 9174 | 9194 | AAGUCAUCAACAACAAAGUUG | UAGUUUUGAUUUUGAUGGUUU | Translation | | 1 |
| sbi-miR821d | SbNFY-B6 | 5 | -1 | 1 | 21 | 8802 | 8822 | AAGUCAUCAACAACAAAGUUG | CAAUUGUUUGGUUGAUGACUC | Cleavage |  | 1 |
| sbi-miR821e | SbNFY-B11 | 5 | -1 | 1 | 21 | 16645 | 16664 | AAGUCAUCAAAAUAAAAGUUG | GAUUUUUUAUUUU-AUGAUUU | Cleavage |  | 2 |
| sbi-miR821e | SbNFY-B14 | 5 | -1 | 1 | 21 | 9213 | 9233 | AAGUCAUCAAAAUAAAAGUUG | AAAUAUUUAUUGUGAUCAUUU | Translation | | 2 |
| sbi-miR821e | SbNFY-B14 | 5 | -1 | 1 | 21 | 5588 | 5607 | AAGUCAUCAAAAUAAAAGUUG | AAAUUUUCAUUUU-AUGAUUU | Cleavage |  | 2 |
| sbi-miR821e | SbNFY-B4 | 5 | -1 | 1 | 21 | 8539 | 8559 | AAGUCAUCAAAAUAAAAGUUG | UCCAUUUGGUUUUGAUGAGUU | Cleavage |  | 1 |
