# Supplementary material for: Genome-wide identification and expression profile analysis of nuclear factor Y family genes in Sorghum bicolor L. (Moench)
Source: PLoS One. 2019 Sep 19;14(9):e0222203. doi: 10.1371/journal.pone.0222203 (PMC6752760; doi:10.1371/journal.pone.0222203)
Supplement: S13 Table — (DOC) [file pone.0222203.s021.doc]

**S13 Table.** In silico analysis of miRNAs for SbNFY-C

| miRNA_Acc. | | Target_Acc. | | Expectation | | UPE$ | | miRNA_start | | miRNA_end | | Target_start | | Target_end | | miRNA_aligned_fragment | | Target_aligned_fragment | | Inhibition | | Target_Desc. | | Multiplicity | |
| --- | --- | --- | --- | --- | --- | --- | --- | --- | --- | --- | --- | --- | --- | --- | --- | --- | --- | --- | --- | --- | --- | --- | --- | --- | --- |
| sbi-miR6232b-3p | SbNFY-C11 | | 0 | | -1 | | 1 | | 21 | | 11695 | | 11715 | | AAUUCGAUGUACCAAAAAAGU | | ACUUUUUUGGUACAUCGAAUU | | Cleavage | |  | | 1 | |  |
| sbi-miR6225-5p | SbNFY-C8 | | 0.5 | | -1 | | 1 | | 24 | | 16080 | | 16103 | | AACUAGACUCAAAAGAUUCAUCUC | | GAGACGAAUCUUUUGAGUCUGGUU | | Cleavage | |  | | 2 | |  |
| sbi-miR6235-5p | SbNFY-C12 | | 0.5 | | -1 | | 1 | | 24 | | 13241 | | 13264 | | UUGUGAGAGAAAAAUACUGUUGGC | | UUUAGCAGUGUUUUUCUCUCACAA | | Cleavage | |  | | 1 | |  |
| sbi-miR395f | SbNFY-C2 | | 1 | | -1 | | 1 | | 21 | | 3593 | | 3613 | | AUGAAGUGUUUGGGGGAACUC | | GAGUUCCUCCAAGCACUUCAU | | Cleavage | |  | | 2 | |  |
| sbi-miR395k | SbNFY-C2 | | 1 | | -1 | | 1 | | 21 | | 3593 | | 3613 | | GUGAAGUGUUUGGAGGAACUC | | GAGUUCCUCCAAGCACUUCAU | | Cleavage | |  | | 1 | |  |
| sbi-miR395l | SbNFY-C2 | | 1 | | -1 | | 1 | | 21 | | 3593 | | 3613 | | GUGAAGUGCUUGGGGGAACUC | | GAGUUCCUCCAAGCACUUCAU | | Cleavage | |  | | 2 | |  |
| sbi-miR437a | SbNFY-C7 | | 1 | | -1 | | 1 | | 21 | | 14099 | | 14119 | | AAAGUUAGAGAAGUUUGACUU | | AAAUCAAACUUCUCUAACUUU | | Cleavage | |  | | 2 | |  |
| sbi-miR437b | SbNFY-C7 | | 1 | | -1 | | 1 | | 21 | | 14099 | | 14119 | | AAAGUUAGAGAAGUUUGACUU | | AAAUCAAACUUCUCUAACUUU | | Cleavage | |  | | 2 | |  |
| sbi-miR437c | SbNFY-C7 | | 1 | | -1 | | 1 | | 21 | | 14099 | | 14119 | | AAAGUUAGAGAAGUUUGACUU | | AAAUCAAACUUCUCUAACUUU | | Cleavage | |  | | 2 | |  |
| sbi-miR437d | SbNFY-C7 | | 1 | | -1 | | 1 | | 21 | | 14099 | | 14119 | | AAAGUUAGAGAAGUUUGACUU | | AAAUCAAACUUCUCUAACUUU | | Cleavage | |  | | 2 | |  |
| sbi-miR437e | SbNFY-C7 | | 1 | | -1 | | 1 | | 21 | | 14099 | | 14119 | | AAAGUUAGAGAAGUUUGACUU | | AAAUCAAACUUCUCUAACUUU | | Cleavage | |  | | 2 | |  |
| sbi-miR437f | SbNFY-C7 | | 1 | | -1 | | 1 | | 21 | | 14099 | | 14119 | | AAAGUUAGAGAAGUUUGACUU | | AAAUCAAACUUCUCUAACUUU | | Cleavage | |  | | 2 | |  |
| sbi-miR437g | SbNFY-C7 | | 1 | | -1 | | 1 | | 21 | | 14099 | | 14119 | | AAAGUUAGAGAAGUUUGACUU | | AAAUCAAACUUCUCUAACUUU | | Cleavage | |  | | 2 | |  |
| sbi-miR437i | SbNFY-C7 | | 1 | | -1 | | 1 | | 21 | | 14099 | | 14119 | | AAAGUUAGAGAAGUUUGACUU | | AAAUCAAACUUCUCUAACUUU | | Cleavage | |  | | 2 | |  |
| sbi-miR437j | SbNFY-C7 | | 1 | | -1 | | 1 | | 21 | | 14099 | | 14119 | | AAAGUUAGAGAAGUUUGACUU | | AAAUCAAACUUCUCUAACUUU | | Cleavage | |  | | 2 | |  |
| sbi-miR437k | SbNFY-C7 | | 1 | | -1 | | 1 | | 21 | | 14099 | | 14119 | | AAAGUUAGAGAAGUUUGACUU | | AAAUCAAACUUCUCUAACUUU | | Cleavage | |  | | 2 | |  |
| sbi-miR437l | SbNFY-C7 | | 1 | | -1 | | 1 | | 21 | | 14099 | | 14119 | | AAAGUUAGAGAAGUUUGACUU | | AAAUCAAACUUCUCUAACUUU | | Cleavage | |  | | 2 | |  |
| sbi-miR437m | SbNFY-C7 | | 1 | | -1 | | 1 | | 21 | | 14099 | | 14119 | | AAAGUUAGAGAAGUUUGACUU | | AAAUCAAACUUCUCUAACUUU | | Cleavage | |  | | 2 | |  |
| sbi-miR437n | SbNFY-C7 | | 1 | | -1 | | 1 | | 21 | | 14099 | | 14119 | | AAAGUUAGAGAAGUUUGACUU | | AAAUCAAACUUCUCUAACUUU | | Cleavage | |  | | 2 | |  |
| sbi-miR437o | SbNFY-C7 | | 1 | | -1 | | 1 | | 21 | | 14099 | | 14119 | | AAAGUUAGAGAAGUUUGACUU | | AAAUCAAACUUCUCUAACUUU | | Cleavage | |  | | 2 | |  |
| sbi-miR437p | SbNFY-C7 | | 1 | | -1 | | 1 | | 21 | | 14099 | | 14119 | | AAAGUUAGAGAAGUUUGACUU | | AAAUCAAACUUCUCUAACUUU | | Cleavage | |  | | 2 | |  |
| sbi-miR437q | SbNFY-C7 | | 1 | | -1 | | 1 | | 21 | | 14099 | | 14119 | | AAAGUUAGAGAAGUUUGACUU | | AAAUCAAACUUCUCUAACUUU | | Cleavage | |  | | 2 | |  |
| sbi-miR437r | SbNFY-C7 | | 1 | | -1 | | 1 | | 21 | | 14099 | | 14119 | | AAAGUUAGAGAAGUUUGACUU | | AAAUCAAACUUCUCUAACUUU | | Cleavage | |  | | 2 | |  |
| sbi-miR437s | SbNFY-C7 | | 1 | | -1 | | 1 | | 21 | | 14099 | | 14119 | | AAAGUUAGAGAAGUUUGACUU | | AAAUCAAACUUCUCUAACUUU | | Cleavage | |  | | 2 | |  |
| sbi-miR437t | SbNFY-C7 | | 1 | | -1 | | 1 | | 21 | | 14099 | | 14119 | | AAAGUUAGAGAAGUUUGACUU | | AAAUCAAACUUCUCUAACUUU | | Cleavage | |  | | 2 | |  |
| sbi-miR437u | SbNFY-C7 | | 1 | | -1 | | 1 | | 21 | | 14099 | | 14119 | | AAAGUUAGAGAAGUUUGACUU | | AAAUCAAACUUCUCUAACUUU | | Cleavage | |  | | 2 | |  |
| sbi-miR437v | SbNFY-C7 | | 1 | | -1 | | 1 | | 21 | | 14099 | | 14119 | | AAAGUUAGAGAAGUUUGACUU | | AAAUCAAACUUCUCUAACUUU | | Cleavage | |  | | 2 | |  |
| sbi-miR437w | SbNFY-C7 | | 1 | | -1 | | 1 | | 21 | | 14099 | | 14119 | | AAAGUUAGAGAAGUUUGACUU | | AAAUCAAACUUCUCUAACUUU | | Cleavage | |  | | 2 | |  |
| sbi-miR5568c-5p | SbNFY-C1 | | 1 | | -1 | | 1 | | 21 | | 1933 | | 1953 | | UCUGUUCCAAAUUGUAAGUCG | | UGACUUAUAAUUUGGAACGGA | | Cleavage | |  | | 2 | |  |
| sbi-miR6227-3p | SbNFY-C1 | | 1 | | -1 | | 1 | | 22 | | 8502 | | 8523 | | CUCACAACACUUGCUAUUUGGG | | CCCAAAUAACAAGUGUUGUGAG | | Cleavage | |  | | 1 | |  |
| sbi-miR6230-3p | SbNFY-C9 | | 1 | | -1 | | 1 | | 21 | | 3327 | | 3347 | | UAACAAGUUUAGGGAUCUAGA | | CAUAUAUCCCUAAACUUGUUA | | Cleavage | |  | | 2 | |  |
| sbi-miR395a | SbNFY-C2 | | 1.5 | | -1 | | 1 | | 21 | | 3593 | | 3613 | | GUGAAGUGUUUGGGGGAACUC | | GAGUUCCUCCAAGCACUUCAU | | Cleavage | |  | | 2 | |  |
| sbi-miR395b | SbNFY-C2 | | 1.5 | | -1 | | 1 | | 21 | | 3593 | | 3613 | | GUGAAGUGUUUGGGGGAACUC | | GAGUUCCUCCAAGCACUUCAU | | Cleavage | |  | | 2 | |  |
| sbi-miR395c | SbNFY-C2 | | 1.5 | | -1 | | 1 | | 21 | | 3593 | | 3613 | | GUGAAGUGUUUGGGGGAACUC | | GAGUUCCUCCAAGCACUUCAU | | Cleavage | |  | | 2 | |  |
| sbi-miR395d | SbNFY-C2 | | 1.5 | | -1 | | 1 | | 21 | | 3593 | | 3613 | | GUGAAGUGUUUGGGGGAACUC | | GAGUUCCUCCAAGCACUUCAU | | Cleavage | |  | | 2 | |  |
| sbi-miR395e | SbNFY-C2 | | 1.5 | | -1 | | 1 | | 21 | | 3593 | | 3613 | | GUGAAGUGUUUGGGGGAACUC | | GAGUUCCUCCAAGCACUUCAU | | Cleavage | |  | | 2 | |  |
| sbi-miR395g | SbNFY-C2 | | 1.5 | | -1 | | 1 | | 21 | | 3593 | | 3613 | | GUGAAGUGUUUGGGGGAACUC | | GAGUUCCUCCAAGCACUUCAU | | Cleavage | |  | | 2 | |  |
| sbi-miR395h | SbNFY-C2 | | 1.5 | | -1 | | 1 | | 21 | | 3593 | | 3613 | | GUGAAGUGUUUGGGGGAACUC | | GAGUUCCUCCAAGCACUUCAU | | Cleavage | |  | | 2 | |  |
| sbi-miR395i | SbNFY-C2 | | 1.5 | | -1 | | 1 | | 21 | | 3593 | | 3613 | | GUGAAGUGUUUGGGGGAACUC | | GAGUUCCUCCAAGCACUUCAU | | Cleavage | |  | | 2 | |  |
| sbi-miR395j | SbNFY-C2 | | 1.5 | | -1 | | 1 | | 21 | | 3593 | | 3613 | | GUGAAGUGUUUGGGGGAACUC | | GAGUUCCUCCAAGCACUUCAU | | Cleavage | |  | | 2 | |  |
| sbi-miR437x-3p | SbNFY-C4 | | 1.5 | | -1 | | 1 | | 24 | | 5371 | | 5394 | | AUUUGACUGACACGGAUUCUAGGA | | UUUUAGAAUCCGUGUCAGUCAAUU | | Cleavage | |  | | 1 | |  |
| sbi-miR5568g-3p | SbNFY-C5 | | 1.5 | | -1 | | 1 | | 21 | | 12760 | | 12780 | | AAAACGUCUUAUAAUUUGGAG | | CUCCAAAAUAUAAGAUGUUUU | | Cleavage | |  | | 1 | |  |
| sbi-miR6220-3p | SbNFY-C1 | | 1.5 | | -1 | | 1 | | 24 | | 1710 | | 1733 | | AUGCCUUAUAAUUUGGGAUGGAGA | | CCUCCAUCCCAAAUUAUAAGUCAU | | Cleavage | |  | | 3 | |  |
| sbi-miR6225-5p | SbNFY-C5 | | 1.5 | | -1 | | 1 | | 24 | | 8102 | | 8125 | | AACUAGACUCAAAAGAUUCAUCUC | | GAAACGAAUCUUUUGAGCCUAGUU | | Cleavage | |  | | 2 | |  |
| sbi-miR6225-5p | SbNFY-C7 | | 1.5 | | -1 | | 1 | | 24 | | 2349 | | 2372 | | AACUAGACUCAAAAGAUUCAUCUC | | GAGACGAAUCUUUUGAGCCUAGUU | | Cleavage | |  | | 4 | |  |
| sbi-miR6227-5p | SbNFY-C1 | | 1.5 | | -1 | | 1 | | 24 | | 8316 | | 8339 | | GGGCCCAAAUAGCAAGUGUUGUGA | | UUACAACACUUGCUAUUUUGGCCC | | Cleavage | |  | | 1 | |  |
| sbi-miR6232a-3p | SbNFY-C11 | | 1.5 | | -1 | | 1 | | 24 | | 11689 | | 11712 | | UGGAUGUACCAAAAAAGUCAAAGC | | GCUUUGACUUUUUUGGUACAUCGA | | Cleavage | |  | | 1 | |  |
| sbi-miR6235-5p | SbNFY-C2 | | 1.5 | | -1 | | 1 | | 24 | | 2569 | | 2592 | | UUGUGAGAGAAAAAUACUGUUGGC | | UCCAGCAGUGUUUUUCUCUUAUAA | | Cleavage | |  | | 1 | |  |
| sbi-miR5567 | SbNFY-C11 | | 2 | | -1 | | 1 | | 24 | | 17695 | | 17719 | | UUAAUGAUUCAUGUAUGUGUC-CAA | | UUGCGGCACAUGCAUGAAGCAUUAA | | Cleavage | |  | | 2 | |  |
| sbi-miR5568a | SbNFY-C11 | | 2 | | -1 | | 1 | | 21 | | 11674 | | 11694 | | CAGAGCGACUUACAAUUUGGA | | UUCAAAUUAUAAGUCGCUUUG | | Cleavage | |  | | 2 | |  |
| sbi-miR5568g-3p | SbNFY-C11 | | 2 | | -1 | | 1 | | 21 | | 2535 | | 2555 | | AAAACGUCUUAUAAUUUGGAG | | UUCCAAAUUAUAAGUUGUUUU | | Cleavage | |  | | 2 | |  |
| sbi-miR5568g-5p | SbNFY-C11 | | 2 | | -1 | | 1 | | 21 | | 2638 | | 2658 | | CAAAUUAUAAGAUGUUUUGGC | | GUCAAAGCAACUUAUAAUUUG | | Cleavage | |  | | 2 | |  |
| sbi-miR5569 | SbNFY-C12 | | 2 | | -1 | | 1 | | 24 | | 17210 | | 17233 | | UAUUGCAUGCUUGAACUAUGGUAA | | UUACCAUAGUUCGAGCAUACAAUA | | Cleavage | |  | | 2 | |  |
| sbi-miR5569 | SbNFY-C6 | | 2 | | -1 | | 1 | | 24 | | 14913 | | 14936 | | UAUUGCAUGCUUGAACUAUGGUAA | | UUACCACAAUUCAAGCAUGCAAUA | | Cleavage | |  | | 1 | |  |
| sbi-miR6225-5p | SbNFY-C7 | | 2 | | -1 | | 1 | | 24 | | 7053 | | 7076 | | AACUAGACUCAAAAGAUUCAUCUC | | GAGACGAAUCUUUUGAGCCUGGUU | | Cleavage | |  | | 4 | |  |
| sbi-miR6231-5p | SbNFY-C10 | | 2 | | -1 | | 1 | | 21 | | 7149 | | 7169 | | GUCCGUGAGUCCACAAAUAGG | | CAUGUUUGUGGACUUAUGGAU | | Cleavage | |  | | 1 | |  |
| sbi-miR437a | SbNFY-C5 | | 2.5 | | -1 | | 1 | | 21 | | 12792 | | 12812 | | AAAGUUAGAGAAGUUUGACUU | | AAAUCGAACUUCUCUAGUUUU | | Cleavage | |  | | 1 | |  |
| sbi-miR437b | SbNFY-C5 | | 2.5 | | -1 | | 1 | | 21 | | 12792 | | 12812 | | AAAGUUAGAGAAGUUUGACUU | | AAAUCGAACUUCUCUAGUUUU | | Cleavage | |  | | 1 | |  |
| sbi-miR437c | SbNFY-C5 | | 2.5 | | -1 | | 1 | | 21 | | 12792 | | 12812 | | AAAGUUAGAGAAGUUUGACUU | | AAAUCGAACUUCUCUAGUUUU | | Cleavage | |  | | 1 | |  |
| sbi-miR437d | SbNFY-C5 | | 2.5 | | -1 | | 1 | | 21 | | 12792 | | 12812 | | AAAGUUAGAGAAGUUUGACUU | | AAAUCGAACUUCUCUAGUUUU | | Cleavage | |  | | 1 | |  |
| sbi-miR437e | SbNFY-C5 | | 2.5 | | -1 | | 1 | | 21 | | 12792 | | 12812 | | AAAGUUAGAGAAGUUUGACUU | | AAAUCGAACUUCUCUAGUUUU | | Cleavage | |  | | 1 | |  |
| sbi-miR437f | SbNFY-C5 | | 2.5 | | -1 | | 1 | | 21 | | 12792 | | 12812 | | AAAGUUAGAGAAGUUUGACUU | | AAAUCGAACUUCUCUAGUUUU | | Cleavage | |  | | 1 | |  |
| sbi-miR437g | SbNFY-C5 | | 2.5 | | -1 | | 1 | | 21 | | 12792 | | 12812 | | AAAGUUAGAGAAGUUUGACUU | | AAAUCGAACUUCUCUAGUUUU | | Cleavage | |  | | 1 | |  |
| sbi-miR437i | SbNFY-C5 | | 2.5 | | -1 | | 1 | | 21 | | 12792 | | 12812 | | AAAGUUAGAGAAGUUUGACUU | | AAAUCGAACUUCUCUAGUUUU | | Cleavage | |  | | 1 | |  |
| sbi-miR437j | SbNFY-C5 | | 2.5 | | -1 | | 1 | | 21 | | 12792 | | 12812 | | AAAGUUAGAGAAGUUUGACUU | | AAAUCGAACUUCUCUAGUUUU | | Cleavage | |  | | 1 | |  |
| sbi-miR437k | SbNFY-C5 | | 2.5 | | -1 | | 1 | | 21 | | 12792 | | 12812 | | AAAGUUAGAGAAGUUUGACUU | | AAAUCGAACUUCUCUAGUUUU | | Cleavage | |  | | 1 | |  |
| sbi-miR437l | SbNFY-C5 | | 2.5 | | -1 | | 1 | | 21 | | 12792 | | 12812 | | AAAGUUAGAGAAGUUUGACUU | | AAAUCGAACUUCUCUAGUUUU | | Cleavage | |  | | 1 | |  |
| sbi-miR437m | SbNFY-C5 | | 2.5 | | -1 | | 1 | | 21 | | 12792 | | 12812 | | AAAGUUAGAGAAGUUUGACUU | | AAAUCGAACUUCUCUAGUUUU | | Cleavage | |  | | 1 | |  |
| sbi-miR437n | SbNFY-C5 | | 2.5 | | -1 | | 1 | | 21 | | 12792 | | 12812 | | AAAGUUAGAGAAGUUUGACUU | | AAAUCGAACUUCUCUAGUUUU | | Cleavage | |  | | 1 | |  |
| sbi-miR437o | SbNFY-C5 | | 2.5 | | -1 | | 1 | | 21 | | 12792 | | 12812 | | AAAGUUAGAGAAGUUUGACUU | | AAAUCGAACUUCUCUAGUUUU | | Cleavage | |  | | 1 | |  |
| sbi-miR437p | SbNFY-C5 | | 2.5 | | -1 | | 1 | | 21 | | 12792 | | 12812 | | AAAGUUAGAGAAGUUUGACUU | | AAAUCGAACUUCUCUAGUUUU | | Cleavage | |  | | 1 | |  |
| sbi-miR437q | SbNFY-C5 | | 2.5 | | -1 | | 1 | | 21 | | 12792 | | 12812 | | AAAGUUAGAGAAGUUUGACUU | | AAAUCGAACUUCUCUAGUUUU | | Cleavage | |  | | 1 | |  |
| sbi-miR437r | SbNFY-C5 | | 2.5 | | -1 | | 1 | | 21 | | 12792 | | 12812 | | AAAGUUAGAGAAGUUUGACUU | | AAAUCGAACUUCUCUAGUUUU | | Cleavage | |  | | 1 | |  |
| sbi-miR437s | SbNFY-C5 | | 2.5 | | -1 | | 1 | | 21 | | 12792 | | 12812 | | AAAGUUAGAGAAGUUUGACUU | | AAAUCGAACUUCUCUAGUUUU | | Cleavage | |  | | 1 | |  |
| sbi-miR437t | SbNFY-C5 | | 2.5 | | -1 | | 1 | | 21 | | 12792 | | 12812 | | AAAGUUAGAGAAGUUUGACUU | | AAAUCGAACUUCUCUAGUUUU | | Cleavage | |  | | 1 | |  |
| sbi-miR437u | SbNFY-C5 | | 2.5 | | -1 | | 1 | | 21 | | 12792 | | 12812 | | AAAGUUAGAGAAGUUUGACUU | | AAAUCGAACUUCUCUAGUUUU | | Cleavage | |  | | 1 | |  |
| sbi-miR437v | SbNFY-C5 | | 2.5 | | -1 | | 1 | | 21 | | 12792 | | 12812 | | AAAGUUAGAGAAGUUUGACUU | | AAAUCGAACUUCUCUAGUUUU | | Cleavage | |  | | 1 | |  |
| sbi-miR437w | SbNFY-C5 | | 2.5 | | -1 | | 1 | | 21 | | 12792 | | 12812 | | AAAGUUAGAGAAGUUUGACUU | | AAAUCGAACUUCUCUAGUUUU | | Cleavage | |  | | 1 | |  |
| sbi-miR5567 | SbNFY-C7 | | 2.5 | | -1 | | 1 | | 24 | | 6983 | | 7007 | | UUAAUGAUUCAUGUAUGUGUC-CAA | | UUGCGACACAUGCAUGGAGCAUUAA | | Cleavage | |  | | 4 | |  |
| sbi-miR5568c-3p | SbNFY-C9 | | 2.5 | | -1 | | 1 | | 21 | | 10234 | | 10254 | | ACUUACAGUUUGGAACGGAGG | | CCUCCGUUCUAAAUUAUAAGU | | Cleavage | |  | | 1 | |  |
| sbi-miR5568c-3p | SbNFY-C3 | | 2.5 | | -1 | | 1 | | 21 | | 11200 | | 11220 | | ACUUACAGUUUGGAACGGAGG | | CCUCCAUUCCAAACUAUAAGU | | Cleavage | |  | | 1 | |  |
| sbi-miR5568c-5p | SbNFY-C1 | | 2.5 | | -1 | | 1 | | 21 | | 14497 | | 14517 | | UCUGUUCCAAAUUGUAAGUCG | | UGGCUUAUAAUUUAGAACAGA | | Cleavage | |  | | 2 | |  |
| sbi-miR5568c-5p | SbNFY-C11 | | 2.5 | | -1 | | 1 | | 21 | | 11757 | | 11777 | | UCUGUUCCAAAUUGUAAGUCG | | CAACUUAUAAUUUGGAAAGGA | | Cleavage | |  | | 2 | |  |
| sbi-miR5568f-3p | SbNFY-C9 | | 2.5 | | -1 | | 1 | | 21 | | 10235 | | 10255 | | GUCUUAUAAUUUGGAAUGGAG | | CUCCGUUCUAAAUUAUAAGUC | | Cleavage | |  | | 2 | |  |
| sbi-miR5568f-3p | SbNFY-C1 | | 2.5 | | -1 | | 1 | | 21 | | 1711 | | 1731 | | GUCUUAUAAUUUGGAAUGGAG | | CUCCAUCCCAAAUUAUAAGUC | | Cleavage | |  | | 3 | |  |
| sbi-miR5568f-3p | SbNFY-C1 | | 2.5 | | -1 | | 1 | | 21 | | 4091 | | 4111 | | GUCUUAUAAUUUGGAAUGGAG | | CUCCAGUCCAAUUUAUAAGAC | | Translation | | | | 3 | |  |
| sbi-miR5568g-3p | SbNFY-C1 | | 2.5 | | -1 | | 1 | | 21 | | 14477 | | 14497 | | AAAACGUCUUAUAAUUUGGAG | | UUCCAAAUUGUUAGAUGUUUU | | Translation | | | | 2 | |  |
| sbi-miR5568g-5p | SbNFY-C11 | | 2.5 | | -1 | | 1 | | 21 | | 11750 | | 11770 | | CAAAUUAUAAGAUGUUUUGGC | | GCCAAACCAACUUAUAAUUUG | | Cleavage | |  | | 2 | |  |
| sbi-miR6220-3p | SbNFY-C12 | | 2.5 | | -1 | | 1 | | 24 | | 4222 | | 4245 | | AUGCCUUAUAAUUUGGGAUGGAGA | | UCUCCAUCCAAAAUUAUAAGUCAU | | Cleavage | |  | | 1 | |  |
| sbi-miR6220-5p | SbNFY-C9 | | 2.5 | | -1 | | 1 | | 24 | | 10461 | | 10484 | | CUCCAUCCUAAAUUAUAAGACAUU | | AAUAACUUAUAGUUUGGGAGGGAG | | Cleavage | |  | | 1 | |  |
| sbi-miR6225-3p | SbNFY-C5 | | 2.5 | | -1 | | 1 | | 24 | | 1234 | | 1257 | | GAAACGAAUCUUUUAAGUCUAAUU | | AACUAGGUUUAAAAGAUUCGUCUC | | Cleavage | |  | | 4 | |  |
| sbi-miR6225-3p | SbNFY-C5 | | 2.5 | | -1 | | 1 | | 24 | | 5781 | | 5804 | | GAAACGAAUCUUUUAAGUCUAAUU | | AACUAGGCUCAAAAGAUUUGUUUU | | Cleavage | |  | | 4 | |  |
| sbi-miR6225-3p | SbNFY-C12 | | 2.5 | | -1 | | 1 | | 24 | | 8403 | | 8426 | | GAAACGAAUCUUUUAAGUCUAAUU | | AACUAGACUCAAAAGAUUCGUCUC | | Cleavage | |  | | 5 | |  |
| sbi-miR6225-3p | SbNFY-C2 | | 2.5 | | -1 | | 1 | | 24 | | 11239 | | 11262 | | GAAACGAAUCUUUUAAGUCUAAUU | | AACUAGACUCAAAAGAUUCGUCUC | | Cleavage | |  | | 2 | |  |
| sbi-miR6225-5p | SbNFY-C11 | | 2.5 | | -1 | | 1 | | 24 | | 17767 | | 17790 | | AACUAGACUCAAAAGAUUCAUCUC | | GAGAUGAAUUUUUUGAGCUUAGUU | | Cleavage | |  | | 2 | |  |
| sbi-miR6225-5p | SbNFY-C12 | | 2.5 | | -1 | | 1 | | 24 | | 11090 | | 11113 | | AACUAGACUCAAAAGAUUCAUCUC | | GAUACGAAUCUUUUGAGUCUAAUA | | Cleavage | |  | | 2 | |  |
| sbi-miR6226-3p | SbNFY-C6 | | 2.5 | | -1 | | 1 | | 24 | | 6562 | | 6585 | | GAUUAGUCACGAUUAGUCGUCCGA | | CAGGACGAUUAAUCGCGAUUAAUC | | Cleavage | |  | | 2 | |  |
| sbi-miR6226-3p | SbNFY-C6 | | 2.5 | | -1 | | 1 | | 24 | | 6118 | | 6141 | | GAUUAGUCACGAUUAGUCGUCCGA | | CCAGGCGAUUAAUCGCGAUUAAUC | | Cleavage | |  | | 2 | |  |
| sbi-miR6230-3p | SbNFY-C6 | | 2.5 | | -1 | | 1 | | 21 | | 3584 | | 3604 | | UAACAAGUUUAGGGAUCUAGA | | UCUCGGUCCCUAAACUUGUUU | | Cleavage | |  | | 4 | |  |
| sbi-miR5381 | SbNFY-C1 | | 3 | | -1 | | 1 | | 19 | | 12279 | | 12297 | | AAGAUCUGUGGCGCCGAGC | | GAUCGGAGCCACGGAUCUU | | Cleavage | |  | | 1 | |  |
| sbi-miR5389 | SbNFY-C1 | | 3 | | -1 | | 1 | | 21 | | 3690 | | 3710 | | GCUUGAGUUUAUCAGCCGAGU | | AUUCGACUGAUAAGCUCAAAC | | Cleavage | |  | | 1 | |  |
| sbi-miR5564b | SbNFY-C1 | | 3 | | -1 | | 1 | | 21 | | 10104 | | 10124 | | GCAAUUCGUCGAACAGCUUGA | | ACUAGAUGUUUGAUGAAUUGC | | Cleavage | |  | | 1 | |  |
| sbi-miR5567 | SbNFY-C8 | | 3 | | -1 | | 1 | | 24 | | 15763 | | 15786 | | UUAAUGAUUCAUGUAUGUGUCCAA | | UACGCCACAUGCAUGGAGUAUUAA | | Cleavage | |  | | 3 | |  |
| sbi-miR5568a | SbNFY-C11 | | 3 | | -1 | | 1 | | 21 | | 2536 | | 2556 | | CAGAGCGACUUACAAUUUGGA | | UCCAAAUUAUAAGUUGUUUUG | | Cleavage | |  | | 2 | |  |
| sbi-miR5568c-3p | SbNFY-C11 | | 3 | | -1 | | 1 | | 21 | | 11667 | | 11687 | | ACUUACAGUUUGGAACGGAGG | | ACUCUGUUUCAAAUUAUAAGU | | Cleavage | |  | | 2 | |  |
| sbi-miR5568e-5p | SbNFY-C7 | | 3 | | -1 | | 1 | | 21 | | 15241 | | 15261 | | GAUGUUUUGGGUUUUCUAGAU | | CGCUAGAAAGCCUAGAAUAUA | | Cleavage | |  | | 3 | |  |
| sbi-miR5568f-3p | SbNFY-C1 | | 3 | | -1 | | 1 | | 21 | | 14472 | | 14492 | | GUCUUAUAAUUUGGAAUGGAG | | UUCUAUUCCAAAUUGUUAGAU | | Cleavage | |  | | 3 | |  |
| sbi-miR5568f-3p | SbNFY-C11 | | 3 | | -1 | | 1 | | 21 | | 11668 | | 11688 | | GUCUUAUAAUUUGGAAUGGAG | | CUCUGUUUCAAAUUAUAAGUC | | Cleavage | |  | | 3 | |  |
| sbi-miR5568f-3p | SbNFY-C3 | | 3 | | -1 | | 1 | | 21 | | 11201 | | 11221 | | GUCUUAUAAUUUGGAAUGGAG | | CUCCAUUCCAAACUAUAAGUC | | Cleavage | |  | | 1 | |  |
| sbi-miR5568f-3p | SbNFY-C8 | | 3 | | -1 | | 1 | | 21 | | 5374 | | 5394 | | GUCUUAUAAUUUGGAAUGGAG | | UUAGAUUCCAAAUUGUAAGAU | | Cleavage | |  | | 1 | |  |
| sbi-miR5568f-5p | SbNFY-C11 | | 3 | | -1 | | 1 | | 21 | | 11757 | | 11777 | | UCCAUUCCAAAUUGUAAGAUG | | CAACUUAUAAUUUGGAAAGGA | | Cleavage | |  | | 1 | |  |
| sbi-miR5568f-5p | SbNFY-C1 | | 3 | | -1 | | 1 | | 21 | | 1933 | | 1953 | | UCCAUUCCAAAUUGUAAGAUG | | UGACUUAUAAUUUGGAACGGA | | Cleavage | |  | | 1 | |  |
| sbi-miR6220-3p | SbNFY-C9 | | 3 | | -1 | | 1 | | 24 | | 10234 | | 10257 | | AUGCCUUAUAAUUUGGGAUGGAGA | | CCUCCGUUCUAAAUUAUAAGUCAU | | Cleavage | |  | | 1 | |  |
| sbi-miR6225-3p | SbNFY-C1 | | 3 | | -1 | | 1 | | 24 | | 15565 | | 15588 | | GAAACGAAUCUUUUAAGUCUAAUU | | UACUAGGUUUAAAAGAUUUGUCUC | | Cleavage | |  | | 2 | |  |
| sbi-miR6225-3p | SbNFY-C8 | | 3 | | -1 | | 1 | | 24 | | 16293 | | 16316 | | GAAACGAAUCUUUUAAGUCUAAUU | | AAUUAGGCUCAAAAGAUUCGUGUC | | Cleavage | |  | | 1 | |  |
| sbi-miR6225-3p | SbNFY-C12 | | 3 | | -1 | | 1 | | 24 | | 13734 | | 13757 | | GAAACGAAUCUUUUAAGUCUAAUU | | AACUAGGCUCAAAAGAUUCGUCUC | | Cleavage | |  | | 5 | |  |
| sbi-miR6225-5p | SbNFY-C5 | | 3 | | -1 | | 1 | | 24 | | 1469 | | 1492 | | AACUAGACUCAAAAGAUUCAUCUC | | GAGAUGAAUCUUUUGAGCAUAGUU | | Cleavage | |  | | 2 | |  |
| sbi-miR6225-5p | SbNFY-C7 | | 3 | | -1 | | 1 | | 24 | | 9604 | | 9627 | | AACUAGACUCAAAAGAUUCAUCUC | | GAGAUAAAUUUUUUGAGCCUAGUU | | Cleavage | |  | | 4 | |  |
| sbi-miR6225-5p | SbNFY-C7 | | 3 | | -1 | | 1 | | 24 | | 16434 | | 16457 | | AACUAGACUCAAAAGAUUCAUCUC | | GAGACGAAUCUUUUAAGCCUAGUU | | Translation | | | | 4 | |  |
| sbi-miR6225-5p | SbNFY-C14 | | 3 | | -1 | | 1 | | 24 | | 15544 | | 15567 | | AACUAGACUCAAAAGAUUCAUCUC | | GAGACGAAUCUUUUAAGCCUAGUU | | Translation | | | | 3 | |  |
| sbi-miR6225-5p | SbNFY-C14 | | 3 | | -1 | | 1 | | 24 | | 15878 | | 15901 | | AACUAGACUCAAAAGAUUCAUCUC | | GAGACGAAUCUUUUAAGUCUAAUU | | Translation | | | | 3 | |  |
| sbi-miR6225-5p | SbNFY-C9 | | 3 | | -1 | | 1 | | 24 | | 10721 | | 10744 | | AACUAGACUCAAAAGAUUCAUCUC | | GAGACGAAUCUUUUAAGCCUAGUU | | Translation | | | | 1 | |  |
| sbi-miR6225-5p | SbNFY-C4 | | 3 | | -1 | | 1 | | 24 | | 11395 | | 11418 | | AACUAGACUCAAAAGAUUCAUCUC | | GAUACGAAUAUUUUAAGUUUAGUU | | Translation | | | | 1 | |  |
| sbi-miR6232a-3p | SbNFY-C3 | | 3 | | -1 | | 1 | | 24 | | 11221 | | 11244 | | UGGAUGUACCAAAAAAGUCAAAGC | | CACUUGGUUUUUUAGGUAUAUCCA | | Translation | | | | 1 | |  |
| sbi-miR6232b-3p | SbNFY-C3 | | 3 | | -1 | | 1 | | 21 | | 11227 | | 11247 | | AAUUCGAUGUACCAAAAAAGU | | GUUUUUUAGGUAUAUCCAAUU | | Cleavage | |  | | 1 | |  |
| sbi-miR6232b-5p | SbNFY-C11 | | 3 | | -1 | | 1 | | 21 | | 11728 | | 11748 | | UUUUUGGUACAUUGAAUUUGC | | AGACAUUGGAUGUACUAAAAA | | Cleavage | |  | | 4 | |  |
| sbi-miR395a | SbNFY-C4 | | 3.5 | | -1 | | 1 | | 21 | | 4117 | | 4137 | | GUGAAGUGUUUGGGGGAACUC | | AAUUUCUCCAAAACAUUUCAC | | Cleavage | |  | | 1 | |  |
| sbi-miR395b | SbNFY-C4 | | 3.5 | | -1 | | 1 | | 21 | | 4117 | | 4137 | | GUGAAGUGUUUGGGGGAACUC | | AAUUUCUCCAAAACAUUUCAC | | Cleavage | |  | | 1 | |  |
| sbi-miR395c | SbNFY-C4 | | 3.5 | | -1 | | 1 | | 21 | | 4117 | | 4137 | | GUGAAGUGUUUGGGGGAACUC | | AAUUUCUCCAAAACAUUUCAC | | Cleavage | |  | | 1 | |  |
| sbi-miR395d | SbNFY-C4 | | 3.5 | | -1 | | 1 | | 21 | | 4117 | | 4137 | | GUGAAGUGUUUGGGGGAACUC | | AAUUUCUCCAAAACAUUUCAC | | Cleavage | |  | | 1 | |  |
| sbi-miR395e | SbNFY-C4 | | 3.5 | | -1 | | 1 | | 21 | | 4117 | | 4137 | | GUGAAGUGUUUGGGGGAACUC | | AAUUUCUCCAAAACAUUUCAC | | Cleavage | |  | | 1 | |  |
| sbi-miR395g | SbNFY-C4 | | 3.5 | | -1 | | 1 | | 21 | | 4117 | | 4137 | | GUGAAGUGUUUGGGGGAACUC | | AAUUUCUCCAAAACAUUUCAC | | Cleavage | |  | | 1 | |  |
| sbi-miR395h | SbNFY-C4 | | 3.5 | | -1 | | 1 | | 21 | | 4117 | | 4137 | | GUGAAGUGUUUGGGGGAACUC | | AAUUUCUCCAAAACAUUUCAC | | Cleavage | |  | | 1 | |  |
| sbi-miR395i | SbNFY-C4 | | 3.5 | | -1 | | 1 | | 21 | | 4117 | | 4137 | | GUGAAGUGUUUGGGGGAACUC | | AAUUUCUCCAAAACAUUUCAC | | Cleavage | |  | | 1 | |  |
| sbi-miR395j | SbNFY-C4 | | 3.5 | | -1 | | 1 | | 21 | | 4117 | | 4137 | | GUGAAGUGUUUGGGGGAACUC | | AAUUUCUCCAAAACAUUUCAC | | Cleavage | |  | | 1 | |  |
| sbi-miR5386 | SbNFY-C7 | | 3.5 | | -1 | | 1 | | 20 | | 3873 | | 3892 | | CGUCGCUGUCGCGCGCGCUG | | UGGUGCGCGCGGAAGCGGCG | | Cleavage | |  | | 1 | |  |
| sbi-miR5386 | SbNFY-C9 | | 3.5 | | -1 | | 1 | | 20 | | 14320 | | 14339 | | CGUCGCUGUCGCGCGCGCUG | | UGGAGCGCGCGGUGGCGGCG | | Cleavage | |  | | 2 | |  |
| sbi-miR5389 | SbNFY-C12 | | 3.5 | | -1 | | 1 | | 21 | | 8112 | | 8132 | | GCUUGAGUUUAUCAGCCGAGU | | AUUCGACUGAUAAGUUCAAAC | | Cleavage | |  | | 1 | |  |
| sbi-miR5564b | SbNFY-C7 | | 3.5 | | -1 | | 1 | | 21 | | 7790 | | 7810 | | GCAAUUCGUCGAACAGCUUGA | | UCGAGCUGUUCGCCGAAAUGC | | Cleavage | |  | | 1 | |  |
| sbi-miR5567 | SbNFY-C7 | | 3.5 | | -1 | | 1 | | 24 | | 16363 | | 16386 | | UUAAUGAUUCAUGUAUGUGUCCAA | | UUGGACACGUGCAUGGAAUAUUAA | | Cleavage | |  | | 4 | |  |
| sbi-miR5567 | SbNFY-C5 | | 3.5 | | -1 | | 1 | | 24 | | 204 | | 227 | | UUAAUGAUUCAUGUAUGUGUCCAA | | AAUGACAUAUACAUAAAACAUUAA | | Translation | | | | 3 | |  |
| sbi-miR5568c-5p | SbNFY-C9 | | 3.5 | | -1 | | 1 | | 21 | | 10463 | | 10483 | | UCUGUUCCAAAUUGUAAGUCG | | UAACUUAUAGUUUGGGAGGGA | | Cleavage | |  | | 1 | |  |
| sbi-miR5568d-3p | SbNFY-C3 | | 3.5 | | -1 | | 1 | | 21 | | 3780 | | 3800 | | AAAGUUGUGUAUCUAGAAAAG | | CUUUUUGAGAUCUACAACUUU | | Translation | | | | 2 | |  |
| sbi-miR5568d-3p | SbNFY-C3 | | 3.5 | | -1 | | 1 | | 21 | | 3933 | | 3953 | | AAAGUUGUGUAUCUAGAAAAG | | CUUUUUGAGAUCUACAACUUU | | Translation | | | | 2 | |  |
| sbi-miR5568d-3p | SbNFY-C14 | | 3.5 | | -1 | | 1 | | 21 | | 5185 | | 5205 | | AAAGUUGUGUAUCUAGAAAAG | | CUUUUAUAGGUACAUAUCUUU | | Cleavage | |  | | 1 | |  |
| sbi-miR5568g-3p | SbNFY-C11 | | 3.5 | | -1 | | 1 | | 21 | | 11673 | | 11693 | | AAAACGUCUUAUAAUUUGGAG | | UUUCAAAUUAUAAGUCGCUUU | | Cleavage | |  | | 2 | |  |
| sbi-miR5568g-5p | SbNFY-C14 | | 3.5 | | -1 | | 1 | | 21 | | 5269 | | 5289 | | CAAAUUAUAAGAUGUUUUGGC | | AUUAAAACGACUUAUAAUUUA | | Cleavage | |  | | 2 | |  |
| sbi-miR5568g-5p | SbNFY-C9 | | 3.5 | | -1 | | 1 | | 21 | | 10456 | | 10476 | | CAAAUUAUAAGAUGUUUUGGC | | CUUGAAAUAACUUAUAGUUUG | | Cleavage | |  | | 1 | |  |
| sbi-miR5568g-5p | SbNFY-C4 | | 3.5 | | -1 | | 1 | | 21 | | 8021 | | 8041 | | CAAAUUAUAAGAUGUUUUGGC | | CUCAAAUUAUUUUAUAGUUUU | | Cleavage | |  | | 1 | |  |
| sbi-miR5569 | SbNFY-C8 | | 3.5 | | -1 | | 1 | | 24 | | 2750 | | 2773 | | UAUUGCAUGCUUGAACUAUGGUAA | | UUAUUAUAGUUGAGGCAUACAAUA | | Cleavage | |  | | 1 | |  |
| sbi-miR6219-5p | SbNFY-C1 | | 3.5 | | -1 | | 1 | | 24 | | 15516 | | 15539 | | GAACCGGGACUAAAGGUGGGACAU | | CCAAGCCACGUUUAGGCCUGGUUU | | Cleavage | |  | | 1 | |  |
| sbi-miR6220-5p | SbNFY-C1 | | 3.5 | | -1 | | 1 | | 24 | | 1931 | | 1954 | | CUCCAUCCUAAAUUAUAAGACAUU | | AAUGACUUAUAAUUUGGAACGGAG | | Cleavage | |  | | 1 | |  |
| sbi-miR6220-5p | SbNFY-C11 | | 3.5 | | -1 | | 1 | | 24 | | 11755 | | 11778 | | CUCCAUCCUAAAUUAUAAGACAUU | | ACCAACUUAUAAUUUGGAAAGGAG | | Cleavage | |  | | 1 | |  |
| sbi-miR6225-3p | SbNFY-C1 | | 3.5 | | -1 | | 1 | | 24 | | 7957 | | 7980 | | GAAACGAAUCUUUUAAGUCUAAUU | | AAUUAGACUUAAAAGAUUUAUAUC | | Cleavage | |  | | 2 | |  |
| sbi-miR6225-3p | SbNFY-C14 | | 3.5 | | -1 | | 1 | | 24 | | 15098 | | 15121 | | GAAACGAAUCUUUUAAGUCUAAUU | | AAUUAGGCUUAAAAGUUUCGUCUC | | Cleavage | |  | | 1 | |  |
| sbi-miR6225-3p | SbNFY-C12 | | 3.5 | | -1 | | 1 | | 24 | | 15159 | | 15182 | | GAAACGAAUCUUUUAAGUCUAAUU | | AACUAGGCUCAAAAGAUUUGUCUC | | Cleavage | |  | | 5 | |  |
| sbi-miR6225-3p | SbNFY-C12 | | 3.5 | | -1 | | 1 | | 24 | | 9611 | | 9634 | | GAAACGAAUCUUUUAAGUCUAAUU | | CAGUAGAUUCAAAAGAUUCGUCUU | | Cleavage | |  | | 5 | |  |
| sbi-miR6225-3p | SbNFY-C11 | | 3.5 | | -1 | | 1 | | 24 | | 6621 | | 6644 | | GAAACGAAUCUUUUAAGUCUAAUU | | AACUAGUCUCAAAAGAUUCGUCUC | | Cleavage | |  | | 1 | |  |
| sbi-miR6230-5p | SbNFY-C2 | | 3.5 | | -1 | | 1 | | 21 | | 16269 | | 16289 | | UUUUGGGUCCCUAAACUUGUU | | CUCAAGAUUAGUGACUUAAAA | | Translation | | | | 1 | |  |
| sbi-miR6232b-5p | SbNFY-C15 | | 3.5 | | -1 | | 1 | | 21 | | 8661 | | 8682 | | UUUUUGGUACAUUGAAU-UUGC | | GUAAUAUUCAAUGUAACAAAAA | | Cleavage | |  | | 2 | |  |
| sbi-miR6232b-5p | SbNFY-C12 | | 3.5 | | -1 | | 1 | | 21 | | 15850 | | 15870 | | UUUUUGGUACAUUGAAUUUGC | | AGAGAUUCAAUGUGAUAAAGA | | Cleavage | |  | | 5 | |  |
| sbi-miR6232b-5p | SbNFY-C1 | | 3.5 | | -1 | | 1 | | 21 | | 15656 | | 15676 | | UUUUUGGUACAUUGAAUUUGC | | AAAGAUUCGAUGUGACAGAAA | | Cleavage | |  | | 3 | |  |
| sbi-miR6233-3p | SbNFY-C6 | | 3.5 | | -1 | | 1 | | 24 | | 767 | | 791 | | CAAGUUUGGUUUUGG-UAAUUAAUG | | CAUCAAUUAUCCAAAACCAAACUAG | | Cleavage | |  | | 2 | |  |
| sbi-miR6235-5p | SbNFY-C4 | | 3.5 | | -1 | | 1 | | 24 | | 5179 | | 5202 | | UUGUGAGAGAAAAAUACUGUUGGC | | AUAUAUAAUUUUUUUCUUUUACAA | | Cleavage | |  | | 3 | |  |
| sbi-miR6235-5p | SbNFY-C1 | | 3.5 | | -1 | | 1 | | 24 | | 4641 | | 4664 | | UUGUGAGAGAAAAAUACUGUUGGC | | AUGAACAAUGUUUUUCUCUAAUAA | | Cleavage | |  | | 1 | |  |
| sbi-miR159a | SbNFY-C5 | | 4 | | -1 | | 1 | | 21 | | 12440 | | 12460 | | UUUGGAUUGAAGGGAGCUCUG | | ACAAGCUUUCUUCACUUCAAA | | Cleavage | |  | | 1 | |  |
| sbi-miR159a | SbNFY-C3 | | 4 | | -1 | | 1 | | 21 | | 7157 | | 7177 | | UUUGGAUUGAAGGGAGCUCUG | | CCCAGCAACUUUUAAUCCAAA | | Cleavage | |  | | 4 | |  |
| sbi-miR162 | SbNFY-C6 | | 4 | | -1 | | 1 | | 21 | | 5262 | | 5282 | | UCGAUAAACCUCUGCAUCCAG | | GCGGAUGGAGAGAUUUGUUGG | | Cleavage | |  | | 1 | |  |
| sbi-miR2118-3p | SbNFY-C4 | | 4 | | -1 | | 1 | | 22 | | 14020 | | 14041 | | UUCCUGAUGCCUCCCAUGCCUA | | AAUGCAUUGGGGGCAUUAUGAG | | Cleavage | |  | | 1 | |  |
| sbi-miR396a | SbNFY-C7 | | 4 | | -1 | | 1 | | 21 | | 2494 | | 2514 | | UUCCACAGCUUUCUUGAACUG | | AAGUACAGGAGGGCUUUGGAA | | Cleavage | |  | | 1 | |  |
| sbi-miR396b | SbNFY-C7 | | 4 | | -1 | | 1 | | 21 | | 2494 | | 2514 | | UUCCACAGCUUUCUUGAACUG | | AAGUACAGGAGGGCUUUGGAA | | Cleavage | |  | | 1 | |  |
| sbi-miR396c | SbNFY-C7 | | 4 | | -1 | | 1 | | 21 | | 2494 | | 2514 | | UUCCACAGCUUUCUUGAACUU | | AAGUACAGGAGGGCUUUGGAA | | Cleavage | |  | | 1 | |  |
| sbi-miR408 | SbNFY-C8 | | 4 | | -1 | | 1 | | 21 | | 3442 | | 3462 | | CUGCACUGCCUCUUCCCUGGC | | CGCAGAGAAGAGGCAGUCCGC | | Cleavage | |  | | 1 | |  |
| sbi-miR437x-5p | SbNFY-C4 | | 4 | | -1 | | 1 | | 24 | | 8109 | | 8132 | | UAGAGUUGUCCUAAGUCAAACUUU | | AAAGCUUAACUUAAAACAACUCUA | | Translation | | | | 1 | |  |
| sbi-miR5386 | SbNFY-C9 | | 4 | | -1 | | 1 | | 20 | | 14558 | | 14577 | | CGUCGCUGUCGCGCGCGCUG | | GGGCACCCGCGGCGGCGGCG | | Cleavage | |  | | 2 | |  |
| sbi-miR5565e | SbNFY-C7 | | 4 | | -1 | | 1 | | 19 | | 13876 | | 13894 | | UUGUUUGGAUGUUGUCGGA | | UACGACAAUGUUCAGGUAA | | Cleavage | |  | | 1 | |  |
| sbi-miR5565f | SbNFY-C13 | | 4 | | -1 | | 1 | | 20 | | 3388 | | 3407 | | UAGUCGGAUUUAUAUCAAUC | | CAUUUAUAUAUAUCCAACUA | | Translation | | | | 2 | |  |
| sbi-miR5567 | SbNFY-C7 | | 4 | | -1 | | 1 | | 24 | | 2278 | | 2301 | | UUAAUGAUUCAUGUAUGUGUCCAA | | UUAGACGUAUGCAUGGAGUAUUAA | | Cleavage | |  | | 4 | |  |
| sbi-miR5567 | SbNFY-C4 | | 4 | | -1 | | 1 | | 24 | | 11333 | | 11356 | | UUAAUGAUUCAUGUAUGUGUCCAA | | UCGGACACAUGCAUUGAACAUUAA | | Translation | | | | 1 | |  |
| sbi-miR5567 | SbNFY-C5 | | 4 | | -1 | | 1 | | 24 | | 8032 | | 8056 | | UUAAUGAUUCAUGUAUGUGUC-CAA | | UUGCGACACAUGCAUAGAGCAUUAA | | Translation | | | | 3 | |  |
| sbi-miR5567 | SbNFY-C3 | | 4 | | -1 | | 1 | | 24 | | 6110 | | 6133 | | UUAAUGAUUCAUGUAUGUGUCCAA | | CUGCAUGCAUAUAUGCGUCAUUGA | | Cleavage | |  | | 2 | |  |
| sbi-miR5567 | SbNFY-C8 | | 4 | | -1 | | 1 | | 24 | | 2153 | | 2176 | | UUAAUGAUUCAUGUAUGUGUCCAA | | CGCGGCACAUGCAUAGAGCAUUAA | | Translation | | | | 3 | |  |
| sbi-miR5567 | SbNFY-C15 | | 4 | | -1 | | 1 | | 24 | | 8776 | | 8799 | | UUAAUGAUUCAUGUAUGUGUCCAA | | UACGGCACAUACAUUGAAUAUUAA | | Translation | | | | 1 | |  |
| sbi-miR5568b-3p | SbNFY-C11 | | 4 | | -1 | | 1 | | 21 | | 2598 | | 2618 | | ACUAUGUAUCUAGAAAAGCUA | | UUAUUUAUCUAGAUGCAUAGC | | Cleavage | |  | | 1 | |  |
| sbi-miR5568b-5p | SbNFY-C1 | | 4 | | -1 | | 1 | | 21 | | 11857 | | 11877 | | UUUCUAGGUACAUAGCUUUUG | | GUGAAGCUUUGUAUUUGGAGA | | Cleavage | |  | | 1 | |  |
| sbi-miR5568b-5p | SbNFY-C13 | | 4 | | -1 | | 1 | | 21 | | 5177 | | 5197 | | UUUCUAGGUACAUAGCUUUUG | | GCAAACCCAUGUGUUUGGAAA | | Cleavage | |  | | 1 | |  |
| sbi-miR5568c-3p | SbNFY-C1 | | 4 | | -1 | | 1 | | 21 | | 1710 | | 1730 | | ACUUACAGUUUGGAACGGAGG | | CCUCCAUCCCAAAUUAUAAGU | | Cleavage | |  | | 2 | |  |
| sbi-miR5568c-5p | SbNFY-C3 | | 4 | | -1 | | 1 | | 21 | | 11271 | | 11291 | | UCUGUUCCAAAUUGUAAGUCG | | AGUCAUAUAAUUUGAAACAGA | | Cleavage | |  | | 1 | |  |
| sbi-miR5568c-5p | SbNFY-C10 | | 4 | | -1 | | 1 | | 21 | | 3869 | | 3889 | | UCUGUUCCAAAUUGUAAGUCG | | CAAAUUAUAGUUUGGAACGAA | | Cleavage | |  | | 1 | |  |
| sbi-miR5568d-5p | SbNFY-C8 | | 4 | | -1 | | 1 | | 21 | | 10068 | | 10088 | | UGGCUUUUCUAGAUACAUAGC | | GUGAUGUAUUUAUAGAAGCUA | | Cleavage | |  | | 2 | |  |
| sbi-miR5568d-5p | SbNFY-C12 | | 4 | | -1 | | 1 | | 21 | | 8762 | | 8782 | | UGGCUUUUCUAGAUACAUAGC | | CCUUUUGAUUUAGAAAGGCCA | | Cleavage | |  | | 2 | |  |
| sbi-miR5568e-3p | SbNFY-C14 | | 4 | | -1 | | 1 | | 21 | | 5177 | | 5196 | | UAUCUAGAAAAGCUAAAACGU | | UUGUUUU-GCUUUUAUAGGUA | | Cleavage | |  | | 2 | |  |
| sbi-miR5568e-5p | SbNFY-C12 | | 4 | | -1 | | 1 | | 21 | | 4253 | | 4273 | | GAUGUUUUGGGUUUUCUAGAU | | AUCUUGGAGAGUCAAAACAUC | | Translation | | | | 2 | |  |
| sbi-miR5568e-5p | SbNFY-C6 | | 4 | | -1 | | 1 | | 21 | | 11843 | | 11863 | | GAUGUUUUGGGUUUUCUAGAU | | AGGUUGAAAAUUCAAGGCAUC | | Cleavage | |  | | 1 | |  |
| sbi-miR5568f-3p | SbNFY-C10 | | 4 | | -1 | | 1 | | 21 | | 3667 | | 3687 | | GUCUUAUAAUUUGGAAUGGAG | | CUCCAUUCCAAAUUAUAAUUU | | Cleavage | |  | | 1 | |  |
| sbi-miR5568f-3p | SbNFY-C11 | | 4 | | -1 | | 1 | | 21 | | 2530 | | 2550 | | GUCUUAUAAUUUGGAAUGGAG | | ACUCUUUCCAAAUUAUAAGUU | | Cleavage | |  | | 3 | |  |
| sbi-miR5568f-3p | SbNFY-C12 | | 4 | | -1 | | 1 | | 21 | | 4223 | | 4243 | | GUCUUAUAAUUUGGAAUGGAG | | CUCCAUCCAAAAUUAUAAGUC | | Cleavage | |  | | 2 | |  |
| sbi-miR5568f-3p | SbNFY-C5 | | 4 | | -1 | | 1 | | 21 | | 12755 | | 12775 | | GUCUUAUAAUUUGGAAUGGAG | | AUCUGCUCCAAAAUAUAAGAU | | Cleavage | |  | | 1 | |  |
| sbi-miR5568f-3p | SbNFY-C2 | | 4 | | -1 | | 1 | | 21 | | 14855 | | 14875 | | GUCUUAUAAUUUGGAAUGGAG | | AUCAAUUUUGAAUUGUAAGGU | | Cleavage | |  | | 1 | |  |
| sbi-miR5568f-5p | SbNFY-C9 | | 4 | | -1 | | 1 | | 21 | | 10463 | | 10483 | | UCCAUUCCAAAUUGUAAGAUG | | UAACUUAUAGUUUGGGAGGGA | | Cleavage | |  | | 1 | |  |
| sbi-miR5568g-3p | SbNFY-C1 | | 4 | | -1 | | 1 | | 21 | | 3886 | | 3906 | | AAAACGUCUUAUAAUUUGGAG | | UCCUUGGAUAUAGGACGUUUU | | Cleavage | |  | | 2 | |  |
| sbi-miR5568g-5p | SbNFY-C1 | | 4 | | -1 | | 1 | | 21 | | 1926 | | 1946 | | CAAAUUAUAAGAUGUUUUGGC | | GAUGGAAUGACUUAUAAUUUG | | Cleavage | |  | | 2 | |  |
| sbi-miR5568g-5p | SbNFY-C3 | | 4 | | -1 | | 1 | | 21 | | 11264 | | 11284 | | CAAAUUAUAAGAUGUUUUGGC | | CCCCAAAAGUCAUAUAAUUUG | | Translation | | | | 1 | |  |
| sbi-miR5570 | SbNFY-C4 | | 4 | | -1 | | 1 | | 21 | | 426 | | 446 | | AAAAGACAAAUCAGCAUGUCA | | GCACAAACUGAGUUGUUUUUU | | Translation | | | | 1 | |  |
| sbi-miR6217a-3p | SbNFY-C11 | | 4 | | -1 | | 1 | | 24 | | 5143 | | 5166 | | AAAAUUAUCGUAAAUAGAGGUGGC | | CGGAUAGUUCUUUAUGAUAAUUUU | | Cleavage | |  | | 1 | |  |
| sbi-miR6217b-3p | SbNFY-C11 | | 4 | | -1 | | 1 | | 24 | | 5143 | | 5166 | | AAAAUUAUCGUAAAUAGAGGUGGC | | CGGAUAGUUCUUUAUGAUAAUUUU | | Cleavage | |  | | 1 | |  |
| sbi-miR6218-3p | SbNFY-C3 | | 4 | | -1 | | 1 | | 21 | | 6635 | | 6655 | | ACAAGUUUCGUGAUUUUUGGA | | GGCAAUAAUUGGGAAAUUUGU | | Translation | | | | 1 | |  |
| sbi-miR6220-3p | SbNFY-C11 | | 4 | | -1 | | 1 | | 24 | | 2529 | | 2552 | | AUGCCUUAUAAUUUGGGAUGGAGA | | UACUCUUUCCAAAUUAUAAGUUGU | | Cleavage | |  | | 2 | |  |
| sbi-miR6222-5p | SbNFY-C13 | | 4 | | -1 | | 1 | | 21 | | 15845 | | 15865 | | CCUGUUUGGAUCAGCCAAGGC | | AGCUUUGCUGAUCCAAAAGGA | | Cleavage | |  | | 1 | |  |
| sbi-miR6223-5p | SbNFY-C9 | | 4 | | -1 | | 1 | | 21 | | 10027 | | 10047 | | UUCUUGGGAGGAGCAUGCUAG | | UAAUCAUGUUCCUCCUUGGAA | | Cleavage | |  | | 1 | |  |
| sbi-miR6224a-3p | SbNFY-C14 | | 4 | | -1 | | 1 | | 21 | | 8862 | | 8882 | | CUUAUAUACUAGGACGGAGGG | | GUACCUGUUCUAGUGUGUAAG | | Cleavage | |  | | 1 | |  |
| sbi-miR6224b-3p | SbNFY-C14 | | 4 | | -1 | | 1 | | 21 | | 8862 | | 8882 | | CUUAUAUACUAGGACGGAGGG | | GUACCUGUUCUAGUGUGUAAG | | Cleavage | |  | | 1 | |  |
| sbi-miR6224c-3p | SbNFY-C14 | | 4 | | -1 | | 1 | | 21 | | 8862 | | 8882 | | CUUAUAUACUAGGACGGAGGG | | GUACCUGUUCUAGUGUGUAAG | | Cleavage | |  | | 1 | |  |
| sbi-miR6225-3p | SbNFY-C5 | | 4 | | -1 | | 1 | | 24 | | 574 | | 597 | | GAAACGAAUCUUUUAAGUCUAAUU | | AAUUAGGGUCAAAAGAUUCGUCUC | | Cleavage | |  | | 4 | |  |
| sbi-miR6225-3p | SbNFY-C15 | | 4 | | -1 | | 1 | | 24 | | 17680 | | 17703 | | GAAACGAAUCUUUUAAGUCUAAUU | | AACUAGGAUCAAAAGAUUCGUCUC | | Cleavage | |  | | 2 | |  |
| sbi-miR6225-5p | SbNFY-C8 | | 4 | | -1 | | 1 | | 24 | | 2220 | | 2243 | | AACUAGACUCAAAAGAUUCAUCUC | | GAGACAAAUCUUUUGAGCCUAUUU | | Cleavage | |  | | 2 | |  |
| sbi-miR6225-5p | SbNFY-C11 | | 4 | | -1 | | 1 | | 24 | | 16164 | | 16187 | | AACUAGACUCAAAAGAUUCAUCUC | | AAGACAAAUCUUUUAAGUCUAAUU | | Translation | | | | 2 | |  |
| sbi-miR6227-3p | SbNFY-C9 | | 4 | | -1 | | 1 | | 22 | | 8604 | | 8625 | | CUCACAACACUUGCUAUUUGGG | | GCACAAUAGAAACUGUUGUGAG | | Translation | | | | 1 | |  |
| sbi-miR6228-5p | SbNFY-C15 | | 4 | | -1 | | 1 | | 24 | | 4025 | | 4048 | | UUCUAUCUCUAUUAAUUGUGUUGC | | CAAAUACAAGUAAUACAAAUAGAA | | Cleavage | |  | | 1 | |  |
| sbi-miR6228-5p | SbNFY-C12 | | 4 | | -1 | | 1 | | 24 | | 11032 | | 11055 | | UUCUAUCUCUAUUAAUUGUGUUGC | | GCAGAGCAUUAAAUAUAGAUAGAA | | Cleavage | |  | | 1 | |  |
| sbi-miR6230-3p | SbNFY-C6 | | 4 | | -1 | | 1 | | 21 | | 3553 | | 3573 | | UAACAAGUUUAGGGAUCUAGA | | UUUAGGUCCUCGAACUUGUUC | | Translation | | | | 4 | |  |
| sbi-miR6230-3p | SbNFY-C6 | | 4 | | -1 | | 1 | | 21 | | 3644 | | 3664 | | UAACAAGUUUAGGGAUCUAGA | | UUUAGGUCCUCAAAUUUGUUC | | Translation | | | | 4 | |  |
| sbi-miR6230-3p | SbNFY-C9 | | 4 | | -1 | | 1 | | 21 | | 3292 | | 3312 | | UAACAAGUUUAGGGAUCUAGA | | UCAGGAUCUUUAAACUUGGUA | | Cleavage | |  | | 2 | |  |
| sbi-miR6232b-5p | SbNFY-C12 | | 4 | | -1 | | 1 | | 21 | | 12135 | | 12155 | | UUUUUGGUACAUUGAAUUUGC | | AAAGAUUCGAUGUGACAGAGA | | Cleavage | |  | | 5 | |  |
| sbi-miR6232b-5p | SbNFY-C12 | | 4 | | -1 | | 1 | | 21 | | 15252 | | 15272 | | UUUUUGGUACAUUGAAUUUGC | | AAAAAUUCGAUGUGACGAGGA | | Cleavage | |  | | 5 | |  |
| sbi-miR6233-3p | SbNFY-C1 | | 4 | | -1 | | 1 | | 24 | | 12920 | | 12943 | | CAAGUUUGGUUUUGGUAAUUAAUG | | UCCAAGUUUCUAAGGUCAAAUUUG | | Cleavage | |  | | 1 | |  |
| sbi-miR6234a-3p | SbNFY-C7 | | 4 | | -1 | | 1 | | 24 | | 4203 | | 4226 | | UUAGCGUCAAGAGACGAACACACU | | GUUCCGUUCGUCGUUUGGCGCUGC | | Cleavage | |  | | 1 | |  |
| sbi-miR6234a-5p | SbNFY-C14 | | 4 | | -1 | | 1 | | 24 | | 16902 | | 16925 | | AAGUGUGUUCCUCUAUUUGACGCU | | UAAGGCAAAUAGAGGAAUGCGCGC | | Cleavage | |  | | 1 | |  |
| sbi-miR6234b-3p | SbNFY-C7 | | 4 | | -1 | | 1 | | 24 | | 4203 | | 4226 | | UUAGCGUCAAGAGACGAACACACU | | GUUCCGUUCGUCGUUUGGCGCUGC | | Cleavage | |  | | 1 | |  |
| sbi-miR6234b-5p | SbNFY-C14 | | 4 | | -1 | | 1 | | 24 | | 16902 | | 16925 | | AAGUGUGUUCCUCUAUUUGACGCU | | UAAGGCAAAUAGAGGAAUGCGCGC | | Cleavage | |  | | 1 | |  |
| sbi-miR6235-5p | SbNFY-C4 | | 4 | | -1 | | 1 | | 24 | | 13583 | | 13606 | | UUGUGAGAGAAAAAUACUGUUGGC | | ACAAACAGUGUAUUGCUCUCGCAA | | Translation | | | | 3 | |  |
| sbi-miR6235-5p | SbNFY-C10 | | 4 | | -1 | | 1 | | 24 | | 12831 | | 12854 | | UUGUGAGAGAAAAAUACUGUUGGC | | AAGGCCUGUGUUUUUUUUUCAUAC | | Cleavage | |  | | 1 | |  |
| sbi-miR821a | SbNFY-C1 | | 4 | | -1 | | 1 | | 21 | | 11888 | | 11908 | | AAGUCAUCAACAUAAAAGUUG | | GUUCUAUUCUGUUGAUGAUUU | | Cleavage | |  | | 1 | |  |
| sbi-miR821b | SbNFY-C13 | | 4 | | -1 | | 1 | | 21 | | 16627 | | 16647 | | AAGUUAUGAACAUAAAAGUUG | | CGGCUUUCUUGUUCAUAGUUU | | Cleavage | |  | | 1 | |  |
| sbi-miR821c | SbNFY-C1 | | 4 | | -1 | | 1 | | 21 | | 11888 | | 11908 | | AAGUCAUCAACAUAAAAGUUG | | GUUCUAUUCUGUUGAUGAUUU | | Cleavage | |  | | 1 | |  |
| sbi-miR821e | SbNFY-C3 | | 4 | | -1 | | 1 | | 21 | | 3799 | | 3819 | | AAGUCAUCAAAAUAAAAGUUG | | UUAAUUUUAUUUUGGUCGUUU | | Cleavage | |  | | 1 | |  |
| sbi-miR1435a | SbNFY-C13 | | 4.5 | | -1 | | 1 | | 20 | | 7802 | | 7821 | | UUUCUUAAGUCAAACUUUUC | | UGGAAGUUUCAUGUAAGAAA | | Translation | | | | 1 | |  |
| sbi-miR1435b | SbNFY-C2 | | 4.5 | | -1 | | 1 | | 20 | | 1121 | | 1140 | | UUUCUUAAGUCAAACCUUUU | | UGAGGGUUUGGCUUUGGAAU | | Cleavage | |  | | 1 | |  |
| sbi-miR156d | SbNFY-C4 | | 4.5 | | -1 | | 1 | | 21 | | 437 | | 457 | | UGACAGAAGAGAGAGAGCACA | | GUUGUUUUUUUUUUUUUGUUC | | Cleavage | |  | | 1 | |  |
| sbi-miR156d | SbNFY-C13 | | 4.5 | | -1 | | 1 | | 21 | | 11785 | | 11805 | | UGACAGAAGAGAGAGAGCACA | | GCUGUUUUCUUUCUUGUGUGA | | Cleavage | |  | | 1 | |  |
| sbi-miR159a | SbNFY-C14 | | 4.5 | | -1 | | 1 | | 21 | | 6114 | | 6134 | | UUUGGAUUGAAGGGAGCUCUG | | AUUAGCUCCUUUGAAUCUAAU | | Cleavage | |  | | 1 | |  |
| sbi-miR159a | SbNFY-C6 | | 4.5 | | -1 | | 1 | | 21 | | 16465 | | 16485 | | UUUGGAUUGAAGGGAGCUCUG | | CCGGGCUUCCUCCAAUCUACA | | Translation | | | | 2 | |  |
| sbi-miR159b | SbNFY-C14 | | 4.5 | | -1 | | 1 | | 21 | | 6114 | | 6134 | | CUUGGAUUGAAGGGAGCUCCU | | AUUAGCUCCUUUGAAUCUAAU | | Cleavage | |  | | 1 | |  |
| sbi-miR164a | SbNFY-C1 | | 4.5 | | -1 | | 1 | | 21 | | 14596 | | 14616 | | UGGAGAAGCAGGGCACGUGCA | | CACUUGUGCUCUCCUUUUUCA | | Cleavage | |  | | 1 | |  |
| sbi-miR164a | SbNFY-C14 | | 4.5 | | -1 | | 1 | | 21 | | 15734 | | 15754 | | UGGAGAAGCAGGGCACGUGCA | | AUCACGUGCCUUCUUUCACUA | | Cleavage | |  | | 1 | |  |
| sbi-miR164b | SbNFY-C14 | | 4.5 | | -1 | | 1 | | 21 | | 15734 | | 15754 | | UGGAGAAGCAGGGCACGUGCU | | AUCACGUGCCUUCUUUCACUA | | Cleavage | |  | | 1 | |  |
| sbi-miR164b | SbNFY-C1 | | 4.5 | | -1 | | 1 | | 21 | | 14596 | | 14616 | | UGGAGAAGCAGGGCACGUGCU | | CACUUGUGCUCUCCUUUUUCA | | Cleavage | |  | | 1 | |  |
| sbi-miR164d | SbNFY-C1 | | 4.5 | | -1 | | 1 | | 21 | | 14596 | | 14616 | | UGGAGAAGCAGGGCACGUGCA | | CACUUGUGCUCUCCUUUUUCA | | Cleavage | |  | | 1 | |  |
| sbi-miR164d | SbNFY-C14 | | 4.5 | | -1 | | 1 | | 21 | | 15734 | | 15754 | | UGGAGAAGCAGGGCACGUGCA | | AUCACGUGCCUUCUUUCACUA | | Cleavage | |  | | 1 | |  |
| sbi-miR164e | SbNFY-C1 | | 4.5 | | -1 | | 1 | | 21 | | 14596 | | 14616 | | UGGAGAAGCAGGGCACGUGCA | | CACUUGUGCUCUCCUUUUUCA | | Cleavage | |  | | 1 | |  |
| sbi-miR164e | SbNFY-C14 | | 4.5 | | -1 | | 1 | | 21 | | 15734 | | 15754 | | UGGAGAAGCAGGGCACGUGCA | | AUCACGUGCCUUCUUUCACUA | | Cleavage | |  | | 1 | |  |
| sbi-miR168 | SbNFY-C9 | | 4.5 | | -1 | | 1 | | 21 | | 5676 | | 5696 | | UCGCUUGGUGCAGAUCGGGAC | | GCCCUGGUGUGCAUCAAGCCA | | Cleavage | |  | | 1 | |  |
| sbi-miR169i | SbNFY-C10 | | 4.5 | | -1 | | 1 | | 21 | | 275 | | 295 | | UAGCCAAGAAUGACUUGCCUA | | GGGACAAGUCAUUUGUGGCGA | | Cleavage | |  | | 1 | |  |
| sbi-miR169p | SbNFY-C3 | | 4.5 | | -1 | | 1 | | 21 | | 15369 | | 15389 | | UAGCCAAGAAUGGCUUGCCUA | | CUGGCAAGCUUUAUUUGGUUA | | Translation | | | | 1 | |  |
| sbi-miR169q | SbNFY-C3 | | 4.5 | | -1 | | 1 | | 21 | | 15369 | | 15389 | | UAGCCAAGAAUGGCUUGCCUA | | CUGGCAAGCUUUAUUUGGUUA | | Translation | | | | 1 | |  |
| sbi-miR172b | SbNFY-C4 | | 4.5 | | -1 | | 1 | | 20 | | 3134 | | 3153 | | GGAAUCUUGAUGAUGCUGCA | | UGAAGUAUUAUUAAGAUGUC | | Cleavage | |  | | 1 | |  |
| sbi-miR2118-5p | SbNFY-C4 | | 4.5 | | -1 | | 1 | | 22 | | 16431 | | 16452 | | GGCAUGGGAACAUGUAGGAAGG | | CAGUUUUACAUGUAUUUAUGCU | | Cleavage | |  | | 1 | |  |
| sbi-miR319a | SbNFY-C13 | | 4.5 | | -1 | | 1 | | 20 | | 11301 | | 11320 | | UUGGACUGAAGGGUGCUCCC | | AGGACUGUCCUUCACUCUAA | | Cleavage | |  | | 1 | |  |
| sbi-miR319b | SbNFY-C13 | | 4.5 | | -1 | | 1 | | 20 | | 11301 | | 11320 | | UUGGACUGAAGGGUGCUCCC | | AGGACUGUCCUUCACUCUAA | | Cleavage | |  | | 1 | |  |
| sbi-miR390 | SbNFY-C11 | | 4.5 | | -1 | | 1 | | 21 | | 5216 | | 5236 | | AAGCUCAGGAGGGAUAGCGCC | | CUUGCUAGCGCUACUGAGCUU | | Cleavage | |  | | 1 | |  |
| sbi-miR390 | SbNFY-C10 | | 4.5 | | -1 | | 1 | | 21 | | 16805 | | 16825 | | AAGCUCAGGAGGGAUAGCGCC | | ACCGUUACUUCUCUUGAGAUU | | Cleavage | |  | | 1 | |  |
| sbi-miR390 | SbNFY-C12 | | 4.5 | | -1 | | 1 | | 21 | | 5367 | | 5387 | | AAGCUCAGGAGGGAUAGCGCC | | ACCUUUAGUUCUUCUGGGCUU | | Cleavage | |  | | 1 | |  |
| sbi-miR393a | SbNFY-C14 | | 4.5 | | -1 | | 1 | | 21 | | 9202 | | 9221 | | UCCAAAGGGAUCGCAUUGAUC | | UGUCAG-GCGUUCUCUUUGGA | | Translation | | | | 1 | |  |
| sbi-miR393b | SbNFY-C14 | | 4.5 | | -1 | | 1 | | 21 | | 9202 | | 9221 | | UCCAAAGGGAUCGCAUUGAUC | | UGUCAG-GCGUUCUCUUUGGA | | Translation | | | | 1 | |  |
| sbi-miR395f | SbNFY-C2 | | 4.5 | | -1 | | 1 | | 21 | | 7926 | | 7946 | | AUGAAGUGUUUGGGGGAACUC | | AGGCUUCCCUGAGCUCUUCAU | | Cleavage | |  | | 2 | |  |
| sbi-miR395f | SbNFY-C4 | | 4.5 | | -1 | | 1 | | 21 | | 4117 | | 4137 | | AUGAAGUGUUUGGGGGAACUC | | AAUUUCUCCAAAACAUUUCAC | | Cleavage | |  | | 1 | |  |
| sbi-miR395l | SbNFY-C2 | | 4.5 | | -1 | | 1 | | 21 | | 7926 | | 7946 | | GUGAAGUGCUUGGGGGAACUC | | AGGCUUCCCUGAGCUCUUCAU | | Cleavage | |  | | 2 | |  |
| sbi-miR395l | SbNFY-C13 | | 4.5 | | -1 | | 1 | | 21 | | 6269 | | 6289 | | GUGAAGUGCUUGGGGGAACUC | | CAGUUUCCCCGUGCGCGUCAC | | Translation | | | | 1 | |  |
| sbi-miR396a | SbNFY-C13 | | 4.5 | | -1 | | 1 | | 21 | | 8029 | | 8049 | | UUCCACAGCUUUCUUGAACUG | | AAGUACCAGAAAGUUGUUGGA | | Cleavage | |  | | 2 | |  |
| sbi-miR396b | SbNFY-C13 | | 4.5 | | -1 | | 1 | | 21 | | 8029 | | 8049 | | UUCCACAGCUUUCUUGAACUG | | AAGUACCAGAAAGUUGUUGGA | | Cleavage | |  | | 2 | |  |
| sbi-miR396c | SbNFY-C13 | | 4.5 | | -1 | | 1 | | 21 | | 8029 | | 8049 | | UUCCACAGCUUUCUUGAACUU | | AAGUACCAGAAAGUUGUUGGA | | Cleavage | |  | | 2 | |  |
| sbi-miR408 | SbNFY-C9 | | 4.5 | | -1 | | 1 | | 21 | | 17108 | | 17128 | | CUGCACUGCCUCUUCCCUGGC | | GGAUGAGAAGAGGUAGUGCAC | | Cleavage | |  | | 2 | |  |
| sbi-miR437a | SbNFY-C7 | | 4.5 | | -1 | | 1 | | 21 | | 10653 | | 10673 | | AAAGUUAGAGAAGUUUGACUU | | AAUUCAUACUUUUUUAAGUUU | | Cleavage | |  | | 2 | |  |
| sbi-miR437a | SbNFY-C4 | | 4.5 | | -1 | | 1 | | 21 | | 13522 | | 13542 | | AAAGUUAGAGAAGUUUGACUU | | AUGUUAUAUGUCUCUAACUUA | | Cleavage | |  | | 2 | |  |
| sbi-miR437a | SbNFY-C4 | | 4.5 | | -1 | | 1 | | 21 | | 5396 | | 5416 | | AAAGUUAGAGAAGUUUGACUU | | UUUUAAAAUUUUACUAACUUU | | Cleavage | |  | | 2 | |  |
| sbi-miR437b | SbNFY-C7 | | 4.5 | | -1 | | 1 | | 21 | | 10653 | | 10673 | | AAAGUUAGAGAAGUUUGACUU | | AAUUCAUACUUUUUUAAGUUU | | Cleavage | |  | | 2 | |  |
| sbi-miR437b | SbNFY-C4 | | 4.5 | | -1 | | 1 | | 21 | | 13522 | | 13542 | | AAAGUUAGAGAAGUUUGACUU | | AUGUUAUAUGUCUCUAACUUA | | Cleavage | |  | | 2 | |  |
| sbi-miR437b | SbNFY-C4 | | 4.5 | | -1 | | 1 | | 21 | | 5396 | | 5416 | | AAAGUUAGAGAAGUUUGACUU | | UUUUAAAAUUUUACUAACUUU | | Cleavage | |  | | 2 | |  |
| sbi-miR437c | SbNFY-C7 | | 4.5 | | -1 | | 1 | | 21 | | 10653 | | 10673 | | AAAGUUAGAGAAGUUUGACUU | | AAUUCAUACUUUUUUAAGUUU | | Cleavage | |  | | 2 | |  |
| sbi-miR437c | SbNFY-C4 | | 4.5 | | -1 | | 1 | | 21 | | 13522 | | 13542 | | AAAGUUAGAGAAGUUUGACUU | | AUGUUAUAUGUCUCUAACUUA | | Cleavage | |  | | 2 | |  |
| sbi-miR437c | SbNFY-C4 | | 4.5 | | -1 | | 1 | | 21 | | 5396 | | 5416 | | AAAGUUAGAGAAGUUUGACUU | | UUUUAAAAUUUUACUAACUUU | | Cleavage | |  | | 2 | |  |
| sbi-miR437d | SbNFY-C7 | | 4.5 | | -1 | | 1 | | 21 | | 10653 | | 10673 | | AAAGUUAGAGAAGUUUGACUU | | AAUUCAUACUUUUUUAAGUUU | | Cleavage | |  | | 2 | |  |
| sbi-miR437d | SbNFY-C4 | | 4.5 | | -1 | | 1 | | 21 | | 13522 | | 13542 | | AAAGUUAGAGAAGUUUGACUU | | AUGUUAUAUGUCUCUAACUUA | | Cleavage | |  | | 2 | |  |
| sbi-miR437d | SbNFY-C4 | | 4.5 | | -1 | | 1 | | 21 | | 5396 | | 5416 | | AAAGUUAGAGAAGUUUGACUU | | UUUUAAAAUUUUACUAACUUU | | Cleavage | |  | | 2 | |  |
| sbi-miR437e | SbNFY-C7 | | 4.5 | | -1 | | 1 | | 21 | | 10653 | | 10673 | | AAAGUUAGAGAAGUUUGACUU | | AAUUCAUACUUUUUUAAGUUU | | Cleavage | |  | | 2 | |  |
| sbi-miR437e | SbNFY-C4 | | 4.5 | | -1 | | 1 | | 21 | | 13522 | | 13542 | | AAAGUUAGAGAAGUUUGACUU | | AUGUUAUAUGUCUCUAACUUA | | Cleavage | |  | | 2 | |  |
| sbi-miR437e | SbNFY-C4 | | 4.5 | | -1 | | 1 | | 21 | | 5396 | | 5416 | | AAAGUUAGAGAAGUUUGACUU | | UUUUAAAAUUUUACUAACUUU | | Cleavage | |  | | 2 | |  |
| sbi-miR437f | SbNFY-C7 | | 4.5 | | -1 | | 1 | | 21 | | 10653 | | 10673 | | AAAGUUAGAGAAGUUUGACUU | | AAUUCAUACUUUUUUAAGUUU | | Cleavage | |  | | 2 | |  |
| sbi-miR437f | SbNFY-C4 | | 4.5 | | -1 | | 1 | | 21 | | 13522 | | 13542 | | AAAGUUAGAGAAGUUUGACUU | | AUGUUAUAUGUCUCUAACUUA | | Cleavage | |  | | 2 | |  |
| sbi-miR437f | SbNFY-C4 | | 4.5 | | -1 | | 1 | | 21 | | 5396 | | 5416 | | AAAGUUAGAGAAGUUUGACUU | | UUUUAAAAUUUUACUAACUUU | | Cleavage | |  | | 2 | |  |
| sbi-miR437g | SbNFY-C7 | | 4.5 | | -1 | | 1 | | 21 | | 10653 | | 10673 | | AAAGUUAGAGAAGUUUGACUU | | AAUUCAUACUUUUUUAAGUUU | | Cleavage | |  | | 2 | |  |
| sbi-miR437g | SbNFY-C4 | | 4.5 | | -1 | | 1 | | 21 | | 13522 | | 13542 | | AAAGUUAGAGAAGUUUGACUU | | AUGUUAUAUGUCUCUAACUUA | | Cleavage | |  | | 2 | |  |
| sbi-miR437g | SbNFY-C4 | | 4.5 | | -1 | | 1 | | 21 | | 5396 | | 5416 | | AAAGUUAGAGAAGUUUGACUU | | UUUUAAAAUUUUACUAACUUU | | Cleavage | |  | | 2 | |  |
| sbi-miR437i | SbNFY-C7 | | 4.5 | | -1 | | 1 | | 21 | | 10653 | | 10673 | | AAAGUUAGAGAAGUUUGACUU | | AAUUCAUACUUUUUUAAGUUU | | Cleavage | |  | | 2 | |  |
| sbi-miR437i | SbNFY-C4 | | 4.5 | | -1 | | 1 | | 21 | | 13522 | | 13542 | | AAAGUUAGAGAAGUUUGACUU | | AUGUUAUAUGUCUCUAACUUA | | Cleavage | |  | | 2 | |  |
| sbi-miR437i | SbNFY-C4 | | 4.5 | | -1 | | 1 | | 21 | | 5396 | | 5416 | | AAAGUUAGAGAAGUUUGACUU | | UUUUAAAAUUUUACUAACUUU | | Cleavage | |  | | 2 | |  |
| sbi-miR437j | SbNFY-C7 | | 4.5 | | -1 | | 1 | | 21 | | 10653 | | 10673 | | AAAGUUAGAGAAGUUUGACUU | | AAUUCAUACUUUUUUAAGUUU | | Cleavage | |  | | 2 | |  |
| sbi-miR437j | SbNFY-C4 | | 4.5 | | -1 | | 1 | | 21 | | 13522 | | 13542 | | AAAGUUAGAGAAGUUUGACUU | | AUGUUAUAUGUCUCUAACUUA | | Cleavage | |  | | 2 | |  |
| sbi-miR437j | SbNFY-C4 | | 4.5 | | -1 | | 1 | | 21 | | 5396 | | 5416 | | AAAGUUAGAGAAGUUUGACUU | | UUUUAAAAUUUUACUAACUUU | | Cleavage | |  | | 2 | |  |
| sbi-miR437k | SbNFY-C7 | | 4.5 | | -1 | | 1 | | 21 | | 10653 | | 10673 | | AAAGUUAGAGAAGUUUGACUU | | AAUUCAUACUUUUUUAAGUUU | | Cleavage | |  | | 2 | |  |
| sbi-miR437k | SbNFY-C4 | | 4.5 | | -1 | | 1 | | 21 | | 13522 | | 13542 | | AAAGUUAGAGAAGUUUGACUU | | AUGUUAUAUGUCUCUAACUUA | | Cleavage | |  | | 2 | |  |
| sbi-miR437k | SbNFY-C4 | | 4.5 | | -1 | | 1 | | 21 | | 5396 | | 5416 | | AAAGUUAGAGAAGUUUGACUU | | UUUUAAAAUUUUACUAACUUU | | Cleavage | |  | | 2 | |  |
| sbi-miR437l | SbNFY-C7 | | 4.5 | | -1 | | 1 | | 21 | | 10653 | | 10673 | | AAAGUUAGAGAAGUUUGACUU | | AAUUCAUACUUUUUUAAGUUU | | Cleavage | |  | | 2 | |  |
| sbi-miR437l | SbNFY-C4 | | 4.5 | | -1 | | 1 | | 21 | | 13522 | | 13542 | | AAAGUUAGAGAAGUUUGACUU | | AUGUUAUAUGUCUCUAACUUA | | Cleavage | |  | | 2 | |  |
| sbi-miR437l | SbNFY-C4 | | 4.5 | | -1 | | 1 | | 21 | | 5396 | | 5416 | | AAAGUUAGAGAAGUUUGACUU | | UUUUAAAAUUUUACUAACUUU | | Cleavage | |  | | 2 | |  |
| sbi-miR437m | SbNFY-C7 | | 4.5 | | -1 | | 1 | | 21 | | 10653 | | 10673 | | AAAGUUAGAGAAGUUUGACUU | | AAUUCAUACUUUUUUAAGUUU | | Cleavage | |  | | 2 | |  |
| sbi-miR437m | SbNFY-C4 | | 4.5 | | -1 | | 1 | | 21 | | 13522 | | 13542 | | AAAGUUAGAGAAGUUUGACUU | | AUGUUAUAUGUCUCUAACUUA | | Cleavage | |  | | 2 | |  |
| sbi-miR437m | SbNFY-C4 | | 4.5 | | -1 | | 1 | | 21 | | 5396 | | 5416 | | AAAGUUAGAGAAGUUUGACUU | | UUUUAAAAUUUUACUAACUUU | | Cleavage | |  | | 2 | |  |
| sbi-miR437n | SbNFY-C7 | | 4.5 | | -1 | | 1 | | 21 | | 10653 | | 10673 | | AAAGUUAGAGAAGUUUGACUU | | AAUUCAUACUUUUUUAAGUUU | | Cleavage | |  | | 2 | |  |
| sbi-miR437n | SbNFY-C4 | | 4.5 | | -1 | | 1 | | 21 | | 13522 | | 13542 | | AAAGUUAGAGAAGUUUGACUU | | AUGUUAUAUGUCUCUAACUUA | | Cleavage | |  | | 2 | |  |
| sbi-miR437n | SbNFY-C4 | | 4.5 | | -1 | | 1 | | 21 | | 5396 | | 5416 | | AAAGUUAGAGAAGUUUGACUU | | UUUUAAAAUUUUACUAACUUU | | Cleavage | |  | | 2 | |  |
| sbi-miR437o | SbNFY-C7 | | 4.5 | | -1 | | 1 | | 21 | | 10653 | | 10673 | | AAAGUUAGAGAAGUUUGACUU | | AAUUCAUACUUUUUUAAGUUU | | Cleavage | |  | | 2 | |  |
| sbi-miR437o | SbNFY-C4 | | 4.5 | | -1 | | 1 | | 21 | | 13522 | | 13542 | | AAAGUUAGAGAAGUUUGACUU | | AUGUUAUAUGUCUCUAACUUA | | Cleavage | |  | | 2 | |  |
| sbi-miR437o | SbNFY-C4 | | 4.5 | | -1 | | 1 | | 21 | | 5396 | | 5416 | | AAAGUUAGAGAAGUUUGACUU | | UUUUAAAAUUUUACUAACUUU | | Cleavage | |  | | 2 | |  |
| sbi-miR437p | SbNFY-C7 | | 4.5 | | -1 | | 1 | | 21 | | 10653 | | 10673 | | AAAGUUAGAGAAGUUUGACUU | | AAUUCAUACUUUUUUAAGUUU | | Cleavage | |  | | 2 | |  |
| sbi-miR437p | SbNFY-C4 | | 4.5 | | -1 | | 1 | | 21 | | 13522 | | 13542 | | AAAGUUAGAGAAGUUUGACUU | | AUGUUAUAUGUCUCUAACUUA | | Cleavage | |  | | 2 | |  |
| sbi-miR437p | SbNFY-C4 | | 4.5 | | -1 | | 1 | | 21 | | 5396 | | 5416 | | AAAGUUAGAGAAGUUUGACUU | | UUUUAAAAUUUUACUAACUUU | | Cleavage | |  | | 2 | |  |
| sbi-miR437q | SbNFY-C7 | | 4.5 | | -1 | | 1 | | 21 | | 10653 | | 10673 | | AAAGUUAGAGAAGUUUGACUU | | AAUUCAUACUUUUUUAAGUUU | | Cleavage | |  | | 2 | |  |
| sbi-miR437q | SbNFY-C4 | | 4.5 | | -1 | | 1 | | 21 | | 13522 | | 13542 | | AAAGUUAGAGAAGUUUGACUU | | AUGUUAUAUGUCUCUAACUUA | | Cleavage | |  | | 2 | |  |
| sbi-miR437q | SbNFY-C4 | | 4.5 | | -1 | | 1 | | 21 | | 5396 | | 5416 | | AAAGUUAGAGAAGUUUGACUU | | UUUUAAAAUUUUACUAACUUU | | Cleavage | |  | | 2 | |  |
| sbi-miR437r | SbNFY-C7 | | 4.5 | | -1 | | 1 | | 21 | | 10653 | | 10673 | | AAAGUUAGAGAAGUUUGACUU | | AAUUCAUACUUUUUUAAGUUU | | Cleavage | |  | | 2 | |  |
| sbi-miR437r | SbNFY-C4 | | 4.5 | | -1 | | 1 | | 21 | | 13522 | | 13542 | | AAAGUUAGAGAAGUUUGACUU | | AUGUUAUAUGUCUCUAACUUA | | Cleavage | |  | | 2 | |  |
| sbi-miR437r | SbNFY-C4 | | 4.5 | | -1 | | 1 | | 21 | | 5396 | | 5416 | | AAAGUUAGAGAAGUUUGACUU | | UUUUAAAAUUUUACUAACUUU | | Cleavage | |  | | 2 | |  |
| sbi-miR437s | SbNFY-C7 | | 4.5 | | -1 | | 1 | | 21 | | 10653 | | 10673 | | AAAGUUAGAGAAGUUUGACUU | | AAUUCAUACUUUUUUAAGUUU | | Cleavage | |  | | 2 | |  |
| sbi-miR437s | SbNFY-C4 | | 4.5 | | -1 | | 1 | | 21 | | 13522 | | 13542 | | AAAGUUAGAGAAGUUUGACUU | | AUGUUAUAUGUCUCUAACUUA | | Cleavage | |  | | 2 | |  |
| sbi-miR437s | SbNFY-C4 | | 4.5 | | -1 | | 1 | | 21 | | 5396 | | 5416 | | AAAGUUAGAGAAGUUUGACUU | | UUUUAAAAUUUUACUAACUUU | | Cleavage | |  | | 2 | |  |
| sbi-miR437t | SbNFY-C7 | | 4.5 | | -1 | | 1 | | 21 | | 10653 | | 10673 | | AAAGUUAGAGAAGUUUGACUU | | AAUUCAUACUUUUUUAAGUUU | | Cleavage | |  | | 2 | |  |
| sbi-miR437t | SbNFY-C4 | | 4.5 | | -1 | | 1 | | 21 | | 13522 | | 13542 | | AAAGUUAGAGAAGUUUGACUU | | AUGUUAUAUGUCUCUAACUUA | | Cleavage | |  | | 2 | |  |
| sbi-miR437t | SbNFY-C4 | | 4.5 | | -1 | | 1 | | 21 | | 5396 | | 5416 | | AAAGUUAGAGAAGUUUGACUU | | UUUUAAAAUUUUACUAACUUU | | Cleavage | |  | | 2 | |  |
| sbi-miR437u | SbNFY-C7 | | 4.5 | | -1 | | 1 | | 21 | | 10653 | | 10673 | | AAAGUUAGAGAAGUUUGACUU | | AAUUCAUACUUUUUUAAGUUU | | Cleavage | |  | | 2 | |  |
| sbi-miR437u | SbNFY-C4 | | 4.5 | | -1 | | 1 | | 21 | | 13522 | | 13542 | | AAAGUUAGAGAAGUUUGACUU | | AUGUUAUAUGUCUCUAACUUA | | Cleavage | |  | | 2 | |  |
| sbi-miR437u | SbNFY-C4 | | 4.5 | | -1 | | 1 | | 21 | | 5396 | | 5416 | | AAAGUUAGAGAAGUUUGACUU | | UUUUAAAAUUUUACUAACUUU | | Cleavage | |  | | 2 | |  |
| sbi-miR437v | SbNFY-C7 | | 4.5 | | -1 | | 1 | | 21 | | 10653 | | 10673 | | AAAGUUAGAGAAGUUUGACUU | | AAUUCAUACUUUUUUAAGUUU | | Cleavage | |  | | 2 | |  |
| sbi-miR437v | SbNFY-C4 | | 4.5 | | -1 | | 1 | | 21 | | 13522 | | 13542 | | AAAGUUAGAGAAGUUUGACUU | | AUGUUAUAUGUCUCUAACUUA | | Cleavage | |  | | 2 | |  |
| sbi-miR437v | SbNFY-C4 | | 4.5 | | -1 | | 1 | | 21 | | 5396 | | 5416 | | AAAGUUAGAGAAGUUUGACUU | | UUUUAAAAUUUUACUAACUUU | | Cleavage | |  | | 2 | |  |
| sbi-miR437w | SbNFY-C7 | | 4.5 | | -1 | | 1 | | 21 | | 10653 | | 10673 | | AAAGUUAGAGAAGUUUGACUU | | AAUUCAUACUUUUUUAAGUUU | | Cleavage | |  | | 2 | |  |
| sbi-miR437w | SbNFY-C4 | | 4.5 | | -1 | | 1 | | 21 | | 13522 | | 13542 | | AAAGUUAGAGAAGUUUGACUU | | AUGUUAUAUGUCUCUAACUUA | | Cleavage | |  | | 2 | |  |
| sbi-miR437w | SbNFY-C4 | | 4.5 | | -1 | | 1 | | 21 | | 5396 | | 5416 | | AAAGUUAGAGAAGUUUGACUU | | UUUUAAAAUUUUACUAACUUU | | Cleavage | |  | | 2 | |  |
| sbi-miR529 | SbNFY-C3 | | 4.5 | | -1 | | 1 | | 20 | | 13643 | | 13662 | | CUGUACCCUCUCUCUUCUUC | | GAGGAGGAGGGAUGGCACAG | | Cleavage | |  | | 1 | |  |
| sbi-miR529 | SbNFY-C11 | | 4.5 | | -1 | | 1 | | 20 | | 10786 | | 10805 | | CUGUACCCUCUCUCUUCUUC | | AAAAAAGGGAAAAGGUACAG | | Translation | | | | 2 | |  |
| sbi-miR5386 | SbNFY-C14 | | 4.5 | | -1 | | 1 | | 20 | | 1873 | | 1892 | | CGUCGCUGUCGCGCGCGCUG | | GACCGCGUGUGACGGUGAAG | | Cleavage | |  | | 2 | |  |
| sbi-miR5389 | SbNFY-C15 | | 4.5 | | -1 | | 1 | | 21 | | 5300 | | 5320 | | GCUUGAGUUUAUCAGCCGAGU | | UAUUGGUCAAUGAAUUCAAGC | | Cleavage | |  | | 1 | |  |
| sbi-miR5564c-5p | SbNFY-C7 | | 4.5 | | -1 | | 1 | | 21 | | 8010 | | 8030 | | AAUUCGUCGAACAGCUGCAGC | | CCGGCGGGUGUUCGACGGCUU | | Cleavage | |  | | 2 | |  |
| sbi-miR5564c-5p | SbNFY-C1 | | 4.5 | | -1 | | 1 | | 21 | | 10102 | | 10122 | | AAUUCGUCGAACAGCUGCAGC | | CGACUAGAUGUUUGAUGAAUU | | Cleavage | |  | | 1 | |  |
| sbi-miR5564c-5p | SbNFY-C6 | | 4.5 | | -1 | | 1 | | 21 | | 7366 | | 7386 | | AAUUCGUCGAACAGCUGCAGC | | AAUGGAUCUCUUUGAUGAAUU | | Cleavage | |  | | 1 | |  |
| sbi-miR5565c | SbNFY-C4 | | 4.5 | | -1 | | 1 | | 21 | | 2789 | | 2810 | | UACACAUGUGGAUUG-AGGUGA | | UGACUUGCGGUUCACAUGUGUG | | Cleavage | |  | | 1 | |  |
| sbi-miR5565d | SbNFY-C6 | | 4.5 | | -1 | | 1 | | 24 | | 5914 | | 5937 | | ACUUCAAUCCAUGUAUGUUGGUGU | | CAUGGAACCAACGUGAGUUGAAGU | | Cleavage | |  | | 1 | |  |
| sbi-miR5567 | SbNFY-C7 | | 4.5 | | -1 | | 1 | | 24 | | 9533 | | 9556 | | UUAAUGAUUCAUGUAUGUGUCCAA | | UUGGUCGCAUGCAUAGAGCAUUAA | | Translation | | | | 4 | |  |
| sbi-miR5567 | SbNFY-C5 | | 4.5 | | -1 | | 1 | | 24 | | 7282 | | 7305 | | UUAAUGAUUCAUGUAUGUGUCCAA | | UGAAGCACAUGCAUAGGGCAUUAA | | Translation | | | | 3 | |  |
| sbi-miR5567 | SbNFY-C3 | | 4.5 | | -1 | | 1 | | 24 | | 1627 | | 1650 | | UUAAUGAUUCAUGUAUGUGUCCAA | | AAUCACACCAAUGUGAACCAUUAA | | Cleavage | |  | | 2 | |  |
| sbi-miR5567 | SbNFY-C9 | | 4.5 | | -1 | | 1 | | 24 | | 10651 | | 10674 | | UUAAUGAUUCAUGUAUGUGUCCAA | | UAUGGCACAUGUAUAGAACAUUAA | | Translation | | | | 1 | |  |
| sbi-miR5567 | SbNFY-C6 | | 4.5 | | -1 | | 1 | | 24 | | 10993 | | 11016 | | UUAAUGAUUCAUGUAUGUGUCCAA | | CCUGGCAAAUAUGUGAACCAUUAC | | Cleavage | |  | | 1 | |  |
| sbi-miR5568a | SbNFY-C1 | | 4.5 | | -1 | | 1 | | 21 | | 14478 | | 14498 | | CAGAGCGACUUACAAUUUGGA | | UCCAAAUUGUUAGAUGUUUUG | | Translation | | | | 1 | |  |
| sbi-miR5568b-3p | SbNFY-C14 | | 4.5 | | -1 | | 1 | | 21 | | 5182 | | 5202 | | ACUAUGUAUCUAGAAAAGCUA | | UUGCUUUUAUAGGUACAUAUC | | Cleavage | |  | | 1 | |  |
| sbi-miR5568b-3p | SbNFY-C2 | | 4.5 | | -1 | | 1 | | 21 | | 2257 | | 2277 | | ACUAUGUAUCUAGAAAAGCUA | | AUGUUUUUUUAUAUAUAUAUU | | Translation | | | | 1 | |  |
| sbi-miR5568b-5p | SbNFY-C3 | | 4.5 | | -1 | | 1 | | 21 | | 12602 | | 12622 | | UUUCUAGGUACAUAGCUUUUG | | AUAAAAUUAUGAACCAAGAAA | | Translation | | | | 2 | |  |
| sbi-miR5568b-5p | SbNFY-C15 | | 4.5 | | -1 | | 1 | | 21 | | 15190 | | 15210 | | UUUCUAGGUACAUAGCUUUUG | | UUAGAUCUAUGUACAUAAAAA | | Cleavage | |  | | 1 | |  |
| sbi-miR5568c-3p | SbNFY-C11 | | 4.5 | | -1 | | 1 | | 21 | | 2529 | | 2549 | | ACUUACAGUUUGGAACGGAGG | | UACUCUUUCCAAAUUAUAAGU | | Cleavage | |  | | 2 | |  |
| sbi-miR5568c-3p | SbNFY-C1 | | 4.5 | | -1 | | 1 | | 21 | | 14471 | | 14491 | | ACUUACAGUUUGGAACGGAGG | | CUUCUAUUCCAAAUUGUUAGA | | Cleavage | |  | | 2 | |  |
| sbi-miR5568c-3p | SbNFY-C10 | | 4.5 | | -1 | | 1 | | 21 | | 3666 | | 3686 | | ACUUACAGUUUGGAACGGAGG | | CCUCCAUUCCAAAUUAUAAUU | | Cleavage | |  | | 1 | |  |
| sbi-miR5568c-3p | SbNFY-C6 | | 4.5 | | -1 | | 1 | | 21 | | 6808 | | 6828 | | ACUUACAGUUUGGAACGGAGG | | UGCCUGUUUUAGUCUGUAAGU | | Cleavage | |  | | 1 | |  |
| sbi-miR5568c-5p | SbNFY-C11 | | 4.5 | | -1 | | 1 | | 21 | | 2645 | | 2665 | | UCUGUUCCAAAUUGUAAGUCG | | CAACUUAUAAUUUGGAACUAA | | Cleavage | |  | | 2 | |  |
| sbi-miR5568c-5p | SbNFY-C5 | | 4.5 | | -1 | | 1 | | 21 | | 2130 | | 2149 | | UCUGUUCCAAAUUGUAAGUCG | | UGGCUU-CAAUUAGGAACGGA | | Cleavage | |  | | 1 | |  |
| sbi-miR5568d-3p | SbNFY-C13 | | 4.5 | | -1 | | 1 | | 21 | | 996 | | 1016 | | AAAGUUGUGUAUCUAGAAAAG | | GUUUGCUUGUUACAUGACUUU | | Cleavage | |  | | 1 | |  |
| sbi-miR5568d-5p | SbNFY-C8 | | 4.5 | | -1 | | 1 | | 21 | | 12847 | | 12867 | | UGGCUUUUCUAGAUACAUAGC | | UAUAUGUAUUUUGAGAAACUA | | Translation | | | | 2 | |  |
| sbi-miR5568e-5p | SbNFY-C7 | | 4.5 | | -1 | | 1 | | 21 | | 5249 | | 5269 | | GAUGUUUUGGGUUUUCUAGAU | | ACCCAGAAAGCUCAAAUUAUU | | Cleavage | |  | | 3 | |  |
| sbi-miR5568e-5p | SbNFY-C12 | | 4.5 | | -1 | | 1 | | 21 | | 10851 | | 10871 | | GAUGUUUUGGGUUUUCUAGAU | | AGCUAGAAAUCCCAAGGCCUU | | Cleavage | |  | | 2 | |  |
| sbi-miR5568e-5p | SbNFY-C1 | | 4.5 | | -1 | | 1 | | 21 | | 1741 | | 1761 | | GAUGUUUUGGGUUUUCUAGAU | | UUUUUGGAGAGUCAAAACAUC | | Translation | | | | 2 | |  |
| sbi-miR5568e-5p | SbNFY-C4 | | 4.5 | | -1 | | 1 | | 21 | | 8011 | | 8031 | | GAUGUUUUGGGUUUUCUAGAU | | UACUAUAGAACUCAAAUUAUU | | Cleavage | |  | | 1 | |  |
| sbi-miR5568e-5p | SbNFY-C11 | | 4.5 | | -1 | | 1 | | 21 | | 13648 | | 13669 | | GAUGUUUUGGGUUUU-CUAGAU | | CAAUAGCAGAACCUGAAACAUC | | Cleavage | |  | | 1 | |  |
| sbi-miR5568f-3p | SbNFY-C9 | | 4.5 | | -1 | | 1 | | 21 | | 8004 | | 8024 | | GUCUUAUAAUUUGGAAUGGAG | | UCCUGAUACAAAAUAUAAGAC | | Cleavage | |  | | 2 | |  |
| sbi-miR5568f-3p | SbNFY-C12 | | 4.5 | | -1 | | 1 | | 21 | | 4524 | | 4544 | | GUCUUAUAAUUUGGAAUGGAG | | AUCUAUACUAAAUUAAAGGAU | | Cleavage | |  | | 2 | |  |
| sbi-miR5568f-5p | SbNFY-C13 | | 4.5 | | -1 | | 1 | | 21 | | 5062 | | 5082 | | UCCAUUCCAAAUUGUAAGAUG | | CAUUGUGUCAUUUGGAAUGGG | | Cleavage | |  | | 1 | |  |
| sbi-miR5568g-3p | SbNFY-C14 | | 4.5 | | -1 | | 1 | | 21 | | 5163 | | 5183 | | AAAACGUCUUAUAAUUUGGAG | | UUUUAAAUUAUAAAUUGUUUU | | Cleavage | |  | | 1 | |  |
| sbi-miR5570 | SbNFY-C10 | | 4.5 | | -1 | | 1 | | 21 | | 3359 | | 3379 | | AAAAGACAAAUCAGCAUGUCA | | GUACAUGUUUGUUUGUUUGUU | | Cleavage | |  | | 1 | |  |
| sbi-miR5570 | SbNFY-C13 | | 4.5 | | -1 | | 1 | | 21 | | 4359 | | 4379 | | AAAAGACAAAUCAGCAUGUCA | | AAGCAUGUAGGUUUUUCUUUU | | Cleavage | |  | | 1 | |  |
| sbi-miR5570 | SbNFY-C7 | | 4.5 | | -1 | | 1 | | 21 | | 16550 | | 16569 | | AAAAGACAAAUCAGCAUGUCA | | AGAUAUG-UAAUUUGUUUUUU | | Cleavage | |  | | 1 | |  |
| sbi-miR5570 | SbNFY-C1 | | 4.5 | | -1 | | 1 | | 21 | | 164 | | 185 | | AAAAGACAAAUCAG-CAUGUCA | | AAACAUUUCUGAUUUGUAUUUU | | Cleavage | |  | | 2 | |  |
| sbi-miR5570 | SbNFY-C3 | | 4.5 | | -1 | | 1 | | 21 | | 3177 | | 3197 | | AAAAGACAAAUCAGCAUGUCA | | AUGUAUUAUGAUGUGUCUUUU | | Cleavage | |  | | 1 | |  |
| sbi-miR6217a-3p | SbNFY-C1 | | 4.5 | | -1 | | 1 | | 24 | | 13847 | | 13870 | | AAAAUUAUCGUAAAUAGAGGUGGC | | AAGUCCAUUAUUUACGACAAUCUU | | Cleavage | |  | | 1 | |  |
| sbi-miR6217a-5p | SbNFY-C1 | | 4.5 | | -1 | | 1 | | 24 | | 14040 | | 14063 | | UAGCCACUUUGAGUUACGAUAAUU | | GAUUGUUGAAACACAAAGUGUCUA | | Cleavage | |  | | 1 | |  |
| sbi-miR6217b-3p | SbNFY-C1 | | 4.5 | | -1 | | 1 | | 24 | | 13847 | | 13870 | | AAAAUUAUCGUAAAUAGAGGUGGC | | AAGUCCAUUAUUUACGACAAUCUU | | Cleavage | |  | | 1 | |  |
| sbi-miR6217b-5p | SbNFY-C1 | | 4.5 | | -1 | | 1 | | 24 | | 14040 | | 14063 | | UAGCCACUUUGAGUUACGAUAAUU | | GAUUGUUGAAACACAAAGUGUCUA | | Cleavage | |  | | 1 | |  |
| sbi-miR6218-5p | SbNFY-C10 | | 4.5 | | -1 | | 1 | | 21 | | 15101 | | 15121 | | CGAAAAUCACGAAACUUGUCG | | AGAAAAGAUUUGAGAUUUUUG | | Cleavage | |  | | 1 | |  |
| sbi-miR6220-3p | SbNFY-C1 | | 4.5 | | -1 | | 1 | | 24 | | 4090 | | 4113 | | AUGCCUUAUAAUUUGGGAUGGAGA | | UCUCCAGUCCAAUUUAUAAGACAU | | Cleavage | |  | | 3 | |  |
| sbi-miR6220-3p | SbNFY-C3 | | 4.5 | | -1 | | 1 | | 24 | | 11200 | | 11223 | | AUGCCUUAUAAUUUGGGAUGGAGA | | CCUCCAUUCCAAACUAUAAGUCAC | | Translation | | | | 1 | |  |
| sbi-miR6220-3p | SbNFY-C11 | | 4.5 | | -1 | | 1 | | 24 | | 11667 | | 11690 | | AUGCCUUAUAAUUUGGGAUGGAGA | | ACUCUGUUUCAAAUUAUAAGUCGC | | Cleavage | |  | | 2 | |  |
| sbi-miR6220-3p | SbNFY-C2 | | 4.5 | | -1 | | 1 | | 24 | | 14854 | | 14877 | | AUGCCUUAUAAUUUGGGAUGGAGA | | CAUCAAUUUUGAAUUGUAAGGUGC | | Cleavage | |  | | 1 | |  |
| sbi-miR6220-5p | SbNFY-C12 | | 4.5 | | -1 | | 1 | | 24 | | 8138 | | 8161 | | CUCCAUCCUAAAUUAUAAGACAUU | | UGCCCGUUAAAAUUAAGGGUGGGG | | Translation | | | | 1 | |  |
| sbi-miR6222-5p | SbNFY-C9 | | 4.5 | | -1 | | 1 | | 21 | | 17561 | | 17581 | | CCUGUUUGGAUCAGCCAAGGC | | GAUCUGAUUGGUCCGAACAGG | | Cleavage | |  | | 1 | |  |
| sbi-miR6223-5p | SbNFY-C11 | | 4.5 | | -1 | | 1 | | 21 | | 14888 | | 14908 | | UUCUUGGGAGGAGCAUGCUAG | | CUAAAAUGGUUCUUCCAAGAA | | Cleavage | |  | | 1 | |  |
| sbi-miR6225-3p | SbNFY-C2 | | 4.5 | | -1 | | 1 | | 24 | | 11055 | | 11078 | | GAAACGAAUCUUUUAAGUCUAAUU | | AACUAGGCUCAAAAAAUUCGUCUC | | Translation | | | | 2 | |  |
| sbi-miR6226-3p | SbNFY-C11 | | 4.5 | | -1 | | 1 | | 24 | | 12366 | | 12389 | | GAUUAGUCACGAUUAGUCGUCCGA | | CAGGACGACUAAUCGCGAUUAACG | | Cleavage | |  | | 2 | |  |
| sbi-miR6226-3p | SbNFY-C11 | | 4.5 | | -1 | | 1 | | 24 | | 11859 | | 11882 | | GAUUAGUCACGAUUAGUCGUCCGA | | CCAGAUGAUUAAUCUUGAUUUAUC | | Translation | | | | 2 | |  |
| sbi-miR6226-5p | SbNFY-C8 | | 4.5 | | -1 | | 1 | | 24 | | 455 | | 478 | | AGAUCGGACGACUAAUCGCGAUUA | | GCUCGGGGACUGGUCGUCCAGUCU | | Cleavage | |  | | 1 | |  |
| sbi-miR6228-3p | SbNFY-C7 | | 4.5 | | -1 | | 1 | | 24 | | 15259 | | 15282 | | GUGGCAGUAGAAUUAAUGAAGGGA | | AUAGCAUGUUAUUUUUACUGCUAC | | Cleavage | |  | | 1 | |  |
| sbi-miR6228-5p | SbNFY-C10 | | 4.5 | | -1 | | 1 | | 24 | | 4323 | | 4346 | | UUCUAUCUCUAUUAAUUGUGUUGC | | CAAAUACAAGUAAUAGACGGAGAA | | Cleavage | |  | | 1 | |  |
| sbi-miR6228-5p | SbNFY-C14 | | 4.5 | | -1 | | 1 | | 24 | | 6260 | | 6283 | | UUCUAUCUCUAUUAAUUGUGUUGC | | AGCCUAGAUUCAAUAGAGAUAUAA | | Cleavage | |  | | 1 | |  |
| sbi-miR6229-3p | SbNFY-C13 | | 4.5 | | -1 | | 1 | | 24 | | 7521 | | 7544 | | GUUUUUCUCGCCGGGUGAGAAGGC | | UACAACUCAUUUGGCUAGAAAAGA | | Cleavage | |  | | 2 | |  |
| sbi-miR6229-3p | SbNFY-C13 | | 4.5 | | -1 | | 1 | | 24 | | 7657 | | 7680 | | GUUUUUCUCGCCGGGUGAGAAGGC | | UACACCUCAUUUGGCUAGAAAAGA | | Cleavage | |  | | 2 | |  |
| sbi-miR6230-3p | SbNFY-C3 | | 4.5 | | -1 | | 1 | | 21 | | 4314 | | 4335 | | UAACAAGUUUAGGGA-UCUAGA | | ACUAGACUAUCUAAAUUUGUUG | | Cleavage | |  | | 2 | |  |
| sbi-miR6230-3p | SbNFY-C15 | | 4.5 | | -1 | | 1 | | 21 | | 15275 | | 15295 | | UAACAAGUUUAGGGAUCUAGA | | UGUAAACCCUUGGAUUUGUUG | | Cleavage | |  | | 1 | |  |
| sbi-miR6230-5p | SbNFY-C6 | | 4.5 | | -1 | | 1 | | 21 | | 4162 | | 4182 | | UUUUGGGUCCCUAAACUUGUU | | UUUAAGUUUAGAGACCAAAAU | | Translation | | | | 4 | |  |
| sbi-miR6232b-3p | SbNFY-C5 | | 4.5 | | -1 | | 1 | | 21 | | 7260 | | 7280 | | AAUUCGAUGUACCAAAAAAGU | | GAUUCUUCGUUACAUCGAAUC | | Cleavage | |  | | 1 | |  |
| sbi-miR6232b-5p | SbNFY-C15 | | 4.5 | | -1 | | 1 | | 21 | | 17773 | | 17793 | | UUUUUGGUACAUUGAAUUUGC | | CAAGAUUCGAUGUGACAGGGA | | Cleavage | |  | | 2 | |  |
| sbi-miR6232b-5p | SbNFY-C12 | | 4.5 | | -1 | | 1 | | 21 | | 13549 | | 13569 | | UUUUUGGUACAUUGAAUUUGC | | CAAGAUUCGAUGUGACGGGAA | | Cleavage | |  | | 5 | |  |
| sbi-miR6232b-5p | SbNFY-C12 | | 4.5 | | -1 | | 1 | | 21 | | 8496 | | 8516 | | UUUUUGGUACAUUGAAUUUGC | | AAAGAUUUGAUGUGACAGAGA | | Cleavage | |  | | 5 | |  |
| sbi-miR6232b-5p | SbNFY-C1 | | 4.5 | | -1 | | 1 | | 21 | | 8048 | | 8068 | | UUUUUGGUACAUUGAAUUUGC | | UAAGGUUUGAUGUGACAAGAA | | Cleavage | |  | | 3 | |  |
| sbi-miR6232b-5p | SbNFY-C3 | | 4.5 | | -1 | | 1 | | 21 | | 8640 | | 8660 | | UUUUUGGUACAUUGAAUUUGC | | CCAAGUUUGGUAUGCUAAAAA | | Translation | | | | 1 | |  |
| sbi-miR6232b-5p | SbNFY-C11 | | 4.5 | | -1 | | 1 | | 21 | | 6714 | | 6734 | | UUUUUGGUACAUUGAAUUUGC | | AAAGAUUCGAUGUGACGGAGA | | Cleavage | |  | | 4 | |  |
| sbi-miR6232b-5p | SbNFY-C11 | | 4.5 | | -1 | | 1 | | 21 | | 13052 | | 13072 | | UUUUUGGUACAUUGAAUUUGC | | AUGAGAUGAAUGUGCUGAAAA | | Cleavage | |  | | 4 | |  |
| sbi-miR6232b-5p | SbNFY-C13 | | 4.5 | | -1 | | 1 | | 21 | | 13259 | | 13279 | | UUUUUGGUACAUUGAAUUUGC | | UUACAAUCAAUGUGUCAAGAU | | Cleavage | |  | | 2 | |  |
| sbi-miR6233-3p | SbNFY-C6 | | 4.5 | | -1 | | 1 | | 24 | | 12860 | | 12883 | | CAAGUUUGGUUUUGGUAAUUAAUG | | UGGUAAAUAUCAAAACUGAUUUUG | | Cleavage | |  | | 2 | |  |
| sbi-miR6233-3p | SbNFY-C2 | | 4.5 | | -1 | | 1 | | 24 | | 16607 | | 16630 | | CAAGUUUGGUUUUGGUAAUUAAUG | | CAUUCAUUAGAAGAACGAAGCUUG | | Cleavage | |  | | 1 | |  |
| sbi-miR6233-3p | SbNFY-C11 | | 4.5 | | -1 | | 1 | | 24 | | 2266 | | 2289 | | CAAGUUUGGUUUUGGUAAUUAAUG | | AUGCAAGUGCUAAAACCAGGAUUG | | Cleavage | |  | | 1 | |  |
| sbi-miR6233-3p | SbNFY-C12 | | 4.5 | | -1 | | 1 | | 24 | | 12633 | | 12656 | | CAAGUUUGGUUUUGGUAAUUAAUG | | UCUCUUUUAUCAAGAACAAACUUA | | Cleavage | |  | | 1 | |  |
| sbi-miR6233-3p | SbNFY-C10 | | 4.5 | | -1 | | 1 | | 24 | | 16564 | | 16587 | | CAAGUUUGGUUUUGGUAAUUAAUG | | AAAGCAAUGCAAGGACCAAACUUU | | Cleavage | |  | | 1 | |  |
| sbi-miR6235-5p | SbNFY-C4 | | 4.5 | | -1 | | 1 | | 24 | | 10806 | | 10829 | | UUGUGAGAGAAAAAUACUGUUGGC | | UUCUGUAUCAUUUUUCUUUCAUAC | | Cleavage | |  | | 3 | |  |
| sbi-miR6235-5p | SbNFY-C8 | | 4.5 | | -1 | | 1 | | 24 | | 6092 | | 6115 | | UUGUGAGAGAAAAAUACUGUUGGC | | UAAUACUGUAUUUUUUUUUCUCAU | | Cleavage | |  | | 1 | |  |
| sbi-miR821d | SbNFY-C4 | | 4.5 | | -1 | | 1 | | 21 | | 9332 | | 9352 | | AAGUCAUCAACAACAAAGUUG | | CAGAUUUGUUGUUUGUGACUG | | Cleavage | |  | | 1 | |  |
| sbi-miR821d | SbNFY-C13 | | 4.5 | | -1 | | 1 | | 21 | | 9739 | | 9759 | | AAGUCAUCAACAACAAAGUUG | | AUCAUUUCUUGUUGAUGACAU | | Cleavage | |  | | 1 | |  |
| sbi-miR1435a | SbNFY-C2 | | 5 | | -1 | | 1 | | 20 | | 1121 | | 1140 | | UUUCUUAAGUCAAACUUUUC | | UGAGGGUUUGGCUUUGGAAU | | Cleavage | |  | | 2 | |  |
| sbi-miR1435a | SbNFY-C2 | | 5 | | -1 | | 1 | | 20 | | 14761 | | 14780 | | UUUCUUAAGUCAAACUUUUC | | CAAAAGAUUGAGUCAGGAGA | | Cleavage | |  | | 2 | |  |
| sbi-miR1435a | SbNFY-C5 | | 5 | | -1 | | 1 | | 20 | | 465 | | 484 | | UUUCUUAAGUCAAACUUUUC | | UAGGUGUUUGGUUUGAGUAA | | Cleavage | |  | | 1 | |  |
| sbi-miR1435a | SbNFY-C8 | | 5 | | -1 | | 1 | | 20 | | 13074 | | 13093 | | UUUCUUAAGUCAAACUUUUC | | AAGAAUUUUGGAUGAAGAAA | | Cleavage | |  | | 1 | |  |
| sbi-miR1435b | SbNFY-C5 | | 5 | | -1 | | 1 | | 20 | | 465 | | 484 | | UUUCUUAAGUCAAACCUUUU | | UAGGUGUUUGGUUUGAGUAA | | Cleavage | |  | | 1 | |  |
| sbi-miR156a | SbNFY-C4 | | 5 | | -1 | | 1 | | 20 | | 11098 | | 11117 | | UGACAGAAGAGAGUGAGCAC | | GUGCUCACAUUUUGUUGUUA | | Cleavage | |  | | 1 | |  |
| sbi-miR156a | SbNFY-C9 | | 5 | | -1 | | 1 | | 20 | | 7861 | | 7880 | | UGACAGAAGAGAGUGAGCAC | | AUCCUUAUUUUUUUCCGUCG | | Cleavage | |  | | 1 | |  |
| sbi-miR156b | SbNFY-C4 | | 5 | | -1 | | 1 | | 20 | | 11098 | | 11117 | | UGACAGAAGAGAGUGAGCAC | | GUGCUCACAUUUUGUUGUUA | | Cleavage | |  | | 1 | |  |
| sbi-miR156b | SbNFY-C9 | | 5 | | -1 | | 1 | | 20 | | 7861 | | 7880 | | UGACAGAAGAGAGUGAGCAC | | AUCCUUAUUUUUUUCCGUCG | | Cleavage | |  | | 1 | |  |
| sbi-miR156c | SbNFY-C4 | | 5 | | -1 | | 1 | | 20 | | 11098 | | 11117 | | UGACAGAAGAGAGUGAGCAC | | GUGCUCACAUUUUGUUGUUA | | Cleavage | |  | | 1 | |  |
| sbi-miR156c | SbNFY-C9 | | 5 | | -1 | | 1 | | 20 | | 7861 | | 7880 | | UGACAGAAGAGAGUGAGCAC | | AUCCUUAUUUUUUUCCGUCG | | Cleavage | |  | | 1 | |  |
| sbi-miR156d | SbNFY-C6 | | 5 | | -1 | | 1 | | 21 | | 10537 | | 10557 | | UGACAGAAGAGAGAGAGCACA | | CCAAAUCUUUCUCUUCUAUCA | | Cleavage | |  | | 2 | |  |
| sbi-miR156d | SbNFY-C6 | | 5 | | -1 | | 1 | | 21 | | 617 | | 637 | | UGACAGAAGAGAGAGAGCACA | | GUUUUUCUCUUUCUUCUUUUC | | Cleavage | |  | | 2 | |  |
| sbi-miR156d | SbNFY-C1 | | 5 | | -1 | | 1 | | 21 | | 6322 | | 6342 | | UGACAGAAGAGAGAGAGCACA | | AAUGCUUUUUUUACUUUGUCA | | Cleavage | |  | | 1 | |  |
| sbi-miR156d | SbNFY-C7 | | 5 | | -1 | | 1 | | 21 | | 15154 | | 15174 | | UGACAGAAGAGAGAGAGCACA | | CCUCUUUUUUUUUUUCUGUUU | | Cleavage | |  | | 1 | |  |
| sbi-miR156f | SbNFY-C4 | | 5 | | -1 | | 1 | | 20 | | 11098 | | 11117 | | UGACAGAAGAGAGUGAGCAC | | GUGCUCACAUUUUGUUGUUA | | Cleavage | |  | | 1 | |  |
| sbi-miR156f | SbNFY-C9 | | 5 | | -1 | | 1 | | 20 | | 7861 | | 7880 | | UGACAGAAGAGAGUGAGCAC | | AUCCUUAUUUUUUUCCGUCG | | Cleavage | |  | | 1 | |  |
| sbi-miR156g | SbNFY-C4 | | 5 | | -1 | | 1 | | 20 | | 11098 | | 11117 | | UGACAGAAGAGAGUGAGCAC | | GUGCUCACAUUUUGUUGUUA | | Cleavage | |  | | 1 | |  |
| sbi-miR156g | SbNFY-C9 | | 5 | | -1 | | 1 | | 20 | | 7861 | | 7880 | | UGACAGAAGAGAGUGAGCAC | | AUCCUUAUUUUUUUCCGUCG | | Cleavage | |  | | 1 | |  |
| sbi-miR156h | SbNFY-C4 | | 5 | | -1 | | 1 | | 20 | | 11098 | | 11117 | | UGACAGAAGAGAGUGAGCAC | | GUGCUCACAUUUUGUUGUUA | | Cleavage | |  | | 1 | |  |
| sbi-miR156h | SbNFY-C9 | | 5 | | -1 | | 1 | | 20 | | 7861 | | 7880 | | UGACAGAAGAGAGUGAGCAC | | AUCCUUAUUUUUUUCCGUCG | | Cleavage | |  | | 1 | |  |
| sbi-miR156i | SbNFY-C4 | | 5 | | -1 | | 1 | | 20 | | 11098 | | 11117 | | UGACAGAAGAGAGUGAGCAC | | GUGCUCACAUUUUGUUGUUA | | Cleavage | |  | | 1 | |  |
| sbi-miR156i | SbNFY-C9 | | 5 | | -1 | | 1 | | 20 | | 7861 | | 7880 | | UGACAGAAGAGAGUGAGCAC | | AUCCUUAUUUUUUUCCGUCG | | Cleavage | |  | | 1 | |  |
| sbi-miR159a | SbNFY-C13 | | 5 | | -1 | | 1 | | 21 | | 10383 | | 10403 | | UUUGGAUUGAAGGGAGCUCUG | | UAAAGCUUCCUUCAACUAAAA | | Cleavage | |  | | 1 | |  |
| sbi-miR159a | SbNFY-C6 | | 5 | | -1 | | 1 | | 21 | | 6318 | | 6338 | | UUUGGAUUGAAGGGAGCUCUG | | UUGAACUCUUUUUGAUGUAAA | | Cleavage | |  | | 2 | |  |
| sbi-miR159a | SbNFY-C3 | | 5 | | -1 | | 1 | | 21 | | 11192 | | 11212 | | UUUGGAUUGAAGGGAGCUCUG | | AAGUACUCCCUCCAUUCCAAA | | Translation | | | | 4 | |  |
| sbi-miR159a | SbNFY-C3 | | 5 | | -1 | | 1 | | 21 | | 13153 | | 13173 | | UUUGGAUUGAAGGGAGCUCUG | | GGGAGUGCUUUUGAAUUUAAA | | Cleavage | |  | | 4 | |  |
| sbi-miR159a | SbNFY-C3 | | 5 | | -1 | | 1 | | 21 | | 6075 | | 6095 | | UUUGGAUUGAAGGGAGCUCUG | | CUGAUCUGCCUUCAUUUUGAA | | Cleavage | |  | | 4 | |  |
| sbi-miR159b | SbNFY-C5 | | 5 | | -1 | | 1 | | 21 | | 12440 | | 12460 | | CUUGGAUUGAAGGGAGCUCCU | | ACAAGCUUUCUUCACUUCAAA | | Cleavage | |  | | 1 | |  |
| sbi-miR159b | SbNFY-C3 | | 5 | | -1 | | 1 | | 21 | | 7157 | | 7177 | | CUUGGAUUGAAGGGAGCUCCU | | CCCAGCAACUUUUAAUCCAAA | | Cleavage | |  | | 1 | |  |
| sbi-miR159b | SbNFY-C9 | | 5 | | -1 | | 1 | | 21 | | 5445 | | 5465 | | CUUGGAUUGAAGGGAGCUCCU | | UUGAGUGCCCUUGUAUCCAGG | | Cleavage | |  | | 1 | |  |
| sbi-miR160a | SbNFY-C1 | | 5 | | -1 | | 1 | | 21 | | 11442 | | 11462 | | UGCCUGGCUCCCUGUAUGCCA | | UUAUUUGCAGGGAGCCAGGGA | | Cleavage | |  | | 1 | |  |
| sbi-miR160b | SbNFY-C1 | | 5 | | -1 | | 1 | | 21 | | 11442 | | 11462 | | UGCCUGGCUCCCUGUAUGCCA | | UUAUUUGCAGGGAGCCAGGGA | | Cleavage | |  | | 1 | |  |
| sbi-miR160c | SbNFY-C1 | | 5 | | -1 | | 1 | | 21 | | 11442 | | 11462 | | UGCCUGGCUCCCUGUAUGCCA | | UUAUUUGCAGGGAGCCAGGGA | | Cleavage | |  | | 1 | |  |
| sbi-miR160d | SbNFY-C1 | | 5 | | -1 | | 1 | | 21 | | 11442 | | 11462 | | UGCCUGGCUCCCUGUAUGCCA | | UUAUUUGCAGGGAGCCAGGGA | | Cleavage | |  | | 1 | |  |
| sbi-miR160e | SbNFY-C1 | | 5 | | -1 | | 1 | | 21 | | 11442 | | 11462 | | UGCCUGGCUCCCUGUAUGCCA | | UUAUUUGCAGGGAGCCAGGGA | | Cleavage | |  | | 1 | |  |
| sbi-miR164a | SbNFY-C9 | | 5 | | -1 | | 1 | | 21 | | 17692 | | 17713 | | UGGAGAAGC-AGGGCACGUGCA | | CCCCCGUGCUCUCGCUUUUCCA | | Cleavage | |  | | 1 | |  |
| sbi-miR164a | SbNFY-C13 | | 5 | | -1 | | 1 | | 21 | | 16234 | | 16254 | | UGGAGAAGCAGGGCACGUGCA | | AUCAAGUGGUCUGCUUCAUCA | | Cleavage | |  | | 1 | |  |
| sbi-miR164b | SbNFY-C9 | | 5 | | -1 | | 1 | | 21 | | 17692 | | 17713 | | UGGAGAAGC-AGGGCACGUGCU | | CCCCCGUGCUCUCGCUUUUCCA | | Cleavage | |  | | 1 | |  |
| sbi-miR164b | SbNFY-C13 | | 5 | | -1 | | 1 | | 21 | | 16234 | | 16254 | | UGGAGAAGCAGGGCACGUGCU | | AUCAAGUGGUCUGCUUCAUCA | | Cleavage | |  | | 1 | |  |
| sbi-miR164c | SbNFY-C13 | | 5 | | -1 | | 1 | | 21 | | 16234 | | 16254 | | UGGAGAAGCAGGACACGUGAG | | AUCAAGUGGUCUGCUUCAUCA | | Cleavage | |  | | 1 | |  |
| sbi-miR164c | SbNFY-C15 | | 5 | | -1 | | 1 | | 21 | | 1606 | | 1626 | | UGGAGAAGCAGGACACGUGAG | | UUUCCUUGUCCUUUUUCUCUA | | Cleavage | |  | | 1 | |  |
| sbi-miR164c | SbNFY-C7 | | 5 | | -1 | | 1 | | 21 | | 4788 | | 4808 | | UGGAGAAGCAGGACACGUGAG | | CCCACCCUUCUUGCUUCUCUU | | Cleavage | |  | | 1 | |  |
| sbi-miR164d | SbNFY-C9 | | 5 | | -1 | | 1 | | 21 | | 17692 | | 17713 | | UGGAGAAGC-AGGGCACGUGCA | | CCCCCGUGCUCUCGCUUUUCCA | | Cleavage | |  | | 1 | |  |
| sbi-miR164d | SbNFY-C13 | | 5 | | -1 | | 1 | | 21 | | 16234 | | 16254 | | UGGAGAAGCAGGGCACGUGCA | | AUCAAGUGGUCUGCUUCAUCA | | Cleavage | |  | | 1 | |  |
| sbi-miR164e | SbNFY-C9 | | 5 | | -1 | | 1 | | 21 | | 17692 | | 17713 | | UGGAGAAGC-AGGGCACGUGCA | | CCCCCGUGCUCUCGCUUUUCCA | | Cleavage | |  | | 1 | |  |
| sbi-miR164e | SbNFY-C13 | | 5 | | -1 | | 1 | | 21 | | 16234 | | 16254 | | UGGAGAAGCAGGGCACGUGCA | | AUCAAGUGGUCUGCUUCAUCA | | Cleavage | |  | | 1 | |  |
| sbi-miR166a | SbNFY-C10 | | 5 | | -1 | | 1 | | 20 | | 11394 | | 11413 | | UCGGACCAGGCUUCAUUCCC | | UAGAACUAAGGCUGGUCUGA | | Translation | | | | 1 | |  |
| sbi-miR166b | SbNFY-C10 | | 5 | | -1 | | 1 | | 20 | | 11394 | | 11413 | | UCGGACCAGGCUUCAUUCCC | | UAGAACUAAGGCUGGUCUGA | | Translation | | | | 1 | |  |
| sbi-miR166c | SbNFY-C10 | | 5 | | -1 | | 1 | | 20 | | 11394 | | 11413 | | UCGGACCAGGCUUCAUUCCC | | UAGAACUAAGGCUGGUCUGA | | Translation | | | | 1 | |  |
| sbi-miR166d | SbNFY-C10 | | 5 | | -1 | | 1 | | 20 | | 11394 | | 11413 | | UCGGACCAGGCUUCAUUCCC | | UAGAACUAAGGCUGGUCUGA | | Translation | | | | 1 | |  |
| sbi-miR166f | SbNFY-C10 | | 5 | | -1 | | 1 | | 21 | | 11393 | | 11413 | | UCGGACCAGGCUUCAUUCCUC | | AUAGAACUAAGGCUGGUCUGA | | Translation | | | | 1 | |  |
| sbi-miR166h | SbNFY-C10 | | 5 | | -1 | | 1 | | 20 | | 11394 | | 11413 | | UCGGACCAGGCUUCAUUCCC | | UAGAACUAAGGCUGGUCUGA | | Translation | | | | 1 | |  |
| sbi-miR166i | SbNFY-C10 | | 5 | | -1 | | 1 | | 20 | | 11394 | | 11413 | | UCGGACCAGGCUUCAUUCCC | | UAGAACUAAGGCUGGUCUGA | | Translation | | | | 1 | |  |
| sbi-miR166j | SbNFY-C10 | | 5 | | -1 | | 1 | | 20 | | 11394 | | 11413 | | UCGGACCAGGCUUCAUUCCC | | UAGAACUAAGGCUGGUCUGA | | Translation | | | | 1 | |  |
| sbi-miR166k | SbNFY-C10 | | 5 | | -1 | | 1 | | 20 | | 11394 | | 11413 | | UCGGACCAGGCUUCAUUCCU | | UAGAACUAAGGCUGGUCUGA | | Translation | | | | 1 | |  |
| sbi-miR168 | SbNFY-C11 | | 5 | | -1 | | 1 | | 21 | | 1591 | | 1611 | | UCGCUUGGUGCAGAUCGGGAC | | GUCCUGAUUUGAGCAAAGUGA | | Translation | | | | 1 | |  |
| sbi-miR169c | SbNFY-C10 | | 5 | | -1 | | 1 | | 21 | | 275 | | 295 | | UAGCCAAGGAUGACUUGCCUA | | GGGACAAGUCAUUUGUGGCGA | | Cleavage | |  | | 1 | |  |
| sbi-miR169d-5p | SbNFY-C10 | | 5 | | -1 | | 1 | | 20 | | 276 | | 295 | | UAGCCAAGGAUGACUUGCCU | | GGACAAGUCAUUUGUGGCGA | | Cleavage | |  | | 1 | |  |
| sbi-miR169e | SbNFY-C10 | | 5 | | -1 | | 1 | | 21 | | 275 | | 295 | | UAGCCAAGGAUGACUUGCCGG | | GGGACAAGUCAUUUGUGGCGA | | Cleavage | |  | | 1 | |  |
| sbi-miR169f | SbNFY-C10 | | 5 | | -1 | | 1 | | 21 | | 275 | | 295 | | UAGCCAAGGAUGACUUGCCUG | | GGGACAAGUCAUUUGUGGCGA | | Cleavage | |  | | 1 | |  |
| sbi-miR169g | SbNFY-C10 | | 5 | | -1 | | 1 | | 21 | | 275 | | 295 | | UAGCCAAGGAUGACUUGCCUG | | GGGACAAGUCAUUUGUGGCGA | | Cleavage | |  | | 1 | |  |
| sbi-miR169h | SbNFY-C10 | | 5 | | -1 | | 1 | | 21 | | 275 | | 295 | | UAGCCAAGGAUGACUUGCCUA | | GGGACAAGUCAUUUGUGGCGA | | Cleavage | |  | | 1 | |  |
| sbi-miR169i | SbNFY-C1 | | 5 | | -1 | | 1 | | 21 | | 15933 | | 15953 | | UAGCCAAGAAUGACUUGCCUA | | GCUGUUGGUCGGUCUUGGCUA | | Translation | | | | 1 | |  |
| sbi-miR169j | SbNFY-C10 | | 5 | | -1 | | 1 | | 21 | | 275 | | 295 | | UAGCCAAGGAUGACUUGCCGG | | GGGACAAGUCAUUUGUGGCGA | | Cleavage | |  | | 1 | |  |
| sbi-miR169l | SbNFY-C10 | | 5 | | -1 | | 1 | | 21 | | 275 | | 295 | | UAGCCAAGGAUGACUUGCCUG | | GGGACAAGUCAUUUGUGGCGA | | Cleavage | |  | | 1 | |  |
| sbi-miR169m | SbNFY-C10 | | 5 | | -1 | | 1 | | 21 | | 275 | | 295 | | UAGCCAAGGAUGACUUGCCUA | | GGGACAAGUCAUUUGUGGCGA | | Cleavage | |  | | 1 | |  |
| sbi-miR169n | SbNFY-C10 | | 5 | | -1 | | 1 | | 21 | | 275 | | 295 | | UAGCCAAGGAUGACUUGCCUA | | GGGACAAGUCAUUUGUGGCGA | | Cleavage | |  | | 1 | |  |
| sbi-miR169p | SbNFY-C10 | | 5 | | -1 | | 1 | | 21 | | 275 | | 295 | | UAGCCAAGAAUGGCUUGCCUA | | GGGACAAGUCAUUUGUGGCGA | | Cleavage | |  | | 1 | |  |
| sbi-miR169q | SbNFY-C10 | | 5 | | -1 | | 1 | | 21 | | 275 | | 295 | | UAGCCAAGAAUGGCUUGCCUA | | GGGACAAGUCAUUUGUGGCGA | | Cleavage | |  | | 1 | |  |
| sbi-miR171a | SbNFY-C2 | | 5 | | -1 | | 1 | | 21 | | 11440 | | 11460 | | UGAUUGAGCCGUGCCAAUAUC | | UGUGUUUGCACCACUUAAUCA | | Translation | | | | 1 | |  |
| sbi-miR171a | SbNFY-C11 | | 5 | | -1 | | 1 | | 21 | | 14926 | | 14946 | | UGAUUGAGCCGUGCCAAUAUC | | UUUACAGGUAUGGCUCAGUGA | | Cleavage | |  | | 1 | |  |
| sbi-miR171b | SbNFY-C2 | | 5 | | -1 | | 1 | | 21 | | 11440 | | 11460 | | UGAUUGAGCCGUGCCAAUAUC | | UGUGUUUGCACCACUUAAUCA | | Translation | | | | 1 | |  |
| sbi-miR171b | SbNFY-C11 | | 5 | | -1 | | 1 | | 21 | | 14926 | | 14946 | | UGAUUGAGCCGUGCCAAUAUC | | UUUACAGGUAUGGCUCAGUGA | | Cleavage | |  | | 1 | |  |
| sbi-miR171c | SbNFY-C5 | | 5 | | -1 | | 1 | | 21 | | 15674 | | 15694 | | GAGGUGAGCCGAGCCAAUAUC | | GAUCGUGGCUCGGCACAUUUU | | Cleavage | |  | | 1 | |  |
| sbi-miR171d | SbNFY-C2 | | 5 | | -1 | | 1 | | 21 | | 11440 | | 11460 | | UGAUUGAGCCGUGCCAAUAUC | | UGUGUUUGCACCACUUAAUCA | | Translation | | | | 1 | |  |
| sbi-miR171d | SbNFY-C11 | | 5 | | -1 | | 1 | | 21 | | 14926 | | 14946 | | UGAUUGAGCCGUGCCAAUAUC | | UUUACAGGUAUGGCUCAGUGA | | Cleavage | |  | | 1 | |  |
| sbi-miR171f | SbNFY-C5 | | 5 | | -1 | | 1 | | 21 | | 12210 | | 12229 | | AUGAGCCGAACCAAUAUCACU | | ACUGA-AUUGGUUGGGAUCAU | | Cleavage | |  | | 1 | |  |
| sbi-miR171h | SbNFY-C3 | | 5 | | -1 | | 1 | | 21 | | 4158 | | 4178 | | GGAUUGAGCCGCGUCAAUAUC | | UUUGUAGAUGUGGCUCAUUUU | | Cleavage | |  | | 1 | |  |
| sbi-miR171i | SbNFY-C2 | | 5 | | -1 | | 1 | | 21 | | 11440 | | 11460 | | UGAUUGAGCCGUGCCAAUAUC | | UGUGUUUGCACCACUUAAUCA | | Translation | | | | 1 | |  |
| sbi-miR171i | SbNFY-C11 | | 5 | | -1 | | 1 | | 21 | | 14926 | | 14946 | | UGAUUGAGCCGUGCCAAUAUC | | UUUACAGGUAUGGCUCAGUGA | | Cleavage | |  | | 1 | |  |
| sbi-miR171k | SbNFY-C2 | | 5 | | -1 | | 1 | | 21 | | 11440 | | 11460 | | UGAUUGAGCCGUGCCAAUAUC | | UGUGUUUGCACCACUUAAUCA | | Translation | | | | 1 | |  |
| sbi-miR171k | SbNFY-C11 | | 5 | | -1 | | 1 | | 21 | | 14926 | | 14946 | | UGAUUGAGCCGUGCCAAUAUC | | UUUACAGGUAUGGCUCAGUGA | | Cleavage | |  | | 1 | |  |
| sbi-miR172a | SbNFY-C1 | | 5 | | -1 | | 1 | | 20 | | 7670 | | 7689 | | AGAAUCUUGAUGAUGCUGCA | | GGUGGCAUCACCGACGUUCU | | Translation | | | | 1 | |  |
| sbi-miR172c | SbNFY-C1 | | 5 | | -1 | | 1 | | 20 | | 7670 | | 7689 | | AGAAUCUUGAUGAUGCUGCA | | GGUGGCAUCACCGACGUUCU | | Translation | | | | 1 | |  |
| sbi-miR172d | SbNFY-C1 | | 5 | | -1 | | 1 | | 20 | | 7670 | | 7689 | | AGAAUCUUGAUGAUGCUGCA | | GGUGGCAUCACCGACGUUCU | | Translation | | | | 1 | |  |
| sbi-miR172e | SbNFY-C1 | | 5 | | -1 | | 1 | | 21 | | 12737 | | 12757 | | UGAAUCUUGAUGAUGCUGCAC | | UUAGAUCAUUAUUAGGAUUUA | | Cleavage | |  | | 2 | |  |
| sbi-miR172e | SbNFY-C1 | | 5 | | -1 | | 1 | | 21 | | 6930 | | 6949 | | UGAAUCUUGAUGAUGCUGCAC | | UGGAAGC-UCAUCAAAAUUUA | | Cleavage | |  | | 2 | |  |
| sbi-miR172e | SbNFY-C4 | | 5 | | -1 | | 1 | | 21 | | 15342 | | 15362 | | UGAAUCUUGAUGAUGCUGCAC | | AUGCAUGGUCAUCAUGGUUUA | | Cleavage | |  | | 1 | |  |
| sbi-miR2118-5p | SbNFY-C14 | | 5 | | -1 | | 1 | | 22 | | 4527 | | 4548 | | GGCAUGGGAACAUGUAGGAAGG | | UUUUACUAUAUAUUCUCAUGGC | | Translation | | | | 1 | |  |
| sbi-miR2118-5p | SbNFY-C5 | | 5 | | -1 | | 1 | | 22 | | 11491 | | 11512 | | GGCAUGGGAACAUGUAGGAAGG | | GAGUACUGUAUGUUUUCAGGCU | | Cleavage | |  | | 1 | |  |
| sbi-miR2118-5p | SbNFY-C11 | | 5 | | -1 | | 1 | | 22 | | 4720 | | 4741 | | GGCAUGGGAACAUGUAGGAAGG | | AGUUCUUGCCUUUUCUCAUGUC | | Translation | | | | 1 | |  |
| sbi-miR390 | SbNFY-C5 | | 5 | | -1 | | 1 | | 21 | | 16377 | | 16397 | | AAGCUCAGGAGGGAUAGCGCC | | UGCAGUAUUUCUUCUGAGAUU | | Cleavage | |  | | 1 | |  |
| sbi-miR390 | SbNFY-C15 | | 5 | | -1 | | 1 | | 21 | | 8383 | | 8403 | | AAGCUCAGGAGGGAUAGCGCC | | GACCUUCUUCAUCCUGGGCUU | | Translation | | | | 1 | |  |
| sbi-miR393a | SbNFY-C3 | | 5 | | -1 | | 1 | | 21 | | 10878 | | 10898 | | UCCAAAGGGAUCGCAUUGAUC | | UCUCAAUUUGAGCUUUUUGGC | | Translation | | | | 1 | |  |
| sbi-miR393b | SbNFY-C3 | | 5 | | -1 | | 1 | | 21 | | 10878 | | 10898 | | UCCAAAGGGAUCGCAUUGAUC | | UCUCAAUUUGAGCUUUUUGGC | | Translation | | | | 1 | |  |
| sbi-miR394a | SbNFY-C10 | | 5 | | -1 | | 1 | | 20 | | 13573 | | 13592 | | UUGGCAUUCUGUCCACCUCC | | CCUGGUGGACAUAAUCCCAA | | Cleavage | |  | | 1 | |  |
| sbi-miR394b | SbNFY-C10 | | 5 | | -1 | | 1 | | 20 | | 13573 | | 13592 | | UUGGCAUUCUGUCCACCUCC | | CCUGGUGGACAUAAUCCCAA | | Cleavage | |  | | 1 | |  |
| sbi-miR395a | SbNFY-C2 | | 5 | | -1 | | 1 | | 21 | | 7926 | | 7946 | | GUGAAGUGUUUGGGGGAACUC | | AGGCUUCCCUGAGCUCUUCAU | | Cleavage | |  | | 2 | |  |
| sbi-miR395a | SbNFY-C13 | | 5 | | -1 | | 1 | | 21 | | 6269 | | 6289 | | GUGAAGUGUUUGGGGGAACUC | | CAGUUUCCCCGUGCGCGUCAC | | Translation | | | | 1 | |  |
| sbi-miR395b | SbNFY-C2 | | 5 | | -1 | | 1 | | 21 | | 7926 | | 7946 | | GUGAAGUGUUUGGGGGAACUC | | AGGCUUCCCUGAGCUCUUCAU | | Cleavage | |  | | 2 | |  |
| sbi-miR395b | SbNFY-C13 | | 5 | | -1 | | 1 | | 21 | | 6269 | | 6289 | | GUGAAGUGUUUGGGGGAACUC | | CAGUUUCCCCGUGCGCGUCAC | | Translation | | | | 1 | |  |
| sbi-miR395c | SbNFY-C2 | | 5 | | -1 | | 1 | | 21 | | 7926 | | 7946 | | GUGAAGUGUUUGGGGGAACUC | | AGGCUUCCCUGAGCUCUUCAU | | Cleavage | |  | | 2 | |  |
| sbi-miR395c | SbNFY-C13 | | 5 | | -1 | | 1 | | 21 | | 6269 | | 6289 | | GUGAAGUGUUUGGGGGAACUC | | CAGUUUCCCCGUGCGCGUCAC | | Translation | | | | 1 | |  |
| sbi-miR395d | SbNFY-C2 | | 5 | | -1 | | 1 | | 21 | | 7926 | | 7946 | | GUGAAGUGUUUGGGGGAACUC | | AGGCUUCCCUGAGCUCUUCAU | | Cleavage | |  | | 2 | |  |
| sbi-miR395d | SbNFY-C13 | | 5 | | -1 | | 1 | | 21 | | 6269 | | 6289 | | GUGAAGUGUUUGGGGGAACUC | | CAGUUUCCCCGUGCGCGUCAC | | Translation | | | | 1 | |  |
| sbi-miR395e | SbNFY-C2 | | 5 | | -1 | | 1 | | 21 | | 7926 | | 7946 | | GUGAAGUGUUUGGGGGAACUC | | AGGCUUCCCUGAGCUCUUCAU | | Cleavage | |  | | 2 | |  |
| sbi-miR395e | SbNFY-C13 | | 5 | | -1 | | 1 | | 21 | | 6269 | | 6289 | | GUGAAGUGUUUGGGGGAACUC | | CAGUUUCCCCGUGCGCGUCAC | | Translation | | | | 1 | |  |
| sbi-miR395f | SbNFY-C7 | | 5 | | -1 | | 1 | | 21 | | 14165 | | 14185 | | AUGAAGUGUUUGGGGGAACUC | | UAGUUCCACUAAAUUCUCCAU | | Cleavage | |  | | 1 | |  |
| sbi-miR395f | SbNFY-C13 | | 5 | | -1 | | 1 | | 21 | | 17662 | | 17682 | | AUGAAGUGUUUGGGGGAACUC | | GUGUCACAUCAAAUGUUUCAU | | Cleavage | |  | | 1 | |  |
| sbi-miR395f | SbNFY-C11 | | 5 | | -1 | | 1 | | 21 | | 14059 | | 14079 | | AUGAAGUGUUUGGGGGAACUC | | UUGUGACUUCAGACACUUCCU | | Cleavage | |  | | 1 | |  |
| sbi-miR395g | SbNFY-C2 | | 5 | | -1 | | 1 | | 21 | | 7926 | | 7946 | | GUGAAGUGUUUGGGGGAACUC | | AGGCUUCCCUGAGCUCUUCAU | | Cleavage | |  | | 2 | |  |
| sbi-miR395g | SbNFY-C13 | | 5 | | -1 | | 1 | | 21 | | 6269 | | 6289 | | GUGAAGUGUUUGGGGGAACUC | | CAGUUUCCCCGUGCGCGUCAC | | Translation | | | | 1 | |  |
| sbi-miR395h | SbNFY-C2 | | 5 | | -1 | | 1 | | 21 | | 7926 | | 7946 | | GUGAAGUGUUUGGGGGAACUC | | AGGCUUCCCUGAGCUCUUCAU | | Cleavage | |  | | 2 | |  |
| sbi-miR395h | SbNFY-C13 | | 5 | | -1 | | 1 | | 21 | | 6269 | | 6289 | | GUGAAGUGUUUGGGGGAACUC | | CAGUUUCCCCGUGCGCGUCAC | | Translation | | | | 1 | |  |
| sbi-miR395i | SbNFY-C2 | | 5 | | -1 | | 1 | | 21 | | 7926 | | 7946 | | GUGAAGUGUUUGGGGGAACUC | | AGGCUUCCCUGAGCUCUUCAU | | Cleavage | |  | | 2 | |  |
| sbi-miR395i | SbNFY-C13 | | 5 | | -1 | | 1 | | 21 | | 6269 | | 6289 | | GUGAAGUGUUUGGGGGAACUC | | CAGUUUCCCCGUGCGCGUCAC | | Translation | | | | 1 | |  |
| sbi-miR395j | SbNFY-C2 | | 5 | | -1 | | 1 | | 21 | | 7926 | | 7946 | | GUGAAGUGUUUGGGGGAACUC | | AGGCUUCCCUGAGCUCUUCAU | | Cleavage | |  | | 2 | |  |
| sbi-miR395j | SbNFY-C13 | | 5 | | -1 | | 1 | | 21 | | 6269 | | 6289 | | GUGAAGUGUUUGGGGGAACUC | | CAGUUUCCCCGUGCGCGUCAC | | Translation | | | | 1 | |  |
| sbi-miR395k | SbNFY-C4 | | 5 | | -1 | | 1 | | 21 | | 4116 | | 4137 | | GUGAAGUG-UUUGGAGGAACUC | | AAAUUUCUCCAAAACAUUUCAC | | Cleavage | |  | | 1 | |  |
| sbi-miR395k | SbNFY-C9 | | 5 | | -1 | | 1 | | 21 | | 12665 | | 12685 | | GUGAAGUGUUUGGAGGAACUC | | CAUCGCCUCCGGACGCUUCGC | | Cleavage | |  | | 1 | |  |
| sbi-miR395k | SbNFY-C12 | | 5 | | -1 | | 1 | | 21 | | 11830 | | 11850 | | GUGAAGUGUUUGGAGGAACUC | | GUGGUCCUCCUAAUAUUUUAG | | Translation | | | | 1 | |  |
| sbi-miR395k | SbNFY-C13 | | 5 | | -1 | | 1 | | 21 | | 13846 | | 13865 | | GUGAAGUGUUUGGAGGAACUC | | AUGUUUC-CUAAAUACAUCAC | | Cleavage | |  | | 1 | |  |
| sbi-miR395l | SbNFY-C4 | | 5 | | -1 | | 1 | | 21 | | 4117 | | 4137 | | GUGAAGUGCUUGGGGGAACUC | | AAUUUCUCCAAAACAUUUCAC | | Cleavage | |  | | 1 | |  |
| sbi-miR396a | SbNFY-C13 | | 5 | | -1 | | 1 | | 21 | | 16469 | | 16489 | | UUCCACAGCUUUCUUGAACUG | | CCGUUGGAGGGAGUUGAGGGA | | Cleavage | |  | | 2 | |  |
| sbi-miR396b | SbNFY-C13 | | 5 | | -1 | | 1 | | 21 | | 16469 | | 16489 | | UUCCACAGCUUUCUUGAACUG | | CCGUUGGAGGGAGUUGAGGGA | | Cleavage | |  | | 2 | |  |
| sbi-miR396c | SbNFY-C13 | | 5 | | -1 | | 1 | | 21 | | 16469 | | 16489 | | UUCCACAGCUUUCUUGAACUU | | CCGUUGGAGGGAGUUGAGGGA | | Cleavage | |  | | 2 | |  |
| sbi-miR396e | SbNFY-C15 | | 5 | | -1 | | 1 | | 22 | | 5129 | | 5150 | | UUCCACAGGCUUUCUUGAACUG | | GGAUUGAAUGAAUUUUGUGGAA | | Translation | | | | 1 | |  |
| sbi-miR397-5p | SbNFY-C2 | | 5 | | -1 | | 1 | | 21 | | 5113 | | 5133 | | UCAUUGAGUGCAGCGUUGAUG | | UCGCAAUCCUGUACACAAUGG | | Cleavage | |  | | 1 | |  |
| sbi-miR398 | SbNFY-C6 | | 5 | | -1 | | 1 | | 21 | | 6583 | | 6603 | | UGUGUUCUCAGGUCGCCCCCG | | AUCGGACGACCUGAAAACAUU | | Cleavage | |  | | 1 | |  |
| sbi-miR399a | SbNFY-C5 | | 5 | | -1 | | 1 | | 21 | | 9446 | | 9466 | | UGCCAAAGGAGAAUUGCCCUG | | UUUGGCUCUUCUCCUUUGUUA | | Cleavage | |  | | 1 | |  |
| sbi-miR399c | SbNFY-C5 | | 5 | | -1 | | 1 | | 21 | | 9446 | | 9466 | | UGCCAAAGGAGAAUUGCCCUG | | UUUGGCUCUUCUCCUUUGUUA | | Cleavage | |  | | 1 | |  |
| sbi-miR399e | SbNFY-C12 | | 5 | | -1 | | 1 | | 21 | | 7149 | | 7169 | | UGCCAAAGGAGAUUUGCCCAG | | AAAGGUAUAGCUUCUUUGGUA | | Cleavage | |  | | 1 | |  |
| sbi-miR399f | SbNFY-C12 | | 5 | | -1 | | 1 | | 21 | | 7149 | | 7169 | | UGCCAAAGGAGAUUUGCCCAG | | AAAGGUAUAGCUUCUUUGGUA | | Cleavage | |  | | 1 | |  |
| sbi-miR399g | SbNFY-C5 | | 5 | | -1 | | 1 | | 21 | | 15988 | | 16007 | | UGCCAAAGGAAAUUUGCCCCG | | CAUGACAAAUUUCCUU-GGCA | | Cleavage | |  | | 1 | |  |
| sbi-miR399g | SbNFY-C12 | | 5 | | -1 | | 1 | | 21 | | 3065 | | 3085 | | UGCCAAAGGAAAUUUGCCCCG | | CAGAGAAGGUUUGCUUUGGCG | | Cleavage | |  | | 1 | |  |
| sbi-miR399h | SbNFY-C5 | | 5 | | -1 | | 1 | | 21 | | 9446 | | 9466 | | UGCCAAAGGAGAAUUGCCCUG | | UUUGGCUCUUCUCCUUUGUUA | | Cleavage | |  | | 1 | |  |
| sbi-miR399j | SbNFY-C5 | | 5 | | -1 | | 1 | | 21 | | 9446 | | 9466 | | UGCCAAAGGAGAAUUGCCCUG | | UUUGGCUCUUCUCCUUUGUUA | | Cleavage | |  | | 1 | |  |
| sbi-miR408 | SbNFY-C9 | | 5 | | -1 | | 1 | | 21 | | 12078 | | 12098 | | CUGCACUGCCUCUUCCCUGGC | | GACGGCGAGGAGGCAGAGCGU | | Cleavage | |  | | 2 | |  |
| sbi-miR437a | SbNFY-C14 | | 5 | | -1 | | 1 | | 21 | | 16373 | | 16393 | | AAAGUUAGAGAAGUUUGACUU | | AAUUCAGAUUUCUCGAAUUUC | | Cleavage | |  | | 1 | |  |
| sbi-miR437a | SbNFY-C13 | | 5 | | -1 | | 1 | | 21 | | 8423 | | 8443 | | AAAGUUAGAGAAGUUUGACUU | | UUGACAAAUUUAUCUCAUUUU | | Translation | | | | 1 | |  |
| sbi-miR437b | SbNFY-C14 | | 5 | | -1 | | 1 | | 21 | | 16373 | | 16393 | | AAAGUUAGAGAAGUUUGACUU | | AAUUCAGAUUUCUCGAAUUUC | | Cleavage | |  | | 1 | |  |
| sbi-miR437b | SbNFY-C13 | | 5 | | -1 | | 1 | | 21 | | 8423 | | 8443 | | AAAGUUAGAGAAGUUUGACUU | | UUGACAAAUUUAUCUCAUUUU | | Translation | | | | 1 | |  |
| sbi-miR437c | SbNFY-C14 | | 5 | | -1 | | 1 | | 21 | | 16373 | | 16393 | | AAAGUUAGAGAAGUUUGACUU | | AAUUCAGAUUUCUCGAAUUUC | | Cleavage | |  | | 1 | |  |
| sbi-miR437c | SbNFY-C13 | | 5 | | -1 | | 1 | | 21 | | 8423 | | 8443 | | AAAGUUAGAGAAGUUUGACUU | | UUGACAAAUUUAUCUCAUUUU | | Translation | | | | 1 | |  |
| sbi-miR437d | SbNFY-C14 | | 5 | | -1 | | 1 | | 21 | | 16373 | | 16393 | | AAAGUUAGAGAAGUUUGACUU | | AAUUCAGAUUUCUCGAAUUUC | | Cleavage | |  | | 1 | |  |
| sbi-miR437d | SbNFY-C13 | | 5 | | -1 | | 1 | | 21 | | 8423 | | 8443 | | AAAGUUAGAGAAGUUUGACUU | | UUGACAAAUUUAUCUCAUUUU | | Translation | | | | 1 | |  |
| sbi-miR437e | SbNFY-C14 | | 5 | | -1 | | 1 | | 21 | | 16373 | | 16393 | | AAAGUUAGAGAAGUUUGACUU | | AAUUCAGAUUUCUCGAAUUUC | | Cleavage | |  | | 1 | |  |
| sbi-miR437e | SbNFY-C13 | | 5 | | -1 | | 1 | | 21 | | 8423 | | 8443 | | AAAGUUAGAGAAGUUUGACUU | | UUGACAAAUUUAUCUCAUUUU | | Translation | | | | 1 | |  |
| sbi-miR437f | SbNFY-C14 | | 5 | | -1 | | 1 | | 21 | | 16373 | | 16393 | | AAAGUUAGAGAAGUUUGACUU | | AAUUCAGAUUUCUCGAAUUUC | | Cleavage | |  | | 1 | |  |
| sbi-miR437f | SbNFY-C13 | | 5 | | -1 | | 1 | | 21 | | 8423 | | 8443 | | AAAGUUAGAGAAGUUUGACUU | | UUGACAAAUUUAUCUCAUUUU | | Translation | | | | 1 | |  |
| sbi-miR437g | SbNFY-C14 | | 5 | | -1 | | 1 | | 21 | | 16373 | | 16393 | | AAAGUUAGAGAAGUUUGACUU | | AAUUCAGAUUUCUCGAAUUUC | | Cleavage | |  | | 1 | |  |
| sbi-miR437g | SbNFY-C13 | | 5 | | -1 | | 1 | | 21 | | 8423 | | 8443 | | AAAGUUAGAGAAGUUUGACUU | | UUGACAAAUUUAUCUCAUUUU | | Translation | | | | 1 | |  |
| sbi-miR437i | SbNFY-C14 | | 5 | | -1 | | 1 | | 21 | | 16373 | | 16393 | | AAAGUUAGAGAAGUUUGACUU | | AAUUCAGAUUUCUCGAAUUUC | | Cleavage | |  | | 1 | |  |
| sbi-miR437i | SbNFY-C13 | | 5 | | -1 | | 1 | | 21 | | 8423 | | 8443 | | AAAGUUAGAGAAGUUUGACUU | | UUGACAAAUUUAUCUCAUUUU | | Translation | | | | 1 | |  |
| sbi-miR437j | SbNFY-C14 | | 5 | | -1 | | 1 | | 21 | | 16373 | | 16393 | | AAAGUUAGAGAAGUUUGACUU | | AAUUCAGAUUUCUCGAAUUUC | | Cleavage | |  | | 1 | |  |
| sbi-miR437j | SbNFY-C13 | | 5 | | -1 | | 1 | | 21 | | 8423 | | 8443 | | AAAGUUAGAGAAGUUUGACUU | | UUGACAAAUUUAUCUCAUUUU | | Translation | | | | 1 | |  |
| sbi-miR437k | SbNFY-C14 | | 5 | | -1 | | 1 | | 21 | | 16373 | | 16393 | | AAAGUUAGAGAAGUUUGACUU | | AAUUCAGAUUUCUCGAAUUUC | | Cleavage | |  | | 1 | |  |
| sbi-miR437k | SbNFY-C13 | | 5 | | -1 | | 1 | | 21 | | 8423 | | 8443 | | AAAGUUAGAGAAGUUUGACUU | | UUGACAAAUUUAUCUCAUUUU | | Translation | | | | 1 | |  |
| sbi-miR437l | SbNFY-C14 | | 5 | | -1 | | 1 | | 21 | | 16373 | | 16393 | | AAAGUUAGAGAAGUUUGACUU | | AAUUCAGAUUUCUCGAAUUUC | | Cleavage | |  | | 1 | |  |
| sbi-miR437l | SbNFY-C13 | | 5 | | -1 | | 1 | | 21 | | 8423 | | 8443 | | AAAGUUAGAGAAGUUUGACUU | | UUGACAAAUUUAUCUCAUUUU | | Translation | | | | 1 | |  |
| sbi-miR437m | SbNFY-C14 | | 5 | | -1 | | 1 | | 21 | | 16373 | | 16393 | | AAAGUUAGAGAAGUUUGACUU | | AAUUCAGAUUUCUCGAAUUUC | | Cleavage | |  | | 1 | |  |
| sbi-miR437m | SbNFY-C13 | | 5 | | -1 | | 1 | | 21 | | 8423 | | 8443 | | AAAGUUAGAGAAGUUUGACUU | | UUGACAAAUUUAUCUCAUUUU | | Translation | | | | 1 | |  |
| sbi-miR437n | SbNFY-C14 | | 5 | | -1 | | 1 | | 21 | | 16373 | | 16393 | | AAAGUUAGAGAAGUUUGACUU | | AAUUCAGAUUUCUCGAAUUUC | | Cleavage | |  | | 1 | |  |
| sbi-miR437n | SbNFY-C13 | | 5 | | -1 | | 1 | | 21 | | 8423 | | 8443 | | AAAGUUAGAGAAGUUUGACUU | | UUGACAAAUUUAUCUCAUUUU | | Translation | | | | 1 | |  |
| sbi-miR437o | SbNFY-C14 | | 5 | | -1 | | 1 | | 21 | | 16373 | | 16393 | | AAAGUUAGAGAAGUUUGACUU | | AAUUCAGAUUUCUCGAAUUUC | | Cleavage | |  | | 1 | |  |
| sbi-miR437o | SbNFY-C13 | | 5 | | -1 | | 1 | | 21 | | 8423 | | 8443 | | AAAGUUAGAGAAGUUUGACUU | | UUGACAAAUUUAUCUCAUUUU | | Translation | | | | 1 | |  |
| sbi-miR437p | SbNFY-C14 | | 5 | | -1 | | 1 | | 21 | | 16373 | | 16393 | | AAAGUUAGAGAAGUUUGACUU | | AAUUCAGAUUUCUCGAAUUUC | | Cleavage | |  | | 1 | |  |
| sbi-miR437p | SbNFY-C13 | | 5 | | -1 | | 1 | | 21 | | 8423 | | 8443 | | AAAGUUAGAGAAGUUUGACUU | | UUGACAAAUUUAUCUCAUUUU | | Translation | | | | 1 | |  |
| sbi-miR437q | SbNFY-C14 | | 5 | | -1 | | 1 | | 21 | | 16373 | | 16393 | | AAAGUUAGAGAAGUUUGACUU | | AAUUCAGAUUUCUCGAAUUUC | | Cleavage | |  | | 1 | |  |
| sbi-miR437q | SbNFY-C13 | | 5 | | -1 | | 1 | | 21 | | 8423 | | 8443 | | AAAGUUAGAGAAGUUUGACUU | | UUGACAAAUUUAUCUCAUUUU | | Translation | | | | 1 | |  |
| sbi-miR437r | SbNFY-C14 | | 5 | | -1 | | 1 | | 21 | | 16373 | | 16393 | | AAAGUUAGAGAAGUUUGACUU | | AAUUCAGAUUUCUCGAAUUUC | | Cleavage | |  | | 1 | |  |
| sbi-miR437r | SbNFY-C13 | | 5 | | -1 | | 1 | | 21 | | 8423 | | 8443 | | AAAGUUAGAGAAGUUUGACUU | | UUGACAAAUUUAUCUCAUUUU | | Translation | | | | 1 | |  |
| sbi-miR437s | SbNFY-C14 | | 5 | | -1 | | 1 | | 21 | | 16373 | | 16393 | | AAAGUUAGAGAAGUUUGACUU | | AAUUCAGAUUUCUCGAAUUUC | | Cleavage | |  | | 1 | |  |
| sbi-miR437s | SbNFY-C13 | | 5 | | -1 | | 1 | | 21 | | 8423 | | 8443 | | AAAGUUAGAGAAGUUUGACUU | | UUGACAAAUUUAUCUCAUUUU | | Translation | | | | 1 | |  |
| sbi-miR437t | SbNFY-C14 | | 5 | | -1 | | 1 | | 21 | | 16373 | | 16393 | | AAAGUUAGAGAAGUUUGACUU | | AAUUCAGAUUUCUCGAAUUUC | | Cleavage | |  | | 1 | |  |
| sbi-miR437t | SbNFY-C13 | | 5 | | -1 | | 1 | | 21 | | 8423 | | 8443 | | AAAGUUAGAGAAGUUUGACUU | | UUGACAAAUUUAUCUCAUUUU | | Translation | | | | 1 | |  |
| sbi-miR437u | SbNFY-C14 | | 5 | | -1 | | 1 | | 21 | | 16373 | | 16393 | | AAAGUUAGAGAAGUUUGACUU | | AAUUCAGAUUUCUCGAAUUUC | | Cleavage | |  | | 1 | |  |
| sbi-miR437u | SbNFY-C13 | | 5 | | -1 | | 1 | | 21 | | 8423 | | 8443 | | AAAGUUAGAGAAGUUUGACUU | | UUGACAAAUUUAUCUCAUUUU | | Translation | | | | 1 | |  |
| sbi-miR437v | SbNFY-C14 | | 5 | | -1 | | 1 | | 21 | | 16373 | | 16393 | | AAAGUUAGAGAAGUUUGACUU | | AAUUCAGAUUUCUCGAAUUUC | | Cleavage | |  | | 1 | |  |
| sbi-miR437v | SbNFY-C13 | | 5 | | -1 | | 1 | | 21 | | 8423 | | 8443 | | AAAGUUAGAGAAGUUUGACUU | | UUGACAAAUUUAUCUCAUUUU | | Translation | | | | 1 | |  |
| sbi-miR437w | SbNFY-C14 | | 5 | | -1 | | 1 | | 21 | | 16373 | | 16393 | | AAAGUUAGAGAAGUUUGACUU | | AAUUCAGAUUUCUCGAAUUUC | | Cleavage | |  | | 1 | |  |
| sbi-miR437w | SbNFY-C13 | | 5 | | -1 | | 1 | | 21 | | 8423 | | 8443 | | AAAGUUAGAGAAGUUUGACUU | | UUGACAAAUUUAUCUCAUUUU | | Translation | | | | 1 | |  |
| sbi-miR437x-3p | SbNFY-C2 | | 5 | | -1 | | 1 | | 24 | | 7999 | | 8022 | | AUUUGACUGACACGGAUUCUAGGA | | UUGAAGUAUUCGUGAUAGGCAAAU | | Translation | | | | 1 | |  |
| sbi-miR528 | SbNFY-C9 | | 5 | | -1 | | 1 | | 21 | | 13599 | | 13619 | | UGGAAGGGGCAUGCAGAGGAG | | AAUUUCUCCAUGCCUCUCCCC | | Cleavage | |  | | 1 | |  |
| sbi-miR528 | SbNFY-C2 | | 5 | | -1 | | 1 | | 21 | | 17151 | | 17171 | | UGGAAGGGGCAUGCAGAGGAG | | GGCCUCAGUUUGCAUCUUCCA | | Cleavage | |  | | 2 | |  |
| sbi-miR528 | SbNFY-C2 | | 5 | | -1 | | 1 | | 21 | | 14393 | | 14413 | | UGGAAGGGGCAUGCAGAGGAG | | UACCUACGUGUGCACCUUUCA | | Cleavage | |  | | 2 | |  |
| sbi-miR529 | SbNFY-C11 | | 5 | | -1 | | 1 | | 20 | | 11768 | | 11787 | | CUGUACCCUCUCUCUUCUUC | | UUGGAAAGGAGGGAGUACAG | | Cleavage | |  | | 2 | |  |
| sbi-miR529 | SbNFY-C9 | | 5 | | -1 | | 1 | | 20 | | 2521 | | 2540 | | CUGUACCCUCUCUCUUCUUC | | AAAGAAAAGAGGGGAGAUAG | | Cleavage | |  | | 1 | |  |
| sbi-miR5381 | SbNFY-C10 | | 5 | | -1 | | 1 | | 19 | | 9971 | | 9989 | | AAGAUCUGUGGCGCCGAGC | | UAUCGAUGCUACAGAUCUC | | Cleavage | |  | | 1 | |  |
| sbi-miR5381 | SbNFY-C2 | | 5 | | -1 | | 1 | | 19 | | 15404 | | 15422 | | AAGAUCUGUGGCGCCGAGC | | GCUUGUGGCUACUGAUCUU | | Cleavage | |  | | 1 | |  |
| sbi-miR5385 | SbNFY-C4 | | 5 | | -1 | | 1 | | 22 | | 17608 | | 17629 | | ACCACCAACCCCACCGCUUCUC | | CAAAAGCCGUGGGCUAGGUGGC | | Cleavage | |  | | 1 | |  |
| sbi-miR5386 | SbNFY-C3 | | 5 | | -1 | | 1 | | 20 | | 7353 | | 7372 | | CGUCGCUGUCGCGCGCGCUG | | CGGAGCGCGCGUCGGCGUCG | | Cleavage | |  | | 1 | |  |
| sbi-miR5386 | SbNFY-C14 | | 5 | | -1 | | 1 | | 20 | | 17225 | | 17244 | | CGUCGCUGUCGCGCGCGCUG | | CGGCGGCGGCGGCGGCGACG | | Cleavage | |  | | 2 | |  |
| sbi-miR5386 | SbNFY-C12 | | 5 | | -1 | | 1 | | 20 | | 982 | | 1001 | | CGUCGCUGUCGCGCGCGCUG | | CAGCGGCGGCGGCGGCGGCG | | Cleavage | |  | | 1 | |  |
| sbi-miR5387b | SbNFY-C6 | | 5 | | -1 | | 1 | | 24 | | 9246 | | 9269 | | CGUGGCUCUGACCGGUGCUAAAGG | | CAAUGAGGACUAGUCAGGGCCAUU | | Cleavage | |  | | 1 | |  |
| sbi-miR5388 | SbNFY-C1 | | 5 | | -1 | | 1 | | 22 | | 11588 | | 11609 | | AUCUUUGCCGGGUGUCUCUGAC | | UUUACAUAUACCCGGAGAGGAU | | Cleavage | |  | | 1 | |  |
| sbi-miR5389 | SbNFY-C7 | | 5 | | -1 | | 1 | | 21 | | 17104 | | 17124 | | GCUUGAGUUUAUCAGCCGAGU | | CAUUGGCGGAUACAUUCAACC | | Cleavage | |  | | 1 | |  |
| sbi-miR5564a | SbNFY-C8 | | 5 | | -1 | | 1 | | 22 | | 6345 | | 6366 | | UGGGGAAGCAAUUCGUCGAACA | | AUGUGGGCCAACUGUUUCUCCA | | Translation | | | | 1 | |  |
| sbi-miR5564b | SbNFY-C14 | | 5 | | -1 | | 1 | | 21 | | 12271 | | 12291 | | GCAAUUCGUCGAACAGCUUGA | | UAAGGUCGUUCGACUAGUUGA | | Cleavage | |  | | 2 | |  |
| sbi-miR5564b | SbNFY-C14 | | 5 | | -1 | | 1 | | 21 | | 2954 | | 2974 | | GCAAUUCGUCGAACAGCUUGA | | CUAGACGGUUUGGUGGAUUGU | | Cleavage | |  | | 2 | |  |
| sbi-miR5564b | SbNFY-C6 | | 5 | | -1 | | 1 | | 21 | | 7368 | | 7388 | | GCAAUUCGUCGAACAGCUUGA | | UGGAUCUCUUUGAUGAAUUUC | | Cleavage | |  | | 1 | |  |
| sbi-miR5564c-3p | SbNFY-C11 | | 5 | | -1 | | 1 | | 21 | | 3295 | | 3315 | | ACGCGAGCUGUUUGGCGAAUU | | AAUUUGCCAAACUGUUCGAGA | | Cleavage | |  | | 1 | |  |
| sbi-miR5564c-3p | SbNFY-C8 | | 5 | | -1 | | 1 | | 21 | | 1063 | | 1083 | | ACGCGAGCUGUUUGGCGAAUU | | CAUUCUUCGAACAACUCGCUU | | Cleavage | |  | | 1 | |  |
| sbi-miR5564c-5p | SbNFY-C4 | | 5 | | -1 | | 1 | | 21 | | 12729 | | 12749 | | AAUUCGUCGAACAGCUGCAGC | | GCUUCAGUUUUGUGACGAAUU | | Translation | | | | 1 | |  |
| sbi-miR5564c-5p | SbNFY-C7 | | 5 | | -1 | | 1 | | 21 | | 7788 | | 7808 | | AAUUCGUCGAACAGCUGCAGC | | GCUCGAGCUGUUCGCCGAAAU | | Cleavage | |  | | 2 | |  |
| sbi-miR5565a | SbNFY-C6 | | 5 | | -1 | | 1 | | 24 | | 8424 | | 8447 | | AACACAUGUGGAUUGAGGCGAAUC | | UGAUAGUUUCAAUGCAUGUGUCUU | | Translation | | | | 1 | |  |
| sbi-miR5565b | SbNFY-C6 | | 5 | | -1 | | 1 | | 24 | | 8424 | | 8447 | | AACACAUGUGGAUUGAGGCGAAUC | | UGAUAGUUUCAAUGCAUGUGUCUU | | Translation | | | | 1 | |  |
| sbi-miR5565d | SbNFY-C9 | | 5 | | -1 | | 1 | | 24 | | 15694 | | 15717 | | ACUUCAAUCCAUGUAUGUUGGUGU | | AAAAAAAAAUACAUGGUUUGAAAA | | Cleavage | |  | | 1 | |  |
| sbi-miR5565e | SbNFY-C5 | | 5 | | -1 | | 1 | | 19 | | 4324 | | 4342 | | UUGUUUGGAUGUUGUCGGA | | UUCAACAGUGUUCACACAA | | Cleavage | |  | | 2 | |  |
| sbi-miR5565e | SbNFY-C5 | | 5 | | -1 | | 1 | | 19 | | 7416 | | 7434 | | UUGUUUGGAUGUUGUCGGA | | UGCUACAAUGUCAAAAUAA | | Cleavage | |  | | 2 | |  |
| sbi-miR5565e | SbNFY-C3 | | 5 | | -1 | | 1 | | 19 | | 8387 | | 8405 | | UUGUUUGGAUGUUGUCGGA | | UUCAACAAUAACCAAAUAU | | Cleavage | |  | | 1 | |  |
| sbi-miR5565e | SbNFY-C12 | | 5 | | -1 | | 1 | | 19 | | 16200 | | 16218 | | UUGUUUGGAUGUUGUCGGA | | UUAGAGGACGUUUAAAUAA | | Cleavage | |  | | 1 | |  |
| sbi-miR5565f | SbNFY-C13 | | 5 | | -1 | | 1 | | 20 | | 16850 | | 16869 | | UAGUCGGAUUUAUAUCAAUC | | GAUUGAAGUAAAAAUGACUA | | Cleavage | |  | | 2 | |  |
| sbi-miR5565f | SbNFY-C1 | | 5 | | -1 | | 1 | | 20 | | 680 | | 699 | | UAGUCGGAUUUAUAUCAAUC | | ACUUAACAUGAAUCUGGCUG | | Cleavage | |  | | 2 | |  |
| sbi-miR5565f | SbNFY-C1 | | 5 | | -1 | | 1 | | 20 | | 8662 | | 8681 | | UAGUCGGAUUUAUAUCAAUC | | CAUUAACAUUAGUUUGACUA | | Translation | | | | 2 | |  |
| sbi-miR5565g-3p | SbNFY-C3 | | 5 | | -1 | | 1 | | 24 | | 947 | | 970 | | ACACAUGUGGAUUGAGAUGAAUAC | | CAAGUCAUCAAAAUCCAGAUGAGU | | Cleavage | |  | | 1 | |  |
| sbi-miR5565g-3p | SbNFY-C6 | | 5 | | -1 | | 1 | | 24 | | 5657 | | 5680 | | ACACAUGUGGAUUGAGAUGAAUAC | | AUUGUUAUGUUGGUCUAAAUGUGU | | Cleavage | |  | | 1 | |  |
| sbi-miR5565g-5p | SbNFY-C15 | | 5 | | -1 | | 1 | | 24 | | 3321 | | 3344 | | UUCACAUCAAUCCACAUAUGUUGG | | UUGAUAGAUGGGGCAUGAUGUGAA | | Translation | | | | 1 | |  |
| sbi-miR5567 | SbNFY-C11 | | 5 | | -1 | | 1 | | 24 | | 13531 | | 13554 | | UUAAUGAUUCAUGUAUGUGUCCAA | | UCAGAUGUGUACGUGAAUCAUUUC | | Cleavage | |  | | 2 | |  |
| sbi-miR5567 | SbNFY-C8 | | 5 | | -1 | | 1 | | 24 | | 2478 | | 2502 | | UUAAUGAUUCAUGUAUGUGUC-CAA | | UUGCGGAACAUGCAUUGAACAUUAA | | Translation | | | | 3 | |  |
| sbi-miR5567 | SbNFY-C1 | | 5 | | -1 | | 1 | | 24 | | 5292 | | 5315 | | UUAAUGAUUCAUGUAUGUGUCCAA | | CUGGGUGAAAAUAUGAAUUAUUAU | | Cleavage | |  | | 1 | |  |
| sbi-miR5567 | SbNFY-C14 | | 5 | | -1 | | 1 | | 24 | | 15473 | | 15496 | | UUAAUGAUUCAUGUAUGUGUCCAA | | UAUGGCAUAUGUAUGGAGCACUAA | | Cleavage | |  | | 2 | |  |
| sbi-miR5567 | SbNFY-C14 | | 5 | | -1 | | 1 | | 24 | | 15807 | | 15830 | | UUAAUGAUUCAUGUAUGUGUCCAA | | UGUGGCAUAUGUAUGGUGCAUUAA | | Cleavage | |  | | 2 | |  |
| sbi-miR5568a | SbNFY-C9 | | 5 | | -1 | | 1 | | 21 | | 10241 | | 10261 | | CAGAGCGACUUACAAUUUGGA | | UCUAAAUUAUAAGUCAUUCUA | | Cleavage | |  | | 1 | |  |
| sbi-miR5568a | SbNFY-C14 | | 5 | | -1 | | 1 | | 21 | | 5164 | | 5184 | | CAGAGCGACUUACAAUUUGGA | | UUUAAAUUAUAAAUUGUUUUG | | Cleavage | |  | | 1 | |  |
| sbi-miR5568b-5p | SbNFY-C10 | | 5 | | -1 | | 1 | | 21 | | 3481 | | 3501 | | UUUCUAGGUACAUAGCUUUUG | | AAAAUGUUGUGUACUUAGAUU | | Cleavage | |  | | 1 | |  |
| sbi-miR5568b-5p | SbNFY-C3 | | 5 | | -1 | | 1 | | 21 | | 1449 | | 1469 | | UUUCUAGGUACAUAGCUUUUG | | CAACAGGUAUGUAGCUAGAGC | | Cleavage | |  | | 2 | |  |
| sbi-miR5568b-5p | SbNFY-C11 | | 5 | | -1 | | 1 | | 21 | | 11712 | | 11732 | | UUUCUAGGUACAUAGCUUUUG | | AAUUUGCUAUGUAUCUAGACA | | Cleavage | |  | | 1 | |  |
| sbi-miR5568b-5p | SbNFY-C14 | | 5 | | -1 | | 1 | | 21 | | 1518 | | 1538 | | UUUCUAGGUACAUAGCUUUUG | | ACAACGAUAUAUACCUAGAGU | | Translation | | | | 1 | |  |
| sbi-miR5568c-3p | SbNFY-C14 | | 5 | | -1 | | 1 | | 21 | | 6295 | | 6315 | | ACUUACAGUUUGGAACGGAGG | | UCUUUAUUUUAAAUUGUAAAU | | Cleavage | |  | | 1 | |  |
| sbi-miR5568c-3p | SbNFY-C2 | | 5 | | -1 | | 1 | | 21 | | 14854 | | 14874 | | ACUUACAGUUUGGAACGGAGG | | CAUCAAUUUUGAAUUGUAAGG | | Cleavage | |  | | 1 | |  |
| sbi-miR5568d-3p | SbNFY-C15 | | 5 | | -1 | | 1 | | 21 | | 3894 | | 3914 | | AAAGUUGUGUAUCUAGAAAAG | | CUUUGUGAGAUCCACAACUUA | | Translation | | | | 2 | |  |
| sbi-miR5568d-3p | SbNFY-C15 | | 5 | | -1 | | 1 | | 21 | | 13727 | | 13747 | | AAAGUUGUGUAUCUAGAAAAG | | GAUUUCAAGAACCACAACUUG | | Translation | | | | 2 | |  |
| sbi-miR5568d-3p | SbNFY-C8 | | 5 | | -1 | | 1 | | 21 | | 11592 | | 11612 | | AAAGUUGUGUAUCUAGAAAAG | | AUUUUUUAGAUGUUCUAUUUU | | Cleavage | |  | | 1 | |  |
| sbi-miR5568d-3p | SbNFY-C10 | | 5 | | -1 | | 1 | | 21 | | 17370 | | 17390 | | AAAGUUGUGUAUCUAGAAAAG | | UAUUAUUAGGUAUAUAGAUUU | | Cleavage | |  | | 1 | |  |
| sbi-miR5568d-5p | SbNFY-C12 | | 5 | | -1 | | 1 | | 21 | | 4246 | | 4266 | | UGGCUUUUCUAGAUACAUAGC | | UCUAAGAAUCUUGGAGAGUCA | | Translation | | | | 2 | |  |
| sbi-miR5568d-5p | SbNFY-C13 | | 5 | | -1 | | 1 | | 21 | | 12039 | | 12059 | | UGGCUUUUCUAGAUACAUAGC | | UUUGUGGUUCUGCAGAAGCCA | | Cleavage | |  | | 1 | |  |
| sbi-miR5568d-5p | SbNFY-C6 | | 5 | | -1 | | 1 | | 21 | | 222 | | 242 | | UGGCUUUUCUAGAUACAUAGC | | CUUAAAUUUUUAGAGAAGCCC | | Cleavage | |  | | 1 | |  |
| sbi-miR5568e-3p | SbNFY-C14 | | 5 | | -1 | | 1 | | 21 | | 691 | | 712 | | UAUCUAGAAAAG-CUAAAACGU | | UAUUUUGAGACUUUUCUAGAUA | | Cleavage | |  | | 2 | |  |
| sbi-miR5568e-3p | SbNFY-C9 | | 5 | | -1 | | 1 | | 21 | | 7251 | | 7271 | | UAUCUAGAAAAGCUAAAACGU | | AGAUUUUUGUUUUUUUACAUG | | Cleavage | |  | | 1 | |  |
| sbi-miR5568e-3p | SbNFY-C10 | | 5 | | -1 | | 1 | | 21 | | 4080 | | 4100 | | UAUCUAGAAAAGCUAAAACGU | | UUGUAUGAAUUUUUCUAGAUU | | Cleavage | |  | | 1 | |  |
| sbi-miR5568e-5p | SbNFY-C7 | | 5 | | -1 | | 1 | | 21 | | 13010 | | 13030 | | GAUGUUUUGGGUUUUCUAGAU | | GCCUAGAAGAUCAGAAACCUU | | Cleavage | |  | | 3 | |  |
| sbi-miR5568e-5p | SbNFY-C1 | | 5 | | -1 | | 1 | | 21 | | 2418 | | 2438 | | GAUGUUUUGGGUUUUCUAGAU | | ACACAGAAAAUCCAAAACCUA | | Cleavage | |  | | 2 | |  |
| sbi-miR5568f-3p | SbNFY-C11 | | 5 | | -1 | | 1 | | 21 | | 16148 | | 16168 | | GUCUUAUAAUUUGGAAUGGAG | | UUUAGUUAUAAAUCAUAAGAC | | Cleavage | |  | | 3 | |  |
| sbi-miR5568f-3p | SbNFY-C14 | | 5 | | -1 | | 1 | | 21 | | 4855 | | 4875 | | GUCUUAUAAUUUGGAAUGGAG | | GUCCUUUUCAAAUAAUAGUAC | | Cleavage | |  | | 1 | |  |
| sbi-miR5568f-3p | SbNFY-C6 | | 5 | | -1 | | 1 | | 21 | | 15787 | | 15807 | | GUCUUAUAAUUUGGAAUGGAG | | CACCAUUAUAGAUUAUGAGUG | | Cleavage | |  | | 1 | |  |
| sbi-miR5568f-3p | SbNFY-C15 | | 5 | | -1 | | 1 | | 21 | | 7991 | | 8012 | | GUCUUAUAAUUUGGA-AUGGAG | | AUUUAUCUAUAAAUUAUAAGAU | | Cleavage | |  | | 1 | |  |
| sbi-miR5568f-3p | SbNFY-C4 | | 5 | | -1 | | 1 | | 21 | | 13231 | | 13251 | | GUCUUAUAAUUUGGAAUGGAG | | UACCCUUCCAGAUUCUAGUAC | | Cleavage | |  | | 1 | |  |
| sbi-miR5568f-5p | SbNFY-C7 | | 5 | | -1 | | 1 | | 21 | | 10099 | | 10119 | | UCCAUUCCAAAUUGUAAGAUG | | AAUUUAGAGACUUGGAAUGGA | | Translation | | | | 1 | |  |
| sbi-miR5568g-5p | SbNFY-C14 | | 5 | | -1 | | 1 | | 21 | | 6545 | | 6565 | | CAAAUUAUAAGAUGUUUUGGC | | GUCGAGACGAUUUAUAAUCUG | | Cleavage | |  | | 2 | |  |
| sbi-miR5568g-5p | SbNFY-C1 | | 5 | | -1 | | 1 | | 21 | | 1751 | | 1770 | | CAAAUUAUAAGAUGUUUUGGC | | GUCAAAACAUCUCA-AGUUUG | | Cleavage | |  | | 2 | |  |
| sbi-miR5568g-5p | SbNFY-C12 | | 5 | | -1 | | 1 | | 21 | | 4263 | | 4282 | | CAAAUUAUAAGAUGUUUUGGC | | GUCAAAACAUCUCA-AGUUUG | | Cleavage | |  | | 2 | |  |
| sbi-miR5568g-5p | SbNFY-C12 | | 5 | | -1 | | 1 | | 21 | | 12148 | | 12168 | | CAAAUUAUAAGAUGUUUUGGC | | GACAGAGAAUCUUGAAAUUUU | | Cleavage | |  | | 2 | |  |
| sbi-miR5568g-5p | SbNFY-C15 | | 5 | | -1 | | 1 | | 21 | | 1741 | | 1761 | | CAAAUUAUAAGAUGUUUUGGC | | CCCAAAGAGUGUUAUAGUUUA | | Translation | | | | 1 | |  |
| sbi-miR5568g-5p | SbNFY-C8 | | 5 | | -1 | | 1 | | 21 | | 7936 | | 7956 | | CAAAUUAUAAGAUGUUUUGGC | | UUGAAAACAGCUUAUCAUUUA | | Cleavage | |  | | 1 | |  |
| sbi-miR5569 | SbNFY-C12 | | 5 | | -1 | | 1 | | 24 | | 17560 | | 17583 | | UAUUGCAUGCUUGAACUAUGGUAA | | GUUUUAUAGUUCGGGGGUGAAGUA | | Cleavage | |  | | 2 | |  |
| sbi-miR5569 | SbNFY-C2 | | 5 | | -1 | | 1 | | 24 | | 15352 | | 15375 | | UAUUGCAUGCUUGAACUAUGGUAA | | CAGUACUCGAUUAAGCCUGCAAUA | | Cleavage | |  | | 1 | |  |
| sbi-miR5569 | SbNFY-C1 | | 5 | | -1 | | 1 | | 24 | | 1314 | | 1337 | | UAUUGCAUGCUUGAACUAUGGUAA | | UGGUGCAAGUGUAAACAUGCAAUA | | Translation | | | | 1 | |  |
| sbi-miR5570 | SbNFY-C1 | | 5 | | -1 | | 1 | | 21 | | 17022 | | 17041 | | AAAAGACAAAUCAGCAUGUCA | | GUACA-GAUAAUUUGUUUUUU | | Cleavage | |  | | 2 | |  |
| sbi-miR6217a-3p | SbNFY-C8 | | 5 | | -1 | | 1 | | 24 | | 16253 | | 16276 | | AAAAUUAUCGUAAAUAGAGGUGGC | | UUUUCGUUUGUUUGUAGUAAUUUU | | Cleavage | |  | | 1 | |  |
| sbi-miR6217a-3p | SbNFY-C12 | | 5 | | -1 | | 1 | | 24 | | 8363 | | 8386 | | AAAAUUAUCGUAAAUAGAGGUGGC | | CUUUCGUUUGUUUGUGGUAAUUCU | | Cleavage | |  | | 2 | |  |
| sbi-miR6217a-3p | SbNFY-C12 | | 5 | | -1 | | 1 | | 24 | | 17426 | | 17449 | | AAAAUUAUCGUAAAUAGAGGUGGC | | UUUUCUAUGAUUUAUUAUAAUUUU | | Cleavage | |  | | 2 | |  |
| sbi-miR6217a-3p | SbNFY-C6 | | 5 | | -1 | | 1 | | 24 | | 8365 | | 8388 | | AAAAUUAUCGUAAAUAGAGGUGGC | | AGUUGUUGUAUUGAUGUUAAUUUU | | Cleavage | |  | | 1 | |  |
| sbi-miR6217a-3p | SbNFY-C13 | | 5 | | -1 | | 1 | | 24 | | 3489 | | 3512 | | AAAAUUAUCGUAAAUAGAGGUGGC | | CAGAUCUAUAAUUUUGAUAAUUUA | | Translation | | | | 1 | |  |
| sbi-miR6217a-3p | SbNFY-C9 | | 5 | | -1 | | 1 | | 24 | | 15602 | | 15625 | | AAAAUUAUCGUAAAUAGAGGUGGC | | AAAAUUGUAAUUUAUUAUAAUUUU | | Cleavage | |  | | 1 | |  |
| sbi-miR6217b-3p | SbNFY-C8 | | 5 | | -1 | | 1 | | 24 | | 16253 | | 16276 | | AAAAUUAUCGUAAAUAGAGGUGGC | | UUUUCGUUUGUUUGUAGUAAUUUU | | Cleavage | |  | | 1 | |  |
| sbi-miR6217b-3p | SbNFY-C12 | | 5 | | -1 | | 1 | | 24 | | 8363 | | 8386 | | AAAAUUAUCGUAAAUAGAGGUGGC | | CUUUCGUUUGUUUGUGGUAAUUCU | | Cleavage | |  | | 2 | |  |
| sbi-miR6217b-3p | SbNFY-C12 | | 5 | | -1 | | 1 | | 24 | | 17426 | | 17449 | | AAAAUUAUCGUAAAUAGAGGUGGC | | UUUUCUAUGAUUUAUUAUAAUUUU | | Cleavage | |  | | 2 | |  |
| sbi-miR6217b-3p | SbNFY-C6 | | 5 | | -1 | | 1 | | 24 | | 8365 | | 8388 | | AAAAUUAUCGUAAAUAGAGGUGGC | | AGUUGUUGUAUUGAUGUUAAUUUU | | Cleavage | |  | | 1 | |  |
| sbi-miR6217b-3p | SbNFY-C13 | | 5 | | -1 | | 1 | | 24 | | 3489 | | 3512 | | AAAAUUAUCGUAAAUAGAGGUGGC | | CAGAUCUAUAAUUUUGAUAAUUUA | | Translation | | | | 1 | |  |
| sbi-miR6217b-3p | SbNFY-C9 | | 5 | | -1 | | 1 | | 24 | | 15602 | | 15625 | | AAAAUUAUCGUAAAUAGAGGUGGC | | AAAAUUGUAAUUUAUUAUAAUUUU | | Cleavage | |  | | 1 | |  |
| sbi-miR6218-3p | SbNFY-C4 | | 5 | | -1 | | 1 | | 21 | | 7201 | | 7221 | | ACAAGUUUCGUGAUUUUUGGA | | UCCAAAAAUCAUGAAAUUAAU | | Cleavage | |  | | 1 | |  |
| sbi-miR6218-3p | SbNFY-C13 | | 5 | | -1 | | 1 | | 21 | | 12657 | | 12677 | | ACAAGUUUCGUGAUUUUUGGA | | ACCGAAAUUCGCGAAAUUUCG | | Cleavage | |  | | 1 | |  |
| sbi-miR6218-3p | SbNFY-C7 | | 5 | | -1 | | 1 | | 21 | | 9116 | | 9136 | | ACAAGUUUCGUGAUUUUUGGA | | GGCAAGAAUCUCGGGGCCUGU | | Translation | | | | 1 | |  |
| sbi-miR6218-3p | SbNFY-C9 | | 5 | | -1 | | 1 | | 21 | | 3325 | | 3345 | | ACAAGUUUCGUGAUUUUUGGA | | UACAUAUAUCCCUAAACUUGU | | Translation | | | | 1 | |  |
| sbi-miR6218-5p | SbNFY-C15 | | 5 | | -1 | | 1 | | 21 | | 17693 | | 17713 | | CGAAAAUCACGAAACUUGUCG | | AGAUUCGUCUCGUGAUUUUCA | | Cleavage | |  | | 1 | |  |
| sbi-miR6218-5p | SbNFY-C9 | | 5 | | -1 | | 1 | | 21 | | 7817 | | 7837 | | CGAAAAUCACGAAACUUGUCG | | GGCUGAUAUUCGUGAUUUUCA | | Cleavage | |  | | 1 | |  |
| sbi-miR6220-3p | SbNFY-C1 | | 5 | | -1 | | 1 | | 24 | | 14471 | | 14494 | | AUGCCUUAUAAUUUGGGAUGGAGA | | CUUCUAUUCCAAAUUGUUAGAUGU | | Cleavage | |  | | 3 | |  |
| sbi-miR6220-5p | SbNFY-C14 | | 5 | | -1 | | 1 | | 24 | | 6550 | | 6573 | | CUCCAUCCUAAAUUAUAAGACAUU | | GACGAUUUAUAAUCUGAGAUGGAA | | Translation | | | | 1 | |  |
| sbi-miR6221-3p | SbNFY-C6 | | 5 | | -1 | | 1 | | 21 | | 12186 | | 12206 | | CCGGGGCCAGAUCUCAGAAGC | | UGUUCUCAUGUCUGGCUCUGA | | Cleavage | |  | | 1 | |  |
| sbi-miR6222-5p | SbNFY-C10 | | 5 | | -1 | | 1 | | 21 | | 12974 | | 12994 | | CCUGUUUGGAUCAGCCAAGGC | | UUUCUGGUUGAUCUGGAUGGG | | Cleavage | |  | | 1 | |  |
| sbi-miR6223-3p | SbNFY-C4 | | 5 | | -1 | | 1 | | 21 | | 12564 | | 12584 | | CUAGCAUGUUCCUCCUAAGAG | | AGCCUAGGAGGAGCAAGUUCG | | Cleavage | |  | | 1 | |  |
| sbi-miR6224a-3p | SbNFY-C13 | | 5 | | -1 | | 1 | | 21 | | 703 | | 723 | | CUUAUAUACUAGGACGGAGGG | | CCCUCUGUCCUAUAAUAUAGU | | Cleavage | |  | | 1 | |  |
| sbi-miR6224a-5p | SbNFY-C7 | | 5 | | -1 | | 1 | | 21 | | 16672 | | 16692 | | CUCCGUCCUAAUAUAUAAGGC | | UCUUUUUCUAUUGGGACUGGG | | Cleavage | |  | | 1 | |  |
| sbi-miR6224b-3p | SbNFY-C13 | | 5 | | -1 | | 1 | | 21 | | 703 | | 723 | | CUUAUAUACUAGGACGGAGGG | | CCCUCUGUCCUAUAAUAUAGU | | Cleavage | |  | | 1 | |  |
| sbi-miR6224b-5p | SbNFY-C7 | | 5 | | -1 | | 1 | | 21 | | 16672 | | 16692 | | CUCCGUCCUAAUAUAUAAGGC | | UCUUUUUCUAUUGGGACUGGG | | Cleavage | |  | | 1 | |  |
| sbi-miR6224c-3p | SbNFY-C13 | | 5 | | -1 | | 1 | | 21 | | 703 | | 723 | | CUUAUAUACUAGGACGGAGGG | | CCCUCUGUCCUAUAAUAUAGU | | Cleavage | |  | | 1 | |  |
| sbi-miR6224c-5p | SbNFY-C7 | | 5 | | -1 | | 1 | | 21 | | 16672 | | 16692 | | CUCCGUCCUAAUAUAUAAGGC | | UCUUUUUCUAUUGGGACUGGG | | Cleavage | |  | | 1 | |  |
| sbi-miR6225-3p | SbNFY-C5 | | 5 | | -1 | | 1 | | 24 | | 7753 | | 7776 | | GAAACGAAUCUUUUAAGUCUAAUU | | AACUAGAUUCAGAAGAUUCAUCUC | | Cleavage | |  | | 4 | |  |
| sbi-miR6225-3p | SbNFY-C12 | | 5 | | -1 | | 1 | | 24 | | 15757 | | 15780 | | GAAACGAAUCUUUUAAGUCUAAUU | | AACUAGGACCAAAAGAUUCGUCUC | | Cleavage | |  | | 5 | |  |
| sbi-miR6225-3p | SbNFY-C15 | | 5 | | -1 | | 1 | | 24 | | 9839 | | 9862 | | GAAACGAAUCUUUUAAGUCUAAUU | | AAUUAGGCUCAAAAAAUUUGUCUC | | Translation | | | | 2 | |  |
| sbi-miR6225-5p | SbNFY-C14 | | 5 | | -1 | | 1 | | 24 | | 6618 | | 6641 | | AACUAGACUCAAAAGAUUCAUCUC | | AAGCUUAAAAUUUUGAUUUUAGUU | | Cleavage | |  | | 3 | |  |
| sbi-miR6225-5p | SbNFY-C12 | | 5 | | -1 | | 1 | | 24 | | 10942 | | 10965 | | AACUAGACUCAAAAGAUUCAUCUC | | CACCAAAAUCUUUUGGUUUUGGUG | | Cleavage | |  | | 2 | |  |
| sbi-miR6228-3p | SbNFY-C1 | | 5 | | -1 | | 1 | | 24 | | 11791 | | 11814 | | GUGGCAGUAGAAUUAAUGAAGGGA | | AUCUAGGUUUGAUUCUGUUGCCAU | | Cleavage | |  | | 1 | |  |
| sbi-miR6228-3p | SbNFY-C2 | | 5 | | -1 | | 1 | | 24 | | 8069 | | 8092 | | GUGGCAGUAGAAUUAAUGAAGGGA | | GGAAAUCUAUGAUUCCAUUGCCAU | | Cleavage | |  | | 1 | |  |
| sbi-miR6228-3p | SbNFY-C9 | | 5 | | -1 | | 1 | | 24 | | 1667 | | 1690 | | GUGGCAGUAGAAUUAAUGAAGGGA | | CAAUGUCAUUUGCUCUGUUGCCAA | | Cleavage | |  | | 1 | |  |
| sbi-miR6228-3p | SbNFY-C8 | | 5 | | -1 | | 1 | | 24 | | 13792 | | 13815 | | GUGGCAGUAGAAUUAAUGAAGGGA | | GACUACCAUUGCUUCUACUGAUAC | | Cleavage | |  | | 2 | |  |
| sbi-miR6228-3p | SbNFY-C8 | | 5 | | -1 | | 1 | | 24 | | 16625 | | 16648 | | GUGGCAGUAGAAUUAAUGAAGGGA | | GGUCGACGCUGAUGCUGCUGCCAC | | Translation | | | | 2 | |  |
| sbi-miR6228-3p | SbNFY-C12 | | 5 | | -1 | | 1 | | 24 | | 6418 | | 6441 | | GUGGCAGUAGAAUUAAUGAAGGGA | | GAGUACCAUUGCUUCUACUGAUAC | | Cleavage | |  | | 1 | |  |
| sbi-miR6228-3p | SbNFY-C6 | | 5 | | -1 | | 1 | | 24 | | 5892 | | 5916 | | GUGGCAGUAGAAUUA-AUGAAGGGA | | AAACCUCAUAUUAUUCUGUUGUCAU | | Cleavage | |  | | 1 | |  |
| sbi-miR6228-5p | SbNFY-C4 | | 5 | | -1 | | 1 | | 24 | | 11345 | | 11368 | | UUCUAUCUCUAUUAAUUGUGUUGC | | AUUGAACAUUAAAUAUAGAUAAAA | | Cleavage | |  | | 1 | |  |
| sbi-miR6228-5p | SbNFY-C9 | | 5 | | -1 | | 1 | | 24 | | 10291 | | 10314 | | UUCUAUCUCUAUUAAUUGUGUUGC | | GUUUGACAAAAAAUAUAGAGAGAA | | Cleavage | |  | | 1 | |  |
| sbi-miR6229-3p | SbNFY-C4 | | 5 | | -1 | | 1 | | 24 | | 1015 | | 1038 | | GUUUUUCUCGCCGGGUGAGAAGGC | | CUGGCUUCAUCAGCCGAGAAAGAU | | Translation | | | | 1 | |  |
| sbi-miR6229-5p | SbNFY-C1 | | 5 | | -1 | | 1 | | 24 | | 16599 | | 16622 | | AUUCUCACUUGGGCGACGGAAAGG | | UCUCUCCACCGCCUGAGUGAGGUU | | Cleavage | |  | | 1 | |  |
| sbi-miR6230-3p | SbNFY-C6 | | 5 | | -1 | | 1 | | 21 | | 3493 | | 3513 | | UAACAAGUUUAGGGAUCUAGA | | CACUGGUCCCUAAACUUGUAC | | Cleavage | |  | | 4 | |  |
| sbi-miR6230-3p | SbNFY-C3 | | 5 | | -1 | | 1 | | 21 | | 212 | | 232 | | UAACAAGUUUAGGGAUCUAGA | | ACUGGCUCUCUAAAUUAGUUC | | Cleavage | |  | | 2 | |  |
| sbi-miR6230-3p | SbNFY-C5 | | 5 | | -1 | | 1 | | 21 | | 13201 | | 13221 | | UAACAAGUUUAGGGAUCUAGA | | CUGGGAACUUUUAACUUGUUA | | Translation | | | | 1 | |  |
| sbi-miR6230-5p | SbNFY-C6 | | 5 | | -1 | | 1 | | 21 | | 4133 | | 4153 | | UUUUGGGUCCCUAAACUUGUU | | AACAAGUUUGAGGACCUAUAU | | Translation | | | | 4 | |  |
| sbi-miR6230-5p | SbNFY-C6 | | 5 | | -1 | | 1 | | 21 | | 3915 | | 3935 | | UUUUGGGUCCCUAAACUUGUU | | AACAUAUUUUGGGACCCUAAA | | Cleavage | |  | | 4 | |  |
| sbi-miR6230-5p | SbNFY-C6 | | 5 | | -1 | | 1 | | 21 | | 4222 | | 4242 | | UUUUGGGUCCCUAAACUUGUU | | UGCGAGUUUAAGGACUGAAAU | | Translation | | | | 4 | |  |
| sbi-miR6230-5p | SbNFY-C15 | | 5 | | -1 | | 1 | | 21 | | 1178 | | 1198 | | UUUUGGGUCCCUAAACUUGUU | | AUAAAGUUUUGGCACCCAAAC | | Cleavage | |  | | 1 | |  |
| sbi-miR6231-3p | SbNFY-C2 | | 5 | | -1 | | 1 | | 21 | | 702 | | 722 | | UAUUUGUGGACUCAUGGACAU | | UAGCCCUUGGGGCUAUAAAUA | | Translation | | | | 1 | |  |
| sbi-miR6232a-5p | SbNFY-C1 | | 5 | | -1 | | 1 | | 24 | | 15648 | | 15671 | | GUCGCUUUGACUUUUUUGGUACAU | | AUGUGCUAAAAGAUUCGAUGUGAC | | Translation | | | | 1 | |  |
| sbi-miR6232a-5p | SbNFY-C13 | | 5 | | -1 | | 1 | | 24 | | 12803 | | 12826 | | GUCGCUUUGACUUUUUUGGUACAU | | CCAGCUCAGAAAGGUUAACGAGAC | | Cleavage | |  | | 1 | |  |
| sbi-miR6232a-5p | SbNFY-C5 | | 5 | | -1 | | 1 | | 24 | | 13667 | | 13690 | | GUCGCUUUGACUUUUUUGGUACAU | | GUUACCCCAAGAACUCAAAGUCAC | | Translation | | | | 1 | |  |
| sbi-miR6232b-5p | SbNFY-C1 | | 5 | | -1 | | 1 | | 21 | | 5811 | | 5831 | | UUUUUGGUACAUUGAAUUUGC | | AGAGCUAUAGUGUACAAAAAA | | Cleavage | |  | | 3 | |  |
| sbi-miR6232b-5p | SbNFY-C7 | | 5 | | -1 | | 1 | | 21 | | 268 | | 288 | | UUUUUGGUACAUUGAAUUUGC | | GCUAGUUUAAUGGACGAAAAA | | Cleavage | |  | | 1 | |  |
| sbi-miR6232b-5p | SbNFY-C11 | | 5 | | -1 | | 1 | | 21 | | 2617 | | 2637 | | UUUUUGGUACAUUGAAUUUGC | | GCAAAAUAGAUAUAUUAAAAA | | Translation | | | | 4 | |  |
| sbi-miR6232b-5p | SbNFY-C5 | | 5 | | -1 | | 1 | | 21 | | 7223 | | 7243 | | UUUUUGGUACAUUGAAUUUGC | | UCUUGUUUAGUUUACCAAAAA | | Translation | | | | 1 | |  |
| sbi-miR6232b-5p | SbNFY-C13 | | 5 | | -1 | | 1 | | 21 | | 10560 | | 10580 | | UUUUUGGUACAUUGAAUUUGC | | AAGAGUUUAAAGUACCAGAGC | | Translation | | | | 2 | |  |
| sbi-miR6232b-5p | SbNFY-C8 | | 5 | | -1 | | 1 | | 21 | | 16387 | | 16407 | | UUUUUGGUACAUUGAAUUUGC | | AAAGAUUUGAUGUGACGGAGA | | Cleavage | |  | | 1 | |  |
| sbi-miR6232b-5p | SbNFY-C2 | | 5 | | -1 | | 1 | | 21 | | 11148 | | 11168 | | UUUUUGGUACAUUGAAUUUGC | | AAAGAUUCGAUGUGACGGGGA | | Cleavage | |  | | 1 | |  |
| sbi-miR6232b-5p | SbNFY-C6 | | 5 | | -1 | | 1 | | 21 | | 8381 | | 8401 | | UUUUUGGUACAUUGAAUUUGC | | UUAAUUUUGAUGGACCAAAUA | | Cleavage | |  | | 1 | |  |
| sbi-miR6232b-5p | SbNFY-C9 | | 5 | | -1 | | 1 | | 21 | | 16716 | | 16736 | | UUUUUGGUACAUUGAAUUUGC | | GAUGAUCCAGUGUAGCAAAGA | | Cleavage | |  | | 1 | |  |
| sbi-miR6233-3p | SbNFY-C4 | | 5 | | -1 | | 1 | | 24 | | 7259 | | 7282 | | CAAGUUUGGUUUUGGUAAUUAAUG | | AAAGAAUUAUUGAAAUCAGAGUUA | | Cleavage | |  | | 3 | |  |
| sbi-miR6233-3p | SbNFY-C4 | | 5 | | -1 | | 1 | | 24 | | 9507 | | 9530 | | CAAGUUUGGUUUUGGUAAUUAAUG | | UGCCUAUUGCCAAAGCCUAACUCA | | Cleavage | |  | | 3 | |  |
| sbi-miR6233-3p | SbNFY-C4 | | 5 | | -1 | | 1 | | 24 | | 8098 | | 8121 | | CAAGUUUGGUUUUGGUAAUUAAUG | | GAGACGUUACAAAAGCUUAACUUA | | Cleavage | |  | | 3 | |  |
| sbi-miR6233-3p | SbNFY-C15 | | 5 | | -1 | | 1 | | 24 | | 426 | | 448 | | CAAGUUUGGUUUUGGUAAUUAAUG | | CAACAAUCAC-AAAACCAUAUUUG | | Cleavage | |  | | 1 | |  |
| sbi-miR6233-5p | SbNFY-C11 | | 5 | | -1 | | 1 | | 24 | | 13811 | | 13834 | | UGUUGAGGCUGGAGCGAAACUCGG | | GUUCGUUUCCCUUCAGCUCCAGCU | | Cleavage | |  | | 1 | |  |
| sbi-miR6233-5p | SbNFY-C15 | | 5 | | -1 | | 1 | | 24 | | 5259 | | 5282 | | UGUUGAGGCUGGAGCGAAACUCGG | | AAUUCUUGUGUUCCAGCCUAAAGA | | Cleavage | |  | | 2 | |  |
| sbi-miR6233-5p | SbNFY-C15 | | 5 | | -1 | | 1 | | 24 | | 1612 | | 1634 | | UGUUGAGGCUGGAGCGAAACUCGG | | UGUCCUUUUUCUCUAGC-UCAACA | | Cleavage | |  | | 2 | |  |
| sbi-miR6234a-5p | SbNFY-C10 | | 5 | | -1 | | 1 | | 24 | | 749 | | 772 | | AAGUGUGUUCCUCUAUUUGACGCU | | AUUGUUAAACAGAGGAGCAUACAA | | Cleavage | |  | | 1 | |  |
| sbi-miR6234b-5p | SbNFY-C10 | | 5 | | -1 | | 1 | | 24 | | 749 | | 772 | | AAGUGUGUUCCUCUAUUUGACGCU | | AUUGUUAAACAGAGGAGCAUACAA | | Cleavage | |  | | 1 | |  |
| sbi-miR6235-3p | SbNFY-C4 | | 5 | | -1 | | 1 | | 24 | | 1388 | | 1411 | | AACGAACAGUAUUUUUCUCUUACA | | UAUGAAAGAAAAAUAUUAUUUCUU | | Cleavage | |  | | 1 | |  |
| sbi-miR821d | SbNFY-C2 | | 5 | | -1 | | 1 | | 21 | | 4634 | | 4653 | | AAGUCAUCAACAACAAAGUUG | | AUGCUUUGUUGUAGAU-ACUU | | Cleavage | |  | | 1 | |  |
| sbi-miR821d | SbNFY-C1 | | 5 | | -1 | | 1 | | 21 | | 11888 | | 11908 | | AAGUCAUCAACAACAAAGUUG | | GUUCUAUUCUGUUGAUGAUUU | | Cleavage | |  | | 1 | |  |
| sbi-miR821e | SbNFY-C7 | | 5 | | -1 | | 1 | | 21 | | 537 | | 557 | | AAGUCAUCAAAAUAAAAGUUG | | AUAUUUUUCUUUUGGUGACAG | | Cleavage | |  | | 1 | |  |
| sbi-miR821e | SbNFY-C12 | | 5 | | -1 | | 1 | | 21 | | 9660 | | 9680 | | AAGUCAUCAAAAUAAAAGUUG | | UAAUUAUUAUUUUUAUUAUUU | | Cleavage | |  | | 1 | |  |
| sbi-miR821e | SbNFY-C13 | | 5 | | -1 | | 1 | | 21 | | 3491 | | 3511 | | AAGUCAUCAAAAUAAAAGUUG | | GAUCUAUAAUUUUGAUAAUUU | | Cleavage | |  | | 1 | |  |
