# Supplementary material for: Genome-wide identification and expression profile analysis of nuclear factor Y family genes in Sorghum bicolor L. (Moench)
Source: PLoS One. 2019 Sep 19;14(9):e0222203. doi: 10.1371/journal.pone.0222203 (PMC6752760; doi:10.1371/journal.pone.0222203)
Supplement: S14 Table — (DOC) [file pone.0222203.s022.doc]

| **Genes**  **S14 Table.**Native and relative **expression analysis of SbNFYs** | **Tissues and stresses** | | | | | | | | | | | | | | | | | |
| --- | --- | --- | --- | --- | --- | --- | --- | --- | --- | --- | --- | --- | --- | --- | --- | --- | --- | --- |
|  | **R** | **S** | **L** | **MR** | **SR** | **HR** | **CR** | **ABAR** | **MS** | **SS** | **HS** | **CS** | **ABAS** | **ML** | **SL** | **HL** | **CL** | **ABAL** |
| **SbNFY-A1** | 0.3748 | 0.4719 | 2.224 | 5.199 | 9.464 | 3.077 | 0.8665 | 0.5949 | 1.446 | 8.669 | 2.283 | 0.3453 | 1.696 | 1.132 | 0.2995 | 0.8531 | 0.4108 | 1.738 |
| **SbNFY-A2** | 0.1997 | 1.246 | 0.2787 | 4.469 | 9.264 | 6.151 | 6.476 | 3.09 | 0.4166 | 0.757 | 2.451 | 2.251 | 0.3842 | 14.81 | 1.008 | 6.205 | 2.947 | 9.63 |
| **SbNFY-A3** | 0.9876 | 0.4399 | 0.829 | 0.656 | 1.196 | 3.555 | 1.655 | 0.3303 | 1.287 | 2.986 | 1.861 | 1.339 | 1.65 | 1.425 | 0.3538 | 9.77 | 1.927 | 2.003 |
| **SbNFY-A4** | 0.7312 | 0.2106 | 0.3437 | 1.63 | 0.9101 | 4.136 | 5.716 | 0.4005 | 3.628 | 3.595 | 6.754 | 12.65 | 1.485 | 2.825 | 0.5475 | 58.01 | 5.576 | 3.911 |
| **SbNFY-A5** | 0.9101 | 0.3033 | 0.3249 | 3.007 | 1.081 | 6.165 | 5.67 | 0.2342 | 3.462 | 2.719 | 2.825 | 1.149 | 3.149 | 4.826 | 0.3358 | 20.19 | 3.219 | 7.111 |
| **SbNFY-A6** | 0.3193 | 0.0638 | 0.05522 | 0.06143 | 8.599 | 1419 | 4.627 | 0.9234 | 9.312 | 3.478 | 12.74 | 22.33 | 8.985 | 31.41 | 29.41 | 39.8 | 16.6 | 31.78 |
| **SbNFY-A7** | 0.7187 | 0.2375 | 1.03 | 0.5494 | 1.094 | 141.7 | 0.2212 | 0.8341 | 2.57 | 15.52 | 0.7466 | 1.829 | 4.547 | 1.341 | 0.2681 | 6.849 | 2.187 | 1.616 |
| **SbNFY-A8** | 0.8454 | 0.3178 | 0.3667 | 0.1533 | 0.8015 | 25.02 | 13.15 | 3.109 | 0.7191 | 2.156 | 1.916 | 5.346 | 1.542 | 5.829 | 0.2929 | 11.4 | 9.361 | 1.952 |
| **SbNFY-B1** | 3.008 | 0.2586 | 2.884 | 3.401 | 0.04878 | 1.154 | 31.2 | 0.06009 | 0.07188 | 43.1 | 3.747 | 2.489 | 0.3523 | 0.02223 | 1.008206 | 0.2227 | 0.1209 | 42.96 |
| **SbNFY-B2** | 0.1703 | 1.281 | 0.5573 | 8.569 | 13.09 | 13.22 | 2.066 | 1.523 | 1.301 | 1.46 | 2.012 | 0.7014 | 0.4068 | 2.485 | 0.2849 | 9.838 | 0.9704 | 9 |
| **SbNFY-B3** | 1.536 | 0.02856 | 0.876 | 10.57 | 1.137 | 0.247 | 3.367 | 0.2283 | 162.2 | 6.778 | 0.6544 | 12.4 | 8.5 | 0.5609 | 0.1198 | 1.289 | 1.827 | 22.75 |
| **SbNFY-B4** | 0.7194 | 0.5837 | 0.7071 | 1.937 | 1.551 | 5.342 | 0.5574 | 0.7561 | 1.256 | 0.2949 | 6.638 | 4.143 | 0.5586 | 3.521 | 0.1747 | 17.49 | 1.124 | 3.133 |
| **SbNFY-B5** | 0.8425 | 0.4636 | 1.025 | 0.3331 | 1.343 | 1.532 | 0.4852 | 28.57 | 0.5963 | 2.609 | 3.092 | 1.864 | 1.143 | 0.8987 | 0.2358 | 7.115 | 1.174 | 1.412 |
| **SbNFY-B6** | 0.6756 | 0.2365 | 1.075 | 1.907 | 2.068 | 4.84 | 0.3431 | 1.182 | 4.175 | 1.543 | 28.81 | 7.745 | 0.7645 | 3.157 | 0.1338 | 8.866 | 0.5084 | 2.403 |
| **SbNFY-B7** | 0.05012 | 1.109 | 0.1748 | 39.65 | 34.14 | 705.5 | 75.93 | 4.397 | 0.4159 | 0.8255 | 7.799 | 0.8057 | 1.08348 | 0.6705 | 8.82 | 209.1 | 0.7316 | 22.82 |
| **SbNFY-B8** | 2.479 | 1.297 | 16.45 | 0.1947 | 0.1225 | 0.2557 | 2.268 | 1.03688 | 0.8822 | 1.02897 | 10.95 | 0.3341 | 0.0842 | 1.01487 | 1.02953 | 0.4178 | 0.2065 | 0.3004 |
| **SbNFY-B9** | 1.706 | 0.2007 | 0.3259 | 0.4841 | 0.1979 | 2.487 | 1.116 | 0.2408 | 1.801 | 8.49 | 5.075 | 2.421 | 3.918 | 7.366 | 0.6321 | 21.76 | 4.697 | 23.1 |
| **SbNFY-B10** | 0.8283 | 0.2265 | 1.377 | 1.033 | 0.6078 | 5.819 | 2.959 | 0.4204 | 4.322 | 1.122 | 17.95 | 13.63 | 1.516 | 1.374 | 0.05435 | 9.372 | 0.3235 | 1.818 |
| **SbNFY-B11** | 0.971 | 0.545 | 1.138 | 0.4601 | 0.7228 | 2.729 | 30.5 | 0.4514 | 0.336 | 1.243 | 0.6388 | 0.7479 | 0.3654 | 0.9175 | 0.4171 | 7.612 | 1.035 | 0.7645 |
| **SbNFY-B12** | 0.07294 | 0.266 | 2.149 | 20.03 | 19.2 | 4.002 | 43.41 | 10.83 | 3.486 | 0.3987 | 43.74 | 36.53 | 0.02725 | 0.1288 | 0.1594 | 0.6499 | 2.942 | 3.144 |
| **SbNFY-B13** | 1.003 | 0.7407 | 0.8073 | 2.135 | 1.8 | 7.336 | 0.3039 | 0.9738 | 1.241 | 0.5064 | 1.826 | 2.413 | 0.2143 | 2.696 | 0.1369 | 9.006 | 0.5255 | 2.492 |
| **SbNFY-B14** | 0.2802 | 0.5556 | 1.256 | 4.893 | 5.979 | 10.96 | 3.94 | 0.6544 | 1.16 | 0.1085 | 10.82 | 11.94 | 0.5026 | 7.679 | 1.01729 | 12.37 | 0.438 | 3.681 |
| **SbNFY-B15** | 2.04 | 0.005911 | 1.259 | 0.0692 | 1.239 | 1.437 | 25.28 | 1.959 | 29.9 | 35.5 | 1055 | 50.42 | 40.2 | 0.9482 | 1.02994 | 1.447 | 0.02595 | 2.789 |
| **SbNFY-B16** | 0.4145 | 0.351 | 0.2493 | 6.502 | 11.37 | 13.52 | 18.87 | 0.4564 | 6.028 | 0.08952 | 37.23 | 42.4 | 5.483 | 7.005 | 1.009912 | 2.622 | 0.6391 | 43.86 |
| **SbNFY-B17** | 0.5541 | 1.255 | 0.5769 | 0.2978 | 23.95 | 4.694 | 10.41 | 1.65 | 0.2555 | 0.4733 | 0.2893 | 1.709 | 0.1497 | 2.533 | 0.8817 | 8.624 | 1.764 | 1.373 |
| **SbNFY-B18** | 1.412 | 1.074 | 0.7981 | 0.3681 | 0.3796 | 0.4488 | 0.5446 | 0.1266 | 0.4031 | 0.5371 | 1.379 | 0.9417 | 0.4609 | 2.981 | 0.7676 | 6.502 | 15.69 | 2.433 |
| **SbNFY-B19** | 0.4837 | 0.1742 | 0.537 | 1.821 | 5.069 | 1.114 | 18 | 0.7474 | 4.729 | 6.014 | 7.34 | 3.995 | 5.637 | 4.629 | 0.5418 | 16.37 | 4.012 | 1.087 |
| **SbNFY-C1** | 1.088 | 2.991 | 1.6262 | 0.5943 | 1.045 | 0.3481 | 1.914 | 1.536 | 0.06356 | 0.1886 | 0.1277 | 0.5503 | 0.1212 | 1.887 | 1.609 | 17.1 | 3.287 | 1.259 |
| **SbNFY-C2** | 0.3597 | 0.0871 | 4.471 | 7.89 | 0.3168 | 3.167 | 2.549 | 0.8409 | 0.9587 | 2.175 | 18.97 | 29.67 | 25.68 | 0.8414 | 0.02419 | 0.9033 | 0.0462 | 30 |
| **SbNFY-C3** | 0.8921 | 0.3569 | 0.6565 | 0.8477 | 0.9988 | 2.049 | 2.245 | 0.7587 | 0.939 | 1.709 | 7.679 | 2.413 | 2.554 | 1.766 | 0.4266 | 18.01 | 0.8961 | 4.392 |
| **SbNFY-C4** | 0.5431 | 0.6556 | 0.9092 | 3.352 | 6.494 | 6.335 | 4.675 | 2.746 | 0.3274 | 2.494 | 4.479 | 3.954 | 0.1044 | 1.94 | 0.2639 | 5.199 | 0.2935 | 0.4161 |
| **SbNFY-C5** | 0.8884 | 0.361 | 0.5913 | 0.6628 | 1.854 | 0.5044 | 5.528 | 0.3996 | 0.3467 | 3.582 | 1.611 | 4.037 | 1.039 | 37.16 | 0.454 | 17.61 | 4.329 | 1.454 |
| **SbNFY-C6** | 0.4334 | 0.7201 | 0.1005 | 1.45 | 3.411 | 5.673 | 51.03 | 0.5334 | 1.15 | 40.39 | 4.743 | 2.083 | 0.7182 | 17.16 | 0.3272 | 14.13 | 1.16 | 45.33 |
| **SbNFY-C7** | 3.774 | 0.4239 | 0.9717 | 0.07948 | 0.2404 | 0.2867 | 0.4606 | 0.2072 | 2.373 | 2.715 | 1.122 | 1.089 | 1.338 | 0.2709 | 0.9466 | 23.48 | 1.261 | 1.696 |
| **SbNFY-C8** | 1.286 | 0.3631 | 0.7778 | 0.693 | 0.8706 | 3.208 | 2.875 | 0.3133 | 0.6458 | 2.673 | 1.785 | 1.293 | 1.955 | 1.11 | 1.032 | 8.374 | 1.497 | 2.231 |
| **SbNFY-C9** | 3.494 | 1.2373 | 2.094 | 0.3048 | 0.6678 | 2.172 | 1.049 | 0.2007 | 4.436 | 1.03078 | 16.81 | 4.962 | 1.033 | 2.008 | 1.007306 | 7.214 | 0.2364 | 1.331 |
| **SbNFY-C10** | 2.511 | 0.841 | 0.1084 | 0.5998 | 0.5328 | 4.936 | 0.06143 | 2.923 | 1.353 | 1.07902 | 12.74 | 8.959 | 0.5434 | 28.46 | 1.07998 | 40.9 | 0.4997 | 8.098 |
| **SbNFY-C11** | 0.1846 | 1.6 | 0.1849 | 1.655 | 8.959 | 76.11 | 3.882 | 0.401 | 0.08017 | 1.08936 | 9.275 | 4.597 | 0.5294 | 77.48 | 0.6354 | 25.1 | 2.21 | 4.773 |
| **SbNFY-C12** | 0.1407 | 0.3216 | 0.2045 | 2.94 | 22.55 | 26.57 | 23.24 | 6.151 | 1.549 | 0.3726 | 37.16 | 71.55 | 5.028 | 11.3 | 0.7051 | 7.679 | 0.7747 | 17.27 |
| **SbNFY-C13** | 0.208 | 2.288 | 0.3877 | 0.43 | 1.884 | 42.84 | 63.78 | 2.071 | 0.3449 | 1.01333 | 7.066 | 3.813 | 0.1512 | 1.427 | 0.1297 | 73.43 | 3.915 | 5.54 |
| **SbNFY-C14** | 0.1479 | 2.891 | 0.1409 | 45.23 | 35.59 | 22.9 | 0.573 | 14.14 | 1.59 | 1.01621 | 4.768 | 1.509 | 1.01058 | 43.59 | 0.3261 | 9.053 | 1.062 | 26.77 |
| **SbNFY-C15** | 1.729 | 1.08372 | 4.654 | 0.09841 | 0.07175 | 2.89 | 8.196 | 1.558 | 3.888 | 23.4 | 14.94 | 3.49 | 52.44 | 0.09695 | 1.006539 | 0.7742 | 1.635 | 0.2055 |

(R; Root, S; Stem, L; Leaf, MR; Mannitol Root, SR; Salt Root, HR; High temperature Root, CR; Cold Root, ABAR; Abscisic Acid Root, MS; Mannitol Stem, SS; Salt Stem, HS; High temperature Stem, CS; Cold Stem, ABAS; Abscisic Acid Stem, ML; Mannitol Leaf, SL; Salt Leaf, HL; High temperature Leaf, CL; Cold Leaf, ABAL; Abscisic Acid Leaf)
